# Evolutionary genomics of plant genes encoding N-terminal-TM-C2 domain proteins and the similar FAM62 genes and synaptotagmin genes of metazoans

**DOI:** 10.1186/1471-2164-8-259

**Published:** 2007-07-31

**Authors:** Molly Craxton

**Affiliations:** 1 Medical Research Council Laboratory of Molecular Biology, Hills Road, Cambridge CB2 0QH, UK

## Abstract

**Background:**

Synaptotagmin genes are found in animal genomes and are known to function in the nervous system. Genes with a similar domain architecture as well as sequence similarity to synaptotagmin C2 domains have also been found in plant genomes. The plant genes share an additional region of sequence similarity with a group of animal genes named *FAM62. FAM62 *genes also have a similar domain architecture. Little is known about the functions of the plant genes and animal *FAM62 *genes. Indeed, many members of the large and diverse *Syt *gene family await functional characterization. Understanding the evolutionary relationships among these genes will help to realize the full implications of functional studies and lead to improved genome annotation.

**Results:**

I collected and compared plant *Syt*-like sequences from the primary nucleotide sequence databases at NCBI. The collection comprises six groups of plant genes conserved in embryophytes: *NTMC2Type1 *to *NTMC2Type6*. I collected and compared metazoan *FAM62 *sequences and identified some similar sequences from other eukaryotic lineages. I found evidence of RNA editing and alternative splicing. I compared the intron patterns of *Syt *genes. I also compared Rabphilin and Doc2 genes.

**Conclusion:**

Genes encoding proteins with N-terminal-transmembrane-C2 domain architectures resembling synaptotagmins, are widespread in eukaryotes. A collection of these genes is presented here. The collection provides a resource for studies of intron evolution. I have classified the collection into homologous gene families according to distinctive patterns of sequence conservation and intron position. The evolutionary histories of these gene families are traceable through the appearance of family members in different eukaryotic lineages. Assuming an intron-rich eukaryotic ancestor, the conserved intron patterns distinctive of individual gene families, indicate independent origins of *Syt*, *FAM62 *and *NTMC2 *genes. Resemblances among these large, multi-domain proteins are due not only to shared ancestry (homology) but also to convergent evolution (analogy). During the evolution of these gene families, duplications and other gene rearrangements affecting domain composition, have occurred along with sequence divergence, leading to complex family relationships with accordingly complex functional implications. The functional homologies and analogies among these genes remain to be established empirically.

## Background

Synaptotagmins (Syts) share a common structure: an N-terminal transmembrane (TM) sequence followed by a variable length linker and two tandem, distinctly conserved C2 domains, C2A and C2B. Syt1 [1] identified as a protein component of synaptic vesicles, is known to be required for nervous system function, acting crucially in the fast, synchronous component of calcium regulated synaptic vesicle exocytosis [2]. Genomic analysis of *Syt *genes [3,4] indicates that animal genomes encode diverse sets of *Syt *genes but always maintain a *Syt1 *orthologue. Although it is likely that *Syt1 *orthologues function similarly [2,5-8] the functions of the other *Syt *genes, in different species, still remain to be established. The complexity of this task increases with the number of *Syt *genes and these increase along with organism complexity. The first study of the full set of *Syt *genes in a model organism [9] indicated that only *Syt1 *is expressed on synaptic vesicles. The other *Syt *genes were found to be expressed in different and distinct places. Many studies using different mammalian *Syt *genes, indicate tissue distributions which are primarily neural eg. [[10,11] and references therein]. Naturally occurring, cell type-specific expression patterns have, however, rarely been described eg. [[7,9,12,13] and references therein]. The discovery of genes in plants which are similar to *Syt *genes [3,4,14] further complicates functional predictions. While the plant genes and another group of animal genes (*FAM62*) share similarity with *Syt *genes, little is known about their functions. A preliminary biochemical analysis of proteins from the human *FAM62 *gene family has just been published [15] but growing speculation about the plant genes [16-18] necessitates a more detailed description of their similarities and differences which could usefully inform future functional studies. I have made use of the abundance of recently deposited nucleotide sequences from a wide range of organisms, to carry out a comparative genomics analysis of these genes, in order to shed light on their evolutionary relationships.

## Results

### Collection of plant gene sequences

In order to undertake a comparative analysis of the plant *Syt*-like genes, I collected and compared full-length homologues from an evolutionary range of plants. In order to perform an unbiased search for as many homologues of these relatively unknown genes as possible, I looked at all of the primary nucleotide sequence data in the NCBI sequence databases [19]. This information is fragmentary, little of it being in the form of complete sequences, either of transcripts or genomes. By far the most abundant source of new plant sequences are ESTs, but these represent particularly small fragments and their sequences are not determined to high accuracy. I therefore needed to gather sets of overlapping ESTs to find full-length gene sequences. In order to focus the search to the detection of genuinely homologous sequences, I used nucleotide sequence probes of plant sequences already identified. Only those database sequences closely related to the probe sequence would be identified in a given search. These matching sequences were added to the collection and joined to any overlapping sequences already present in the collection. Reiterated searches served to expand the collection and extend the length of gene fragments. Had I used amino acid sequence probes to search for homologues of these genes, I would have detected a wider range of fragments with amino acid similarity, but these would not necessarily be homologous. Overlapping nucleotide sequences would be required in any case, to piece together whole genes from the identified EST fragments, so the simplest strategy to gather full-length relatives of these genes was to use nucleotide probes. I avoided gathering processed sequences in the sequence databases: these include genes predicted from genome annotation pipelines, as well as the vast majority of amino acid sequences which are predicted from nucleotide sequences. These sequences may not be accurate and could mislead subsequent analyses if used without verification.

So I carried out reiterated rounds of blastn searching of nucleotide sequences at NCBI [19]. In the first few rounds, I used probes representing the plant gene coding sequences I had already identified (genes 85 to 117) [4]. After each round, I collected all of the statistically significant hits with high scoring segments longer than 30 nucleotides and assembled these sequences into a gap4 database [20]. Repeated searching with different probes, followed by gap4 assembly of only previously uncollected hits, allowed me to gradually but efficiently build a comprehensive collection. Each probe detected a unique spectrum of homologous plant sequences. Probes from a given species could be used to find similar sequences from related species. Probes covering more conserved regions could be used to find sequences from a wider range of relatives. Sequences from closely related species could be used to bridge non-overlapping contigs from a single species. In the later stages of the collection process, I carefully separated the contigs so that in most cases, each represents a set of overlapping sequences from one species only. As a final step, to ensure that the collection was as comprehensive as it could be at this time, I searched the nucleotide sequences at NCBI using tblastn with amino acid sequence probes and confirmed that the top scoring hits had already been collected.

As well as examining transcript sequences, I also collected genomic sequences where available. I particularly wanted to examine the genome of *Physcomitrella patens *which is currently being sequenced [21]. I had previously identified *Syt*-like genes in the genome sequences of *Arabidopsis thaliana *and *Oryza sativa *but both of these represent relatively recently evolved angiosperms whereas the moss genome represents an ancient bryophyte. I used the trace archive at NCBI [22] as well as resources at PHYSCObase [23] where transcript sequences are also available. I confirmed the genomic and transcript sequences from several *Physcomitrella patens *gene loci and deposited these sequences in the public databases [EMBL:AM140045, EMBL: AM140046, EMBL: AM140047, EMBL: AM140048, EMBL: AM140049, EMBL: AM140050]. In contrast to animal *Syt *genes, which appear to increase in number along with organism complexity [4], I found that the haploid genome of *Physcomitrella patens *has even more of these plant genes (19 or more) than either *Oryza sativa *(13) or *Arabidopsis thaliana *(11). Additional file 1 lists full details of each gene identified. Additional file 2 lists alphabetically, in rough phylogenetic order, all of the plant species in which genes in this collection have been identified. Genes were identified in a wide evolutionary range of land plants, from bryophytes to rosids.

### Analysis of full-length plant genes

**Figure 1:**
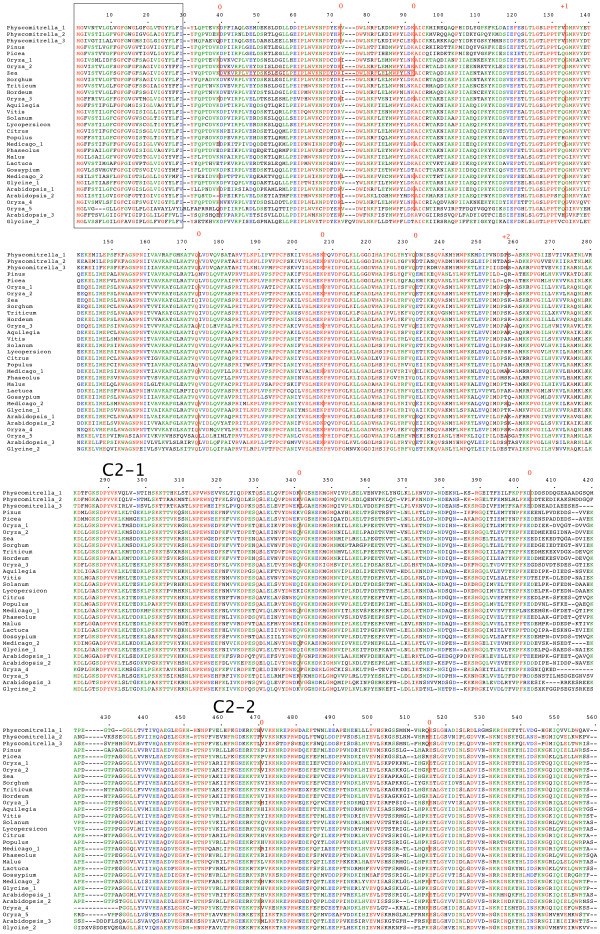
**plant *NTMType1 *genes**. Amino acid sequences of full-length gene products are aligned. The N-terminal TM region is boxed. Intron positions and phases are marked. C2 domains are indicated. Alternatively spliced regions are boxed. Full details are in additional file 1.

**Figure 2:**
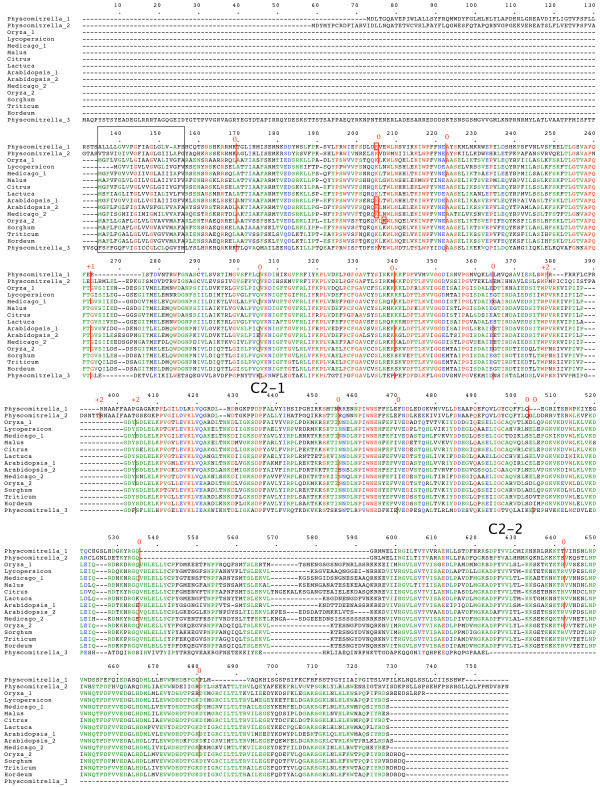
**plant *NTMC2Type2 *genes**. Amino acid sequences of full-length gene products are aligned. The N-terminal TM region is boxed. Intron positions and phases are marked. C2 domains are indicated. RNA edited regions are boxed. Full details are in additional file 1.

**Figure 3:**
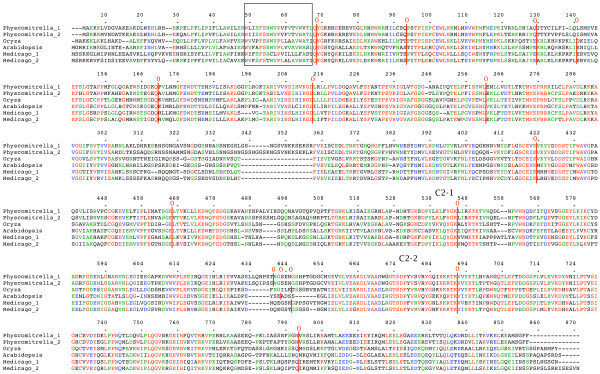
**plant *NTMC2Type3 *genes**. Amino acid sequences of full-length gene products are aligned. The N-terminal TM region is boxed. Intron positions and phases are marked. C2 domains are indicated. Full details are in additional file 1.

**Figure 4:**
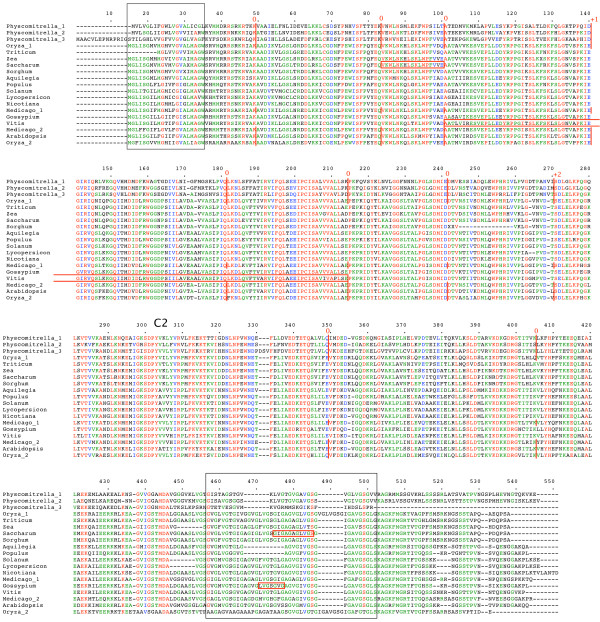
**plant *NTMC2Type4 *genes**. Amino acid sequences of full-length gene products are aligned. TM regions are boxed. Intron positions and phases are marked. C2 domains are indicated. Alternatively spliced regions are boxed. Full details are in additional file 1.

**Figure 5:**
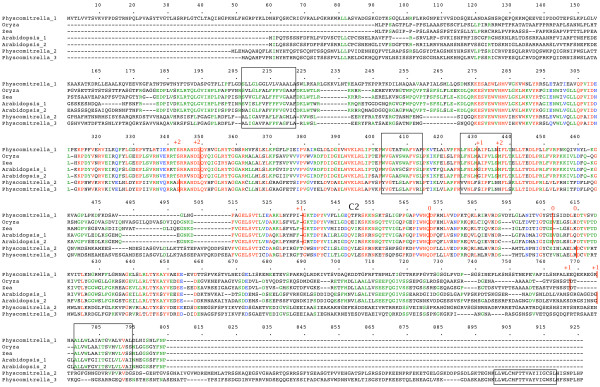
**plant *NTMC2Type5 *genes**. Amino acid sequences of full-length gene products are aligned. TM regions are boxed. Intron positions and phases are marked. C2 domains are indicated. Full details are in additional file 1.

**Figure 6:**
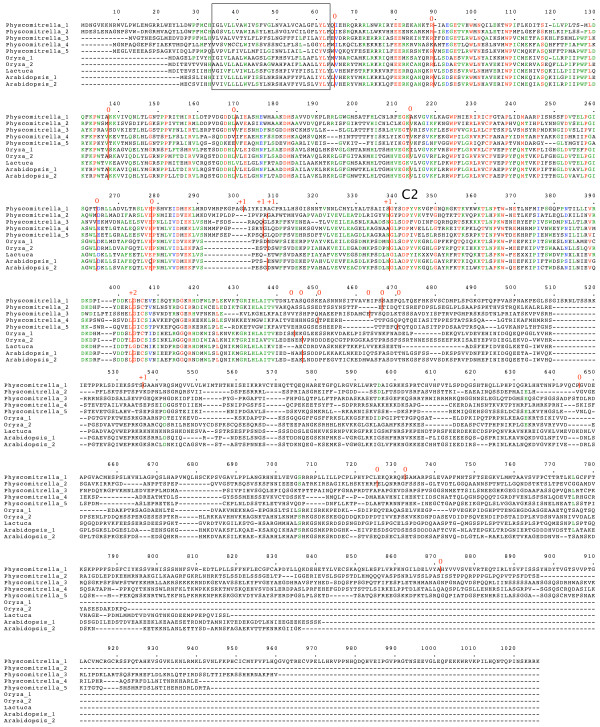
**plant *NTMC2Type6 *genes**. Amino acid sequences of full-length gene products are aligned. The N-terminal TM region is boxed. Intron positions and phases are marked. C2 domains are indicated. Full details are in additional file 1.

Database searching identified six distinct groups of plant genes. Since all of the genes encode relatively long proteins, most of the collection comprises gene fragments which cannot yet be extended to full-length. Only where a large number of overlapping sequences were available was it possible to derive full-length gene sequences from EST contigs. Consequently, the full-length sequences represent the relatively abundantly transcribed, or the shorter genes. Genomic sequences were useful for identifying full-length sequences, irrespective of transcript abundance, as well as for providing the intron-exon structure of the gene. Full-length amino acid sequences were compared using Multalin [24]. The previously used nomenclature (*SytA*, *SytB*, *SytC *etc.) following [14] is somewhat arbitrary and is inadequate for a consistent and meaningful description of these plant genes. I propose the following naming convention for these plant N-terminal-TM-C2 domain genes: *NTMC2Type1.1*, *NTMC2Type1.2*, *NTMC2Type6 *and so on. Multiple alignments of full-length sequences from each group are presented in figures 1, 2, 3, 4, 5, 6

Figures to 1, 2, 3, 4, 5, 6 show the overall domain pattern common to all of the genes: the N-terminal region, TM region, linker, C2 domain region and C-terminal region. Strongly conserved intron patterns, as well as distinctive patterns of sequence conservation, distinguish the six types of *NTMC2 *genes. The six groups are not entirely homogeneous. *Physcomitrella patens NTMC2Type2.3 *for example, while sharing its bulk with the other members of the *NTMC2Type2 *group, has different N and C termini and lacks the second C2 domain. The *NTMC2Type2 *group is also notable in that some of its members are RNA edited (see figure 2 and full details in additional file 1). In some members of this group, the genomic sequence of the second coding exon lacks one nucleotide at its 3' terminus, resulting in a faulty, frameshifted gene. However, these genes are still able to produce functional transcripts with the missing guanosine restored. Transcripts for both *Arabidopsis thaliana NTMC2Type2 *genes, and the *Oryza sativa NTMC2Type2.2 *gene are edited in this way. Transcript sequences have not yet been deposited in the sequence databases for the *Physcomitrella patens NTMC2Type2.1 *and the *Medicago truncatula NTMC2Type2.2 *genes, but I have assumed that they are similarly edited. The genomic loci of the *Physcomitrella patens NTMC2Type2.2 *and *NTMC2Type2.3 *genes and the *Oryza sativa NTMC2Type2.1 *gene, do not lack the equivalent nucleotide, and are not frameshifted. The genomic locus of the *Medicago truncatula NTMC2Type2.1 *gene lacks the equivalent exon-intron boundary altogether and is not frameshifted. The first coding exon of the *Medicago truncatula NTMC2Type2.1 *gene is equivalent to a fusion of the first three coding exons of the *NTMC2Type2 *genes mentioned above, with the corresponding two introns missing. The frameshift error thus appears to be associated with a particular intron. Other examples of divergent members of a group are *Physcomitrella patens NTMC2Type4.3*, which diverges at its C terminus and *Physcomitrella patens NTMC2Type5.2 *and *NTMC2Type5.3*, which have a different intron pattern. Group 6, as a whole, is not well conserved C-terminal of the C2 domain.

### Collection of animal *FAM62 *genes

I had previously identified genes in metazoans and non-metazoans which encode N-terminal-TM-C2 domain proteins sharing similarity with those of plants [4]. In the meantime, with the annotation of the human genome, the three members of this gene family in *Homo sapiens *have been named *FAM62A, FAM62B *and *FAM62C *[25]. I sought to identify homologues of these genes in other organisms by tblastn searching genomic sequences, thereby identifying full-length genes and their intron-exon structures. In contrast to the current status of primary nucleotide sequences from plants, many more animal genomic sequences are available to search. One reason for this is that animal genomes are relatively small in comparison to plant genomes and are therefore relatively less expensive to sequence. After identifying *FAM62 *gene homologues in genomic sequences, I searched transcript sequences using blastn with nucleotide probes, to confirm the predicted gene structures. I identified *FAM62 *homologues in a range of metazoan genomes. Details of each gene are listed in additional file 3.

### Analysis of full-length *FAM62 *genes

**Figure 7:**
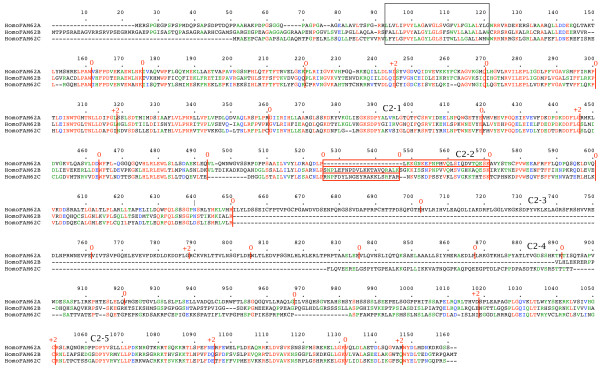
**Three *Homo sapiens FAM62 *genes**. Amino acid sequences of the gene products are aligned. The N-terminal TM region is boxed. Intron positions and phases are marked. C2 domains are indicated. An alternatively spliced region in the second C2 domain is boxed.

**Figure 8:**
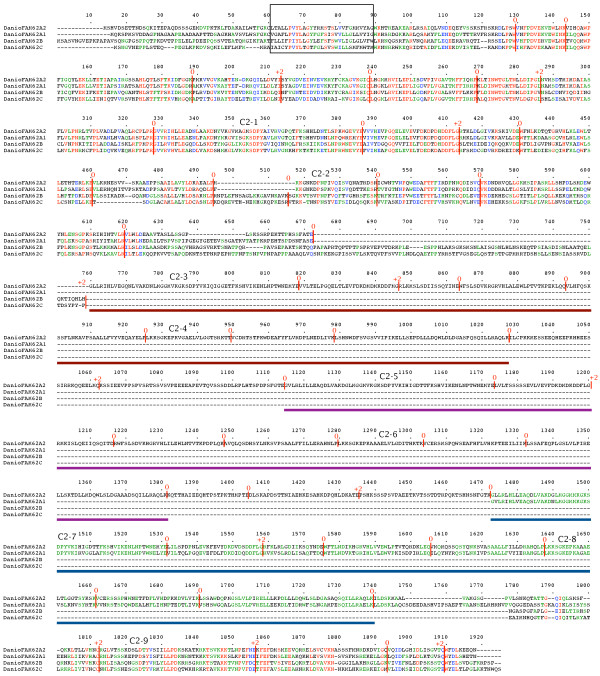
**Four *Danio rerio FAM62 *genes**. Amino acid sequences of the gene products are aligned. The N-terminal TM region is boxed. Intron positions and phases are marked. C2 domains are indicated. Repeated modules are underlined.

Full-length amino acid sequences were compared using Multalin [24]. Figure 7 shows a multiple alignment of the three *FAM62 *gene products from *Homo sapiens*. All three share a common gene structure, but while *FAM62B *and *FAM62C *each encode three C2 domains, *FAM62A *contains a repeat of the portion of the gene encoding the first two C2 domains, resulting in a total of five C2 domains. Figure 8 shows a multiple alignment of four *FAM62 *gene products from *Danio rerio*. The genome of *Danio rerio *encodes at least four *FAM62 *genes. All four share a common gene structure, but while *FAM62B *and *FAM62C *each encode three C2 domains, the *FAM62A *homologues contain additional repeats of the module which encodes the first two C2 domains. This results in a total of five C2 domains in *FAM62A1 *and nine C2 domains in *FAM62A2*.

**Figure 9:**
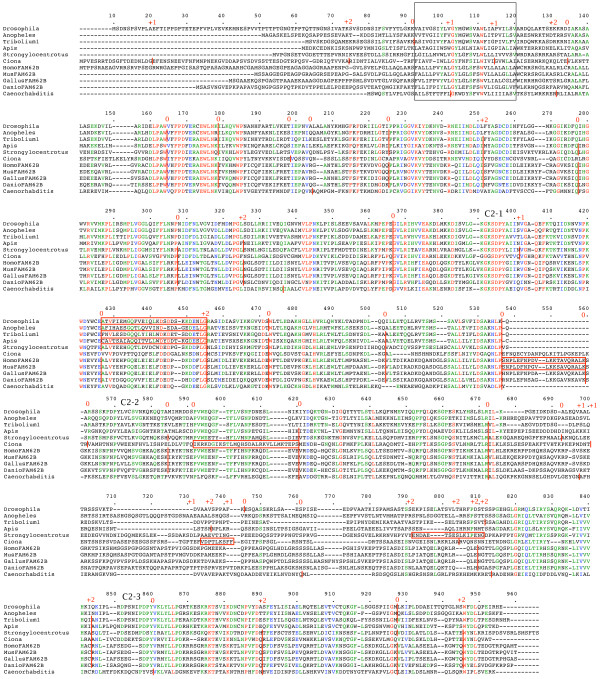
**Metazoan *FAM62 *genes**. Amino acid sequences of the gene products are aligned. The N-terminal TM region is boxed. Intron positions and phases are marked. C2 domains are indicated. Alternatively expressed regions are boxed.

**Figure 10:**
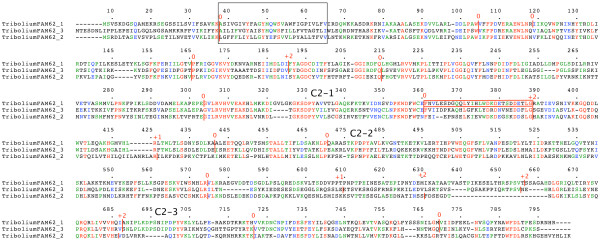
**Three *Tribolium castaneum FAM62 *genes**. Amino acid sequences of the gene products are aligned. The N-terminal TM region is boxed. Intron positions and phases are marked. C2 domains are indicated. Alternatively expressed regions are boxed.

Figure 9 shows a multiple alignment of *FAM62 *gene products from a range of metazoans. The genomes of *Drosophila melanogaster*, *Anopheles gambiae*, *Apis mellifera*, *Strongylocentrotus purpuratus*, *Ciona intestinalis *and *Caenorhabditis elegans *each appear to encode one *FAM62*. The genome of *Tribolium castaneum *has an unusual and compact *FAM62 *locus. It is approximately 12 kilobases long and contains three closely spaced *FAM62 *copies in tandem. Only the first copy retains the intron pattern common to other *FAM62 *genes and is shown in figure 9. The other two copies have diverged from the first and from each other, both in terms of amino acid sequence and intron position (see figure 10 and further details in additional file 3). A duplicated, alternative exon in the region of the first C2 domain of the insect *FAM62 *genes is shown boxed in figures 9 and 10. This is reminiscent of alternative readings of the C2B region of certain *Syt1 *genes [3,26,27]. In the *Syt1 *cases, the insect *Syt1 *genes employ RNA editing to alter this region, while *Caenorhabditis elegans *and *Aplysia californica *encode duplicate alternative exons like the insect *FAM62 *genes here. The duplicated alternative exons are absent from the second and third *FAM62 *copies in *Tribolium castaneum *(figure 10). Vertebrate genomes encode at least three *FAM62 *genes. The *FAM62B *genes of vertebrates appear to be most similar to the single *FAM62 *genes of other organisms and are therefore included in figure 9. Alternatively spliced regions of *Homo sapiens *and *Mus musculus FAM62B *are shown boxed as well as some alternatively spliced regions of *Ciona intestinalis FAM62*.

### Analysis of the structure of Syt genes

Collection and analysis of the plant *NTMC2 *genes and animal *FAM62 *genes revealed intron patterns which are highly conserved within the different groups, implying a long evolutionary history for the whole length of each gene. I have previously looked at the intron patterns of *Syt *genes and found strong conservation of particular intron positions [3,4]. To make clear the differences between the plant and animal N-terminal-TM-C2 domain genes and *Syt *genes which are also N-terminal-TM-C2 domain genes, I analyzed the intron positions within the coding regions of *Syt *genes from a wide a range of metazoans. Details of *Syt *genes shown here but not previously reported [4] are in additional file 4.

**Figure 11:**
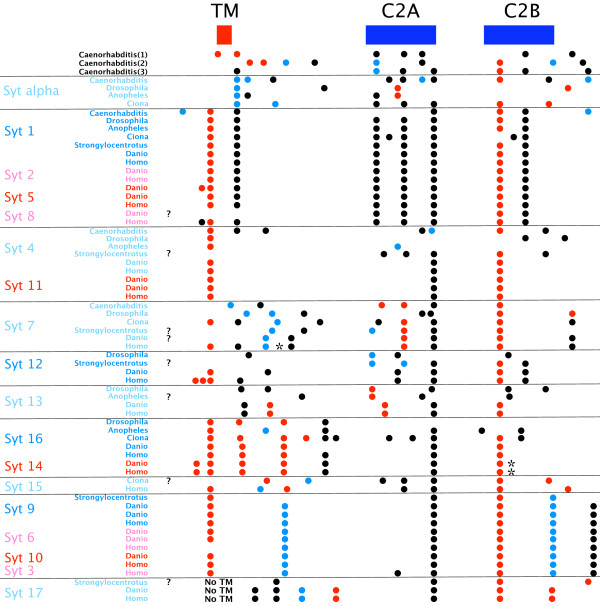
**Intron pattern in *Syt *coding regions**. This figure shows an overview of the structures of *Syt *genes. Intron positions relative to TM, C2A and C2B domains, and their phases are indicated. Phase 0 introns are indicated by black dots. Phase 1 introns are indicated by red dots. Phase 2 introns are indicated by blue dots. Question marks indicate unknown regions where the genomic sequence is incomplete. The positions of additional alternative exons [11,4] are indicated by asterisks. Groups of likely orthologues are indicated in shades of blue. Groups of likely paralogues are indicated in shades of red.

**Figure 12:**
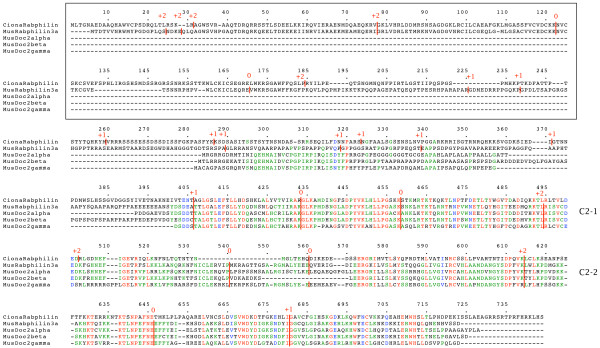
**Rabphilin and Doc2 genes**. Amino acid sequences of the gene products are aligned. Intron positions and phases are marked. The Rabphilin effector domain is boxed and C2 domains are indicated.

Figure 11 shows an overview of the intron patterns in *Syt *genes. Intron positions and their phases are shown relative to TM, C2A and C2B domains. The conserved introns between the C2A and C2B domains stand out clearly. I have included *Syt17 *(also known as B/K [28]) homologues here. Although *Syt17 *homologues lack the N-terminal TM domain and were therefore excluded from my previous analysis [4] their intron structure is indeed characteristic of *Syt *genes and different from other *Syt-*like genes, such as those encoding Doc2 and Rabphilin proteins (figure 12, details in additional file 4). The HUGO gene nomenclature committee [25] have agreed to name the *Homo sapiens *gene locus *SYT17 *so I follow this nomenclature here. The finding of a *Syt9 *homologue in *Strongylocentrotus purpuratus *expands beyond vertebrates a group of *Syt *genes *(Syt3, Syt6, Syt9 *and *Syt10) *previously seen only in vertebrates. I have identified additional *Syt *genes in genomes examined previously. The *Ciona intestinalis Syt*α (following the nomenclature of [9]) is a previously unidentified member of a group present in *Caenorhabditis elegans*, *Drosophila melanogaster*, *Anopheles gambiae *and *Ciona intestinalis *but not present in *Strongylocentrotus purpuratus*, *Danio rerio *or *Homo sapiens*. The *Danio rerio *genome sequence is still being completed and has yielded substantially more information since my last analysis [4].

In figure 11, I have arranged the *Syt *genes into groups of likely orthologues and paralogues. Genes from different species, which are more similar to each other than to other genes from the same species, can be classed as orthologues, and thus defined, are taken to be related by vertical descent from a common ancestor [29]. The functional implications of such a relationship are that orthologues may fulfil similar, perhaps equivalent, roles in different species. As mentioned in the Background section of this paper, this may be broadly true for *Syt1 *genes which appear to be present in all animals. The intron pattern distinctive of *Syt1 *genes, is highly similar to the intron patterns of the *Syt2*, *Syt5 *and *Syt8 *genes. These genes appear only in the evolutionarily more modern vertebrate lineages, so it is likely that they have arisen via *Syt1 *duplication during the evolution of vertebrate lineages and could therefore be classed as paralogues, relative to *Syt1*. The functional implications of such a relationship are that paralogues may fulfil a subset of the roles of the parent orthologue through a process of subfunctionalization, or acquire new roles through a process of neofunctionalization [29]. The *Syt11 *genes appear similarly related to the *Syt4 *group and the *Syt14 *genes similarly related to the *Syt16 *group. The *Syt6*, *Syt10 *and *Syt3 *genes also appear similarly related to the *Syt9 *group. Until a more complete picture emerges from the accurate identification of complete genome complements of *Syt *genes and *Syt*-like genes from many more eukaryotic lineages, it will not be possible to classify these genes more accurately as orthologues and paralogues.

## Discussion

**Figure 13:**
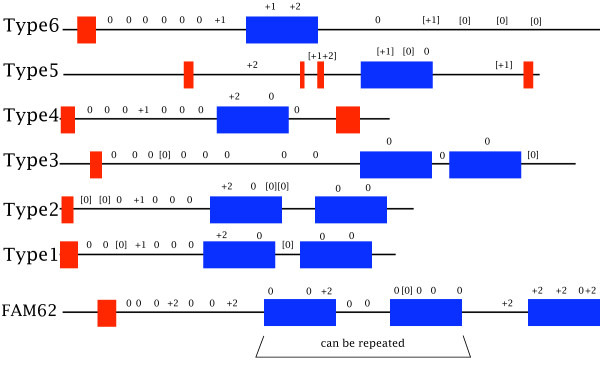
**Structures of 6 groups of plant genes and the similar FAM62 genes of metazoans**. TM regions are represented by red boxes and C2 domains by blue boxes. Intron positions and phases are indicated. Those within square brackets are not always present.

I have examined groups of genes in plants and animals which encode N-terminal TMs followed by a linker and one or more C2 domains. The *NTMC2 *genes and the *FAM62 *genes share sequence similarity in the linker region between the N-terminus and the first C2 domain. This region has recently been identified as a conserved domain of unknown function named SMP [30]. The *NTMC2 *genes have one or two C2 domains and the *FAM62 *genes have three or more C2 domains. The plant genes and the animal genes each have modular gene structures with conserved intron positions. Figure 13 shows a summary of the structures of the *FAM62 *genes and the *NTMC2 *genes.

*FAM62*-like genes are identifiable in yeasts and fungi, but their more divergent sequences and general lack of introns set them apart from the group of metazoan *FAM62 *genes and I have not analysed them here. I have identified similar genes in other non-metazoans, such as *Trypanosoma brucei*, *Ostreococcus tauri *and *Cyanidioschyzon merolae*, but these too are quite divergent and lack introns (details in additional file 5). All of the full-length nucleotide sequences in this paper are listed in additional file 6. All of the full-length amino acid sequences in this paper are listed in additional file 7.

The *NTMC2Type1*, *NTMC2Type2 *and *NTMC2Type3 *genes are *Syt*-like, in that they have an N-terminal TM and two separately conserved C2 domains. Their conserved intron patterns distinguish them from *Syt *genes which have only been found in metazoans and have their own distinctive intron patterns. The *NTMC2Type1*, *NTMC2Type2 *and *NTMC2Type4 *genes are highly similar up to the first C2 domain, indicating a possible gene fusion or fission.

A gene fission event is apparent in the genes encoding Doc2 and Rabphilin proteins (figure 12, details in additional file 4). Rabphilin and Doc2 are related proteins, each with two tandem C-terminal C2 domains which share amino acid sequence similarity with Syt C2 domains. They have partly shared gene structures. The genes encoding the Doc2 proteins comprise the C-terminal half of the genes encoding Rabphilin and thus lack the N-terminal Rabphilin effector domain. Whereas genes encoding Rabphilin are widely distributed among metazoans, genes encoding Doc2 appear to have arisen in the vertebrate lineage. *Ciona intestinalis *has one Rabphilin gene and no Doc2 genes. *Mus musculus *has one Rabphilin gene and three Doc2 genes. Figure 12 illustrates these sequences and their common gene structure. The conserved intron positions help to clarify the relationship between the Doc2 genes and the Rabphilin genes. The intron patterns within the C2 domain regions of these genes appear dissimilar to those of any of the other groups of C2 domains analysed here, further demonstrating that genes which share similarity at the amino acid level, can be divided into genuinely homologous families on the basis of their gene structures.

The difficulty of applying a consistent and meaningful gene nomenclature is highlighted by this work. In the past, gene naming was usually the result of slow and painstaking research. Genes were given names indicating a phenotype or functional aspect of an expressed product. Now in the genome era, vast numbers of genes are appearing at great speed. To make sense of all this new information, evolutionary genomics [29] aims to dissect the complex relationships between genes in different life forms over evolutionary time scales, thereby improving genome annotation. Genes can express multiple functional products and be regulated differently in different contexts. This means that it cannot be straightforward to predict the functional consequences of variations at particular genomic loci, in different species or even different individuals. Functional annotation of genomes is therefore not a straightforward task.

There is already confusion with *Syt *nomenclature (see for example *SYT5, Syt5*, *SYT9 *and *Syt9 *in the Gene and Pubmed databases at NCBI). Equivalent genomic loci in different species can be given different names through separate genome annotation pipelines, and the community of researchers engaged in functional studies of the gene products, continue to supply yet more names relating to the particular functions they have studied (for example, see [15]). In this paper I have named the *Syt*α genes, which lack human homologues, in line with [9]. I have named those with human homologues, according to the HUGO gene nomenclature committee approved human gene names [25]. Three *Syt *genes in *Caenorhabditis elegans *remain unclassified at present and I have simply numbered them (1) to (3) for now. The Wormbase [31] nomenclature for *Caenorhabditis elegans Syt *genes: *snt-1 *to *snt-6 *does not (apart from *snt-1 *being numbered consistent with its relationship to other *Syt1 *genes) yet take account of their evolutionary relationships. Flybase [32]*Syt *gene names are currently restricted to three of the seven *Syt *genes in *Drosophila melanogaster*: *Syt1,4 *and *7 *(yet see [33] where four *Syt *genes were identified in *Drosophila melanogaster*, but only two of these match Flybase *Syt *genes, likely due to inaccuracies in the source databases used). While the *Homo sapiens *and *Mus musculus *genes encoding Rabphilin have now been named *RPH3A *and *Rph3a*, respectively, the genes encoding Doc2 proteins have not yet acquired genome nomenclature committee approved names. I named the *FAM62 *genes in this paper according to the HUGO gene nomenclature committee approved names, but these names have no functional meaning. I suggest a nomenclature for the plant genes which describes their domain composition. This may have some functional relevance.

For the future annotation of genomes with homologues of the genes discussed here, it would be useful to incorporate these gene predictions into the sequence databases such that they are obviously visible and appropriately connected. This should be possible via the recently introduced Third Party Annotation (TPA) facility at the NCBI and EMBL nucleotide sequence databases. Genome annotation needs to be updated continuously and the information from separate genome projects integrated. A possible wiki solution to the problem of updating genome annotation has recently been proposed [34].

## Conclusion

A comparative genomics analysis of genes with N-terminal-TM-C2 domain architectures helps to understand how these genes have evolved. Although it is not possible to draw firm conclusions about the total gene complement of organisms from incomplete genome sequences, such information is needed for sound inferences about the origin and diversification of gene families. The examination of a wide variety of fragmentary sequences does, however, provide much information, useful both for understanding the evolution of genes and their functional products. Large scale, structure-based comparisons of protein sequences inform functional perspectives on the evolution of protein repertoires eg. [[35-37] and references therein]. A structural analysis of eukaryotic C2 domain proteins [38] has considered the evolution of this particular domain. For more gene-oriented perspectives, see eg. [29,39,40] and for a consideration of non-coding sequence evolution, see eg. [41,42].

The collection of genes used here, includes evolutionarily widely dispersed genes with distinctive intron-exon patterns. It includes several gene families with long evolutionary histories. The origins of these gene families are not yet clear but appear to be several. Genome sequences from more lineages of simple, deep-branching eukaryotes may, in future, reveal the earlier histories of these gene families. The collection demonstrates different modes of gene evolution: the C2 domain duplication of *FAM62A *genes, the whole gene duplication of the *Tribolium castaneum FAM62 *genes and *Mus musculus *Doc2 genes, the alternative exons of the C2-1 domain encoded by insect *FAM62 *genes, the gene fusion/fission of *NTMC2Type2*/*NTMC2Type4 *and Rabphilin/Doc2 genes, and the expansion and diversification of the *Syt *gene family. Intron gains and losses are also demonstrated. Intron movements in the duplicated *Tribolium castaneum FAM62 *genes and intron movement with functional consequences in the *NTMC2Type2 *genes are interesting examples. The mechanisms of intron gain and loss and the causes of intron evolution are matters of considerable debate [39,43]. This gene collection provides some useful information for this area of investigation.

Different gene products in this collection share a domain architecture which implies membrane proteins tethered by TM domains, which via their C2 domains, interact with lipids, other membranes and other proteins, sometimes in a calcium regulated manner. Functional studies on many of these genes have yet to be undertaken. It remains to be seen exactly what levels of functional equivalence exist even between different members of the same gene family, for example, the *Syt *gene family. An empirical approach to investigating the functions of plant *NTMC2 *genes and animal *FAM62 *genes would therefore seem more wise than attempting to make functional predictions based on their shared structural domains, which are not homologous. Improved understanding of the evolutionary relationships among these genes will help to guide and interpret future functional studies as well as informing the effort to annotate genome sequences. I hope that innovations in gene and genome annotation will in future allow the easy integration of new results from functional studies and that new functional studies can likewise be informed by evolutionary considerations based on good annotation. Complex, eukaryotic genes are difficult to predict accurately from genome sequences and need to be verified by comparison with transcript sequences. This is especially important when subtle gene regulation by alternative splicing and RNA editing is involved. Ideally, in time, it will be possible to integrate all sources of data into a comprehensible resource.

## Methods

### Cloning and sequencing of *Physcomitrella patens *genes

*Physcomitrella patens *genomic DNA was a gift from Didier Schaefer. I used this as a template for PCR reactions. I amplified genomic regions using Pfu turbo polymerase with phosphorylated primers and cloned the products into Sma digested pBSIIKS-. After sequencing, overlapping clones were selected and digested with restriction enzymes in such a way as to ligate the genomic locus into one piece. The sequence of each genomic clone was deposited in the public sequence databases [EMBL:AM410046, EMBL:AM4100449, EMBL:AM410050]. cDNA clones, also gifts from Didier Schaefer, were obtained from the M. Hasebe collection [44] at PHYSCObase [23] and sequenced completely. These sequences were deposited in the public sequence databases [EMBL:AM410045, EMBL:AM410047, EMBL:AM410048].

### Confirmation of RNA editing of *Arabidopsis thaliana NTMC2Type2.2*

A full-length cDNA clone of *Arabidopsis thaliana NTMC2Type2.2 *was a gift from Boris Voigt. I confirmed the coding sequence and deposited this in the public sequence databases [EMBL:AM410051].

## Supplementary Material

Additional file 1 Plant *NTMC2 *genes. Click here for file

Additional file 2 Plant species. Click here for file

Additional file 3 *FAM62 *genes. Click here for file

Additional file 4 New *Syt *sequences, Rabphilin and Doc2 sequences. Click here for file

Additional file 5 Other non-metazoan genes. Click here for file

Additional file 6 All full-length nucleotide sequences. Click here for file

Additional file 7 All full-length amino acid sequences. Click here for file
