# Supplementary material for: Evolutionary genomics of plant genes encoding N-terminal-TM-C2 domain proteins and the similar FAM62 genes and synaptotagmin genes of metazoans
Source: BMC Genomics. 2007 Jul 31;8:259. doi: 10.1186/1471-2164-8-259 (PMC1976326; doi:10.1186/1471-2164-8-259)
Supplement: Additional file 1 — Plant NTMC2 genes. [file 1471-2164-8-259-S1.pdf]

Additional file 1 - Plant *NTMC2* genes

Plant gene predictions and transcript evidence.
Organisms are arranged in rough phylogenetic order.
Accession numbers of transcripts and additional information is listed.

translation start/stop codons are underlined
^ indicates an intron
complementary strand nucleotide positions indicated within parentheses

Physcomitrella patens Full Length ***NTMC2Type1.1***

Gene 101 Craxton, M. BMC Genomics. 2004 Jul 6;5(1):43

Sequence predicted from genome http://www.ncbi.nlm.nih.gov/Traces
MGVVNTVLGLVGFGWGLGFGLVTGYFLFIYLQPTEVK**^ 0**
DPFIAQLGEMDEKSLDDLLDDIPLWVKNPDYER**^ 0**
VDWLNRFLKDMWPYLDK**^ 0**
AICKMIREQAQPHIDLYGPKFKLDAIEFESLTLGSLPPTFV**^ +1**
GMKVYETKEKEMILEPSFKFAGNPNIIVAVRAFGMKATVQ**^ 0**
LVDVQVSATARVTLKPLIPVFPCFSKIIVSLMSK**^ 0**
PQVDFGLKLLGGDIMAIPGLYRYVQ**^ 0**
DNISSQVAKMYMWPKKMDIPVNDDP**^ +2**
SASKKPVGIIEVTIVKATNLVRKDTFGKSDPYVKVQLVNTIHSKKTTHKLSTLNPVWNEV
VKLTIQDPKTQSLELQVFDWDK**^ 0**
VGSHEKMGMVIVPLSELVENVPKLYNGLKLLKNVDPNDEKNLKSRGEITFEILFKPFKD**^ 0**
DDDSDDQGEAAADGSQMTPEGTGGGLLTVTIVQAEGLEGKHHNNPFVELHFKGDKRKTH**^ 0**
VVKKNREPRWDAEFTWNLEEAPENEHLLLEVHSRGSSMNMVHRQ**^ 0**
ESLGHADISLRDLRGMSKRINETYTLVDGHGKIQVVLDWQAV

ATGGGGGTCGTGAACACGGTCCTCGGCTTGGTGGGGTTCGGATGGGGACTCGGATTTGGT
CTCGTCACTGGATACTTCCTCTTCATCTACCTGCAGCCCACCGAAGTGAAGGATCCATTT
ATCGCGCAGCTGGGGGAGATGGATGAAAAGTCCCTAGATGACCTTCTTGATGATATACCT
TTGTGGGTGAAGAACCCAGACTACGAGCGGGTGGATTGGTTGAACAGATTCTTGAAAGAT
ATGTGGCCTTATCTCGACAAGGCCATTTGCAAGATGATTCGAGAGCAGGCCCAACCACAT
ATCGATCTGTATGGTCCAAAGTTTAAGCTGGACGCTATCGAGTTTGAGTCACTCACTTTG
GGATCACTTCCTCCTACTTTTGTTGGTATGAAAGTATACGAAACTAAGGAGAAGGAGATG
ATTCTTGAGCCCTCGTTCAAGTTTGCTGGAAATCCGAACATAATTGTCGCTGTGAGGGCT
TTTGGTATGAAAGCAACAGTACAGCTAGTAGATGTTCAAGTTTCTGCCACCGCGCGAGTT
ACTCTGAAGCCGCTGATTCCCGTTTTCCCCTGCTTCTCGAAGATAATTGTTTCTTTGATG
AGCAAGCCTCAGGTAGATTTTGGTTTGAAGTTGCTGGGTGGAGACATCATGGCAATTCCT
GGATTGTACCGTTATGTTCAGGACAATATTAGTAGTCAAGTTGCCAAAATGTATATGTGG
CCCAAGAAAATGGATATTCCTGTTAATGATGATCCCAGTGCTTCAAAGAAACCCGTAGGC
ATAATTGAGGTAACGATTGTTAAAGCTACGAACCTGGTGAGGAAAGACACTTTTGGGAAA
TCTGACCCTTACGTGAAAGTCCAATTGGTCAACACTATACATTCCAAGAAGACCACTCAT
AAGCTGAGCACATTGAACCCCGTATGGAATGAGGTTGTCAAGCTGACGATCCAAGATCCC
AAGACGCAGTCTCTTGAATTACAGGTTTTTGACTGGGATAAGGTTGGATCGCATGAGAAG
ATGGGTATGGTAATAGTGCCATTGAGCGAATTGGTGGAGAATGTACCGAAATTATATAAT
GGTCTCAAACTGTTGAAGAACGTGGACCCTAACGACGAGAAGAACCTGAAATCCCGTGGC
GAGATTACCTTCGAAATTTTATTCAAGCCTTTCAAAGATGATGATGATTCAGATGATCAA
GGGGAAGCAGCAGCGGATGGTTCACAAATGACACCCGAGGGTACCGGAGGAGGCTTGCTT
ACAGTCACCATAGTGCAGGCTGAGGGTTTAGAAGGCAAACATCATAATAATCCGTTTGTC
GAATTGCATTTCAAAGGAGATAAGAGGAAAACCCATGTTGTGAAGAAGAACAGGGAACCA
AGATGGGATGCTGAATTCACTTGGAATTTGGAGGAGGCACCTGAAAATGAGCATTTGTTA
CTTGAGGTTCACAGCAGAGGCTCAAGCATGAACATGGTTCATCGTCAGGAATCTTTGGGT
CATGCGGATATTTCTCTCCGGGACTTGAGGGGCATGAGCAAGAGAATCAACGAAACATAT
ACTTTGGTCGATGGTCATGGAAAGATCCAAGTGGTGTTGGATTGGCAGGCCGTTTAG

Transcripts

bj962935
bj963639
bj192791
bj190370
bj206609
bj196386
bj194585
bj965585

bj186779
bj180504
bj158769
bj190651
bj202860
bj195062
bj195596
bj597798
bj974104
bj598213
bj585764
bj595777
bj609430
bj599408
bj587600
bj595702
bj596625
bj607095
bj171961
bj971467
aw145652
bj972176
am410045 full length cDNA sequence from Hasebe clone 38d15

am410046 genomic sequence

Mitsuyasu Hasebe cDNA clones at PHYSCObase http://moss.nibb.ac.jp
21d08
21k18
114h13
6l08
22l14
10h08
28a23
47d08
123d16
26m14
38d15
17j02
10k02
33n20
13a06
30l11
10h08
48p06
24p05
4d11
6d11

Physcomitrella patens Full Length ***NTMC2Type1.2***

Gene 100 Craxton, M. BMC Genomics. 2004 Jul 6;5(1):43

Sequence predicted from genome http://www.ncbi.nlm.nih.gov/Traces
MGIVSTILGFVGFGWGIGVGLAIGYFLFIYFQPVDEK**^ 0**
DPVIRNLDELDSRTLQGLLGEIPLWVKNPDYHR**^ 0**
VDWVNRFLKDIWPYLDK**^ 0**
AICKMVRQQAQPYIDKYGPQYKMDSIEFQSLTLGTLPPTFV**^ +1**
GMKVYDTKEAEMILEPSFKFAGNPNIIVAVKAFGLKATVQ**^ 0**
VVDVQVFATARITLKPLIPVFPCFSKIVVSLMEK**^ 0**
PHVDFGLKLLGGDIMAIPGLYGFVK**^ 0**
DTIANQVADMYMWPKSLEIPINTDA**^ +2**
SADKKPVGVVEVKIIRATNLMKKDFMGKADPYVKIQLVNTMLSKTTRAKMNTLNPEWNQT
FKLSVQDLKSQSLELQVFDWEK**^ 0**
VGAHDKMGMQVVPLKDLQENVPKLQTVPLFKNMDPNDEANSKKRGELTFEMNLRLFKE**^ 0**
DDTEEDIKAKSMDDGQFANGVKSSEGGLLSVIIHQAQELEGKHHTNPFVEVNFRGDKKKTP**^ 0**
VVKKNKNPRWDQLFTWQLDDPPVSDSLHIEVLSKGSSLNMVHRH**^ 0**
EILGSVNIPLGDVVKNKNINSKYGLANSHGMIQVELKWKPV

<u>ATG</u>GGGATTGTTAGCACGATCCTGGGGTTTGTGGGGTTTGGATGGGGCATCGGCGTCGGA
CTTGCGATAGGATACTTCCTATTCATCTACTTCCAGCCTGTTGATGAGAAGGATCCTGTC
ATTCGGAACCTGGATGAGTTGGATTCGAGAACCTTGCAAGGGCTCCTCGGCGAAATTCCT
CTATGGGTGAAGAACCCAGATTACCATCGGGTGGATTGGGTGAACAGGTTCTTAAAGGAC
ATCTGGCCTTATCTTGATAAGGCAATTTGCAAGATGGTTCGACAGCAGGCACAGCCTTAC

ATTGATAAATATGGGCCACAGTACAAGATGGATTCTATCGAGTTCCAGTCACTTACCTTG
GGAACGTTGCCTCCTACATTTGTTGGTATGAAAGTTTATGATACTAAGGAGGCAGAGATG
ATTCTCGAGCCTTCCTTTAAGTTCGCTGGGAATCCAAATATTATTGTTGCTGTGAAAGCT
TTTGGTTTGAAGGCAACAGTACAGGTGGTAGACGTGCAGGTTTTTGCGACAGCAAGAATC
ACACTGAAGCCTTTGATTCCAGTTTTTCCATGTTTCTCGAAAATAGTAGTCTCTTTGATG
GAAAAGCCTCACGTAGATTTTGGTCTGAAGCTGTTGGGCGGAGATATTATGGCAATTCCT
GGACTTTACGGTTTCGTTAAAGATACCATCGCAAATCAAGTCGCTGATATGTACATGTGG
CCGAAGTCTTTAGAAATCCCAATAAATACTGATGCCAGTGCTGATAAGAAGCCAGTGGGC
GTGGTTGAGGTGAAGATTATTCGAGCCACGAACTTAATGAAGAAAGATTTCATGGGTAAA
GCCGACCCTTACGTCAAAATTCAATTGGTTAATACGATGCTTTCGAAGACAACACGCGCC
AAGATGAACACTTTGAACCCCGAATGGAATCAGACTTTCAAGCTTTCTGTTCAAGACCTC
AAGTCACAGTCTCTTGAGCTACAGGTTTTTGACTGGGAGAAGGTTGGAGCACACGATAAA
ATGGGAATGCAGGTCGTGCCATTAAAGGACTTGCAGGAAAACGTTCCAAAATTACAAACT
GTTCCGCTGTTTAAGAATATGGATCCTAATGACGAAGCCAATTCGAAAAAGCGTGGCGAG
TTAACGTTTGAAATGAATTTGAGACTCTTCAAGGAAGACGATACTGAGGAAGATATCAAA
GCGAAATCAATGGATGATGGACAATTCGCAAATGGGGTTAAATCTTCTGAAGGAGGATTG
CTGTCGGTCATCATTCATCAGGCCCAGGAATTAGAAGGCAAACATCACACAAACCCCTTC
GTGGAGGTTAATTTTCGGGGGGATAAAAAGAAAACTCCGGTTGTGAAGAAAAATAAAAAC
CCCAGATGGGATCAACTATTTACTTGGCAACTGGACGATCCCCCCGTTAGTGACTCCCTG
CACATCGAGGTCCTGAGCAAGGGCTCAAGTCTGAACATGGTGCATCGTCATGAAATTTTG
GGATCTGTGAACATTCCTCTTGGAGACGTGGTAAAGAATAAGAACATCAATTCTAAGTAT
GGGCTGGCTAACTCTCATGGTATGATCCAAGTGGAGCTGAAGTGGAAGCCTGTGTAG

Transcripts
bj158693
bj166699
bj602006
am410047 full length cDNA sequence from Hasebe clone 6e22
am410048 full length cDNA sequence from Hasebe clone 12m11

am410049 genomic sequence

Mitsuyasu Hasebe cDNA clones at PHYSCObase http://moss.nibb.ac.jp
62i06
23m18
72e04
108l04
12m11
6e22
132m20
68p19

Physcomitrella patens Full Length ***NTMC2Type1.3***

Sequence predicted from genome http://www.ncbi.nlm.nih.gov/Traces
MGIVGTILALVGFGWGMSIGLGIGYFLFIYMQPAEVQ**^ 0**
DPIIRQLGELDARSLEELLNEIPLWVKNPDYDR**^ 0**
VDWLNKFLKDIWPCLEK**^ 0**
AICKKLRKKAQPYIDKYGSKYMMNSIDFESLTLGTLPPTFV**^ +1**
GMKVYDTKEREIIFEPSFKFAGNPNIIIAVKAFGLKATVQ**^ 0**
LVDVQAFATARITLKHLVPMFPCFSKVVISLMDK**^ 0**
PHIDFGLKLLGGDVMAIPGLYGFVQ**^ 0**
DTIRDRVAEMYMWPKTLEIPIIDDH**^ +2**
SAAKRPVGTVEVKIIRARNLLKTDFMGKADPYVKIRLVNSVLSKTTRTKANTLNPEWHEIFK
LPVQDPKSQSLELEVFDWEK**^ 0**
LGAHEKMGMQIVPLKDLVDDEPKSFTLPLVKNVDPNDEANSKKSRGDIVFEMTFKAFKE**^ 0**
DDNEADIAEESHSASESVPHHGGVLSVTVHQAEEVEGKHHTNPFVELHFRGDKKKTL**^ 0**
VIKKSTDPSWEQEFSWQLDDSPISDSLHVEVLSKRSSMNLFHRQ**^ 0**
ESLGYVDIPLQDVVNNKTINEKFQLVDSPGMIQLELTWRIS

ATGGGGATCGTTGGTACGATTCTTGCATTAGTGGGGTTTGGATGGGGCATGAGCATTGGA
CTAGGGATCGGATACTTCCTCTTCATCTACATGCAGCCTGCTGAAGTGCAAGATCCTATC
ATTCGTCAACTGGGAGAGTTGGATGCACGGTCCTTGGAAGAGCTGCTTAACGAAATTCCT
CTATGGGTGAAGAATCCAGACTACGATCGGGTCGATTGGTTAAACAAGTTCTTGAAGGAC
ATCTGGCCCTGTCTTGAAAAGGCGATTTGTAAGAAGCTTCGAAAGAAAGCACAGCCTTAC
ATTGATAAATATGGTTCGAAGTATATGATGAACTCTATCGACTTCGAGTCGCTTACCTTG

GGGACACTTCCTCCTACTTTTGTTGGTATGAAAGTTTACGATACCAAGGAAAGGGAGATA
ATTTTCGAGCCTTCCTTCAAGTTCGCTGGAAATCCAAATATAATTATTGCTGTGAAAGCT
TTTGGCTTGAAAGCAACAGTGCAGCTTGTAGATGTGCAGGCTTTTGCAACAGCGAGAATC
ACACTAAAGCATTTGGTTCCCATGTTCCCTTGTTTCTCAAAAGTAGTCATCTCTTTAATG
GATAAGCCTCACATAGATTTTGGTCTGAAGCTGTTGGGTGGGGATGTCATGGCAATTCCT
GGTCTTTACGGTTTCGTTCAGGATACTATCAGAGATCGTGTTGCTGAAATGTACATGTGG
CCAAAGACATTAGAAATTCCTATAATTGATGATCACAGTGCTGCGAAGAGGCCAGTGGGA
ACAGTTGAGGTGAAAATTATTCGAGCTAGGAACCTGTTGAAGACGGACTTCATGGGCAAA
GCTGACCCATACGTTAAAATCCGCTTGGTTAACAGTGTGCTTTCGAAGACAACACGCACA
AAGGCGAATACATTGAACCCTGAATGGCATGAGATTTTCAAGCTTCCGGTTCAGGACCCC
AAATCACAATCTCTTGAGCTGGAAGTTTTTGACTGGGAGAAGTTAGGAGCACACGAGAAA
ATGGGAATGCAGATCGTGCCATTAAAGGATTTGGTGGACGACGAGCCGAAATCGTTTACT
TTGCCTTTGGTTAAGAACGTGGACCCTAACGACGAAGCTAACTCTAAGAAATCCCGAGGC
GACATTGTTTTTGAAATGACTTTCAAGGCTTTCAAGGAAGATGACAATGAGGCAGATATT
GCAGAGGAATCACACAGCGCGTCAGAGAGCGTACCTCATCATGGAGGTGTCCTGTCGGTC
ACAGTCCATCAGGCTGAGGAAGTGGAAGGCAAGCATCACACCAATCCCTTCGTGGAGTTG
CATTTTCGTGGGGATAAAAAGAAAACTTTGGTGATCAAGAAGAGCACAGATCCAAGTTGG
GAGCAAGAATTTTCTTGGCAACTGGACGACTCCCCAATTAGTGATAGCCTGCACGTTGAG
GTTCTTAGCAAGCGCTCAAGCATGAATCTATTTCATCGTCAGGAGTCTTTGGGGTATGTA
GACATTCCTCTTCAGGACGTAGTGAATAACAAGACCATCAACGAAAAGTTTCAGCTGGTG
GACTCTCCTGGAATGATTCAACTGGAGCTAACCTGGAGGATTTCGTAG

Physcomitrella patens Full Length ***NTMC2Type2.1***

Sequence predicted from genome http://www.ncbi.nlm.nih.gov/Traces
MDLTGQAVEPIWLALLSYFRQMWDYFGLMLHLYLAPDERLGREAVDIFLIGTVPSFLLRS
TSALLLLGVVPGFIAGLGLVAFSHCQTSRSHKRRMK**^ 0**
TGLIHMISEMNKDDYWSLFPKSVLPRWIEFSDLDK**^ 0**
it is unclear how this splice can occur
VEWLNSVIKKIWPFFNE**^ 0**
AYSKMLMKRWEPYLDSHKPSFVNLVSFHELTLGSVAPQFE**^ +1**
EISTDVNTRWFGNASCTLSVSTIMGVSFPLQ**^ 0**
VKDIHIKGVFRFIYKPLVDELPGFGAVTYSIRKK**^ 0**
KKFDFVVMVVGGDISNVPGMVQKLH**^ 0**
LMVQSAVIESLSWP**^ +2**
RFRRFLCPRRNAAFFAAPGAGAKPPLGILDLRLVQGRDLRDRGKPPDPFALVYIHSIPGHIR
KSMTN**^ 0**
RRENNPIWNEFFELEFDDLEDGKVMVVLLDEAAPQEFQVLGYCQFFLQ**^ 0**
EGRITERWPKIYEGTQCHGSLHDGKYRGQ**^ 0**
GRMWELIRGILTVTVVRAENLLSTDFHRKSDPYVVLCMIKHKRLRKKTT**^ 0**
VIHSNLNPVWDESFEFQIEDASQDMLLLHVWNHDSFGK**^ 0**
PLMVAQKHISGSPSIFKCFHFSSTYGYIIAIPGITSLVFILKLNQLSSLLCIISSHWF

ATGGATCTTACGGGTCAAGCAGTAGAGCCAATATGGCTTGCTTTGTTATCATACTTTCGA
CAAATGTGGGATTATTTCGGATTGATGCTGCACCTGTATCTTGCACCCGACGAGCGATTA
GGACGCGAAGCAGTCGACATCTTCCTTATTGGGACGGTTCCTTCTTTCTTACTAAGGAGT
ACATCAGCACTATTACTGTTAGGTGTTGTTCCTGGGTTCATTGCAGGCCTAGGGTTGGTG
GCATTCAGCCATTGCCAAACCAGCCGGTCACACAAGCGTCGAATGAAAACAGGCCTTATT
CACATGATATCTGAAATGAACAAGGACGACTACTGGTCCCTGTTTCCAAAATCAGTACTT
CCTCGATGGATTGAATTCAGTGACCTCGACAAGGTTGAATGGCTGAATTCTGTCATCAAA
AAGATATGGCCCTTCTTCAACGAGGCTTACTCAAAAATGCTAATGAAGCGCTGGGAGCCC
TATCTTGATTCACACAAGCCTAGTTTCGTCAATTTAGTCTCTTTCCATGAGCTTACACTT
GGATCAGTTGCACCTCAATTTGAAGAGATTTCAACTGATGTGAACACACGATGGTTTGGT
AACGCAAGTTGCACACTATCGGTGTCAACAATAATGGGCGTATCTTTCCCTCTCCAGGTA
AAAGATATACACATAAAGGGTGTATTTCGTTTTATTTATAAGCCGTTGGTGGACGAGCTA
CCTGGATTTGGAGCTGTCACTTACTCAATCAGGAAAAAGAAAAAATTTGATTTTGTTGTT
ATGGTTGTTGGAGGAGATATCTCAAATGTTCCCGGTATGGTGCAAAAGCTCCATCTCATG
GTTCAATCTGCGGTGATAGAATCCTTAAGCTGGCCTAGATTCCGGCGCTTTTTGTGCCCA
CGAAGAAATGCAGCGTTTTTTGCAGCACCAGGTGCGGGCGCAAAACCACCTCTTGGAATC
CTTGACCTAAGATTGGTGCAAGGAAGGGACCTTAGGGACAGAGGAAAGCCCCCTGACCCC
TTTGCTTTGGTATACATCCATTCTATTCCAGGGCATATAAGGAAGAGCATGACTAATAGA
CGCGAGAATAATCCGATATGGAATGAATTCTTTGAGCTGGAGTTTGATGACTTGGAAGAT
GGAAAAGTTATGGTTGTTTTGCTCGATGAAGCGGCCCCTCAAGAATTTCAGGTGTTGGGT
TACTGTCAATTTTTCTTGCAGGAGGGACGAATCACAGAACGATGGCCAAAAAATTTACGAG
GGTACGCAATGTCACGGTAGCTTGCATGACGGCAAGTACAGGGGACAGGGAAGAATGTGG

GAGCTGATTCGTGGGATATTGACGGTCACAGTGGTACGTGCTGAGAACCTTCTTTCCACC
GATTTCCACCGCAAGAGTGACCCGTATGTGGTTCTCTGCATGATCAAACATAAGCGATTG
AGAAAGAAAACCACGGTTATACACAGCAACTTGAATCCAGTATGGGACGAAAGCTTTGAA
TTCCAGATCGAAGATGCCAGCCAAGATATGCTTCTTCTGCATGTTTGGAACCACGATTCG
TTCGGCAAGCCTCTCATGGTTGCGCAAAAGCATATTTCGGGCAGCCCATCCATTTTTAAA
TGCTTTCATTTCTCATCCACATATGGGTATATTATCGCAATTCCTGGCATAACTTCGTTG
GTTTTTATCCTGAAATTAAATCAATTATCGTCGCTGCTATGCATCATTTCTTCACATTGG
TTTTAA

Physcomitrella patens Full Length ***NTMC2Type2.2***

Sequence predicted from genome http://www.ncbi.nlm.nih.gov/Traces
MDYMYPCRDFIARVIDLLNQATETVCVSLPAYFLQGWESFQTAPQRNVGPGEKVEREATS
LFLVETVPSFVAGTASVTSVIGIVVGFIAGLGIILAFGYAQRKRSSERRMK**^ 0**
AGLIHLISEMKRSDYRSVFPKSHLPRWIVNRELEK**^ 0**
VDWMNIILRKLWPFFNE**^ 0**
AYSKILLKDWEHRLETFKPAFANSVRVQELTLGSVAPNLE**^ +1**
GLRMLELEPKGISMDVDTRWIGNASCVLEVSSIVGVSFPVQ**^ 0**
VKDIHSKMVFRFIFKPLVDELPGFGAFTLSIRKM**^ 0**
KKFDFTLKVVGGDVSSIPGVTEKLN**^ 0**
EMIHNAVLESLSWPMRICIQIISTPADSNTT**^ +2**
RNAAIFAATGSEGKPPVGILDVKLVQGRDLWDTGKPPDPFALLYIHATPGQIRRSTTI**^ 0**
RQNNNPIWNEFFELEFNDLKEEKIVIVLFDNAAPQEFQVLGYCQFYLQ**^ 0**
GLDDRRVTERWLKVFKDARCLGNLDETKYRGQ**^ 0**
GNLWELIRGILTITVARAENLLPADFQGRSHPYVALHMMKQKRLKKKTS**^ 0**
VKQSTLNPIWHETFDFHVQDARQDMLIAEVWNDEIIGR**^ 0**
DYLGSTATTLTRVLQEYAFEEKFRLVGVPSGRLYLRLSWENSIGTSVISDKPSLLSPSEHFP
SSHGLLQLFPMDVSPN

ATGGATTACATGTACCCCTGCCGAGATTTCATTGCACGGGTGATCGATCTTCTGAACCAA
GCCACAGAAACAGTCTGTGTTTCTTTGCCAGCATACTTCCTGCAAGGGTGGGAGAGTTTC
CAAACCGCGCCTCAAAGGAATGTGGGGCCCGGCGAGAAGGTTGAACGTGAAGCAACTTCA
TTGTTCCTTGTTGAAACGGTTCCGAGTTTCGTAGCTGGGACTGCATCCGTGACATCAGTA
ATTGGTATTGTTGTTGGGTTTATTGCTGGCTTGGGAATAATTCTGGCATTCGGCTATGCC
CAAAGGAAACGGTCAAGTGAACGTCGAATGAAGGCAGGCCTTATTCATTTGATTTCCGAA
ATGAAGAGGAGTGACTACCGGTCTGTGTTCCCCAAATCCCATCTTCCTCGATGGATTGTT
AACCGTGAGCTTGAGAAGGTTGATTGGATGAATATTATTTTGAGAAAATTGTGGCCCTTC
TTCAATGAGGCCTACTCAAAGATTTTACTGAAGGATTGGGAGCATCGTCTTGAGACGTTT
AAGCCTGCCTTTGCCAATTCAGTCAGAGTCCAGGAGCTTACGCTTGGCTCCGTTGCCCCC
AACTTGGAAGGACTTCGAATGCTGGAATTGGAGCCGAAAGGGATTTCAATGGATGTGGAC
ACGCGGTGGATTGGGAACGCTAGCTGCGTATTGGAAGTGTCATCGATAGTGGGCGTATCT
TTTCCTGTACAGGTTAAAGACATACATTCAAAGATGGTATTTCGGTTCATTTTCAAGCCT
TTGGTAGACGAGCTGCCTGGATTTGGAGCTTTTACCTTATCAATTAGGAAAATGAAAAAA
TTCGACTTCACCCTGAAAGTTGTCGGAGGAGACGTTTCGAGCATTCCTGGTGTGACGGAG
AAGCTGAACGAAATGATACACAACGCGGTATTAGAGTCCTTAAGCTGGCCTATGCGGATC
TGCATACAAATAATTTCAACACCTGCAGATAGCAACACGACTAGAAACGCGGCCATATTT
GCAGCAACGGGTTCGGAAGGAAAACCACCAGTCGGTATCTTGGACGTAAAATTGGTGCAG
GGAAGGGACTTATGGGATACAGGAAAGCCCCCCGACCCCTTTGCACTGTTATACATTCAT
GCTACTCCGGGGCAAATAAGGAGGAGCACGACCATAAGGCAAAACAATAATCCAATATGG
AATGAGTTTTTTGAACTAGAGTTTAATGACCTGAAAGAAGAAAAAATTGTGATTGTTTTG
TTCGACAATGCGGCTCCTCAAGAATTTCAGGTGTTGGGCTACTGCCAATTTTACTTGCAG
GGACTTGACGACAGGCGGGTCACAGAGCGATGGCTTAAAGTTTTTAAGGATGCGCGTTGT
CTTGGAAATTTGGATGAAACCAAGTATAGGGGACAGGGAAACTTGTGGGAATTGATTCGT
GGGATATTGACGATCACAGTGGCGCGCGCTGAAAACCTTCTTCCTGCCGATTTCCAGGGC
AGGAGTCATCCGTACGTGGCCCTTCACATGATGAAGCAAAAAAGATTGAAGAAAAAGACC
TCGGTTAAGCAGAGCACCTTGAATCCAATCTGGCATGAAACATTTGATTTTCATGTTCAA
GACGCCCGCCAAGATATGCTCATCGCAGAAGTTTGGAACGACGAAATTATTGGCAGGGAT
TATCTAGGGTCGACAGCCACAACGTTGACCCGAGTGTTGCAGGAATATGCCTTTGAAGAA
AAGTTCAGACTTGTTGGGGTACCCAGTGGAAGATTGTATCTCCGACTCTCATGGGAAAAT
AGTATAGGGACGTCTGTCATTTCAGATAAACCTTCACTTCTTTCTCCTAGTGAGCATTTC
CCATCTTCACATGGCCTCCTACAACTGTTCCCGATGGATGTTTCCCCGAACTGA

Physcomitrella patens Full Length ***NTMC2Type2.3***

Sequence predicted from genome http://www.ncbi.nlm.nih.gov/Traces
MAQPYSTSYEADEGLRRRTAGQGEIDYGTTPVVGRVAGRYEGYDTAPIRRQYDESKSTTS
TSAPPAEQYRKNPNTENERLADESARREDDDSKTSNSGSMGVVGMLKNPRNRMYLATLVA
ATPFMISFTFYVSQFSFFGQFVIGICCGLCLQGVYMLYSLHRKHQRKRK**^ 0**
TLQVAQMAILEENQLKILLPTQEAFPRWISFTDFEK**^ 0**
VEWLNDTLTKLWPYIDQ**^ 0**
AASSLIKEKVQPILDQYAMGIIQKLELKQVAFGNKAPQVT**^ +1**
GLEDETVLEIKILWETSQEGVVLSVDFPGPNYTVK**^ 0**
LKNWFLEGTAKLIFKPLTGTIPGFGAVLVSLTEP**^ 0**
PEFDFDLKFLGGDVGMVPGVEKMID**^ 0**
NSIRTALMDSLVWPSRIVVPMIPGGDF**^ +2**
SFLELHPVGELEVKLIEAKNIKNTDLIGKADPFVTLFVRQTKDKVKRSTSKSNTLRPVW
NEDFKIE**^ 0**
VEDPESQALTLRLMDDESVQKSEYIGTVQLAIKE**^ 0**
FEPHVKKELWCDVLEDPESHATDQIRGSIHVIVTYIPYTREQVEAKRGFNETEKKIYEEH
RLIAEQINRPHKTTGDAPKQQGNITQHPEEQPRQPGPAAEL

ATGGCGCAACCATATAGCACATCCTATGAAGCCGACGAAGGCCTAAGGCGCAGAACTGCG
GGACAGGGAGAGATTGACTACGGCACCACTCCCGTGGTCGGCAGAGTAGCCGGACGTTAC
GAGGGGTACGACACAGCTCCTATTAGGAGGCAATACGATGAATCCAAATCTACTACATCA
ACGTCTGCACCACCTGCAGAACAGTACCGGAAGAATCCGAACACGGAAAATGAGCGGTTG
GCAGATGAATCCGCGAGAAGGGAAGATGACGACTCCAAAACTAGCAATTCTGGCAGCATG
GGCGTTGTCGGTATGCTGAAAAATCCTCGTAATCGAATGTATCTCGCTACCTTGGTTGCA
GCCACGCCGTTCATGATCTCTTTCACTTTTTATGTGAGCCAATTCTCCTTCTTCGGACAG
TTCGTGATCGGCATTTGCTGCGGTTTATGTCTACAAGGAGTGTACATGTTGTACAGTTTG
CACCGGAAGCATCAACGCAAGCGCAAGACTCTGCAAGTAGCGCAAATGGCGATTCTGGAG
GAGAACCAGCTCAAAATTCTTTTACCCACGCAGGAGGCTTTCCCCCGATGGATCTCCTTC
ACCGACTTTGAAAAGGTGGAATGGCTGAACGACACTTTGACCAAGTTATGGCCGTATATT
GATCAGGCAGCTTCAAGCCTGATCAAAGAGAAGGTTCAGCCTATTTTGGACCAATATGCT
ATGGGAATCATTCAGAAGCTCGAGCTCAAGCAGGTTGCATTCGGCAACAAAGCTCCGCAA
GTTACAGGTTTGGAAGACGAGACTGTCTTGGAGATCAAGATCCTCTGGGAGACCTCTCAA
GAGGGTGTGGTCCTGTCTGTTGATTTCCCCGGTCCAAACTACACAGTGAAGCTCAAGAAC
TGGTTTCTGGAAGGAACTGCGAAGCTTATATTCAAGCCCTTGACAGGCACCATTCCTGGA
TTTGGAGCCGTCTTAGTGTCACTAACGGAGCCACCAGAGTTCGACTTCGATTTGAAATTC
CTGGGTGGAGATGTTGGAATGGTTCCAGGAGTGGAGAAGATGATTGACAACTCTATCAGG
ACAGCTTTGATGGACTCTCTGGTATGGCCAAGTCGCATTGTGGTTCCTATGATCCCCGGT
GGTGATTTCAGTTTCTTGGAGCTTCATCCAGTTGGGGAACTGGAAGTGAAATTGATCGAA
GCTAAGAACATCAAGAACACCGACCTCATTGGCAAGGCAGATCCATTTGTAACCTTGTTT
GTGCGCCAAACAAAGGACAAAGTGAAGCGCAGTACATCCAAGAGTAATACGCTACGCCCT
GTCTGGAATGAGGACTTCAAGATAGAGGTGGAAGACCCAGAATCTCAGGCCTTGACACTG
CGGCTCATGGACGATGAAAGTGTTCAGAAGTCGGAGTACATTGGAACCGTTCAGTTAGCC
ATCAAGGAGTTCGAGCCACACGTGAAGAAAGAGTTGTGGTGCGACGTCTTGGAGGACCCC
GAGTCTCATGCGACGGACCAGATCCGGGGCAGTATCCACGTTATTGTGACCTACATCCCG
TACACACGAGAGCAGGTTGAAGCGAAGCGCGGGTTCAACGAGACAGAGAAGAAGATCTAC
GAGGAGCACCGGCTCATTGCGGAGCAGATCAATCGCCCTCACAAGACCACCGGCGACGCC
CCGAAGCAGCAAGGTAACATCACCCAGCACCCAGAGGAGCAACCGAGGCAGCCTGGCCCA
GCAGCGGAGCTCTGA

Transcripts
cj972563
by947539
bj607346
bj168416

Mitsuyasu Hasebe cDNA clones at PHYSCObase http://moss.nibb.ac.jp
22o12
8f15
39b12
18d13

Physcomitrella patens Full Length ***NTMC2Type3.1***
Gene 106 Craxton, M. BMC Genomics. 2004 Jul 6;5(1):43

Sequence predicted from genome http://www.ncbi.nlm.nih.gov/Traces
MAKKKLVDGVAKEAKDLWEHLRHEKPELPYLIPIFLLAWILERWLISFSNWVPVFVTVWV
TLQ**^ 0**

YGKHRREREVEGLNNRWRRHILCSQ**^ 0**
PSTPIEPCEWLNKMLMNVWPNFMEPKIVRRLSHIAQ**^ 0**
ITYCILFILQLSMEVEEFSLGTAPPMFGLQGAYWSIDGKQ**^ 0**
PVLNMGFEWDTTEMSVLISAKLGGPLRGKTARIVVNSIHVKGD**^ 0**
LRLLPVLDGQAVLFSFANTPEVRIGLVFGSGANAIPQTELPFISSWL**^ 0**
EMLLVDTLTRTMVEPRRRILCLPAVDLKKKAVGGIFSVTVVSARNLAKLDHRESRNSGNGA
VSNGDGSNHASSNEGSLGSNGSVNKKSEKSRFVEISCEDLTRKTGMQSGPFLHVWNES
YDMVLHDNLGTVRLNVYEQGHNNVNYDFLGSCEVK**^ 0**
VKYVDDDSTIFWAVGPAQSVLISRVPCCGKEVELTIPLENATSGE**^ 0**
LTVKLLLKEWQFSDGSKAVANYNPALVIHDQQNAVGTQPVQPTFTGRKLKISAIEGRNLAPM
DRTGKSDPYLKLFYGK**^ 0**
LIRKTKTVNQDLNPVWNQDFIFQEVSGGEYLKIKCYDADRFGDENLGNARVNLEGIEEGAP
KDVWVPLEKINQGEIHLRIEVVASELLQNPST**^ 0**
NGSENGSHPTGDGCMVEVVLVEARDLVAANWGGTSDPYVSVRYGQIKKRTK**^ 0**
VVYKTLNPAWGQTLEFTDDGSPLVLHVKDYNNILPTVSIGHCEVD
YDKLPPNQTLDQWLPLQGVNKGEIHFQVTRRVPERHLKAASEEQPKLIASSNFSGN**^ 0**
VRSLIRKAMTLAEEEEEIEYIRQMLEELEGAEEERELTVTQLQKDRDLLITKVKELEKAMSGFF

ATGGCGAAGAAGAAGCTGGTAGATGGGGTAGCTAAGGAAGCAAAGGACCTCTGGGAACAT
CTTCGTCATGAGAAGCCGGAGTTGCCCTATTTAATCCCCATCTTCCTCCTGGCGTGGATT
TTGGAGCGCTGGCTAATATCCTTCTCCAATTGGGTTCCCGTGTTTGTTACCGTCTGGGTT
ACTCTTCAGTATGGAAAACACCGACGTGAACGTGAAGTGGAAGGCTTGAATAACCGATGG
AGGCGGCATATACTTTGTAGTCAGCCAAGTACACCTATTGAGCCCTGTGAATGGCTCAAC
AAGATGCTGATGAATGTTTGGCCTAACTTCATGGAGCCCAAGATTGTTCGGAGGCTTTCT
CATATTGCTCAGATAACTTATTGCATACTTTTTATTTTACAGTTAAGTATGGAAGTGGAG
GAGTTTTCACTTGGAACAGCCCCCCCTATGTTTGGGCTTCAGGGGGCGTACTGGTCGATA
GACGGCAAGCAGCCGGTGTTAAATATGGGTTTTGAGTGGGATACTACGGAGATGAGCGTG
CTGATTTCCGCCAAATTGGGGGGACCTTTACGGGGGAAAACCGCTCGCATCGTCGTTAAC
AGTATCCATGTTAAGGGTGATCTACGCTTACTACCTGTTCTGGATGGACAAGCTGTGCTC
TTTTCGTTTGCAAATACCCCTGAAGTTAGGATAGGCTTGGTCTTCGGCAGTGGCGCTAAT
GCTATTCCTCAAACAGAGCTTCCTTTCATTTCATCTTGGCTGGAGATGTTACTCGTTGAT
ACGCTCACTCGTACAATGGTGGAGCCTCGGAGAAGAATCTTATGTCTGCCTGCCGTGGAT
CTGAAAAAAAAGGCAGTAGGGGGCATCTTCTCGGTGACAGTTGTTTCAGCTCGGAACTTG
GCAAAACTTGATCACCGAGAATCTAGAAACTCTGGAAATGGAGCGGTGTCTAACGGTGAT
GGAAGTAACCATGCGAGTAGCAATGAGGGCAGCCTCGGCAGTAATGGATCTGTGAATAAA
AAGTCTGAAAAATCCAGGTTTGTGGAGATTTCGTGTGAGGATTTGACTCGCAAGACAGGC
ATGCAAAGTGGTCCTTTTCTCCACGTGTGGAACGAGTCTTACGATATGGTCTTGCATGAC
AACCTGGGTACTGTACGCCTGAATGTGTATGAGCAAGGCCATAATAACGTGAATTATGAC
TTTCTTGGAAGCTGTGAGGTTAAGGTGAAGTATGTCGACGATGACTCCACTATCTTCTGG
GCTGTGGGTCCAGCACAGAGTGTTCTCATTTCTCGAGTCCCGTGTTGCGGAAAAGAAGTC
GAGCTGACGATCCCTCTTGAAAACGCGACCTCTGGAGAGCTGACCGTGAAGCTATTACTT
AAGGAATGGCAATTTTCTGATGGCTCAAAAGCAGTGGCAAATTACAATCCTGCTCTTGTG
ATTCATGACCAGCAAAATGCGGTGGGTACTCAACCAGTTCAACCAACTTTTACTGGCAGA
AAGCTCAAAATTTCTGCGATCGAAGGCCGAAATCTGGCTCCGATGGATAGGACTGGCAAA
AGTGACCCTTATCTTAAGCTGTTTTATGGCAAGCTTATTCGCAAAACGAAGACCGTAAAT
CAGGACCTGAATCCTGTGTGGAATCAAGATTTTATATTTCAGGAAGTAAGCGGTGGAGAG
TATCTTAAAATCAAGTGTTACGATGCTGACCGCTTTGGCGACGAGAATTTGGGAAATGCG
CGAGTAAATTTGGAAGGAATCGAGGAGGGAGCCCCTAAAGATGTGTGGGTACCTTTGGAA
AAGATTAATCAAGGAGAAATCCACCTTAGAATCGAAGTTGTTGCCTCTGAGTTATTGCAG
AATCCTTCTACGAATGGTTCTGAGAATGGATCACATCCAACTGGAGATGGGTGCATGGTA
GAGGTTGTCCTTGTTGAGGCTCGAGATCTTGTAGCAGCTAATTGGGGAGGAACCAGTGAT
CCTTATGTTAGTGTTCGTTACGGCCAAATCAAAAAACGTACCAAGGTTGTCTACAAAACT
TTAAATCCAGCCTGGGGCCAAACACTCGAATTTACGGATGATGGTAGTCCTTTAGTTCTT
CATGTGAAAGACTACAATAACATTCTCCCCACCGTCAGCATAGGCCACTGTGAGGTAGAT
TATGATAAATTACCACCTAATCAGACGTTAGATCAATGGCTACCATTGCAAGGTGTAAAC
AAGGGCGAAATTCACTTTCAAGTGACTCGTAGAGTGCCCGAGAGGCATCTGAAGGCCGCT
TCTGAAGAGCAGCCCAAGCTCATAGCGTCCTCGAACTTCAGTGGCAATGTACGGTCATTG
ATTCGGAAGGCGATGACTCTCGCTGAAGAAGAGGAGGAAATTGAATACATTCGACAGATG
TTAGAAGAGTTAGAAGGTGCTGAAGAGGAGCGCGAGCTAACCGTTACACAGCTTCAGAAG
GATCGTGATCTCTTAATCACTAAAGTGAAGGAGCTGGAGAAAGCCATGAGTGGATTTTTCTAA

Transcripts
bq827116
bu052380

am410050 partial genomic sequence

Mitsuyasu Hasebe cDNA clones at PHYSCObase http://moss.nibb.ac.jp
67p04

Physcomitrella patens Full Length ***NTMC2Type3.2***

Sequence predicted from genome http://www.ncbi.nlm.nih.gov/Traces
MSKKKMVNDATKEAKELWEHLLHEKQALPYLIPVFLLAWILERWIISFSNWVPVFVTVWA
TLQ**^ 0**
YGKHRREREVEDLNNRWRRHILCSQ**^ 0**
PSTPIEPCEWLNRMLMNVWPNFLEPKLTERFRRIVQ**^ 0**
TTYFVPFLSLQISMEVAEFHLGTAPPMFAHEGAYWSTEGEQ**^ 0**
AVLNMGFEWDTTEMTILISAKLGGPLRGKTASILVNSIHIKGD**^ 0**
LRLLPVLDGQAVLFSFKNTPEVRIGLAFGSGTLSYPQTELPFISSWL**^ 0**
EKLLVDTLNRTMVEPRRRCFSLPAEDRKKKAVGGIFSVTIVSARDLTKSSAQDSRNSSN
AMMSNGDASVHLSNNGSNNGSSHGGSKNGSVNKKSEKLRFVEISCEDLTRKTGMQS
GPFPHVWNETYDMVLHENVGTVHLNVYEQGQNVKYDFLGSCEIK**^ 0**
VKYVDDDSTIFWAVGPAQSVLISRVESCGKEVEFTIPLENVASGE**^ 0**
VTVKLVLKEWQFSDGSKAVANSSPSSSMLGQQNSAGAQSMRPTLTGRKLRISAIEARDLA
PMDRTGKSDPYLKLFYGK**^ 0**
LIRKTKIVNQELNPTWNQDFLFQEVTGGEYLKIKCYDADRFGDENLGSARVNLQGIEEGT
PKDVWVPLEKIKQGEIHLRIEVVAPELSQIPSS**^ 0**
NGSENGSHPTGDGCVVEIVLVEARDLVAADWGGTSDPYVSVRYGQIKKRTK**^ 0**
VVYKTLTPTWGQTLEFPDDGSPLVLHVKDYNNILPTVSIGHCDVDYEGLPPNQMLDSWL
PLQGVNKGEIHVKVTRKVPERLVKAASEEQPKPTFAPTYSGN**^ 0**
VRSLLRKAMALAEEEEDIEEIRQMLEELEGAEEEREVTISQLQKDRDVLIAKVRELEKAMSGFF

ATGTCGAAGAAGAAGATGGTGAATGATGCAACGAAGGAAGCAAAGGAACTCTGGGAACAT
CTACTTCATGAGAAGCAGGCTTTGCCCTATTTAATCCCCGTCTTCCTCTTGGCGTGGATT
TTGGAGCGGTGGATCATATCATTCTCCAATTGGGTTCCCGTGTTTGTTACCGTCTGGGCC
ACTCTCCAGTATGGTAAACATCGACGTGAGCGTGAAGTGGAGGATCTAAACAACCGATGG
AGAAGGCATATTCTTTGTAGTCAGCCAAGCACACCTATTGAGCCCTGCGAATGGCTCAAC
AGGATGCTTATGAATGTCTGGCCTAACTTCTTGGAGCCCAAGCTTACTGAAAGATTTCGT
CGAATTGTCCAGACAACTTATTTTGTACCTTTTCTGTCCCTACAGATCAGTATGGAAGTC
GCGGAATTCCATCTAGGAACAGCCCCACCTATGTTCGCGCATGAGGGAGCTTACTGGTCG
ACGGAAGGCGAGCAGGCGGTATTAAACATGGGTTTCGAGTGGGATACTACTGAAATGACT
ATTCTGATTTCTGCCAAATTGGGGGGACCTCTGCGCGGGAAAACCGCTTCCATCTTAGTC
AATAGCATCCATATCAAGGGTGATTTGCGCTTACTTCCTGTTCTGGACGGACAAGCAGTG
CTTTTTTCCTTCAAAAATACCCCGGAGGTCAGGATAGGCTTGGCCTTCGGCAGCGGCACC
CTGTCTTATCCTCAAACAGAGCTTCCTTTTATCTCATCTTGGTTGGAGAAATTACTAGTC
GACACTCTTAATCGCACAATGGTGGAGCCCCGAAGAAGGTGTTTCTCTCTTCCTGCCGAG
GATAGGAAAAAGAAGGCAGTTGGGGGCATCTTTTCGGTTACTATTGTTTCAGCTCGAGAT
TTGACAAAAAGCAGCGCCCAAGACTCTAGAAACTCTAGCAATGCAATGATGTCCAACGGT
GATGCCAGTGTCCATTTGAGCAACAATGGAAGTAACAATGGAAGTAGCCATGGTGGCAGC
AAGAATGGATCTGTAAACAAGAAGTCTGAGAAACTGAGGTTTGTGGAGATTTCATGTGAG
GATTTGACCCGCAAGACAGGCATGCAGAGTGGTCCCTTCCCTCATGTATGGAACGAGACC
TACGACATGGTTTTGCATGAAAATGTGGGCACTGTGCACCTAAATGTGTACGAGCAAGGC
CAAAATGTCAAATATGACTTTTTAGGAAGTTGCGAAATTAAGGTGAAGTACGTCGACGAT
GACTCCACGATCTTCTGGGCTGTGGGTCCAGCTCAGAGTGTTCTTATTTCTCGAGTTGAA
AGCTGTGGAAAAGAAGTCGAGTTCACTATCCCCCTCGAAAACGTAGCATCTGGAGAGGTG
ACCGTAAAACTTGTCCTTAAAGAATGGCAATTTTCTGATGGATCGAAAGCAGTAGCAAAT
TCTTCTCCGAGCAGTTCAATGCTTGGCCAACAGAACTCAGCGGGAGCTCAATCAATGCGA
CCAACTCTTACTGGTAGAAAGCTCCGCATCTCTGCAATTGAGGCCCGAGATTTGGCTCCA
ATGGACAGGACTGGTAAAAGTGATCCATATCTAAAGTTGTTTTATGGCAAGCTTATTCGC
AAGACAAAAATCGTGAATCAGGAACTGAATCCCACGTGGAATCAAGATTTTTTATTTCAA
GAGGTAACCGGTGGAGAGTATCTCAAAATCAAGTGTTATGATGCTGACCGCTTTGGCGAC
GAGAATTTGGGAAGTGCACGAGTGAATTTGCAAGGAATCGAGGAGGGAACTCCTAAAGAT
GTGTGGGTACCCTTGGAGAAAATTAAGCAAGGAGAAATTCACCTTAGAATCGAAGTTGTT
GCCCCTGAGCTCTCACAGATTCCTTCCAGTAATGGCTCCGAAAATGGATCACATCCAACT
GGGGATGGATGCGTGGTGGAGATCGTGCTCGTTGAGGCCCGAGACCTTGTAGCAGCGGAC
TGGGGAGGAACAAGTGATCCGTATGTTAGTGTGCGATACGGTCAAATCAAAAAACGTACT
AAGGTTGTCTACAAAACATTAACTCCAACCTGGGGTCAAACTCTGGAATTCCCAGATGAT
GGAAGTCCTTTAGTCCTTCATGTAAAGGACTACAACAACATTCTCCCTACTGTTAGCATA
GGTCATTGTGATGTGGATTATGAGGGACTACCTCCAAACCAAATGCTGGATTCATGGCTA
CCATTGCAAGGTGTGAACAAGGGCGAAATCCATGTTAAGGTAACTCGCAAAGTTCCAGAG

AGGCTTGTCAAGGCCGCTTCTGAAGAGCAGCCTAAGCCCACATTTGCCCCGACCTACAGT
GGGAATGTGCGGTCGTTGCTTAGAAAGGCCATGGCTCTCGCCGAAGAGGAGGAGGACATT
GAAGAGATCCGACAAATGTTGGAAGAGTTAGAAGGTGCTGAAGAGGAGCGTGAGGTTACC
ATTTCACAGCTTCAGAAGGACAGGGATGTCCTAATCGCTAAAGTAAGGGAGCTGGAAAAA
GCAATGAGTGGATTTTTC<u>TAG</u>

Transcript
bj599198

Mitsuyasu Hasebe cDNA clones at PHYSCObase http://moss.nibb.ac.jp
70n02
25d20

Physcomitrella patens Full Length ***NTMC2Type4.1***

Sequence predicted from genome http://www.ncbi.nlm.nih.gov/Traces
MVLVGLIFGWLVGVALICGLKVMMDRRSRKRTKK**^ 0**
VAAIELFNLIDEVELKKLCSDSYPNHVSFTTYEK**^ 0**
VNWLNSMLEKFWPSILT**^ 0**
ATEDMVKMKLAPVLESYKPTGISALTLDKFQLGKTPPQID**^ +1**
GIRIQRLVKGQVHMDMDFKWAGTGDIVLNIGFMGSKLPVQ**^ 0**
LKNLSFFATIRVIFQLSEEIPCISALVVALLSK**^ 0**
PKFQVSYKLNVLGGFNNNLPGLSDMIE**^** 0
DMVESSIADQLEWPHRIVLPVGDTPANVI**^ +2**
SDLGLKPQGQLKVTVVKAENLKNQEAIGKSDPYVKLYVRVLFKEKTTTIGDNLNPVWNQ
EFLLDVEDTETQALVLQ**^ 0**
IMDEDVGSDKQMGIASIPLNELVPDTEVLITQKVLKSLDTARVKDKGDRGTITVK**^ 0**
LKFHPYTEEEQEIAILREKEMLAAKEALKNSGVVGGAMDAVGGGVKLVGTGISTAGSTGVKL
VGTGVGAVGSGVGLVGSGVVRAGRMMSSGVKRLSSSSRLATSVATPVNGSPLHEVNGTQKVKE

<u>ATG</u>GTGCTCGTTGGGCTTATTTTCGGATGGCTGGTTGGGGTCGCACTCATCTGTGGGTTA
AAAGTTATGATGGACAGACGGAGCAGGAAGAGAACAAAGAAGGTTGCTGCGATTGAGCTA
TTCAACCTCATAGATGAAGTGGAACTCAAGAAGCTTTGTTCTGATAGTTATCCTAACCAC
GTCTCATTCACCACCTACGAAAAGGTGAACTGGTTGAACTCTATGTTGGAGAAGTTCTGG
CCGTCAATATTAACGGCCACCGAAGATATGGTAAAAATGAAGCTTGCTCCAGTGTTAGAA
TCTTACAAGCCTACCGGGATTTCGGCGCTCACATTGGATAAGTTTCAACTTGGGAAAACA
CCTCCCCAAATAGATGGGATTAGAATACAGAGGTTGGTCAAGGGTCAAGTGCACATGGAT
ATGGATTTCAAGTGGGCTGGCACTGGTGATATCGTACTCAACATTGGTTTCATGGGCTCC
AAATTGCCCGTTCAGTTAAAAAACCTGTCCTTCTTCGCAACTATTCGTGTCATCTTTCAA
CTATCAGAAGAAATTCCGTGCATCTCCGCTCTAGTTGTCGCTCTTCTTTCTAAGCCAAAG
TTTCAAGTTAGTTATAAACTAAATGTTCTTGGAGGATTCAACAACAACCTCCCTGGCCTA
AGCGACATGATTGAGGATATGGTGGAGAGTTCTATTGCGGATCAGTTAGAGTGGCCTCAC
AGAATTGTACTTCCTGTTGGAGACACTCCTGCGAACGTTATAAGCGATCTGGGATTAAAG
CCCCAAGGCCAGTTGAAGGTCACCGTTGTGAAGGCTGAAAATTTGAAGAACCAAGAGGCT
ATTGGAAAGTCGGATCCTTACGTTAAACTGTATGTACGAGTGCTATTCAAAGAGAAAACC
ACCACGATTGGGGACAATCTGAATCCAGTATGGAATCAAGAATTCCTGCTTGATGTGGAG
GATACCGAAACACAGGCTCTCGTGTTGCAGATCATGGACGAAGACGTTGGATCAGATAAG
CAAATGGGAATTGCTTCAATTCCTCTGAATGAGTTGGTACCGGACACTGAAGTTTTGATT
ACTCAAAAAGTACTAAAATCTTTGGACACTGCGAGAGTTAAGGACAAAGGTGATCGCGGG
ACCATCACAGTGAAACTGAAATTCCACCCGTATACCGAGGAGGAGCAGGAGATCGCTATT
TTGCGTGAGAAGGAAATGCTCGCAGCAAAAGAGGCACTCAAGAATAGTGGTGTTGTTGGA
GGAGCTATGGATGCTGTGGGAGGAGGTGTGAAGCTGGTGGGGACAGGAATTTCCACTGCT
GGTTCTACTGGAGTGAAGCTGGTTGGCACTGGTGTTGGAGCTGTAGGCTCGGGTGTGGGT
TTGGTAGGTTCCGGAGTGGTCAGAGCTGGCAGGATGATGAGCAGTGGAGTGAAGAGGCTG
TCAAGCAGCAGCAGGCTTGCTACTTCAGTCGCTACCCCAGTGAATGGTAGTCCTTTGCAT
GAAGTGAATGGCACTCAGAAGGTCAAAGAGGTG<u>TAA</u>

Transcripts
bj160282
bu051584
bj166677
bj978020
bj167871
bj171906
bj605388
bj167628

bj168539
bj585280
bj954168
bj591499
bj164611
bj168461

Mitsuyasu Hasebe cDNA clones at PHYSCObase http://moss.nibb.ac.jp
12l11
121f21
44f07
16j09
30i19
33j15
15m05
18j11
136k19
93n15
27g11
21h15
44i03
21e10
18f14
41l17

Physcomitrella patens Full Length ***NTMC2Type4.2***

Sequence predicted from genome http://www.ncbi.nlm.nih.gov/Traces
MVLSGLIIGWLVGVVIIARWRYMMDKRNKKRIQK**^ 0**
ATGIELLNVIDEMDLKKLCEQSLPNHISFLTFEK**^ 0**
VEWLNKTLDKFWPSIVE**^ 0**
ATEKEVKMRLGPMLVAYKPVEISSLTLDKFHLGKTPPKID**^ +1**
GVRIQRFREGQVHMDMEFKWGGSGEIVLNIGFMRTKLPVQ**^ 0**
LKNLSFFATIRVIFQLSEVIPCISALVVALLPK**^ 0**
PKFQIGYKLNVIGGNNANLPGLGDMIE**^ 0**
DLVNSTVADQVEWPHRIVVPVGDTPADIM**^ +2**
SDLGLKLQGQLKVKVFKAEKLKNKETVGRSDPYVLLFVRVLFKKKTKVIHSNLNPEWMES
FLFNVEDTETQTLILQ**^ 0**
VMDEDIGADKELGIASVPLHDLKPDTEIEITQKLLKSLDTAKVKDKSDRGSITIS**^ 0**
LKYHPYTKEEQVAAMLAEQNELKAREQMNNGVIGGAMDAVGGGVKMVGSGISAVGSGGS
KLVGTGVGAVGSSVGIVGSGVVKASRLVSSGVKRLSSSNRLVSTTSTPVNGSPMHEVNGISKLKEV

ATGGTGCTTAGTGGGCTTATAATCGGATGGTTGGTGGGAGTGGTAATTATTGCCAGGTGG
AGGTATATGATGGACAAACGAAACAAGAAGAGAATTCAGAAGGCTACTGGCATAGAGCTT
TTGAACGTCATAGACGAAATGGACCTCAAAAAGCTTTGTGAACAAAGCTTACCAAATCAT
ATTTCGTTCTTAACCTTTGAAAAGGTGGAATGGTTGAACAAAACGCTGGATAAGTTCTGG
CCGTCTATAGTAGAGGCCACTGAAAAGGAGGTGAAGATGAGGCTCGGACCAATGTTAGTG
GCGTATAAGCCTGTTGAGATTTCATCGCTCACGCTGGACAAGTTTCACCTAGGAAAAACG
CCCCCCAAAATTGATGGGGTAAGAATACAGAGGTTTCGTGAAGGTCAAGTGCACATGGAT
ATGGAGTTCAAGTGGGGTGGCAGTGGTGAAATTGTACTCAACATAGGTTTTATGAGAACC
AAACTGCCTGTTCAGTTGAAAAATCTTTCCTTCTTCGCAACAATTCGTGTTATCTTCCAA
CTTTCAGAAGTGATTCCGTGTATCTCCGCCCTTGTTGTCGCTCTTCTGCCTAAGCCAAAA
TTTCAAATTGGTTACAAACTGAATGTTATCGGAGGAAACAATGCTAACCTCCCTGGCCTG
GGTGACATGATTGAGGATTTGGTGAACAGTACTGTTGCGGATCAAGTGGAGTGGCCACAC
AGAATCGTAGTTCCTGTTGGCGATACACCTGCAGACATCATGAGTGACTTAGGTTTAAAG
CTCCAAGGCCAGTTGAAAGTTAAAGTATTTAAGGCTGAAAAACTGAAAAACAAGGAGACT
GTTGGAAGATCAGATCCTTACGTTTTACTGTTTGTACGAGTGCTTTTCAAGAAAAAAACT
AAAGTCATTCACAGCAATCTGAATCCGGAATGGATGGAGAGTTTTTTGTTTAATGTCGAG
GACACTGAAACTCAGACTCTCATACTGCAGGTCATGGACGAGGACATTGGAGCAGATAAG
GAACTTGGCATAGCTTCAGTGCCGCTACATGATTTGAAACCAGACACTGAAATTGAGATT
ACTCAAAAGCTGCTCAAATCTTTGGACACTGCGAAGGTTAAGGACAAAAGTGATCGTGGT
TCCATCACAATTAGTCTCAAATACCACCCTTACACCAAAGAGGAGCAGGTGGCTGCTATG
CTAGCCGAGCAGAACGAGCTCAAAGCAAGAGAACAAATGAACAATGGTGTAATTGGAGGC
GCTATGGATGCCGTGGGAGGAGGTGTGAAGATGGTGGGCTCAGGAATTTCCGCTGTTGGT
TCTGGTGGATCAAAGCTTGTGGGTACTGGTGTTGGAGCTGTAGGGTCGAGTGTGGGTATT
GTAGGTTCGGGGGTTGTCAAGGCTAGCAGGTTGGTAAGCAGTGGCGTGAAAAGGCTGTCA
AGTAGCAACAGACTTGTTAGTACAACTTCAACTCCAGTCAATGGAAGCCCAATGCATGAA
GTGAATGGCATTTCGAAATTGAAAGAGGTTTAA

Transcripts
bi741064
bi487969

Physcomitrella patens Full Length ***NTMC2Type4.3***

Sequence predicted from genome http://www.ncbi.nlm.nih.gov/Traces
MAACVLEPNRPRIGSTILGHLVVGFVVGVGLVAGFKYFSDKRSKWRLQK**^ 0**
IAGIHLLSLADEFDFKRLCKESYPSHISFLTFEK**^ 0**
VRWVNEILEKIWPFVVE**^ 0**
ATEKPGKEWLGPVVEFYRPTRISSLTVEKFHLGKAAPHID**^ +1**
GIRVQSLRKSQVHLDMDFKWGSEGDVVLNAAIMGSNVSIQ**^ 0**
LKDLSFYATIRLIFQLSDQIPCISAYVVAVLPD**^ 0**
PKYRIDYNLKVGGGNTAAIPGLGDMIE**^ 0**
DLVHSCITDMLEWPRRLIFPIGDTPMNVT**^ +2**
SDLELKPQGKLTVTVVRANDLKNMETIGISDPYVVLYVRVLFKKKTRVIHHNLNPEWNDPD
SVFHFDVEDTETQTLVLQ**^ 0**
VKDEEHFGTDKELGVTVVPLCVLKPDTEIEIRKKLAPSLDTVRVKDEGDRGSITVK**^ 0**
LLYHLYTETEQLRAMV
EEKEEIQAKEDLKNAGVIGGNMDALTKSLKPSRNGTETVESGVMKVGRMMSKGIKSFIHD
SLSPR

ATGGCAGCGTGCGTGTTGGAACCAAATAGACCAAGGATTGGCAGCACGATTCTTGGTCAT
CTTGTCGTTGGATTCGTGGTCGGGGTGGGACTCGTTGCAGGTTTTAAATACTTTTCGGAC
AAACGAAGTAAATGGAGATTACAGAAGATTGCTGGAATACATCTCTTAAGCCTAGCAGAT
GAATTTGACTTCAAGAGGCTCTGTAAGGAAAGTTATCCGAGCCATATTTCATTCCTAACT
TTTGAGAAGGTTAGATGGGTAAATGAAATTCTGGAGAAAATCTGGCCATTTGTAGTAGAG
GCCACGGAGAAACCGGGGAAGGAGTGGCTTGGACCAGTCGTAGAATTCTACAGGCCTACT
AGGATTTCATCTCTCACAGTCGAAAAGTTCCATCTTGGAAAAGCGGCTCCCCATATTGAT
GGAATTAGGGTGCAAAGCTTGCGTAAAAGTCAGGTGCACTTGGACATGGATTTCAAGTGG
GGTAGTGAAGGCGATGTTGTGCTTAATGCGGCCATCATGGGCTCCAATGTTTCTATTCAG
TTAAAAGATCTGTCGTTCTATGCAACAATTCGGCTGATCTTCCAGCTTTCAGACCAGATT
CCCTGCATCTCAGCCTATGTTGTTGCTGTTCTGCCTGATCCAAAATACCGAATCGATTAC
AACCTAAAAGTTGGTGGAGGCAACACTGCAGCCATTCCTGGATTAGGTGACATGATTGAG
GATTTGGTGCATAGCTGTATTACAGATATGTTGGAGTGGCCCCGCAGGCTTATATTTCCT
ATTGGTGACACCCCTATGAACGTTACAAGTGATCTGGAATTAAAGCCTCAAGGCAAGTTA
ACGGTCACTGTGGTGAGGGCTAACGATCTGAAAAATATGGAAACAATTGGCATATCAGAT
CCGTATGTTGTGCTTTATGTACGGGTTCTCTTTAAGAAAAAAACTAGAGTCATCCATCAC
AACCTAAATCCAGAATGGAATGATCCTGATTCAGTCTTTCATTTTGATGTGGAGGATACT
GAAACACAGACTCTGGTGTTGCAGGTCAAAGACGAGGAACATTTTGGAACAGATAAAGAA
CTTGGTGTAACTGTAGTGCCACTGTGTGTTTTGAAACCTGATACCGAAATCGAGATAAGA
AAAAAATTAGCTCCATCTTTGGACACTGTTAGAGTAAAAGACGAAGGTGACCGTGGCTCA
ATTACTGTCAAACTCTTATACCATCTGTACACGGAAACAGAGCAGCTCAGGGCTATGGTT
GAAGAAAAAGAAGAGATCCAGGCAAAAGAAGATCTGAAGAATGCAGGTGTAATTGGAGGG
AATATGGATGCTTTGACTAAAAGTCTCAAGCCTTCGAGGAATGGCACCGAAACCGTAGAG
TCGGGTGTGATGAAGGTTGGGAGGATGATGAGTAAGGGAATCAAAAGCTTTATACATGAC
AGTTTGTCGCCACGCTGA

Transcripts
bj166794
bj609761
bj598767
bj954891

Mitsuyasu Hasebe cDNA clones at PHYSCObase http://moss.nibb.ac.jp
4k07
13b13
54h10
24c13
48o05
23o05

Physcomitrella patens Full Length ***NTMC2Type5.1***

Sequence predicted from genome http://www.ncbi.nlm.nih.gov/Traces
MVTLVVTSVKVFPDSTRNPQLPVASYTVGTLHSRPLGTCLTAQIHGPKNLFHGRPYKLDN
HFQSKCRIGVRALPGKRKWRLLASVADGSKGDTKSGQLLNRFLKRGNPEIVVSDDSQELA
NDASNSRQEPQKKMQEEVEIPDDGTEPSLKPLGLVKAAKATKDRLLEAALKQVEEVKGFA
TNTSWTNYFTSVDASPGTPLIPFLGDSLLGLGGLVLVAAALASWLRKARLGKKVLNTKEA
GPTKPPRTKDILNAQAAVAAPIALSMLLQSDMKKKESAEWLNMVVGKVWNLYRRSLETAT
IEAVQPVIDEIPEKPPFVERVILKQFFLGDEPVTLRTIERRTSRRANDL**^ +2**
QYHVGLRYTGNSRMVFSLKLKFGFLPIEIPVAIRGLDLDGEVWVKLRLIPTEPWVGTATWA
FVAPPKVTLALVPFRLFNLM**^ +1**
AIPLLN**^ +2**
IFLTNLLTRDLPLLFVRPNKQIVNYLKGKVAGPLPKDFKDSAVGLNGFAGELSVTLIEARKL
NYFPI**^ +1**
GKTDPYVVFLLGEQTFRSKKNSKTSLIGPPGAPVWNQ**^ 0**
DFRMLVVDPKTQKLRIRVRDSVDYLGLANITVGYST**^ 0**
ISIDDLEDTVSVDKVITLKNGRWFFLGRNAGELSLRLTYKAYVAEEEEVDTTSPVKSTVLN
EIGELISKEMEETEVSPAKRQLRDKIVTSVDAQKDDTGFRFTNPMLTIGTTRKPPGVTSGS
GLPVDPSGSINEPLKSNSSTNEPEVAPILSNPALEEQEKREGK**^ +1**
NAALLWLAIATGVALVVALDLNISNLFNP

ATGGTTACACTCGTGGTTACATCTGTGAAGGTCTTCCCAGACTCTACACGTAATCCTCAG
CTCCCTGTAGCATCTTACACTGTAGGAACGTTGCACTCTCGTCCGCTGGGCACCTGTTTA
ACTGCTCAGATTCATGGCCCTAAAAATTTATTTCATGGTAGACCATATAAACTGGATAAT
CATTTCCAGAGCAAGTGTAGAATTGGAGTCAGAGCTTTACCTGGGAAAAGAAAATGGCGT
TTATTAGCCAGTGTAGCAGATGGATCGAAGGGTGATACAAAGAGTGGACAGCTTCTTAAT
CGGTTTTTGAAGAGAGGAAACCCAGAGATAGTCGTGAGCGATGACTCACAAGAGTTGGCG
AATGACGCCTCTAATTCCAGACAGGAGCCTCAGAAGAAAATGCAAGAGGAGGTTGAAATT
CCGGACGATGGCACTGAGCCATCTCTAAAACCATTGGGACTTGTAAAAGCAGCAAAAGCC
ACGAAAGATAGACTGTTGGAAGCAGCACTGAAGCAAGTAGAGGAGGTCAAGGGGTTTGCC
ACTAATACGTCTTGGACGAATTACTTCACCAGTGTAGATGCTTCACCTGGTACTCCACTT
ATTCCCTTCTTAGGTGACAGCTTGCTGGGACTGGGAGGTTTAGTGCTTGTTGCAGCAGCT
TTAGCTAGTTGGTTGAGGAAAGCGCGGCTTGGCAAGAAGGTTTTGAATACAAAGGAGGCA
GGTCCAACTAAGCCACCTCGCACTAAAGACATTCTTAATGCTCAGGCTGCTGTGGCAGCT
CCCATTGCACTGTCGATGTTACTGCAAAGTGACATGAAGAAGAAAGAGAGCGCAGAATGG
CTGAATATGGTGGTCGGTAAGGTTTGGAACTTATATCGTAGGAGCTTGGAAACCGCAACG
ATTGAGGCTGTGCAGCCCGTTATTGACGAAATTCCGGAGAAACCACCTTTTGTGGAGCGA
GTGATCCTCAAACAGTTCTTTTTGGGAGATGAACCAGTAACTTTGCGAACTATCGAGCGT
CGTACATCCCGGCGTGCTAATGACCTCCAGTACCACGTTGGATTACGATACACGGGCAAC
TCTCGCATGGTATTTTCTCTAAAATTGAAGTTCGGTTTTTTACCGATCGAAATTCCAGTT
GCTATCCGGGGTTTGGATCTAGATGGAGAAGTTTGGGTGAAGCTTAGATTAATTCCCACA
GAACCCTGGGTAGGAACTGCAACCTGGGCCTTTGTTGCTCCACCCAAAGTCACTTTGGCT
TTGGTTCCATTTCGGCTCTTTAATCTCATGGCTATACCATTACTTAATATATTCTTGACA
AATTTGCTCACTCGAGATCTTCCATTACTCTTTGTCCGACCCAACAAACAAATAGTCAAT
TATCTTAAAGGAAAAGTGGCTGGCCCTCTTCCGAAGGATTTCAAGGATTCCGCGGTGGGT
TTAAACGGTTTTGCAGGGGAGCTTTCAGTAACACTAATTGAAGCACGGAAATTGAATTAT
TTTCCGATAGGTAAGACAGATCCGTATGTGGTTTTTTTGTTAGGAGAACAAACTTTTCGA
AGTAAAAAAAATAGTAAAACATCACTTATTGGACCGCCAGGCGCACCAGTATGGAACCAG
GACTTTCGTATGCTTGTTGTCGACCCCAAGACGCAGAAACTCAGAATAAGAGTCAGAGAT
TCTGTTGATTATCTGGGTTTGGCTAATATCACAGTCGGCTATAGTACGATTTCTATCGAT
GATCTTGAAGACACGGTTTCTGTTGACAAAGTTATTACTTTGAAGAATGGTCGGTGGTTT
TTCCTTGGTCGAAATGCCGGGGAGCTTTCGCTTCGCTTGACATATAAAGCATATGTTGCA
GAAGAAGAGGAAGTTGATACTACGAGTCCCGTAAAGAGCACGGTTCTGAATGAAATTGGA
GAACTTATTAGTAAAGAAATGGAGGAAACAGAAGTGAGTCCCGCGAAACGTCAGCTGCGG
GACAAAATTGTCACATCAGTTGATGCTCAAAAAGATGATACAGGGTTTCGGTTCACTAAT
CCTATGCTGACTATAGGTACCACAAGAAAACCACCAGGTGTAACAAGTGGATCTGGACTT
CCTGTTGATCCCAGCGGTTCGATAAATGAACCTCTAAAATCGAATTCTTCAACAAATGAA
CCTGAAGTAGCACCAATCCTGAGTAATCCTGCCTTAGAAGAGCAGGAAAAAAGGGAAGGG
AAAAACGCGGCTCTCTTATGGTTGGCTATTGCAACAGGAGTTGCTTTAGTGGTAGCTCTC
GATCTCAATATATCTAATTTGTTCAATCCTTGA

Transcript
bq039593

Physcomitrella patens Full Length ***NTMC2Type5.2***

Sequence predicted from genome http://www.ncbi.nlm.nih.gov/Traces

MLEMAQAHQFLKIMYHTQSTSCQNTSSQSMVLLLPVIDENHEQFVTLRAPSSRSLGSYDT
AGSMKYHRSKVLPTSRICRVPESQLRRVDQSWKPQIGRNIPWSLATTGPHAFMTNNRMSI
SYSARQRKDSTVVHAQAVHRSISFLPGAGPLRDFLFALIGAVFAFLFAGMLAKVWSLEER
YFRATSVERGTANMPEMGSIQGQKESVEWVNMVIHKVWKVYRRSLEVWLVQLLQPAIDNL
GKPNWVKRVKIVELNLDYEPIIVRNVQRRAS**^ +2**
RRANDLQYHFGLRYAGGARCLLNLKLGRAGFETSIPVGVYELDVDAELWVKLRLAPVSP
YVGTLSLAFVRLPTIKLVLAPFRVVNLFSIPFLNNFLSKLLTVDLPRLLVLPRHITFDFLPQG
QNVMDSMKAMEESMDESIASGVLDLLKTTSTEPAVPQDDPSEVFVGELSVTICDARGLP
IRGFTGWSNPYCILSLGDQVLESKRNKETSHPSGPKDPVWNQDFLLLVEDPRRQRLMLR
VRDSAMTLNPNIGYCEINLAELR**^ 0**
DCVPRTMWLNLKRDGLFGLKKVPGRVRLALTYKSYVDEEEEDSDENEGSFSPYIKVYGDS
GAETVEDIGVSLGEEIAASIEAKAKNGEFESDDEAEIVRTRRKDSSVEINSSPKAHGNGHGR
VSGSNRNADRVRRETGAARTRDQSESHSIRYSNKDASENTFRGFGNNGDVRVPVRVDGS
DSGEHTGVSGNAIVGVWDREMEMLATRSSKAQEGSVGHASRTQSRRSSFYSEASGTPVV
EHGATSPLSRSSDPKEENPRFEKETGELGSKDDPSSDLSKTEGNKLLWLCMFTTVAYIIGCS
LHISNPLHP

ATGTTAGAAATGGCGCAAGCACACCAGTTTCTCAAAATCATGTACCATACACAATCAACA
TCATGTCAGAATACATCCTCTCAATCAATGGTATTGCTATTACCTGTAATAGATGAAAAC
CATGAGCAATTTGTTACGTTAAGGGCACCATCCTCTAGGTCTCTTGGGAGCTATGATACT
GCCGGGTCAATGAAGTATCATCGGTCTAAAGTGTTGCCAACTTCTCGAATTTGCCGAGTG
CCTGAGTCTCAGCTGCGTCGAGTAGACCAGTCTTGGAAACCTCAGATTGGTAGAAATATA
CCCTGGAGCCTTGCTACTACAGGGCCGCATGCGTTCATGACAAATAATAGGATGTCGATT
TCGTATTCTGCAAGACAGAGAAAAGATAGCACAGTGGTTCATGCGCAAGCTGTTCATCGC
TCGATCTCCTTCCTGCCGGGAGCTGGTCCACTTCGAGATTTCCTATTTGCATTAATTGGA
GCAGTATTTGCCTTTCTTTTTGCGGGAATGTTAGCGAAGGTTTGGAGTTTGGAAGAACGC
TACTTTAGGGCTACGTCTGTCGAGAGAGGAACTGCAAACATGCCAGAGATGGGCTCGATC
CAAGGTCAAAAGGAATCCGTGGAATGGGTGAACATGGTGATTCACAAAGTCTGGAAAGTG
TACAGGCGGAGCCTCGAGGTATGGTTGGTTCAGCTTCTGCAACCGGCTATTGATAATTTG
GGAAAGCCCAATTGGGTAAAGAGGGTGAAAATTGTAGAACTCAACTTAGATTATGAGCCA
ATTATTGTGCGCAATGTACAGCGCCGTGCATCCAGACGAGCGAACGATTTGCAATATCAC
TTCGGGTTGCGATACGCTGGCGGTGCGAGGTGTTTGCTTAACTTGAAACTTGGGCGGGCA
GGCTTCGAGACCTCCATTCCAGTTGGAGTGTATGAGCTTGACGTGGATGCAGAGCTTTGG
GTGAAACTGCGATTAGCTCCAGTGAGTCCATACGTTGGGACTCTCTCTCTGGCTTTTGTG
CGATTGCCGACAATCAAACTTGTCTTGGCCCCCTTTAGAGTTGTTAACCTCTTTTCCATC
CCATTTTTAAACAATTTTCTCTCGAAGCTGTTGACAGTGGATCTTCCTCGCCTACTGGTT
CTTCCACGACACATTACCTTCGACTTCTTGCCCCAAGGTCAGAATGTCATGGATTCCATG
AAGGCAATGGAAGAATCTATGGATGAGTCGATTGCATCAGGCGTTCTGGACCTGCTGAAA
ACTACGTCTACCGAGCCGGCTGTTCCACAGGACGACCCGAGCGAGGTCTTTGTTGGGGAG
CTCTCTGTCACCATCTGTGACGCTCGTGGCCTTCCTATCCGTGGATTTACAGGTTGGAGT
AATCCTTATTGTATTCTCTCACTGGGAGACCAAGTTTTAGAAAGCAAGAGAAATAAAGAA
ACCTCACACCCATCTGGTCCCAAGGACCCAGTTTGGAATCAAGATTTCCTGTTATTGGTC
GAGGATCCTAGGAGACAGAGGCTGATGCTTCGTGTTCGGGACAGCGCAATGACCTTAAAC
CCCAACATAGGCTATTGTGAGATAAATCTGGCAGAGCTCAGGGATTGCGTGCCACGCACC
ATGTGGTTGAACTTGAAGCGAGATGGTCTGTTTGGGCTCAAAAAGGTGCCTGGAAGGGTT
CGATTAGCTCTCACGTACAAATCCTATGTTGACGAGGAAGAGGAGGACAGTGACGAAAAT
GAGGGCTCCTTCTCTCCATACATCAAGGTTTACGGTGATTCAGGGGCCGAAACTGTAGAA
GACATTGGTGTGAGCCTTGGTGAAGAGATAGCTGCGAGTATAGAGGCCAAAGCGAAAAAT
GGAGAGTTTGAAAGTGATGACGAAGCAGAAATAGTTCGGACGCGAAGGAAGGATTCCAGT
GTTGAGATCAACAGTAGTCCAAAGGCCCATGGTAATGGACATGGTAGAGTTTCAGGATCG
AATAGGAATGCAGATAGGGTTAGGCGAGAAACCGGAGCTGCGCGGACTAGAGACCAGTCA
GAATCCCATTCAATACGCTATTCGAATAAAGATGCTAGTGAAAATACATTCAGGGGCTTT
GGTAACAACGGAGATGTTCGCGTGCCTGTGAGGGTAGATGGCAGTGATAGTGGAGAGCAC
ACAGGCGTATCTGGAAATGCCATAGTTGGTGTGTGGGACAGAGAAATGGAGATGTTGGCA
ACAAGGTCCTCTAAAGCTCAGGAAGGCTCGGTGGGGCATGCGTCTCGAACTCAGAGTAGG
AGATCAAGCTTTTATTCAGAAGCCAGTGGAACTCCTGTAGTCGAACATGGGGCCACTTCT
CCCTTATCCAGGAGTTCTGATCCGAAGGAAGAGAACCCTAGATTTGAAAAGGAGACAGGA
GAATTAGGTTCAAAAGATGACCCATCTAGTGACTTGTCAAAAACCGAGGGTAACAAATTA
CTCTGGTTGTGTATGTTTACAACTGTTGCTTACATTATTGGGTGTAGCTTACATATATCA
AATCCATTACATCCATGA

Physcomitrella patens Full Length ***NTMC2Type5.3***

Sequence predicted from genome http://www.ncbi.nlm.nih.gov/Traces
MAQAHPVPNIMYHTHRQSCQSITSSSPVLLLRELDGKHQQFVNLNSISSSFYGSCGNAKL

MKHHQLRVLPSSRICRVPESQLCRVDQPRKPQIDRNISWSLATAGSHVVMTDFTRSNLYP
TRQGRYSAVVHAQAVHRSFSLLPGAGPLRDFLFALIGAVFAFFFAGFLARLWSLEERYFR
ATSAERGSATMPEMGSNQGQKESVEWVNMVIHKVWKVYRRSLQVWLVQLLQPAIDNLGKP
NWVKRVKIVELNLDYEPIIVRNVQRRAS**^ +2**
RRANDLQYHFGLRYAGGARCLLNLKLGRAGFETFIPVGVYELDVDAELWVKLRLAPVKPYV
GTLSLAFVRLPTIKLVLAPFRVVNLFSIPFLNNFLSKLLTIDLPRLLVLPRHITFDFLPQGQNVMD
SMEAMEASVESGAMDESIASGVLDLLKTASSEPAVPQKDPSEAFVGELSVTICDARGLPIRGF
TGWSNPYCILTLGDQVLESKRNKETSQPSGPKDPVWNQDFLLLVEDPRRQRLMLRVRDSTM
TLNPNIGYCYVNLAELR**^ 0**
DCVPRTMWLNLRRDGLFGPKKVPGRVRLTLTFKSYVDREDDIDENAGSFSPYIKVFKDSGAE
TVEDIGVSLGEEIAASIEGNAKRNEYESDVEAEIVQTQRKDSSVEISSSPKGKGKPIANGNARA
SGSNSSVVETQSSQDQFRTRSAAETVKQGNGSARRGEQVESNSTHSSNTNASENATTYPSI
FSSDGDIRVPRRVNDSEQQGEYSSIPADCRASVWDNNVERLTISGSYDPKEESTQLENEAGLV
SKGDASKAEGNKLLWLCMFTTVAYVIGWSLHFSNPLHP

ATGGCCCAAGCACACCCGGTGCCCAATATTATGTACCACACACATAGACAATCCTGTCAG
AGCATAACTTCCTCATCACCAGTGTTACTCTTGCGCGAACTGGATGGAAAGCATCAGCAA
TTTGTTAATCTCAACTCAATATCTTCTAGTTTTTATGGCAGCTGTGGTAATGCCAAGTTA
ATGAAGCACCATCAGCTTAGAGTGTTGCCAAGTTCTCGAATTTGCCGAGTGCCTGAGTCT
CAGCTGTGTCGAGTAGACCAACCTCGGAAGCCTCAGATTGATAGAAACATATCCTGGAGC
CTTGCTACGGCAGGGTCGCATGTAGTTATGACAGATTTTACGAGATCGAACTTGTATCCT
ACGAGACAGGGGAGGTACAGCGCGGTGGTTCATGCGCAAGCTGTTCATCGCTCTTTCTCC
CTCTTACCTGGAGCTGGTCCGCTTCGAGATTTCCTGTTTGCACTGATTGGAGCAGTGTTT
GCATTCTTTTTTGCGGGATTTTTAGCGAGGCTCTGGAGTTTGGAAGAACGGTACTTTAGA
GCCACGTCAGCTGAGAGAGGGAGTGCAACCATGCCAGAGATGGGCTCAAATCAAGGTCAA
AAAGAGTCTGTGGAGTGGGTGAATATGGTGATTCACAAGGTCTGGAAAGTGTACAGGCGT
AGTCTCCAAGTATGGCTGGTTCAGCTTCTGCAACCAGCTATTGACAATTTGGGGAAGCCC
AACTGGGTGAAGAGGGTGAAAATTGTAGAACTCAACTTGGACTATGAGCCTATTATTGTG
CGTAATGTGCAGCGCCGTGCATCTAGGCGCGCGAATGACTTACAATATCACTTTGGGCTA
CGGTACGCTGGTGGTGCTAGGTGTTTGCTTAACTTGAAACTTGGCCGGGCAGGTTTCGAG
ACCTTCATTCCAGTTGGAGTGTATGAGCTCGATGTGGATGCGGAGCTGTGGGTGAAATTG
CGGTTGGCTCCAGTGAAACCATATGTGGGGACTTTGTCTTTGGCCTTTGTGCGATTGCCT
ACTATCAAGCTGGTCCTGGCTCCCTTCAGAGTTGTGAATCTCTTCTCTATCCCATTTTTA
AACAATTTTCTCTCGAAGTTGTTGACAATAGACCTTCCCCGCCTGCTAGTTCTGCCACGA
CATATTACCTTCGACTTCTTGCCTCAGGGCCAGAATGTCATGGATTCCATGGAGGCAATG
GAAGCATCTGTAGAATCAGGTGCCATGGATGAATCAATTGCATCAGGTGTGCTGGACCTC
CTGAAAACTGCTTCCTCTGAGCCGGCTGTTCCGCAAAAAGACCCGAGTGAGGCCTTTGTT
GGGGAGCTTTCTGTCACTATCTGTGATGCTCGTGGCCTACCAATACGTGGATTTACTGGG
TGGAGTAATCCTTATTGTATCCTCACACTAGGAGATCAAGTTTTAGAAAGCAAACGAAAC
AAGGAAACTTCACAACCTTCTGGTCCCAAAGATCCAGTTTGGAATCAAGATTTCCTGCTA
CTGGTCGAGGATCCTAGGAGACAGAGGCTCATGCTACGTGTTCGCGACAGCACAATGACC
TTGAACCCTAACATAGGTTACTGCTATGTTAATCTTGCAGAGCTCAGAGATTGTGTGCCA
CGTACCATGTGGTTAAACTTGAGGCGAGATGGACTGTTTGGGCCGAAAAAGGTGCCTGGG
AGGGTTCGTCTCACTTTGACATTCAAATCTTACGTTGACAGAGAGGATGACATTGACGAA
AATGCGGGCTCGTTCTCTCCATACATTAAGGTTTTTAAAGATTCGGGCGCTGAAACTGTG
GAAGACATAGGAGTGAGCTTAGGTGAAGAGATAGCTGCGAGTATAGAGGGCAACGCCAAA
AGAAACGAGTACGAAAGTGATGTCGAAGCAGAAATAGTTCAGACACAACGGAAGGACTCG
AGTGTTGAGATTAGTAGCAGTCCGAAGGGCAAAGGAAAACCAATTGCCAATGGGAATGCT
AGAGCTTCAGGATCGAATAGTAGTGTTGTAGAAACGCAGTCAAGTCAGGATCAGTTCAGA
ACAAGAAGTGCTGCAGAAACTGTGAAGCAAGGGAACGGATCTGCACGGAGGGGAGAGCAG
GTGGAGTCGAATTCAACCCACTCTTCCAACACAAACGCCAGTGAGAATGCAACCACTTAC
CCCAGCATCTTCAGTAGCGATGGAGATATTCGCGTGCCTAGGAGGGTGAATGACAGTGAG
CAGCAGGGAGAATACTCCAGCATACCTGCAGATTGCAGAGCCAGTGTGTGGGACAACAAC
GTTGAGAGGTTGACCATTTCTGGGAGTTATGATCCGAAGGAGGAGAGCACCCAACTGGAA
AATGAGGCAGGATTAGTTTCAAAAGGTGATGCGTCCAAAGCCGAGGGTAATAAATTACTC
TGGTTGTGTATGTTTACTACTGTTGCCTACGTGATTGGGTGGAGCTTACATTTCTCAAAC
CCATTGCATCCATGA

Physcomitrella patens Full Length ***NTMC2Type6.1***

Sequence predicted from genome http://www.ncbi.nlm.nih.gov/Traces
MDNGVKKNRWVLPWLEMGRRLWEYLLDWPFMCHIGLVLLVAWIVSFVGLNVALVCALGFL
YLYQ**^ 0**
IEHRQRRRLNWRIRYEEREKANKTR**^ 0**
IAEGETVHWMNQILEKTWPIFLKDITSILLVPLTSMLDQFKPWIA**^ 0**

KKVIVQNLTLGNTPPRITMIRILDTPVDGDDL**^ 0**
AIEASMEWMAAKDMSAVVDVKPLRRLGWGMSATFHLCNLRFEGK**^ 0**
AKVGVKLKAGWPMIERIRICFGTAPLIDMAARPISNSSFDVTELPGISQFT**^ 0**
DRLLADVLTRSLVE**^ 0**
PSMVEIDMEKLMRDVMRPKGPAG**^ +1**
AIYKIRACPRLLHSGISNNTVNNLCMYLYALTSAIIS**^ +1**
GYSDPYVKVGFGNQRGKTKVKWKTLNPTWNETLNFMIPSGQPPNTILLIVRDKDPIFDDKL**^ +2**
GHCEVEISQYRDGKRHDFWLPLEKVKTGRIHLAITVTDNLTASQGSKEASNNNSITVAVHIPS**^ 0**
SAEPQLTQEFHKSVSCDMPLSPGKGPTQPPVASPNAKPEGLSSGISRNLTSSRKIETPPRL
SDIEKSSTS**^ +1**
GAANVRQSMQVVLVLWIMTHTHHISEIKRRVCYEHQTTQEQNARETGFQLRGVRLLWRTDA
IGRKKSPRTCHVHFVTLSPDQGSHTHQLLPHPIQGRLHHTNNPLPVQCP**^ 0**
GVDEAPGVACMHSPSLHVHLAGPQSLHAPVWQLHSCKPSVGAVLPPLANGRHNELVEVGS
RRRPLLILLPCPLPHYPCLEKQRRQK**^ 0**
SAMARPSLEVAPFMNTSFTSEGKVLMMTAAVSVFPCTTRTLKLGCFFTKSKPPPFSDSFCIYK
SSVRNISSSNNFSVREDTLPLLSFFNFLECGFCAPDYLLQKDEHETYLVECSKAQNLHSFLVR
FKNGILDELVYA**^ 0**
HYVVVSVEVRTEQTIRLYSVFISSSYHDYTVGTSVVPTGLACVMCRGCRSSFQTAHKVSGVKLK
NLRMKLSVNLFKPHCICMYFVFLHQGVQTRECVPELLHRVPPNHQDQHVEIPGVPRGTNSEEVG
LEQFEKKWRVKPILHQNTQPINSKRRK

ATGGACAACGGCGTGAAGAAGAACAGATGGGTTCTTCCATGGCTGGAGATGGGGAGGAGG
CTGTGGGAATACTTGCTGGACTGGCCCTTCATGTGCCATATAGGGCTCGTTCTGTTGGTC
GCTTGGATCGTCTCTTTCGTGGGCCTCAATGTCGCCCTCGTTTGCGCTTTGGGGTTCCTT
TACCTCTACCAGATTGAGCACCGACAACGGAGGAGGTTAAATTGGAGGATACGCTACGAA
GAGAGGGAGAAAGCAAATAAAACGAGGATTGCGGAAGGGGAGACTGTGCATTGGATGAAC
CAAATATTGGAAAAAACGTGGCCCATATTTCTGAAAGACATTACTTCAATTCTTTTGGTT
CCCCTCACCTCGATGCTAGACCAATTCAAGCCTTGGATTGCGAAAAAAGTGATTGTGCAA
AACTTGACTCTGGGAAATACCCCACCACGCATAACAATGATCCGCATACTGGACACGCCC
GTTGATGGTGATGACCTGGCTATCGAAGCTTCCATGGAGTGGATGGCAGCAAAAGATATG
TCTGCTGTCGTGGATGTGAAACCTTTACGTCGCTTGGGATGGGGAATGTCGGCTACTTTT
CACCTCTGCAATCTACGTTTTGAAGGCAAGGCGAAGGTAGGGGTGAAACTTAAGGCTGGA
TGGCCTATGATAGAGCGAATACGAATCTGCTTCGGTACAGCACCTCTTATCGATATGGCT
GCTCGTCCTATAAGTAATTCTAGTTTCGATGTGACCGAGCTTCCGGGAATATCACAATTT
ACGGATCGTCTATTGGCAGATGTTTTGACACGGTCCCTGGTAGAGCCAAGCATGGTTGAA
ATTGATATGGAGAAGCTCATGAGAGACGTCATGAGACCGAAAGGACCTGCCGGAGCAATT
TATAAAATTAGAGCGTGTCCAAGGCTACTTCATAGTGGCATCTCAAATAATACTGTTAAC
AATCTCTGCATGTACTTGTATGCACTTACCTCGGCTATAATTTCAGGTTACTCTGACCCT
TATGTAAAAGTCGGGTTTGGAAATCAGCGGGGGAAAACAAAGGTGAAGTGGAAAACATTG
AATCCTACATGGAACGAGACCTTGAATTTCATGATTCCAAGTGGGCAGCCACCGAATACA
ATTCTTCTGATAGTGCGTGATAAGGATCCCATATTTGATGATAAGCTGGGACATTGTGAA
GTAGAGATCAGTCAATACCGCGATGGAAAACGACATGATTTTTGGCTACCACTCGAAAAA
GTGAAGACTGGTAGAATTCATCTGGCAATCACTGTAACCGACAATCTCACTGCCTCACAA
GGTTCCAAAGAGGCATCCAACAACAACTCCATAACCGTTGCGGTACACATTCCCAGCAGT
GCTGAACCGCAATTGACCCAAGAATTCCATAAGTCTGTCTCTTGCGATATGCCATTGTCA
CCTGGAAAAGGTCCTACTCAACCACCCGTAGCGTCGCCAAATGCAAAACCTGAGGGTCTG
AGCTCTGGAATTTCCAGAAACCTCACATCATCTCGGAAAATTGAGACGCCTCCACGTTTA
TCTGATATCGAAAAATCCTCAACTTCTGGCGCAGCAAACGTGCGGCAATCGATGCAAGTG
GTGCTTGTGCTCTGGATCATGACTCACACACACCATATAAGCGAAATAAAGCGGAGGGTG
TGTTACGAACATCAAACTACCCAAGAACAGAATGCAAGAGAGACTGGCTTCCAGCTGAGA
GGGGTACGGCTGCTCTGGCGAACTGACGCCATTGGGAGGAAAAAGTCACCTCGAACTTGT
CATGTACACTTTGTCACCTTGTCACCAGATCAAGGCTCCCACACTCACCAACTCCTTCCT
CACCCCATTCAAGGCCGCCTTCATCATACCAACAACCCATTGCCAGTCCAATGTCCAGGC
GTGGACGAGGCCCCCGGCGTGGCATGCATGCACAGCCCTTCTCTCCATGTTCATCTCGCA
GGTCCTCAATCCCTTCACGCCCCTGTCTGGCAGCTTCATTCCTGCAAGCCTTCCGTAGGT
GCAGTCCTTCCGCCACTCGCCAATGGCAGGCACAACGAACTGGTGGAAGTGGGTTCCCGG
AGGCGCCCATTGCTGATCCTTTTGCCATGTCCACTTCCCCACTATCCATGTCTGGAAAAG
CAACGCCGACAGAAAAGCGCGATGGCGCGGCCCAGTTTGGAGGTCGCTCCGTTCATGAAT
ACTTCCTTCACATCCGAAGGTAAAGTTTTGATGATGACTGCCGCAGTGAGCGTGTTTCCA
TGTACCACGCGCACCCTCAAATTGGGATGTTTCTTCACAAAGAGCAAGCCTCCGCCATTC
AGCGATTCATTCTGCATTTATAAAAGTAGTGTTAGAAACATATCAAGTAGCAATAACTTC
AGTGTAAGAGAGGATACCCTCCCTCTACTGAGTTTCTTCAACTTTTTGGAGTGTGGCTTC
TGTGCCCCGGACTATTTGTTACAAAAGGATGAGCACGAGACTTACCTTGTTGAGTGCAGC
AAGGCTCAGAACCTTCACTCCTTTCTCGTCCGCTTCAAGAATGGAATTCTCGATGAACTT
GTTTATGCGCATTATGTGGTTGTCTCTGTAGAAGTACGCACCGAACAAACCATCAGATTA
TACTCGGTCTTCATTTCAAGCTCATACCATGACTACACAGTAGGTACTTCAGTTGTACCG

ACAGGCCTCGCATGTGTGATGTGCAGAGGTTGCAGAAGTTCGTTCCAAACTGCTCACAAA
GTTAGTGGCGTGAAGTTGAAAAACTTACGAATGAAACTCTCTGTCAATTTGTTCAAACCC
CACTGCATATGCATGTATTTCGTATTTTTGCACCAAGGAGTGCAGACGCGTGAGTGTGTT
CCAGAATTGTTGCACCGCGTACCGCCCAATCATCAGGACCAACATGTAGAAATACCAGGT
GTCCCCCGCGGCACGAACTCCGAGGAAGTAGGACTGGAACAATTTGAAAAGAAATGGCGC
GTAAAACCAATACTGCATCAAAATACACAACCCATCAATTCTAAACGAAGAAAATGA

Transcripts
bu051702
aw145588

Mitsuyasu Hasebe cDNA clones at PHYSCObase http://moss.nibb.ac.jp
42n16
29p22
15k15

Note that of these supporting cdnas and ests, some indicate transcription of an antisense overlapping gene, the WAX2 homolog and none of them indicate correctly spliced sense transcription.

Physcomitrella patens Full Length ***NTMC2Type6.2***

Sequence predicted from genome http://www.ncbi.nlm.nih.gov/Traces
MDESLENAGNPSVWDRGRMVAQYLLGWPFMCHAGSVLLVAWIASYLHLSVGLVCILGFLY
LFQ**^ 0**
IESRQRAKLLWKVHHEEENKARKMR**^ 0**
ISEGETVRWMNKALETIWPMFLGEFSSKHLKIPLSSFLDRFKPWSM**^ 0**
KKISVSDIFLGKSPPIVTMIRMLDDPVDGDHL**^ 0**
AVEASIEWMAAKDMAAVVDVQFLRRISFGIRTTVHICNLCLKGK**^ 0**
VKAGIKFKNGWPVIERLRVCFATAPHVQMTIHPLYNNGVDVSELPGIAQWM**^ 0**
DRLMADIFARSLVE**^ 0**
PNMIEIDVEKLMKDVMIPLDPIPVPR**^ +1**
GAFWTMHVGAPVADVIVEVLEATDLRIGYVN**^ +1**
GYPDPYVKVTVGHQTKTTKVQPKTLHPKWNETLKFSIATLEQLDKILINVRDKDHFYDERL**^ +2**
GSCTVNLNSYRDGIRRDIWCELEDIKTGKIHLAITVVAKQASSLSSEDTSSYNSTTFE**^ 0**
TIDQTISREFDPSLSEGLPLSAAPSPKTHVSTRQKLKALRLREPFLSKTSPTSPVKDPNLNSGRQ
RSVIIDSPESRRRLESPRIASGREIFFIPGDDSSDLFTSDDVSRFASVHSTTKIEAEAINQSSDEPM
AIELHVGVGESGAVKIRRFGDANPPTSPEPQVVGSTRSQSVSKSFNNIEEEGSATRKIRSAGIKL
HSKYHRF**^ 0**
ELQNRRGFSSSNGLTVDTLGGQAANNSPTNTLLGQNDSTKTSLTPEVCGGAMDVRAIGDLEPD
KEHMRNRAKGILKAAGRFGKRLNHRKTSLSSDELSSSGELTPIGGERIEVSLSGPSTDLHMQQQ
VASPSNRLVSSLPASISSTPPQRPPPGLPQPVTPSFDTSE

ATGGACGAGTCTTTGGAGAATGCTGGAAATCCGTCTGTGTGGGATCGTGGACGGATGGTT
GCACAGTATTTGTTGGGGTGGCCATTTATGTGCCATGCAGGATCGGTGCTTCTGGTTGCT
TGGATAGCCTCCTACCTGCACCTTAGCGTTGGTCTTGTTTGCATTCTAGGCTTCCTTTAT
CTCTTTCAGATTGAGAGCCGACAGCGGGCGAAATTATTATGGAAGGTACATCACGAAGAG
GAAAATAAAGCAAGAAAAATGAGGATTTCTGAAGGAGAAACTGTGCGATGGATGAATAAA
GCTTTAGAAACCATTTGGCCCATGTTCTTGGGAGAGTTCTCTTCTAAACACCTTAAGATT
CCTCTCTCCTCGTTCCTGGACCGGTTCAAGCCCTGGAGTATGAAAAAAATATCTGTTTCA
GACATCTTTCTTGGGAAGAGTCCGCCGATTGTAACAATGATCCGCATGTTGGACGATCCT
GTGGATGGTGATCACTTGGCAGTGGAAGCGTCTATCGAGTGGATGGCAGCCAAAGACATG
GCTGCTGTCGTGGATGTGCAATTTCTACGTCGCATTAGCTTCGGAATTCGAACTACTGTG
CACATCTGCAATCTATGTCTTAAGGGCAAGGTGAAAGCAGGGATTAAGTTCAAAAATGGA
TGGCCAGTGATTGAACGACTACGAGTTTGTTTTGCGACGGCACCTCATGTACAGATGACC
ATCCATCCTCTGTATAATAATGGGGTTGATGTATCTGAGCTTCCGGGAATTGCACAATGG
ATGGATCGTCTAATGGCAGATATTTTCGCCCGGTCTTTGGTAGAGCCGAACATGATAGAA
ATTGACGTGGAAAAGCTCATGAAAGATGTAATGATACCCCTAGATCCCATTCCAGTGCCA
AGAGGTGCCTTTTGGACCATGCATGTGGGTGCGCCAGTGGCGGATGTCATTGTGGAAGTT
CTTGAAGCTACTGATCTACGAATAGGGTATGTTAATGGGTACCCCGATCCTTATGTAAAA
GTTACAGTCGGACATCAGACGAAAACAACCAAGGTGCAACCCAAAACATTGCATCCAAAA
TGGAACGAAACCCTCAAGTTTTCTATTGCAACCTTGGAACAACTCGACAAAATACTTATC
AATGTGCGTGATAAAGATCATTTTTATGATGAACGGCTTGGTTCCTGTACAGTGAATTTA
AATAGTTACCGAGATGGAATCCGACGTGATATTTGGTGCGAACTTGAAGACATAAAAACT
GGTAAAATTCACCTAGCCATTACGGTTGTCGCCAAGCAGGCTTCCTCTCTCAGTTCTGAG
GATACCTCGAGCTACAACTCTACAACTTTTGAGACGATTGATCAAACAATCAGCCGAGAA

TTTGATCCATCTCTTTCTGAAGGTTTGCCATTATCTGCAGCACCATCCCCTAAGACTCAT
GTCTCAACACGTCAGAAGTTGAAGGCCTTAAGATTAAGAGAACCTTTCTTAAGCAAAACT
TCACCAACTTCACCAGTGAAGGATCCCAACTTAAATAGTGGACGTCAGAGATCTGTAATT
ATCGATAGTCCCGAGTCCAGGAGGAGGCTTGAGTCGCCTCGGATTGCATCTGGCAGAGAA
ATCTTTTTTATTCCTGGTGATGATTCATCAGATCTGTTTACTTCTGATGATGTTTCCAGA
TTTGCATCAGTACATTCTACAACGAAAATAGAAGCAGAAGCCATAAACCAATCTAGTGAT
GAACCAATGGCAATTGAGCTACATGTAGGCGTTGGGGAATCTGGCGCTGTAAAAATCCGC
AGGTTTGGGGATGCAAATCCCCCAACTAGCCCCGAACCGCAGGTTGTCGGGTCAACACGG
TCTCAGAGTGTGTCTAAAAGTTTTAACAACATTGAGGAAGAAGGAAGTGCTACCCGGAAG
ATTCGAAGCGCTGGAATTAAACTGCACAGCAAGTATCACAGGTTTGAATTGCAAAATCGC
AGGGGGTTTAGTAGCAGTAATGGATTGACCGTAGATACATTAGGTGGGCAGGCCGCAAAT
AATTCTCCTACTAATACACTGTTAGGGCAGAATGACTCAACTAAGACCTCATTGACTCCG
GAAGTGTGTGGTGGAGCAATGGATGTTCGTGCTATTGGGGATCTTGAACCAGATAAAGAA
CACATGCGAAACAGAGCAAAGGGCATACTCAAGGCTGCGGGGCGTTTCGGCAAGAGATTG
AATCATCGGAAAACCTCGCTAAGCTCAGATGAGCTTTCTAGTTCTGGTGAACTGACGCCC
ATAGGAGGCGAAAGGATTGAGGTTTCTCTGTCAGGACCAAGTACTGATCTCCATATGCAA
CAACAGGTAGCTTCTCCTAGCAACCGATTAGTGTCTTCTCTCCCTGCTTCGATTTCCTCG
ACGCCACCCCAGAGACCACCTCCAGGACTGCCACAACCCGTCACTCCAAGTTTCGATACA
AGTGAGTAG

Mitsuyasu Hasebe cDNA clones at PHYSCObase http://moss.nibb.ac.jp
85b08
35m03

Physcomitrella patens Full Length ***NTMC2Type6.3***

Sequence predicted from genome http://www.ncbi.nlm.nih.gov/Traces
MVGTNESGLMEDVDPFPFMLHVLVALVVTYLFLPFSLNGFFFILFILAYLFE**^ 0**
VDRRGRERERRRIASEEWRKINTKR**^ 0**
TFDEGETLRWLNQAIKSMWPVCMETFASKHLFSHMLPWFLKAYKPRAV**^ 0**
SDVKIETLHLGSTPPVFNLIRTLERPTGGDHV**^ 0**
VFESNMDFNSGDDMNCKMSVLLKSLGLRTTFYISRLHVEGT**^ 0**
VRISVKFLESWPVVGRLRLCFTSKPLVSMSYRPMDKNGIDVSLIPVIANVV**^ 0**
EKMVDNALELSVVE**^ 0**
PNLLIVDIEKLVSHMFSDSRAQQ**^ +1**
GLSRFFKVENEATLVVEILEASNLKAADSN**^ +1**
GLSDPFVDINFGQRHQRTSKKKKTLNPKWENERYEFPIVSWDMPNLLTLRVRDWDILPT
ISSHEL**^ +2**
GICSVSVNDYRNGERHVFNKRLEKVLKGHLKFAISVVYSHPPQMAEEPRISDAMCE**^ 0**
TVSQDLKTSSSAVDYISPTRVPGMPRSKSDTNLPRIATSKVPVSRHRRRNSSGDSALSEVFG
EQKPHGDKIDFIDMPYGPRGAFTIFHPGLQTPLLFTPARDASKASETDKSGEFKQSRRYRWIR
RMRKQNGGNNSLEGLQTDGMSEQSCFSGSSADFSTDLRDFNPDQYRGPVKMNLEDVEPPL
MDMAPSIVPIHVSGQLEPIQSGKTPTTPAINQFFGNAPRDTFAPAPGEVDSRPSPVSGGSNME
TTSILSNEEGHKGEGIGDAAFASSQPVQRLRTCPKRQSGKPSFSWPSTWYKKMRLLAKRSKK
KKRNDKGEVAVSSELKYPVGEYAEGDHRAVSFYHEDSDQSFPSGPQTFIFESPLETRHMANA
GNPGNAQVDSPQQRLIINSSSSSAANSGSQSDRHWASKVKRLIPDKLARTSQSFRHEFLDLKR
LQTPIRDSSLTTIPERSSHHRNAKPHV

ATGGTGGGGACGAATGAATCAGGGTTGATGGAGGATGTGGACCCGTTTCCGTTCATGCTT
CACGTGCTTGTGGCGCTGGTCGTCACTTATCTGTTCTTGCCCTTCAGCCTAAACGGGTTT
TTCTTTATACTCTTCATACTCGCCTATCTCTTTGAGGTAGATAGGCGTGGTCGAGAGAGA
GAGCGACGTCGGATAGCTTCTGAGGAGTGGAGAAAGATCAATACAAAACGGACGTTCGAT
GAGGGAGAAACTCTAAGGTGGCTCAACCAAGCCATCAAGAGCATGTGGCCAGTGTGCATG
GAAACGTTTGCATCGAAGCATTTATTTAGTCATATGCTGCCTTGGTTTTTAAAAGCTTAT
AAGCCTAGGGCTGTAAGTGATGTGAAGATAGAAACCCTGCACCTTGGTAGCACACCACCA
GTATTCAACCTCATTCGCACGCTTGAGCGTCCCACAGGTGGTGATCATGTGGTGTTTGAG
TCAAACATGGATTTCAATTCCGGCGATGACATGAACTGCAAGATGTCTGTACTACTGAAA
AGCCTTGGTCTTCGGACTACTTTCTACATCTCGCGACTGCATGTCGAAGGCACGGTCAGA
ATATCGGTGAAGTTTCTAGAAAGTTGGCCAGTGGTAGGGCGATTACGCTTATGTTTTACC
AGCAAACCTCTAGTATCAATGTCATATCGTCCGATGGACAAGAACGGAATAGATGTGAGC
CTTATTCCCGTTATCGCTAATGTGGTGGAAAAAATGGTGGATAATGCATTGGAGCTCTCT
GTTGTTGAGCCGAACTTGTTGATTGTAGACATTGAGAAACTAGTAAGCCACATGTTTTCT
GATTCAAGAGCCCAACAAGGTTTGTCGAGGTTTTTCAAGGTGGAGAACGAGGCCACTCTT
GTCGTAGAAATCCTCGAGGCCTCGAACTTGAAGGCTGCTGACAGTAACGGACTCTCTGAC
CCTTTCGTGGACATAAATTTTGGGCAACGCCATCAGAGAACTTCAAAAAAGAAGAAGACA

TTAAACCCGAAGTGGGAAAATGAACGTTATGAGTTCCCTATTGTAAGCTGGGATATGCCA
AATCTGCTGACTCTGCGAGTTCGAGACTGGGACATATTGCCCACTATTAGTTCCCACGAG
CTTGGAATCTGTTCTGTCTCTGTGAATGACTACCGAAACGGGGAGCGTCATGTGTTCAAT
AAACGTCTTGAGAAAGTCTTGAAGGGCCACTTAAAATTCGCTATCAGTGTGGTTTACAGT
CATCCTCCTCAAATGGCAGAGGAACCACGTATAAGTGATGCCATGTGTGAGACTGTCTCA
CAAGACCTTAAAACTAGTAGCTCAGCTGTTGATTACATATCACCAACCAGGGTACCTGGA
ATGCCGAGGTCGAAGAGTGACACAAACTTACCAAGGATTGCGACTTCGAAAGTCCCAGTG
AGTAGGCACCGTCGTCGCAACTCGTCTGGAGATTCTGCGCTGTCGGAAGTCTTTGGAGAG
CAGAAGCCCCACGGTGACAAAATTGACTTTATTGATATGCCGTATGGTCCTCGTGGTGCT
TTCACAATATTCCATCCTGGATTGCAGACACCTCTTCTTTTTACTCCCGCAAGGGACGCC
TCAAAGGCATCTGAAACTGACAAAAGCGGAGAGTTCAAGCAATCTCGAAGATATCGCTGG
ATTCGAAGGATGAGAAAGCAAAACGGTGGAAATAATTCCCTGGAGGGTCTCCAGACTGAT
GGCATGAGTGAACAGTCATGTTTCTCTGGAAGTAGTGCAGATTTTTCGACAGATCTCAGG
GATTTCAATCCAGATCAGTACCGAGGGCCGGTGAAGATGAACTTAGAAGACGTTGAGCCC
CCATTGATGGACATGGCTCCATCTATAGTCCCCATCCACGTTTCTGGACAACTAGAACCG
ATTCAAAGCGGGAAGACGCCGACCACGCCAGCCATTAACCAGTTTTTCGGGAATGCGCCT
CGTGACACATTCGCCCCTGCACCTGGGGAGGTAGATTCTAGGCCCTCTCCTGTGTCTGGC
GGTTCTAATATGGAAACTACTTCTATTTTATCGAATGAGGAGGGACACAAGGGTGAGGGA
ATTGGAGATGCTGCTTTTGCCTCTTCACAGCCAGTGCAACGACTGAGAACCTGTCCGAAG
CGACAGTCAGGAAAGCCATCGTTCTCCTGGCCGTCGACTTGGTACAAGAAAATGCGGTTG
CTGGCAAAGCGCTCTAAGAAGAAGAAGCGGAACGACAAAGGTGAAGTCGCCGTGTCCTCA
GAGTTGAAATACCCTGTTGGCGAGTACGCAGAAGGCGATCATCGAGCCGTGTCATTCTAT
CACGAAGATTCGGACCAGAGTTTTCCCTCTGGTCCACAAACCTTTATTTTTGAATCTCCA
CTTGAGACTAGACACATGGCCAATGCGGGCAACCCCGGGAATGCGCAAGTCGACTCCCCA
CAGCAGCGTCTGATCATCAACAGCTCCAGCAGCAGTGCTGCAAATTCAGGCAGCCAGTCA
GACAGACACTGGGCCTCCAAGGTGAAGAGGCTAATCCCTGACAAGTTGGCTCGCACGAGC
CAGTCGTTCCGCCACGAGTTCTTGGACCTCAAGCGGTTGCAGACGCCCATTCGCGATTCA
TCGCTGACCACCATCCCGGAGAGATCTTCTCATCATCGCAACGCCAAACCACATGTTTGA

Physcomitrella patens Full Length ***NTMC2Type6.4***

Sequence predicted from genome http://www.ncbi.nlm.nih.gov/Traces
MGNPAQGEPSFIAEKTMDQFLLMFYVVSVMCCLWIVSSVSSNCLFLSLLGGYLFW**^ 0**
ILKQCLEREKREILFEERKRVNASK**^ 0**
AMTEGETLQWLNESLNVMWPICMEKFASQHFFTPIAPWFLKKFKPKYV**^ 0**
KEVTLQSLHLGSTSPLFSLIRVLPASQDDDV**^ 0**
IFEAEMEFSSDKDMKAQMSVQMKHINTTTTFYISKLYIKGT**^ 0**
VKFSVKFEKGWPILGRVRFCFANAPYIDMTARPYAKKGIDMRIIPGAASWL**^ 0**
EETLGTALEQSVVE**^ 0**
PYMLVIDMKKLVSNMMFPGPISRY**^ +1**
GLQDFFSVEHKSGVMVLVEVLEAGELKAGNAA**^ +1**
GLPDPMVELLLGTRREITKPKLQTVNPVWTREMHRMPIVNWEYPNILTLRVMSKPSWG
RSVDL**^ +2**
GICSIAVKEFQNGERKEKKLRLESVNKKEYMGWIKFAITVEHHNGSQ**^ 0**
TPEERHTTFGSQPQTQEIASITPPESTATSRSQALGTRIKAFMHMRNSSTEVETAILVRAAT
SEPSEIDGGSTDIIEMPSNIPPGSFSIQCPEKEETSYFTSDPRRRSNAKKHKKDKSAPGCK
ERDAIMSNTKVKTPYMRFLRMRKPRNISKISFIEKSPADREATMTDLSGYSSSMEHSSEVK
VCTDSSKTNMQLFPDMAHAVHNIIHEKTIIDSCNATTQLQGNSQQQTIGRDFVENLSFSNL
QDEVTLRHGCRSQSATAPMPPKQYTKNNSWLRNFKLTKWPKKSRKTMVSKQEHVSVDN
LERDPASLPRFEHEFGDPSYTSTTLSIRKELPPTDLKTALLQAQSPLTGSLLENHRSILASG
RPVSGSQSCRQDASILKRILPHRAQSFRFDLLNITNRKHARLE

ATGGGAAACCCGGCACAGGGGGAGCCTTCTTTCATTGCGGAGAAGACGATGGATCAGTTT
CTTCTCATGTTCTATGTAGTGTCAGTGATGTGCTGTTTATGGATCGTTTCCTCCGTGAGT
TCGAACTGCCTCTTTTTGAGCCTCCTCGGCGGCTACCTGTTCTGGATTCTGAAACAATGT
TTGGAGAGGGAGAAGCGGGAAATTCTGTTCGAAGAGAGGAAAAGGGTGAACGCAAGTAAG
GCAATGACGGAAGGCGAGACGCTGCAGTGGTTGAATGAAAGCTTGAATGTCATGTGGCCG
ATATGTATGGAGAAGTTTGCTTCACAGCACTTCTTCACACCCATTGCGCCGTGGTTTTTG
AAAAAATTCAAGCCCAAGTATGTGAAGGAAGTGACGCTGCAATCTCTTCATTTAGGAAGC
ACCTCACCCCTTTTCAGCCTCATACGCGTCTTGCCAGCTTCCCAAGATGATGATGTGATT
TTTGAGGCAGAAATGGAGTTTTCGTCTGACAAGGACATGAAAGCACAAATGTCAGTTCAA
ATGAAACACATAAATACCACCACCACTTTTTATATCTCAAAACTTTACATCAAAGGCACG
GTCAAGTTTTCGGTGAAGTTCGAAAAAGGTTGGCCTATTTTAGGTCGAGTACGCTTCTGC
TTTGCTAATGCTCCCTATATCGACATGACCGCTCGGCCATACGCCAAGAAGGGTATCGAC
ATGAGGATCATTCCAGGAGCTGCAAGCTGGCTGGAGGAAACACTGGGCACAGCACTGGAG

CAATCTGTCGTTGAGCCATACATGTTGGTCATCGACATGAAAAAGCTCGTCAGCAACATG
ATGTTTCCTGGGCCGATATCACGATATGGCCTGCAAGATTTTTTTAGTGTGGAGCACAAA
TCGGGTGTCATGGTGTTAGTTGAAGTTTTGGAGGCCGGCGAGCTGAAGGCTGGAAATGCT
GCTGGTTTGCCTGATCCAATGGTTGAGTTATTACTGGGGACGCGAAGAGAAATAACCAAA
CCAAAGTTGCAGACGGTCAATCCAGTGTGGACACGTGAGATGCATCGTATGCCCATAGTG
AACTGGGAGTACCCAAATATTCTCACCTTGCGCGTGATGTCCAAGCCTAGCTGGGGACGC
TCAGTTGACCTTGGGATATGTAGTATTGCAGTGAAAGAATTTCAGAACGGTGAACGGAAA
GAAAAGAAATTAAGACTTGAAAGTGTGAATAAAAAGGAGTATATGGGTTGGATAAAATTT
GCAATAACTGTGGAACATCACAACGGTTCTCAAACTCCTGAGGAGCGACACACGACTTTT
GGATCTCAGCCTCAAACTCAGGAAATTGCAAGCATTACGCCACCTGAGTCTACCGCCACA
AGTAGAAGCCAAGCACTTGGAACCAGAATTAAAGCTTTCATGCATATGCGAAACAGCTCC
ACTGAGGTTGAAACTGCAATCCTCGTGCGGGCTGCAACCTCCGAACCAAGTGAGATTGAT
GGTGGCAGCACTGATATCATAGAAATGCCCTCTAACATTCCTCCCGGCTCCTTCTCAATT
CAATGTCCTGAAAAAGAGGAAACCTCGTATTTTACTTCTGATCCTAGGAGAAGATCTAAT
GCAAAGAAGCACAAGAAGGACAAGAGCGCACCGGGTTGCAAGGAGAGGGACGCAATAATG
TCCAACACCAAAGTTAAGACGCCATACATGAGATTCTTAAGGATGAGGAAACCACGTAAC
ATCAGCAAGATCTCTTTCATCGAGAAGTCCCCTGCTGACAGAGAGGCTACCATGACTGAT
CTCTCTGGGTACTCCAGCAGCATGGAGCATTCCTCGGAGGTGAAGGTGTGCACTGACTCT
TCAAAGACAAATATGCAGCTGTTCCCTGACATGGCACACGCTGTGCACAACATCATTCAT
GAGAAGACCATCATCGACAGTTGTAACGCAACCACACAACTTCAGGGTAACAGCCAACAA
CAAACAATCGGGAGGGATTTTGTAGAAAACCTCAGTTTCTCGAATTTGCAGGACGAGGTG
ACACTCAGACATGGGTGCAGGTCCCAGAGTGCGACAGCACCAATGCCTCCGAAGCAGTAC
ACCAAGAATAATTCATGGTTGCGCAATTTCAAACTCACCAAGTGGCCTAAGAAATCAAGG
AAAACCATGGTTAGCAAGCAGGAACATGTTTCAGTCGACAACTTAGAACGGGACCCGGCT
TCATTGCCACGGTTTGAACACGAGTTTGGGGATCCGAGTTACACTTCCACCACCCTGTCG
ATTAGGAAAGAGCTTCCACCGACAGATCTGAAAACGGCTCTCCTTCAAGCGCAGTCCCCT
TTGACAGGATCCTTGCTTGAAAATCACCGCAGCATCCTTGCCTCCGGTCGACCTGTCAGC
GGCAGCCAATCTTGCAGACAAGATGCCAGTATTCTTAAGAGGATTCTTCCCCACAGAGCA
CAGTCGTTCCGATTCGACTTGCTCAACATCACGAACAGAAAACATGCTCGTCTCGAATGA

Transcripts
bj583119
bj591903
bj592949

Mitsuyasu Hasebe cDNA clones at PHYSCObase http://moss.nibb.ac.jp
21l01
4b12
7f08

Physcomitrella patens Full Length ***NTMC2Type6.5***

Sequence predicted from genome http://www.ncbi.nlm.nih.gov/Traces
MEGLVEEEASPSAGKVIDQLFPMPQLVPVLFIVLLMFILGINWLIAILLICYVSW**^ 0**
VQKQYFESERRRILFEERKRSNTKR**^ 0**
VMSEGETLRWVNESLKVMWPVCMQKFASQNFFTPMAPWFLDRYKPKYV**^ 0**
TKVTMNSLHLGNTPPFFRLIHVLQSSQDKEE**^ 0**
VFEAQMEFLSDEVMGARLIVEISGVKITFYISKLYIKGT**^ 0**
VKFSLNFVGGWPVLGRIQFCFANAPTVNMDARGYGLYMGYIPGAKSWL**^ 0**
EETLGRALEESVVE**^ 0**
PYMLVIDMEKLVSNMMFSEPKPSQ**^ +1**
GLQDFFSVEHRSDFTVLVEILEAGDLKAGRAN**^ +1**
GLPDPIVELSLRSHIQKTKSRPKTINPVWTDEKHRFPIENWEYSNILILRVIHKSWRGQVEL**^ +2**
GICSTPVKEFQGGERKERKLPLERDKETVGWIKFAVTVEHRGGSESHEEPRTTFEFQ**^ 0**
TQTQDIASITPPDSAATTRSQALGVKANPLTHTGNSSTEVETGSLAHVTSEPSEPDGGSTE
IIKIHNIPDGAVLSDSKVKQSMKRLLRKARNISKVSSHDKSPPDREMIQGEHSGYSSSLDCS
SELKVYHGSLKVNLQLSPDMASLEPRETELPCEPLNLDSSTKLETVHSVQDPDNFLLKTPD
LAHTRRNNFHEKINEGCTGAPQSEIKGPQLSLVGGAVGKSSISNELNEATLSSTPILQSHMP
EKQNSKNRTWLTKFNLPKRRKNPRKSKGGKVDYVSAYSFERDPGSLPLSETDTSATQSFR
EEGSKKDFKTLSRQGQPIATDGSPENHRSILASSGGASGSQSCRVEPSNAKKITGTQSMS
MRFDSLNTIHERHDRLDRTA

ATGGAAGGGCTAGTTGAAGAGGAGGCTTCCCCCTCCGCGGGGAAGGTGATAGATCAGTTG
TTTCCCATGCCCCAGCTGGTGCCAGTACTCTTCATTGTGTTGCTGATGTTCATCTTGGGC
ATCAACTGGCTCATAGCGATTCTCCTCATCTGCTACGTTTCTTGGGTTCAGAAGCAGTAT
TTTGAGAGTGAGAGGCGACGGATACTGTTCGAAGAAAGGAAGAGAAGCAACACAAAAAGG

GTAATGAGCGAGGGCGAGACTCTACGTTGGGTGAACGAAAGCTTGAAGGTCATGTGGCCA
GTATGTATGCAAAAATTTGCGTCACAGAACTTCTTCACACCCATGGCGCCGTGGTTTTTG
GACAGATACAAGCCCAAATATGTGACGAAAGTGACGATGAACTCTCTTCATTTAGGAAAC
ACGCCACCATTTTTCAGACTTATTCATGTACTGCAATCTTCCCAAGACAAAGAAGAAGTT
TTTGAGGCGCAAATGGAGTTTTTGTCTGACGAGGTTATGGGAGCTCGGTTGATTGTTGAA
ATAAGTGGCGTCAAAATCACCTTTTACATCTCAAAACTGTACATTAAGGGGACGGTCAAG
TTTTCGTTGAACTTTGTGGGAGGTTGGCCAGTTCTAGGTCGAATACAGTTCTGTTTTGCA
AACGCTCCCACTGTGAATATGGACGCACGGGGATACGGTCTCTACATGGGTTACATTCCA
GGAGCTAAAAGCTGGCTGGAGGAAACATTAGGCAGAGCTCTTGAGGAGTCAGTCGTTGAG
CCATACATGTTAGTCATCGATATGGAAAAGCTTGTGAGCAACATGATGTTTTCTGAACCT
AAACCATCGCAGGGCTTGCAAGACTTTTTTAGTGTGGAGCACAGGTCAGATTTTACAGTG
CTCGTTGAGATTTTGGAGGCTGGTGATCTGAAGGCTGGAAGAGCCAATGGTTTGCCTGAT
CCAATTGTTGAGTTATCACTGAGGTCTCATATACAGAAAACCAAATCGAGACCGAAGACG
ATCAACCCAGTATGGACCGATGAGAAACATCGTTTTCCCATAGAGAACTGGGAGTATTCA
AACATCCTTATCTTACGGGTGATACACAAAAGCTGGAGAGGTCAAGTTGAGCTTGGGATA
TGTAGCACTCCAGTGAAAGAATTCCAGGGCGGTGAACGCAAAGAAAGGAAATTACCGCTC
GAAAGAGATAAGGAGACTGTGGGTTGGATAAAGTTTGCAGTCACCGTGGAACATCGTGGC
GGTTCTGAGAGTCATGAGGAACCACGTACAACTTTTGAATTTCAGACTCAAACTCAGGAC
ATAGCAAGCATCACACCACCTGATTCTGCCGCCACCACGAGAAGTCAAGCACTTGGGGTC
AAGGCCAATCCACTAACACATACAGGAAATAGTTCCACCGAGGTTGAAACTGGAAGTCTC
GCACATGTTACCTCCGAACCAAGTGAGCCTGATGGTGGCAGCACTGAAATTATTAAAATT
CATAACATTCCTGATGGCGCCGTCTTGTCGGATTCTAAAGTGAAGCAATCGATGAAGCGC
CTTCTGAGAAAGGCGCGTAACATCAGCAAGGTTTCTTCCCACGACAAGTCTCCACCGGAC
AGAGAAATGATACAGGGTGAGCATTCTGGATACTCCAGCAGCTTAGACTGTTCCTCAGAG
CTAAAGGTGTATCATGGCTCTTTGAAAGTGAACTTGCAGCTGTCTCCTGACATGGCATCT
CTAGAGCCTCGTGAGACTGAGCTTCCTTGTGAGCCTCTGAATCTGGATTCAAGCACCAAG
CTGGAAACCGTCCATAGCGTCCAGGATCCAGATAATTTTTTGCTGAAGACACCTGACCTG
GCTCATACGAGACGCAACAATTTTCATGAGAAGATCAATGAGGGCTGCACTGGAGCCCCA
CAATCCGAGATCAAAGGCCCGCAATTATCCCTCGTGGGGGGAGCGGTAGGGAAGTCGAGT
ATCTCGAACGAACTGAACGAGGCAACACTTAGTAGCACCCCGATATTGCAATCTCATATG
CCTGAGAAGCAGAACTCCAAGAATAGAACATGGTTGACCAAGTTTAATCTCCCCAAACGG
CGGAAGAACCCAAGGAAAAGTAAGGGTGGCAAGGTGGATTACGTCTCAGCGTACAGCTTC
GAACGGGACCCGGGTTCTTTACCATTATCTGAAACTGACACTTCCGCCACTCAATCCTTT
AGGGAAGAAGGTTCAAAGAAAGATTTCAAGACGTTGTCTCGCCAAGGGCAACCAATTGCC
ACCGATGGGTCACCTGAAAATCATCGCAGCATCCTTGCCTCCAGTGGGGGTGCCAGTGGC
AGCCAGTCCTGCAGAGTAGAGCCCAGCAATGCCAAGAAGATTACTGGAACACAGTCAATG
TCAATGCGGTTTGACTCCTTGAACACCATTCACGAGAGGCACGACCGTCTGGACCGCACA
GCATGA

Transcript
bj157784

Mitsuyasu Hasebe cDNA clones at PHYSCObase http://moss.nibb.ac.jp
5eo7

Physcomitrella patens fragment ***NTMC2Type6***

Sequence predicted from genome http://www.ncbi.nlm.nih.gov/Traces
MVNSAETEKKSEPANAMGAARFLLFLVLVVVTTWLFLPVSLLGFICLCSFIVLVIK**^ 0**
EEVRYRAEERSRMDDAERSRVNKKR**^ 0**
TCEEGESLRWLNEGMKRIWPMSTEFASTYFFGPMASTFLDKYKPRGSGLV**^ 0**
EATIDVSTLCLGNTPPEFYCIQTLEQSMDGYDV**^ 0**
AYEARMVFHADERMTAQMKVVLNFLGTYNIYISGLRIEGT**^ 0**
VRISAKFLKGWPFVKQVRFCFKTKPETVDVKYGFKFLRDVRNGYVKQRVVSSQVT
YPVCVFRFLCKHSH**^ 0**

ATGGTGAACTCAGCGGAGACCGAAAAGAAGTCTGAGCCAGCAAATGCCATGGGCGCGGCT
CGTTTCTTATTGTTCCTGGTGCTGGTGGTCGTCACCACGTGGCTGTTCTTGCCTGTCAGC
CTATTAGGATTCATCTGTTTGTGCAGCTTCATCGTCCTCGTCATCAAGGAAGAAGTACGA
TACCGGGCGGAAGAACGAAGTCGAATGGATGACGCAGAGCGGAGCAGGGTCAATAAAAAA
CGGACATGTGAGGAGGGAGAGTCATTAAGATGGCTAAACGAGGGCATGAAGAGAATATGG
CCGATGAGCACGGAGTTTGCATCAACTTATTTTTTTGGTCCAATGGCTTCGACGTTTCTA
GATAAATACAAGCCTCGGGGGTCGGGGTTGGTGGAAGCGACGATAGATGTCAGCACTTTG
TGCCTTGGAAACACGCCACCAGAGTTCTATTGCATTCAGACGCTTGAGCAATCTATGGAT

GGTTATGATGTGGCATACGAGGCAAGGATGGTATTCCATGCCGATGAGCGCATGACTGCT
CAGATGAAGGTAGTTCTGAATTTTTTAGGGACGTACAATATCTACATCTCAGGTCTGCGA
ATTGAGGGAACGGTCAGGATATCAGCGAAGTTTCTGAAAGGTTGGCCATTTGTCAAGCAA
GTGCGCTTCTGCTTCAAAACTAAGCCTGAAACTGTAGACGTGAAATATGGGTTTAAGTTC
CTTCGTGACGTGAGGAATGGCTATGTAAAGCAGCGAGTGGTAAGTTCACAGGTTACGTAT
CCTGTCTGCGTGTTTCGATTCTTGTGCAAACATTCACAC

Physcomitrella patens fragment ***NTMC2Type6***

Sequence predicted from genome http://www.ncbi.nlm.nih.gov/Traces
HLVVSRVAGNIHLTLEHHNNSQNPEERYTTFGSQ**^ 0**
PQTQKIASITPPVSIATSRNQALGTRIKAFRHKRNSSIEVETVTLIRAATSEPSEIDGGSTDIIE
MPSNIPSGSFSIQCPGKEEPSYFIPDPRRRSNAKKYKDSSTPFCKEREAIMSNSKVKTPYM
RFLRMRKPCNISKIYFIEKSRADREATMTDHSGYPSSMEHFSEVKVCTDSLKTNMQLFPDM
AYVVHNIVHEKTIIDSCNATTQLQDNSQKQIVGKDFAENLSFSKLQDEVTLRHGCRSQSATA
PMPPKxYSKNNSWLGNFKLTKWPKKSRKTMVSEQDHVSVDNLEHDPASLPRFEQEFVDP
SDTSTTLSIRKERPQIDLKTALLQExSPLTGSLLENHRSILASGRPVSGSQSCRQDASILMNV
NKSDVAECLFCILIMSWSLLNEGVRC

CATCTAGTGGTTTCTCGAGTTGCAGGTAATATTCACTTAACTTTGGAACATCACAACAAT
TCTCAAAATCCTGAGGAGCGATACACAACTTTTGGATCTCAGCCTCAAACTCAGAAAATT
GCAAGCATCACGCCACCTGTTTCTATTGCCACAAGTAGAAACCAAGCCCTTGGAACCAGA
ATTAAAGCTTTCAGGCATAAGCGAAACAGCTCCATTGAGGTTGAAACTGTAACCCTCATT
CGGGCTGCAACCTCCGAACCAAGTGAGATTGATGGTGGCAGCACTGATATCATAGAAATG
CCCTCTAACATTCCTTCTGGCTCCTTCTCAATTCAATGTCCTGGAAAAGAGGAACCCTCG
TATTTTATCCCTGATCCTAGGAGAAGATCTAATGCAAAGAAATACAAGGACAGTAGCACA
CCGTTTTGCAAGGAGAGGGAGGCAATAATGTCCAACTCCAAAGTTAAGACGCCATACATG
AGATTCTTAAGGATGAGGAAACCATGTAACATCAGCAAGATCTATTTCATTGAGAAGTCC
CGTGCTGACAGAGAGGCTACCATGACTGATCACTCCGGGTACCCCAGCAGCATGGAGCAT
TTCTCGGAGGTGAAGGTGTGCACTGACTCTTTAAAGACAAATATGCAGCTGTTCCCTGAC
ATGGCATACGTTGTGCACAACATCGTTCATGAGAAGACCATTATTGACAGTTGTAACGCA
ACCACTCAACTTCAGGATAACAGCCAAAAACAGATAGTCGGAAAGGATTTTGCAGAAAAC
CTCAGTTTCTCAAAATTGCAAGACGAGGTGACACTCAGACATGGGTGCAGGTCCCAGAGT
GCGACAGCACCAATGCCTCCGAAGTAGTACTCTAAGAATAATTCATGGTTGGGCAATTTC
AAACTCACCAAGTGGCCTAAGAAATCAAGAAAAACCATGGTTAGCGAGCAGGATCATGTT
TCAGTCGACAACTTAGAACACGACCCAGCTTCATTGCCTCGGTTTGAACAAGAGTTTGTG
GATCCAAGTGACACTTCCACCACCCTGTCGATTAGGAAAGAGCGCCCACAGATAGATCTG
AAAACGGCTCTCCTCCAAGAGTAATCTCCTTTGACAGGATCCTTGCTTGAAAATCACCGC
AGCATCCTTGCCTCCGGTCGTCCTGTCAGCGGTAGCCAATCTTGCAGACAAGATGCCAGT
ATTCTCATGAATGTCAACAAGTCCGATGTGGCTGAATGTCTGTTCTGCATTTTGATCATG
AGCTGGTCTTTGCTGAATGAAGGAGTTCGCTGTTGA

Tortula ruralis fragment ***NTMC2Type1***
cn202307
cn205934
cn208420
cn203070

Tortula ruralis fragment ***NTMC2Type1***
cn202897

Tortula ruralis fragment ***NTMC2Type2***
cn202513
cn208634
cn202962
cn207402

Selaginella lepidophylla fragment ***NTMC2Type2***
bm402966

Selaginella lepidophylla fragment ***NTMC2Type3***
bm402352

Adiantum capillus-veneris fragment ***NTMC2Type3***

bp915757

Adiantum capillus-veneris fragment ***NTMC2Type5***
bp915736

Ceratopteris richardii fragment ***NTMC2Type1***
Gene 99 Craxton, M. BMC Genomics. 2004 Jul 6;5(1):43
be642373
be642184

Ceratopteris richardii fragment ***NTMC2Type3***
Gene 107 Craxton, M. BMC Genomics. 2004 Jul 6;5(1):43
be643481

Cycas rumphii fragment ***NTMC2Type1***
dr062506

Ginkgo biloba fragment ***NTMC2Type2***
dr065108

Ginkgo biloba fragment ***NTMC2Type4***
dr073299

Ginkgo biloba fragment ***NTMC2Type6***
dr073481
dr063834

Welwitschia mirabilis fragment ***NTMC2Type1***
ck746596

Welwitschia mirabilis fragment ***NTMC2Type1***
dt592585

Welwitschia mirabilis fragment ***NTMC2Type3***
ck762431

Welwitschia mirabilis fragment ***NTMC2Type4***
dt593502
ck757678
ck766066
ck765293

Cryptomeria japonica fragment ***NTMC2Type2***
au298802

Cryptomeria japonica fragment ***NTMC2Type4***
au299542

Picea glauca Full Length ***NTMC2Type1***

MGFVSTVVGFFGFGVGVTIGVLIGYFLFIYFQPTDVKDPIIRPLGELDSKTLEGLLPEIP
LWVKNPDYDRVDWLNTFIHEMWPYLDKAICQIIRDKTKPMIEQYVGKFKIESIEIETLTL
GTLPPTLQGMKVYDTQEKELIMEPVLKWAGNPNVIVAVKAFGLRATVQLVDLQVFAIPRV
TLKPLVPSFPCFAKIFVSLMEKPHVDFGLKLLGGDLMAIPGLYQFAQEMIKEQVANLYLW
PKTLEVPILDQRATHKPVGMLHVKVVRAINLKKKDMMGKSDPYVKLKMMGEKLPSKKTTV
KSSNLNPEWNEEFKFVVKDPESQALELSVYDWEKVGSHEKMGIQAYDLKELTPSETKSVT
LNLLKSLDPNDPQNAKARGQITIEMTYNPFKEDENSPADDEDSAVEKAPEGTPAGGGLLV
VRVLEAEDVEGKHHTNPYVRLLFKGEEKKTKPVKKNRDPRWDQEFEFMLEDPPVNDKIHV
EVMSKGSSLALHSRESLGYVDINLSDVVNNKRINEKYHLIDSKNGKLQLELLWRSS

ATGGGTTTTGTGAGTACTGTAGTGGGTTTCTTCGGCTTTGGAGTGGGCGTTACAATTGGTGTTCTTA
TTGGGTATTTCCTTTTCATCTACTTCCAGCCCACTGATGTGAAGGACCCTATCATCAGGCCTCTTGG

AGAATTAGATTCAAAGACTTTGGAAGGACTCTTACCTGAAATTCCATTATGGGTGAAAAATCCTGAT
TATGATAGAGTTGATTGGCTCAACACGTTTATTCATGAGATGTGGCCTTATCTTGATAAGGCAATCT
GTCAGATAATAAGAGACAAAACAAAGCCTATGATAGAACAATATGTTGGGAAGTTCAAAATAGAATC
AATTGAAATTGAAACACTTACATTGGGCACACTTCCTCCTACACTGCAAGGTATGAAAGTCTATGAT
ACTCAAGAAAAGGAATTGATCATGGAACCTGTTCTCAAATGGGCTGGAAACCCAAATGTGATCGTGG
CAGTGAAAGCATTCGGACTAAGGGCGACTGTGCAGCTGGTGGATTTGCAGGTATTTGCAATTCCACG
TGTCACACTGAAGCCTCTTGTCCCGAGTTTCCCATGCTTTGCAAAGATATTTGTCTCTCTTATGGAG
AAGCCCCATGTAGACTTCGGACTAAAATTACTTGGTGGTGATCTTATGGCTATCCCTGGTCTTTACC
AATTTGCCCAGGAAATGATTAAAGAGCAGGTTGCAAACTTATACTTGTGGCCAAAAACACTGGAGGT
ACCAATTTTGGACCAGAGAGCTACACATAAACCTGTTGGCATGCTCCACGTAAAGGTCGTTAGGGCA
ATTAACTTGAAAAAGAAAGATATGATGGGTAAATCTGACCCATATGTAAAGCTCAAGATGATGGGAG
AAAAACTTCCGTCAAAGAAAACCACAGTCAAATCAAGTAATCTGAACCCAGAATGGAATGAGGAGTT
CAAGTTTGTTGTTAAAGATCCAGAATCTCAAGCTCTTGAACTTTCTGTCTATGACTGGGAAAAGGTG
GGTTCACATGAAAAGATGGGTATACAAGCGTATGATTTAAAAGAGCTTACACCGTCTGAGACCAAAA
GTGTTACCCTCAATCTCCTTAAAAGTTTGGATCCAAATGATCCTCAAAATGCCAAAGCACGTGGACA
AATCACGATAGAGATGACTTATAATCCTTTCAAGGAGGATGAAAATTCACCTGCTGATGATGAAGAT
AGTGCAGTTGAGAAGGCTCCTGAAGGCACACCAGCAGGTGGAGGCTTGCTAGTAGTTAGAGTCCTCG
AGGCTGAGGATGTTGAAGGCAAACATCATACTAATCCTTATGTCCGACTTCTTTTCAAAGGAGAGGA
GAAGAAGACTAAGCCTGTCAAGAAAAAATAGAGATCCAAGGTGGGATCAGGAATTTGAATTTATGTTG
GAAGATCCACCTGTCAACGATAAAATCCATGTGGAAGTTATGAGCAAGGGCTCGAGCTTGGCATTGC
ATTCAAGGGAATCTCTGGGTTATGTTGACATTAATCTATCGGATGTGGTGAACAATAAACGTATCAA
TGAGAAATATCATCTTATTGATTCGAAGAATGGAAAACTTCAACTCGAGCTGTTGTGGAGAAGTTCGTAA

Transcripts
co472989
dr500372 (Picea sitchensis)
dv974147
co474659
dv973862
ck436330
dr557947
dr577356
dv990599
co252992
co252893 (record removed)
dv970889
dv977712
dr543273 (Picea sitchensis)
dr492773 (Picea sitchensis)
co257381
co240904
dr566475
co251447
co220867 (Picea sitchensis)
ck435791
dr540734 (Picea sitchensis)
co203255 (Picea engelmannii x sitchensis)
co225636 (Picea sitchensis)
co210582 (Picea engelmannii x sitchensis)
dr562822
co208000 (Picea engelmannii x sitchensis)
dr558231
co234930
dr559481
co234770
co257869

Picea glauca fragment ***NTMC2Type1***
co477109
dv973985
co482192
dr592245
co477969
co217122 (Picea sitchensis)

Picea glauca fragment ***NTMC2Type1***
dr594051

Picea glauca fragment ***NTMC2Type1***
dr593471

Picea glauca fragment ***NTMC2Type2***
co255230
dr555273
co210894 (Picea engelmannii x sitchensis)
dr543682 (Picea sitchensis)
co242865

Picea glauca fragment ***NTMC2Type2***
co472951

Picea glauca fragment ***NTMC2Type2***
co473281

Picea glauca fragment ***NTMC2Type3***
co475322
dv995210

Picea glauca fragment ***NTMC2Type3***
co239740
dv993871
dr450957 (Picea engelmannii x sitchensis)
dr471747 (Picea engelmannii x sitchensis)

Picea glauca fragment ***NTMC2Type4***
dr551971
co481852
dr475023 (Picea engelmannii x sitchensis)
co482048
dr476111 (Picea engelmannii x sitchensis)
dr449157 (Picea engelmannii x sitchensis)
dr469967 (Picea engelmannii x sitchensis)
dr541368 (Picea sitchensis)

Picea engelmannii x glauca fragment ***NTMC2Type5***
dr449894

Pinus taeda Full Length ***NTMC2Type1***
Genes 102 and 104 Craxton, M. BMC Genomics. 2004 Jul 6;5(1):43

MGFVSTLVGFFGFGVGVTIGVIIGYFLFIYFQPTDVKDPIIRPLGELDSKTLEGLLPEIP
LWVKNPDYDRVDWLNTFIHDMWPYLDKAICRKIRDTTKPMIDQYVGKFKIESIEFETLTL
GTLPPTLQGMKVYETQEKELIMEPALKWAGNPNVIVAVKAFGLRATLQMVDLQVFAVPRV
TLKPLVPSFPCFAKIFVSLMEKPHVDFGLKLLGGDVMAIPSLYQFVQEMIKEQVANLYLW
PKTLEVPILDQRATHKPVGMLHVKVVKATDLKKKDMLGKSDPYVKLKLTGEKLPSKKTTV
KQSNLNPEWNEEFNFVVKDPESQALELTVYDWEKVGSHEKMGMQAYHLKELTPSETRSVT
LNLLKTLDPNDPQNIKPRGQITLEMTYNPFKEEENLPVDEDDSVVEKAPEGAPAGGGLLV
VRVIEAEDVEGKHHTNPYVRLLLKGEERKTKPIKKNRDPRWDQEFEFMLEDPPVNDRIHV
EVMSKGSSLGLHARESLGYVDINLSDVVNNKRINEKYHLIDSKNGKIQIELLWRSS

<u>ATG</u>GGTTTTGTGAGTACTTTAGTGGGTTTCTTCGGCTTTGGCGTGGGCGTTACAATTGGTGTTATTA
TTGGGTATTTCCTTTTCATCTACTTCCAGCCCACTGATGTGAAGGACCCTATTATCAGGCCTCTTGG
AGAATTAGATTCAAAGACTTTGGAAGGACTCTTACCTGAAATTCCATTATGGGTGAAAAATCCTGAT
TATGATAGAGTTGATTGGCTCAACACGTTTATTCATGATATGTGGCCTTATCTTGATAAGGCAATCT
GTCGGAAAATAAGAGACACTACAAAGCCTATGATAGACCAATATGTTGGGAAGTTCAAAATAGAATC
TATTGAATTTGAAACACTTACATTGGGCACACTTCCTCCTACACTGCAAGGGATGAAAGTCTATGAA
ACTCAAGAAAAGGAACTGATCATGGAACCTGCTCTCAAATGGGCTGGAAATCCAAATGTGATTGTGG
CAGTGAAAGCATTCGGACTAAGGGCAACTCTGCAGATGGTGGATTTGCAGGTATTTGCAGTTCCACG
TGTCACACTGAAGCCTCTTGTCCCAAGTTTCCCATGCTTTGCAAAAATATTTGTCTCTCTTATGGAG
AAGCCCCACGTTGACTTTGGACTAAAATTACTTGGTGGTGATGTTATGGCTATCCCCAGTCTTTACC
AATTTGTCCAGGAAATGATTAAAGAGCAGGTTGCGAACTTATACTTGTGGCCAAAAACACTGGAGGT
ACCTATTTTGGACCAGAGAGCTACGCATAAACCTGTTGGCATGCTCCACGTAAAGGTTGTTAAGGCA
ACTGACTTAAAAAAGAAAGACATGCTGGGTAAATCCGACCCATATGTAAAGCTCAAGTTGACGGGAG
AAAAACTTCCATCAAAGAAAACCACAGTCAAACAAAGTAATCTGAACCCAGAATGGAATGAGGAGTT
CAACTTTGTTGTTAAAGATCCAGAATCTCAAGCTCTTGAACTTACTGTCTACGACTGGGAGAAGGTG

GGTTCACATGAAAAGATGGGTATGCAAGCTTATCATTTGAAAGAGCTTACACCATCTGAGACCAGAA
GTGTTACGCTGAATCTCCTTAAAACTTTGGATCCAAATGATCCTCAAAATATCAAACCACGTGGACA
AATCACGCTAGAGATGACTTATAATCCTTTCAAGGAGGAAGAAAATTTACCTGTTGATGAGGACGAT
AGTGTAGTTGAGAAGGCTCCTGAAGGTGCACCAGCAGGTGGAGGCTTGCTAGTTGTTAGAGTCATTG
AGGCTGAGGATGTTGAAGGCAAACACCATACTAATCCTTATGTCCGACTTCTTTTGAAAGGGGAGGA
GAGGAAGACTAAGCCTATCAAGAAAAACAGAGATCCAAGGTGGGATCAGGAATTTGAATTTATGTTG
GAAGATCCACCTGTCAATGATAGAATCCATGTGGAAGTTATGAGCAAGGGCTCAAGCCTGGGATTGC
ATGCAAGGGAATCTCTGGGTTATGTTGACATTAATCTATCGGATGTGGTGAATAATAAACGTATCAA
TGAGAAATATCATCTTATTGATTCGAAGAACGGAAAAATTCAAATCGAGCTGTTGTGGAGAAGTTCTTAA

Transcripts
bq290916
bf779042
bq702468
co163365
bf010852
aw226220
aw758642
ct580275 (Pinus pinaster)
dt637486
dr692900
aw784097
dr091410
ct576095 (Pinus pinaster)
dr054664
aw065061
bx679680 (Pinus pinaster)

Pinus taeda fragment ***NTMC2Type1***
Gene 103 Craxton, M. BMC Genomics. 2004 Jul 6;5(1):43
bm427788

Pinus taeda fragment ***NTMC2Type1***
bg318027

Pinus taeda fragment ***NTMC2Type1***
dr055547

Pinus pinaster fragment ***NTMC2Type1***
bx679120

Pinus taeda fragment ***NTMC2Type2***
Gene 105 Craxton, M. BMC Genomics. 2004 Jul 6;5(1):43
bi202834
bq702119
bf609097
ct576975 (Pinus pinaster)

Pinus taeda fragment ***NTMC2Type2***
dt629520
dn615161
dn615165
dt629490

Pinus taeda fragment ***NTMC2Type2***
dn613104
dt628669
dr684029
dn608599
dt629426

Pinus taeda fragment ***NTMC2Type3***
dr097299
dr097212

Pinus taeda fragment ***NTMC2Type4***
dr684141

dt627907
dn610849
ct576772 (Pinus pinaster)

Pinus taeda fragment ***NTMC2Type4***
aw226346
co366076

Pinus taeda fragment ***NTMC2Type6***
bi397625

Pseudotsuga menziesii fragment ***NTMC2Type1***
cn637294

Amborella trichopoda fragment ***NTMC2Type1***
ck748359

Amborella trichopoda fragment ***NTMC2Type5***
ck764140

Nuphar advena fragment ***NTMC2Type2***
cv004767

Nuphar advena fragment ***NTMC2Type4***
cd472451

Liriodendron tulipifera fragment ***NTMC2Type1***
ck743782

Persea americana fragment ***NTMC2Type1***
cv458842

Acorus americanus fragment ***NTMC2Type1***
ck768227

Acorus americanus fragment ***NTMC2Type4***
cv187283

Acorus americanus fragment ***NTMC2Type4***
dt590992

Agrostis stolonifera fragment ***NTMC2Type1***
dv862007

Agrostis stolonifera fragment ***NTMC2Type2***
dv865601

Agrostis capillaris fragment ***NTMC2Type4***
dv857121

Agrostis capillaris fragment ***NTMC2Type6***
dv853608

Agrostis stolonifera fragment ***NTMC2Type6***
dv865994

Allium cepa fragment ***NTMC2Type1***
cf446729
cf443143

Allium cepa fragment ***NTMC2Type2***
cf440841
cf440842

Allium cepa fragment ***NTMC2Type3***
cf439750

Allium cepa fragment ***NTMC2Type4***
cf446838

Allium cepa fragment ***NTMC2Type4***
cf444917

Allium cepa fragment ***NTMC2Type5***
cf434802

Allium cepa fragment ***NTMC2Type6***
cf435819

Allium cepa fragment ***NTMC2Type6***
cf446222

Ananas comosus fragment ***NTMC2Type1***
dt339236

Ananas comosus fragment ***NTMC2Type2***
dt339175

Ananas comosus fragment ***NTMC2Type5***
dt336531

Asparagus officinalis fragment ***NTMC2Type1***
cv288048
cv289361

Asparagus officinalis fragment ***NTMC2Type2***
dy037081
dy033521

Asparagus officinalis fragment ***NTMC2Type4***
cv289154

Avena sativa fragment ***NTMC2Type1***
cn817151
cn817859
cn818543
cn818953
cn820464

Avena sativa fragment ***NTMC2Type1***
cn815321

Avena sativa fragment ***NTMC2Type2***
cn816436

Avena sativa fragment ***NTMC2Type5***
cn819021

Avena sativa fragment ***NTMC2Type6***
aa231647

Avena sativa fragment ***NTMC2Type6***
cn816927
aa231646

Avena sativa fragment ***NTMC2Type6***
cn816343

Brachypodium distachyon fragment ***NTMC2Type1***
dv484488

Brachypodium distachyon fragment ***NTMC2Type2***
dv478409

Brachypodium distachyon fragment ***NTMC2Type5***
dv477547

Brachypodium distachyon fragment ***NTMC2Type6***
dv469041
dv469439
dv480226

Crocus sativus fragment ***NTMC2Type1***
bm956317

Curcuma longa fragment ***NTMC2Type5***
dy394396

Curcuma longa fragment ***NTMC2Type3***
dy389003

Cynodon dactylon fragment ***NTMC2Type4***
dn987073

Eragrostis tef fragment ***NTMC2Type6***
dn483411

Festuca arundinacea fragment ***NTMC2Type1***
dt682127

Festuca arundinacea fragment ***NTMC2Type1***
dt705199
dt703394

Festuca arundinacea fragment ***NTMC2Type1***
dt708308

Festuca arundinacea fragment ***NTMC2Type2***
dt696521

Festuca arundinacea fragment ***NTMC2Type2***
dt701820

Festuca arundinacea fragment ***NTMC2Type3***
dt703630

Festuca arundinacea fragment ***NTMC2Type4***
dt710061
dt709395
dt713392

Festuca arundinacea fragment ***NTMC2Type5***

dt710181
dt691711

Hordeum vulgare Full Length ***NTMC2Type1***

MGVVSTVLGLFGFGFGFSSGIVIGYYFFIYFQPTNVKDVEVRPLVEYDSTSLDGILPEIP
MWVKNPDYDRIDWLNRFLELMWPNLNKAICRMAQDIAKPIIAENCEKYKIDSVEFETLTL
GSLPPTFQGMKVYITDEKELIMEPSLKWAANPNITVVAKAYGLKATVQIVDLQVFASPRI
TLKPLVPTFPCFANISVSLMEKPHVDFGLKLFGADLMAIPVLYKFVQDTIKKQVANMYLW
PKTLEVPIMDPSKASKKPVGILLLKVVRAQNLKKKDLLGKSDPYAKLKMTDDKLPSKKTS
VKRSNLNPEWNEEFKFVVTDPENQSLEVNVFDWEQVGKHEKMGMNRILLKELPPEETKVM
TLNLLKTMDPNDIQNEKSRGQIILEATYKPFKEDDMEKESVDGVDEVQKAPENTPAGGGL
LFVVVHEAQDLEGKHHTNPYAKIIFKGEEKKTKVIKKNRDPRWEDEFEFVCEEPPTNDKL
HVEVLSKAGKKGILHGKEALGYIDITLADVISNKRINEKFHLIDSKNGQIQIELQWRTS

ATGGGCGTAGTCAGCACCGTGCTTGGTCTCTTTGGATTTGGATTTGGATTCTCTTCTGGTATTGTTA
TTGGGTACTACTTCTTCATCTACTTCCAGCCAACGAATGTCAAGGATGTCGAAGTTCGCCCACTTGT
GGAATATGACTCAACTTCATTAGACGGAATCCTTCCTGAAATTCCTATGTGGGTCAAGAATCCCGAC
TATGATAGAATTGATTGGCTCAACAGGTTTCTGGAATTGATGTGGCCCAATCTTAATAAGGCTATCT
GCAGAATGGCGCAGGATATTGCAAAGCCAATTATTGCTGAGAACTGTGAGAAGTACAAGATAGATTC
GGTTGAGTTTGAAACACTTACACTGGGTAGCTTACCACCCACCTTTCAAGGAATGAAAGTCTATATC
ACCGATGAGAAAGAGTTGATAATGGAACCATCTCTAAAGTGGGCTGCAAATCCCAATATTACTGTTG
TTGCGAAGGCCTATGGGTTGAAAGCAACTGTCCAGATTGTGGATCTGCAAGTCTTTGCATCGCCTCG
TATTACTCTGAAGCCGCTGGTGCCTACATTTCCTTGCTTCGCTAATATTAGTGTCTCTCTCATGGAG
AAGCCACATGTTGATTTTGGGCTCAAACTATTCGGAGCAGATTTAATGGCTATTCCTGTTCTTTACA
AATTCGTTCAGGACACCATCAAGAAGCAAGTTGCGAACATGTATCTGTGGCCAAAGACACTAGAAGT
CCCTATAATGGATCCCTCGAAAGCATCAAAGAAGCCTGTTGGAATTCTACTATTGAAGGTTGTAAGA
GCTCAAAATCTGAAAAAGAAGGATCTGCTGGGTAAATCAGATCCATACGCGAAACTTAAGATGACAG
aTGACAAGCTTCCATCCAAGAAAACATCAGTAAAGCGCAGCAATCTCAATCCAGAGTGGAATGAAGA
ATTCAAATTTGTCGTGACAGATCCAGAAAACCAGTCGCTGGAAGTTAATGTCTTCGACTGGGAACAG
GTTGGTAAACATGAAAAGATGGGCATGAACAGGATTCTGTTGAAAGAACTTCCCCCAGAGGAGACTA
AAGTGATGACTCTTAACTTACTGAAGACAATGGATCCAAATGATATACAAAATGAGAAATCTCGTGG
TCAGATTATTTTGGAGGCGACATATAAGCCTTTCAAGGAAGACGACATGGAGAAAGAAAGCGTGGAT
GGTGTCGATGAAGTACAGAAAGCTCCAGAAAATACTCCAGCTGGAGGCGGGCTGCTTTTTGTTGTTG
TTCACGAAGCTCAAGATCTTGAAGGGAAGCATCACACAAACCCATATGCAAAGATAATCTTTAAAGG
AGAAGAGAAGAAAACAAAGGTGATCAAGAAGAACAGGGATCCACGATGGGAGGATGAATTTGAGTTT
GTTTGCGAGGAACCACCTACAAATGATAAGTTGCATGTTGAAGTCCTAAGCAAAGCTGGAAAGAAAG
GAATATTGCATGGCAAGGAAGCCTTGGGCTACATTGATATAACCCTGGCAGACGTGATCAGCAACAA
GCGGATTAACGAGAAGTTCCATCTCATTGACTCTAAAAATGGGCAGATTCAAATCGAGTTGCAGTGG
AGAACTTCATAG

Transcripts

bf265878
av832777
bg414858
av833679
bj452000
cb870208
av914058
av935522
av921286
av919089
bf265282
bq767673
aj436437
bj459559
cx625997
aj486853
cx625924
cd053848
bg343523
ck566364
be438146
cd662572

Hordeum vulgare fragment ***NTMC2Type1***
bi947857
bi952651

Hordeum vulgare fragment ***NTMC2Type1***
bu981534
al511834

Hordeum vulgare Full Length ***NTMC2Type2***

MAFLFGALLGLVLGVGVVMAFARLENSRAEQRRELAATVSSFSKLTVQDLKTLIPTESYP
SWVSFTQKQKLKWLNQELVKIWPFVNEAASELIKSSVEPVFEQYKSFILASIHFSKLTLG
TVAPQFTGVQILDSDSAGITMELDMQWDGNPNIVLDIQTTLGISLPVQVKNIGFTGTLRL
LFKPLVAELPCFGAVCVSLREKSKVDFTLKVVGGEMTAIPGISDAIEGTIRDTIEDTLTW
PNRIIVPIVPGDYSDLELKPVGLLEVKLVEARDLKNKDPLGKSDPFAVLYIRPLSAKTKK
SKTINNDLNPIWNEHYEFVVEDSSTQHLTVKIYDDEGLQPSEIIGCARVDLSDIMPGKVK
DVWLELVKDLEIQRDKKPRGQVHLELLYYPFDKQEGVSNPFASQIQLTSLEKVLKTESNG
YDVNQRKNVITRGVLSVTVISAEDIPAMDVMGKADPFVVLYLKKGETKKKTRVVTETLNP
IWNQTFDFVVEDALHDLLMVEVWDHDTFGKDYIGRCILTLTRAILEGEFQDTYALQGAKS
GRLNLHFKWTAQPIYRDRDRDQ

ATGGCGTTCCTCTTCGGCGCCTTGCTGGGCCTGGTGCTCGGCGTCGGCGTCGTCATGGCG
TTCGCGCGCCTCGAGAATTCCCGCGCCGAGCAGCGCCGCGAGCTGGCTGCTACAGTTTCA
TCTTTCTCAAAATTGACTGTTCAAGATCTGAAGACACTTATTCCTACTGAGTCCTATCCA
TCGTGGGTATCTTTCACCCAAAAGCAGAAGCTTAAATGGCTGAATCAAGAATTGGTGAAA
ATCTGGCCATTTGTCAATGAGGCTGCATCGGAACTGATAAAATCTTCTGTGGAGCCTGTA
TTCGAGCAGTACAAGTCCTTCATTTTGGCCTCCATCCATTTCTCGAAGTTGACACTTGGT
ACTGTCGCTCCTCAGTTTACAGGAGTTCAAATTCTGGATAGTGATAGTGCTGGTATTACT
ATGGAGCTTGATATGCAGTGGGATGGTAATCCCAACATAGTACTTGACATTCAAACAACT
CTGGGAATTTCACTTCCTGTACAGGTGAAAAATATTGGATTCACAGGCACACTACGGCTA
CTCTTTAAGCCTCTGGTAGCCGAACTCCCATGCTTTGGAGCTGTTTGCGTTTCTTTGAGA
GAGAAGAGCAAGGTGGATTTTACCCTCAAAGTTGTTGGTGGCGAAATGACAGCAATTCCT
GGAATTTCTGATGCAATTGAGGGAACAATACGTGATACCATCGAGGACACACTGACATGG
CCTAATCGCATAATTGTCCCCATTGTGCCAGGAGATTATAGTGATCTGGAGCTAAAACCT
GTTGGATTATTAGAAGTAAAACTTGTGGAAGCTAGGGATTTGAAGAACAAGGACCCGTTG
GGGAAGTCTGACCCTTTTGCTGTGCTATACATACGCCCACTGAGTGCAAAAACGAAGAAA
AGCAAAACAATAAACAATGATTTGAACCCCATCTGGAATGAACACTATGAATTTGTGGTC
GAGGACTCATCTACCCAGCACCTGACTGTGAAAATTTACGACGATGAAGGGCTCCAGCCG
TCAGAGATTATTGGCTGCGCTCGTGTAGACTTATCGGATATCATGCCTGGAAAGGTCAAG
GATGTTTGGTTGGAACTTGTGAAAGACCTGGAAATTCAGCGTGATAAGAAACCTCGTGGT
CAGGTCCACCTAGAGCTCCTATACTACCCTTTTGATAAACAAGAAGGAGTTTCCAATCCT
TTTGCTAGTCAGATCCAGTTAACTTCTTTGGAAAAGGTCCTCAAGACGGAATCTAATGGA
TATGATGTCAACCAGAGGAAGAATGTTATTACGAGAGGAGTCCTTTCAGTAACTGTTATA
TCTGCAGAGGACATACCAGCAATGGATGTGATGGGGAAGGCTGACCCATTTGTGGTCCTG
TACCTGAAGAAGGGGGAAACCAAAAAGAAGACAAGGGTTGTGACTGAGACATTGAATCCG
ATATGGAATCAGACATTTGATTTTGTAGTAGAAGATGCCCTGCATGATTTGCTTATGGTG
GAAGTATGGGACCATGATACATTTGGAAAGGATTACATAGGGAGATGCATCTTGACACTT
ACCAGAGCGATACTTGAAGGTGAGTTCCAAGATACATATGCGCTGCAAGGTGCTAAATCT
GGAAGGCTGAACCTGCACTTCAAATGGACGGCACAGCCAATTTACCGTGATCGTGACAGG
GATCAGTGA

Transcripts
ca018655
be455724
bq760748
bu984222
bu970346
aj483685
cb870851
bj481849
bu992885
bj481572
av922773
bj463857
av915343
bj463866
bg365574

dn182708
av920433
dn188678
bj466747
bu987790
av927494
bj468026
bi959351
bj486242
bj486644
cx632082
aj461357
bj468035
bu970948
aj460210

Hordeum vulgare fragment ***NTMC2Type2***
bm371438
bm371407
bq548066
be439011
dn159292
bj461611
aj000238
bq548067
be438982

Hordeum vulgare fragment ***NTMC2Type2***
bm372753

Hordeum vulgare fragment ***NTMC2Type3***
dn188986
ck565959
dn183795
dn189936
cx632745
cx627756
cx626274
ca016550

Hordeum vulgare fragment ***NTMC2Type3***
be194293

Hordeum vulgare fragment ***NTMC2Type3***
ca018315

Hordeum vulgare fragment ***NTMC2Type3***
cd054256

Hordeum vulgare fragment ***NTMC2Type4***
av916010
dn181859
ca002839
cd055852
ck568563
bj468725
dn188306
dn185564
av921127
al510159
av945135
av921551
av930556

Hordeum vulgare fragment ***NTMC2Type4***
bj480785
av925402
bj465477
dn178660
av940038
av913937
bf627916

Hordeum vulgare fragment ***NTMC2Type5***
bg299490
be194330
cd055819
bq470136
ck568532
aj462130
cx626932

Hordeum vulgare fragment ***NTMC2Type5***
bj467034
av918392
av917133

Hordeum vulgare fragment ***NTMC2Type6***
av833523
dn180479
cd056024
bu988504
bu989105
ca023171
av920554
bg415765
av918837

Hordeum vulgare fragment ***NTMC2Type6***
cx627438
cx626622
bm441346
bm372534

Hordeum vulgare fragment ***NTMC2Type6***
bg299269
bf260819
bf624525

Hordeum vulgare fragment ***NTMC2Type6***
ca024253
av833187

Hordeum vulgare fragment ***NTMC2Type6***
bq755822

Leymus chinensis fragment ***NTMC2Type6***
cn466135

Lilium longiflorum fragment ***NTMC2Type1***
bp177416

Lolium temulentum fragment ***NTMC2Type1***
dt670689

Lolium temulentum fragment ***NTMC2Type4***
dt672583

Lolium temulentum fragment ***NTMC2Type5***
dt673534
dt673558

Lycoris longituba fragment ***NTMC2Type4***
cn451760

Musa acuminata fragment ***NTMC2Type1***
dt723886

Oryza sativa Full Length ***NTMC2Type1.1***
Gene 89 Craxton, M. BMC Genomics. 2004 Jul 6;5(1):43
ci170953
ak107799
ck049757
ck069322
ci667242
ci663656
ci392696

Oryza sativa Full Length ***NTMC2Type1.2***
Gene 90 Craxton, M. BMC Genomics. 2004 Jul 6;5(1):43
ak068006
ck053434
ci654030
ci609967
ci586138
ak065681
cr287822
ck051163
ak068206
ck015154
cf303673
cf324992
cx118561
ci339966
ci334919
ci335841
ci363206
ci395060
ci388311
au030858
ci145541
cb683410
cr287801
ca755689
cf956190
au101494
bx901101
bx901097
aq840555

Oryza sativa Full Length ***NTMC2Type1.3***
Gene 91 Craxton, M. BMC Genomics. 2004 Jul 6;5(1):43
ck061684
ck009754
ck038532
cb637033
ck059090
ck054068
ck040367
ck039966
ak064509
ck045612
ck046917
ck037668
cx113249
ck039554
cf991668
cf326078
ck074996
ck042641
cr281097
d22040
cb000316
ci260303
au100617
ci138644
cb683132
cb637034
ci180583
ci180572
cr286214
au096229
bq908396

Oryza sativa Full Length ***NTMC2Type1.4***

Gene 88 Craxton, M. BMC Genomics. 2004 Jul 6;5(1):43

Oryza sativa Full Length ***NTMC2Type1.5***

Gene 92 Craxton, M. BMC Genomics. 2004 Jul 6;5(1):43
cb657575
cf304573
c26570
aa752034
au057096
au057097
bm421741
au181420

Oryza sativa Full Length ***NTMC2Type2.1***

Gene 95 Craxton, M. BMC Genomics. 2004 Jul 6;5(1):43
ck034062
ck049676
cf306819
ck053102
ck063320
ck067089
ca998179
ci032780
ak062763
ci297453
ci045878
ci537693
ci525929
ci537713

Oryza sativa Full Length ***NTMC2Type2.2***

Gene 96 Craxton, M. BMC Genomics. 2004 Jul 6;5(1):43
ak069706
ak119574
cx106297
ci650922
ci580339
cb674323
ci667604
ci638238
ci580959
ci592857
ci772510
ci764761
ci759731
ci569484
ci768499
c28110
ck068164
ck072627
ci257098
ci089108
ci106585
ci040971
ci229845
ck055956
ci092821
ci320914
ci117592
ci313738
ci089532
ci162408
ci099967
ci350147
ck078350
ci447390
ci125724
ci424337
ci552172
bi811680
ci438686
ci419723
ci556445
ci126796

ci073637
cb637288
ck058562
ci409220
ci095812
ci343821
ci542819
ci444894

Oryza sativa Full Length ***NTMC2Type3***
Gene 98 Craxton, M. BMC Genomics. 2004 Jul 6;5(1):43
ci565880
ca767264
ci576285
ak120447
ci583692
cf993060
ck083349
ck083057
ck079387
cv728528
bi812030
bq908984
bm421263
bi805222

Oryza sativa Full Length ***NTMC2Type4.1***
Gene 113 Craxton, M. BMC Genomics. 2004 Jul 6;5(1):43
cb635147
cb637448
bx899001
cb659206
cb652250
ak060230
ck034709
cr287882
cb630004
cb630002
ck070025
ci310696
cb661402
cb681535
cb654417
ck053240
cb685859
bf428998
bm326372
be429243
cb659255
cb661403
cb632166
cb653700
cb681536
cb630003
cb654418
cb685860
cr292586
ci123676
ck062549
ci759516
cb637449
ck079520
bi802084
ci049450
ci000757
ci032039
ca756581
bi810514

Oryza sativa Full Length ***NTMC2Type4.2***
Gene 114 Craxton, M. BMC Genomics. 2004 Jul 6;5(1):43
This gene as descirbed in the above reference is incorrect. The corrected gene description is below.
This has been altered June 2006

chromosome 3
no evidence of expression

MGFISGVVMGMIIGVALIAGWSRAMARRAAKRSAK**^ 0**
AADVNALASLDREDVKKICGENLPEWVSFPEYEQ**^ 0**
VKWLNKQLSKLWPFVEE**^ 0**
AATMVIRDSVEPILDDYRPAGISSLKFSKLSLGTVPPKIE**^ +1**
GIRIQSFKKGQITMDVDFRWGGDPNIVLAVDTLVASLPIQ**^ 0**
FKNLQVYTIIRVVFQLCDEIPCISAVVVALLAE**^ 0**
PKPRIDYILKAVGGSLTAMPGLSDMID**^ 0**
DTVASLIADMLQWPHRIVVPLGGVDVDV**^ +2**
SDLELKPHGKLTVTVVRAESLKNKELIGKSDPYVVLYIRPMFKEKTSVIDDNLNPEW
NETFSLIAEDKETQHLILQ**^ 0**
VFDEDKLKQDKRLGIAKLPLNDLEMESVQEINLQLLSSLDTTKVKDKKDRGVLTIK**^ 0**
VLYHPFTKAEALEALELEKKTVEERRKTKEETAAVSGAADAASGVTSTVTPAAGAGVAAG
AAAPGAGATAAGSGVGLVGTGIGAVGSGIGAFGTGLSKAGKFVGRTVTGPFSSARRSASS
VPTIDE

<u>ATG</u>GGGTTCATATCGGGGGTGGTGATGGGAATGATCATCGGCGTCGCCCTCATCGCCGGC
TGGTCGCGCGCGATGGCTCGCCGCGCCGCCAAACGCAGTGCCAAG
phase 0
ac134235.2 (149526-149422) atypical gc donor

GCTGCAGATGTCAATGCACTTGCATCTCTCGACCGCGAAGACGTGAAGAAAATATGTGGA
GAAAATCTCCCAGAATGGGTGTCATTCCCAGAGTATGAACAG
phase 0
ac134235.2 (148905-148804)

GTTAAATGGCTCAACAAACAATTGAGCAAGCTTTGGCCTTTTGTTGAAGAG
phase 0
ac134235.2 (148644-148594)

GCAGCAACTATGGTCATCAGGGATTCGGTGGAGCCTATACTTGATGATTACAGGCCGGCA
GGGATATCCTCACTGAAGTTCAGCAAACTCTCCCTGGGGACTGTTCCTCCGAAAATTGAAG
phase +1
ac134235.2 (148440-148320)

GCATTCGGATTCAGAGCTTCAAGAAAGGGCAAATTACAATGGACGTCGACTTTCGTTGGG
GCGGGGATCCTAATATTGTCCTTGCAGTTGACACTCTAGTTGCTTCACTTCCCATTCAG
phase 0
ac134235.2 (148229-148111)

TTCAAGAACCTTCAGGTTTACACCATCATCCGTGTCGTCTTCCAATTGTGTGATGAAATA
CCTTGCATCTCTGCTGTTGTTGTTGCTCTTCTGGCAGAG
phase 0
ac134235.2 (147963-147865)

CCAAAACCAAGGATCGACTACATACTGAAGGCTGTCGGAGGAAGCCTGACAGCAATGCCT
GGTCTTTCAGATATGATCGAC
phase 0
ac134235.2 (147752-147672)

GACACCGTGGCATCTTTGATCGCTGACATGCTCCAATGGCCACACAGAATCGTCGTCCCA
CTGGGCGGAGTCGACGTCGACGTAAG
phase +2
ac134235.2 (147573-147488)

TGACCTTGAGCTGAAGCCGCATGGGAAGCTGACGGTGACCGTGGTGCGCGCGGAGTCGCT
CAAGAACAAGGAGCTGATCGGCAAGTCCGACCCGTACGTGGTGCTGTACATCCGCCCAAT
GTTCAAGGAGAAGACCAGCGTCATCGACGACAACCTCAACCCCGAGTGGAACGAGACGTT
CTCGCTCATCGCCGAGGACAAGGAGACGCAGCATCTGATCCTTCAG
phase 0
ac134235.2 (147346-147121)

GTGTTCGACGAGGACAAGCTGAAGCAAGACAAGAGGCTGGGCATAGCCAAGCTGCCCCTG
AACGACCTGGAGATGGAGTCCGTGCAGGAGATCAACCTGCAGCTGCTGTCGTCGCTGGAC
ACGACCAAGGTCAAGGACAAGAAGGACAGGGGCGTGCTCACCATCAAG
phase 0
ac134235.2 (147035-146868)

GTGCTGTACCACCCGTTCACCAAGGCGGAGGCGCTCGAGGCGCTGGAGCTGGAGAAGAAG
ACGGTGGAGGAGCGGCGGAAGACCAAGGAGGAGACGGCGGCCGTCAGCGGCGCCGCGGAC
GCGGCGAGCGGCGTGACGTCCACGGTGACCCCCGCGGCTGGGGCGGGCGTCGCTGCGGGA
GCCGCCGCGCCCGGAGCGGGCGCCACCGCCGCCGGCTCCGGCGTGGGGCTGGTCGGCACG
GGCATCGGCGCCGTGGGCAGCGGCATCGGAGCGTTCGGCACCGGCCTCAGCAAGGCTGGC
AAGTTCGTCGGCCGGACCGTCACTGGCCCGTTCAGCAGCGCCAGGCGCAGCGCCAGCAGC
GTGCCCACCATTGACGAATAA
ac134235.2 (146777-146397)

Oryza sativa Full Length ***NTMC2Type5***
Gene 115 Craxton, M. BMC Genomics. 2004 Jul 6;5(1):43
cb630057
ak070520
cb644777
cb652221
ck061403
ci647227
cb643344
ck038985
ck039386
cb644778
ci124218
cb630056
c27937
ci273322
cf991817
ck085305
ci406749
ci426876
ci131859
ci313732
ck054603
ci078438
ci000506
ci128254
ci337994
ck033636
ci344044
ci387847
ci443785
ci431926
ci533624

Oryza sativa Full Length ***NTMC2Type6.1***
Gene 116 Craxton, M. BMC Genomics. 2004 Jul 6;5(1):43
ci606886
ci606887
ci570478
ci575058
ci578702
ak067756
ci579906
ci570553
cb636971
ci767068
ci757862
ci755730
ci563636
ak098860
ci738569
ci773119
ci593281
ci576435
ci574385
ci584511
ci565550
ci575151
ci626623
cb645326
ci576492
ci565049
ck078793
cb672710
bp432945
ck071809

cf991789
cf312582
cb645327
cb646269
cb672711
ci136090
ci312102
ci030285
ci327289
ci321933
ci315194
cb636972
ci037674
cb637062
ci315831
cb636967
ci331299
ci368214
ci296500
ci350585
ci336757
ci045163
cf957055
ci324976
ca760978

Oryza sativa Full Length ***NTMC2Type6.2***

Gene 117 Craxton, M. BMC Genomics. 2004 Jul 6;5(1):43
cb671727
ak072366
ci593643
ci743847
ci650924
ci640335
ci654945
ci645701
ci645091
ci664536
ak101258
ci607791
ci759657
ci776286
cf196551
cf196668
ci366730
ci350928
ck062651
ci194618
ci122371
ci269920
ca766103
ci194611
cf304094
cf304703

Panicum virgatum fragment ***NTMC2Type1***

dn152143

Panicum virgatum fragment ***NTMC2Type3***

dn142170

Panicum virgatum fragment ***NTMC2Type4***

dn146301

Panicum virgatum fragment ***NTMC2Type6***

dn141747

Pennisetum glaucum fragment ***NTMC2Type5***

cd726369

Saccharum officinarum fragment ***NTMC2Type1***

ca194874
bq531152

ca193135
ca167642
ca164616
ca294919
ca180213
ca179720
bu102871
ca097106
ca097140
ca158220
ca158231
ca159723
ca179705
ca156151
ca159072
ca155696
ca162564
ca185692
ca182587
ca165532

Saccharum officinarum fragment ***NTMC2Type1***
ca292988
ca121456
ca201490
ca220204
ca195253
ca159803
ca294978

Saccharum officinarum fragment ***NTMC2Type1***
ca207242
ca192950
ca184297
ca081629
ca272140

Saccharum officinarum fragment ***NTMC2Type1***
bq534268
ca088487
cf572969

Saccharum officinarum fragment ***NTMC2Type1***
ca197985

Saccharum officinarum fragment ***NTMC2Type1***
ca211878

Saccharum officinarum fragment ***NTMC2Type1***
ca128230

Saccharum officinarum fragment ***NTMC2Type1***
ca081972

Saccharum officinarum fragment ***NTMC2Type2***
ca206543
ca171580
ca207579
ca178902
ca084824
ca092099

Saccharum officinarum fragment ***NTMC2Type2***
ca230764
ca241440
ca213779
cf573029 (Saccharum hybrid)
ca160478

Saccharum officinarum fragment ***NTMC2Type2***
ca299693
cf577180
ca235684
ca235602

Saccharum officinarum fragment ***NTMC2Type2***
ca065316
ca213695

Saccharum officinarum fragment ***NTMC2Type2***
ca091900

Saccharum officinarum fragment ***NTMC2Type2***
ca160472

Saccharum hybrid fragment ***NTMC2Type2***
cf574571

Saccharum officinarum fragment ***NTMC2Type3***
cf571765 (Saccharum hybrid)
ca261663
ca139888
ca099773
ca200173
ca212329

Saccharum officinarum Full Length ***NTMC2Type4***

MGLISGMVMGVMVGVAxMAGWSRVMRRRSTKRIAKAADIKVLGSLTRDDLRKLCGDSFPE
WISFPQFEQVKWLNKHLSKLWPFVVEAATVVVKESVEPLLDDYRPPGIKSLKFRKFSLGN
VSPKIEGIRIQNLQPGQIIMDIDFRWGGNPSIILAVDAVVASLPIQLKDLEVYTVIRVIF
QLSEDIPCISAVVVALLADPEPKIQYTLKAIGGSLTAVPGLSDMIDDTVNSIVSDMLLWP
HRHVVKLGVNVDTSDLELKPQGRLSVTVVKATSLRNKEMIGKSDPYVKLYVRPMFKVKTK
VIDDDLNPEWNETFDLIVEDKETQSVIFEVYDEDNLQQDKRLGVAKLAVNTLEPVITQEV
TLKLLHSLDPIKNRDTKDRGTLHLKVKYHPFTKEEQLEALEMEKQAIEERKRLKEAGVIG
STMDAVGGAASLVGSGVGLVGTGIGAGIGLVGSGIGAGAGLVGSGIGAVGSGLGKAGKFM
GRTVTGPFSMSRKNGSSSTAPQPDQPSA

ATGGGGCTGATATCCGGGATGGTGATGGGGGTCATGGTCGGCGTCGCCnTCATGGCCGGCTGGAGCCG
CGTCATGCGCCGGCGCAGCACGAAGCGCATCGCCAAGGCAGCGGATATCAAGGTGCTCGGGTCTCTCA
CCAGGGACGACCTCAGGAAGCTGTGCGGTGATAGCTTCCCGGAGTGGATATCCTTCCCGCAGTTTGAG
CAGGTTAAATGGCTGAACAAGCATCTAAGCAAACTTTGGCCTTTTGTTGTAGAAGCTGCAACAGTAGT
GGTTAAGGAATCCGTTGAACCACTGCTAGATGATTACCGGCCTCCAGGAATAAAATCTTTGAAGTTCA
GAAAATTTTCTCTTGGAAATGTGTCACCAAAGATAGAAGGTATTCGTATTCAAAATCTTCAACCTGGC
CAAATCATCATGGATATAGATTTCCGTTGGGGTGGTAATCCAAGCATAATCCTAGCTGTTGATGCTGT
AGTTGCATCACTGCCTATTCAGCTCAAGGATCTTGAGGTCTATACTGTCATACGTGTTATATTTCAAC
TGTCTGAAGACATCCCTTGCATTTCTGCTGTTGTTGTGGCTCTCCTTGCCGATCCAGAACCAAAAATT
CAGTACACCCTGAAGGCCATTGGAGGAAGCCTAACTGCAGTTCCTGGACTTTCTGACATGATTGATGA
CACTGTCAATTCAATTGTTTCTGATATGCTCTTGTGGCCACACAGGCATGTTGTTAAACTTGGTGTCA
ATGTTGATACAAGTGACTTGGAGCTTAAACCTCAGGGAAGACTTTCTGTTACTGTGGTAAAAGCAACT
TCTTTGAGAAATAAGGAGATGATCGGTAAATCAGACCCTTATGTGAAATTGTACGTGCGGCCAATGTT
TAAGGTCAAAACAAAAGTCATAGATGACGACCTGAATCCGGAATGGAATGAAACATTTGATCTGATTG
TCGAAGACAAAGAAACCCAATCTGTCATTTTTGAGGTTTACGACGAAGACAACCTTCAGCAAGACAAG
AGGCTGGGTGTGGCAAAACTAGCAGTGAACACTCTTGAACCTGTGATCACCCAAGAAGTCACTCTGAA
GCTGCTACATTCACTGGATCCAATTAAAAATAGGGACACCAAGGATAGAGGCACATTGCATCTTAAGG
TAAAGTATCACCCCTTTACAAAGGAAGAGCAGCTGGAAGCCCTAGAGATGGAAAAGCAAGCCATAGAG
GAGAGGAAGCGGCTGAAGGAGGCTGGGGTAATTGGTAGCACAATGGATGCTGTTGGCGGTGCTGCATC
ACTAGTTGGTTCCGGTGTTGGACTTGTGGGCACTGGCATTGGTGCTGGCATTGGCCTCGTTGGTTCAG
GAATTGGTGCTGGCGCTGGCCTTGTTGGCTCTGGTATTGGTGCTGTCGGCAGTGGCCTTGGTAAAGCC
GGGAAGTTCATGGGCAGGACTGTGACAGGGCCTTTCAGCATGTCCCGCAAGAACGGCAGCAGCTCTAC
TGCTCCTCAGCCTGATCAACCTTCGGCATAG

Transcripts

ca173853
ca248399
ca163109
ca088378
ca088379
ca088374
ca196495
ca211662
ca262938
ca283428
ca205527
ca249395
ca215269
ca070998
ca258092
ca282937
ca248551
ca284806

Saccharum officinarum fragment ***NTMC2Type5***
ca178115
ca206288
ca191850
ca285179
ca149676

Saccharum officinarum fragment ***NTMC2Type5***
ca078838
ca291552
ca078809

Saccharum officinarum fragment ***NTMC2Type5***
ca245451
ca155252

Saccharum officinarum fragment ***NTMC2Type6***
ca135047
cf574770
ca195947
ca157100

Saccharum officinarum fragment ***NTMC2Type6***
ca138725
ca137216

Saccharum officinarum fragment ***NTMC2Type6***
ca184808

Saccharum officinarum fragment ***NTMC2Type6***
ca201634

Saccharum officinarum fragment ***NTMC2Type6***
bq533045

Saccharum officinarum fragment ***NTMC2Type6***
ca201714

Saccharum officinarum fragment ***NTMC2Type6***
ca068648

Saccharum officinarum fragment ***NTMC2Type6***
ca068575

Saccharum officinarum fragment ***NTMC2Type6***
ca253749

Sorghum bicolor Full Length ***NTMC2Type1***

MGVISTVLGFTGFGFGFSAGIVIGYFLFIYVQPADVKDVKVRPLVEYDSKSLEGILPEIP
LWVKNPDYDRIDWLNRFLELMWPYLDKAICRTAQDIAKPIIAENTAKYKIDSVEFETLTL
GSLPPTFQGMKVYVTEEQELIMEPCLKWAANPNVTVVIKAYGLKATVQIVDLQVFALPRI
TLKPLVPTFPCFAKILVSLMEKPHVDFGLKILGADVMAIPGLYRFVQETIKKQVAIMYLW
PKTLEVPIMDPSKASKKPVGILLVKVIRAQNLRKKDLLGKSDPYVKLKMSDDKLPSKKTT
VKRSNLNPEWNEDFKFVVTDPENQALEVNVFDWEQVGKHEKMGMNMxPTARGPPEETKVT
TLNLLKTMDPNDVQNEKSRGQLTLELTYKPFKEEDGEIEDTEGTNVIEKAPDGTPAGGGL
LFVIVHEAKDLEGKHHTNPYAKIIFKGEEKKTKVIKKNRDPRWVDEFEFVCEEPPVNDKL
HVEVLSKAPKKGLIYGKETLGYIDVSLADVISNKRINEKYHLIDSKNGQIQIELQWRTS

ATGGGTGTAATCAGCACTGTTCTTGGTTTCACTGGATTTGGCTTTGGGTTCTCGGCTGGCATTGTTATTGG
GTACTTCCTCTTCATCTATGTCCAGCCAGCTGATGTCAAGGATGTCAAAGTTCGCCCGCTTGTGGAATATG
ATTCAAAATCTTTGGAGGGCATCCTTCCTGAAATTCCACTGTGGGTCAAGAATCCCGACTATGATAGAATT
GATTGGCTGAACAGGTTTTTGGAATTGATGTGGCCTTATCTTGACAAGGCTATCTGCAGAACTGCACAGGA
TATCGCAAAGCCAATTATTGCTGAGAATACTGCAAAGTATAAGATAGACTCTGTTGAGTTTGAAACACTTA
CTTTGGGTAGTCTACCACCCACCTTTCAAGGGATGAAAGTCTATGTCACAGAGGAGCAAGAGTTGATAATG
GAACCATGTCTTAAATGGGCTGCAAATCCAAATGTCACAGTTGTTATAAAGGCTTATGGTTTGAAAGCTAC
TGTCCAGATTGTTGATCTACAAGTCTTTGCATTACCTCGTATTACTCTGAAGCCATTAGTGCCTACATTTC
CTTGCTTTGCAAAAATCCTTGTCTCGCTAATGGAGAAGCCACATGTTGACTTTGGGCTAAAGATTCTTGGA
GCTGATGTAATGGCTATTCCTGGTCTTTACAGATTTGTCCAGGAGACCATCAAGAAGCAAGTCGCAATCAT
GTATTTGTGGCCGAAGACACTAGAAGTACCTATAATGGACCCCTCAAAAGCATCAAAAAAGCCTGTTGGAA
TTCTACTTGTGAAGGTCATAAGAGCCCAAAATTTGCGAAAGAAGGATCTGCTGGGTAAATCAGACCCATAT
GTGAAACTTAAAATGTCAGATGATAAGCTTCCATCCAAGAAGACTACTGTAAAGCGCAGCAATCTCAATCC
AGAGTGGAATGAAGATTTTAAGTTTGTAGTGACTGATCCAGAAAACCAGGCTCTTGAAGTTAATGTCTTCG
ACTGGGAACAGGTTGGGAAACATGAAAAGATGGGAATGAACATGGnTCCTACTGCACGAGGCCCACCTGAG
GAGACTAAAGTTACTACCCTCAACTTGCTTAAGACCATGGATCCAAATGATGTACAAAATGAGAAGTCTCG
TGGCCAGCTTACTCTAGAGCTCACATACAAACCTTTCAAGGAAGAAGATGGCGAGATAGAAGATACAGAGG
GTACCAATGTGATAGAAAAAGCTCCAGATGGCACTCCAGCTGGCGGTGGATTGCTTTTTGTTATTGTTCAT
GAAGCCAAAGATCTTGAGGGGAAGCACCATACAAACCCCTATGCAAAAATAATTTTCAAAGGCGAAGAGAA
GAAAACAAAGGTCATCAAGAAGAATAGGGATCCACGATGGGTGGATGAATTCGAGTTTGTGTGCGAGGAGC
CTCCTGTAAATGATAAACTACATGTTGAAGTCCTAAGTAAAGCCCCGAAGAAAGGGCTGATATATGGCAAG
GAAACTTTGGGCTACATTGATGTCAGCCTTGCAGACGTGATCAGTAACAAGAGAATTAATGAGAAGTACCA
TCTCATAGACTCAAAAAATGGTCAGATCCAAATTGAGTTGCAGTGGAGAACTTCCTAG

Transcripts
be356447
cn130479
cf487242
bm323269
bm324923
bm326043
bg947875
bm324400
cn130893
bg356253
bg240590
bf420873
bf317938
cn130404
aw565309
bm329411
be356518
bg240004
bm331356
bm328885
bg356518
bi076363
cf487160
bf317801
bg947582
bf317699

Sorghum bicolor fragment ***NTMC2Type1***
cd231788
cn138909
bg241179
bg240530
cn138849

bg240860
bg239951
cf757726
cf481272

Sorghum bicolor fragment ***NTMC2Type2***
cd423173
bm317619
cb924551
cd461548
cd462153
cn148944
cb927872
cx608117
bg104099 (Sorghum propinquum)
bm324295
bg948678
bg948560
cf761800
cx608037
bg103762 (Sorghum propinquum)
cd462072
cb927788
cb924454
bg948359

Sorghum bicolor fragment ***NTMC2Type2***
cd206538
cx615993
bg946959
cd219530
cd206519
cf071897
cd423442
bg946877

Sorghum bicolor fragment ***NTMC2Type2***
cd423519

Sorghum bicolor fragment ***NTMC2Type3***
be363249
aw676834
aw676847
cd234636
bm328163
cd425885
bm327129
cd425575
bg356744

Sorghum bicolor fragment ***NTMC2Type3***
cd426024
bm323659
bm322735

Sorghum bicolor fragment ***NTMC2Type3***
cd234733

Sorghum bicolor fragment ***NTMC2Type4***
be355024
cd431731
cn139219
cn139144
bm330762
be355214

Sorghum bicolor fragment ***NTMC2Type4***
cn136088
cn136005

Sorghum bicolor fragment ***NTMC2Type5***
cd426079
cd423676
cx610129

Sorghum bicolor fragment ***NTMC2Type5***
cn138306
cf757198
cf757909

Sorghum bicolor fragment ***NTMC2Type5***
cn138390

Sorghum bicolor fragment ***NTMC2Type5***
cd206393

Sorghum propinquum fragment ***NTMC2Type5***
bg104024

Sorghum bicolor fragment ***NTMC2Type6***
cf760156
bg464600
aw676691
cf757172
bg557621
bf587822

Sorghum bicolor fragment ***NTMC2Type6***
be125131
bg464277

Sorghum bicolor fragment ***NTMC2Type6***
bg557511

Triticum aestivum Full Length ***NTMC2Type1***

MGVVSTVLGLFGFGFGFSSGIAIGYYFFIYFQPTNVKDVEVRPLVEYDSNSLDGILPEIP
MWVKNPDYDRIDWLNRFLELMWPNLNKAICRMAQDIAKPIIAENCEKYKIDSVEFETLTL
GTLPPTFQGMKVYVTDEKELIMEPSLKWAANPNITVVAKAYGLKATVQIVDLQVFASPRI
TLKPLVPTFPCFANxSVSLMEKPHVDxGLKLFGADLMAIPVLYKFVQDTIKKQVGNMYLW
PKTLEVPIMDPSKASKxPVGILLxKVVRAQNLKKKDLLGKSDPYAKLKMTDDKLPSKKTS
VxRSNLNPEWNEDFKFVVTDPENQSLEVDVFDWEQVGKHEKMGMNMVLLKELPPEETKVL
TLNLLKTMDPNDIQNEKSRGQIILEATYKPFKEEDMEKExVDGxDEVQKAPDNTPAGGGL
LFVVVHEAQDLEGKHHTNPYAKIIFKGEEKKTKVIKKNRDPRWEDxFEFVCEEPPTNDKL
HVEVLSKAGKKGILHGKEALGYIDISLADVISNKRINEKFHLIDSKNGQIQIELQWRTS

ATGGGCGTAGTCAGCACGGTGCTTGGTCTCTTTGGATTTGGATTTGGATTCTCTTCTGGTATTGCTATT
GGGTACTACTTCTTCATCTACTTCCAGCCAACGAATGTCAAGGATGTCGAAGTTCGCCCACTTGTGGAA
TATGACTCAAATTCTTTGGATGGAATCCTTCCTGAAATTCCTATGTGGGTCAAGAATCCCGACTATGAT
AGAATTGATTGGCTCAACAGGTTTCTGGAATTGATGTGGCCCAATCTTAATAAGGCTATCTGCAGAATG
GCGCAGGATATTGCAAAGCCAATTATTGCTGAGAACTGTGAGAAGTACAAGATAGATTCGGTTGAGTTC
GAAACACTTACTCTGGGTACCTTACCACCCACCTTTCAAGGAATGAAAGTCTATGTCACCGATGAGAAA
GAGTTGATAATGGAACCATCTCTAAAGTGGGCTGCAAATCCCAATATTACTGTTGTTGCAAAGGCGTAC
GGGTTGAAAGCAACCGTCCAAATTGTGGATCTGCAAGTCTTTGCATCnCCTCGTATTACTCTGAAGCCG
TTGGTGCCTACATTTCCTTGCTTCGCTAATATnAGTGTnTCTCTCATGGAGAAGCCACATGTTGATTTn
GGGCTCAAACTATTCGGAGCAGATTTAATGGCTATTCCTGTTCTTTACAAATTCGTTCAGGACACCATT
AAGAAGCAAGTTGGGAACATGTATCTGTGGCCAAAGACACTAGAAGTCCCTATAATGGATCCCTCnAAA
GCATCAAAGAnGCCTGTTGGAATTCTACTTnTGAAGGTTGTAAGAGCTCAAAATCTGAAAAAGAAGGAT
CTGCTGGGTAAATCAGATCCATACGCGAAACTTAAGATGACAGATGACAAGCTTCCATCCAAGAAAACA
TCAGTAAAnCGCAGCAATCTCAATCCnGAGTGGAATGAAGATTTCAAATTTGTCGTGACAGATCCAGAA
AACCAGTCGCTGGAAGTTGATGTCTTTGACTGGGAACAGGTTGGTAAACATGAAAAGATGGGCATGAAC
ATGGTTCTGTTGAAAGAACTTCCCCCAGAGGAGACTAAAGTGTTGACTCTTAACTTGCTGAAGACCATG
GATCCAAATGATATACAAAATGAGAAATCTCGTGGTCAGATTATTTTGGAGGCGACATATAAGCCTTTC
AAGGAAGAAGATATGGAGAAAGAAAnCGTGGATGGTGnTGATGAAGTGCAGAAAGCTCCAGACAATACT

CCAGCTGGTGGTGGGCTGCTTTTTGTTGTTGTTCACGAAGCTCAAGATCTTGAAGGGAAGCATCACACA
AACCCATACGCAAAGATAATCTTTAAAGGAGAAGAGAAGAAAACAAAGGTGATCAAGAAGAACAGGGAT
CCACGATGGGAGGATGAnTTTGAGTTTGTTTGTGAGGAACCACCTACAAATGATAAATTGCATGTTGAA
GTCCTAAGTAAAGCTGGAAAGAAAGGAATATTGCATGGCAAGGAAGCCTTGGGCTACATTGATATATCC
CTGGCAGACGTGATCAGCAACAAGCGGATCAACGAGAAGTTCCATCTCATTGACTCGAAAAATGGGCAG
ATTCAAATCGAGTTGCAGTGGAGAACTTCATAG

Transcripts
cj699338
cj502332
bg906660
bg263622
cj672084
cj502142
cj496725
ca501275
cj635288
bj235455
cd929832
al817151
ca502468
cv778467
be428825
bm135578
cd887226
ck155489
bg906661
ck155479
bq247911
cd916585
al827949
cd454764
be419369
cd929833
ca665948
ca628753
bq171547
be444750
be443322
be443445
be446213
be445873
bq161538
ca648049

Triticum aestivum fragment ***NTMC2Type1***
ca620713
ca622766

Triticum aestivum fragment ***NTMC2Type1***
ca706949

Triticum aestivum fragment ***NTMC2Type1***
bj307101

Triticum aestivum fragment ***NTMC2Type1***
bq620491

Triticum aestivum Full Length ***NTMC2Type2***

MAFLFGALLGLVLGVGVVMAFARIENSRAEQRRQLAATVSSFSKLSVQDLKTLIPTEAYP
SWVSFNQKQKLKWLNQELVKIWPFVNEAASELIKSSVEPVFEQYKSFILASIHFSKLTLG
TVAPQFTGVQILDSDSAGITMELDMQWDGNPNIVLDIQTTLGISLPVQVKNIGFTGTLRL
LFKPLVAELPCFGAVCVSLREKSKVDFTLKVVGGEMTAIPGISDAIEGTIRDTIEDTLTW
PNRIIVPIVPGDYSDLELKPVGLLEVKLVEARDLKNKDPLGKSDPFAVLYIRPLSAKTKK
SKTINNDLNPIWNEHYEFVVEDSSTQHLTVKIYDDEGLQPSEIIGCARVDLSDVTPGKVK
DVWLELVKDLEIQRDKKPRGQVHLELLYYPFEKQEGVSNPFAGQIQLTSLEKVLKTESNG
YDVNQRKNVITRGVLSVTVISAEDIPAMDVMGKADPFVVLYLKKGETKKKTRVVTETLNP
IWNQTFDFVVEDALHDLLIVEVWDHDTFGKDYIGRCILTLTRAILEGEFQDTYVLQGAKS
GKLNLHFKWTAQPIYRDRDRDQ

ATGGCGTTCCTCTTCGGCGCCCTGCTGGGGCTGGTGCTCGGCGTCGGCGTCGTCATGGCG
TTCGCGCGCATCGAGAATTCCCGCGCCGAGCAGCGCCGCCAGCTGGCTGCTACAGTTTCA
TCTTTCTCAAAATTGTCTGTTCAAGATCTGAAGACACTTATTCCTACTGAGGCCTATCCA
TCGTGGGTATCTTTCAACCAAAAGCAGAAGCTTAAATGGCTGAATCAAGAATTGGTGAAA
ATCTGGCCATTTGTCAATGAGGCTGCATCGGAACTGATAAAATCTTCTGTGGAGCCTGTA
TTCGAGCAGTACAAGTCATTCATTTTGGCCTCCATCCATTTCTCAAAGTTGACACTTGGT
ACTGTTGCTCCTCAGTTTACAGGAGTTCAAATTCTGGATAGCGACAGTGCTGGTATTACT
ATGGAGCTTGATATGCAGTGGGATGGTAATCCCAACATAGTACTTGACATTCAAACAACT
CTGGGAATTTCACTTCCTGTACAGGTGAAAAATATTGGATTCACAGGCACACTACGGCTA
CTCTTTAAGCCTCTGGTAGCCGAACTCCCATGCTTTGGAGCTGTTTGCGTTTCTTTGAGA
GAGAAGAGCAAGGTGGATTTTACCCTCAAAGTTGTTGGTGGCGAAATGACAGCAATTCCT
GGAATTTCTGATGCAATTGAGGGAACAATACGTGATACCATCGAGGACACACTGACATGG
CCTAATCGCATAATTGTTCCCATTGTGCCAGGAGATTATAGTGATCTGGAGCTAAAACCT
GTTGGATTATTAGAAGTAAAACTTGTGGAAGCTAGGGATTTGAAGAACAAGGACCCGTTG
GGGAAGTCTGACCCTTTTGCTGTGCTATACATACGTCCACTGAGTGCAAAAACGAAGAAA
AGCAAAACAATAAACAATGATTTGAACCCCATCTGGAATGAACACTATGAATTTGTGGTG
GAGGACTCATCTACCCAGCACCTGACTGTGAAAATTTACGACGACGAAGGGCTCCAGCCG
TCAGAGATTATTGGCTGCGCTCGAGTAGACTTATCGGATGTTACGCCTGGAAAGGTCAAG
GATGTTTGGTTGGAACTTGTGAAAGACCTGGAAATTCAGCGTGATAAGAAACCTCGTGGT
CAGGTCCACCTAGAGCTCCTGTACTACCCTTTTGAGAAACAAGAAGGGGTTTCCAATCCT
TTTGCTGGTCAGATCCAGTTAACTTCTTTGGAAAAGGTCCTCAAGACGGAATCTAATGGA
TATGATGTCAACCAGAGGAAAAATGTTATTACGAGAGGAGTCCTTTCAGTAACTGTTATA
TCTGCAGAGGACATACCAGCAATGGATGTGATGGGGAAGGCTGACCCATTTGTCGTCCTG
TACCTGAAGAAGGGGGAAACCAAAAAGAAGACAAGGGTTGTGACTGAGACGTTAAATCCA
ATATGGAATCAGACATTTGATTTTGTAGTAGAAGATGCCCTGCATGATTTGCTCATTGTG
GAAGTATGGGACCATGATACATTTGGAAAGGATTACATAGGGAGATGCATCTTGACACTT
ACCAGAGCGATACTCGAAGGCGAGTTCCAAGATACATATGTGCTGCAAGGTGCAAAATCT
GGAAAGCTGAACCTGCACTTCAAGTGGACGGCACAGCCAATCTACCGTGATCGGGACAGG
GACCAGTGA

Transcripts

cv773673
dr739188
cd921639
cd937635
dr735750
ca660786
cd875554
cd907808
cj701917
ck163560
bj278360
bj283413
bg607899 (Triticum monococcum)
cd905825
cj688815
cj717934
ca657942
bu672280
bu099749
cd910506
dr739985
cd907872
ck216259
ck163888
bj323040
bf203066
cd905826
ca602061
bg263096
cd907873
cd910507
cv778440
ca657233

Triticum aestivum fragment ***NTMC2Type2***

cj713921
cd874557
ca704120
ca654135

Triticum aestivum fragment ***NTMC2Type2***
aj604185
ca600524
ca607768

Triticum aestivum fragment ***NTMC2Type2***
cv770027

Triticum aestivum fragment ***NTMC2Type3***
bq237228
bq167837
be438286
bg604370
be438292
ck203643
dr735318
ca661706
ca746109
ca746366
ca746390
cf569205
cd861474
cf569201
bq240252
cd918999

Triticum aestivum Full Length ***NTMC2Type4***

MGLVVGVVIMAGWSRVMQRRSRKRVAKAADIKVLGSLGREDLKKLCGDNFPEWISFPQYE
QVKWLNKHLSKLWPFVSQAATAVVKESVEPLLDDYRPPGIKSLKFNKFSLGNVSPKIEGI
RIQNLQPGQIIMDIDFRWGGDPSIILAVDARVASLPIQLKDLQVFTVVRVVFQLSEEIPC
ISAVVVALLAEPEPKIQYTLKAVGGSLTAIPGLSDMIDDTVNSIVNDMLQWPHRLVVPLG
VNVDTSELELKPEGKLSVTVVKATSLKNKELIGKSDPYVTLYVRPMFKVKTKVIDDNLNP
EWNETFELIVEDKETQSVIFEVYDEDNLQQDKRLGVAKLAVNNIVPETPSEITLKLMQSV
DSLKIKDYRDRGSLHLKVMYHPFTKEEQLEALESEKKAIEERKRLKEAGVIGSTMDALGG
AASLVGSGVGFVGTGVAGGVGLVGSGLGAGAGLVGSGIGAVGSGLGKAGKFMGRTVTGHL
GMSRKSGSSSTVPQPDQPSA

ATGGGGTTGGTGGTCGGCGTCGTGATCATGGCCGGGTGGAGCCGCGTGATGCAGCGGCGC
AGCAGGAAGCGCGTCGCCAAGGCTGCGGATATCAAGGTCCTTGGGTCTCTTGGCAGGGAG
GACCTCAAGAAGCTCTGCGGCGACAATTTCCCCGAGTGGATATCCTTCCCACAGTATGAG
CAGGTGAAATGGCTGAACAAGCATCTCAGCAAACTTTGGCCTTTTGTTTCACAAGCTGCA
ACTGCAGTTGTTAAGGAATCTGTTGAACCCCTGCTAGATGATTATCGACCTCCAGGAATA
AAATCTCTGAAGTTCAACAAATTCTCTCTTGGAAATGTCTCGCCAAAGATCGAAGGCATC
CGTATCCAAAATCTTCAGCCAGGCCAAATCATAATGGATATAGATTTCCGTTGGGGTGGT
GATCCAAGCATAATCCTTGCTGTTGATGCTCGAGTTGCATCACTGCCTATTCAGCTCAAG
GATCTCCAGGTCTTTACCGTTGTTCGTGTCGTATTTCAACTATCAGAAGAGATTCCCTGC
ATCTCTGCTGTTGTCGTTGCTCTCCTCGCAGAGCCAGAGCCTAAAATACAGTACACATTG
AAGGCTGTTGGGGGTAGTCTGACTGCTATTCCAGGACTTTCTGATATGATTGATGATACT
GTCAATTCAATTGTCAATGACATGCTCCAGTGGCCACACAGGCTTGTTGTTCCACTTGGC
GTCAATGTTGATACAAGTGAGCTGGAGCTTAAACCTGAGGGAAAACTTTCTGTTACTGTA
GTAAAAGCAACATCTCTGAAGAATAAGGAGTTGATTGGTAAATCTGATCCCTACGTGACC
CTGTATGTGCGTCCCATGTTCAAGGTCAAAACAAAAGTCATAGATGACAACCTAAATCCT
GAATGGAATGAAACATTTGAACTGATTGTTGAAGACAAGGAAACCCAATCTGTCATTTTT
GAGGTTTATGATGAAGACAACCTTCAGCAAGACAAGAGGCTAGGTGTGGCTAAATTAGCA
GTGAATAATATTGTGCCCGAGACTCCCAGTGAAATCACTCTGAAGCTTATGCAGTCAGTA
GATTCACTTAAGATTAAAGACTACAGGGATAGAGGGTCATTACATCTTAAGGTCATGTAT
CATCCATTTACCAAGGAAGAACAACTGGAAGCCCTGGAGTCGGAAAAGAAAGCAATAGAG
GAGAGAAAGCGGCTGAAGGAGGCTGGGGTAATTGGCAGCACGATGGATGCACTTGGTGGC
GCCGCATCACTAGTTGGTTCAGGTGTTGGATTTGTGGGCACTGGCGTTGCCGGGGGTGTT
GGGCTCGTGGGCTCAGGGCTTGGTGCTGGCGCTGGTCTGGTTGGTTCTGGTATTGGTGCC
GTGGGCAGCGGCCTTGGTAAAGCTGGGAAGTTCATGGGGAGGACTGTGACAGGGCACTTG
GGCATGTCTCGCAAGAGTGGTAGCAGCTCCACTGTTCCCCAACCTGACCAGCCTTCTGCATAA

Transcripts

ck206031
bj281050
cn009970
ck206123
ca665599
ck211075
bj266962
cj674312
ca597378
cd889182
bj316243
bj315195
cv767891
cj692133
cd924763
be415662
be414862
al812415
bj305279
bj257773
ca597376
bg313350
cd877043
ck161950
bj263192
ca501951
ck194055
cd889183
bj320675
bj228637
bj321743
cd920165
bj311832
bj272073
ca610460
ck193713
bj485432
cd920166
bj311885
cd877044
bj311772
bj286112

Triticum aestivum fragment ***NTMC2Type5***
ca679251
cj543368

Triticum aestivum fragment ***NTMC2Type5***
bg905763
cj641478

Triticum aestivum fragment ***NTMC2Type5***
cd490947

Triticum aestivum fragment ***NTMC2Type5***
al809130

Triticum aestivum fragment ***NTMC2Type5***
cj533157

Triticum aestivum fragment ***NTMC2Type6***
al823034
cn011762
bq238885
bj312556
bj318972
bg605141
cd876310

Triticum aestivum fragment ***NTMC2Type6***
ca731730
cv771255
cd454573

bj277991

Triticum aestivum fragment ***NTMC2Type6***
bj283051
bj279751
ca645962
bj284735

Triticum aestivum fragment ***NTMC2Type6***
cd915786
cd881634
bj299187

Triticum aestivum fragment ***NTMC2Type6***
cv762577
bj292757

Triticum aestivum fragment ***NTMC2Type6***
bq235978
bj282403

Triticum aestivum fragment ***NTMC2Type6***
ca682766

Triticum aestivum fragment ***NTMC2Type6***
ck169955

Triticum aestivum fragment ***NTMC2Type6***
cv764844

Triticum aestivum fragment ***NTMC2Type6***

ca665734

Triticum aestivum fragment ***NTMC2Type6***
ca619905

Zea mays Full Length ***NTMC2Type1***

MGVISTVLGFSGFGFGFSAGIVIGYFLFIYVQPTDVKDVKVRPLVEYDSKSLEGILPEIP
LWVKNPDYDRIDWLNRFLELMWPYLNKAICRTAQDIAKPIIAENTAKYKIDSVEFESLTL
GSLPPTFQGMKVYVTEEQELIMEPSLKWAANPNITVAVKAYGLKATIQIVDLQVFASPRI
TLKPLVPTFPCFAKILVSLMEKPHVDFGLKLLGADVMAIPGLYRFVQETIKKQVASMYLW
PKTLEVPIMDPSKASKRPVGILLVKVVRAQNLRKKDLLGKSDPYVKLKMSDDKLPSKKTT
VKRSNLNPEWGEDFKFVVTDPENQALEVNVFDWEQVGKHEKMGMNMIPLRELLPEGTKVT
TLNLLKTMDPNDVQNEKSRGELTLELTYKPFKEEDIEKEDTQGADVIEKAPDGTPAGGGL
LYVVVHEAQDLEGKHHTNPYAKIIFKGEEKKTKVIKKNRDPRWEDEFEFVCEEPPVNDKL
HVEVISKAPKAGLIHGKETLGYIDISLADVISNKRINEKYHLIDSKNGQIQIELQWRTS

ATGGGTGTAATCAGCACAGTGCTGGGTTTCTCTGGTTTTGGCTTTGGGTTCTCGGCTGGCATTGTTATTG
GTTACTTCCTCTTCATCTACGTCCAGCCAACTGATGTCAAGGATGTCAAGGTTCGCCCGCTTGTAGAATA
TGATTCAAAATCCTTGGAGGGCATCCTTCCTGAAATTCCTTTATGGGTCAAGAATCCTGACTATGATAGA
ATTGATTGGCTGAATAGGTTCTTGGAGTTGATGTGGCCTTATCTTAACAAGGCTATCTGCAGAACTGCAC
AGGATATCGCGAAGCCAATTATTGCTGAGAACACTGCAAAGTATAAGATAGACTCTGTTGAGTTTGAATC
ACTTACTTTGGGTAGCTTACCACCCACCTTTCAAGGGATGAAAGTCTATGTCACAGAGGAGCAAGAGCTG
ATAATGGAACCATCTCTTAAATGGGCTGCAAACCCAAATATTACTGTTGCTGTAAAAGCCTATGGTTTGA
AAGCTACTATCCAGATTGTGGATCTACAAGTCTTTGCATCACCTCGCATTACTCTGAAGCCATTGGTGCC
TACATTTCCTTGCTTTGCAAAAATCCTTGTCTCACTCATGGAGAAGCCACATGTTGACTTTGGGCTAAAA
CTTCTTGGAGCAGATGTAATGGCTATTCCTGGTCTTTACAGATTTGTTCAGGAGACCATCAAGAAACAAG
TAGCAAGCATGTATTTGTGGCCGAAGACACTAGAAGTACCTATAATGGATCCCTCAAAAGCATCAAAAAG
GCCTGTTGGAATTCTACTTGTGAAGGTTGTGAGAGCCCAAAATCTGCGGAAGAAGGATCTGCTGGGTAAA
TCTGACCCATACGTGAAACTTAAGATGTCAGATGATAAACTTCCATCCAAGAAGACAACTGTAAAGCGCA
GCAATCTCAATCCAGAGTGGGGTGAAGACTTCAAGTTTGTTGTGACAGATCCAGAAAACCAGGCTCTTGA

AGTTAATGTCTTCGACTGGGAACAGGTTGGGAAACATGAAAAGATGGGAATGAACATGATCCCACTTAGA
GAACTCCTACCAGAGGGGACTAAAGTTACTACCCTCAACTTGCTTAAGACCATGGACCCAAACGATGTAC
AAAATGAGAAGTCTCGTGGTGAGCTTACTCTAGAGCTCACATACAAGCCTTTCAAGGAAGAAGATATAGA
GAAAGAAGATACACAGGGTGCTGATGTGATAGAGAAAGCTCCAGATGGCACTCCAGCTGGTGGTGGCTTG
CTTTATGTTGTTGTTCATGAAGCCCAAGATCTTGAGGGGAAGCACCATACAAACCCATATGCAAAAATAA
TTTTCAAAGGCGAGGAGAAGAAAACTAAGGTCATCAAGAAGAATAGGGATCCAAGATGGGAGGATGAGTT
TGAGTTCGTGTGTGAGGAGCCTCCTGTGAATGATAAACTGCATGTTGAAGTCATAAGTAAAGCCCCGAAG
GCAGGGCTGATACATGGCAAGGAAACTTTGGGCTACATTGATATTAGCCTTGCAGACGTGATCAGCAACA
AGCGGATTAATGAAAAGTACCATCTCATAGACTCGAAAAATGGTCAGATCCAGATCGAGTTGCAGTGGAG
AACTTCCTAG

Transcripts

dt941521
dt646632
dr807380
dy397094
dr820585
dt648378
ay105555
aw126451
dv942548
dr807379
cd963476
dn211466
cd996846
cf044903
bm500936
aw053165
ai987440
ai795653
cf637784
bm381181
ai600976
cf017778
cf631428
dr787273
cd955004
cd941523
aw126531
ai712058

Zea mays fragment ***NTMC2Type1***

dv028076
dr799485
dr805093
dr972872
dy538544
dv512037
dr823303
dr823302
dv028075
dr805092
dr799484
dv512036
dv494303
cf036361
ai677012
ay111680
aw520224

Zea mays fragment ***NTMC2Type1***

eb407376
bm895414
bm896341
bu051108
bm895413
ai649677
cd951694
cd940891

Zea mays fragment ***NTMC2Type1***

aw231542
cd949045
cd949386

cd949912

Zea mays fragment ***NTMC2Type1***
cf650049
ac185283

Zea mays fragment ***NTMC2Type1***
dr972871

Zea mays fragment ***NTMC2Type2***
ay108443
co455760
ca400768
dy620234
dn560087
cf052742
cf037474
cd966888
cd946481
cd964181
dn231588
dn223197
cf627238
cf630568
dv495521
dv493964
bm500549
aw171804
dn221012
cf626248
cf626168
cd973777
ca620945
ai738129
aw927961

Zea mays fragment ***NTMC2Type2***
cf919848
dn228073
bm660090
dn226781
dy536619
dt641462
cd945360
cd964603
cd966948
cf012066
cf011636
cf630166
cf012065

Zea mays fragment ***NTMC2Type2***
dt641463
aw067031

Zea mays fragment ***NTMC2Type2***
co459294

Zea mays fragment ***NTMC2Type2***
aw158019

Zea mays fragment ***NTMC2Type3***
ay107424
cf638007
dr796284
dr796283
cf629390
bg874228
cd219428
bm952569
aw171872
dv494148

Zea mays Full Length ***NTMC2Type4***

MGLISGMMMGVIVGVAIMAGWSRVMRRRSTKRIAKAADIKVLGSLSRDDLRKLCGDNFPE
WISFPQFEQVKWLNKHLSKLWPFVVEAATVVVKESVEPLLDDYRPPGIKSLKxSKFSLGN
VSPKIEGIRIQNLQPGQIIMDIDFRWGGNPSIILAVDAVVASLPIQLKDLQVYTVIRVIF
QLSEDIPCISAVGVALLADPEPKIQYTLEVIGGKLTAVPGLSDMIDDTVDSIVSDMLLWP
HRHVVKLGVNVDTSDLELKPQGRLSVTVVKATSLRNKEMIGKSDPYVKLYVRPMFKVKTK
VIDDDLNPEWNETFDLIVEDKETQSVIFEVYDEDKLQQDKRLGVAKLAVNTLESEITQDA
TLKLLHSLDPIKNKDTKDRGTLHLKVKYHPFTKEEQLEALEMEKQAIEERKRLKEAGIIG
STMDAVGSGVGFVGTGIGAGLGFVGSGIGAGAGLVTSGIGAVSSGLGKAGKFMGRTVTGP
FSMSRKNGSSSTAPQHDQPSA

ATGGGGTTGATATCCGGGATGATGATGGGGGTCATTGTTGGCGTCGCCATCATGGCCGGC
TGGAGCCGCGTCATGCGCCGACGCAGCACGAAGCGCATCGCCAAGGCTGCGGATATCAAG
GTGCTTGGGTCTCTCAGCAGGGACGACCTCAGGAAGCTGTGCGGTGATAACTTCCCGGAG
TGGATATCCTTCCCGCAGTTTGAGCAGGTTAAATGGTTGAACAAGCATCTGAGCAAACTT
TGGCCTTTTGTTGTAGAAGCTGCAACAGTAGTGGTTAAGGAATCCGTTGAACCACTGCTA
GATGATTACCGGCCTCCAGGAATAAAATCTTTGAAGnTCAGCAAATTTTCTCTTGGAAAT
GTGTCACCAAAGATAGAAGGnATTCGTATTCAAAATCTTCAACCAGGTCAAATCATCATG
GATATAGATTTCCGTTGGGGTGGTAATCCAAGCATAATCCTAGCTGTTGATGCTGTAGTT
GCATCACTGCCAATTCAGCTCAAGGATCTTCAGGTCTATACTGTCATACGTGTTATATTT
CAACTGTCTGAAGACATCCCTTGCATTTCTGCTGTTGGTGTGGCTCTCCTTGCTGATCCA
GAGCCAAAAATTCAGTACACCCTGGAGGTCATTGGAGGAAAGCTAACTGCAGTTCCTGGA
CTTTCTGACATGATTGATGACACTGTCGATTCAATTGTTTCTGATATGCTCTTGTGGCCA
CACAGGCATGTTGTTAAACTTGGTGTCAATGTTGATACGAGTGACTTGGAGCTTAAACCT
CAGGGAAGACTTTCTGTTACTGTGGTAAAAGCAACTTCTTTGAGAAATAAGGAGATGATC
GGTAAATCAGACCCTTATGTGAAACTGTATGTGCGGCCAATGTTTAAGGTCAAAACAAAA
GTCATAGATGATGACCTGAATCCGGAATGGAATGAAACATTTGACTTGATTGTCGAAGAC
AAAGAAACCCAATCTGTTATTTTTGAGGTTTACGACGAAGACAAACTTCAGCAAGACAAG
AGACTAGGTGTGGCAAAACTAGCAGTAAACACTCTTGAATCTGAGATTACCCAAGACGCC
ACTCTGAAGCTGCTACATTCACTGGATCCAATTAAAAATAAGGACACCAAGGATAGGGGC
ACATTACATCTTAAGGTAAAGTACCACCCCTTTACAAAGGAAGAGCAGCTGGAAGCCCTA
GAGATGGAAAAACAAGCCATAGAGGAGAGGAAGCGTCTGAAGGAGGCTGGGATAATTGGT
AGCACAATGGATGCTGTTGGTTCTGGTGTTGGATTTGTGGGTACTGGCATTGGCGCTGGC
CTCGGTTTCGTTGGTTCAGGAATTGGTGCTGGCGCAGGCCTTGTTACCTCTGGTATTGGT
GCTGTCAGCAGTGGCCTTGGTAAAGCCGGGAAGTTCATGGGCAGGACTGTGACAGGGCCT
TTCAGCATGTCCCGCAAGAACGGCAGCAGCTCTACTGCTCCCCAGCATGATCAACCTTCG
GCGTAG

Transcripts
dy233464
dv544157
dr802511
dt649051
co454695
ay105616
co440434
dr802510
cd955534
aw520000
dv494034
cf624255
aw308707
co443976
dn212847

Zea mays Full Length ***NTMC2Type5***

MLTPSAGIPPPSLSAASSPSTSYSLLYLPRRCRRLPVPRASSSFAPKRSAPVPTTTLPPE
APSTSAAVEAPAPFSVTAPGTYRGGEDPLVSKLRTQLGVIHPLPAPPISRSVVGLFALFF
FVGAAFDKLWTLRKRRRAERELKVNGSWPQVPTPSFSLFLEKDLQRKESVEWVNMVLGKL
WKVYRTGIENWIVGLLQPVIDNLHKPDYVNRVEIRQFYLGEEPLSVRNVERRTSRRANDL
QYQIGIRYAGGARMALALYLKFTKVPVVVPVWVRDFDIDGELWVKLRLIPTEPWVGAVSW
AFVSLPKVKFELSLFRLFNLMAIPVLSMFLTELLTEDLPRLFVRPKKIVLDFQKGRAMGP
VSGSVASDIIQNVATDLIQEGNKDFVGELSVTLVDARKLSFVLFGKTDPYVVMIIDDQVI
KSKKNSQTTVIGLPGEPIWNQDFHMLVANPRKQKLTIQVKDSIGLTDITIGTGELGSLKD

TVPTDKIVTLYGGWGLFGKREAGEVLLRLTYKAYVEDEEEEDEAVRSEIGAGYASDEDVL
DYVSGMSKGTDFVGKERETFMDLLAALLVSEEFQGIVSSETGSSSREGEQAGSGPEGTDS
VTVPTSAAAADTEASTVPNSSTDTALVWLAAITSVMVLVSSNLGGSGYFNP

ATGCTGACCCCCTCGGCCGGCATACCGCCGCCCTCCCTCTCCGCAGCTAGCAGCCCCTCC
ACGTCCTACTCTCTCCTCTACCTTCCCCGACGATGCCGACGCCTTCCCGTCCCCCGAGCC
TCCTCCAGCTTTGCCCCCAAGCGCTCGGCGCCGGTCCCCACCACCACCCTCCCGCCCGAG
GCCCCCTCCACCTCCGCCGCTGTCGAGGCGCCGGCGCCCTTCTCGGTCACGGCCCCGGGG
ACCTACCGGGGAGGGGAGGACCCGCTCGTCTCCAAGCTCCGCACCCAGCTCGGCGTCATC
CACCCGCTCCCGGCGCCCCCCATCAGCCGCTCCGTCGTCGGCCTCTTCGCCCTCTTCTTC
TTCGTCGGCGCCGCCTTCGACAAGCTCTGGACGCTGCGGAAGCGCCGTCGCGCCGAGCGC
GAGCTCAAGGTCAACGGCTCCTGGCCGCAGGTGCCCACCCCGAGCTTCTCCCTCTTCCTC
GAGAAGGACCTGCAGCGCAAGGAGTCCGTCGAGTGGGTCAACATGGTGCTCGGCAAGCTC
TGGAAGGTCTACCGCACCGGCATCGAGAACTGGATCGTCGGCCTGCTCCAGCCCGTCATC
GACAACCTCCACAAGCCCGATTACGTCAACAGGGTCGAAATCAGGCAGTTCTACCTCGGC
GAGGAGCCCCTCTCCGTCAGGAACGTCGAGCGCCGCACCTCAAGGCGGGCCAACGACCTG
CAGTACCAGATTGGCATCCGCTATGCCGGTGGAGCACGCATGGCATTGGCACTCTACTTG
AAGTTCACCAAAGTACCGGTCGTCGTGCCGGTCTGGGTTCGAGATTTTGATATCGATGGG
GAGTTGTGGGTTAAACTAAGGCTCATACCAACAGAACCGTGGGTGGGAGCCGTATCTTGG
GCTTTTGTGTCCCTTCCCAAGGTTAAGTTTGAGCTTTCGCTGTTCCGGCTATTCAATCTT
ATGGCGATTCCTGTTCTGTCTATGTTTCTCACAGAACTTCTGACAGAAGATTTGCCACGT
CTCTTTGTGCGGCCCAAAAAGATTGTCCTTGATTTTCAAAAGGGGAGAGCTATGGGACCT
GTTTCAGGAAGTGTTGCAAGTGATATAATTCAAAATGTTGCAACTGACCTGATCCAAGAG
GGGAATAAGGACTTTGTTGGAGAGCTATCAGTGACACTAGTTGATGCTAGAAAGCTTAGC
TTTGTCTTGTTTGGCAAGACAGATCCTTATGTTGTCATGATTATTGATGATCAAGTAATA
AAGAGCAAGAAGAACAGTCAAACAACTGTGATTGGACTACCGGGAGAACCAATTTGGAAT
CAAGATTTCCATATGCTTGTCGCAAATCCTCGCAAACAGAAGTTGACCATTCAAGTGAAG
GATTCAATTGGTCTAACTGACATCACTATTGGCACCGGAGAGCTAGGGTCACTTAAAGAT
ACAGTACCTACGGACAAAATCGTGACTCTATATGGAGGATGGGGTTTGTTTGGGAAGCGA
GAGGCTGGGGAAGTTCTACTTCGGCTGACATACAAGGCATATGTCGAGGATGAAGAGGAA
GAAGACGAGGCGGTGAGAAGCGAGATTGGTGCGGGTTACGCCTCAGACGAGGATGTGCTG
GACTACGTTAGTGGCATGAGCAAGGGGACTGATTTCGTGGGTAAAGAAAGGGAAACTTTC
ATGGATCTTCTCGCCGCGCTGCTGGTGAGCGAGGAGTTCCAAGGCATAGTGTCATCCGAA
ACAGGAAGCTCGTCAAGAGAAGGTGAGCAGGCAGGCAGTGGACCAGAAGGCACAGACAGC
GTCACTGTCCCTACTAGCGCTGCGGCGGCTGATACGGAGGCATCCACCGTGCCAAACAGC
TCCACAGATACGGCTCTGGTGTGGCTGGCGGCCATAACGAGTGTGATGGTGCTGGTGTCC
TCCAACCTCGGTGGCTCAGGCTACTTCAACCCGTGA

Transcripts
ac182834
ac185634
cd973253
cd972410
bg842160
ai612344

Zea mays fragment ***NTMC2Type5***
cd973253
cd972410
bg842160
ai612344

Zea mays fragment ***NTMC2Type6***
cd438017
dn234240
ca403860
dy541975

Zea mays fragment ***NTMC2Type6***
ck828032
ck369003

Zea mays fragment ***NTMC2Type6***
ck347265
cd951781

Zea mays fragment ***NTMC2Type6***
dy532655

Zea mays fragment ***NTMC2Type6***
dy541976

Zea mays fragment ***NTMC2Type6***
dn559870

Zingiber officinale fragment ***NTMC2Type4***
dy371591
dy371592

Zingiber officinale fragment ***NTMC2Type5***
dy381812

Zingiber officinale fragment ***NTMC2Type5***
dy381813

Zantedeschia aethiopica fragment ***NTMC2Type4***
aj701717

Aquilegia formosa x pubescens Full Length ***NTMC2Type1***

MGFFSTIFGLFGFGMGISIGLVAGYYLFIYFQPTDVKDPTIRPLVEQDSQTLQRILPEIP
LWVKNPDYDRVDWLNKFIELMWPYLDKAICKTAKNIAKPIIAEQIPKYKIDSVEFEVLTL
GSLPPTFQGMKVYVTDEKELIMEPCLKWAGNPNVTVAVKAFGLKATIQVVDLQVFAAPRI
TLKPLVPSFPCFAKILVSLMDKPHVDFGLKLLGADVMSIPGLYSFVQEFIKEQVANMYLW
PKTLEVVIMDPAKAQKRPVGILHVKVVRAIKLKKKDLIGASDPYVKLKLTEDKLPSKKTT
VKHKNLNPEWNEEFNFVVKDPGSQALEFTVYDWEQVGKHDKMGMNVIPLKDLIPEETKVM
THDLLKNMDPNDAQNEKSRGQLVLEVTYKPFKEEDIPKDIEDSNSVEKAPEGTPSTGGLL
VVMIHEAEDVEGKHHTNPYVRILFRGEERKTKHIKKNRDPRWEEEFTFMLEEPPTNDRMH
VEVISASSRMGILHPKETLGYIDINLADVVSNKRINEKYHLIDSKNGRIQIELQWRTGS

ATGGGGTTTTTCAGTACGATATTTGGTTTATTTGGATTTGGAATGGGGATCTCAATTGGT
CTTGTTGCTGGTTATTATCTCTTCATTTACTTTCAACCCACTGATGTTAAGGATCCCACA
ATTCGTCCACTGGTTGAGCAAGATTCACAAACCTTGCAGCGAATACTTCCAGAGATTCCT
CTTTGGGTGAAAAATCCAGACTATGATCGTGTTGATTGGCTAAACAAATTTATTGAGCTC
ATGTGGCCTTATCTGGACAAGGCGATTTGCAAGACTGCAAAGAACATTGCCAAGCCCATA
ATTGCGGAGCAAATTCCTAAATATAAAATAGATTCAGTTGAATTTGAAGTACTCACTTTA
GGCTCTCTACCTCCAACTTTTCAAGGCATGAAAGTCTACGTCACAGATGAGAAGGAGCTG
ATAATGGAACCTTGCTTGAAATGGGCAGGAAATCCTAATGTTACTGTTGCTGTGAAGGCA
TTTGGATTGAAAGCTACCATACAGGTTGTGGACTTGCAAGTATTTGCTGCACCACGTATC
ACTTTGAAGCCATTGGTTCCAAGTTTTCCTTGTTTTGCTAAAATTCTCGTCTCTCTCATG
GACAAGCCTCATGTTGACTTCGGACTCAAGCTTTTAGGAGCAGATGTTATGTCGATACCT
GGTTTGTACAGCTTTGTCCAGGAGTTTATCAAAGAACAAGTGGCAAATATGTACCTGTGG
CCCAAAACCCTGGAAGTAGTAATCATGGACCCTGCAAAAGCCCAAAAGAGGCCTGTTGGA
ATTCTTCATGTGAAAGTAGTTCGAGCTATAAAGCTAAAAAAGAAAGATCTTATTGGGGCA
TCTGACCCCTATGTGAAATTAAAGCTCACTGAGGACAAGCTACCGTCAAAAAAGACCACT
GTAAAACATAAGAACCTGAATCCTGAATGGAATGAGGAGTTCAACTTCGTTGTTAAAGAC
CCnGGTTCTCAAGCTCTTGAGTTCACTGTTTATGATTGGGAACAGGTTGGTAAGCATGAC
AAGATGGGTATGAACGTCATTCCTTTGAAAGATCTTATCCCCGAGGAGACAAAAGTCATG
ACTCATGATCTTTTGAAAAACATGGATCCCAATGATGCCCAGAATGAGAAGTCAAGGGGA
CAGCTTGTGTTGGAAGTGACATACAAGCCTTTCAAGGAGGAGGATATCCCTAAAGATATT
GAAGATTCAAATTCAGTAGAAAAGGCTCCTGAAGGGACACCTTCTACTGGAGGTTTGCTC
GTTGTTATGATTCATGAAGCTGAAGATGTTGAAGGGAAGCACCATACAAATCCATATGTG
CGGATACTTTTTAGAGGAGAGGAGAGGAAAACTAAGCATATAAAGAAAAACAGGGATCCA
AGATGGGAAGAGGAGTTTACTTTTATGCTGGAGGAACCACCAACAAATGATAGAATGCAT
GTAGAAGTCATTAGTGCTTCTTCAAGAATGGGCATTTTGCATCCTAAGGAAACCCTGGGT
TATATTGATATCAATCTGGCAGATGTTGTAAGCAACAAACGAATCAACGAGAAGTACCAT
TTGATCGATTCAAAGAATGGACGAATTCAGATTGAGTTGCAGTGGAGAACAGGGTCTTAA

Transcripts
dr942974
dr913587
dr942973
dr913586

Aquilegia formosa x pubescens fragment ***NTMC2Type2***
dt760327

Aquilegia formosa x pubescens fragment ***NTMC2Type*3**
dt765437
dt765232

Aquilegia formosa x pubescens fragment ***NTMC2Type3***
dt765436

Aquilegia formosa x pubescens Full Length ***NTMC2Type4***

MGLISGMLMGMVVGIALMAGWQYMMRYRSTKRISKAVDIKLLGSLSRDDLKKLCGDNYPE
WISFPMFEQVKWMNKQLSKLWPFIADAATIIIRESVEPLLEEYRPPGITSLKFSKLSLGN
VAPKIEGIRVQSLQKGQITMDIDFRWGGDPSIILAVEAALVASIPIQLKDLQVFTVVRVI
FQLTDEIPCISAVVVALLSEPKPRIDYTLKAVGGSLTALPGISDMIDDTVNTIVTDMLQW
PHRIVVPIGGIPVDTSELELKPQGKLSVTVAKANDLKNMEMLGKSDPYAVVYIRPLFKVK
TKVINNNLNPVWNEVFELIAEDKETQSLVLEVFDKDVAGQDKRLGIAKLPLINVEPDAPQ
EIDLRLLPSLDMMKIKDKKDRGTITIKVLYHQFNKEEQLAALEEEKKLLEERKKMKEAGM
IGSTMDALDGAASFVGSGVGMVGTGLGAGVGIVGSGLGAVGSGLSKAGRFMGRSITGQSS
GPKKSGTSTPVGSQENGGAKQA

ATGGGTTTGATATCGGGAATGTTAATGGGAATGGTTGTTGGAATTGCTTTGATGGCTGGT
TGGCAATACATGATGCGTTATCGAAGCACTAAACGTATCTCTAAGGCAGTTGACATAAAA
CTTCTTGGTTCCCTTAGCCGAGATGATTTGAAGAAACTTTGTGGTGACAATTATCCTGAA
TGGATATCTTTCCCTATGTTTGAACAGGTGAAATGGATGAACAAGCAACTGAGCAAACTG
TGGCCTTTTATTGCGGATGCAGCAACAATAATCATAAGAGAATCAGTTGAACCATTGTTG
GAAGAATACCGGCCTCCAGGAATTACTTCCCTAAAATTCAGTAAACTATCTCTTGGAAAC
GTTGCTCCCAAGATTGAAGGAATTCGTGTTCAGAGCCTTCAAAAAGGCCAAATTACTATG
GATATTGACTTTAGATGGGGTGGTGATCCAAGCATCATTCTCGCGGTAGAAGCTGCCCTT
GTTGCTTCAATACCCATTCAGTTGAAGGATCTTCAGGTTTTTACTGTGGTGCGTGTCATT
TTTCAACTGACTGATGAGATTCCATGCATTTCAGCTGTTGTTGTTGCTCTGTTGTCTGAG
CCGAAGCCTAGGATTGACTATACATTGAAGGCTGTTGGAGGTAGTCTAACGGCACTTCCT
GGGATTTCAGATATGATCGATGATACTGTGAATACAATCGTCACCGATATGCTCCAGTGG
CCACATAGAATTGTTGTTCCAATTGGTGGTATACCTGTGGATACAAGTGAATTGGAGCTT
AAACCACAGGGGAAGCTTTCAGTCACAGTGGCAAAAGCTAATGATTTGAAAAATATGGAA
ATGCTTGGTAAATCGGATCCTTATGCTGTTGTATATATCCGTCCTCTCTTCAAGGTTAAG
ACGAAGGTCATCAACAACAATCTGAATCCTGTTTGGAATGAAGTTTTTGAGTTGATAGCT
GAAGACAAGGAAACTCAATCACTTGTCCTTGAGGTTTTCGACAAGGATGTTGCAGGCCAG
GACAAGCGACTGGGAATAGCTAAATTACCTTTGATTAATGTAGAACCTGATGCTCCACAG
GAGATAGATCTGAGGCTGCTACCATCACTTGACATGATGAAAATTAAAGATAAGAAAGAT
AGAGGAACCATCACTATCAAGGTATTGTATCATCAGTTTAACAAGGAAGAGCAACTGGCA
GCCCTGGAAGAAGAAAAGAAGCTACTTGAAGAGAGGAAAAAGATGAAAGAAGCAGGAATG
ATTGGAAGCACGATGGATGCACTTGATGGGGCAGCATCCTTCGTTGGTTCAGGTGTTGGG
ATGGTAGGCACTGGTTTAGGTGCAGGAGTTGGAATAGTTGGTAGTGGTCTTGGTGCGGTT
GGCAGCGGATTGAGCAAGGCAGGGAGGTTTATGGGTAGGAGCATTACAGGACAATCAAGT
GGCCCCAAGAAGAGTGGCACCAGCACACCAGTTGGTTCTCAAGAAAATGGAGGTGCAAAA
CAAGCTTAG

Transcripts
dt745060
dr926150
dr925253
dt758398
dt733730
dt731385
dt768431
dr929699

dt730480
dt753246
dr926149
dt745059
dt758397
dt730479
dt768430
dr925252
dr929698
dt753245

Aquilegia formosa x pubescens fragment ***NTMC2Type4***
dt760328
dt755599

Aquilegia formosa x pubescens fragment ***NTMC2Type5***
dr951217
dt733147
dt733146
dt733013
dr951216

Aquilegia formosa x pubescens fragment ***NTMC2Type6***
dt755693

Eschscholzia californica fragment ***NTMC2Type1***
ck745808
cd480757
ck753562

Beta vulgaris fragment ***NTMC2Type1***
bq590781

Beta vulgaris fragment ***NTMC2Type1***
bq591021

Beta vulgaris fragment ***NTMC2Type1***
bq594469

Beta vulgaris fragment ***NTMC2Type2***
bq588498

Beta vulgaris fragment ***NTMC2Type3***
bq589003

Beta vulgaris fragment ***NTMC2Type3***
bq594707

Beta vulgaris fragment ***NTMC2Type4***
cf542678
bq589017

Beta vulgaris fragment ***NTMC2Type5***
bq584858
bq584687

Beta vulgaris fragment ***NTMC2Type6***
cf543566
cf543565
bq591921

Beta vulgaris fragment ***NTMC2Type6***
bq585043

Mesembryanthemum crystallinum fragment ***NTMC2Type1***
ca837747
dy034021

Mesembryanthemum crystallinum fragment ***NTMC2Type1***
ca832671

Mesembryanthemum crystallinum fragment ***NTMC2Type4***
be131272
bf479559

Ribes americanum fragment ***NTMC2Type6***
dt601708

Antirrhinum majus fragment ***NTMC2Type1***
aj800055

Antirrhinum majus fragment ***NTMC2Type3***
aj801434

Apium graveolens fragment ***NTMC2Type1***
cn254310

Capsicum annuum fragment ***NTMC2Type1***
bm062732

Capsicum annuum fragment ***NTMC2Type1***
ca847416

Capsicum annuum fragment ***NTMC2Type1***
ca520365

Capsicum annuum fragment ***NTMC2Type2***
co912318

Capsicum annuum fragment ***NTMC2Type4***
ca517186
co776820
ca517527

Capsicum annuum fragment ***NTMC2Type4***
co909155

Coffea canephora fragment ***NTMC2Type1***
dv702785
dv701356
dv695897
dv686021

Coffea canephora fragment ***NTMC2Type1***
dv687026
dv685235

Coffea canephora fragment ***NTMC2Type1***
dv691674

Coffea canephora fragment ***NTMC2Type4***
dv685397
dv712730

Gerbera hybrid fragment ***NTMC2Type1***

aj761030

Gerbera hybrid fragment ***NTMC2Type1***
aj760948

Gerbera hybrid fragment ***NTMC2Type1***
aj758503

Gerbera hybrid fragment ***NTMC2Type1***
aj762639

Gerbera hybrid fragment ***NTMC2Type2***
aj757335

Gerbera hybrid fragment ***NTMC2Type4***
aj753500
aj757800

Hedyotis terminalis fragment ***NTMC2Type1***
cb079040
cb077782

Hedyotis terminalis fragment ***NTMC2Type5***
cb076375

Helianthus annuus fragment ***NTMC2Type1***
dy907148
dy907176
dy935946 (Helianthus petiolaris)
dy904526
dy907355
dy904334
dy907371
dy932214 (Helianthus petiolaris)
cf080724 (Helianthus paradoxus)

Helianthus annuus fragment ***NTMC2Type1***
dy907025
dy909431
dy907039

Helianthus annuus fragment ***NTMC2Type1***
cd853971
dy908800
dy920443

Helianthus annuus fragment ***NTMC2Type1***
bu024919
cf084861 (Helianthus paradoxus)
cf080419 (Helianthus paradoxus)

Helianthus annuus fragment ***NTMC2Type1***
bu016203

Helianthus annuus fragment ***NTMC2Type1***
bq910657

Helianthus paradoxus fragment ***NTMC2Type1***
cf080724

Helianthus paradoxus fragment ***NTMC2Type1***
cf078762
cf096609 (Helianthus argophyllus)

Helianthus petiolaris fragment ***NTMC2Type1***
dy957255

Helianthus annuus fragment ***NTMC2Type2***
cx944153
cf084616 (Helianthus paradoxus)

Helianthus annuus fragment ***NTMC2Type2***
dy908401
dy908781

Helianthus annuus fragment ***NTMC2Type3***
bq971985
bq967919

Helianthus petiolaris fragment ***NTMC2Type4***
dy941307
dy932294

Helianthus argophyllus fragment ***NTMC2Type5***
cf087359

Helianthus annuus fragment ***NTMC2Type6***
bu026807
bu026769
bu027054
bq915494
bu026902
bu023455
bu025212
bq967664
bu024607
bu026171
bu022595
bq969225

Helianthus annuus fragment ***NTMC2Type6***
bq970512

Ipomoea nil fragment ***NTMC2Type1***
cj746242
cj769929
cj770593
cj764607
cj766204
cj766731

Ipomoea nil fragment ***NTMC2Type1***
cj747845
cj748621
cj749500

Ipomoea nil fragment ***NTMC2Type1***
cj737415
cj751830

Ipomoea nil fragment ***NTMC2Type1***
cj756021
cj768417

Ipomoea trifida fragment ***NTMC2Type1***
au224000
au224205

Ipomoea batatas fragment ***NTMC2Type1***
dv037364

Ipomoea nil fragment ***NTMC2Type2***
bj560565
cj754925

Ipomoea nil fragment ***NTMC2Type2***
bj575832
cj772303

Ipomoea nil fragment ***NTMC2Type2***
bj555590

Ipomoea nil fragment ***NTMC2Type3***
cj747995

Ipomoea nil fragment ***NTMC2Type3***
cj770073

Ipomoea nil fragment ***NTMC2Type4***
bj555663
cj746127
cj749947

Ipomoea nil fragment ***NTMC2Type4***
bj571510
cj767165
cj764499

Ipomoea nil fragment ***NTMC2Type4***
bj570990
cj764831

Ipomoea nil fragment ***NTMC2Type4***
bj555115

Ipomoea nil fragment ***NTMC2Type4***
bj554086

Ipomoea nil fragment ***NTMC2Type5***
cj772678
cj770676

Ipomoea nil fragment ***NTMC2Type5***
cj748966
cj755309

Ipomoea nil fragment ***NTMC2Type6***
bj556907

Ipomoea nil fragment ***NTMC2Type6***
bj572451

Lactuca sativa Full Length ***NTMC2Type1***

MGVVSTIMGAFGFGIGVPTGLVIGYYLFIYYQPTHVETPKVRPLVERDTKSLEQMLPEIP
MWVKNPDHDRVDWLNKFIELMWPYLDKAICKTVKTIAEPIIKEQIPKYKIDAVEFDTLTL
GNLPPTFQGMKVYSTDDKELIMEPSFKWAANPNIHVAVKAFGLRPTIQVVDLQVFASPRI
TLKPLVPSFPCFCQILVSLMEKPHVDFGLKLLGADLMSIPGLYRFVQELIKTQVANMYLW
PKTLVVPVLDPAKAMKRPVGMLNVKVLRAMKLKKKDILGASDPYVKLKLTEDKLPSKKTV
VKHKNLNPEWNEEFHLVVKDPESQALEIIVYDWEQVGKHDKMGMNVIPLKEITPGEPKVM

TLELLKNMDPNDTQNEKSRGQIMIELVYKPFTDDQIPAESKNGEVIEKAPEGTPEGGGLL
VVIIHQAEDFEGKHHTNPFVRMLFRGEEKRTKPVKKNRDPRWDEEFSFSLEEPPTNDRMH
FEVVSTFSRMGLIHPKETLGYVDIQLGDVVSNKRINGKYNLIDSRNGKLQVELQWRTSS

ATGGGTGTCGTTAGCACAATTATGGGGGCATTTGGTTTTGGAATTGGAGTTCCAACTGGG
CTTGTGATTGGGTACTACTTGTTCATTTACTACCAACCAACTCATGTTGAGACACCAAAA
GTACGACCGTTAGTTGAACGAGATACAAAGTCCCTCGAACAGATGCTTCCGGAAATACCA
ATGTGGGTCAAAAATCCTGACCATGACCGGGTTGATTGGCTCAACAAGTTTATCGAGCTA
ATGTGGCCATATCTAGACAAGGCGATTTGCAAGACTGTGAAGACTATAGCAGAACCGATC
ATAAAAGAGCAGATTCCAAAGTACAAAATAGACGCAGTCGAATTTGATACACTTACATTA
GGCAACCTACCACCCACATTTCAAGGAATGAAAGTTTATTCTACGGATGACAAGGAGTTA
ATAATGGAACCATCATTTAAATGGGCTGCTAATCCCAACATTCATGTTGCTGTTAAGGCC
TTCGGGTTGAGACCCACTATTCAGGTGGTGGACTTGCAAGTATTCGCTTCACCTCGTATC
ACCTTAAAGCCATTAGTTCCGAGCTTCCCTTGCTTCTGTCAAATTCTCGTTTCTCTCATG
GAGAAGCCTCACGTTGATTTCGGACTAAAACTTCTCGGAGCCGATTTGATGTCCATCCCT
GGCTTGTACAGATTCGTTCAGGAACTTATCAAAACCCAGGTCGCAAACATGTATTTGTGG
CCTAAAACACTCGTCGTACCAGTACTCGATCCTGCAAAAGCGATGAAGAGGCCAGTTGGA
ATGCTGAATGTAAAGGTTTTGAGGGCGATGAAACTGAAAAAGAAGGATATTTTGGGTGCT
TCGGACCCCTATGTAAAATTGAAGCTCACCGAAGATAAGCTTCCCTCCAAGAAAACCGTT
GTCAAACACAAAAACCTTAATCCGGAATGGAATGAGGAGTTCCATTTAGTTGTGAAAGAT
CCGGAATCACAAGCTCTTGAGATCATCGTATATGACTGGGAACAGGTGGGGAAACATGAC
AAGATGGGGATGAATGTCATACCACTAAAAGAAATAACACCGGGAGAACCAAAAGTTATG
ACTCTTGAGCTTCTAAAAAACATGGACCCAAACGACACACAAAACGAAAAATCTCGAGGT
CAAATCATGATTGAATTGGTGTATAAGCCTTTCACAGACGATCAAATCCCGGCTGAAAGT
aaaaatggAGAAGTGATCGAGAAGGCTCCAGAAGGAACCCCAGAAGGTGGAGGTTTGCTT
GTTGTTATAATCCATCAAGCCGAAGATTTTGAAGGCAAACATCATACCAATCCATTTGTT
CGTATGCTTTTTAGAGGAGAAGAAAAAAGAACGAAGCCTGTGAAGAAAAACCGTGATCCG
AGATGGGATGAGGAGTTTTCATTTTCTTTAGAAGAGCCCCCAACAAACGACAGGATGCAT
TTTGAAGTCGTGAGTACTTTTTCAAGGATGGGTTTAATTCATCCCAAGGAAACATTGGGT
TATGTGGATATACAACTTGGGGATGTGGTGAGCAACAAGAGAATCAATGGAAAGTACAAC
TTAATTGATTCGAGGAATGGTAAGCTTCAAGTGGAGTTGCAATGGAGAACATCATCTTAA

Transcripts

dw162043 (Lactuca virosa)
dw158418 (Lactuca virosa)
bu005785 (Lactuca serriola)
dw083234 (Lactuca perennis)
dw155682 (Lactuca virosa)
dw169368 (Lactuca virosa)
dy967065
dw136475
dw107030 (Lactuca serriola)
dy971371
dw136126
dw142033
dy969596
dy963622
bq855709
dw076989
bu001190
bq986144

Lactuca sativa fragment ***NTMC2Type1***

dy971948

Lactuca sativa fragment ***NTMC2Type1***

bq867479

Lactuca saligna fragment ***NTMC2Type1***

dw110015 (Lactuca serriola)
dw062938
dw048632
dw062981
dw057146
dw056871
dw162653 (Lactuca virosa)
dw161891 (Lactuca virosa)
bq868190 (Lactuca sativa)

dw127788 (Lactuca sativa)
dy962394 (Lactuca sativa)
dw056480

Lactuca saligna fragment ***NTMC2Type1***
dw061473
dw060991
dw060709

Lactuca saligna fragment ***NTMC2Type1***
dw060065

Lactuca virosa fragment ***NTMC2Type1***
dw164579
dw165391

Lactuca sativa Full Length ***NTMC2Type2***

MSFIAGLITGLVVGIALIVLFVRSENARSMRRTALATTIAAFARMTVEDSKKLLSPEFYP
SWVVFSQRQKLTWLNAHLTKIWPYVDEAASELIKANLEPTLEQYRPMVLSSLSFSKFTLG
TVAPQFTGVSIVEDGGEGITMELEMNWDGNPSIILDIKTRLGVGLPVQVKNIAFTGVFRL
IFKPLVPEFPCFGAVSFSLRQKKKMDFTLKVVGGDISAIPGVADALESTIRDAVEDSITW
PVRKVIPILAGDYSDLELKPVGTLEVKLVQANGLTNKDIIGKSDPFAELYIRPLRNTTET
SKVINNDLNPIWNEHFEFVVEDTSTQHLIVKIFDDEGLQAAELLGCCHVKLSELVPGKVK
DIWIKLVKDLDLQRDNKDRGKVHLELLYCPYGMENGFTNPFTSNYTMTSLEKVLKSGDNE
NGDFVNKKRTVIIRGVLSVTVISAEDLPAVDLMGKADPFVVLTMKKTGMKNSTRVVNENL
NPVWNQTFDFVVEDGLHDMPVVEVYDHDTFGKDYIGRCILTLTRVILEGEYKECFQLEGA
KSGRLHLNLKWMAQPLYRDS

ATGTCGTTCATTGCGGGACTGATAACCGGACTTGTGGTGGGGATAGCACTGATTGTTCTG
TTTGTTCGGTCTGAGAATGCACGATCTATGCGTCGCACTGCACTTGCAACAACGATTGCG
GCTTTTGCGAGAATGACAGTTGAAGATTCAAAAAAGTTGCTTTCACCAGAATTCTACCCT
TCTTGGGTTGTTTTCTCACAGAGACAGAAGTTGACCTGGCTTAATGCTCATCTTACCAAG
ATCTGGCCCTATGTTGATGAGGCAGCATCTGAGCTTATAAAGGCTAATTTGGAGCCAACT
CTTGAACAATATAGACCAATGGTGTTGTCCTCTTTGTCATTTTCCAAGTTTACCCTCGGC
ACAGTGGCCCCACAGTTTACAGGAGTTTCTATTGTTGAAGATGGAGGTGAAGGAATAACT
ATGGAGTTGGAAATGAATTGGGATGGAAATCCTAGTATAATACTTGATATCAAGACCAGA
CTAGGAGTTGGATTGCCTGTGCAGGTGAAGAATATTGCATTCACTGGGGTTTTCAGGTTG
ATTTTTAAGCCTCTAGTTCCCGAATTTCCATGTTTTGGAGCTGTGTCATTTTCTTTGAGA
CAAAAAAAAAAGATGGATTTTACACTTAAAGTAGTTGGTGGTGACATTTCAGCTATTCCT
GGCGTTGCTGATGCCCTTGAGAGTACAATACGTGATGCTGTTGAAGACTCAATCACGTGG
CCAGTTCGAAAAGTTATTCCCATTTTGGCCGGGGATTACAGTGATCTTGAACTAAAGCCT
GTGGGAACATTGGAGGTGAAACTTGTTCAAGCTAATGGTTTGACAAATAAAGACATTATT
GGGAAATCTGATCCTTTTGCTGAATTATACATACGCCCTTTGCGTAATACAACCGAAACT
AGTAAAGTTATTAACAATGATTTGAATCCAATTTGGAATGAACATTTTGAGTTTGTAGTG
GAAGATACTTCCACACAACATTTAATAGTGAAAATATTCGATGATGAAGGGCTTCAAGCA
GCTGAGCTTCTTGGATGCTGTCATGTGAAATTAAGTGAACTTGTGCCTGGTAAAGTCAAG
GATATATGGATAAAATTGGTAAAAGATTTGGATCTTCAAAGAGACAATAAAGACAGGGGA
AAGGTGCACTTGGAGCTATTGTATTGCCCATATGGAATGGAAAACGGGTTCACAAATCCT
TTTACTTCTAATTACACAATGACATCTCTTGAAAAAGTTCTTAAAAGCGGAGATAACGAA
AATGGCGATTTCGTAAATAAAAAAAGAACCGTAATTATAAGAGGTGTCCTTTCTGTCACT
GTAATATCCGCCGAAGACTTGCCAGCTGTCGATTTAATGGGAAAGGCGGATCCTTTTGTC
GTGCTCACTATGAAGAAAACCGGGATGAAAAACAGCACAAGGGTTGTGAACGAGAATTTG
AACCCGGTTTGGAATCAGACTTTTGACTTTGTTGTTGAGGATGGGTTGCATGATATGCCT
GTTGTTGAAGTATATGATCATGATACATTTGGCAAAGACTACATTGGAAGATGCATTTTG
ACACTAACAAGGGTAATATTGGAAGGAGAATACAAAGAGTGTTTTCAACTTGAAGGGGCT
AAATCAGGAAGATTACATTTGAACCTCAAGTGGATGGCTCAACCACTTTATCGTGATTCTTAG

Transcripts
dy973834
dw159691 (Lactuca virosa)
dw132384
dw138888
dw140097
dy968948

dw058219 (Lactuca saligna)
bq864443
dw108856 (Lactuca serriola)
dw108858 (Lactuca serriola)
dw108857 (Lactuca serriola)
bq991992 (Lactuca serriola)
bu013673 (Lactuca serriola)

Lactuca sativa fragment ***NTMC2Type2***
dw132678
dw139532
bq876056

Lactuca sativa fragment ***NTMC2Type2***
dy968503

Lactuca sativa fragment ***NTMC2Type2***
bq857810

Lactuca virosa fragment ***NTMC2Type2***
dw164369
dw167901
dw170473

Lactuca saligna fragment ***NTMC2Type2***
dw069130
dw069198

Lactuca virosa fragment ***NTMC2Type2***
dw150824

Lactuca virosa fragment ***NTMC2Type2***
dw151073

Lactuca sativa fragment ***NTMC2Type3***
bq860380
dw092639 (Lactuca perennis)
dw170114 (Lactuca virosa)
dw136633
bq857234
bq844118

Lactuca serriola fragment ***NTMC2Type3***
dw147068 (Lactuca virosa)
dw146691 (Lactuca virosa)
dw109733
dw109735
dw109734
bu001779

Lactuca sativa fragment ***NTMC2Type4***
bq864522
bq845995
bq843811

Lactuca serriola fragment ***NTMC2Type4***
dw115275
dw117625

Lactuca sativa fragment ***NTMC2Type5***
dy982440

Lactuca saligna fragment ***NTMC2Type5***
dw045846

Lactuca sativa fragment ***NTMC2Type6***
dw151965 (Lactuca virosa)
dw167362 (Lactuca virosa)
bu004466 (Lactuca serriola)
dw149702 (Lactuca virosa)
dw149729 (Lactuca virosa)
bq868633
bq859593
bq875437
bq856510
bq851844
bq872618
bq875762
bq855311
bq852728
bq871968
bq872875
bq871242

Lactuca serriola fragment ***NTMC2Type6***
bq995706

Lycopersicon esculentum Full Length ***NTMC2Type1***

MGFVSTILGFCGFGVGVSCGLTIGYYLFIYFQPCDVKDPVIRPLVERDSKSLQQLLSEIP
LWVKCPDYDRVDWLNKFIEYMWPYLDKAICRTAKDIAAPIIAEQIPKYKIDSVEFETLTL
GSLPPTFQGMKVYVTEEKELIMEPSIKWAGNPNVTVAVKAFGLKATVQVVDLQVFAAPRI
TLKPLVPSFPCFANIFVSLMEKPHVDFGLKLLGADLMSIPGLYRFVQETIKDQVANMYLW
PKSLEVQILDPSKAMKKPVGVLHVKILRAMNLKKKDLLGASDPYVKLKLTESKLPSKKTP
VRHKNLNPEWNEEFNMVVKDPESQALELSVYDWEKIGKHDKMGMNVIPLKDLTPDETKTM
TLSLLKNMDANDSQNDKDRGQIMVELTYKPFKEDELPKDFEDNDAAHKVPEGTPPGGGVL
MIIVHEAQDVEGKHHTNPYVKILFKGEERKTKQVKKNRDPRWEEEFTFVLEEPPVNDRVH
MEVVSTSTRIGLLHPKETLGYVDINLSDVVSNKRINEKYHLIDSKNGRLQVELQWRTAS

ATGGGTTTTGTGAGTACGATTTTGGGTTTTTGTGGATTTGGAGTTGGGGTTTCATGTGGA
TTGACGATCGGTTACTATCTCTTCATCTATTTCCAACCCTGTGATGTTAAGGACCCTGTT
ATTCGTCCATTGGTTGAGCGAGATTCTAAAAGCTTGCAGCAGTTGCTGTCTGAAATTCCT
CTCTGGGTCAAATGTCCAGATTATGACCGTGTGGACTGGCTCAACAAATTTATCGAGTAT
ATGTGGCCTTACCTGGACAAGGCAATTTGCAGGACTGCAAAAGATATTGCGGCACCAATT
ATTGCTGAACAAATACCGAAGTATAAAATTGATTCTGTTGAATTTGAGACACTAACTTTG
GGGTCCTTACCCCCTACTTTCCAGGGGATGAAGGTCTATGTTACTGAAGAAAAAGAATTG
ATCATGGAACCATCAATAAAGTGGGCTGGAAATCCTAATGTTACCGTTGCGGTCAAAGCA
TTCGGATTGAAAGCAACTGTTCAGGTTGTGGACTTGCAGGTTTTTGCAGCTCCACGTATT
ACTCTGAAGCCTCTGGTTCCAAGCTTTCCATGTTTTGCCAATATCTTTGTGTCCCTCATG
GAAAAGCCACATGTTGACTTTGGATTGAAGCTTTTGGGGGCTGATCTTATGTCCATCCCT
GGCTTGTACAGGTTTGTCCAGGAGACTATTAAAGATCAGGTTGCTAACATGTATCTTTGG
CCTAAATCTCTAGAAGTGCAGATATTGGATCCATCCAAAGCAATGAAAAAACCTGTTGGG
GTCCTGCATGTGAAGATTCTGAGGGCTATGAATCTGAAAAAAAAAGATCTACTGGGTGCA
TCTGATCCTTACGTAAAACTTAAGCTCACTGAGTCAAAACTTCCTTCAAAGAAAACCCCT
GTAAGGCATAAGAATTTAAACCCAGAGTGGAATGAGGAATTTAATATGGTTGTTAAAGAT
CCAGAATCGCAAGCATTGGAGCTCTCTGTTTATGATTGGGAGAAGATTGGCAAGCATGAT
AAGATGGGCATGAACGTTATTCCACTGAAAGATTTGACTCCTGATGAAACAAAAACGATG
ACACTGAGTCTCCTAAAGAACATGGATGCGAATGATTCTCAAAATGATAAGGACCGTGGT
CAGATCATGGTGGAATTAACCTACAAACCATTTAAGGAGGATGAGTTGCCAAAAGATTTT
GAAGATAATGATGCAGCACATAAGGTTCCAGAAGGAACACCACCAGGGGGAGGCGTCCTT
ATGATTATTGTCCACGAAGCTCAAGATGTTGAAGGAAAGCACCATACTAATCCATATGTC
AAGATTCTTTTCAAAGGAGAGGAGAGAAAAACTAAGCAAGTCAAGAAGAACAGGGATCCA
AGATGGGAAGAGGAGTTTACTTTTGTGTTGGAGGAGCCTCCTGTGAATGATAGGGTGCAT
ATGGAAGTTGTCAGCACCTCTACAAGGATTGGCCTATTGCATCCTAAGGAGACATTGGGT
TATGTTGATATAAATCTTTCCGATGTTGTTAGCAACAAAAGGATCAATGAGAAGTACCAC
CTCATTGATTCAAAGAACGGTCGCCTTCAAGTCGAGCTGCAATGGCGAACCGCATCTTGA

Transcripts
aw217064
bt013766
bg131206
bi931710
bg127538

aw931283
be434405
aw216492
aw032323
bf051185
cn385288
bw690732
aw218695
bg132829
be432105
bp877898
aw621856
ai485746
bg127927

Lycopersicon esculentum fragment ***NTMC2Type1***
aw032617
bf050333

Lycopersicon esculentum fragment ***NTMC2Type1***
be354352

Lycopersicon esculentum Full Length ***NTMC2Type2***

MGVVIGVLVGVGLIVGFVKSENYRSKCRSELATTIAAFARMTVEDSRKIFTPEQYPPWVV
FSNQQKLNWLNSHLEKIWPFVDEAASELVRSSVEPILEQYRPMILASLKFSKFTLGTVAP
QFTGISILEGGSEGITMELEMNRDGNPSIILDIMTYLGVGLPVQVKNIGFTGIFRLIFRP
LVDEFPCFGAVCYSLRKKKKLDFTLKVVGGDMTAIPGISDAIEGTIRDAIEDSIIWPVRK
IIPILPGDYSDLELKPTGVLEVKLVQAKELTNKDIIGKSDPFAELYVRPVRDRMKKSKTI
NNELNPIWNEHFEFVVEDPLTQHLVIKIYDDEGLQAAELIGCAHVRLNELEPGKVKDVWL
KLVKDLEIQRDQKNRGQVHLELLYCPYGMTNGFSNPFANNVPLTSLEKVLKSGVEAAQNG
GEINRRKDVIVRGVLSVTVISAEDLAPTDLLGKADPYVVVTMKKTETKNKTRVVPESLNP
VWNQTFDFVVEDGLHDMLILEVWDHDTFGKDYMGRCILTLTRVLMEGEYKETFELDGAKS
GKLNLHLKWAPQPIYRDS

ATGGGTGTTGTGATTGGGGTTTTAGTTGGAGTTGGTTTGATAGTTGGCTTTGTGAAATCT
GAAAATTATCGATCCAAATGCCGTTCTGAACTGGCTACTACAATTGCGGCATTTGCTAGA
ATGACAGTGGAAGATTCCAGAAAGATTTTTACACCAGAGCAGTATCCTCCTTGGGTTGTT
TTCTCCAATCAGCAGAAGTTGAATTGGCTTAATTCTCATCTTGAAAAGATCTGGCCTTTT
GTAGATGAGGCAGCATCAGAACTGGTAAGGTCAAGTGTAGAGCCAATTTTGGAACAATAT
AGGCCTATGATTTTGGCGTCATTGAAATTTTCCAAGTTCACTCTTGGTACTGTTGCTCCT
CAGTTCACAGGAATTTCTATTCTTGAAGGTGGAAGTGAAGGTATTACCATGGAATTAGAG
ATGAATAGGGATGGGAACCCAAGCATTATACTCGATATCATGACATACCTTGGTGTAGGA
TTACCAGTGCAGGTGAAGAACATTGGATTTACGGGGATTTTCAGGCTGATCTTCAGGCCG
CTTGTTGATGAGTTTCCCTGCTTTGGAGCTGTTTGTTATTCACTAAGGAAAAAGAAGAAG
CTGGATTTTACGCTTAAAGTGGTTGGTGGTGACATGACAGCAATACCTGGCATTTCTGAT
GCAATTGAGGGTACCATCCGCGATGCCATTGAAGACTCTATCATATGGCCAGTCCGAAAA
ATTATTCCCATTTTGCCTGGGGATTATAGTGACCTTGAACTGAAACCTACTGGAGTATTG
GAGGTGAAACTTGTTCAAGCAAAAgAGTTAACAAACAAGGACATCATTGGTAAATCTGAT
CCTTTTGCTGAGTTATATGTACGCCCTGTACGAGATAGAATGAAGAAGAGCAAAACAATT
AACAACGAACTGAATCCAATCTGGAATGAGCATTTCGAGTTTGTAGTTGAAGATCCATTG
ACACAACACTTGGTGATAAAGATCTATGATGATGAAGGGCTTCAAGCAGCTGAACTAATT
GGATGCGCACATGTTCGTTTGAATGAGCTTGAGCCTGGTAAAGTGAAGGATGTTTGGCTG
AAGTTGGTGAAAGATTTGGAGATCCAGAGAGACCAAAAGAATAGGGGCCAGGTGCACTTG
GAGCTATTATATTGTCCTTATGGCATGACAAATGGATTCAGTAACCCTTTTGCCAATAAT
GTACCATTGACTTCACTGGAGAAGGTTCTTAAAAGTGGAGTAGAAGCTGCCCAAAATGGA
GGTGAAATCAACAGGAGGAAGGATGTAATAGTACGAGGGGTACTCTCTGTAACCGTGATA
TCAGCTGAGGATCTGGCCCCTACTGATCTACTGGGGAAAGCTGATCCATATGTTGTAGTT
ACAATGAAGAAGACTGAAACAAAGAACAAAACCAGGGTTGTACCAGAAAGCTTAAATCCA
GTATGGAATCAGACTTTTGACTTTGTCGTTGAGGACGGATTGCATGATATGTTAATTCTA
GAAGTTTGGGACCATGACACCTTCGGAAAGGATTACATGGGAAGGTGCATATTGACATTG
ACAAGAGTACTAATGGAAGGTGAATATAAAGAAACCTTTGAACTAGATGGAGCTAAATCA
GGGAAACTGAACTTGCATCTCAAATGGGCACCACAGCCTATATACAGAGACTCCTAA

Transcripts
bt013623
bg135577

bf113177
bm535205
bf112702
aw737383
aw648703
bi210779
bi204472
bi204030

Lycopersicon esculentum fragment ***NTMC2Type2***
bp882205
ai488556

Lycopersicon esculentum fragment ***NTMC2Type2***
bp888331
bp887680

Lycopersicon esculentum fragment ***NTMC2Type2***
aw622856

Lycopersicon esculentum fragment ***NTMC2Type2***
dv105750

Lycopersicon esculentum fragment ***NTMC2Type3***
cv966956

Lycopersicon esculentum fragment ***NTMC2Type3***
bp891304

Lycopersicon esculentum Full Length ***NTMC2Type4***

MGLISGILMGMICGIGLMAVWKHMTRYRSNKRIAKAVDVKVMGCLCRDDLKKVCGDNFPE
WISFPVYEQVKWLNKQLSKLWPSIAEAGEAIIKESVEPLLEDYRPPGITSLKFSKLSLGT
VAPKIEGIRVQSLKKGQITMDIDLRWGGDPNIVLGVEAAMVASIPIQLKNLQVFTVIRVI
FQLTEEIPCISAVVVALLSEPKPRIDYVLKAVGGSLTALPGLSDMIDDTVNTIVTDMLEW
PHRIVVPIAPVDTSDLELKPQGKLTVTIVKANGLKNHEMIGKSDPYAVVHIRPLFKVKTK
TIDNNLNPVWDQTFELIAEDKETQSLFIEVFDKDNIGQDQRMGVAKLPLNELVADAAKEI
ELRLLPKLDMLKVKDKKDRGTITIKVLYHEFNKEEQLAALEAEKAILEERKKLKAEGVIG
STMDAVGSGVGMVGSGIGAGVGLVGTGLGAGVGIVGSGFGAVGSGLSKAGKFMGRTFTGS
SKKNGSSTPVNSVQENGGAKPLKSE

ATGGGGTTGATTTCTGGGATTCTGATGGGGATGATCTGTGGGATCGGGTTAATGGCAGTT
TGGAAGCATATGACGAGGTACAGGAGCAACAAGAGAATTGCTAAGGCAGTAGATGTAAAA
GTAATGGGCTGCCTCTGCAGGGATGATCTGAAGAAAGTTTGTGGCGATAACTTTCCTGAA
TGGATATCATTCCCGGTTTATGAACAGGTCAAATGGTTGAACAAACAGTTGAGCAAGTTG
TGGCCATCCATTGCCGAAGCAGGAGAGGCTATTATAAAAGAATCTGTTGAACCTCTTTTA
GAAGATTATCGACCTCCTGGAATTACTTCATTGAAGTTCAGCAAGTTATCATTGGGAACT
GTGGCACCCAAAATAGAAGGTATTCGTGTTCAGAGCCTTAAAAAAGGTCAAATCACTATG
GATATAGACCTCCGATGGGGTGGTGATCCCAATATTGTCTTAGGTGTTGAAGCTGCAATG
GTTGCTTCGATACCCATTCAGTTGAAAAATCTTCAAGTTTTCACTGTTATTCGTGTCATC
TTCCAACTAACTGAGGAAATCCCTTGCATCTCGGCTGTTGTTGTAGCATTACTTTCTGAG
CCAAAACCTAGAATTGACTATGTCTTGAAGGCGGTCGGTGGAAGTTTAACAGCTCTTCCT
GGACTTTCAGATATGATTGATGACACTGTAAATACAATAGTGACAGACATGCTAGAATGG
CCGCACAGAATTGTTGTTCCAATTGCACCTGTGGATACTAGTGATTTGGAACTTAAGCCA
CAAGGGAAGCTCACAGTAACTATTGTCAAGGCTAATGGCTTGAAGAACCACGAAATGATA
GGAAAATCCGATCCATATGCTGTTGTACACATACGCCCCCTTTTCAAGGTTAAGACAAAA
ACCATTGACAACAACCTAAATCCTGTTTGGGATCAGACGTTTGAATTAATTGCAGAAGAC
AAGGAGACGCAATCCCTATTCATTGAGGTCTTTGATAAAGACAATATTGGGCAAGACCAG
CGAATGGGTGTCGCAAAGTTGCCTCTGAATGAGCTTGTAGCTGATGCTGCTAAAGAAATT
GAATTAAGATTACTTCCAAAACTTGATATGCTCAAAGTCAAAGATAAGAAGGATCGGGGC
ACTATCACAATAAAGGTGTTGTATCATGAGTTCAACAAGGAAGAGCAGTTAGCTGCTCTG
GAGGCAGAGAAAGCGATCCTAGAAGAAAGGAAGAAGCTGAAAGCAGAAGGGGTCATCGGC
AGCACAATGGATGCGGTTGGTTCAGGTGTCGGTATGGTGGGAAGTGGCATTGGAGCAGGC
GTGGGCCTCGTTGGAACGGGACTAGGTGCCGGTGTAGGCATTGTTGGAAGTGGCTTTGGA

GCTGTTGGCAGTGGCCTAAGCAAAGCTGGAAAATTTATGGGAAGGACATTCACTGGCAGT
TCAAAGAAGAATGGTTCCTCTACTCCAGTAAACTCTGTTCAAGAAAATGGTGGTGCAAAG
CCACTCAAAAGTGAATAG

Transcripts
ab001389
bm409673
bp885038
aw035610
bp901775
aw034846

Lycopersicon esculentum fragment ***NTMC2Type5***
bg642550
bp885639

Lycopersicon esculentum fragment ***NTMC2Type5***
aw650683

Lycopersicon esculentum fragment ***NTMC2Type6***
bf096524
ai771821
ai487880
ai487307
ai484763
ai485070
ai487128
aw621622

Lycopersicon esculentum fragment ***NTMC2Type6***
aw931587
be435198
be433599
be433598

Lycopersicon esculentum fragment ***NTMC2Type6***
aw933967
aw930630

Lycopersicon esculentum fragment ***NTMC2Type6***
bi204303

Mimulus guttatus fragment ***NTMC2Type1***
cv520979

Mimulus guttatus fragment ***NTMC2Type1***
dv210377

Nicotiana sylvestris fragment ***NTMC2Type1***
bp747217
dw002979 (Nicotiana tabacum)
bp530131 (Nicotiana tabacum)
bp751867
bp751866
bp748394

Nicotiana tabacum fragment ***NTMC2Type1***
eb428928
eb452216
bp745304 (Nicotiana sylvestris)

Nicotiana tabacum fragment ***NTMC2Type1***
dv159508

Nicotiana tabacum fragment ***NTMC2Type1***

bp131413

Nicotiana tabacum fragment ***NTMC2Type2***
bp128695
bp128507
bp528124

Nicotiana tabacum fragment ***NTMC2Type2***
dw002296
aj718878

Nicotiana tabacum fragment ***NTMC2Type3***
bp129036

Nicotiana benthamiana Full Length ***NTMC2Type4***

MGLISGILMGMMLGIGLMAAWKHMMRYRSNKRVAKAVDVKLMGCLNRDDLKKVCGDNFPE
WISFPVYEQVKWLNKQLSKLWPFIAEAGEAIIRESVEPLLEDYRPPGITSLKFSKLSLGT
VAPKIEGIRVQSLKKGQITMDIDLRWGGDPNIVLGVEAAMVASIPIQLKNLQVFTVIRVI
FQLTEEIPCISAVVVALLSEPKPRIDYVLKAVGGSLTALPGLSDMIDDIVNTIVTDKLEW
PHRIVVPIGGVPVDTSDLELKPQGKLIVTVVKANGLKNHEMIGKSDPYAVVYIRPLFKVK
TKTIDNNLNPVWDQTFELIAEDKETQSLIVEVFDKDVGQDQRMGVAKLPLNELVAEAAKE
IELRLLPKLDMLKVKDKKDRGTIPIKVLYHEFNKEEQLAALEAEKAILEERKKLKSEGVI
GSTMDALDGAASLVGSGVGLVGTGLGAGVGLVGTGVGAGVGIVGSGFGAVGSGLSKAGKF
MGRTFPSSSKKSGSSTPVNSIQENGGAKPLKTVLANTD

ATGGGGTTGATTTCTGGGATACTGATGGGGATGATGTTGGGGATCGGTTTAATGGCTGCT
TGGAAACATATGATGAGGTACAGGAGTAACAAACGAGTTGCTAAGGCAGTAGATGTAAAA
CTAATGGGCTGCCTCAACAGGGATGATCTAAAGAAAGTGTGTGGTGATAACTTCCCTGAA
TGGATATCATTCCCGGTTTACGAGCAGGTCAAGTGGTTGAACAAACAATTGAGCAAATTG
TGGCCATTTATTGCCGAAGCAGGAGAGGCTATTATAAGAGAATCTGTTGAACCTCTTTTA
GAAGATTATCGACCTCCTGGAATTACTTCATTGAAGTTCAGCAAATTATCGTTGGGAACT
GTGGCACCTAAAATAGAAGGTATTCGTGTTCAGAGCCTTAAAAAAGGTCAAATCACTATG
GATATTGACCTCAGATGGGGTGGTGATCCCAATATCGTTTTAGGTGTTGAAGCTGCAATG
GTTGCTTCTATACCCATTCAGCTGAAAAATCTTCAAGTATTCACTGTTATTCGTGTTATC
TTCCAACTAACTGAGGAAATTCCTTGCATCTCGGCTGTTGTTGTAGCATTACTTTCTGAG
CCAAAGCCTAGAATCGACTATGTTTTGAAGGCGGTCGGTGGAAGTTTAACTGCTCTTCCT
GGACTTTCGGATATGATTGATGACATTGTAAATACAATTGTGACAGATAAGTTAGAATGG
CCCCACAGAATTGTTGTTCCAATTGGCGGCGTACCTGTGGATACTAGCGATTTGGAGCTT
AAGCCACAGGGGAAGCTCATAGTAACTGTAGTCAAGGCTAATGGCTTAAAGAACCACGAA
ATGATAGGAAAATCTGATCCATATGCGGTTGTATACATTCGTCCACTTTTCAAGGTTAAG
ACAAAAACCATTGACAACAACCTAAATCCCGTTTGGGATCAGACGTTTGAGTTAATTGCA
GAAGACAAGGAGACCCAATCCCTCATTGTGGAGGTCTTCGATAAAGACGTTGGGCAAGAC
CAGCGAATGGGTGTCGCTAAGTTGCCTCTGAATGAGCTAGTAGCTGAGGCTGCCAAAGAA
ATTGAATTAAGGTTACTGCCAAAACTTGATATGCTCAAAGTCAAAGATAAGAAGGATAGG
GGAACTATCCCAATAAAGGTGTTGTATCATGAATTCAACAAGGAAGAACAGTTAGCTGCT
CTCGAGGCAGAGAAGGCGATCCTAGAAGAAAGGAAGAAACTGAAATCAGAAGGTGTCATT
GGGAGCACAATGGATGCCCTTGACGGGGCTGCATCACTGGTTGGTTCAGGTGTTGGTTTG
GTGGGCACTGGTCTTGGGGCAGGAGTGGGGCTCGTTGGAACCGGGGTAGGTGCTGGTGTT
GGGATAGTTGGAAGTGGCTTTGGAGCTGTTGGCAGTGGCCTAAGCAAAGCTGGAAAATTT
ATGGGAAGGACATTCCCAAGCAGTTCAAAGAAGAGTGGTTCCTCTACTCCAGTAAACTCT
ATTCAAGAAAATGGTGGTGCAAAGCCACTCAAGACTGTACTTGCAAATACTGATTAG

Transcripts
eb427507 (Nicotiana tabacum)
ck289733
ck281978
cn747100
ck281977
ck289734
ck281976
ck281979
cn748601
bp133813 (Nicotiana tabacum)

Nicotiana tabacum fragment ***NTMC2Type4***
bp526529

Nicotiana tabacum fragment ***NTMC2Type5***
bp528499

Nicotiana tabacum fragment ***NTMC2Type6***
bp192602

Nicotiana sylvestris fragment ***NTMC2Type6***
bp750219

Ocimum basilicum fragment ***NTMC2Type1***
dy323149
dy322974

Ocimum basilicum fragment ***NTMC2Type1***
dy341845

Ocimum basilicum fragment ***NTMC2Type2***
dy323638
dy340491
dy323640

Ocimum basilicum fragment ***NTMC2Type3***
dy331165
dy331166
dy323024
dy329129
dy329130
dy338434
dy340945
dy340944
dy327426
dy327427
dy338321

Ocimum basilicum fragment ***NTMC2Type5***
dy338513
dy338512
dy337550

Ocimum basilicum fragment ***NTMC2Type6***
dy328894
dy336678
dy336677
dy333670

Ocimum basilicum fragment ***NTMC2Type6***
dy333669

Ocimum basilicum fragment ***NTMC2Type6***
dy328895

Panax ginseng fragment ***NTMC2Type6***
cn845928

Petunia x hybrida fragment ***NTMC2Type2***
cv299750

Petunia x hybrida fragment ***NTMC2Type2***
cv295655

Petunia x hybrida fragment ***NTMC2Type4***
cv297444

Petunia x hybrida fragment ***NTMC2Type4***
cv297102

Petunia x hybrida fragment ***NTMC2Type4***
cv297457

Solanum tuberosum Full Length ***NTMC2Type1***

MGFVSTILGFCGFGVGVSCGLTIGYYLFIYFQPCDVKDPVIRPLVERDSKSLQQLLSEIP
LWVKCPDYDRVDWLNKFLEYMWPYLDKAICRTAKDIAAPIIAEQIPKYKIDSVEFETLTL
GSLPPTFQGMKVYVTEEKELIMEPSIKWAGNPNVTVAVKAFGLKATVQVVDLQVFAAPRI
TLKPLVPSFPCFANIFVSLMEKPHVDFGLKLLGADLMSIPGLYRFVQETIKDQVANMYLW
PKTLEVQILDPSKAMKKPVGVLHVKILRAMNLKKKDLLGASDPYVKLKLTESKLPSKKTP
VRHKNLNPEWNEEFNMVVKDPESQALELSVYDWEQIGKHDKMGMNVIPLKDLTPDESKTM
TLNLLKNMDANDAQNDKDRGQIMVELTYKPFKEDELPKDFEDNDAAHKVPEGTPPGGGVL
MIIVHEAQDVEGKHHTNPYVKILFKGEERKTKQVKKNRDPRWGEEFTFVLEEPPVNDRLH
MEVVSTSTRIGLLHPKETLGYVDINLSDVVSNKRINEKYHLIDSKNGRLQVELQWRTAS

ATGGGTTTTGTGAGTACGATTTTGGGTTTTTGTGGATTTGGAGTTGGGGTTTCATGTGGA
TTGACGATCGGTTACTATCTGTTCATCTATTTCCAGCCCTGTGATGTTAAGGACCCTGTT
ATTCGTCCATTGGTTGAGCGAGATTCTAAAAGCTTGCAGCAGTTGCTGTCTGAAATTCCT
CTCTGGGTCAAATGTCCGGATTATGACCGTGTGGACTGGCTCAACAAATTTCTTGAGTAT
ATGTGGCCTTACCTGGACAAGGCAATTTGCAGGACTGCAAAAGATATTGCAGCACCAATT
ATTGCTGAACAAATACCAAAGTATAAAATTGATTCTGTTGAATTTGAAACACTAACTTTG
GGGTCCTTACCTCCTACTTTCCAGGGGATGAAGGTTTATGTTACTGAAGAAAAAGAATTG
ATCATGGAACCATCAATAAAGTGGGCTGGAAATCCTAATGTTACTGTTGCGGTCAAAGCA
TTCGGATTGAAAGCAACTGTTCAGGTTGTGGACTTGCAGGTTTTTGCAGCTCCACGTATT
ACTCTGAAGCCTCTGGTTCCAAGCTTTCCATGTTTTGCCAATATCTTTGTGTCCCTCATG
GAAAAGCCACATGTTGACTTTGGATTGAAGCTTTTGGGGGCTGATCTTATGTCCATCCCT
GGCCTGTACAGGTTTGTCCAGGAGACCATTAAAGATCAGGTTGCTAACATGTATCTTTGG
CCTAAAACTCTAGAAGTGCAGATATTGGATCCATCCAAAGCAATGAAAAAACCTGTTGGG
GTCCTGCATGTGAAGATTCTGAGGGCAATGAATCTGAAAAAGAAAGATCTATTGGGTGCA
TCTGATCCTTACGTAAAACTTAAGCTCACTGAGTCAAAACTTCCTTCAAAGAAAACCCCT
GTAAGGCATAAGAATTTAAATCCAGAGTGGAATGAGGAGTTTAATATGGTTGTTAAAGAT
CCAGAATCGCAAGCATTGGAGCTCTCTGTTTATGATTGGGAGCAGATTGGCAAGCATGAT
AAGATGGGCATGAACGTTATTCCACTGAAAGATTTGACTCCTGATGAATCAAAAACGATG
ACACTGAATCTCCTAAAGAATATGGATGCGAATGATGCTCAAAATGATAAGGACCGTGGT
CAGATCATGGTGGAATTAACCTACAAACCATTTAAGGAGGATGAGTTGCCnAAAGATTTT
GAAGATAATGATGCAGCACATAAGGTTCCAGAAGGAACGCCACCAGGGGGAGGCGTCCTT
ATGATTATTGTCCACGAAGCTCAAGATGTTGAAGGAAAGCACCATACTAATCCATATGTC
AAGATTCTTTTCAAAGGAGAGGAGAGAAAAACTAAGCAAGTCAAGAAGAACAGGGATCCA
AGATGGGGAGAGGAGTTTACTTTTGTGTTGGAGGAGCCTCCTGTGAATGATAGGCTGCAT
ATGGAAGTTGTCAGCACCTCTACAAGGATTGGCCTATTGCATCCTAAGGAGACATTGGGT
TATGTTGATATAAATCTTTCCGATGTTGTTAGCAACAAAAGGATCAATGAGAAGTACCAC
CTCATTGATTCAAAGAACGGTCGCCTTCAAGTTGAGCTGCAATGGCGAACTGCTTCTTGA

Transcripts
bm108704
bg888482
be472787
aw906453
bg592436
bg098007
ck854156
bg097629
bg598648
bg596589
dn982760 (Solanum chacoense)
dn850071
bq118841
cx161991
cv505904
ck565429
ck565421

Solanum tuberosum fragment ***NTMC2Type1***
ay356322
bq511071
bg888699
bq511072
ay356282

Solanum tuberosum fragment ***NTMC2Type1***
dn850070
dn978301 (Solanum chacoense)

Solanum tuberosum fragment ***NTMC2Type2***
cn214182
bg600859
bq509051
ck862458

Solanum tuberosum fragment ***NTMC2Type2***
bi405943
bg888045

Solanum tuberosum fragment ***NTMC2Type2***
dv625410

Solanum tuberosum fragment ***NTMC2Type2***
bq509052

Solanum tuberosum fragment ***NTMC2Type2***
dn590525

Solanum tuberosum fragment ***NTMC2Type3***
bq046414
bq517369
bm404138
cv503331
ck862691
cv477399
dn908864
dn908863

Solanum tuberosum fragment ***NTMC2Type3***
ck863158

Solanum tuberosum Full Length ***NTMC2Type4***

MGLITGILMGMICGIGLMAVWKHMTRYRSNKRIAKAVDVTVMGCLCRDDLKKVCGDNFPE
WISFPVYEQVKWLNKQLSKLWPFIAEAGEAIIKECVEPLLEDYRPPGITSLKFSKLSLGT
VAPKIEGIRVQSLKKGQITMDIDFRWGGDPNIVLGVEAAMVASIPIQLKNLQVFTVIRVI
FQLTEEIPCISAVVVALLSEPKPRIDYVLKAVGGSLTALPGLSDMIDDTVNTIVTDMLEW
PHRIVVPIGGIPVDTSDLELKPQGKLTVTIVKANGLKNHEMIGKSDPYAVVYIRPLFKVK
TKTIDNNLNPVWDQTFELIAEDKETQSLFVEVFDKDNIGQDERMGVAKLPLNELVADAAK
EIELRLLPKLDMLKVKDKKDRGTITIKVLYHEFNKEEQLAALEAEKAILEERKKLKAEGV
IGSTMDAVGSGVGMVGSGIGAGVGFVGTGLGAGVGIVGSGFGAVGSGLSKAGKFMGRTFT
GSSKKNGSSTPVNSVQENGGAKPLKSE

ATGGGGTTGATTACTGGGATTCTGATGGGGATGATCTGCGGGATAGGGTTAATGGCAGTT
TGGAAGCATATGACGAGGTACAGGAGCAACAAGAGAATTGCTAAGGCAGTAGATGTAACA
GTAATGGGCTGCCTCTGCAGGGATGATCTAAAGAAAGTTTGTGGCGATAACTTTCCTGAA
TGGATATCATTCCCGGTTTATGAACAGGTCAAATGGTTGAACAAACAGTTGAGCAAATTG
TGGCCATTCATTGCCGAAGCAGGAGAGGCTATTATAAAAGAATGTGTTGAACCTCTTTTA
GAAGATTATCGACCTCCTGGAATTACTTCATTGAAGTTCAGCAAATTATCATTGGGAACT
GTGGCACCTAAAATAGAAGGTATTCGTGTTCAGAGCCTTAAAAAAGGTCAAATCACTATG
GATATAGACTTCCGATGGGGTGGTGATCCCAATATTGTCTTAGGTGTTGAAGCTGCAATG

```
GTTGCTTCTATACCCATTCAGTTGAAAAATCTTCAAGTTTTCACTGTTATTCGTGTTATC
TTCCAACTAACTGAGGAAATCCCTTGCATCTCGGCTGTTGTTGTAGCATTACTTTCTGAG
CCAAAACCTAGAATTGACTATGTCTTGAAGGCGGTCGGTGGAAGTTTAACAGCTCTTCCT
GGACTTTCAGATATGATTGATGACACTGTAAATACAATAGTGACAGATATGCTAGAATGG
CCACACAGAATTGTTGTTCCGATTGGAGGCATACCTGTGGATACCAGTGATTTGGAACTT
AAACCACAAGGGAAGCTCACAGTAACTATTGTCAAGGCTAATGGCTTGAAGAACCACGAA
ATGATAGGAAAATCCGATCCATATGCTGTTGTATACATACGCCCACTTTTCAAGGTTAAG
ACAAAAACCATTGACAACAACCTAAATCCTGTTTGGGATCAGACGTTTGAATTAATTGCA
GAAGACAAGGAGACCCAATCCCTATTCGTCGAGGTCTTTGATAAAGACAATATCGGGCAA
GACGAGCGAATGGGTGTCGCAAAATTGCCTCTGAATGAGCTTGTAGCTGACGCTGCCAAA
GAAATTGAATTAAGATTACTGCCAAAACTTGATATGCTCAAAGTCAAAGATAAGAAGGAT
CGGGGAACTATCACAATAAAGGTGTTGTATCATGAGTTCAACAAGGAAGAGCAGTTAGCT
GCTCTGGAGGCAGAGAAGGCGATCCTAGAAGAAAGGAAGAAGCTGAAAGCAGAAGGGGTC
ATCGGCAGCACAATGGATGCAGTTGGTTCAGGTGTCGGTATGGTGGGAAGTGGCATTGGA
GCAGGCGTGGGGTTCGTTGGAACGGGACTAGGTGCTGGTGTAGGCATAGTTGGAAGTGGC
TTTGGAGCTGTTGGCAGTGGTCTAAGCAAAGCTGGAAAATTTATGGGAAGGACATTCACT
GGCAGTTCAAAGAAGAATGGTTCCTCTACTCCAGTAAACTCTGTTCAAGAAAATGGTGGT
GCAAAGCCACTCAAAAGTGAATAG
```

Transcripts
dn977897 (Solanum chacoense)
ck274725
bg595124
bm405581
bg887809
bg599857
cv431565
bg589214
bg589754
bg592489
be920264
ck273425
bq117808
be342891
bm108677
ck266872
aj487400
ck274726
ck273426
ck266873
bq513430
bq117809
cv476721

Solanum tuberosum fragment ***NTMC2Type4***
bg097505
dn979788 (Solanum chacoense)

Solanum tuberosum fragment ***NTMC2Type4***
ck275320

Solanum tuberosum fragment ***NTMC2Type5***
dn586897
dr036417

Solanum tuberosum fragment ***NTMC2Type5***
bg600954
bq509092

Solanum tuberosum fragment ***NTMC2Type6***
bg595647
ck719791
bg593062
dv626353

Solanum tuberosum fragment ***NTMC2Type6***
dn922126
cv495528

dn922125

Solanum tuberosum fragment ***NTMC2Type6***
dn848833
dn983140

Solanum tuberosum fragment ***NTMC2Type6***
dr035875

Solanum tuberosum fragment ***NTMC2Type6***
ck851107

Solanum tuberosum fragment ***NTMC2Type6***
cn216452

Solanum tuberosum fragment ***NTMC2Type6***
cn465479

Solanum tuberosum fragment ***NTMC2Type6***
dr035876

Taraxacum officinale fragment ***NTMC2Type1***
dy812930
dy814661
dy814695
dy817635
dy808787
dy809157
dy808604
dy808818
dy812519
dy812643
dy803459
dy817065
dy817425
dy817402
dy817662
dy820578

Taraxacum officinale fragment ***NTMC2Type1***
dy813584

Taraxacum officinale fragment ***NTMC2Type1***
dy830493

Taraxacum officinale fragment ***NTMC2Type2***
dy814536
dy817456
dy813975
dy810643

Taraxacum officinale fragment ***NTMC2Type4***
dy822461

Triphysaria versicolor fragment ***NTMC2Type2***
dr176286

Triphysaria versicolor fragment ***NTMC2Type6***
dr170503

Vaccinium corymbosum fragment ***NTMC2Type1***
dr066990
dr067326

Zinnia elegans fragment ***NTMC2Type1***
au307519

Zinnia elegans fragment ***NTMC2Type1***
au287958

Zinnia elegans fragment ***NTMC2Type1***
au292887

Zinnia elegans fragment ***NTMC2Type1***
au307890

Zinnia elegans fragment ***NTMC2Type2***
au293446
au294513

Zinnia elegans fragment ***NTMC2Type2***
au294738

Zinnia elegans fragment ***NTMC2Type2***
au289422

Zinnia elegans fragment ***NTMC2Type3***
au286068

Zinnia elegans fragment ***NTMC2Type4***
au289490

Zinnia elegans fragment ***NTMC2Type4***
au309464

Zinnia elegans fragment ***NTMC2Type5***
au290527
au290528

Zinnia elegans fragment ***NTMC2Type5***
au285544

Arabidopsis thaliana Full Length ***NTMC2Type1.1***
Gene 86 Craxton, M. BMC Genomics. 2004 Jul 6;5(1):43
bp863382
av823576
aj617630
ay045836
av829482
dr297252
bp847581
ay087925
dr297256
dr297258
dr297254
bp854220
bp843853
t21455
bp866911
bp856566
av548903
dr297250
dr297255
t46806
aa712280
ab102951
bt004371
av440032
bu635082
dr235464
bp831811

bp837009
bp819338
dr297251
dr297257
dr297253
av824369
av440219
cf651641
cf651642
dr376497
bp828963
bp822021
av794019
bp611465
bp607117
bp662029
bp633555
bp603477
av804716
av805434
av813685
av804083
av815069
av818050
bp610680
bp660719
bp597757
bp671001
bp783030
av820214
bp605707
bp600978
bp783628
av785876
ai998724
av820896
av814579
bp587855
av797505
av522147
bp785891
av806471
bp667181
bp790057
av786086
bp785766
av804372
av784572
av548720

Arabidopsis thaliana Full Length ***NTMC2Type1.2***
Gene 85 Craxton, M. BMC Genomics. 2004 Jul 6;5(1):43
z18414

Arabidopsis thaliana Full Length ***NTMC2Type1.3***
Gene 87 Craxton, M. BMC Genomics. 2004 Jul 6;5(1):43
av826403
ay059741
bx830716
bp847019
ab102952
be527633
au036557
av794749
bp787052

Arabidopsis thaliana Full Length ***NTMC2Type2.1***
Gene 94 Craxton, M. BMC Genomics. 2004 Jul 6;5(1):43
bp815183
bp562438
ay140038
dr363584
au236937
bp842357
bp804353
bu636347
bt008907

av524824
ai994733
n37369
av557680
dr383723
au227944
bp787918
bp664502
av808397
bp782304
av814733
av555444
av555098

Arabidopsis thaliana Full Length ***NTMC2Type2.2***
Gene 93 Craxton, M. BMC Genomics. 2004 Jul 6;5(1):43
This gene as descirbed in the above reference is incorrect. The corrected gene description is below.
This has been altered September 2006

chromosome 5
MGFLFGLFIGIAVSFGLVVAFARYSSVRSTRRADL**^ 0**
AKTIAAFARMTVQDSRKLLPGDFYPSWVVFSQRQK**^ 0** RNA editing
LNWLNLELEKIWPYVNE**^ 0**
AASELIKSSVEPVLEQYTPAMLASLKFSKFTLGTVAPQFT**^ +1**
GVSILESESGPNGITMELEMQWDGNPKIVLDVKTLLGVSLPIE**^ 0**
VKNIGFTGVFRLIFKPLVDEFPCFGALSYSLREK**^ 0**
KGLDFTLKVIGGELTSIPGISDAIEETIRDAIE**^ 0**
DSITWPVRKIIPILPGDY**^ +2**
SDLELKPVGKLDVKVVQAKDLANKDMIGKSDPYAIVFIRPLPDRTKKTKTI**^ 0**
SNSLNPIWNEHFEFIVEDVSTQHLTVRVFDDEGVGSSQLIGAAQVPLNELVPGKVKDIWL
KLVKDLEIQRDTKNRGQ**^ 0**
VQLELLYCPLGKEGGLKNPFNPDYSLTILEKVLKPESEDSDATDMKKLVTSKKKDVIVRG
VLSVTVVAAEDLPAVDFMGKADAFVVITLKKSETKSKTRVVPDSLNPVWNQTFDFVVEDA
LHDLLTLEVWDHDKFGKDKIGRVIMTLTRVMLEGEFQEWFELDGAKSGKLCVHLKWTPRL
KLRDAS

<u>ATG</u>GGTTTTCTCTTTGGTTTGTTCATAGGAATCGCGGTTTCTTTCGGTTTGGTCGTCG
CATTTGCTCGCTACTCTAGTGTCAGATCCACTCGTCGAGCTGATTTG
phase 0
al391222.1 83542-83646

GCAAAGACCATTGCTGCATTTGCAAGAATGACAGTTCAAGATTCGAGAAAACTTCTCC
CGGGAGATTTTTATCCTTCTTGGGTTGTGTTTTCTCAGAGACAAAA
phase 0
al391222.1 83734-83837

cDNAs have g inserted before next exon

TTAAATTGGCTTAATCTTGAACTTGAAAAGATCTGGCCTTATGTAAATGAG
phase 0
al391222.1 83931-83981

GCAGCTTCTGAGCTGATTAAAAGCAGTGTGGAACCAGTACTTGAACAGTATACACCAG
CTATGTTAGCATCTCTCAAGTTCTCTAAATTCACACTTGGGACTGTGGCTCCTCAGTT
CACAG
phase +1
al391222.1 84082-84202

GAGTTTCTATCTTAGAAAGTGAAAGTGGACCTAATGGAATCACAATGGAACTTGAAAT
GCAATGGGATGGTAATCCAAAAATCGTGTTGGATGTCAAAACCTTACTTGGCGTGTCT
CTACCTATTGAG
phase 0
al391222.1 84510-84637

GTCAAAAACATAGGATTCACAGGTGTTTTCAGGCTGATTTTCAAACCTCTTGTTGATG
AATTCCCTTGCTTTGGGGCACTTTCTTATTCACTCCGCGAGAAG
phase 0
al391222.1 84717-84818

AAAGGGCTTGATTTTACCCTCAAAGTTATAGGTGGTGAATTAACATCAATTCCAGGGAT
TTCAGATGCAATCGAA
phase 0
al391222.1 84893-84967

GAAACTATTCGTGACGCCATTGAAGACAGTATAACATGGCCAGTTAGGAAAATTATACC
TATTTTACCAGGAGATTACAG
phase +2
al391222.1 85057-85136

TGATCTGGAGTTGAAACCTGTTGGAAAATTGGATGTGAAGGTTGTGCAAGCAAAGGACTT
AGCTAACAAAGACATGATTGGAAAATCTGATCCTTACGCTATTGTATTCATTCGCCCTTT
GCCTGACAGAACCAAAAAGACCAAAACTATT
phase 0
al391222.1 85212-85362

AGTAACTCGTTGAATCCGATTTGGAATGAACATTTTGAGTTCATTGTGGAAGATGTCTCA
ACTCAACATTTGACAGTAAGAGTCTTTGACGACGAAGGAGTTGGATCTTCTCAGCTTATT
GGGGCTGCTCAAGTACCGTTAAATGAACTCGTACCCGGGAAAGTTAAAGATATTTGGTTG
AAGTTAGTCAAGGATTTAGAGATCCAAAGGGATACAAAAAATAGGGGTCAG
phase 0
al391222.1 85457-85687

GTGCAGTTGGAGCTCTTATATTGTCCCTTAGGCAAAGAAGGCGGATTGAAGAACCCGTTT
AACCCGGACTACTCATTGACCATTTTAGAGAAGGTACTGAAGCCGGAAAGCGAGGATTCG
GATGCCACGGATATGAAAAAACTGGTGACCTCGAAGAAAAAAGATGTGATAGTGAGAGGA
GTGTTGTCTGTGACAGTGGTGGCAGCTGAAGACTTACCAGCTGTAGATTTTATGGGAAAA
GCAGATGCATTTGTGGTAATCACATTGAAGAAATCAGAAACGAAATCCAAGACAAGGGTA
GTACCGGATAGCTTGAATCCGGTTTGGAACCAGACGTTTGATTTTGTAGTGGAAGATGCT
TTGCATGATCTATTGACGCTTGAAGTATGGGACCATGACAAATTTGGCAAGGACAAGATA
GGGAGAGTGATAATGACATTGACGCGAGTGATGTTAGAAGGAGAGTTTCAAGAGTGGTTT
GAGTTAGATGGAGCTAAATCAGGGAAGCTTTGTGTCCATCTTAAATGGACTCCTAGGCTT
AAGCTCAGAGACGCCTCTTGA
al391222.1 85801-86361

Transcripts
am410051

Arabidopsis thaliana Full Length ***NTMC2Type3***

Gene 97 Craxton, M. BMC Genomics. 2004 Jul 6;5(1):43
This gene as descirbed in the above reference is incorrect. The corrected gene description is below.
This has been altered March 2005

chromosome 3
MGRRIKRKGLINTEAAREFINHLVAERHSLLLLVPLVLAFWAIERWVFAFSNWVPLVVAV
WASLQ**^ 0**
YGSYQRALLAEDLTKKWRQTVFNAS**^ 0**
TITPLEHCQWLNKLLSEIWLNYMNKKLSLRFSSMVE**^ 0**
KRLRQRRSRLI**^ 0**
ENIQLLEFSLGSCPPLLGLHGTCWSKSGEQ**^ 0**
KIMRLDFNWDTTDLSILLQAKLSMPFNRTARIVVNSLCIKGD**^ 0**
ILIRPILEGRALLYSFVSNPEVRIGVAFGGGGGQSLPATELPGVSSWL**^ 0**
VKILTETLNKKMVEPRRGCFSLPATDLHKTAIGGIIYVTVVSGNNLNRRILRGSPSKSSE
IGEGSSGNSSSKPVQTFVEVELEQLSRRTEMKSGPNPAYQSTFNMILHDNTGTLKFNLYE
NNPGSVRYDSLASCEVK**^ 0**
MKYVGDDSTMFWAVGSDNGVIAKHAEFCGQEIEMVVPFEGVSSGE**^ 0**
LTVRLLLKEWHFSDGSHSLNSVNSSSLHSLDSSSALLSKTGRKIIVTVLAGKNLVSKDKS
GKCDASVKLQYGK**^ 0**
IIQKTKIVNAAECVWNQKFEFEELAGEEYLKVKCYREEMLGTDNIGTATLSLQGINNSEM
HIWVPLEDVNSGEIELLIEALDPEYSE**^ 0**
ADSSKGLIELVLVEARDLVAADIRGTSDPYVRVQYGEKKQRTK**^ 0**
VIYKTLQPKWNQTMEFPDDGSSLELHVKDYNTLLPTSSIGNCVVEYQGLKPNETADKWII
LQGVKHGEVHVRVTRKVTEIQRRASAGPGTPFNKALLLSNQMKQVMIKFQNLIDDGDLEG
LAEALEELESLEDEQEQYLLQLQTEQSLLINKIKDLGKEILNSSPAQAPSRDS

GCTATCACAATGTTCAATTTGTCTAAAGATCCGTTACCTCTCTATGCGAGAGAAGCTGTG
AGAGAGTTTCCGACAAGTAACTAGGAGATCGTAATTCTTGTCTGAAAAACAACTTTTTTT
ATTCGAAACTTCTCTCACAGATTTTTCTCAGTTTTTCCAGAAATTTTGTTCTTCTTCGTC
CACTCACCGTGGATCTCTCTAGTTTGTTGAAGAAATCAAATTGTGGAATAAATAAAAAAA
CAGAGAAATAAATACTGCAAGGAAAGTTTCTTACTTCGTCACTTTGACCCAGAAAGCTTT
CAGATTTTCCCGGAAAAGAAATCTGCTGGAGAAATTTCATTGACTGACTTATGGATTTGT
GAAGAAGCTTGAAGGCGAATCGAAGAGATGGGTCGGAGAATAAAGAGGAAAGGTCTGATA
AACACGGAAGCTGCAAGAGAATTTATCAATCATCTAGTTGCTGAGAGGCATTCGCTGTTG
TTGCTGGTTCCTTTGGTCTTAGCTTTCTGGGCAATTGAAAGATGGGTCTTTGCTTTCTCC
AATTGGGTTCCTCTCGTTGTCGCCGTCTGGGCTTCTCTTCAG
phase 0
ab026658.1 24090-24671

TATGGAAGTTACCAAAGGGCACTACTTGCAGAAGATCTAACCAAGAAGTGGAGGCAAACC
GTTTTCAATGCGTCG
phase 0
ab026658.1 25127-25201

ACGATAACTCCATTGGAACATTGTCAATGGCTGAACAAGTTACTGTCTGAAATTTGGCTA
AACTATATGAACAAGAAACTCTCTCTTAGGTTTTCTTCTATGGTGGAG
phase 0
ab026658.1 25295-25402

AAACGATTGAGGCAACGAAGATCAAGATTAATC
phase 0
ab026658.1 25514-25546

GAAAATATACAATTGTTGGAGTTTTCTCTTGGCTCATGCCCTCCTTTGCTAGGACTACAT
GGAACTTGTTGGTCAAAATCAGGGGAACAG
phase 0
ab026658.1 25642-25731

AAAATCATGCGATTGGATTTCAACTGGGACACAACGGATCTAAGTATTTTGTTGCAAGCC
AAGTTATCCATGCCGTTTAATCGAACAGCACGAATCGTTGTCAACAGTCTCTGCATCAAG
GGAGAT
phase 0
ab026658.1 25816-25941

ATTCTGATCAGACCGATTCTTGAAGGAAGAGCATTGCTTTATTCCTTTGTATCGAATCCT
GAAGTTAGAATCGGAGTTGCTTTCGGTGGTGGCGGTGGCCAATCTCTCCCCGCTACAGAG
CTTCCTGGTGTCTCCTCTTGGCTG
phase 0
ab026658.1 26022-26165

GTAAAGATTCTAACGGAAACTTTGAACAAGAAGATGGTAGAGCCACGCCGGGGATGTTTC
TCGTTACCCGCAACAGATCTTCACAAAACAGCCATCGGGGGAATTATCTACGTAACCGTT
GTATCTGGTAACAATCTTAACCGCAGAATTCTTCGGGGAAGCCCCTCGAAAAGTTCTGAG
ATCGGAGAAGGTAGCAGCGGAAATAGCAGCAGCAAACCTGTTCAAACATTTGTGGAGGTC
GAACTCGAGCAACTTTCTCGACGAACCGAGATGAAGTCAGGACCGAATCCAGCTTATCAG
TCGACATTTAACATGATCTTACACGACAATACAGGGACACTCAAGTTTAACCTCTATGAA
AATAATCCTGGGAGTGTCAGATATGACAGTCTCGCTAGCTGTGAAGTTAAG
phase 0
ab026658.1 26254-26664

ATGAAATATGTAGGTGATGATTCAACGATGTTTTGGGCCGTTGGATCTGATAACGGTGTG
ATTGCAAAACACGCTGAGTTCTGTGGTCAAGAAATCGAGATGGTTGTTCCGTTTGAAGGA
GTTAGCTCCGGAGAG
phase 0
ab026658.1 26744-26878

TTGACTGTCCGGCTACTTCTAAAAGAATGGCACTTCTCTGATGGCTCACATAGCTTGAAT
AGCGTTAATTCGAGTTCATTACACTCCCTCGACAGCTCGTCTGCCCTCCTCTCGAAAACC
GGCCGGAAAATTATCGTGACGGTTTTGGCTGGAAAGAACCTTGTTTCTAAAGATAAGTCC
GGAAAATGTGATGCCTCTGTCAAGTTACAGTACGGAAAA
phase 0

ab026658.1 26982-27200

ATTATACAAAAGACGAAGATAGTGAACGCAGCAGAGTGTGTGTGGAACCAGAAATTTGAG
TTCGAAGAGTTAGCCGGAGAAGAATATTTAAAGGTGAAATGCTATAGAGAAGAGATGCTT
GGCACGGACAACATCGGTACAGCTACATTGAGTCTTCAAGGAATCAATAACAGCGAAATG
CATATATGGGTTCCGCTTGAAGATGTTAATTCTGGAGAAATAGAGCTTTTGATCGAAGCT
TTGGATCCCGAGTACAGCGAA
phase 0
ab026658.1 27291-27551

GCGGATTCTAGTAAAGGCTTGATTGAATTGGTTCTCGTTGAAGCGCGGGATCTTGTGGCC
GCGGATATTAGAGGAACCAGTGATCCTTATGTTAGGGTTCAATATGGAGAGAAGAAACAG
AGGACAAAG
phase 0
ab026658.1 27631-27759

GTGATTTATAAGACGTTGCAACCAAAATGGAACCAGACAATGGAGTTTCCAGATGATGGG
AGCTCCTTGGAGCTACATGTGAAAGACTATAACACTCTGCTTCCGACTTCAAGCATCGGT
AACTGCGTTGTGGAGTACCAAGGGTTAAAGCCAAATGAGACTGCCGATAAGTGGATAATT
CTCCAAGGAGTGAAACACGGGGAGGTCCATGTCAGAGTCACGAGGAAAGTCACGGAGATT
CAGAGACGCGCCAGCGCAGGTCCTGGCACACCGTTTAACAAAGCTCTGTTACTGTCCAAC
CAGATGAAGCAGGTGATGATAAAGTTTCAGAATTTGATTGACGATGGAGATCTTGAAGGT
CTTGCTGAAGCTTTAGAAGAGCTGGAAAGTTTGGAGGACGAACAAGAACAGTATTTGCTC
CAGCTTCAGACAGAGCAATCGCTGCTTATCAACAAGATCAAAGACCTTGGTAAGGAGATT
CTTAACTCATCCCCAGCTCAAGCCCCGTCTCGAGATTCTTAA
ab026658.1 27850-28371

Transcripts
av827801
ay056799
av530256
bx842161
av529739
av530152
av529697
bp833967
bp824845
av528456
av529286
z33744
ay133527
av528576
be525146
cb263349
bx824072
be530128
av523931
av523957
av522907
av523007
av524350
bp610671
av561396
av563676
cd529523
av524273
cd531143
bp646941
av523585
av799460
z33745
ai996289
av565446

Arabidopsis thaliana Full Length ***NTMC2Type4***
Gene 108 Craxton, M. BMC Genomics. 2004 Jul 6;5(1):43
bp847472
bp809029
bp814885
bx824688
cb255586
bp562947

au235298
bp841659
bp827505
bp827506
bp801131
bp857692
bp810245
bx837129
x96598
bt004564
av524600
dr265269
dr265268
dr265265
dr265266
dr265267
r30407
ai994558
bx834528
av442158
t42317
av518057
dr370374
av555732
bp581735
bp571707
bp575793
au225964
bp582327
bp790114
av441156
bp606516
av555667
av556670

Arabidopsis thaliana Full Length ***NTMC2Type5.1***

Gene 109 Craxton, M. BMC Genomics. 2004 Jul 6;5(1):43
av830086
ay099625
bt010734
cb259475
av800720
av810041

Arabidopsis thaliana Full Length ***NTMC2Type5.2***

Gene 110 Craxton, M. BMC Genomics. 2004 Jul 6;5(1):43
This gene as descirbed in the above reference is incorrect. The corrected gene description is below.
This has been altered September 2006

chromosome 3
MILQSSSSCSSFDFPSFVSRRLLCPCSNEHGLIVFSDGFTKRRRILRRVHAANSNSRFVS
SGIRTDSKNIGLADSARRAARSLVVTRFSNEFEDEEASSSSQESAIQGDRNNFTNFREDP
IVDKLRTQLGVIHPIPSPPISRNAIGLFAFFFFVGVICDKLWTWRKRRRQMAGDGGQRGA
GPWAQVPTSFSLSLEKDLQRKESVEWVNMVLVKLWKVYRGGIENWLVGLLQPVIDDLKKP
DYVQRVEIKQFSLGDEPLSVRNVERRTSRRVNDL**^ +2**
QYQIGLRYTGGARMLLMLSLKFGIIPVVVPVGIRDFDIDGELWVKLRLIPSAPWVGAASW
AFVSLPKIKFELAPFRLFNLM**^ +1**
GIPVLS**^ +2**
MFLTKLLTEDLPRLFVRPKKIVLDFQKGKAVGPVSEDLKSGEMQEGNKDFVGELSVTLVN
AQKLPYMFS**^ +1**
GRTDPYVILRIGDQVIRSKKNSQTTVIGAPGQPIWNQ**^ 0**
DFQFLVSNPREQVLQIEVNDCLGFADMAIGIGE**^ 0**
VDLESLPDTVPTDRFVSLRGGWSLFGKGSTGEILLRLTYKAYVEDEEDDKRNAKAIYADA
SDDEMSDSEEPSSFVQNDKIPSDDIGQESFMNVLSALILSEEFQGIVSSETGNNKVDDGE
SSVSPVPSMSGADSESRPKDAGNGDVSDLEVKNAKSDRGSINNG**^ +1**
GLALLWFGVITSVLVLVAINMGGSSFFNP

<u>ATG</u>ATTCTACAGTCTTCTTCTTCTTGTTCTAGTTTCGATTTCCCTTCTTTTGTGTCACGT
CGTCTGCTCTGTCCTTGCTCCAATGAGCACGGTCTGATTGTGTTCTCCGATGGATTCACA
AAGAGGCGGAGAATTCTCCGTCGAGTTCATGCGGCGAATTCGAATTCTAGGTTTGTTTCC
AGCGGAATTCGTACGGATTCCAAGAACATTGGATTAGCGGATTCGGCGAGAAGAGCTGCC
AGAAGTCTTGTGGTTACTCGGTTTTCGAATGAATTTGAGGATGAAGAAGCATCTTCATCT
TCACAGGAGTCTGCAATTCAAGGTGATAGAAACAATTTCACTAACTTTAGAGAAGATCCT

ATAGTGGATAAGCTCAGGACTCAGCTAGGTGTAATCCACCCAATCCCGTCTCCACCGATT
AGTCGTAATGCAATTGGTCTATTCGCATTCTTCTTCTTTGTTGGTGTTATTTGTGATAAG
CTATGGACTTGGAGAAAGAGGCGGAGACAAATGGCTGGTGATGGAGGCCAAAGAGGAGCC
GGGCCATGGGCACAGGTTCCCACCAGTTTCTCGCTGTCCCTTGAGAAGGATTTGCAAAGG
AAAGAGTCGGTGGAGTGGGTGAACATGGTGTTGGTTAAGCTCTGGAAAGTTTATAGAGGT
GGGATTGAGAATTGGCTTGTTGGATTGTTGCAGCCTGTGATTGATGATTTGAAGAAGCCT
GATTATGTGCAGAGAGTTGAAATTAAGCAATTCTCTCTTGGGGATGAGCCTTTGTCTGTT
AGAAATGTTGAGAGGAGGACATCTCGCCGTGTTAATGACTTGCA
phase +2
ab025631.1 (22749-21926)

GTACCAGATTGGTCTTCGGTATACTGGTGGTGCTCGGATGTTGTTAATGCTCTCTTTAAA
ATTTGGTATCATCCCAGTAGTTGTGCCAGTTGGTATTCGGGATTTTGACATTGACGGTGA
ACTATGGGTTAAATTAAGATTGATTCCGTCAGCGCCTTGGGTTGGAGCAGCATCATGGGC
GTTTGTTTCACTTCCAAAGATCAAATTTGAGCTTGCACCATTCCGATTGTTTAATCTAAT
GG
phase +1
ab025631.1 (21662-21421)

GAATTCCTGTTTTATCAAT
phase +2 (21294-21276)

GTTCTTGACCAAACTACTGACAGAAGATTTGCCTCGATTATTTGTACGCCCAAAGAAAAT
TGTCTTGGATTTCCAGAAGGGAAAAGCTGTTGGGCCTGTTTCAGAAGACTTAAAATCTGG
AGAAATGCAGGAAGGGAACAAGGATTTTGTAGGGGAACTGTCTGTTACTCTTGTAAATGC
CCAGAAACTTCCATACATGTTCTCTG
phase +1
ab025631.1 (21161-20956)

GTAGAACGGATCCATATGTTATTTTACGAATAGGTGATCAAGTTATCCGCAGTAAGAAGA
ACAGTCAAACTACTGTGATTGGGGCTCCTGGTCAGCCAATCTGGAACCAG
phase 0
ab025631.1 (20735-20626)

GACTTCCAATTCCTTGTGTCAAATCCTAGAGAGCAAGTATTACAAATTGAAGTCAACGAC
TGTCTTGGATTTGCCGATATGGCTATTGGTATCGGAGAG
phase 0
ab025631.1 (20513-20415)

GTTGACCTTGAATCACTGCCAGATACGGTTCCTACAGACAGATTTGTTAGTTTACGAGGC
GGTTGGAGTTTGTTCGGAAAGGGATCTACTGGAGAAATACTACTCCGGCTTACATACAAA
GCATACGTGGAGGATGAAGAAGATGATAAACGCAACGCAAAAGCCATATATGCAGATGCT
TCCGATGATGAAATGTCTGATTCGGAAGAACCTAGCTCATTTGTGCAGAATGACAAGATT
CCTTCAGATGATATTGGTCAAGAGTCATTTATGAACGTGTTGTCTGCATTGATTTTGAGC
GAGGAATTTCAAGGCATAGTTTCATCAGAAACTGGGAACAATAAAGTTGATGATGGTGAA
TCAAGCGTGTCACCTGTTCCTTCAATGTCTGGGGCGGACTCAGAATCTCGACCAAAAGAT
GCTGGCAATGGGGACGTATCAGATTTGGAAGTGAAAAATGCGAAATCTGATAGAGGTTCT
ATTAATAATGGAG
phase +1
ab025631.1 (19773-19281)

GATTAGCATTATTGTGGTTCGGTGTAATCACTTCTGTACTGGTGCTCGTTGCTATCAACA
TGGGCGGCTCAAGTTTCTTCAACCCGTGA
ab025631.1 (19201-19113)

Transcript
cb255017

Arabidopsis thaliana Full Length ***NTMC2Type6.1***
Gene 111 Craxton, M. BMC Genomics. 2004 Jul 6;5(1):43
bp806603
bp562487
bp809755
av821775
ay054685

ai992807
t20499
av555172
av815801
av782317
av558377

Arabidopsis thaliana Full Length ***NTMC2Type6.2***
Gene 112 Craxton, M. BMC Genomics. 2004 Jul 6;5(1):43
av784055
ay094435
av823134

Brassica napus fragment ***NTMC2Type1***
cd819983
cd820681
cn829985
cn830148
ay835402
cd814261
cn830147
cn829984

Brassica napus fragment ***NTMC2Type1***
bq791529 (Brassica rapa)
cd837444
cd827846
cd835330
cd826723
am061972 (Brassica oleracea)

Brassica napus fragment ***NTMC2Type2***
am061205 (Brassica oleracea)
cd832377

Brassica napus fragment ***NTMC2Type4***
am060612 (Brassica oleracea)
ay835401
co750065 (Brassica rapa)
co750142 (Brassica rapa)
cn729341
am058068 (Brassica oleracea)

Brassica napus fragment ***NTMC2Type4***
cd836600
cd836750
cx187933
cx195172

Brassica napus fragment ***NTMC2Type4***
cd836600
cd836750

Brassica napus fragment ***NTMC2Type4***
cv433984

Brassica napus fragment ***NTMC2Type6***
cd826176

Brassica napus fragment ***NTMC2Type6***
cd826959

Brassica oleracea fragment ***NTMC2Type6***
am061521

Bruguiera gymnorrhiza fragment ***NTMC2Type1***
bp949358

Bruguiera gymnorrhiza fragment ***NTMC2Type6***
bp952860

Bruguiera gymnorrhiza fragment ***NTMC2Type6***
bp942177

Citrus sinensis Full Length ***NTMC2Type1***

MGVISTIFGFCGFGVGISSGLVIGYFLFIYFQPTDVKNPEIRPLVERDSETLQQMLPEIP
LWVKCPDYDRVDWLNKFLELMWPYLDKAICKTAKNIAKPIIAEQIPKYKIESVEFETLTL
GTLPPTFQGMKVYVTDEKELIMEPCLKWAANPNVTIGVKAFGLKATVQVVDLQVFAQPRI
TLKPLVPAFPCFANIYVSLMEKPHVDFGLKLVGADLxVIPGLYRFVQELIKTQVANMYLW
PKTLEVPILDPSKAYRRPVGILHVKVVKAMNLKKKDLLGASDPYVKLKITEDKLPSKKTT
VKHKNLNPEWNEEYNFTVRDPESQAVELAVSNWEQVGKHDKMGMNVVPLKELTPEEPTVK
TLDLLKNMDLNDGQNEKSRGQLVVEFMYKPFKEEDLPKSFEESQTVQKAPENTPAGGGLL
VVIVHEAQDVEGKHHTNPYARILFRGEERKTKHVKKNRDPRWEEEFQFILEEPPTNDRLH
VEVCSVSSRIGLLHPKETLGYIDINLSDVVSNKRINEKYHLIDSKNGRIQIELQWRTA

ATGGGAGTTATCAGTACAATCTTCGGTTTTTGTGGATTTGGCGTCGGGATTTCGTCGGGG
CTTGTGATTGGTTACTTCCTTTTCATCTACTTTCAACCAACCGACGTGAAGAATCCTGAG
ATTCGCCCGTTGGTTGAGCGAGATTCTGAGACTCTGCAGCAGATGCTTCCAGAGATACCT
CTTTGGGTGAAATGTCCCGACTATGATCGCGTTGATTGGCTGAATAAGTTCCTTGAATTA
ATGTGGCCTTATCTTGACAAGGCTATTTGTAAGACTGCGAAGAATATTGCAAAGCCTATA
ATTGCAGAACAAATTCCTAAGTATAAAATCGAGTCTGTTGAATTTGAAACTCTTACATTA
GGGACTCTACCACCAACTTTTCAAGGTATGAAAGTTTATGTCACTGATGAGAAGGAGTTG
ATTATGGAACCATGCTTAAAGTGGGCTGCAAATCCTAATGTAACCATTGGTGTTAAAGCA
TTTGGTTTGAAGGCAACAGTTCAGGTGGTGGATTTGCAAGTTTTTGCTCAACCACGCATT
ACTCTGAAGCCCTTGGTCCCTGCTTTTCCTTGTTTTGCGAATATTTATGTGTCTCTCATG
GAGAAGCCTCATGTTGACTTTGGACTGAAGCTAGTAGGGGCTGATCTTATnGTCATTCCT
GGTCTCTATAGGTTTGTTCAGGAGCTTATCAAAACTCAGGTGGCCAACATGTACCTGTGG
CCCAAAACCCTAGAAGTACCAATTTTGGATCCATCCAAAGCTTACCGGAGACCTGTTGGA
ATTCTCCATGTGAAGGTTGTGAAGGCAATGAATCTGAAAAAGAAAGATCTTCTGGGTGCA
TCAGACCCTTACGTGAAACTAAAGATCACCGAGGATAAGCTTCCATCAAAGAAAACCACT
GTGAAGCACAAGAACTTGAACCCTGAATGGAATGAGGAATACAATTTTACTGTTAGAGAT
CCAGAGTCCCAGGCTGTAGAGCTTGCTGTGAGTAATTGGGAGCAGGTTGGCAAACATGAC
AAGATGGGCATGAATGTTGTTCCTTTGAAAGAACTTACCCCTGAAGAGCCCACAGTTAAG
ACTCTTGACCTTCTCAAAAACATGGATTTGAATGATGGTCAAAATGAGAAGTCGCGTGGA
CAGCTTGTGGTGGAATTCATGTATAAACCATTTAAAGAAGAGGACTTACCAAAAAGTTTT
GAAGAGTCGCAGACGGTGCAAAAGGCTCCTGAAAATACACCAGCTGGTGGAGGTTTGCTT
GTAGTTATAGTCCATGAAGCTCAAGACGTGGAAGGCAAGCACCACACTAATCCTTATGCA
CGGATTCTTTTCAGAGGGGAGGAGAGAAAAACTAAGCATGTAAAGAAGAATAGAGATCCC
AGATGGGAGGAGGAGTTTCAATTTATCCTAGAGGAGCCTCCCACTAATGATAGACTACAC
GTGGAAGTTTGCAGTGTCTCATCAAGGATTGGACTGCTGCATCCAAAGGAAACGCTAGGA
TATATTGACATCAATCTTTCAGATGTTGTTAGCAACAAGCGAATCAATGAGAAGTACCAC
CTCATTGACTCGAAGAACGGACGAATCCAGATCGAGTTGCAATGGCGAACTGCATAA

Transcripts
cf509149
cf510011
cv713291
cf417891
cx077271
cv713290
cb291203
cv887048
cv887049
cx305921 (Citrus reshni)
cx299600 (Citrus clementina)
cx077058

Citrus aurantium fragment ***NTMC2Type1***
dn623505

Citrus sinensis fragment ***NTMC2Type1***

cb291204

Citrus sinensis Full Length ***NTMC2Type2***

MGFVFGLVVGLVVGLGIIVGFVRSENARSKLRSELATTIAAFARMTVEDSKKILPAEFYP
SWVVFSHRQKLTWLNHHLEKLWPYVNEAASELIKSSVEPVLEQYRPFILSSLKFSKFTLG
TVAPQFTGVSIIEDGGSGVTMELEMQWDANSSIILAIKTRLGVALPVQVKNIGFTGVFRL
IFRPLVDEFPGFAAVSYSLREKKKLDFTLKVVGGDISTIPALSDSIEATIHDAIEDSITW
PVRKIVPILPGDYSELELKPVGTLEVKLVQAKGLTNKDLIGKSDPYAVLFVRPLPEKTKK
SKTINNDLNPIWNEHFEFIVEDESTQHLVVRIYDDEGIQSSELIGCAQVRLCELEPGKVK
DVWLKLVKDLDVQRDTKYRGQVHLELLYCPFGMENVFTNPFAPNFSMTSLEKVLTNGEKA
LKSGANGTEAIELEKDASQKRREVIIRGVLSVTVILAENLPASDLMGKADPYVVLTMKKS
ETRNKTRVVNDCLNPIWNQTFDFVVEDGLHDMLIAEVWDHDTFGKDYMGRCILTLTRVIL
EGEYTDCFELDGTKSGKLKLHLKWMPQPIYRDT

ATGGGATTTGTTTTCGGGCTAGTTGTCGGGCTAGTGGTCGGGCTTGGAATTATCGTCGGA
TTCGTGCGATCGGAGAATGCGCGATCCAAGCTCCGCTCTGAACTTGCAACAACAATAGCG
GCGTTTGCACGGATGACAGTGGAAGATTCTAAAAAGATCTTGCCGGCCGAGTTCTATCCA
TCTTGGGTGGTCTTCTCGCATCGCCAGAAGTTGACTTGGCTTAATCATCATCTTGAGAAG
CTCTGGCCCTATGTCAATGAGGCAGCTTCTGAGCTGATAAAGTCATCGGTAGAGCCAGTG
CTAGAACAATATAGGCCGTTTATATTGTCTTCTCTCAAGTTTTCCAAATTCACTCTTGGT
ACCGTTGCGCCCCAATTTACAGGAGTTTCAATTATTGAAGACGGAGGCAGTGGCGTTACC
ATGGAGTTGGAAATGCAGTGGGATGCTAATTCAAGTATAATACTTGCAATCAAGACCAGA
CTTGGGGTGGCGCTACCTGTGCAGGTGAAAAACATTGGATTCACTGGAGTTTTCAGATTG
ATCTTTAGGCCGCTAGTGGACGAATTTCCTGGCTTTGCAGCTGTTAGTTATTCTCTAAGA
GAGAAGAAAAAGTTGGATTTTACCTTAAAAGTTGTTGGTGGTGACATATCAACTATTCCT
GCACTTTCTGATTCTATTGAGGCAACCATACATGATGCAATTGAAGACTCAATCACTTGG
CCAGTTAGGAAAATTGTTCCTATTTTGCCTGGGGATTACAGTGAACTTGAGTTGAAGCCC
GTGGGGACATTAGAGGTGAAGCTTGTGCAAGCAAAGGGTTTAACCAATAAAGACCTGATT
GGGAAATCAGATCCCTATGCAGTATTATTTGTACGACCTCTACCTGAAAAAACGAAGAAA
AGTAAAACAATTAACAATGATTTAAATCCAATCTGGAATGAACACTTTGAATTTATTGTT
GAAGACGAATCCACTCAGCACTTGGTTGTAAGAATTTATGATGATGAAGGGATTCAGTCA
TCTGAATTAATTGGATGCGCTCAAGTACGCCTATGTGAACTTGAGCCTGGCAAAGTTAAG
GATGTATGGTTGAAGCTGGTTAAAGATTTGGATGTTCAAAGAGATACCAAATATAGGGGA
CAGGTGCACTTGGAACTCTTGTACTGTCCATTTGGAATGGAGAATGTGTTTACAAACCCT
TTTGCTCCCAATTTTTCAATGACCTCCTTGGAGAAGGTTCTTACAAATGGGGAGAAGGCT
CTTAAAAGTGGGGCAAATGGAACAGAAGCTATTGAACTTGAAAAAGATGCTTCTCAGAAG
AGAAGAGAGGTTATTATTAGAGGAGTACTTTCTGTTACAGTAATATTAGCTGAAAACTTG
CCTGCATCAGATTTGATGGGGAAGGCTGATCCCTATGTGGTGCTCACTATGAAGAAATCA
GAGACAAGAAACAAAACTAGAGTTGTGAATGACTGCCTGAATCCAATTTGGAATCAAACT
TTTGACTTTGTTGTTGAGGATGGATTGCATGACATGCTAATTGCTGAAGTATGGGATCAT
GACACATTTGGGAAGGACTACATGGGACGATGCATCTTGACATTGACAAGGGTTATATTG
GAAGGAGAATACACAGACTGCTTTGAGCTTGATGGAACTAAATCAGGAAAATTAAAGTTG
CACCTCAAGTGGATGCCGCAGCCTATTTATCGCGATACCTGA

Transcripts

ck932592
cx291088 (Citrus clementina)
cf504005
cf828669 (Citrus reticulata)
cn188042
cx672014
cx672293
cf837061
cf828670 (Citrus reticulata)
cv887167
cv887166

Citrus clementina x tangerina fragment ***NTMC2Type2***

cx298789

Citrus sinensis fragment ***NTMC2Type4***

cv712800
cx070520
cx077921
cx299124 (Citrus clementina x tangerina)

cx288655 (Citrus clementina)
cv712799
cx077920
cx070519
co912768 (Citrus jambhiri)
dn618664

Citrus sinensis fragment ***NTMC2Type4***
dn618665

Citrus medica fragment ***NTMC2Type5***
cx304507

Citrus sinensis x Poncirus trifoliata fragment ***NTMC2Type5***
cx297301

Citrus sinensis fragment ***NTMC2Type6***
cn188330
cx073480
cx073481

Citrus sinensis fragment ***NTMC2Type6***
cn184663

Cucumis melo fragment ***NTMC2Type1***
dv631413

Cucumis sativus fragment ***NTMC2Type2***
ck757075

Cucumis melo fragment ***NTMC2Type2***
dv633538

Cucumis sativus fragment ***NTMC2Type4***
cv005572

Cucumis melo fragment ***NTMC2Type6***
dv634002

Eucalyptus grandis fragment ***NTMC2Type4***
cb967970

Euphorbia esula fragment ***NTMC2Type1***
dv122512
dv130126
dv126620
dv132125

Euphorbia tirucalli fragment ***NTMC2Type1***
bp961933
bp961934
bp961935

Euphorbia tirucalli fragment ***NTMC2Type1***
bp953757

Euphorbia esula fragment ***NTMC2Type1***
dv129502

Euphorbia esula fragment ***NTMC2Type2***
dv141021
dv133497

Euphorbia esula fragment ***NTMC2Type2***
dv127801

Euphorbia esula fragment ***NTMC2Type2***
dv138851

Euphorbia esula fragment ***NTMC2Type2***
dv124603

Euphorbia esula fragment ***NTMC2Type3***
dv114672
dv113178

Euphorbia esula fragment ***NTMC2Type4***
bp957428 (Euphorbia tirucalli)
dv131733
dv130745
dv147540
dv127301

Euphorbia esula fragment ***NTMC2Type5***
dv153544

Euphorbia esula fragment ***NTMC2Type6***
dv130940

Fragaria vesca fragment ***NTMC2Type1***
dy673200
dy674275
dy669375
dy670220
dy674262

Glycine max Full Length ***NTMC2Type1.1***

MGILSTIASFFGFGVGTSIGLVIGYYLFIYFQPTDVKDPVIQPLVEQDAKTLQLLLPEIP
TWIKNPDYDRVYWLNKFILYMWPYLDKAICKTARSIAKPIIAEQIPKYKIDSVEFEELSL
GSLPPTFQGMKVYVTDEKELIMEPSVKWAGNPNIIVAIKAFGLRATVQVVDLQVFAAPRI
TLKPLVPSFPCFANIYMSLMEKPHVDFGLKLLGADAMSIPGLYRIVQAIIKDQVAKMYLS
PKALEVQIMDPTKAMKVPVGILHVKVVRAEKLKKKDLLGASDPYVKLKLTEEKLPSKKTT
VKYKNLNPEWNEEFNIVVKDPESQVLELTVYDWEQIGKHDKMGMNVIPLKEITPDEPKAV
TLNLLKTMDPNDPENAKSRGQLTVEVLYKPFKEDELPQSAEDSNAIEKAPEGTPASGGLL
VIIVHEAEDVEGKHHTNPYVRLLFKGEERKTKHVKKNRDPRWGESFQFMLEEPPTNERLY
VEVQSASSKLGLLHPKESLGYVDIKLSDVVTNKRINEKYHLIDSRNGRIQIELQWRTP

ATGGGAATTTTGAGTACTATTGCAAGCTTTTTTGGATTTGGAGTGGGAACTTCAATTGGG
CTAGTCATTGGCTACTATTTGTTCATATACTTCCAGCCAACAGATGTTAAGGATCCAGTA
ATTCAACCTTTGGTTGAGCAAGATGCTAAAACATTACAACTGTTGCTTCCAGAGATACCC
ACTTGGATTAAAAACCCAGACTATGACCGTGTATACTGGCTTAATAAATTTATATTGTAT
ATGTGGCCTTATCTGGACAAGGCAATTTGCAAGACTGCAAGGAGTATAGCAAAGCCCATT
ATTGCTGAGCAAATTCCGAAGTACAAAATTGATTCAGTTGAATTTGAAGAACTTAGCTTG
GGTTCTCTGCCACCAACTTTTCAAGGAATGAAAGTCTATGTGACTGACGAAAAGGAGTTG
ATTATGGAACCATCAGTGAAGTGGGCTGGGAATCCTAACATTATAGTTGCAATTAAAGCA
TTTGGGCTTCGAGCCACAGTCCAGGTTGTTGATTTGCAAGTATTTGCTGCTCCACGTATA
ACTCTGAAGCCTTTGGTTCCAAGTTTTCCATGCTTTGCCAATATTTACATGTCTCTCATG
GAGAAGCCACATGTTGATTTTGGACTAAAACTGCTTGGAGCTGATGCAATGTCTATTCCT
GGTCTTTATAGGATTGTCCAGGCAATCATTAAAGACCAAGTTGCAAAAATGTATCTGTCG
CCCAAGGCCTTAGAGGTTCAAATAATGGATCCAACAAAAGCCATGAAAGTGCCAGTTGGA
ATCCTTCATGTGAAAGTTGTAAGGGCAGAGAAGCTTAAAAAGAAAGATCTACTGGGGGCA
TCAGACCCTTATGTGAAACTCAAACTCACAGAAGAGAAACTTCCTTCAAAGAAAACAACT
GTGAAATACAAGAATTTGAATCCAGAATGGAATGAGGAATTTAATATAGTCGTCAAAGAT
CCAGAATCTCAAGTCTTAGAGCTTACTGTTTATGACTGGGAGCAGATTGGCAAGCATGAC

AAGATGGGTATGAATGTCATTCCATTGAAAGAGATTACACCTGATGAACCAAAAGCGGTG
ACTCTTAATTTACTCAAGACCATGGACCCTAATGATCCTGAGAATGCGAAGTCACGTGGG
CAGCTAACTGTTGAAGTTCTGTACAAACCTTTTAAGGAGGATGAGTTACCTCAGAGTGCA
GAGGATTCCAATGCAATAGAAAAGGCTCCTGAAGGAACACCTGCTAGTGGTGGTCTGCTT
GTAATTATTGTTCATGAAGCTGAAGATGTGGAAGGAAAACACCACACAAATCCATATGTG
CGACTGCTATTTAAGGGAGAGGAGAGAAAAACCAAGCATGTCAAGAAAAATAGAGATCCA
AGATGGGGTGAGTCGTTTCAATTTATGCTTGAGGAACCCCCCACCAATGAGAGACTATAT
GTTGAAGTGCAAAGTGCTTCCTCAAAGCTAGGCCTGCTCCATCCTAAGGAAAGTCTGGGT
TATGTGGATATAAAATTGTCTGATGTTGTTACCAACAAAAGAATCAACGAGAAATATCAT
CTCATTGACTCAAGAAATGGACGAATTCAAATCGAGCTTCAGTGGAGAACTCCTTGA

Transcripts

bu090353
bu090347
bi973812
bg316308
bq297869
bm188484
be806505
aw234507
aw234170
bi427401
be473678
bm177214
bu081213
bf009640
bu081600
bi316855
bm108017
cd417824
bg725175
bi968711
co985944
bi470301
ca801295
be022961
ai938430
cd414624
cd416624
aw309619
bm187893
bg840028
bg510329
bu763903
cd404182
cd418355
cd398783

Glycine max Full Length ***NTMC2Type1.2***

MGLVSSFLGILGFAVGIPLGLFVGFFLFVYSETKHVKDPVVRPISELGPNALQELLPEIP
LWVKTPDYERVFQVDWLNKFLLDTWPFLDTAICKIIRSRAQPIFFEYIGKYQIKAIEFDK
LSLGTLPPTVCGIKVLETNGKELVMEQVIKWAGNPEIVLSVYVASLKITVQLVDLQIFAA
PRVTLRPLVPTFPCFANIVVSLMEKPHVDFGMNVxGGDIMSIPGLYRFVQETIKKQVANL
YLWPQTLEIPILDESTVAIKKPVGILHVNVVRAQKLLKMDLLGTSDPYVKLSLTGDKLPA
KKTTVKRKNLNPEWNEKFKIVVKDPQSQVLQLQVYDWDKVGGHDKLGMQLVPLKVLNPYE
NKEFILDLLKDTNLNETPHKKPRGKIVVDLTFVPFKEDSxKFGGPSEGYSRKESGIDxVS
DDEVQEGAGLLSIVIQEAEEVEGDHHNNPFAVLTFRGEKKRTKxMKKTRHPRWNEEFQFM
LEEPPLHEKIHIEVMSKRKNFSFLPKESLGHVEINLRDVVHNGRINDKYHLINSRNGVMH
VEIRWKVV

ATGGGGTTGGTGAGCAGTTTCTTGGGGATTCTTGGTTTCGCCGTTGGAATACCTCTTGGC
CTCTTTGTGGGGTTCTTTCTCTTTGTCTACTCAGAAACCAAACATGTCAAGGACCCTGTT
GTTAGGCCTATAAGTGAATTAGGCCCAAATGCTTTGCAGGAACTTCTGCCCGAGATTCCT
CTCTGGGTGAAGACGCCTGATTATGAACGAGTTTTTCAGGTTGATTGGTTGAACAAGTTT
TTATTGGATACGTGGCCTTTCTTAGATACGGCAATTTGTAAGATTATTAGAAGCAGGGCT
CAACCAATATTTTTCGAATACATTGGCAAGTATCAGATCAAAGCGATTGAGTTTGATAAG
TTAAGCCTTGGTACTCTTCCTCCTACTGTCTGTGGTATTAAAGTTTTAGAAACAAATGGA
AAGGAATTGGTCATGGAACAAGTTATCAAATGGGCTGGCAATCCAGAAATAGTGTTGTCG
GTGTATGTGGCCTCTTTGAAGATCACCGTTCAGTTGGTAGACTTACAAATATTTGCAGCA
CCACGAGTAACTTTAAGACCTTTGGTGCCAACATTTCCATGTTTTGCAAATATTGTGGTA

TCTTTGATGGAGAAGCCTCATGTGGATTTTGGGATGAACGTATnAGGAGGGGACATCATG
TCAATACCTGGTCTCTATAGATTTGTACAGGAAACAATAAAAAAACAAGTTGCAAACCTC
TATCTCTGGCCTCAAACCCTGGAAATTCCTATTCTTGATGAATCAACGGTGGCAATAAAG
AAGCCTGTGGGGATATTACATGTGAATGTGGTTCGTGCACAAAAGCTTTTAAAGATGGAT
TTGTTGGGTACTTCTGATCCTTATGTTAAACTAAGCTTGACAGGAGACAAACTTCCAGCA
AAGAAAACCACTGTCAAGAGAAAGAATTTGAATCCTGAGTGGAATGAGAAGTTCAAGATT
GTTGTAAAGGACCCTCAATCTCAAGTTCTCCAATTACAAGTTTATGACTGGGACAAGGTT
GGTGGACATGACAAGCTGGGAATGCAGTTAGTCCCTCTTAAAGTGCTAAATCCGTACGAG
AACAAAGAATTTATACTTGATTTACTCAAGGACACAAATCTCAATGAAACTCCACATAAG
AAGCCTAGAGGGAAAATTGTGGTGGACTTGACTTTTGTTCCTTTCAAAGAAGATAGTAnC
AAGTTTGGTGGACCTTCAGAAGGATATAGCAGGAAGGAAAGTGGAATTGATATnGTATCn
GATGATGAGGTCCAAGAAGGAGCnGGTTTGCTTTCAATTGTGATCCAAGAGGCTGAAGAG
GTTGAGGGGGACCATCACAACAACCCATTTGCAGTnCTGACCTTTAGAGGnGAAAAGAAA
AGGACAAAGAnGATGAAGAAAACTCGACACCCGCGTTGGAATGAAGAATTCCAATTCATG
CTAGAGGAGCCTCCTCTACATGAGAAGATACATATTGAGGTTATGAGCAAGAGGAAGAAC
TTTAGTTTTCTGCCAAAGGAATCATTGGGGCATGTGGAGATTAACCTAAGAGATGTGGTG
CACAATGGTCGCATTAACGATAAGTACCATCTAATAAATTCAAGGAATGGAGTGATGCAT
GTTGAGATAAGATGGAAGGTGGTTTAA

Transcripts
ck605729
bu965179
co979674
bu761422
bm270667

Glycine max fragment ***NTMC2Type1***
bm886552
aw597099
aw597093
aw203783
dn590990
cn472511
aw472701
cd406212
bm886886
bi969163
co982863
be347521
bq741657
bu545983
aw317171
bi969221
bq613069
aw761518
bg508507
cd407492
cd408561

Glycine max fragment ***NTMC2Type1***
ai441145

Glycine max fragment ***NTMC2Type2***
bf424237
bq298236
aw202012
bi975059
bi784687
bm523821 (Glycine soja)
bu545280
co983793
aw395370
bi969868
aw831375
be556452
cd397450
co982407
bg507448
be555417
ai416733
bm270863

Glycine max fragment ***NTMC2Type2***
bu091691
bm107766
bg791051
ai416820
bi469113
be210000

Glycine soja fragment ***NTMC2Type2***
bf599015

Glycine max fragment ***NTMC2Type3***
cf805645
be584377
be805777
be804802
be806754
bi974788
bq080634
bf067073
bf008761
bq627818
bq628990

Glycine max fragment ***NTMC2Type3***
bu084207
bi497626
aw459033
bi471888
be801069
be820505
ca803088

Glycine max fragment ***NTMC2Type3***
ai901120

Glycine max fragment ***NTMC2Type3***
bi968143

Glycine max fragment ***NTMC2Type4***
bi469475
bm108106
bu081935
cb063409
bm731487
bf067278
ca801548
bf424371
ca785221
bf066277
bu084058
bu548941
bm178226
bm732781

Glycine max fragment ***NTMC2Type4***
bm731646
bi969161
aw201810
be022352
be611260
bu545393
aw396736

Glycine max fragment ***NTMC2Type4***
be822169
aw186503
bg352509

Glycine max fragment ***NTMC2Type4***
bm085597

Glycine max fragment ***NTMC2Type4***
bf067331

Glycine max fragment ***NTMC2Type4***
ca937917

Glycine max fragment ***NTMC2Type5***
co984610
ca800494
bf068579
bi893041
bi423934

Glycine max fragment ***NTMC2Type5***
aw349475

Glycine max fragment ***NTMC2Type5***
ai442053

Glycine max fragment ***NTMC2Type6***
bu550579
bf425755
be660539
aw831807
bm528774
bf325506
bm886373
be660538
bm094815
co981939
ca784865
bi974846

Glycine max fragment ***NTMC2Type6***
bi893199

Glycine max fragment ***NTMC2Type6***
be057962

Glycine max fragment ***NTMC2Type6***
bu760700

Gossypium raimondii Full Length ***NTMC2Type1***

MGVLSSVMGVFGFGIGTSVGIVIGYYMFIYFLPTDVKDPKIRPLVEEDSKTLQQLLPEIP
LWVKNPDFDRVDWLNKFIETMWPYLDTAICTTAKNIAKPIIDEQIPKYKIQSVEFETLTL
GTLPPTFQGMKVYVTDEKEIIMEPSLKWAGNPNIIIAVKAFGLKATVQVVDLQVFAVPRI
TLKPLLSVFPCFANIYVSLMDKPHVDFGLKLLGADVMAIPGLYRFVQELIKDQVANMYLW
PKALQVPIMDPTQAMKKPVGMLDVKVVKAMKLRKKDFLGKSDPYVKLKLTEEKLSAKKTT
VKQSNLNPEWNEEFSFVVKDPNTQALEIILYDWEQVGKHDKMGMNVVPLKDLTPEEPKVF
TLDLLKNMDPNDQQNEKSRGQLVLEAFYKPFKEDEMPNDVDDSTMVQKAPEGTPAGGGLL
VVIVHEAEDIEGKYHTNPHVRLLFRGEERKTKRVKKSRDPRWEEEFQFMVDDPPPNDKIH
MEAFSTSSRIGLLHPKEFLGYVTISLADVVNNRRINERYHLMDFKNGGIQIERQGRTF

ATGGGTGTTTTGAGCTCTGTAATGGGTGTTTTTGGATTCGGAATTGGAACTTCGGTTGGG
ATCGTCATTGGGTATTACATGTTCATCTACTTCCTTCCCACTGATGTTAAGGATCCTAAA
ATCCGTCCTTTGGTTGAGGAAGACTCTAAAACTTTGCAGCAGTTGCTTCCAGAGATCCCT
TTGTGGGTCAAAAATCCAGATTTCGACCGTGTTGACTGGCTTAACAAATTTATCGAGACC
ATGTGGCCTTATCTAGATACGGCGATTTGCACAACTGCTAAGAATATAGCAAAGCCCATT
ATTGATGAGCAAATTCCAAAATACAAAATTCAATCAGTTGAATTTGAAACACTTACTTTG
GGTACCCTGCCACCAACTTTTCAAGGAATGAAAGTCTATGTCACTGATGAGAAGGAAATT

ATTATGGAACCATCACTTAAGTGGGCAGGGAATCCTAACATCATTATCGCAGTTAAAGCT
TTTGGATTAAAAGCCACTGTTCAGGTTGTCGATTTGCAAGTTTTTGCTGTTCCTCGAATT
ACTCTGAAGCCTTTGCTCAGTGTCTTTCCTTGTTTTGCCAACATATATGTCTCCCTAATG
GACAAGCCACATGTTGACTTTGGGCTTAAGTTACTCGGGGCAGATGTTATGGCCATTCCT
GGATTATACAGGTTTGTCCAGGAGCTCATAAAAGATCAAGTTGCAAATATGTACCTATGG
CCTAAAGCCTTACAAGTGCCAATAATGGATCCTACACAAGCAATGAAGAAACCTGTTGGA
ATGCTTGATGTGAAGGTTGTCAAGGCAATGAAGCTTAGAAAGAAAGATTTCCTAGGCAAA
TCCGATCCTTACGTAAAACTAAAGTTAACAGAGGAAAAACTCTCTGCCAAGAAGACTACT
GTAAAACAAAGCAACTTGAACCCCGAATGGAATGAGGAATTTAGTTTTGTTGTTAAAGAT
CCAAACACTCAAGCGCTGGAGATAATTCTCTATGACTGGGAGCAGGTTGGCAAACATGAC
AAGATGGGCATGAATGTTGTTCCATTGAAAGATCTTACACCTGAAGAGCCAAAAGTTTTT
ACTCTTGATCTGCTGAAAAATATGGATCCAAATGATCAACAAAATGAGAAGTCACGGGGG
CAGCTTGTCTTGGAAGCCTTTTACAAACCTTTCAAGGAGGATGAGATGCCAAATGACGTT
GACGACTCAACTATGGTACAAAAGGCTCCAGAAGGAACACCTGCAGGTGGAGGTTTACTT
GTAGTTATTGTGCATGAAGCTGAAGATATCGAAGGGAAGTACCACACAAACCCACACGTG
CGACTGTTATTCAGAGGGGAGGAGAGAAAAACCAAGCGTGTAAAGAAAAGCAGAGATCCG
AGATGGGAAGAAGAATTTCAGTTTATGGTGGATGACCCCCCCCCGAATGATAAAATTCAC
ATGGAAGCGTTCAGTACATCCTCCAGGATAGGGTTACTGCATCCCAAGGAATTTTTGGGT
TATGTTACTATAAGCCTAGCGGATGTTGTGAATAACAGAAGAATCAATGAAAGGTATCAT
TTGATGGACTTTAAGAATGGTGGGATTCAAATCGAGAGGCAGGGGAGAACTTTTTAG

Transcripts
co127632
co073007
co129113
ai731309 (Gossypium hirsutum)
dr452618 (Gossypium hirsutum)
dr458757 (Gossypium hirsutum)
ai729075 (Gossypium hirsutum)
ai055308 (Gossypium hirsutum)
co499384 (Gossypium hirsutum)
co124382
dv850257 (Gossypium hirsutum)
dv848807 (Gossypium hirsutum)
co124383
co127633
co073006
co100780
co129112

Gossypium arboreum fragment ***NTMC2Type1***
co072050 (Gossypium raimondii)
BQ415280
bq406665
bq415280
BQ406665

Gossypium hirsutum fragment ***NTMC2Type1***
ai731712

Gossypium raimondii fragment ***NTMC2Type1***
co072051

Gossypium raimondii fragment ***NTMC2Type2***
co096370
co070394
co122440
co070393
dn817719 (Gossypium hirsutum)

Gossypium raimondii fragment ***NTMC2Type2***
co081749
co096369
co081748

Gossypium hirsutum fragment ***NTMC2Type3***
ca993639
ca993318

Gossypium hirsutum Full Length ***NTMC2Type4***

MGLISGILLGIIFGISLMAGWRHMMRYRSTKRIAKAADIKVLGALNRDDLKKICGDNFPE
WISFPVYEQVKWLNKQLSKLWPSVAEAASAVIKESVEPLLEEYRPPGITSLKFSKLSLGT
VAPKIEGVRVQSLKKGQITMDIDFRWGGDPSIILGVEAALVASIPIQLKDLQVFTVIRVI
FQLAEEIPCISAVVVALLSEPKPRIDYTLKAVGGSLTAIPGISDMIDDTVKTIVTDMLQW
PHRIVVPIGGIPVDTSELELKPEGRLTVTVVKANDLKNMEMIGKSDPYVVVYIRPLFKVK
TKVIDNNLNPVWNQTFELIAEDRETQALTVEVFDQDIGQDKRLGIAKFRLIELEPETPKE
ITLNLLSSLDTLKVKDKKDRGNCTIKLLYHQFNKEEQLIALEEEKRILEERKKLKEAGVI
GSTMDALDGAASLVGSGVGMVGTGIGAGVGLVGSGVGAGVGIVGSGLGAVGSGLSKAGKF
MGRTFTGHSSKRSGSSTPVNSIxENGGAKPL

ATGGGGCTGATTTCGGGGATTTTGCTGGGGATAATCTTCGGGATTTCATTGATGGCTGGC
TGGCGTCATATGATGAGGTACCGAAGCACCAAGAGAATCGCCAAGGCAGCTGATATAAAA
GTTCTTGGAGCTCTCAACAGGGATGATCTTAAGAAAATTTGCGGCGATAATTTTCCTGAA
TGGATATCTTTTCCTGTCTATGAACAGGTGAAGTGGCTGAACAAGCAATTGAGCAAATTA
TGGCCTTCTGTAGCAGAGGCAGCATCAGCAGTCATCAAAGAATCTGTTGAACCACTCTTG
GAAGAATATCGACCTCCAGGAATTACTTCACTGAAGTTTAGCAAATTGTCTCTCGGCACT
GTTGCTCCCAAGATTGAAGGTGTTCGTGTTCAGAGCCTTAAGAAAGGTCAAATCACAATG
GATATTGATTTCCGGTGGGGTGGTGATCCAAGTATAATTTTAGGTGTTGAAGCAGCACTT
GTTGCTTCAATACCAATTCAGTTAAAGGATCTTCAAGTTTTCACTGTTATTCGTGTCATC
TTCCAGCTTGCTGAAGAGATACCTTGTATTTCTGCTGTAGTCGTTGCTCTCCTTTCTGAG
CCCAAGCCTAGAATTGATTACACTCTGAAGGCTGTTGGTGGAAGCTTAACAGCAATTCCA
GGAATTTCAGATATGATTGATGATACTGTGAAGACAATTGTCACAGACATGCTTCAGTGG
CCACATAGAATTGTTGTTCCAATTGGTGGTATACCAGTTGATACAAGTGAATTAGAGCTG
AAACCGGAGGGAAGGCTGACAGTTACAGTAGTCAAAGCTAATGATTTGAAGAACATGGAA
ATGATTGGAAAGTCTGATCCTTATGTGGTTGTATACATTCGACCTTTGTTCAAGGTTAAA
ACAAAAGTCATCGACAACAACCTGAATCCTGTTTGGAATCAAACTTTTGAGTTGATTGCA
GAAGATAGAGAGACACAGGCACTAACTGTAGAGGTTTTTGACCAGGACATTGGGCAAGAC
AAGAGATTAGGAATTGCAAAATTCCGTTTGATTGAATTGGAACCTGAGACACCAAAGGAG
ATTACTCTGAATCTTCTATCCTCACTCGATACACTTAAAGTAAAAGATAAGAAGGACAGA
GGAAACTGCACCATTAAGCTTTTGTACCATCAGTTCAACAAGGAGGAACAACTGATTGCT
TTAGAGGAAGAAAAGAGGATCCTCGAAGAAAGAAAGAAATTGAAAGAAGCTGGAGTTATA
GGAAGCACAATGGATGCACTTGATGGAGCAGCGTCGTTGGTTGGATCTGGnGTTGGAATG
GTTGGTACCGGTATCGGTGCTGGAGTTGGACTCGTGGGAAGTGGTGTTGGTGCTGGAGTT
GGGATTGTTGGGAGTGGCCTTGGAGCTGTTGGnAGTGGACTGAGCAAAGCnGGAAAATTC
ATGGGCAGGACCTTTACGGGGCATTCCAGCAAGAGAAGTGGAAGCTCAACTCCGGTGAAT
AGCATCCAnGAAAATGGTGGTGCAAAGCCACTTTAA

Transcripts
dt547442
dt547441
dt566894
bf278067 (Gossypium arboreum)
bf278068 (Gossypium arboreum)
ai727727
ai727644
co094847 (Gossypium raimondii)
dt551454
dr455836
co097975 (Gossypium raimondii)
co491738
co089795 (Gossypium raimondii)
dt545134
co093912 (Gossypium raimondii)
co108717 (Gossypium raimondii)
bq404675 (Gossypium arboreum)
bq403513 (Gossypium arboreum)
dr453198
bq412521 (Gossypium arboreum)
ai726134

Gossypium hirsutum fragment ***NTMC2Type4***
dt572296
dt572293
dr455888
co093619 (Gossypium raimondii)

dt556924
dn817058

Gossypium hirsutum fragment ***NTMC2Type4***
dt546437
dt573805

Gossypium raimondii fragment ***NTMC2Type4***
co093911
co097974
co094846
co108716

Gossypium raimondii fragment ***NTMC2Type4***
co111314
bq402997 (Gossypium arboreum)

Gossypium raimondii fragment ***NTMC2Type4***
co111311
bg441673

Gossypium arboreum fragment ***NTMC2Type4***
bg443557

Gossypium arboreum fragment ***NTMC2Type4***
bg443771

Gossypium arboreum fragment ***NTMC2Type4***
bg441924

Gossypium arboreum fragment ***NTMC2Type4***
be053227

Gossypium raimondii fragment ***NTMC2Type6***
bg441908 (Gossypium arboreum)
co114486
co130031
co124963
dt467397 (Gossypium hirsutum)
co103304
co130030
co112270
co124964
co085859
co114485

Gossypium raimondii fragment ***NTMC2Type6***
co112271

Linum usitatissimum fragment ***NTMC2Type1***
ca482644

Linum usitatissimum fragment ***NTMC2Type2***
cv478854

Lotus corniculatus fragment ***NTMC2Type1***
av780095

Lotus corniculatus fragment ***NTMC2Type1***
ai967781

Lotus corniculatus fragment ***NTMC2Type1***
aw719889

Lotus japonicus fragment ***NTMC2Type2***
ap007807.1 genomic exon coordinates (5304-5116) (4838-4725) (3733-3605)
ap007851.1 genomic exon coordinates (301-113)

Transcripts
au251851
au251725
bp060167 (Lotus corniculatus)

Lotus japonicus fragment ***NTMC2Type2***
ap004498.1 genomic exon coordinates (4332-3951) (3333-3209) (2841-2740) (2245-2171) (1073-994) (92-1)
ap007851.1 genomic exon coordinates 80843-81224, 81842-81966, 82334-82435, 82930-83004, 84102-84181, 85083-85233, 85338-85568, 87292-87588, 87850-87963
ap007912.1 genomic exon coordinates (12346-12245)

Lotus japonicus fragment ***NTMC2Type2***
ap007851.1 genomic exon coordinates 6753-6899

Transcript
av779131

Lotus japonicus fragment ***NTMC2Type2***
av768158

Lotus corniculatus fragment ***NTMC2Type3***
cb827513
bp064188
bp075581

Lotus corniculatus fragment ***NTMC2Type3***
av410323
av777557

Lotus corniculatus fragment ***NTMC2Type3***
av425891

Lotus japonicus fragment ***NTMC2Type4***
av778245

Lotus japonicus fragment ***NTMC2Type4***
ap007851.1 genomic exon coordinates 42303-42441, 42558-42788, 44193-44492, 44762-44875, 45819-45944
ap007807.1 genomic exon coordinates 45656-45806, 45912-46142 (77638-77339) (77069-76956) (76011-75886)

Lotus japonicus fragment ***NTMC2Type4***
ap007851.1 genomic exon coordinates 40856-41029

Lotus japonicus fragment ***NTMC2Type4***
ap007851.1 genomic exon coordinates (8725-8504)

Lotus japonicus fragment ***NTMC2Type4***
ap007851.1 genomic exon coordinates 60073-60186

Lotus japonicus fragment ***NTMC2Type4***
ap007851.1 genomic exon coordinates (32958-32650)

Lotus japonicus fragment ***NTMC2Type4***
ap007851.1 genomic exon coordinates 2414-2527

Lotus japonicus fragment ***NTMC2Type4***
ap007807.1 genomic exon coordinates 42690-42941, 43198-43311

Lotus corniculatus fragment ***NTMC2Type5***
bp032367
bp056222
bp083845
bp084517 (Lotus japonicus)
bp084253 (Lotus japonicus)
bp046317 (Lotus japonicus)

Lotus corniculatus fragment ***NTMC2Type5***
bp032367
bp056222
bp046317

Lotus japonicus fragment ***NTMC2Type5***
bp074366

Lupinus albus fragment ***NTMC2Type4***
ca409762

Lupinus luteus fragment ***NTMC2Type6***
bg104137

Malus x domestica Full Length ***NTMC2Type1***

MGILSAILGFVGFGVGTSTGLVIGYYVFIYFLPTDVKNPAIRPLVEQDSKTLQRLLPEIP
MWVKNPDYDRVDWLNKFIELMWPYLDKAICKTARTIAKPIIAEQIPKYKIDSVEFEALSL
GTLPPTFQGIKVYVTGEKELIMELQLKWAGNPNILGLTKAFGLEATVQVVDLQVFACPRI
TLKPLVSAFPCFSKIFVSLMEKPHVDFGLKLLGADAMAIPGLYRFVQELIKEQVANMYLW
PKALEVQIMDPTMAMRKPVGILHIKVLKAMKLKKKDFLGGADPYVKIKLTEDKLPSKKTT
VKHGTLNPEWNEEFNFVVRDPVTQALEFMVYDWEKIGKHDKMGMNVIPLKELTPEEPKVM
TLEVLKNMDPNDAQNEKSRGQLVVELIYKPFTEDEMPKDDEDPKTIVKAPEGTPATGGVL
VIIVHEAEDVEGKHHTNPQVRILFRGEEKRTKLVKKNRDPRWEEEFQFILEEPPKKERLH
AEVVSHSSRMGLLHPKETLGYVDIDLADVVSNRRINEKYHLIDSKNGRLQLELQWRTAS

ATGGGTATTCTGAGTGCGATTTTGGGTTTTGTGGGTTTTGGAGTTGGAACTTCAACTGGG
CTTGTGATCGGGTATTACGTCTTCATCTACTTCCTGCCAACTGATGTCAAGAATCCTGCA
ATTCGTCCATTGGTCGAGCAAGATTCGAAAACTCTGCAGAGGCTGCTTCCGGAGATACCC
ATGTGGGTGAAAAATCCAGACTACGATCGTGTTGACTGGCTCAACAAATTTATTGAGCTC
ATGTGGCCTTACCTGGACAAGGCAATTTGCAAAACTGCGAGGACCATAGCAAAACCCATT
ATTGCTGAGCAAATTCCGAAATACAAAATTGACTCCGTCGAGTTTGAAGCTCTTTCCTTG
GGCACCCTACCGCCAACTTTTCAAGGAATTAAAGTGTATGTTACCGGTGAGAAGGAATTG
ATAATGGAGCTACAACTGAAGTGGGCAGGCAATCCTAACATCCTTGGTTTAACTAAAGCA
TTTGGGTTGGAAGCCACGGTTCAGGTGGTTGATTTGCAAGTGTTTGCTTGTCCACGTATC
ACCCTGAAGCCTTTGGTTTCAGCCTTTCCTTGTTTTTCAAAAATCTTTGTCTCTCTTATG
GAGAAGCCACATGTTGACTTTGGATTAAAACTGCTTGGAGCAGATGCTATGGCTATTCCT
GGCCTGTATAGGTTTGTCCAGGAGCTTATTAAAGAACAAGTGGCAAACATGTACCTATGG
CCCAAAGCCCTAGAAGTACAAATCATGGATCCCACAATGGCTATGAGGAAGCCTGTTGGG
ATTTTGCATATAAAAGTTCTTAAAGCAATGAAGCTTAAAAAGAAAGATTTTCTAGGAGGA
GCAGATCCATATGTGAAAATCAAGCTCACTGAGGACAAGCTTCCTTCAAAGAAAACAACT
GTGAAACATGGCACTTTGAACCCTGAATGGAATGAAGAGTTTAATTTCGTTGTCAGAGAC
CCGGTGACTCAAGCATTAGAATTTATGGTTTATGATTGGGAAAAGATTGGCAAACATGAC
AAGATGGGTATGAATGTCATTCCACTGAAAGAACTTACGCCTGAAGAGCCTAAAGTTATG
ACTCTTGAAGTGCTGAAGAACATGGACCCGAATGATGCTCAAAATGAGAAATCACGTGGA
CAGCTTGTTGTGGAATTGATCTATAAACCCTTTACGGAGGATGAGATGCCTAAAGATGAC
GAAGATCCAAAAACTATAGTAAAGGCTCCTGAAGGAACACCTGCAACTGGTGGTGTGCTT
GTAATTATCGTCCACGAAGCTGAAGATGTGGAAGGAAAGCACCACACTAACCCACAAGTG
CGCATACTTTTCAGGGGCGAAGAGAAAAGAACCAAGCTTGTCAAGAAAAATCGGGATCCA
CGATGGGAAGAGGAGTTTCAGTTTATATTGGAAGAGCCACCGAAAAAGGAGAGGCTTCAT
GCTGAAGTGGTTAGCCACTCCTCAAGGATGGGGCTGTTGCATCCCAAGGAAACTCTTGGA
TACGTGGACATAGACTTGGCGGATGTTGTTAGCAACAGAAGAATCAATGAGAAATACCAT
CTGATAGACTCGAAGAATGGACGGCTTCAACTCGAGCTGCAATGGAGAACAGCTTCTTGA

Transcripts
dt000779

cn896852
cx022651
cn914741
cn932639
cv883302
cn889720
cn927920
cn880685
cn931616
cv880649
cn995293
co722270
cn934939

Malus x domestica fragment ***NTMC2Type1***
cn880034
cn880042

Malus x domestica fragment ***NTMC2Type1***
cv082810

Malus x domestica fragment ***NTMC2Type1***
cn928693

Malus x domestica fragment ***NTMC2Type1***
cv793790

Malus x domestica fragment ***NTMC2Type1***
cn916669

Malus x domestica fragment ***NTMC2Type1***
co753699

Malus x domestica fragment ***NTMC2Type1***
cn913405

Malus x domestica Full Length ***NTMC2Type2***

MAFVFGLVLGILVGLAIIVIFVRFENHRSKLRSELATTVAAFARMTVEDSRKLLPPQYYP
SWVVFSHHQKLGWLNSHLEKMWPYINEAASELIKTSVEPMLEQYRPVILSSLKFSKFTLG
TVAPQFTGVSIIEDGSDGITMELEMQWDGNPSIILAIKTLVGVALPVQVKDIGFTGVFRL
IFKPLVDEFPCFGAVCYSLRNKKKLDFKLKVVGGDISTLPGISDAIEGTIRDAVEDSITW
PVRKIFPILPGDYSDLELKPVGVLEVKLVQAKELTNKDMVGKSDPFARLYIRPLPDRMKR
SKTINNDLNPIWNEHFEFIVEDETTQHLVVKVYDDEGLQASELIGCAQVQLSELEPGKVK
DVWLKLVKSLDLQRDNKNRGQVHLELLYCPFGMENGFANPFTSNSAMTSLEKALKSEMNG
ANATESEKEAAQKRREVIIRGVLTITIISAEDLPPVDLMGKADPYVVLTLKKSGAKNKTR
VVNDNLNPVWNQTFDFVVEDGLHDMLILEVWDHDTFGKDYMGRCILTLTRVILEGEYQDS
IPLDGAKSGKLNVHLKWNAQPIYRES

ATGGCCTTCGTTTTCGGATTAGTGCTCGGAATACTTGTCGGGCTCGCAATCATCGTCATC
TTTGTTCGGTTCGAGAACCACCGGTCCAAGCTCCGGTCCGAGCTCGCCACCACTGTGGCT
GCTTTTGCTCGAATGACCGTCGAGGATTCCAGAAAGCTCTTGCCTCCTCAGTATTACCCT
TCCTGGGTCGTCTTCTCTCACCACCAGAAGTTGGGATGGCTCAATTCTCACCTCGAAAAG
ATGTGGCCTTATATCAATGAGGCAGCGTCTGAGCTGATAAAGACTTCAGTGGAGCCAATG
CTGGAGCAGTATAGACCAGTCATATTGTCCTCGCTCAAATTTTCCAAGTTTACTCTTGGT
ACGGTGGCACCTCAGTTTACAGGGGTTTCTATAATTGAAGATGGAAGTGACGGTATCACT
ATGGAGTTGGAAATGCAGTGGGATGGAAATCCAAGTATAATACTTGCTATCAAGACTTTA
GTTGGTGTAGCACTACCGGTGCAGGTGAAAGATATTGGATTCACGGGGGTATTCAGGCTG
ATTTTTAAGCCTCTGGTTGACGAGTTTCCGTGTTTTGGAGCTGTTTGCTATTCATTGAGA
AATAAGAAAAAGTTAGATTTTAAGCTTAAGGTTGTGGGTGGCGACATATCTACATTGCCT
GGGATATCCGATGCAATTGAGGGGACCATACGGGATGCTGTTGAGGATTCAATTACATGG
CCTGTTCGGAAGATTTTTCCCATTTTGCCCGGGGATTACAGTGATCTGGAGTTAAAGCCA
GTCGGAGTGCTAGAGGTGAAACTTGTGCAGGCAAAGGAGTTAACAAATAAAGACATGGTG
GGAAAATCAGATCCCTTTGCAAGACTGTACATACGACCTTTACCAGACAGAATGAAAAGA
AGCAAAACAATTAACAACGATTTGAATCCCATCTGGAATGAGCACTTTGAATTTATAGTT

GAAGATGAAACGACTCAACATTTGGTGGTTAAAGTTTATGATGATGAGGGACTTCAGGCG
TCTGAGCTCATTGGATGTGCTCAAGTACAACTGAGTGAACTTGAGCCTGGTAAAGTGAAG
GATGTATGGTTGAAGCTGGTTAAAAGCTTGGATCTCCAAAGAGATAATAAAAACCGCGGG
CAGGTGCATTTGGAGCTGTTATATTGTCCATTTGGCATGGAGAATGGCTTTGCTAATCCT
TTTACTTCCAACTCCGCAATGACCTCTTTGGAAAAGGCACTTAAAAGTGAAATGAACGGA
GCAAATGCTACTGAAAGTGAAAAGGAAGCCGCACAGAAGAGAAGGGAGGTCATAATTAGA
GGTGTACTAACTATTACTATAATATCTGCTGAAGACTTGCCACCTGTTGATCTGATGGGG
AAGGCTGATCCTTATGTGGTGCTCACCTTGAAGAAATCAGGAGCGAAAAACAAAACTAGG
GTTGTTAATGATAACTTGAATCCTGTCTGGAATCAAACTTTCGACTTTGTTGTCGAGGAT
GGATTGCATGACATGCTCATACTTGAAGTCTGGGACCACGACACTTTTGGCAAGGACTAT
ATGGGCAGATGCATCTTGACCCTCACTAGGGTCATCTTAGAAGGGGAATACCAAGACTCG
ATACCGCTAGATGGAGCCAAATCTGGGAAATTGAACGTGCATCTTAAGTGGAATGCCCAA
CCAATTTACCGCGAGTCTTGA

Transcripts
cn896908
cn890870
cn893218
cn901580
cn893586
cn895364
cn934194
cn899522
cn927801
cn929162
cn900031
cn878045
cn903998
cv880014
cv656418
cv997627
dt001114

Malus x domestica fragment ***NTMC2Type2***
cn994574

Malus x domestica fragment ***NTMC2Type3***
cn900313

Malus x domestica fragment ***NTMC2Type3***
cn918059

Malus x domestica fragment ***NTMC2Type3***
cn948926

Malus x domestica fragment ***NTMC2Type4***
co723281
cv881006
co865171
dt043090
cn919599
cn940843

Malus x domestica fragment ***NTMC2Type4***
eb148205
cv656024

Malus x domestica fragment ***NTMC2Type5***
cn892605
cn891267
cn891295

Malus x domestica fragment ***NTMC2Type5***
cn896640

Malus x domestica fragment ***NTMC2Type6***

cn491956
cn997680
dr992167
cv882192

Malus x domestica fragment ***NTMC2Type6***
cx022786
cn880229
cn873113

Malus x domestica fragment ***NTMC2Type6***
cv631157
cv631578
cv656817

Malus x domestica fragment ***NTMC2Type6***
cn890174

Malus x domestica fragment ***NTMC2Type6***
cn996448

Manihot esculenta fragment ***NTMC2Type3***
ck641668

Manihot esculenta fragment ***NTMC2Type4***
dr086928
dr086622

Medicago truncatula Full Length ***NTMC2Type1.1***

MGFFSTIFGFFGFGVGISIGLVVGYFLFIYFQPTDVE**^ 0**
DPKITPIVDQDDETLQKMLPEIPNWIKNPDFDR**^ 0**
VDWLNKFIELMWPYLDK**^ 0**
AICKTAKNIAKPIIEEQIPKYKIDSVEFQTLTLGTLPPTFQ**^ +1**
GMKVYVTDEKELIMEPSIKWAGNPNVTIAVKAFGLKATVQ**^ 0**
VVDLQVFLLPRITLKPLVPSFPCFANIYVALMEK**^ 0**
PHVDFGLKLLGADLMSIPGVYRIVQ**^ 0**
ELIKDQVANMYLWPKNLEVQILDMA**^ +2**
KAMRRPVGILHVKVLHAMKLKKKDLLGASDPYVKLKLTDDKMPSKKTTVKHKNLNPEWNE
EFNLVVKDPETQVLQLNVYDWEQ**^ 0**
VGKHDKMGMNVITLKEVSPEEPKRFTLDLLKTMDPNDAQNEKSRGQIVVEVTYKPLNEEE
MGKGFDETQTIPKAPEGTPAGGGQLVVIVHEAQDVEGKHHTNPQARLIFRGEEKKTK**^ 0**
RIKKNRDPRWEDEFQFIAEEPPTNDKLHVEVVSSSSRTLLHQK**^ 0**
ESLGYVDINLGDVVSNKRINEKYHLIDSKNGRIQVELQWRTA

ATGGGTTTCTTCAGTACAATTTTTGGTTTTTTCGGATTTGGTGTTGGAATTTCAATTGGT
CTCGTTGTTGGTTATTTTCTCTTCATCTACTTTCAACCTACAGATGTTGAG
phase 0
ac121235.20 84585-84695

GATCCTAAGATTACGCCAATAGTGGACCAAGACGATGAGACTTTGCAAAAAATGCTTCCT
GAGATTCCCAATTGGATTAAAAATCCTGACTTTGATAG
phase 0
ac121235.20 84817-84914

GGTTGATTGGTTGAACAAGTTTATAGAACTTATGTGGCCTTATCTTGATAAG
phase 0
ac121235.20 85191-85242

GCGATTTGTAAGACCGCGAAGAACATTGCGAAACCCATAATTGAAGAGCAGATTCCAAAA
TATAAAATTGATTCTGTTGAGTTTCAGACGCTCACTCTCGGGACCCTGCCCCCAACTTTT
CAAG
phase +1
ac121235.20 85360-85483

GAATGAAAGTTTATGTGACTGATGAGAAGGAGTTGATTATGGAGCCTTCTATAAAATGGG
CTGGAAATCCCAATGTCACCATTGCTGTTAAGGCATTTGGGTTGAAAGCAACCGTTCAG
phase 0
ac121235.20 85960-86078

GTTGTGGATTTGCAAGTTTTCTTTTGCCTCGCATTACATTGAAGCCTTTGGTTCCAAG
CTTTCCTTGCTTTGCCAACATCTATGTCGCTCTCATGGAAAAG
phase 0
ac121235.20 86169-86270

CCACATGTTGACTTTGGGCTAAAGCTTTTAGGGGCTGATCTTATGTCTATTCCTGGTGTC
TACAGGATTGTTCAG
phase 0
ac121235.20 87528-87602

GAGCTTATCAAAGATCAGGTTGCAAACATGTATCTGTGGCCCAAAAACCTGGAAGTTCAA
ATATTAGATATGGCAAA
phase +2
ac121235.20 88513-88589

AGCTATGAGGAGGCCCGTTGGAATTTTACATGTAAAGGTTCTGCATGCTATGAAGTTAAA
GAAGAAAGATCTTCTGGGTGCATCTGATCCTTACGTGAAACTAAAGCTTACTGACGATAA
GATGCCATCAAAAAAGACTACTGTAAAGCACAAGAACTTAAATCCTGAATGGAATGAAGA
ATTTAATTTGGTAGTCAAAGACCCGGAGACCCAGGTTTTACAGCTCAATGTTTACGACTG
GGAGCAG
phase 0
ac121235.20 88702-88948

GTTGGGAAGCATGACAAGATGGGTATGAATGTGATCACTTTAAAAGAAGTTTCCCCTGAA
GAACCTAAACGTTTTACTCTGGACCTTCTAAAAACTATGGATCCCAATGATGCTCAAAAT
GAGAAATCGCGAGGACAGATTGTTGTGGAAGTGACTTATAAACCCTTAAATGAAGAGGAG
ATGGGCAAGGGCTTTGATGAGACACAGACAATTCCTAAAGCTCCCGAAGGCACTCCAGCT
GGTGGAGGTCAACTTGTAGTTATAGTCCATGAAGCTCAAGATGTTGAGGGGAAGCATCAC
ACTAATCCACAAGCACGTCTTATTTTCAGAGGGGAAGAGAAAAAGACTAAG
phase 0
ac121235.20 89033-89383

CGCATAAAAAAGAACAGAGATCCAAGATGGGAAGATGAGTTCCAATTTATAGCAGAGGAG
CCTCCTACCAATGATAAACTACATGTGGAGGTTGTCAGCAGTTCCTCACGAACCTTGCTT
CATCAAAAG
phase 0
ac121235.20 90641-90769

GAATCACTGGGTTATGTCGACATCAATCTCGGGGATGTTGTTTCTAACAAGAGAATCAAC
GAGAAGTACCATCTTATAGATTCTAAGAATGGTCGCATCCAGGTAGAGTTGCAGTGGAGA
ACTGCATAA
ac121235.20 91722-91850

Transcripts

aw690536
cx527935
aw773820
aw692689
dw019061
aw691313
bi270713
bi311918
bf648865
bg581161
ca857899
bf521449
ca919044
bi270554
cx527946
al384604
bi272892

bm814592
al384605
be239647

Medicago truncatula Full Length ***NTMC2Type1.2***

MSILSTIASFLGFGIGTSLGLLIGYFMFIYFESIDVKDPTFTPLVEQEAKTVQQLLPEIP
LWIKNPDYDRLDWLNKFVECMWPYLNKAICKTTRTIAKPIIAEQIPKYKIDSVEFEELNL
GSLPPTFQGMKVYSTDEKELIMELSMKWAGNPNIIVAVKAFGLRATVQVVDLQVFASPRI
MLKPLVPSFPCFANIYVSLMEKPHVDFGLKLLGADAMSIPGLYRIVQEIIKDQVAKMYLW
PKALQVQIMDPSQAMKKPVGILHVKILKAVKLRKKDIMGGADPYVKLKLKDDKLASKKTT
VKYKNLNPEWNEEFNVVIKDPESQDLMLNVYDWEQFGKAEKMGMNVIPVKELTPNEPKLL
LLKLLKTLVPNDPENEKSRGELIVEVMYKPFKDDEVSKNSEDTEKAPEGTPASGGLLLIS
IHEAEDVEGKHHTNPFARLIFKGEERKTKHVRKNRDPRWGETFQFTLEEPPINERLYVEV
ISASSKLGLLHPKETLGYVDINLSDVVSNKRINEKYHLIDSKNGKIQIELQWRTP

ATGAGTATTTTAAGTACTATAGCTAGTTTTTTAGGATTTGGAATTGGAACTTCACTTGGATTGCTGATAGG
ATACTTTATGTTTATATACTTTGAATCAATAGATGTCAAGGATCCTACATTTACTCCTTTGGTTGAGCAAG
AAGCTAAAACTGTGCAACAATTGCTTCCAGAAATACCCTTATGGATAAAAAATCCAGACTATGACCGTCTT
GATTGGCTTAATAAATTTGTTGAGTGTATGTGGCCTTATTTGAACAAGGCAATTTGCAAGACTACAAGAAC
TATAGCAAAGCCTATTATTGCGGAACAAATTCCAAAGTATAAAATTGATTCAGTTGAATTTGAAGAACTTA
ATTTGGGCTCTTTGCCACCAACTTTTCAAGGAATGAAAGTGTATAGCACTGATGAAAAGGAATTGATTATG
GAACTATCAATGAAATGGGCTGGGAATCCTAACATTATAGTTGCAGTTAAAGCATTTGGATTGCGAGCAAC
CGTTCAGGTTGTTGATTTGCAAGTGTTTGCTTCTCCGCGCATAATGTTGAAGCCTTTGGTTCCTAGTTTTC
CTTGTTTCGCCAATATTTACGTGTCTCTCATGGAAAAGCCACATGTTGATTTTGGATTAAAACTACTTGGA
GCTGATGCAATGTCTATTCCTGGTCTTTACAGGATTGTCCAGGAAATTATAAAAGATCAGGTTGCAAAAAT
GTATCTGTGGCCTAAGGCTTTACAGGTGCAAATAATGGATCCATCACAGGCCATGAAAAAACCAGTTGGAA
TTCTTCATGTGAAGATTCTGAAAGCAGTGAAGCTTAGAAAGAAAGATATAATGGGAGGAGCAGACCCTTAT
GTGAAACTTAAACTCAAAGATGATAAACTTGCTTCAAAGAAAACAACTGTGAAATACAAGAACTTGAATCC
AGAATGGAATGAAGAATTTAATGTGGTGATTAAAGATCCAGAATCTCAAGACTTAATGCTTAATGTTTATG
ATTGGGAACAGTTTGGAAAGGCTGAGAAGATGGGTATGAATGTAATTCCAGTGAAAGAGCTTACACCAAAT
GAACCAAAACTACTACTTCTTAAATTACTCAAGACACTTGTACCTAATGATCCTGAGAATGAGAAGTCGCG
TGGAGAGCTCATTGTGGAAGTTATGTACAAACCTTTTAAGGATGATGAAGTATCTAAAAATTCAGAAGACA
CAGAAAAGGCTCCGGAAGGAACACCTGCTAGTGGTGGTTTGCTTTTGATTAGCATCCATGAAGCTGAAGAT
GTTGAAGGAAAACACCACACAAATCCGTTTGCGCGGCTTATTTTTAAAGGCGAGGAAAGAAAAACCAAGCA
TGTAAGGAAAAATAGAGATCCAAGATGGGGTGAAACATTTCAATTTACACTAGAGGAACCCCCCATCAATG
AGAGGCTTTATGTTGAAGTGATAAGTGCATCCTCAAAGTTAGGCCTGCTGCATCCAAAGGAAACTCTTGGA
TATGTGGATATAAATTTATCTGATGTGGTTAGCAACAAAAGAATCAATGAGAAGTATCATCTTATTGACTC
CAAAAATGGAAAGATTCAAATTGAGCTTCAGTGGAGAACCCCATAA

Transcripts
dw017619
aw688250
ac149634
bm780043
aw695600
aj845838
dw015141
ca920827
aw698368

Medicago truncatula fragment ***NTMC2Type1***
aw685654
bq146433
ca989791
be319003

Medicago truncatula fragment ***NTMC2Type1***
bm779766
bg454004
bg646001

Medicago truncatula fragment ***NTMC2Type1***
ac147179.15 genomic exon coordinates (3731-3630)

Transcript

aw586970

Medicago truncatula fragment ***NTMC2Type1***
ac147179.15 genomic exon coordinates (3106-2954) (2854-2723) (2605-2489)

Medicago truncatula fragment ***NTMC2Type1***
ac144928.17 genomic exon coordinates (2699-2594) (2435-2338) (2245-2195) (2107-1984) (1321-1203) (1118-1017) (908-834)

Medicago truncatula fragment ***NTMC2Type1***
ac146557.32 genomic exon coordinates (108360-108267)

Medicago truncatula Full Length ***NTMC2Type2.1***

MGFVFGVVVGIIVGLAIIIAFVRSENSRSARRSQLATTIAAFARMTVEDSRKLLPSQFYP
SWVVFSNRQKLTWLNSHLTKIWPYVNE**^ 0**
AASELIKTSAEPILEEYRPMILSALKFSKFTLGTVAPQFT**^ +1**
GVSIIEDGGDGVTMELEVQWDGNPSIILDIKTLVGLALPVQ**^ 0**
VKNVGFTGVFRLIFKPLVNEFPGFGAVCYSLRQK**^ 0**
KKLDFTLKVIGGDISTIPGLYDAIE**^ 0**
GAIRDAVEDSITWPVRKIVPILPGDY**^ +2**
SDLELKPVGILEVKLVQAKELTNKDIIGKSDPYAVLYIRPLRNRTKKSKTI**^ 0**
NNDLNPIWNEHFEFIVEDASTQHLFVKVYDDEGLQSSELIGCTDIKLSELEPGKIKDVWL
KLVKDLEIQRDNKNRGQ**^ 0**
VHLELLYCPYGTENSFTNPFARNYSMTSLEKVLKGSSNGIDSNGNESEAAQRKKEVIIRG
VLSVTVISAEDLPAVDFMGKSDPFVVLTLKKAETKNKTR**^ 0**
VVNNSLNPVWNQTFDFVVEDGLHDMLLVEVYDHDTFGK**^ 0**
DYMGRVILTLTRAILEGEYKERFELDGAKSGFLNLHLKWMPQSIYRDS

<u>ATG</u>GGTTTCGTGTTTGGAGTGGTGGTCGGAATAATAGTCGGACTCGCCATCATTATCGCC
TTTGTCCGCTCCGAAAATTCCCGCTCCGCCCGTCGATCCCAGCTCGCTACCACCATCGCC
GCTTTTGCTCGAATGACCGTCGAAGATTCTAGAAAGCTTTTACCTTCTCAGTTCTACCCT
TCCTGGGTCGTCTTCTCCAATAGACAAAAATTAACTTGGCTTAACTCTCATCTTACCAAG
ATCTGGCCCTATGTTAATGAAG
phase 0
ac144657.6 (21868-21607)

CTGCTTCTGAGCTTATTAAGACATCGGCGGAACCGATCCTTGAAGAATACAGACCAATGA
TTTTATCTGCTCTCAAATTTTCCAAGTTTACTCTTGGTACTGTTGCACCACAATTTACAG
phase +1
ac144657.6 (21506-21387)

GGGTGTCTATAATTGAAGATGGAGGAGATGGTGTTACAATGGAGTTAGAAGTGCAGTGGG
ATGGTAACCCAAGTATTATACTTGATATTAAAACTCTGGTTGGTCTTGCACTGCCTGTGCAG
phase 0
ac144657.6 (20470-20349)

GTTAAAAATGTAGGATTTACGGGCGTCTTCAGGTTGATTTTTAAGCCACTAGTTAACGAA
TTTCCCGGCTTTGGAGCTGTTTGCTATTCACTAAGGCAGAAG
phase 0
ac144657.6 (19930-19829)

AAAAAGCTGGACTTTACACTAAAAGTCATTGGTGGTGACATATCAACAATCCCTGGATTA
TACGATGCAATTGAG
phase 0
ac144657.6 (19670-19596)

GGGGCAATCCGGGATGCTGTTGAAGATTCTATTACTTGGCCTGTGAGAAAAATTGTACCC
ATCTTGCCTGGAGATTACAG
phase +2
ac144657.6 (18738-18659)

TGATTTGGAGTTGAAGCCTGTGGGGATATTAGAAGTCAAGCTTGTGCAAGCAAAGGAACT
AACAAACAAGGATATCATTGGAAAATCAGATCCATATGCTGTACTGTACATACGACCTTT
ACGTAACAGAACGAAAAAAAGCAAGACAATT

phase 0
ac144657.6 (17962-17812)

AACAATGATTTGAATCCAATATGGAATGAACACTTCGAATTTATAGTTGAAGATGCGTCC
ACTCAGCACTTATTTGTTAAAGTGTATGATGATGAGGGGTTACAGTCATCGGAATTAATT
GGATGTACTGACATAAAGCTTAGTGAACTCGAACCTGGCAAAATAAAAGACGTGTGGTTG
AAACTGGTCAAGGATTTGGAGATCCAAAGAGATAACAAGAACAGGGGGCAG
phase 0
ac144657.6 (17700-17470)

GTACATTTAGAGCTTCTGTACTGTCCTTATGGCACAGAGAATAGCTTTACCAACCCGTTT
GCACGGAACTACTCAATGACATCCTTGGAAAAGGTTCTTAAAGGTTCATCAAATGGAATA
GATTCCAATGGAAATGAAAGTGAAGCTGCACAGAGGAAAAAGGAGGTTATTATTAGAGGA
GTCCTTTCTGTTACTGTGATATCAGCCGAAGACTTGCCTGCCGTGGATTTCATGGGGAAA
TCTGATCCTTTTGTTGTTCTTACATTGAAGAAAGCAGAAACAAAGAACAAAACCAGG
phase 0
ac144657.6 (16796-16500)

GTTGTGAACAACAGCTTGAATCCTGTTTGGAATCAAACATTTGACTTTGTTGTAGAGGAT
GGATTACATGATATGCTGCTTGTTGAAGTTTATGACCACGACACCTTTGGAAAG
phase 0
ac144657.6 (16292-16179)

GATTACATGGGCAGAGTGATATTGACCCTTACCAGGGCGATACTGGAAGGGGAATATAAA
GAACGTTTTGAGCTAGATGGTGCAAAATCTGGCTTTTTGAATTTGCATCTCAAGTGGATG
CCACAATCAATTTACCGTGATTCTTAA
ac144657.6 (15127-14981)

Transcripts
bi271450
al368643
bi265722
al389099

Medicago truncatula Full Length ***NTMC2Type2.2***

MGFISGMIIGIMIGMILVVAFARQESTRSKRRTDL**^ 0**
AKTIAKFARMTVEDSRKLLPPNFYPSWVVFTQRQK**^ 0** RNA editing?
LNWLNSHLEKIWPFVNE**^ 0**
AATELVKSNVEPILEQYRPVVLSSLTFSTFTLGNVAPQFT**^ +1**
GISIIEEDSGPNGATMEFDLQWDGNPDIVLAIKTKVGIVLPVQ**^ 0**
VKNIGFTGVFRLIFKPLVAEFPAFGAVCFSLRKK**^ 0**
KALDFTLKVVGGDISTLPGVSEAIE**^ 0**
ETIRDAIEDSITWPVRKVIPIIPGDY**^ +2**
SNLELKPVGTLDVKLVQAKNLSNKDIIGKSDPFAVVFVRPLRDKTKTSKII**^ 0**
NNQLNPIWNEHFEFIIEDESTQHLTIRIFDDEGIQAAELIGCAQVSLKELEPGKVKDVWL
KLVKDLEIHKDNKYRGE**^ 0**
VHLELLYCPYGVENTFKSPFVRDYSLTTFEKTLKNGASDGEEEDNSISSSSSSSRRKSNV
IVRGVLSVTVISAEDLPIVDFMGKADPFVVLALKKSEKKQKTR**^ 0**
VVNETLNPVWNQTFDFVVEDGLHDMLIVELWDHDTFGK**^ 0**
EKMGKVIMTLTKVILEGEYDETFILDDAKSGKINLHLRWTPQHKYREP

ATGGGATTCATCAGCGGCATGATCATTGGTATTATGATTGGTATGATATTGGTTGTCGCT
TTCGCTCGTCAAGAAAGTACTCGATCCAAGCGTCGCACAGATTTG
phase 0
ac148405.5 106701-106805

GCAAAGACAATAGCAAAGTTTGCGAGGATGACAGTGGAGGATTCGAGAAAACTTCTACCT
CCAAATTTTTATCCATCATGGGTGGTATTTACTCAAAGACAAAA
phase 0
ac148405.5 106963-107066

g inserted into transcipts between these exons?

TTAAATTGGCTTAATAGCCATTTAGAAAAGATTTGGCCATTTGTCAATGAG

phase 0
ac148405.5 107168-107218

GCAGCGACAGAATTGGTAAAAAGCAACGTCGAACCAATTCTTGAACAGTATAGGCCAGTG
GTTTTGTCTTCTCTCACATTTTCAACGTTTACACTCGGTAATGTAGCTCCACAATTTACAG
phase +1
ac148405.5 107335-107455

GAATTTCGATTATTGAAGAGGATAGTGGGCCCAATGGAGCAACAATGGAGTTTGACTTGC
AGTGGGATGGTAACCCGGATATTGTTCTTGCCATTAAAACCAAAGTTGGTATTGTATTAC
CTGTGCAG
phase 0
ac148405.5 108347-108474

GTAAAAAATATTGGATTCACTGGGGTTTTCAGGTTGATCTTCAAACCTCTTGTAGCTGAA
TTCCCTGCTTTTGGAGCCGTATGTTTTTCTCTAAGAAAAAAG
phase 0
ac148405.5 109102-109203

AAAGCTTTGGATTTTACTCTTAAGGTTGTTGGTGGTGATATTTCAACCTTACCAGGGGTA
TCTGAAGCTATTGAG
phase 0
ac148405.5 109513-109587

GAAACAATTCGAGATGCCATTGAAGACTCTATAACTTGGCCAGTACGTAAAGTCATACCA
ATTATACCAGGAGATTATAG
phase +2
ac148405.5 110494-110573

TAACCTAGAATTGAAACCCGTTGGAACACTAGATGTAAAACTAGTGCAGGCAAAGAACTT
ATCAAATAAAGATATAATTGGAAAATCTGATCCTTTTGCTGTGGTATTTGTACGCCCACT
CCGTGACAAAACCAAAACCAGTAAAATAATT
phase 0
ac148405.5 111129-111279

AACAACCAGTTGAACCCAATATGGAATGAGCACTTTGAATTCATTATTGAAGACGAATCA
ACACAACACTTGACCATAAGAATCTTTGATGATGAAGGCATTCAAGCAGCTGAGCTTATT
GGTTGTGCCCAAGTTTCCCTAAAGGAACTTGAGCCTGGTAAAGTTAAGGATGTATGGTTG
AAATTGGTTAAAGATTTAGAGATCCACAAAGATAACAAATATAGGGGTGAG
phase 0
ac148405.5 111361-111591

GTACACTTGGAGCTATTGTATTGCCCATATGGAGTTGAGAACACTTTTAAGAGCCCTTTT
GTCCGTGATTACTCACTAACAACGTTTGAGAAGACACTGAAGAATGGAGCTAGTGATGGA
GAGGAAGAAGATAACTCAATATCATCATCATCATCATCATCACGGAGGAAAAGCAACGTG
ATTGTAAGAGGAGTTCTATCGGTCACTGTAATTTCAGCTGAAGACTTACCAATAGTAGAT
TTTATGGGCAAGGCTGACCCATTTGTTGTCCTTGCATTGAAAAAATCAGAAAAGAAACAA
AAGACCCGA
phase 0
ac148405.5 111710-112018

GTTGTAAATGAAACTCTAAATCCAGTATGGAATCAAACATTTGACTTCGTTGTGGAGGAT
GGATTACACGACATGCTAATTGTTGAACTATGGGATCATGACACCTTTGGAAAG
phase 0
ac148405.5 112115-112228

GAAAAAATGGGAAAAGTGATCATGACACTAACCAAGGTCATATTGGAAGGGGAATACGAC
GAAACATTTATTTTGGATGATGCTAAATCCGGAAAGATTAATTTGCATCTAAGATGGACT
CCACAACACAAGTATCGGGAACCTTAA
ac148405.5 112550-112696

Medicago truncatula Full Length ***NTMC2Type3.1***

MSSKKGLMIPNNLEEASVKLLNQFVKVKENSRISYFLILVFIAWFIHKWIFSFSNCLPVI

LLLFASTQ**^ 0**
YGNYQRKILEEDLNKKWNRIIVNTS**^ 0**
PVTPLEQCEWLNLLLSQIWSNYFNPKLSTRLSAIVE**^ 0**
KRLKLRKPRFI**^ 0**
ERVEVQEFSLGSRPPSLGLQGIRWSTSGDQ**^ 0**
RLLKMGFDWDTSEMSILMVAKLSVGTARIVINSLHIKGD**^ 0**
LLVTPILDGKALLYSFVSTPEVRIGIAFGSGGSQSATELPGVSPWL**^ 0**
VKLFTDTLVKTMVEPRRRCFSLPAVDLRKYAVGGTIYVSVISANKLSRSCFKGRQQNGTS
DGCLEDNLSDKDLQTFIELEAEELTRRTGVRLGSTPRWDTTFNMVLHDNTGIVRFNLYQC
PSDSVKYDYLASCEIK**^ 0**
MRHVEDDSTIMWAVGTDSGVIAKHAKFCGEEVEMLVPFEGANSAE**^ 0**
LKVRIVVKEWQFSDGSHSLTNLHASPQKSLKGSSNLLSKTGRKLKITVVEAKDLDAKDRF
GKFDPYIKLQYGK**^ 0**
VVMKTKIAPPPATLTAVWNDTFEVDENSGDEYLIVKCFSEEIFGDENIGSAHVNLEGLVQ
GSIRDVWIPLEGVSSGELRLKIEAIWVENQEGSK**^ 0**
GPPSGVTNGWIELVLIEARDLIAADLRGTSDPFVRVNYGNLKKRTK**^ 0**
VVHKTINPRWDQTLEFLDDGSPLTLHVKDHNALLPTSSIGECVVEYQSLPPNQTSDKWIP
LQGVKSGEIHIQIARKVPEIQTRQSPDFEPSLTKLHQSPSQ**^ 0**
IKEMTKKVRYLIEDGNLEELSTTLSELETLEDTQEGYIAQLETEQMLLISKINELGQEII
NSSPSLNGSGN

ATGAGTAGTAAGAAAGGTTTGATGATTCCCAATAATTTGGAAGAAGCTTCAGTGAAGTTG
TTAAACCAATTTGTGAAAGTGAAGGAAAATTCACGGATTTCTTACTTTCTTATTCTCGTT
TTCATTGCTTGGTTTATTCACAAATGGATCTTCTCTTTCTCCAATTGCCTTCCAGTTATC
CTTCTTCTCTTTGCATCTACACAA
phase 0
ac152447.22 134506-134709

TATGGGAATTACCAGAGGAAAATACTTGAAGAGGACTTGAACAAGAAATGGAATCGTATC
ATAGTCAATACTTCG
phase 0
ac152447.22 134804-134878

CCTGTCACACCATTGGAACAATGTGAGTGGTTGAATCTATTGTTGTCACAAATCTGGTC
CAATTATTTTAATCCTAAGCTTTCAACAAGGTTATCAGCCATAGTAGAG
phase 0
ac152447.22 134968-135075

AAACGTTTAAAGCTGCGCAAACCAAGATTTATA
phase 0
ac152447.22 135174-135206

GAAAGGGTTGAAGTGCAGGAATTTTCACTTGGATCACGCCCTCCCAGTTTGGGCCTTCAA
GGGATTAGATGGTCAACGTCTGGTGATCAG
phase 0
ac152447.22 135321-135410

CGACTCCTGAAAATGGGTTTTGATTGGGACACGAGTGAGATGAGCATTTTGATGGTTGCT
AAGCTTTCCGTTGGTACTGCCAGAATTGTGATTAACAGTCTTCATATTAAGGGAGAT
phase 0
ac152447.22 135568-135684

CTTCTTGTGACACCAATACTAGATGGAAAAGCACTTTTGTATTCATTTGTATCTACACCT
GAGGTTAGAATAGGAATTGCCTTTGGAAGCGGAGGAAGTCAATCAGCCACTGAGTTGCCC
GGTGTTTCTCCTTGGCTG
phase 0
ac152447.22 135929-136066

GTTAAACTTTTTACTGACACTTTGGTAAAGACTATGGTGGAACCTCGGCGCCGGTGTTTC
AGTTTGCCTGCAGTTGATTTAAGGAAATATGCTGTTGGAGGCACCATATATGTTTCAGTG
ATTTCCGCAAACAAACTTTCTAGGAGTTGCTTCAAGGGGAGGCAACAAAATGGCACAAGT
GATGGCTGTTTAGAAGACAACTTGAGCGACAAGGACTTGCAGACATTTATAGAGCTAGAA
GCGGAGGAGCTGACAAGGAGAACGGGTGTGCGTCTTGGTTCAACTCCAAGATGGGATACA
ACATTTAATATGGTTTTACATGATAATACAGGAATAGTTCGCTTCAATCTTTATCAGTGC
CCCTCTGACAGTGTGAAGTACGACTACCTAGCATCTTGTGAAATTAAG
phase 0

ac152447.22 136315-136722

ATGAGGCATGTGGAGGATGATTCAACAATAATGTGGGCAGTAGGGACTGATTCTGGTGTA
ATAGCAAAACATGCAAAGTTTTGTGGAGAGGAAGTTGAAATGCTTGTCCCATTTGAGGGG
GCCAACTCAGCAGAG
phase 0
ac152447.22 137668-137802

TTAAAGGTGAGAATTGTAGTGAAAGAATGGCAATTCTCTGATGGTTCTCATAGCTTGACC
AACCTCCATGCCAGTCCTCAGAAATCACTTAAGGGATCATCAAATCTTCTTTCAAAAACT
GGAAGGAAACTTAAGATAACTGTTGTGGAAGCAAAGGATCTTGATGCAAAAGACAGATTT
GGAAAATTTGACCCTTACATTAAGTTGCAGTATGGAAAG
phase 0
ac152447.22 139242-139460

GTTGTCATGAAAACAAAGATTGCTCCTCCTCCTGCTACTTTAACTGCTGTCTGGAATGAT
ACTTTTGAGGTAGATGAGAATAGTGGTGATGAATACCTAATTGTGAAATGCTTTAGTGAA
GAAATTTTTGGAGATGAAAACATTGGTAGTGCACATGTAAATTTGGAAGGACTGGTACAA
GGGTCAATCAGGGATGTATGGATCCCACTTGAAGGAGTGAGTTCTGGTGAATTAAGACTT
AAAATAGAAGCAATTTGGGTGGAGAACCAAGAGGGATCAAAG
phase 0
ac152447.22 139625-139906

GGTCCACCATCGGGCGTGACGAATGGTTGGATTGAACTTGTTCTTATTGAAGCGAGGGAT
CTTATTGCTGCTGATCTTAGAGGGACAAGTGATCCTTTTGTGAGGGTAAACTATGGAAAC
TTAAAGAAAAGAACAAAG
phase 0
ac152447.22 140023-140160

GTTGTACACAAAACTATCAACCCTCGTTGGGACCAGACCTTAGAGTTCTTAGATGATGGA
AGTCCCCTGACATTGCATGTAAAGGACCATAATGCTTTACTACCCACATCAAGTATAGGT
GAATGTGTTGTAGAATATCAAAGCCTTCCTCCGAACCAGACGTCAGACAAGTGGATACCT
CTCCAAGGGGTGAAAAGTGGCGAGATCCACATCCAAATCGCAAGAAAAGTTCCAGAGATT
CAAACAAGACAATCTCCAGACTTTGAGCCTTCTTTGACTAAATTACACCAGAGTCCTAGC
CAG
phase 0
ac152447.22 140354-140656

ATAAAGGAGATGACAAAAAAGGTTCGGTATTTAATAGAGGATGGAAATCTGGAAGAACTG
TCTACAACTTTGAGTGAGTTAGAAACTCTAGAGGATACACAAGAAGGGTATATAGCCCAG
TTAGAGACTGAGCAAATGCTTCTTATCAGCAAAATAAATGAACTGGGCCAAGAGATCATA
AACTCTTCTCCATCTCTCAACGGAAGTGGAAATTGA
ac152447.22 141404-141619

Transcripts
aw696669
cx527463
bg449124
cx535005
bq147442
bq148691
cx540976
bq149339

Medicago truncatula Full Length ***NTMC2Type3.2***

MSRKKRVFSIDSIEEVAVDFFNYVLQEKPKIPFFIPVILIACAVEKWVFSFSTWVPLALA
VWATIQ**^ 0**
YGRYQRKLLVEDLDKKWKRIILNNS**^ 0**
PITPLEHCEWLNKLLTEIWPNYFNPKLSSRLSAIVE**^ 0**
ARLKLRKPRFL**^ 0**
ERVELQEFSLGSCPPSLALQGMRWSTIGDQ**^ 0**
RVMQLGFDWDTHEMSILLLAKLAKPLMGTARIVINSLHIKGD**^ 0**
LIFTPILDGKALLYSFVSAPEVRVGVAFGSGGSQSLPATEWPGVSSWL**^ 0**
EKLFTDTLVKTMVEPRRRCFTLPAVDLRKKAVGGIIYVRVISANKLSSSSFKASRRQQSG
STNGSSEDVSDDKDLHTFVEVEIEELTRRTDVRLGSTPRWDAPFNMVLHDNTGTLRFNLY

ECIPNNVKCDYLGSCEIK**^ 0**
LRHVEDDSTIMWAVGPDSGIIAKQAQFCGDEIEMVVPFEGTNSGE**^ 0**
LKVSIVVKEWQFSDGTHSLNNLRNNSQQSLNGSSNIQLRTGKKLKITVVEGKDLAAAKEK
TGKFDPYIKLQYGK**^ 0**
VMQKTKTSHTPNPVWNQTIEFDEVGGGEYLKLKVFTEELFGDENIGSAQVNLEGLVDGSV
RDVWIPLERVRSGEIRLKIEAIKVDDQEGST**^ 0**
GSGSGNGWIELVLIEGRDLVAADLRGTSDPYVRVHYGNFKKRTK**^ 0**
VIYKTLTPQWNQTLEFPDDGSPLMLYVKDHNALLPTSSIGECVVEYQRLPPNQMADKWIP
LQGVKRGEIHIQITRKVPEMQKRQSMDSEPSLSKLHQIPTQ**^ 0**
IKQMMIKFRSQIEDGNLEGLSTTLSELETLEDTQEGYVAQLETEQMLLLSKIKELGQEII
NSSPSPSLSRRISESVN

ATGAGTAGAAAGAAAAGGGTATTTTCAATTGATAGTATTGAAGAGGTTGCTGTGGATTTC
TTCAACTATGTTTTGCAGGAGAAGCCAAAGATTCCATTTTTCATTCCAGTTATTTTGATT
GCTTGTGCTGTTGAAAAATGGGTCTTTTCTTTTTCTACTTGGGTTCCTCTTGCACTTGCT
GTTTGGGCTACTATACAG
phase 0
ac146563.19 85667-85864

TATGGAAGATACCAACGCAAACTACTTGTAGAGGACTTGGATAAGAAATGGAAGCGAATC
ATATTGAATAACTCG
phase 0
ac146563.19 86901-86975

CCCATCACACCATTGGAGCACTGTGAATGGCTGAATAAATTGTTGACAGAAATCTGGCC
CAACTATTTTAATCCCAAGCTTTCTTCAAGGTTATCAGCTATAGTTGAG
phase 0
ac146563.19 87054-87161

GCACGTTTGAAGCTCCGGAAACCAAGATTTCTT
phase 0
ac146563.19 87458-87490

GAACGAGTTGAGCTACAAGAGTTTTCACTAGGATCATGCCCTCCTAGCTTGGCCTTACAA
GGGATGCGATGGTCAACTATCGGCGATCAG
phase 0
ac146563.19 87613-87702

CGAGTTATGCAACTTGGATTTGATTGGGACACCCATGAAATGAGCATTTTGCTCCTTGCT
AAGCTTGCGAAACCATTGATGGGAACTGCACGAATCGTTATTAACAGTCTTCATATCAAG
GGAGAT
phase 0
ac146563.19 87881-88006

CTTATCTTTACGCCAATTCTAGATGGAAAAGCACTTTTATATTCGTTTGTATCAGCTCCT
GAGGTGAGAGTTGGAGTTGCCTTTGGAAGCGGTGGGAGTCAGTCACTTCCAGCTACTGAG
TGGCCTGGTGTTTCTTCTTGGCTG
phase 0
ac146563.19 88128-88271

GAAAAACTTTTTACTGACACTCTGGTTAAAACCATGGTGGAACCTCGGCGACGATGTTTC
ACTTTGCCTGCTGTTGATTTAAGAAAAAAGGCGGTTGGAGGGATCATATATGTTAGAGTG
ATTTCAGCCAATAAACTTTCTAGTAGTTCCTTCAAGGCATCTAGGAGGCAACAAAGTGGA
TCAACCAATGGTTCTTCGGAAGACGTTTCTGACGACAAGGACCTGCATACATTTGTAGAG
GTAGAAATTGAGGAATTGACGAGGAGAACAGATGTCAGATTAGGTTCAACTCCTAGATGG
GATGCTCCATTTAATATGGTTTTGCATGACAATACAGGAACACTTCGTTTCAATCTTTAC
GAGTGTATTCCCAACAATGTGAAGTGTGACTATCTAGGAAGTTGTGAAATCAAG
phase 0
ac146563.19 88531-88944

CTGAGGCATGTTGAAGATGACTCAACAATAATGTGGGCAGTAGGACCAGATTCCGGGATT
ATAGCAAAGCAAGCACAATTTTGTGGAGATGAAATTGAAATGGTTGTCCCATTTGAGGGT
ACTAACTCTGGAGAG
phase 0
ac146563.19 89325-89459

TTGAAGGTGAGCATTGTAGTGAAAGAGTGGCAATTTTCTGATGGAACTCATAGCTTGAAC
AATCTCCGGAACAACTCTCAGCAGTCACTTAACGGATCATCAAATATTCAGTTAAGAACG
GGAAAGAAACTTAAAATAACTGTTGTAGAAGGAAAGGATCTTGCTGCTGCAAAAGAAAAA
ACTGGAAAATTTGATCCATACATTAAATTGCAATATGGAAAG
phase 0
ac146563.19 89971-90192

GTTATGCAGAAAACAAAGACTTCTCATACTCCAAATCCTGTTTGGAATCAAACAATTGAA
TTTGATGAGGTTGGTGGCGGCGAATACTTAAAACTAAAAGTATTTACTGAGGAACTTTTC
GGAGATGAAAACATTGGTAGTGCACAAGTAAATTTGGAAGGACTGGTTGATGGATCGGTC
AGGGATGTATGGATTCCTCTTGAAAGAGTGCGTTCTGGAGAAATCAGACTTAAAATAGAA
GCCATAAAAGTGGATGACCAAGAAGGATCAACG
phase 0
ac146563.19 90273-90545

GGTTCAGGCTCAGGAAATGGTTGGATAGAACTTGTTCTGATTGAAGGGAGGGATCTTGTT
GCCGCAGATCTCAGAGGCACAAGTGATCCATATGTTAGGGTACACTATGGAAACTTCAAG
AAAAGGACAAAG
phase 0
ac146563.19 90967-91098

GTTATATACAAAACTCTAACCCCTCAATGGAACCAGACCCTAGAGTTCCCTGATGATGGA
AGTCCCTTGATGCTTTATGTGAAGGACCATAATGCGTTACTACCTACATCAAGTATAGGT
GAATGTGTTGTAGAATATCAAAGGCTGCCCCCGAACCAGATGGCTGATAAGTGGATACCA
CTTCAAGGTGTGAAAAGGGGTGAAATTCATATTCAAATTACTAGAAAAGTTCCAGAAATG
CAGAAGAGGCAAAGTATGGACTCTGAACCTTCTTTGAGTAAATTGCACCAAATTCCTACG
CAG
phase 0
ac146563.19 91266-91568

ATAAAACAGATGATGATCAAGTTTAGGTCTCAAATCGAAGATGGAAATCTTGAAGGACTC
TCTACAACATTGAGTGAGCTAGAAACTTTAGAGGATACACAAGAAGGATATGTAGCTCAG
CTAGAGACGGAACAAATGCTTCTTCTCAGCAAGATAAAGGAACTTGGCCAAGAGATCATT
AATTCTTCTCCTTCTCCTTCCTTAAGCAGAAGAATTTCTGAAAGTGTCAACTAA
ac146563.19 92143-92376

Transcripts
bf643626
cx549634
aw695395
aw774947
aw736011
cx534948
cx524499
cx539263
bf634689
ai974598
bq164898
ca921888
ca921550
ca921887
bq164899

Medicago truncatula Full Length ***NTMC2Type4.1***

MGLISGIFMGMLFGIALMAGWARMMRYRSAKRIAK**^ 0**
AVDIKILGSLNREDLKKICGENLPEWISFPVYEQ**^ 0**
VKWLNKLLSKLWPFVAE**^ 0**
AATMVIKESVEPLLEEYRPPGITSLKFSKLSLGNVAPKIE**^ +1**
GIRVQSLTKGQIIMDVDLRWGGDPSIILAVEAALVASIPIQ**^ 0**
LKDLKVFTIARVIFQLAEEIPCISAVVVALLAE**^ 0**
PKPRIDYTLKAVGGSLTALPGISDMID**^ 0**
DTVNTIVTDMLQWPHRIVVPLGGIPVDI**^ +2**
SDLELKPHGSLKVTIVKATDLKNMEMIGKSDPYVVLYIRPLFKVKTKVINNNLNPVWDQT
FELIAEDKETQSLILE**^ 0**
VFDEDIGQDKRLGIVKLPLIELEVQTEKELELRLLSSLDTLKVKDKKDRGTLTVK**^ 0**

VLYYQFNKEEQLAALEAEKAILEERKKLKAAGVIGSTMDAVGSGVGLVGSGIGLVGTGIG
AGAGLVGSGIGAGAGLVGSGFGAFGSGLSKAGKFMGRTITGHSGSRRSGSSTPVHNPQEN
GSPKKTQ

ATGGGTTTGATTTCTGGGATTTTTATGGGGATGCTATTTGGCATAGCATTGATGGCAGGA
TGGGCTCGTATGATGAGGTATAGAAGTGCCAAGAGAATTGCAAAG
phase 0
ac174347.10 (54400-54296)

GCTGTTGACATCAAAATCCTTGGATCCCTCAACAGAGAAGATTTAAAGAAAATTTGTGGG
GAGAATCTTCCTGAATGGATATCTTTTCCTGTATATGAGCAG
phase 0
ac174347.10 (51114-51013)

GTGAAATGGCTAAACAAGCTCCTGAGCAAGCTTTGGCCATTTGTAGCAGAA
phase 0
ac174347.10 (50900-50850)

GCCGCAACAATGGTGATCAAGGAATCTGTTGAACCTCTACTGGAAGAGTACAGACCTCCT
GGAATTACTTCATTGAAGTTCAGCAAGCTGTCACTTGGAAACGTAGCTCCAAAAATTGAAG
phase +1
ac174347.10 (50133-50013)

GTATTCGTGTGCAGAGTCTTACCAAAGGTCAAATCATAATGGACGTAGATCTCCGCTGGG
GTGGTGATCCCAGTATTATTTTGGCTGTCGAAGCTGCCCTTGTTGCATCAATCCCTATTCAG
phase 0
ac174347.10 (48713-48592)

TTGAAGGATCTAAAAGTTTTCACCATTGCCCGTGTTATATTCCAACTTGCTGAAGAGATC
CCTTGCATTTCTGCTGTTGTTGTTGCTCTTCTTGCTGAG
phase 0
ac174347.10 (48494-48396)

CCAAAGCCGAGAATTGATTATACTCTGAAAGCTGTTGGTGGAAGTTTAACTGCTCTTCCT
GGAATTTCAGATATGATTGAT
phase 0
ac174347.10 (47478-47398)

GATACTGTGAACACAATTGTTACTGATATGCTCCAATGGCCACATAGGATTGTTGTTCCA
CTTGGTGGTATCCCTGTTGATATTAG
phase +2
ac174347.10 (47270-47185)

TGACTTGGAGCTTAAACCTCATGGAAGCCTTAAAGTGACTATTGTGAAAGCGACCGATTT
AAAAAATATGGAAATGATTGGAAAATCTGATCCTTATGTTGTTCTGTACATTCGACCTTT
ATTCAAGGTTAAAACCAAGGTTATTAACAACAACTTGAATCCTGTTTGGGATCAAACATT
TGAGTTGATTGCAGAAGACAAGGAGACCCAGTCACTCATTCTTGAG
phase 0
ac174347.10 (47068-46843)

GTTTTTGATGAAGACATTGGGCAAGATAAGCGACTGGGAATCGTAAAATTACCCCTTATT
GAATTGGAAGTACAAACTGAAAAAGAGCTTGAATTGAGGCTGCTGTCATCGCTTGATACA
CTCAAGGTGAAAGATAAGAAGGATCGAGGAACCTTGACCGTAAAG
phase 0
ac174347.10 (45514-45350)

GTCCTGTATTACCAATTTAACAAAGAGGAACAATTGGCTGCACTAGAAGCAGAAAAAGCT
ATATTAGAAGAAAGGAAGAAACTGAAAGCTGCCGGCGTCATAGGTAGCACAATGGATGCA
GTAGGGTCAGGTGTTGGACTAGTCGGAAGTGGCATTGGACTAGTCGGAACTGGCATTGGC
GCCGGAGCTGGACTTGTCGGAAGCGGCATTGGTGCTGGAGCTGGACTTGTTGGCAGTGGT
TTTGGAGCCTTTGGTAGCGGACTTAGCAAGGCAGGAAAGTTTATGGGAAGGACCATAACA
GGTCATAGTGGTTCTAGAAGGAGTGGTTCGTCAACTCCAGTTCATAATCCACAGGAAAAT
GGTAGTCCAAAAAAGACTCAATAA
ac174347.10 (44517-44134)

Transcripts
dw018208
bf645735
bf648797
bf642084
bg455434
bi266815
aw256436
dw016269
cx538754
cx525169
cb894985
al385426
al372268
bi264114
al375526
al381486
ca921243
aj621847
al381487
al385427

Medicago truncatula Full Length ***NTMC2Type4.2***

MGLFFGIFLGVLFGVALMAGWERMMTYRSRKRIAK**^ 0**
AVDIKLLGSLNRDDLKKICGENLPEWISFPVYEQ**^ 0**
VKWLNKQLSKLWPFVAD**^ 0**
AATMVIRESVEPLLEEYRPPGISSLKFSKLSLGTVAPKIE**^ +1**
GIRVQSLKKGQIIMDIDFRWGGDPNIVLGVEALVASIPIQ**^ 0**
LKDLQVFTIIRVIFQLAEEIPCISAVVVALLAE**^ 0**
PKPRIDYTLKAVGGSLTALPGLSDMID**^ 0**
DTVNSIVTDMLQWPHRIVVPLGGTPVDT**^ +2**
SDLELKPQGLLKVTVMKANDLKNMEMIGKSDPYVVVHIRPLFKVKTKVIDNNLNPIWNEE
FDLIAEDKETQSLTLE**^ 0**
VFDKDIGQDKRLGVAKLPLINLEAETEKEIELRLLSSLDTLKVKDKKDRGTLRIK**^ 0**
YFYHEFNKEEQMAALEAEKMTLEQRKKLKEEGVIGSTMDALDGAASVVGSGAGLVGSGIG
AGDGMVGHGFGAGAGIVGSGLGAVGSGLSRAGKFMGRTITGQSASRRSASGSSTPVFNVE
ESGGGAKPR

<u>ATG</u>GGTTTGTTTTTTGGGATTTTTCTGGGAGTATTATTCGGTGTAGCATTGATGGCTGGA
TGGGAACGAATGATGACATACAGAAGCCGTAAGAGAATTGCAAAG
phase 0
ac147014.25 61576-61680

GCAGTTGACATTAAACTCCTTGGATCCCTTAATAGAGACGACTTAAAGAAAATTTGTGGT
GAAAATTTACCTGAATGGATATCTTTCCCTGTATATGAGCAG
phase 0
ac147014.25 62879-62980

GTGAAATGGCTAAACAAGCAGCTCTCCAAATTGTGGCCATTTGTGGCAGATG
phase 0
ac147014.25 63129-63180

CAGCAACTATGGTGATAAGAGAATCTGTTGAACCGTTATTAGAAGAGTATAGACCTCCTG
GAATTTCTTCGTTGAAGTTCAGTAAGCTGTCCCTTGGAACTGTCGCTCCAAAAATTGAAG
phase +1
ac147014.25 64659-64778

GCATTCGTGTTCAGAGTCTTAAAAAAGGTCAAATCATAATGGACATTGATTTCCGTTGGG
GTGGTGATCCTAATATTGTTTTGGGCGTTGAAGCACTTGTTGCGTCAATTCCTATTCAG
phase 0
ac147014.25 65805-65923

TTGAAGGATCTCCAGGTTTTCACCATTATCCGTGTCATATTCCAACTCGCAGAAGAGATC
CCCTGCATTTCTGCTGTTGTTGTTGCCCTACTTGCTGAG
phase 0
ac147014.25 66008-66106

CCAAAGCCTAGAATTGATTACACTCTGAAGGCAGTTGGTGGAAGTTTGACGGCACTTCCT
GGACTTTCAGATATGATTGAT
phase 0
ac147014.25 66648-66728

GATACTGTGAACTCAATTGTTACCGATATGCTCCAATGGCCTCATCGGATTGTTGTTCCA
CTTGGTGGTACTCCTGTGGATACTAG
phase +2
ac147014.25 66822-66907

TGATCTGGAGCTTAAACCCCAGGGACTGCTTAAAGTGACTGTAATGAAAGCAAATGATCT
AAAGAACATGGAAATGATTGGGAAGTCTGATCCTTACGTAGTTGTGCATATTCGACCACT
GTTTAAAGTCAAAACAAAGGTTATTGATAACAACTTGAATCCAATTTGGAATGAGGAATT
TGACTTGATTGCAGAAGACAAGGAGACCCAGTCACTCACTCTTGAG
phase 0
ac147014.25 67114-67339

GTTTTTGATAAAGACATTGGGCAAGATAAACGATTGGGAGTAGCAAAATTGCCACTTATT
AACCTGGAAGCAGAAACTGAAAAGGAAATTGAACTGAGGCTTCTGTCATCGCTTGATACA
TTGAAAGTAAAAGATAAGAAGGATCGAGGAACCTTGAGAATAAAG
phase 0
ac147014.25 67879-68043

TATTTCTATCATGAATTTAACAAGGAAGAACAGATGGCTGCACTAGAAGCAGAGAAAATG
ACACTAGAACAAAGGAAGAAACTGAAAGAGGAAGGAGTTATTGGAAGTACAATGGACGCA
CTAGACGGAGCAGCATCAGTAGTTGGTTCTGGTGCTGGACTTGTAGGAAGTGGGATTGGT
GCTGGAGATGGAATGGTGGGACACGGATTTGGTGCTGGAGCTGGAATTGTTGGCAGTGGG
CTTGGTGCCGTAGGCAGTGGACTGAGCAGGGCAGGAAAGTTCATGGGCAGGACAATCACA
GGGCAATCTGCATCAAGAAGGAGTGCCAGCGGTTCATCTACTCCTGTCTTTAATGTGGAG
GAAAGTGGGGGTGGCGCAAAGCCACGGTAA
ac147014.25 68308-68697

Medicago truncatula fragment ***NTMC2Type5***
cx526554
bg453678
aw560028
aw560027

Medicago truncatula fragment ***NTMC2Type5***
ac171394.2 genomic exon coordinates 39305-39522, 40127-40240, 40324-40422, 41769-42222, 42340-42428

Transcripts
aw698606
aw698597
bq146895
aw698724

Medicago truncatula fragment ***NTMC2Type5***
aw698606
bq146895

Medicago truncatula fragment ***NTMC2Type6***
be325231
bi270281
be942539
be942538
be239975

Medicago truncatula fragment ***NTMC2Type6***
bf642174
ai974733
bf634587
al368519

Phaseolus vulgaris fragment ***NTMC2Type1***
ca908778 (Phaseolus coccineus)
cb540202
cv540810
cv540827
cv541751
cv542177

Phaseolus vulgaris fragment ***NTMC2Type1***
cv534327

Phaseolus coccineus fragment ***NTMC2Type2***
ca908766

Phaseolus coccineus fragment ***NTMC2Type3***
ca913979

Phaseolus vulgaris fragment ***NTMC2Type4***
cv537672
ca908777 (Phaseolus coccineus)
cv530918

Phaseolus coccineus fragment ***NTMC2Type4***
ca908776

Phaseolus vulgaris fragment ***NTMC2Type5***
cv529853

Phaseolus vulgaris fragment ***NTMC2Type6***
cv538119
cv538129

Pisum sativum fragment ***NTMC2Type1***
am161888

Poncirus trifoliata fragment ***NTMC2Type1***
cd576310

Poncirus trifoliata fragment ***NTMC2Type1***
cd575421

Poncirus trifoliata fragment ***NTMC2Type2***
cf421158
cf418269
cf419047

Poncirus trifoliata fragment ***NTMC2Type2***
cx642094
cx642093

Poncirus trifoliata fragment ***NTMC2Type4***
cv708201
cv708033
cd573831
cv705747
cv711786

Poncirus trifoliata fragment ***NTMC2Type4***
cd573832
cv705718
cv705746
cv711547
cv711787

Poncirus trifoliata fragment ***NTMC2Type4***
cv711548
cv705719

Poncirus trifoliata fragment ***NTMC2Type5***
cx641661

Populus tremula Full Length ***NTMC2Type1***

MGFFSTILGFCGFGVGISTGLTIGYYLFIYFQPSDVKDPEVRPLVEHDSETLQRMLPEIP
LWVKNPDHDRIDWLNRFIQLMWPYLDKAICKTAENIAKPIIAEQIPKYKIDAVEFETLTL
GTLPPTFHGMKVYVTDEKELIMEPCIKWAGNPNVTIAVKAFGLKATAQVVDLQVFASPRI
TWKPLVTNFPCIANIYVSLMEKPHVDFGLKLLGADLMSIPGLYRVVQEIIKDQVANMYLW
PKTLEVPILDPANAMKRPVGILKVKVLRAMKLKKKDLMGASDPYVKIKLTEDKLPAKKTT
VKHKNLNPEWNEEFNVVVKDPESQALELRVYDWEQVGKHDKMGMNVVPLKELTPEEPKIM
TLELLKNMDLNDPQNEKSRGQLMVELTYKPFKEDDVNLSFKEQAEQKAPEGTPAGGGLLV
VIVHEAQDVEGKHHTNPYVRLLFRGEEKRTKHVKKNRDPRWEDEFQYTLDKPPVNEKLHV
EVISTSSRIGLLHPKESLGYVDINLTDVVNNRRTNGKYHLIDSKNGQIQIELQWRPAS

ATGGGGTTTTTTAGTACAATACTAGGTTTCTGTGGATTCGGTGTTGGAATTTCCACGGGT
CTTACCATCGGGTACTACCTCTTCATCTACTTCCAACCCTCCGATGTTAAGGATCCTGAG
GTACGGCCATTAGTAGAGCATGACTCGGAAACTTTGCAACGAATGCTTCCAGAAATACCG
CTATGGGTGAAAAATCCGGACCATGATCGCATTGATTGGCTAAACAGGTTTATTCAATTA
ATGTGGCCTTACCTTGATAAGGCCATTTGTAAGACCGCGGAGAATATAGCAAAACCAATA
ATTGCCGAGCAGATCCCAAAATACAAAATTGATGCGGTTGAGTTTGAAACTCTTACACTA
GGCACCCTACCGCCAACCTTTCACGGAATGAAAGTTTATGTCACTGATGAGAAGGAGTTG
ATTATGGAACCATGCATAAAATGGGCTGGAAATCCTAATGTCACTATTGCTGTTAAAGCA
TTTGGGTTGAAAGCAACTGCTCAGGTGGTTGATTTGCAAGTGTTTGCTTCACCACGCATA
ACTTGGAAGCCTTTGGTGACTAACTTTCCTTGTATTGCCAACATCTATGTGTCTCTCATG
GAGAAGCCACATGTAGACTTTGGGCTCAAGCTCTTGGGGGCTGATCTAATGTCAATACCT
GGCCTTTACAGGGTCGTCCAGGAGATTATTAAAGATCAGGTTGCAAACATGTATCTATGG
CCTAAAACCCTGGAAGTACCCATCTTGGATCCTGCAAATGCCATGAAGAGGCCTGTGGGA
ATTCTCAAAGTGAAGGTTCTGAGGGCAATGAAGCTGAAGAAGAAAGATCTGATGGGAGCA
TCAGACCCCTATGTGAAAATCAAGCTGACCGAAGATAAGCTTCCAGCCAAAAAGACCACT
GTGAAGCACAAGAACTTGAACCCTGAATGGAATGAGGAATTCAATGTAGTTGTTAAAGAT
CCAGAATCTCAGGCTCTAGAATTACGTGTTTATGATTGGGAGCAGGTTGGCAAACATGAC
AAGATGGGTATGAATGTAGTGCCGCTGAAAGAACTTACCCCAGAGGAACCAAAAATTATG
ACTCTTGAGCTCCTGAAAAACATGGACTTGAACGATCCTCAAAATGAAAAGTCACGTGGG
CAACTTATGGTTGAATTGACATATAAACCATTCAAAGAGGACGATGTAAACCTAAGTTTT
AAAGAGCAGGCGGAACAGAAGGCTCCTGAAGGAACCCCTGCTGGTGGAGGTCTGCTTGTT
GTTATAGTTCACGAAGCTCAGGATGTTGAAGGAAAGCACCACACTAATCCATATGTGCGA
CTTCTTTTTAGAGGGGAGGAGAAAAGGACTAAGCACGTGAAGAAGAATAGAGATCCAAGA
TGGGAGGATGAATTCCAATATACGCTGGACAAGCCTCCTGTTAATGAGAAGCTTCACGTG
GAAGTCATCAGCACCTCGTCTAGGATTGGCCTGCTGCATCCAAAGGAATCACTTGGTTAC
GTGGATATCAATCTCACAGATGTTGTTAATAACAGAAGAACCAACGGAAAGTATCACCTT
ATAGACTCGAAGAATGGACAGATTCAAATAGAGTTGCAGTGGAGACCAGCTTCATAA

Transcripts
cx173098 (Populus deltoides)
cx170494 (Populus deltoides)
cf232073 (Populus alba x tremula)
ck106084
bu820322
bu820273
cx176671 (Populus deltoides)
bu820990
cx172450 (Populus deltoides)
cf236165 (Populus alba x tremula)
bu895005 (Populus tremula x tremuloides)
ca930321 (Populus tremuloides)
dt489168 (Populus trichocarpa)
ck096524
cx282505 (Populus alba x tremula)
ck096248
bu870546 (Populus balsamifera)
aj776276 (Populus euphratica)

cv228869 (Populus balsamifera)
bu819381
ck096054
cv240857 (Populus balsamifera)
aj779341 (Populus euphratica)
aj779280 (Populus euphratica)
aj779918 (Populus euphratica)
aj780072 (Populus euphratica)
ca825593 (Populus balsamifera)
cf232605 (Populus alba x tremula)

Populus balsamifera fragment ***NTMC2Type1***
dt486594 (Populus trichocarpa)
cv227596
bu811966 (Populus tremula x tremuloides)
cv2458419
bu813879 (Populus tremula x tremuloides)
cv240713
cx178264 (Populus euramericana)
bi120097
ck100922
bu869942

Populus tremula x tremuloides fragment ***NTMC2Type1***
cx171401 (Populus deltoides)
bu885696
bu887010
cx186074 (Populus euramericana)
bu887818

Populus tremula x tremuloides fragment ***NTMC2Type1***
dn484741
dn494215

Populus balsamifera x deltoides fragment ***NTMC2Type1***
cn519260

Populus tremula x tremuloides fragment ***NTMC2Type2***
bu831127
aj646515
bi131701
ck103619
ck093567
cv259304 (Populus balsamifera x nigra)
bu818983 (Populus tremula)
cv250648 (Populus balsamifera)
bu836991
bu810043
cf228109 (Populus alba x tremula
cv233242 (Populus balsamifera)
cv272954 (Populus balsamifera)
cv244926 (Populus balsamifera)

Populus deltoides fragment ***NTMC2Type2***
cx174391
dt470519 (Populus trichocarpa)
cx169633
dt498072 (Populus trichocarpa)
bu809521 (Populus tremula x tremuloides)

Populus tremula x tremuloides fragment ***NTMC2Type2***
dn500504

Populus deltoides fragment ***NTMC2Type2***
cx169539

Populus tremula fragment ***NTMC2Type3***
dt469579 (Populus trichocarpa)
cx656768 (Populus alba x tremula)
bu817558

aj778620 (Populus euphratica)
ck105519
bu873893 (Populus balsamifera)
ck095646
bu871917 (Populus balsamifera)

Populus balsamifera x deltoides fragment ***NTMC2Type3***

cn521531
bi071710 (Populus tremula x tremuloides)

Populus balsamifera fragment ***NTMC2Type3***

cv242917
dt487704 (Populus trichocarpa)

Populus trichocarpa Full Length ***NTMC2Type4***

MGLISGLFLGIVFGIGLMAGWKHMMQYRSTKRVAKAVDIKLLGSLNRDDLKKICGDNFPD
WISFPAFEQVKWLNKQLGKLWPFVAEAATAVVKESVEPLLEDYRPPGITSLKFNKFSLGT
VPPKIEGIRVQSLKQGQVTMDIDLRWCGDPSIILGVEAALVASIPIQLKDLEVYTVIRVI
FQLAEEIPCISAVVIALLSEPKPKIEYILKAVGGSLTALPGVSDMIDDTVNSIVTDMLQW
PHRIVVPIGGIPVDISELELRPQGKLTVTVVKANDLKNMEMIGKSDPYAVVYVRPMFKVK
TQVIDNNLNPVWNQTFDLIAEDKETQSLILEVFDKDIGQDKRLGRAKLALNELEAETWKE
LEFGLLSSFDTLKVKDKKDRGTITIKVFYHEFNKEESLAALEEEKQIIEQRKKLKEAGVI
GSTMDALDGAASLVGSGVGLVGGGVGAGVGFVGSGLGAVGSGLSKAGRFMGRTITGQSSK
RTGNTTPVNSVQENGGAKPL

ATGGGGCTGATTTCAGGGTTATTTTTGGGGATCGTCTTCGGGATCGGGTTGATGGCTGGA
TGGAAACACATGATGCAGTACCGAAGCACCAAACGAGTTGCTAAGGCAGTGGATATAAAA
CTCCTTGGGTCACTCAATAGAGATGATTTGAAGAAAATATGTGGTGATAATTTTCCTGAT
TGGATTTCTTTTCCTGCTTTTGAACAGGTGAAATGGCTAAATAAGCAATTGGGCAAATTA
TGGCCTTTTGTTGCAGAAGCAGCTACAGCGGTGGTTAAAGAATCTGTTGAACCACTATTG
GAAGACTACCGACCCCCAGGGATTACTTCTCTAAAGTTTAACAAATTTTCTCTCGGTACT
GTGCCGCCTAAGATTGAAGGAATTCGTGTCCAGAGTCTCAAGCAAGGTCAAGTTACCATG
GATATTGATTTGCGGTGGTGTGGTGATCCAAGCATCATTCTAGGCGTTGAAGCTGCACTT
GTTGCTTCCATACCCATTCAATTGAAAGATCTTGAAGTTTACACTGTTATCCGTGTTATC
TTTCAACTTGCTGAAGAGATTCCATGTATTTCTGCTGTTGTGATTGCTCTACTTTCTGAG
CCAAAGCCAAAGATTGAATACATTCTCAAGGCTGTTGGTGGAAGCTTAACAGCACTTCCT
GGAGTCTCAGATATGATTGATGATACTGTGAATTCGATTGTTACGGATATGCTTCAGTGG
CCCCACAGGATTGTTGTTCCCATTGGTGGTATCCCTGTTGACATAAGTGAATTAGAGCTT
CGACCTCAAGGAAAGCTTACAGTGACCGTAGTGAAGGCAAATGACTTGAAGAACATGGAA
ATGATTGGAAAATCTGATCCTTATGCAGTTGTATATGTTCGGCCAATGTTCAAGGTTAAA
ACACAAGTCATTGATAATAACCTGAATCCTGTTTGGAATCAAACATTTGATTTGATTGCT
GAAGACAAGGAGACGCAATCACTTATTCTTGAGGTCTTTGATAAGGACATTGGGCAAGAC
AAGAGATTAGGAAGAGCAAAATTAGCTTTAAATGAGCTGGAAGCCGAAACTTGGAAGGAG
CTGGAGTTTGGACTGTTGTCATCGTTTGATACATTGAAGGTTAAGGATAAGAAGGATAGA
GGAACTATTACAATTAAGGTTTTTTACCATGAATTTAACAAGGAAGAGTCGTTGGCTGCT
CTAGAAGAAGAGAAGCAGATCATAGAGCAACGAAAGAAACTAAAAGAAGCTGGAGTTATT
GGGAGCACAATGGATGCGCTTGATGGGGCAGCATCACTGGTGGGATCAGGAGTTGGGCTT
GTGGGAGGTGGTGTTGGTGCTGGAGTTGGGTTTGTGGGAAGTGGCTTAGGAGCTGTTGGT
AGTGGCCTGAGCAAAGCAGGAAGGTTCATGGGCAGGACAATCACCGGACAATCCAGCAAG
AGAACTGGCAACACAACTCCAGTCAATAGTGTGCAAGAAAACGGTGGAGCAAAGCCCCTGTAG

Transcripts

dt502455 (Populus trichocarpa x deltoides)
dv466556 (Populus fremontii x angustifolia)
bp922332 (Populus nigra)
bp928463 (Populus nigra)
dt477290
dv465092 (Populus fremontii x angustifolia)
dv462924 (Populus fremontii x angustifolia)
aj777134 (Populus euphratica)
bu897124 (Populus tremula x tremuloides)
dt482329
bu812504 (Populus tremula x tremuloides)
dt485816
dt480555
cv262114 (Populus trichocarpa x nigra)

dt519120 (Populus trichocarpa x deltoides)
bp935958 (Populus nigra)

Populus alba x tremula fragment ***NTMC2Type4***
cf230890

Populus tremula x tremuloides fragment ***NTMC2Type5***
dn492983
dn502997
bu897051
ck094314 (Populus tremula)
ck104277 (Populus tremula)
bi123448
ck094307 (Populus tremula)

Populus tremula fragment ***NTMC2Type5***
bu891513
dn496261
cv271237 (Populus trichocarpa x nigra)

Populus euramericana x canadensis fragment ***NTMC2Type5***
cx185603
cx184890

Populus euphratica fragment ***NTMC2Type5***
aj775999

Populus deltoides fragment ***NTMC2Type5***
cx170093

Populus tremula x tremuloides fragment ***NTMC2Type5***
bi124988

Populus tremula x tremuloides fragment ***NTMC2Type6***
dn488821 (Populus tremula)
dn488428
bu887478
dn498110

Populus alba x tremula fragment ***NTMC2Type6***
cf228291

Populus euphratica fragment ***NTMC2Type6***
aj771780

Populus tremula fragment ***NTMC2Type6***
dn498545
bu864149

Populus alba x tremula fragment ***NTMC2Type6***
cx653849

Prosopis juliflora fragment ***NTMC2Type1***
dw360443

Prunus persica fragment ***NTMC2Type1***
dy647415
bu573031 (Prunus dulcis)

Prunus persica fragment ***NTMC2Type2***
bu039411
bu041218
cb820623 (Prunus armeniaca)

Prunus dulcis fragment ***NtermTM-C2Type4***
bq641211

Prunus persica fragment ***NtermTM-C2Type4***
bu041457

Prunus persica fragment ***NtermTM-C2Type5***
dy634446
dy644385
cv045766 (Prunus armeniaca)
cb818726 (Prunus armeniaca)

Prunus armeniaca fragment ***NtermTM-C2Type6***
cb822046

Quercus robur fragment ***NtermTM-C2Type1***
dn950944

Ricinus communis fragment ***NtermTM-C2Type1***
t24180
t24165

Robinia pseudoacacia fragment ***NtermTM-C2Type2***
bi642821

Thellungiella salsuginea fragment ***NtermTM-C2Type1***
dn777874
dn778385

Thellungiella salsuginea fragment ***NtermTM-C2Type1***
dn775065

Thellungiella salsuginea fragment ***NtermTM-C2Type2***
dn776393

Thellungiella salsuginea fragment ***NtermTM-C2Type4***
dn775981

Theobroma cacao fragment ***NtermTM-C2Type1***
ca795197

Thlaspi caerulescens fragment ***NtermTM-C2Type1***
dn927210

Thlaspi caerulescens fragment ***NtermTM-C2Type4***
dn926159

Thlaspi caerulescens fragment ***NtermTM-C2Type4***
dn926173

Trifolium pratense fragment ***NtermTM-C2Type1***
bb927984
bb927289

Trifolium pratense fragment ***NtermTM-C2Type1***
bb909489

Trifolium pratense fragment ***NtermTM-C2Type2***
bb903463

Trifolium pratense fragment ***NtermTM-C2Type2***
bb904330

Trifolium pratense fragment ***NtermTM-C2Type3***
bb914584

Trifolium pratense fragment ***NtermTM-C2Type3***
bb914693

Trifolium pratense fragment ***NtermTM-C2Type3***
bb904294

Trifolium pratense fragment ***NtermTM-C2Type4***
bb904726
bb904838

Trifolium pratense fragment ***NtermTM-C2Type5***
bb924716
bb905851

Trifolium pratense fragment ***NtermTM-C2Type6***
bb910859

Vitis vinifera Full Length ***NtermTM-C2Type1***

MGIVSTILGFCGFGVGISIGLLIGYYLFIYFQPTDVKDPIVRPLVEQDSKTLQRLLPEIP
QWVKNPDYDRVDWLNKFIENMWPYLDKAICKTAKNIAKPIIAEQIPKYKIDSVEFEALTL
GSLPPTFQGMKVYATDEKELIMELSMKWAGNPNITVAVKAFGLRATVQVVDLQVFAAPRI
TLKPLVPSFPCFANIFVSLMEKPHVDFGLKLLGADVMAIPGLYRLVQELIKDQVANMYLW
PKTLEVPIMDPAKAMKKPVGILSVKVVRAMKLKKxDLMGASDPYVKMKLTEDKLPSKKTT
VKLKNLNPEWNEEFNMVVKDPESQALEVNVYDWEQVGKHDKMGMNVIPLKELTPDEPKVL
TLDLLKNLDPNDVQNEKSRGQIVLEALYKPFKDTEIPKDLEDPNAIEKAPDGTPAGGGLL
VIIVHEAQEVEGKHHTNPYVRLLFRGEERKTKYIKKNRDPRWEEEFTFMLEEPPTNDRIH
VEVVSTSSRMGLLHPKETLGYVDINLSDVVSNKRINEKYHLIDSKNGKIQIELQWRTST

ATGGGTATTGTGAGTACAATACTGGGTTTTTGTGGATTTGGTGTGGGGATCTCAATTGGG
CTTTTGATTGGGTATTACCTCTTCATCTACTTCCAACCCACTGATGTGAAGGATCCTATA
GTTCGTCCTCTAGTTGAACAAGATTCAAAAACTCTGCAACGCTTGCTTCCAGAGATACCC
CAATGGGTGAAAAATCCAGACTATGATCGAGTTGACTGGCTTAACAAATTCATAGAAAAT
ATGTGGCCTTACCTGGACAAGGCAATTTGCAAGACTGCGAAGAACATAGCAAAACCTATC
ATTGCTGAGCAAATTCCAAAATACAAAATTGATTCAGTTGAATTTGAAGCACTCACCTTG
GGCTCCCTACCACCTACATTTCAAGGAATGAAAGTCTATGCTACTGATGAAAAGGAGTTG
ATCATGGAACTATCCATGAAGTGGGCAGGGAATCCTAACATCACTGTTGCAGTAAAAGCA
TTTGGGTTGAGAGCCACTGTGCAGGTGGTTGATTTGCAAGTATTTGCTGCTCCTCGCATC
ACCCTGAAGCCTTTGGTTCCATCATTTCCTTGTTTCGCCAATATATTTGTGTCTCTCATG
GAGAAGCCACATGTTGACTTTGGACTAAAGCTGCTAGGAGCAGACGTAATGGCCATTCCT
GGCCTGTATCGGTTGGTGCAGGAGCTTATCAAAGATCAGGTTGCAAATATGTACCTATGG
CCCAAAACCCTTGAAGTTCCAATAATGGATCCTGCAAAAGCCATGAAAAAGCCTGTTGGC
ATTCTCAGTGTGAAGGTTGTTAGGGCAATGAAGCTAAAAAAGAAnGATCTTATGGGGGCA
TCAGATCCTTATGTTAAAATGAAGTTGACTGAGGACAAACTTCCTTCAAAGAAAACTACT
GTGAAACTTAAAAACTTGAATCCTGAATGGAATGAGGAGTTTAATATGGTTGTTAAAGAT
CCAGAATCTCAAGCATTAGAGGTCAACGTCTATGATTGGGAACAGGTTGGTAAACATGAT
AAGATGGGCATGAATGTCATTCCCCTGAAAGAGCTTACACCTGATGAGCCAAAAGTTTTG
ACACTTGATCTACTAAAAAACTTGGACCCGAATGATGTTCAAAATGAGAAGTCACGGGGA
CAGATTGTTTTAGAAGCTCTGTACAAACCTTTTAAGGACACTGAAATACCAAAGGATCTT
GAAGATCCCAATGCGATAGAAAAGGCCCCTGACGGAACACCTGCTGGTGGTGGTTTGCTT
GTAATCATTGTCCATGAAGCTCAGGAGGTTGAGGGAAAGCATCACACAAATCCATATGTT
CGATTACTTTTCAGGGGAGAGGAGAGAAAAACCAAGTACATAAAGAAAAACAGAGACCCC
AGATGGGAAGAGGAGTTTACATTTATGCTGGAGGAACCACCTACCAACGATAGAATACAT
GTAGAAGTTGTTAGCACCTCATCAAGGATGGGCTTACTACATCCTAAGGAAACTCTGGGT
TATGTGGATATAAACCTCTCAGACGTTGTTAGCAACAAACGGATCAACGAGAAATACCAT
CTAATTGACTCAAAGAACGGAAAGATTCAAATCGAGTTGCAATGGCGAACTTCAACATAA

Transcripts
cv093024 (Vitis shuttleworthii)
cv093172 (Vitis shuttleworthii)
cf608601
dt012400

cf200738 (Vitis hybrid)
cf200781 (Vitis hybrid)
cf200682 (Vitis hybrid)
cf373318
dt022769
cf404878

Vitis vinifera fragment ***NtermTM-C2Type1***
cn007028

Vitis vinifera fragment ***NtermTM-C2Type2***
bq797488

Vitis vinifera fragment ***NtermTM-C2Type3***
dt011047
dt010069
dt009696
cv096977 (Vitis shuttleworthii)

Vitis vinifera Full Length ***NtermTM-C2Type4***

MGLISGILMGTIFGIALMAGWVHMMRYRSIKRVAKAVDIKLLGSLNREDLKKICGDNFPE
WISFPVYEQVKWLNKQLTKLWPFVADAATLVIRESVEPLLEDYRPPGITSLKFSKLSLGN
VAPKIEGIRVQSLKKGQIIMDIDLRWGGDPSIILAVEAALVASIPIQLKDLQVFTVARVI
FQLAEEIPCISAVIVAPLSEPKPRIDYTLKAVGGSLTALPGISDMIDDTVNTIITDMLQW
PHRIVVPIGGMPVDTSELELKPQGKLTLTIVKANDLKNMEMIGKSDPYVVVHIRPLFKIK
TKVIENNLNPVWNQTFELIAEDKETQSLILEVIDKDITQDKRLGIAKLPLNDLEAENPKE
IELRLLPSLDMLKIKDKKDRGTITIKVLYHAFNKEEQMAALEEEKRILEERKKLKEAGVI
GSTMDALDGAASLVGSGIGLVGSGVGAGVGLVGTGLGAGVGIVGSGLGAVGSGLSKAGKF
MGRSITGQSSSNKRNGSTTPVNNTQENGGVKP

ATGGGTTTGATTTCTGGGATTTTGATGGGGACGATCTTTGGGATCGCATTGATGGCGGGG
TGGGTTCATATGATGAGATACCGGAGCATCAAGCGCGTTGCCAAGGCAGTTGATATAAAA
CTCCTTGGATCCCTTAACAGAGAAGATTTGAAGAAAATTTGTGGTGATAATTTTCCTGAA
TGGATATCTTTCCCTGTGTATGAACAGGTGAAATGGCTCAACAAGCAACTTACCAAATTA
TGGCCATTTGTTGCAGATGCAGCAACATTGGTAATTAGAGAATCTGTTGAGCCCCTTCTG
GAAGACTACCGGCCTCCAGGAATTACTTCATTAAAGTTCAGTAAGCTCTCTCTTGGAAAT
GTGGCACCCAAGATTGAAGGAATTCGTGTTCAAAGCCTTAAGAAAGGGCAAATTATAATG
GATATCGACTTGCGATGGGGTGGTGATCCAAGCATTATTTTAGCTGTTGAAGCTGCTCTT
GTTGCTTCAATACCCATTCAGTTGAAGGATCTCCAAGTTTTCACTGTTGCTCGTGTTATC
TTCCAACTTGCAGAAGAGATACCTTGTATTTCTGCTGTTATTGTTGCTCCACTTTCTGAG
CCAAAGCCAAGAATTGATTATACTCTGAAGGCTGTTGGTGGAAGTTTAACAGCTCTCCCT
GGAATTTCAGATATGATTGATGATACTGTGAATACCATTATCACAGATATGCTCCAATGG
CCCCACAGGATTGTTGTTCCAATTGGTGGTATGCCTGTGGATACAAGTGAACTAGAGCTA
AAACCACAAGGGAAGCTTACTTTGACAATAGTAAAAGCAAATGATCTGAAGAACATGGAA
ATGATTGGAAAATCTGATCCGTATGTAGTTGTACATATTCGGCCACTATTCAAAATTAAA
ACAAAGGTTATTGAAAACAACCTGAATCCTGTTTGGAATCAAACATTTGAGTTGATTGCG
GAAGATAAGGAGACACAATCGCTAATCCTTGAGGTTATTGACAAGGACATCACACAAGAT
AAGCGGTTGGGCATAGCAAAGCTACCTTTGAATGATCTGGAAGCTGAAAACCCAAAAGAG
ATTGAACTGAGACTGCTTCCGTCACTTGATATGCTTAAGATAAAAGATAAGAAGGATAGA
GGAACCATCACCATTAAGGTTTTGTATCATGCATTTAACAAGGAGGAGCAAATGGCAGCT
CTAGAAGAAGAGAAGAGAATCCTAGAGGAGAGAAAGAAACTGAAAGAAGCAGGAGTCATA
GGGAGCACAATGGATGCACTTGATGGAGCAGCATCGCTGGTCGGGTCTGGAATTGGCCTT
GTAGGCAGTGGAGTTGGCGCTGGAGTTGGGCTTGTGGGCACCGGCCTTGGGGCTGGAGTT
GGGATTGTGGGAAGCGGCCTTGGAGCTGTAGGCAGCGGTCTGAGCAAAGCCGGAAAGTTC
ATGGGCAGGAGCATCACCGGCCAATCTAGCAGCAACAAGAGGAATGGCAGCACAACTCCA
GTGAACAACACCCAAGAAAATGGCGGTGTGAAGCCAACATAG

Transcripts
dt010740
dt010531
cf606617
cf568792 (Vitis shuttleworthii)
cn604229 (Vitis shuttleworthii)
cb289420 (Vitis aestivalis)
dt010316
cx016299
cn603730 (Vitis shuttleworthii)
cv096730 (Vitis shuttleworthii)

cv098059 (Vitis shuttleworthii)
dv220351
dv223057
dt026095
cb919334
cf415695
cd716647
cd010446
cd007130

Vitis vinifera fragment ***NtermTM-C2Type5***
cb340403
cb340153

Vitis vinifera fragment ***NtermTM-C2Type5***
cb919459

Vitis vinifera fragment ***NtermTM-C2Type6***
cb009133
cd013224

Vitis vinifera fragment ***NtermTM-C2Type6***
cf603985

Vitis shuttleworthii fragment ***NtermTM-C2Type6***
cv100672
