# Supplementary material for: Evolutionary genomics of plant genes encoding N-terminal-TM-C2 domain proteins and the similar FAM62 genes and synaptotagmin genes of metazoans
Source: BMC Genomics. 2007 Jul 31;8:259. doi: 10.1186/1471-2164-8-259 (PMC1976326; doi:10.1186/1471-2164-8-259)
Supplement: Additional file 2 — Plant species. [file 1471-2164-8-259-S2.pdf]

Additional file 2 - plant species
Taxonomy from NCBI7

Physcomitrella patens
Eukaryota; Viridiplantae; Streptophyta; Embryophyta; Bryophyta; Bryopsida; Funariidae; Funariales; Funariaceae; Physcomitrella

Tortula ruralis
Eukaryota; Viridiplantae; Streptophyta; Embryophyta; Bryophyta; Bryopsida; Dicranidae; Pottiales; Pottiaceae; Tortula

Selaginella lepidophylla
Eukaryota; Viridiplantae; Streptophyta; Embryophyta; Tracheophyta; Lycopodiophyta; Isoetopsida; Selaginellales; Selaginellaceae; Selaginella

Adiantum capillus-veneris
Eukaryota; Viridiplantae; Streptophyta; Embryophyta; Tracheophyta; Moniliformopses; Filicophyta; Filicopsida; Filicales; Pteridaceae; Adiantum

Ceratopteris richardii
Eukaryota; Viridiplantae; Streptophyta; Embryophyta; Tracheophyta; Moniliformopses; Filicophyta; Filicopsida; Filicales; Pteridaceae; Ceratopteris

Cycas rumphii
Eukaryota; Viridiplantae; Streptophyta; Embryophyta; Tracheophyta; Spermatophyta; Cycadophyta; Cycadales; Cycadaceae; Cycas

Ginkgo biloba
Eukaryota; Viridiplantae; Streptophyta; Embryophyta; Tracheophyta; Spermatophyta; Ginkgophyta; Ginkgoales; Ginkgoaceae; Ginkgo

Welwitschia mirabilis
Eukaryota; Viridiplantae; Streptophyta; Embryophyta; Tracheophyta; Spermatophyta; Gnetophyta; Gnetopsida; Welwitschiales; Welwitschiaceae; Welwitschia

Cryptomeria japonica
Eukaryota; Viridiplantae; Streptophyta; Embryophyta; Tracheophyta; Spermatophyta; Coniferopsida; Coniferales; Cupressaceae; Cryptomeria

Picea glauca
Eukaryota; Viridiplantae; Streptophyta; Embryophyta; Tracheophyta; Spermatophyta; Coniferopsida; Coniferales; Pinaceae; Picea

Pinus taeda
Eukaryota; Viridiplantae; Streptophyta; Embryophyta; Tracheophyta; Spermatophyta; Coniferopsida; Coniferales; Pinaceae; Pinus; Pinus

Pseudotsuga menziesii
Eukaryota; Viridiplantae; Streptophyta; Embryophyta; Tracheophyta; Spermatophyta; Coniferopsida; Coniferales; Pinaceae; Pseudotsuga

Amborella trichopoda
Eukaryota; Viridiplantae; Streptophyta; Embryophyta; Tracheophyta; Spermatophyta; Magnoliophyta; basal Magnoliophyta; Amborellales; Amborellaceae; Amborella

Nuphar advena
Eukaryota; Viridiplantae; Streptophyta; Embryophyta; Tracheophyta; Spermatophyta; Magnoliophyta; basal Magnoliophyta; Nymphaeales; Nymphaeaceae; Nuphar

Liriodendron tulipifera
Eukaryota; Viridiplantae; Streptophyta; Embryophyta; Tracheophyta; Spermatophyta; Magnoliophyta; magnoliids; Magnoliales; Magnoliaceae; Liriodendron

Persea americana
Eukaryota; Viridiplantae; Streptophyta; Embryophyta; Tracheophyta; Spermatophyta; Magnoliophyta; magnoliids; Laurales; Lauraceae; Persea

Acorus americanus
Eukaryota; Viridiplantae; Streptophyta; Embryophyta; Tracheophyta; Spermatophyta; Magnoliophyta; Liliopsida; Acoraceae; Acorus

Agrostis stolonifera
Eukaryota; Viridiplantae; Streptophyta; Embryophyta; Tracheophyta; Spermatophyta; Magnoliophyta; Liliopsida; Poales; Poaceae; BEP clade; Pooideae; Aveneae; Agrostis

Allium cepa
Eukaryota; Viridiplantae; Streptophyta; Embryophyta; Tracheophyta; Spermatophyta; Magnoliophyta; Liliopsida; Asparagales; Alliaceae; Allium

Ananas comosus
Eukaryota; Viridiplantae; Streptophyta; Embryophyta; Tracheophyta; Spermatophyta; Magnoliophyta; Liliopsida; Poales; Bromeliaceae; Ananas

Asparagus officinalis
Eukaryota; Viridiplantae; Streptophyta; Embryophyta; Tracheophyta; Spermatophyta; Magnoliophyta; Liliopsida; Asparagales; Asparagaceae; Asparagus

Avena sativa
Eukaryota; Viridiplantae; Streptophyta; Embryophyta; Tracheophyta; Spermatophyta; Magnoliophyta; Liliopsida; Poales; Poaceae; BEP clade; Pooideae; Aveneae; Avena

Brachypodium distachyon
Eukaryota; Viridiplantae; Streptophyta; Embryophyta; Tracheophyta; Spermatophyta; Magnoliophyta; Liliopsida; Poales; Poaceae; BEP clade; Pooideae; Brachypodieae; Brachypodium

Crocus sativus
Eukaryota; Viridiplantae; Streptophyta; Embryophyta; Tracheophyta; Spermatophyta; Magnoliophyta; Liliopsida; Asparagales; Iridaceae; Crocus

Curcuma longa
Eukaryota; Viridiplantae; Streptophyta; Embryophyta; Tracheophyta; Spermatophyta; Magnoliophyta; Liliopsida; Zingiberales; Zingiberaceae; Curcuma

Cynodon dactylon
Eukaryota; Viridiplantae; Streptophyta; Embryophyta; Tracheophyta; Spermatophyta; Magnoliophyta; Liliopsida; Poales; Poaceae; PACCAD clade; Chloridoideae; Cynodonteae; Cynodon

Eragrostis tef
Eukaryota; Viridiplantae; Streptophyta; Embryophyta; Tracheophyta; Spermatophyta; Magnoliophyta; Liliopsida; Poales; Poaceae; PACCAD clade; Chloridoideae; Eragrostideae; Eragrostis

Festuca arundinacea
Eukaryota; Viridiplantae; Streptophyta; Embryophyta; Tracheophyta; Spermatophyta; Magnoliophyta; Liliopsida; Poales; Poaceae; BEP clade; Pooideae; Poeae; Festuca

Hordeum vulgare
Eukaryota; Viridiplantae; Streptophyta; Embryophyta; Tracheophyta; Spermatophyta; Magnoliophyta; Liliopsida; Poales; Poaceae; BEP clade; Pooideae; Triticeae; Hordeum

Leymus chinensis
Eukaryota; Viridiplantae; Streptophyta; Embryophyta; Tracheophyta; Spermatophyta; Magnoliophyta; Liliopsida; Poales; Poaceae; BEP clade; Pooideae; Triticeae; Leymus

Lilium longiflorum
Eukaryota; Viridiplantae; Streptophyta; Embryophyta; Tracheophyta; Spermatophyta; Magnoliophyta; Liliopsida; Liliales; Liliaceae; Lilium

Lolium temulentum
Eukaryota; Viridiplantae; Streptophyta; Embryophyta; Tracheophyta; Spermatophyta; Magnoliophyta; Liliopsida; Poales; Poaceae; BEP clade; Pooideae; Poeae; Lolium

Lycoris longituba
Eukaryota; Viridiplantae; Streptophyta; Embryophyta; Tracheophyta; Spermatophyta; Magnoliophyta; Liliopsida; Asparagales; Amaryllidaceae; Lycoris

Musa acuminata
Eukaryota; Viridiplantae; Streptophyta; Embryophyta; Tracheophyta; Spermatophyta; Magnoliophyta; Liliopsida; Zingiberales; Musaceae; Musa

Oryza sativa
Eukaryota; Viridiplantae; Streptophyta; Embryophyta; Tracheophyta; Spermatophyta; Magnoliophyta; Liliopsida; Poales; Poaceae; BEP clade; Ehrhartoideae; Oryzeae; Oryza

Panicum virgatum
Eukaryota; Viridiplantae; Streptophyta; Embryophyta; Tracheophyta; Spermatophyta; Magnoliophyta; Liliopsida; Poales; Poaceae; PACCAD clade; Panicoideae; Paniceae; Panicum

Pennisetum glaucum
Eukaryota; Viridiplantae; Streptophyta; Embryophyta; Tracheophyta; Spermatophyta; Magnoliophyta; Liliopsida; Poales; Poaceae; PACCAD clade; Panicoideae; Paniceae; Pennisetum

Saccharum officinarum
Eukaryota; Viridiplantae; Streptophyta; Embryophyta; Tracheophyta; Spermatophyta; Magnoliophyta; Liliopsida; Poales; Poaceae; PACCAD clade; Panicoideae; Andropogoneae; Saccharum; Saccharum officinarum complex

Sorghum bicolor
Eukaryota; Viridiplantae; Streptophyta; Embryophyta; Tracheophyta; Spermatophyta; Magnoliophyta; Liliopsida; Poales; Poaceae; PACCAD clade; Panicoideae; Andropogoneae; Sorghum

Triticum aestivum
Eukaryota; Viridiplantae; Streptophyta; Embryophyta; Tracheophyta; Spermatophyta; Magnoliophyta; Liliopsida; Poales; Poaceae; BEP clade; Pooideae; Triticeae; Triticum

Zea mays
Eukaryota; Viridiplantae; Streptophyta; Embryophyta; Tracheophyta; Spermatophyta; Magnoliophyta; Liliopsida; Poales; Poaceae; PACCAD clade; Panicoideae; Andropogoneae; Zea

Zingiber officinale
Eukaryota; Viridiplantae; Streptophyta; Embryophyta; Tracheophyta; Spermatophyta; Magnoliophyta; Liliopsida; Zingiberales; Zingiberaceae; Zingiber

Zantedeschia aethiopica
Eukaryota; Viridiplantae; Streptophyta; Embryophyta; Tracheophyta; Spermatophyta; Magnoliophyta; Liliopsida; Araceae; Aroideae; Zantedeschieae; Zantedeschia

Aquilegia formosa x Aquilegia pubescens
Eukaryota; Viridiplantae; Streptophyta; Embryophyta; Tracheophyta; Spermatophyta; Magnoliophyta; eudicotyledons; Ranunculales; Ranunculaceae; Aquilegia

Eschscholzia californica
Eukaryota; Viridiplantae; Streptophyta; Embryophyta; Tracheophyta; Spermatophyta; Magnoliophyta; eudicotyledons; Ranunculales; Papaveraceae; Eschscholzioideae; Eschscholzia

Beta vulgaris
Eukaryota; Viridiplantae; Streptophyta; Embryophyta; Tracheophyta; Spermatophyta; Magnoliophyta; eudicotyledons; core eudicotyledons; Caryophyllales; Amaranthaceae; Beta

Mesembryanthemum crystallinum
Eukaryota; Viridiplantae; Streptophyta; Embryophyta; Tracheophyta; Spermatophyta; Magnoliophyta; eudicotyledons; core eudicotyledons; Caryophyllales; Aizoaceae; Mesembryanthemum

Ribes americanum
Eukaryota; Viridiplantae; Streptophyta; Embryophyta; Tracheophyta; Spermatophyta; Magnoliophyta; eudicotyledons; core eudicotyledons; Saxifragales; Grossulariaceae; Ribes

Antirrhinum majus
Eukaryota; Viridiplantae; Streptophyta; Embryophyta; Tracheophyta; Spermatophyta; Magnoliophyta; eudicotyledons; core eudicotyledons; asterids; lamiids; Lamiales; Plantaginaceae; Antirrhineae; Antirrhinum

Apium graveolens
Eukaryota; Viridiplantae; Streptophyta; Embryophyta; Tracheophyta; Spermatophyta; Magnoliophyta; eudicotyledons; core eudicotyledons; asterids; campanulids; Apiales; Apiaceae; Apioideae; apioid superclade; Apium clade; Apium

Capsicum annuum
Eukaryota; Viridiplantae; Streptophyta; Embryophyta; Tracheophyta; Spermatophyta; Magnoliophyta; eudicotyledons; core eudicotyledons; asterids; lamiids; Solanales; Solanaceae; Capsicum

Coffea canephora
Eukaryota; Viridiplantae; Streptophyta; Embryophyta; Tracheophyta; Spermatophyta; Magnoliophyta; eudicotyledons; core eudicotyledons; asterids; lamiids; Gentianales; Rubiaceae; Ixoroideae; Coffeeae; Coffea

Gerbera hybrid cv. 'Terra Regina'
Eukaryota; Viridiplantae; Streptophyta; Embryophyta; Tracheophyta; Spermatophyta; Magnoliophyta; eudicotyledons; core eudicotyledons; asterids; campanulids; Asterales; Asteraceae; Mutisioideae; Mutisieae; Gerbera

Hedyotis terminalis
Eukaryota; Viridiplantae; Streptophyta; Embryophyta; Tracheophyta; Spermatophyta; Magnoliophyta; eudicotyledons; core eudicotyledons; asterids; lamiids; Gentianales; Rubiaceae; Rubioideae; Spermacoceae; Hedyotis

Helianthus annuus
Eukaryota; Viridiplantae; Streptophyta; Embryophyta; Tracheophyta; Spermatophyta; Magnoliophyta; eudicotyledons; core eudicotyledons; asterids; campanulids; Asterales; Asteraceae; Asteroideae; Heliantheae; Helianthus

Ipomoea nil
Eukaryota; Viridiplantae; Streptophyta; Embryophyta; Tracheophyta; Spermatophyta; Magnoliophyta; eudicotyledons; core eudicotyledons; asterids; lamiids; Solanales; Convolvulaceae; Ipomoeeae; Ipomoea

Lactuca sativa
Eukaryota; Viridiplantae; Streptophyta; Embryophyta; Tracheophyta; Spermatophyta; Magnoliophyta; eudicotyledons; core eudicotyledons; asterids; campanulids; Asterales; Asteraceae; Cichorioideae; Cichorieae; Lactuca

Lycopersicon esculentum
Eukaryota; Viridiplantae; Streptophyta; Embryophyta; Tracheophyta; Spermatophyta; Magnoliophyta; eudicotyledons; core eudicotyledons; asterids; lamiids; Solanales; Solanaceae; Solanum; Lycopersicon

Mimulus guttatus
Eukaryota; Viridiplantae; Streptophyta; Embryophyta; Tracheophyta; Spermatophyta; Magnoliophyta; eudicotyledons; core eudicotyledons; asterids; lamiids; Lamiales; Phrymaceae; Mimulus

Nicotiana tabacum
Eukaryota; Viridiplantae; Streptophyta; Embryophyta; Tracheophyta; Spermatophyta; Magnoliophyta; eudicotyledons; core eudicotyledons; asterids; lamiids; Solanales; Solanaceae; Nicotiana

Ocimum basilicum
Eukaryota; Viridiplantae; Streptophyta; Embryophyta; Tracheophyta; Spermatophyta; Magnoliophyta; eudicotyledons; core eudicotyledons; asterids; lamiids; Lamiales; Lamiaceae; Nepetoideae; Ocimeae; Ocimum

Panax ginseng
Eukaryota; Viridiplantae; Streptophyta; Embryophyta; Tracheophyta; Spermatophyta; Magnoliophyta; eudicotyledons; core eudicotyledons; asterids; campanulids; Apiales; Araliaceae; Panax

Petunia x hybrida
Eukaryota; Viridiplantae; Streptophyta; Embryophyta; Tracheophyta; Spermatophyta; Magnoliophyta; eudicotyledons; core eudicotyledons; asterids; lamiids; Solanales; Solanaceae; Petunia

Solanum tuberosum
Eukaryota; Viridiplantae; Streptophyta; Embryophyta; Tracheophyta; Spermatophyta; Magnoliophyta; eudicotyledons; core eudicotyledons; asterids; lamiids; Solanales; Solanaceae; Solanum

Taraxacum officinale
Eukaryota; Viridiplantae; Streptophyta; Embryophyta; Tracheophyta; Spermatophyta; Magnoliophyta; eudicotyledons; core eudicotyledons; asterids; campanulids; Asterales; Asteraceae; Cichorioideae; Cichorieae; Taraxacum

Triphysaria versicolor
Eukaryota; Viridiplantae; Streptophyta; Embryophyta; Tracheophyta; Spermatophyta; Magnoliophyta; eudicotyledons; core eudicotyledons; asterids; lamiids; Lamiales; Orobanchaceae; Rhinantheae; Triphysaria

Vaccinium corymbosum
Eukaryota; Viridiplantae; Streptophyta; Embryophyta; Tracheophyta; Spermatophyta; Magnoliophyta; eudicotyledons; core eudicotyledons; asterids; Ericales; Ericaceae; Vaccinioideae; Vaccinieae; Vaccinium

Zinnia elegans
Eukaryota; Viridiplantae; Streptophyta; Embryophyta; Tracheophyta; Spermatophyta; Magnoliophyta; eudicotyledons; core eudicotyledons; asterids; campanulids; Asterales; Asteraceae; Asteroideae; Heliantheae; Zinnia

Arabidopsis thaliana
Eukaryota; Viridiplantae; Streptophyta; Embryophyta; Tracheophyta; Spermatophyta; Magnoliophyta; eudicotyledons; core eudicotyledons; rosids; eurosids II; Brassicales; Brassicaceae; Arabidopsis

Brassica napus
Eukaryota; Viridiplantae; Streptophyta; Embryophyta; Tracheophyta; Spermatophyta; Magnoliophyta; eudicotyledons; core eudicotyledons; rosids; eurosids II; Brassicales; Brassicaceae; Brassica

Bruguiera gymnorhiza
Eukaryota; Viridiplantae; Streptophyta; Embryophyta; Tracheophyta; Spermatophyta; Magnoliophyta; eudicotyledons; core eudicotyledons; rosids; eurosids I; Malpighiales; Rhizophoraceae; Bruguiera

Citrus sinensis
Eukaryota; Viridiplantae; Streptophyta; Embryophyta; Tracheophyta; Spermatophyta; Magnoliophyta; eudicotyledons; core eudicotyledons; rosids; eurosids II; Sapindales; Rutaceae; Citrus

Cucumis melo
Eukaryota; Viridiplantae; Streptophyta; Embryophyta; Tracheophyta; Spermatophyta; Magnoliophyta; eudicotyledons; core eudicotyledons; rosids; eurosids I; Cucurbitales; Cucurbitaceae; Cucumis

Eucalyptus grandis
Eukaryota; Viridiplantae; Streptophyta; Embryophyta; Tracheophyta; Spermatophyta; Magnoliophyta; eudicotyledons; core eudicotyledons; rosids; Myrtales; Myrtaceae; Eucalyptus

Euphorbia tirucalli
Eukaryota; Viridiplantae; Streptophyta; Embryophyta; Tracheophyta; Spermatophyta; Magnoliophyta; eudicotyledons; core eudicotyledons; rosids; eurosids I; Malpighiales; Euphorbiaceae; Euphorbioideae; Euphorbieae; Euphorbia

Fragaria vesca
Eukaryota; Viridiplantae; Streptophyta; Embryophyta; Tracheophyta; Spermatophyta; Magnoliophyta; eudicotyledons; core eudicotyledons; rosids; eurosids I; Rosales; Rosaceae; Rosoideae; Fragaria

Glycine max
Eukaryota; Viridiplantae; Streptophyta; Embryophyta; Tracheophyta; Spermatophyta; Magnoliophyta; eudicotyledons; core eudicotyledons; rosids; eurosids I; Fabales; Fabaceae; Papilionoideae; Phaseoleae; Glycine

Gossypium hirsutum
Eukaryota; Viridiplantae; Streptophyta; Embryophyta; Tracheophyta; Spermatophyta; Magnoliophyta; eudicotyledons; core eudicotyledons; rosids; eurosids II; Malvales; Malvaceae; Malvoideae; Gossypium

Linum usitatissimum
Eukaryota; Viridiplantae; Streptophyta; Embryophyta; Tracheophyta; Spermatophyta; Magnoliophyta; eudicotyledons; core eudicotyledons; rosids; eurosids I; Malpighiales; Linaceae; Linum

Lotus japonicus
Eukaryota; Viridiplantae; Streptophyta; Embryophyta; Tracheophyta; Spermatophyta; Magnoliophyta; eudicotyledons; core eudicotyledons; rosids; eurosids I; Fabales; Fabaceae; Papilionoideae; Loteae; Lotus

Lupinus albus
Eukaryota; Viridiplantae; Streptophyta; Embryophyta; Tracheophyta; Spermatophyta; Magnoliophyta; eudicotyledons; core eudicotyledons; rosids; eurosids I; Fabales; Fabaceae; Papilionoideae; Genisteae; Lupinus

Malus x domestica
Eukaryota; Viridiplantae; Streptophyta; Embryophyta; Tracheophyta; Spermatophyta; Magnoliophyta; eudicotyledons; core eudicotyledons; rosids; eurosids I; Rosales; Rosaceae; Maloideae; Malus

Manihot esculenta
Eukaryota; Viridiplantae; Streptophyta; Embryophyta; Tracheophyta; Spermatophyta; Magnoliophyta; eudicotyledons; core eudicotyledons; rosids; eurosids I; Malpighiales; Euphorbiaceae; Crotonoideae; Manihoteae; Manihot

Medicago truncatula
Eukaryota; Viridiplantae; Streptophyta; Embryophyta; Tracheophyta; Spermatophyta; Magnoliophyta; eudicotyledons; core eudicotyledons; rosids; eurosids I; Fabales; Fabaceae; Papilionoideae; Trifolieae; Medicago

Phaseolus coccineus
Eukaryota; Viridiplantae; Streptophyta; Embryophyta; Tracheophyta; Spermatophyta; Magnoliophyta; eudicotyledons; core eudicotyledons; rosids; eurosids I; Fabales; Fabaceae; Papilionoideae; Phaseoleae; Phaseolus

Pisum sativum
Eukaryota; Viridiplantae; Streptophyta; Embryophyta; Tracheophyta; Spermatophyta; Magnoliophyta; eudicotyledons; core eudicotyledons; rosids; eurosids I; Fabales; Fabaceae; Papilionoideae; Vicieae; Pisum

Poncirus trifoliata
Eukaryota; Viridiplantae; Streptophyta; Embryophyta; Tracheophyta; Spermatophyta; Magnoliophyta; eudicotyledons; core eudicotyledons; rosids; eurosids II; Sapindales; Rutaceae; Poncirus

Populus deltoides
Eukaryota; Viridiplantae; Streptophyta; Embryophyta; Tracheophyta; Spermatophyta; Magnoliophyta; eudicotyledons; core eudicotyledons; rosids; eurosids I; Malpighiales; Salicaceae; Saliceae; Populus

Prosopis juliflora
Eukaryota; Viridiplantae; Streptophyta; Embryophyta; Tracheophyta; Spermatophyta; Magnoliophyta; eudicotyledons; core eudicotyledons; rosids; eurosids I; Fabales; Fabaceae; Mimosoideae; Mimoseae; Prosopis

Prunus persica
Eukaryota; Viridiplantae; Streptophyta; Embryophyta; Tracheophyta; Spermatophyta; Magnoliophyta; eudicotyledons; core eudicotyledons; rosids; eurosids I; Rosales; Rosaceae; Amygdaloideae; Prunus

Quercus robur
Eukaryota; Viridiplantae; Streptophyta; Embryophyta; Tracheophyta; Spermatophyta; Magnoliophyta; eudicotyledons; core eudicotyledons; rosids; eurosids I; Fagales; Fagaceae; Quercus

Ricinus communis
Eukaryota; Viridiplantae; Streptophyta; Embryophyta; Tracheophyta; Spermatophyta; Magnoliophyta; eudicotyledons; core eudicotyledons; rosids; eurosids I; Malpighiales; Euphorbiaceae; Acalyphoideae; Acalypheae; Ricinus

Robinia pseudoacacia
Eukaryota; Viridiplantae; Streptophyta; Embryophyta; Tracheophyta; Spermatophyta; Magnoliophyta; eudicotyledons; core eudicotyledons; rosids; eurosids I; Fabales; Fabaceae; Papilionoideae; Robinieae; Robinia

Thellungiella salsuginea
Eukaryota; Viridiplantae; Streptophyta; Embryophyta; Tracheophyta; Spermatophyta; Magnoliophyta; eudicotyledons; core eudicotyledons; rosids; eurosids II; Brassicales; Brassicaceae; Thellungiella

Theobroma cacao
Eukaryota; Viridiplantae; Streptophyta; Embryophyta; Tracheophyta; Spermatophyta; Magnoliophyta; eudicotyledons; core eudicotyledons; rosids; eurosids II; Malvales; Malvaceae; Byttnerioideae; Theobroma

Thlaspi caerulescens
Eukaryota; Viridiplantae; Streptophyta; Embryophyta; Tracheophyta; Spermatophyta; Magnoliophyta; eudicotyledons; core eudicotyledons; rosids; eurosids II; Brassicales; Brassicaceae; Thlaspi

Trifolium pratense
Eukaryota; Viridiplantae; Streptophyta; Embryophyta; Tracheophyta; Spermatophyta; Magnoliophyta; eudicotyledons; core eudicotyledons rosids; eurosids I; Fabales; Fabaceae; Papilionoideae; Trifolieae; Trifolium

Vitis vinifera
Eukaryota; Viridiplantae; Streptophyta; Embryophyta; Tracheophyta Spermatophyta; Magnoliophyta; eudicotyledons; core eudicotyledons; rosids; Vitaceae; Vitis
