# Supplementary material for: Evolutionary genomics of plant genes encoding N-terminal-TM-C2 domain proteins and the similar FAM62 genes and synaptotagmin genes of metazoans
Source: BMC Genomics. 2007 Jul 31;8:259. doi: 10.1186/1471-2164-8-259 (PMC1976326; doi:10.1186/1471-2164-8-259)
Supplement: Additional file 3 — FAM62 genes. [file 1471-2164-8-259-S3.pdf]

Additional file 3 - *FAM62* genes
Taxonomy from NCBI

translation start/stop codons are underlined
[indicates an alternative splice
] indicates an alternative splice
^ indicates an intron
complementary strand nucleotide positions indicated within parentheses

Caenorhabditis elegans *FAM62*
Gene 118 Craxton, M. BMC Genomics. 2004 Jul 6;5(1):43

Eukaryota; Metazoa; Nematoda; Chromadorea; Rhabditida; Rhabditoidea; Rhabditidae; Peloderinae; Caenorhabditis.

MSWQSYLVPLVGSAILSTFTFFL**^ +1**
GKWDYSFVWVLIIIIASVTKSYLWRKRER**^ +2**
RLIALRATALREREVIMAQLQDLPAWVQFPDTERVEWLNKVIHQLWPYVGEYTKTFMNDF
IIPQVK**^ 0**
AQMPGMFKNFKFTKMDMGDIPCRVGGIKVYTTNVGRDRIIVDMDVAYAGDADFTVSCCGF
TGGMNNIQ**^ 0**
FSGKLRAILKPLLPYPPMVGGVSGTFLEMPKMDFNLTGMGEMVELPGLIDAIRSVINSQ**^ 0**
IAALCVLPNEIVVPLAPDVDVTQLYFPEPD**^ 0**
GVVRLKIIEAKNLENRDISFIKKGKSDPYAEIQVGSQFFKTRTIDDDLNPIWNEYFEAVV
DQADGQKLRIELFDEDQGKDEELGRLSVDLKLVQAKGTIDK**^ 0**
WYPLEGCKHGDLHIKATWMNLSTELRHLEKQEWEAEWGQADKPIHSALLMVYIDSVADLP**^ 0**
YPKSKLEPSPFVEVSLGKETQRTPVKVKTVNPLFQSKFLFFVRHLEGQELKFE**^ 0**
AVDDGTRRSLGSLNIPLTTLLKEPNLEQNQQMHMLTLGVHQSPIVITTRIR**^ 0**
ALIQGKPKKGNNSELGHDVLGEYGN**^ +1**
AFHIERANGKVNGDVVAVEPAKTVNQDAADDEVEIKLNVDNYQ**^ 0**
MLRSDSQGSLNSHGRSNSRLGRLFRSKHEMKKRET**^ +2**
RADENRGEIEIQIDFDDLVNQLKIALIRCRDLMTFDKKDQCNPYVS**^ 0**
VKLVALDGNKEVFKKKTPTAKNTRHPHFDN**^ +2**
HVEIDINPSDLLNHKVVINVKDDTNYGTFVAKPVLGC**^ 0**
LEIRLDSLMNRQLSQRWIPLSVERK

Drosophila melanogaster *FAM62*
Gene 119 Craxton, M. BMC Genomics. 2004 Jul 6;5(1):43

Eukaryota; Metazoa; Arthropoda; Hexapoda; Insecta; Pterygota; Neoptera; Endopterygota; Diptera; Brachycera; Muscomorpha; Ephydroidea; Drosophilidae; Drosophila.

MSDNSPSVPLAEFTIPPDPTETEPFVPLVEVKKMNESPVVTPTTPTGNGTPPTQTTNGNS
IVATKSVSDDNSIFSVFYTLGKKVAIVGSIYLVGYMGWSVAWLIAPVILSVARDQLAKTS
EKKRDIAKASALASEKDVILARIDELPAWVYFPDVERCEWLNK**^ 0**
ILKQVWPNANHFARTLVKETIEPNVALALANYKMHGFRFDRIILGTIPPRIGGVKIYDKN
VDRNEIIMDLDLFYASDCDINFYLGGMKGGIKDFQIHGWVRVVMKPLIRSMPLVGGLQIF
FLNNPNIDFNLVGVIDFMDMPGLSDLLRRIIVEQIGNVMVLPNKLPISLSEEVSAVALKM
PEPE**^ 0**
GILRIHVVEAKDLMKKDISVLGKGKSDPYAIINVGAQEFKTQIIDNNVNPKWDYWCE**^ 0**
ATVFIEMGQFVEIQLKDSDDSKKDENLG**^ +2**
[ACIFTTIGHYIGFSLWDYDQTMPGVQSDDVLG**^ +2]**
RASIDIASVIKKGVVDS**^ 0**
WLTLEDAKHGLLHVRLQWYKLTADPNDLQQILLETQLLRVTSMSSAVLSVFIDSARHLK**^ 0**
QARSSSKPDPYLVCSVNKQKQQTAMIMRDDSPVWEQGFTFLVSNPDNESLNIKIYDQKTG
NDIGQYTYTLSTLLKQFNMEVIQQPFQLQKSGPESKLYMSLSLRILKPGEIDKDSDALEQ
VAALTRSSSVKTPDVAAVSPPAFK**^ 0**
ESQASSKRLSAESPISEEDPVAATKISPAMSASTSSEKPISELATSVLTHRFPDSTSSPG
EHGLGRMQLSIRYSAQRQKLDVTIHKIQKIPLRDPSNIPDPYVKLYLLPGRTKESKRKTS
VIKDNCNPVYDASFEYLISIAELRQTELEVTVCTQKGFLSGGSPIIGM**^ 0**
LKIPLDDAEITTQTGLNSWFDLQPEIRHE

Anopheles gambiae *FAM62*

Eukaryota; Metazoa; Arthropoda; Hexapoda; Insecta; Pterygota; Neoptera; Endopterygota; Diptera; Nematocera; Culicoidea; Culicidae; Anophelinae; Anopheles.

MAGASKELSPEKQSAPPESSEVAKTKDDSIMTLLYSFAKKVVTVGIIYFVGYMGWSVAWL

ITPVILSVARESWRKTNDTRRSVAKASALANDKEVILARLHDLPAW**^ 0**
VFFPDVERCEWLNR**^ 0**
ILKQVWPNANFYAKNLIKESIEPNIQQAMAGYKLNGFKFDRMILGTI**^ 0**
PPRIGGVKVYDKNVSRNEIIMDLDLFYAGDCDISFALSGLRGGIKDFQIHGTVRVIMKPL
ISQMPLIGGLQIFFLNNPNIDFNLVGVVDLLDMPGLSDILRKIIVEQVAAIMVLPNKLPI
VLSDGVPALSLKMPEPE**^ 0**
GVLRIHVVEAKDLMKKDISVLGKGKSDPYAIISVGAQQFRTQTIDNTVNPKWDYWCE**^ 0**
AFIHAESGQTLQVVINDEDAGEDELLG**^ +2**
[AEVNAILRQEIELNLWDYDPGFPGVQNDDFLG**^ +2]**
RATVEISSVTKNGEIDT**^ 0**
WLTLEQAKHGLVHLRMTWFKLSSEKSDLKQALEETQHLRVTSMSTALLTVFIDSAKNLP**^ 0**
QARQQSQPDPYLVLSVGKKNEQTSVQMRTDAPVWEQGFTFLVGNPDNDTLQLKVIDQKTG
NTIGTLTYILSALMEKKNLEIMSQPFQLQKSGPETKIIMSLSLRILKRHREQEPAVTTPD
KGPASEADSVLSRTSSIRTSASHGSQSGTLQQQPSTGDSNAAEAAALSHQGSVRKQDSRK
STTSAIMEQMSIQEEPFVVSTLNTVMMATPPRSPNLSDGGTELLRRSPSTTSSSGSAGLG
RIQLTVAYSVQRQRLLVIVHKINNIPLKDPNNIPDPYVKLYLLPGRSKESKRKTNVVKDN
CDPVFDTTFEYIISNAELVNSELEVTVCTQKGFFGSPVIGM**^ 0**
QKLSLSDPDISSGQGIKAWYDLLPESKFE

ATGGCGGGCGCAAGCAAAGAGCTAAGCCCGGAAAAGCAATCAGCACCGCCGGAGTCCTCC
GAGGTTGCAAAAACGAAGGACGACAGCATCATGACGCTGCTGTACTCGTTCGCGAAGAAG
GTGGTGACGGTCGGTATCATCTACTTCGTCGGCTACATGGGCTGGTCGGTGGCGTGGCTC
ATCACGCCCGTCATCCTGTCGGTGGCACGCGAATCCTGGCGCAAAACCAACGACACACGA
CGCAGCGTAGCGAAGGCGTCCGCTCTCGCCAACGATAAGGAAGTCATCCTGGCCCGGCTG
CACGATCTGCCGGCTTGG
phase 0
aaab01008844.1 67202-67519

GTATTCTTTCCCGACGTTGAACGTTGCGAATGGCTTAACAGG
phase 0
aaab01008844.1 72904-72945

ATCCTGAAACAAGTCTGGCCCAATGCAAACTTTTACGCGAAAAATCTCATCAAGGAATCG
ATCGAACCCAACATCCAGCAGGCGATGGCTGGCTACAAGCTGAACGGATTCAAATTTGAC
CGAATGATTCTGGGAACGATT
phase 0
aaab01008844.1 73047-73187

CCACCACGCATCGGCGGCGTCAAGGTGTACGACAAGAACGTGTCTCGCAATGAAATCATC
ATGGATCTGGACCTGTTCTATGCGGGCGACTGCGATATCAGCTTCGCGCTCAGCGGGCTG
CGGGGTGGCATCAAGGACTTCCAGATTCACGGCACGGTGCGCGTAATAATGAAGCCACTC
ATTTCCCAGATGCCGCTCATCGGCGGGCTGCAGATATTCTTCCTCAACAATCCCAACATT
GACTTTAACCTAGTCGGCGTGGTGGATCTGCTCGATATGCCCGGGCTGAGCGACATACTG
CGAAAGATTATCGTGGAGCAGGTGGCCGCCATCATGGTCCTGCCGAACAAGCTGCCTATC
GTGCTGAGCGATGGGGTGCCGGCACTGTCGTTGAAGATGCCCGAACCCGAG
phase 0
aaab01008844.1 73306-73716

GGCGTACTTAGGATTCATGTCGTCGAGGCGAAGGACCTCATGAAAAAGGACATCAGCGTG
CTCGGCAAGGGCAAATCGGACCCGTACGCCATCATTTCCGTCGGTGCACAGCAGTTCCGC
ACGCAAACCATCGACAACACGGTCAACCCCAAGTGGGACTACTGGTGCGAG
phase 0
aaab01008844.1 74278-74448

GCCTTTATCCACGCGGAAAGTGGTCAAACACTTCAGGTCGTGATCAACGATGAGGACGCC
GGCGAAGATGAACTGTTGGGAAG
phase +2
aaab01008844.1 76511-76593

an alternative to the above
GCTGAAGTCAATGCCATTCTTAGGCAAGAGATTGAACTCAACCTGTGGGATTATGATCCA
GGCTTTCCCGGGGTACAGAACGACGATTTCCTTGGCAG
phase +2
aaab01008844.1 77613-77710

AGCAACGGTGGAAATCAGCAGCGTTACCAAGAACGGTGAAATCGATACG

phase 0
aaab01008844.1 80282-80330

TGGCTGACTCTGGAACAAGCAAAGCATGGGTTGGTGCATCTACGCATGACTTGGTTCAAA
CTGAGCTCCGAAAAGTCTGATCTGAAGCAAGCGCTGGAAGAAACGCAACATCTGCGTGTG
ACCTCTATGAGCACCGCACTGCTGACCGTTTTCATCGATTCGGCCAAGAACCTGCCG
phase 0
aaab01008844.1 80506-80682

CAAGCACGCCAACAGTCACAACCGGACCCGTACCTGGTGCTTTCGGTGGGCAAAAAGAAC
GAGCAAACCTCGGTACAGATGCGCACGGATGCGCCGGTCTGGGAGCAGGGCTTTACTTTC
CTGGTGGGCAACCCGGACAACGACACGCTGCAGCTGAAGGTGATCGACCAGAAGACGGGC
AACACCATCGGCACACTGACGTACATCCTGAGCGCACTGATGGAGAAGAAAAATCTCGAG
ATCATGTCACAACCGTTCCAGCTGCAAAAGTCCGGCCCAGAGACGAAGATCATTATGTCG
CTGTCGCTGCGCATCCTGAAGCGCCACCGGGAGCAGGAGCCAGCGGTCACCACGCCGGAC
AAGGGACCGGCGTCCGAGGCGGACAGTGTGCTGTCACGCACCAGCTCCATCCGCACCTCG
GCGTCCCACGGTTCGCAGTCCGGTACGCTGCAGCAGCAACCGTCGACCGGGGACTCGAAC
GCAGCGGAAGCGGCCGCCCTGTCCCATCAGGGTAGCGTCCGGAAGCAGGACTCGCGCAAG
TCAACCACCTCCGCCATCATGGAGCAGATGTCGATCCAGGAGGAACCGTTCGTCGTGTCG
ACGCTGAACACGGTCATGATGGCAACGCCACCGCGCAGCCCGAATCTGAGCGACGGCGGC
ACGGAGCTGTTGCGTCGCAGCCCCAGCACCACGTCCTCCTCCGGCTCGGCTGGCCTCGGA
CGCATCCAGCTGACGGTGGCGTACAGTGTGCAGCGCCAGCGGCTGCTAGTGATCGTGCAC
AAGATCAACAATATTCCGCTGAAAGATCCCAACAACATTCCGGACCCGTACGTGAAGCTG
TACCTGCTGCCGGGCCGGTCGAAGGAATCGAAGCGCAAGACGAACGTGGTAAAGGACAAC
TGCGATCCCGTGTTTGACACCACGTTCGAGTACATCATCTCGAACGCGGAGCTGGTCAAT
TCGGAGCTGGAGGTGACGGTGTGCACACAGAAAGGGTTCTTCGGCAGTCCCGTCATCGGAATG
phase 0
aaab01008844.1 80757-81779

CAAAAACTCTCCCTAAGCGATCCGGACATTTCGAGCGGACAGGGCATCAAGGCGTGGTAC
GATCTGCTGCCGGAGTCCAAGTTTGAGTAA
aaab01008844.1 81851-81940

Apis mellifera *FAM62*

Eukaryota; Metazoa; Arthropoda; Hexapoda; Insecta; Pterygota; Neoptera; Endopterygota; Hymenoptera; Apocrita; Aculeata; Apoidea; Apidae; Apis.

MEDKCEDNKISKSNPWPYMNIGSLTISFLTKLATAGIIWSWGYLNLNIAWLIGPIALIAW
KTERRKDNELKLITAQASVMAKEKELIMSRLDELPSWVYFPDFDRAEWLNK**^ 0**
ILYKVWPNINQFARELCKQSIEPAILEKLAEFKIKGFQFERLVLGRI**^ 0**
PLKIYGIKAYDKNTSRNEVIMDADI**^ +2**
MYAGDCDITFSVGNIKGGIRDFQ**^ 0**
IRGMMRIVMKPLLPVMPIVGGVQAFFLNPPAINFNLIGVADVLDLPGF**^ +2**
NEILRKTIVEQIAAFVVLPNKIVIPLSEAVPIESLKIPEPE**^ 0**
GVLRIHVVEAKHLMKKDIGMLGKGKSDPYAIINIGAQEFRTKTIDNTVNPKWDFWCE**^ 0**
CAVTSAIAQQITVLLWDYDDTKGDESLG**^ +2**
[AVICSCIMQNIMVFLWDRDVLTIPYDDFLG**^ +2]**
RATIEVSxSEKKGNIDT**^ 0**
WISxEQAKHGMIHLRLTWFQLSKNIVDLKA**^ 0**
ALVETQELRVTSMSTALLILYIDSAKNLP**^ 0**
CVRGNKQPDVYLEASIGGNTKRTATMLRSCDPVWEQGFTFLVSNPETGILHIK**^ 0**
ITDEKTNLIVGEMNYNISLLLTQNNLEISQQPYDLQMAEVDSKLILSMSLS**^ 0**
ILKYEEPEPISEEDDDDHDINQLKKKIERQESNISNTLSTSR**^ +2**
NPLKRQSSKDSINSLTPSIGSGAVIIPEELGTAEEELIVVTSAPSSENAQLIHRNPNMTS
SAGDAKLGRIQLSLRYSVQRQKFIIVVHKI**^ +2**
ANLPLPQNDPHNIPDPYVKLYLLPDRHKETKRKTAVMKDNCNPIFDEQFEYVVSQADLNS
RILEVSVCTQKGWLSTGSNVMGQVHINLNEIDVTKSFTSWYDLQPETKD

ATGGAAGATAAATGTGAAGACAATAAAATATCTAAATCAAATCCTTGGCCATATATGAAT
ATTGGTTCATTAACCATATCATTTTTGACTAAATTGGCAACTGCTGGCATTATTTGGAGT
TGGGGATATTTAAATTTAAATATAGCATGGTTAATTGGACCAATTGCTTTGATAGCATGG
AAAACAGAACGTAGGAAAGACAACGAATTAAAATTAATTACTGCCCAAGCTAGTGTTATG
GCTAAAGAAAAAGAACTGATAATGAGCAGATTAGATGAACTACCTTCTTGGGTTTACTTT
CCTGATTTTGATAGAGCTGAATGGTTAAATAAA
phase 0

aadg05006483.1 (46065-45733)

ATTTTATATAAAGTTTGGCCAAACATTAATCAGTTTGCTCGTGAACTTTGCAAACAAAGT
ATAGAACCTGCTATATTAGAAAAATTGGCAGAATTTAAGATAAAAGGATTTCAATTTGAA
AGATTGGTTTTGGGTCGTATT
phase 0
aadg05006483.1 (45640-45500)

CCTTTAAAAATTTATGGTATTAAAGCATATGATAAAAACACTTCAAGAAATGAAGTCATT
ATGGATGCAGATATTAT
phase +2
aadg05006483.1 (45402-45326)

GTATGCTGGTGACTGTGATATTACCTTTTCAGTTGGAAATATAAAAGGTGGTATTAGGGA
CTTTCAG
phase 0
aadg05006483.1 (45187-45121)

ATTCGTGGAATGATGAGAATAGTTATGAAACCATTATTACCTGTAATGCCTATAGTTGGA
GGTGTTCAAGCATTCTTTTTAAATCCACCAGCTATTAATTTTAATTTGATTGGAGTTGCA
GATGTTCTTGATTTGCCTGGATTTAA
phase +2
aadg05006483.1 (45049-44904)

TGAAATTTTAAGAAAAACAATAGTAGAACAAATAGCAGCTTTTGTAGTATTGCCAAACAA
AATTGTTATACCATTAAGTGAAGCAGTACCAATTGAATCATTAAAAATACCAGAACCTGAA
phase 0
aadg05006483.1 (44828-44708)

GGTGTTTTAAGGATTCATGTAGTAGAAGCGAAACATCTTATGAAAAAGGACATAGGGATG
TTAGGTAAAGGTAAATCTGATCCATATGCCATTATAAATATTGGAGCACAAGAATTTAGG
ACAAAAACTATAGATAATACTGTGAATCCAAAATGGGATTTCTGGTGTGAG
phase 0
aadg05006483.1 (44628-44458)

TGTGCCGTGACGTCAGCCATCGCTCAACAAATTACTGTACTACTATGGGACTATGATGAC
ACCAAAGGAGATGAAAGTCTTGGAAG
phase +2
aadg05006483.1 (43944-43859)

an alternative to the above
GCTGTGATCTGCTCATGCATAATGCAGAATATTATGGTGTTTCTTTGGGATAGGGATGTA
TTAACCATCCCTTATGATGACTTCCTTGGCAG
aadg05006483.1 (43210-43119)
phase +2

GGCTACTATTGAAGTTAGTAGnAGTGAAAAAAAAGGAAATATCGATACA
phase 0
aadg05006483.1 (41759-41712)

TGGATTTCATTnGAACAAGCAAAACATGGCATGATTCATTTACGATTGACATGGTTTCAA
CTATCAAAAAATATTGTTGATTTAAAAGCT
phase 0
aadg05006483.1 (41632-41542)

GCTTTAGTGGAAACTCAAGAACTTAGAGTAACATCAATGAGTACTGCTCTTCTTATACTT
TATATTGATTCAGCAAAAAAATTTACCA
phase 0
aadg05006483.1 (41454-41368)

TGTGTTCGAGGAAATAAACAGCCTGATGTTTATCTTGAAGCAAGTATTGGTGGAAATACA
AAAAGAACAGCTACCATGTTACGTTCTTGTGACCCAGTATGGGAACAAGGTTTCACTTTT
TTAGTTAGCAATCCAGAAACTGGTATTTTACATATAAAG
phase 0
aadg05006483.1 (41279-41121)

ATTACAGATGAAAAAACTAATCTCATAGTGGGTGAAATGAATTATAATATTTCTTTACTT
TTAACACAAAATAATCTTGAAATTTCACAACAGCCTTATGATTTGCAAATGGCTGAAGTT
GATAGCAAATTAATATTATCTATGTCATTAAGT
phase 0
aadg05006483.1 (41043-40891)

ATTTTAAAATATGAGGAACCTGAACCTATTTCTGAAGAAGATGATGATGATCATGATATC
AATCAATTAAAGAAAAAAATCGAACGTCAAGAATCAAATATTAGTAATACATTATCAACT
AGTCGTAA
phase +2
aadg05006483.1 (40803-40676)

TCCATTGAAAAGACAATCATCAAAAGATTCAATTAATAGTCTAACACCAAGTATTGGATC
TGGTGCTGTAATTATACCTGAAGAACTAGGTACAGCAGAAGAAGAATTAATTGTCGTTAC
TTCTGCTCCATCCTCTGAAAATGCACAGTTAATTCATAGAAATCCAAACATGACATCATC
TGCAGGTGATGCTAAACTAGGTCGGATACAATTATCTTTACGTTATAGCGTACAAAGACA
GAAATTTATAATTGTCGTACACAAAATAGC
phase +2
aadg05006483.1 (40608-40339)

TAATTTACCTCTGCCACAAAATGATCCGCATAACATACCAGATCCATATGTAAAACTATA
TCTTCTTCCTGATCGTCATAAGGAAACCAAACGTAAAACAGCAGTGATGAAGGATAATTG
TAATCCGATATTCGATGAACAATTTGAATATGTTGTTTCTCAAGCTGATTTAAATAGTCG
TATATTGGAAGTGTCTGTATGTACACAAAAAGGTTGGTTATCGACCGGAAGTAACGTAAT
GGGTCAAGTTCATATAAATTTGAATGAAATTGATGTTACAAAATCATTCACGAGCTGGTA
CGATTTACAACCAGAGACTAAGGATTAG
aadg05006483.1 (40257-39930)

Tribolium castaneum *FAM62* genes

Eukaryota; Metazoa; Arthropoda; Hexapoda; Insecta; Pterygota; Neoptera; Endopterygota; Coleoptera; Polyphaga; Cucujiformia; Tenebrionidae; Tribolium.

Tribolium *FAM62-1*

MSVSKDGSQAMEKRSEGSSILSVIFSAVKK**^ 0**
ASIVGIVYFAGYMQWSVAWFIGPIVLFVIRDQWKKASDRKRNIAKAAALASEKDVVLARL
DDLPAW**^ 0**
VFFPDVERAEWLNR**^ 0**
IIKQVWPNINHYTRDLIRDTIQPILKESLETYKLSGFKFERIILGTV**^ 0**
PFRIGGVKVYDKNVARNEIIMDLDI**^ +2**
FYAGDCDITFYLAGIKGGIRDFQLHGMLRVVMKPLITTIPLVGGLQVFFLNNPDIDFDLI
GIADLLDMPGLSDILRRIVVETVASMMVLPNKFPIKLSDDVDAMELKAPEPE**^ 0**
GVLRVHVVEAKHLMKKDIGVLGKGKSDPYAVVTLGAQEFKTKVIDNSVDPKWDFWCE**^ 0**
FNVLESDGQQLYIHLWDKDETSDDETLG**^ +2**
[AQISSYESQLLTVNVWDWDPGVPGAQNHDYLG**^ +2]**
RATIEVSNIVKKGQDDLWVTLEQAKHGMVHLRLTWLTLSDNYSDLKA**^ 0**
ALEETQQLRVTSMSTALLTIFLDSAKNLP**^ 0**
QARASTKPDPYAVLKVGNTTKETKVLERTIHPVWEQGFSFLVANPESDTLYLTIIDRKTT
NELGQVTYNISKLAKKTKMEVYKEPFSLLKSGPESKVIWSMHLR**^ 0**
VLKRAEGVDDTDSGDLPSLQREDSKVLTSDDVPPTPNPTPEINESATEPIPNYDEMIKAT
AATVASTPIKESELTHRSPSVT**^ +2**
SSAGAHDLGRIQLTIRYSVQRQRLIVVVHQI**^ +2**
ANIPLKDPSNIPDPYVKLYLLPERAKDTKRKTHVVKDNCNPIFDESFEYILSQGELNTKQ
LEVTVASQKQLFYSSSNILGM**^ 0**
VIIDFEKLNVSQPYNAWFDLTPESDRNHR

ATGAGTGTGAGTAAAGACGGGAGTCAAGCCATGGAGAAACGCTCCGAGGGCTCCAGCATC
TTGAGCGTTATTTTCAGCGCAGTCAAAAAG
phase 0
aajj01000056.1 84082-84171

GCCTCCATTGTCGGCATTGTGTACTTTGCGGGTTACATGCAATGGTCGGTCGCGTGGTTC
ATTGGCCCCATTGTCCTTTTCGTGATCAGAGACCAATGGAAAAAAGCCAGCGACAGGAAA

CGCAACATAGCAAAAGCGGCAGCTTTAGCGAGCGAAAAAGACGTTGTTTTGGCGAGACTT
GATGATCTTCCAGCCTGG
phase 0
aajj01000056.1 84219-84416

GTGTTTTTCCCTGACGTTGAAAGAGCCGAGTGGCTCAACAGA
phase 0
aajj01000056.1 84511-84552

ATTATTAAACAGGTTTGGCCTAACATTAACCACTACACACGCGACTTAATCAGGGACACC
ATCCAACCAATTTTGAAAGAAAGCCTAGAAACGTATAAACTGTCCGGTTTCAAATTTGAA
CGCATTATCCTAGGCACCGTG
phase 0
aajj01000056.1 84603-84743

CCGTTCCGAATCGGGGGTGTTAAAGTATACGATAAAAATGTAGCCAGGAACGAAATTATC
ATGGATTTGGACATTTT
phase +2
aajj01000056.1 84790-84866

TTATGCCGGCGATTGCGACATTACGTTCTACTTGGCCGGGATCAAGGGCGGAATCCGCGA
TTTCCAGCTCCACGGCATGCTCCGCGTTGTGATGAAGCCCCTGATCACCACAATACCGCT
GGTGGGTGGCCTCCAAGTCTTCTTCCTCAACAACCCCGATATAGACTTCGACCTTATCGG
CATCGCCGACTTGCTCGACATGCCCGGCTTGAGCGACATCTTGCGGCGCATCGTGGTGGA
GACCGTGGCCTCAATGATGGTCCTCCCCAATAAATTCCCGATCAAGTTGAGCGACGACGT
GGATGCCATGGAACTAAAAGCCCCGGAACCGGAA
phase 0
aajj01000056.1 84909-85242

GGCGTTTTGCGCGTCCATGTGGTCGAGGCCAAGCACCTGATGAAGAAGGACATAGGAGTG
CTAGGTAAAGGTAAATCCGACCCTTACGCAGTGGTCACTTTAGGTGCCCAAGAGTTCAAA
ACGAAGGTTATCGACAATAGCGTGGACCCGAAGTGGGATTTCTGGTGCGAG
phase 0
aajj01000056.1 85288-85458

TTTAATGTGTTGGAATCGGACGGCCAACAACTTTATATCCATCTGTGGGATAAGGACGAA
ACGTCGGATGATGAGACTTTGGGCAG
phase +2
aajj01000056.1 85624-85709

an alternative to the above
GCGCAGATAAGCTCGTACGAATCGCAATTACTAACGGTGAATGTGTGGGATTGGGATCCT
GGGGTTCCCGGTGCCCAGAATCACGACTACTTGGGAAG
phase +2
aajj01000056.1 85888-85985

GGCCACGATTGAAGTATCCAACATCGTAAAGAAAGGCCAAGACGACTTGTGGGTCACTTT
GGAGCAAGCCAAACACGGCATGGTCCATTTACGTTTGACTTGGCTCACGTTAAGCGACAA
TTACTCCGATTTGAAGGCT
phase 0
aajj01000056.1 86342-86480

GCCCTCGAGGAAACGCAACAATTACGAGTCACATCGATGAGTACGGCCTTGTTAACAATC
TTCCTTGATTCGGCCAAAAACCTTCCG
phase 0
aajj01000056.1 86530-86616

CAAGCCCGCGCGAGCACAAAACCCGACCCTTACGCCGTCCTCAAAGTCGGCAATACGACC
AAAGAGACCAAAGTCTTGGAACGCACAATACACCCAGTTTGGGAGCAAGGCTTCTCCTTC
CTTGTGGCCAACCCCGAATCCGACACACTTTACTTGACAATAATTGATCGGAAGACAACA
AATGAGCTCGGCCAAGTCACTTACAACATCAGTAAACTGGCGAAAAAGACCAAAATGGAG
GTTTACAAAGAACCGTTTTCGTTACTTAAATCCGGACCGGAGAGTAAAGTCATCTGGTCG
ATGCACTTAAGG
phase 0
aajj01000056.1 86667-86978

GTACTTAAAAGGGCCGAAGGAGTCGATGACACTGACTCTGGTGATCTTCCATCTTTGCAA
CGCGAGGATTCTAAAGTATTAACAAGTGACGATGTTCCTCCAACCCCAAACCCAACTCCC
GAAATAAATGAAAGTGCCACTGAACCGATCCCTAATTACGACGAAATGATTAAAGCGACT
GCAGCAACAGTAGCGAGTACTCCAATCAAAGAGAGCGAACTCACTCATCGGAGCCCGAGT
GTCACTTC
phase +2
aajj01000056.1 87029-87276

ATCGGCGGGTGCCCACGACTTGGGTCGTATTCAATTGACAATCAGATATAGCGTCCAACG
CCAGCGTCTCATTGTTGTAGTGCACCAAATCGC
phase +2
aajj01000056.1 87326-87418

AAATATACCCCTGAAAGACCCCTCCAATATCCCCGACCCCTACGTCAAGCTCTACCTCCT
TCCAGAACGGGCTAAGGATACGAAACGCAAAACGCACGTCGTTAAGGACAACTGCAATCC
GATTTTTGATGAAAGTTTTGAATATATCCTCTCCCAAGGCGAACTGAACACTAAACAACT
CGAAGTTACTGTCGCATCGCAAAAACAGCTGTTTTACAGCAGCAGTAACATTTTGGGAATG
phase 0
aajj01000056.1 87467-87707

GTGATTATCGATTTTGAGAAACTTAATGTAAGTCAGCCGTACAACGCTTGGTTTGATCTT
ACGCCGGAGTCTGACCGGAATCACCGCTAACCGTCAATTATTTATCAATCTACTTATACA
AGTGATGTAATACCGTTTTTAACTTTTTTATGTGTTGCTATTTATGCCAGTTATGTATTA
ATTAATAAAGCGATGTTGA
aajj01000056.1 87752-87950

Tribolium *FAM62-2*

MSRDLTASEVSVPLSETILNVETVVKLIKK**^ 0**
LGFFFLIYLLGYYEFSVVLPYALLTFVVLNTKWRKETKNKFRVARSIALGSEKNVVLEIF
QNELPAWIKFPEIEKVEWLNNVFKLIWQQINEYTHDLVPKVLEPAIQGYVSDFKFNKVIL
GNV**^ 0**
PLRVDGVKVYDQEDKRKIVMDLNISYAGDCYVTFHTFRFTGGIEKIQ**^ 0**
FHGTVRVVLTPLISKMPLIGGLQVYFMDEPHIDFDLIKATSILDLPYVRNKIKNTTMNVI
NSMFMYPNVYSINLTEGINMSKLTVFRTE**^ 0**
GILRVHVVEAKNLVNRDLIGKSDPYVVLSCGSIRVETPVVENCLNPKWDFWTNFEIEPNS
ELKIEVWDKDEGSKDDSLG**^ +2**
HAKINVAQVAKIGQSDMVSTQILVTHILQIILANRLAR**^ +1**
ILKFDKSPKYCTFLKIMKETELLSPNLHTALLMIYLESSLNLPKFSKTSPNPYAELEVEN
ETKTTDPEQQTCEPLWETGFTFLLRDPKKAVLNLRIIDAESKNKMGEVSFRVDHLKNEPN
MDLKRHTFFFNKPFSEASVCCSMKLR**^ 0**
VLKNDSLEDEKDDSDAPKPQKKLVKQESNLSMISKKSVTTESEGLASEDNATINSS**^ +2**
FDSEGDTNLGKIKLSLSYSQQRQKLIVEVHE**^ +2**
VDLVRKRAQIYVKLYLQTDKQRNHRKKTK**^ 0**
IAKTKDAVFNESFDYLISNADLNWTNLLVMVKTDKGLLKKSLGRTVISLQACGNLTEPFT
DWFDLRSKHGEHVYHLIK

ATGAGTCGTGACCTCACCGCAAGTGAAGTTTCCGTCCCGTTGTCCGAAACCATTCTGAAC
GTAGAAACCGTCGTCAAGCTAATTAAGAAG
phase 0
aajj01000056.1 88339-88428

CTCGGTTTCTTCTTCCTCATTTATTTACTCGGCTATTATGAATTTTCCGTCGTTTTGCCC
TACGCCCTCCTTACCTTCGTGGTGCTAAACACGAAATGGCGCAAAGAAACGAAAAACAAA
TTTCGGGTGGCTAGAAGCATCGCTTTGGGAAGCGAGAAAAATGTCGTGCTAGAAATCTTC
CAAAATGAGCTTCCCGCCTGGATAAAATTTCCCGAAATTGAGAAAGTCGAATGGCTCAAT
AATGTGTTCAAATTAATTTGGCAACAAATAAACGAGTACACGCATGATTTGGTGCCGAAA
GTCCTCGAGCCGGCCATACAGGGCTACGTTTCGGACTTCAAATTTAATAAAGTGATACTC
GGGAATGTG
phase 0
aajj01000056.1 88533-88901

CCGTTGCGAGTCGATGGAGTCAAAGTATACGATCAGGAGGACAAACGGAAGATTGTGATG
GACTTGAATATTTCGTACGCTGGCGACTGTTATGTCACGTTTCACACGTTCCGATTCACA

GGCGGTATTGAAAAAATCCAG
phase 0
aajj01000056.1 88942-89082

TTCCATGGCACGGTTCGAGTCGTTCTCACACCGCTAATTTCGAAAATGCCCCTCATTGGT
GGCCTGCAAGTCTACTTCATGGACGAGCCCCACATCGACTTTGATTTAATCAAAGCGACT
TCGATTCTAGACCTCCCGTACGTGCGCAACAAAATCAAAAACACCACAATGAATGTCATT
AACTCAATGTTCATGTATCCCAACGTTTACTCAATTAACTTGACCGAAGGAATAAACATG
TCCAAGTTGACCGTTTTCCGCACTGAA
phase 0
aajj01000056.1 89128-89394

GGAATTTTGAGGGTGCACGTAGTCGAGGCTAAAAACTTAGTAAACAGGGATTTAATCGGG
AAATCTGACCCTTACGTAGTGCTCAGCTGCGGCTCAATTCGGGTCGAGACGCCCGTCGTT
GAAAATTGTCTCAACCCGAAGTGGGATTTTTGGACGAATTTTGAAATTGAGCCGAATTCG
GAGTTAAAAATTGAAGTTTGGGACAAGGACGAAGGGTCAAAGGACGATTCATTGGGACA
phase +2
aajj01000056.1 89439-89677

CGCCAAAATCAACGTGGCCCAAGTTGCTAAAATTGGTCAATCGGACATGGTGAGTACCCA
AATTTTGGTAACTCATATATTACAAATAATTCTAGCCAATCGCCTTGCAAGGA
phase +1
aajj01000056.1 89723-89835

TCCTAAAATTCGACAAGTCCCCGAAATATTGTACTTTTTTGAAGATAATGAAAGAAACGG
AACTGCTGAGTCCTAATCTACACACTGCCCTTCTGATGATTTACTTGGAATCGTCCCTAA
ACTTGCCCAAATTTTCCAAAACGAGTCCTAATCCTTACGCTGAACTTGAGGTCGAGAATG
AGACAAAAACGACTGATCCGGAGCAACAAACTTGCGAGCCTTTGTGGGAAACCGGCTTCA
CTTTCTTGCTTCGGGACCCAAAAAAGGCTGTTTTAAATCTAAGAATTATCGACGCTGAGA
GTAAAAATAAAATGGGAGAGGTATCTTTCCGAGTTGATCATTTGAAAAACGAACCTAACA
TGGACCTAAAACGCCACACGTTTTTCTTCAATAAGCCGTTTTCAGAGGCGTCCGTTTGTT
GCTCAATGAAACTACGA
phase 0
aajj01000056.1 89913-90349

GTACTAAAAAATGACAGTCTTGAAGACGAAAAAGATGACAGTGATGCGCCCAAACCACAA
AAAAAATTAGTAAAGCAAGAATCGAACCTTTCAATGATATCTAAAAAAAGTGTGACGACT
GAAAGCGAAGGACTTGCATCGGAAGACAATGCAACAATCAATTCGTCCTT
phase +2
aajj01000056.1 90398-90567

TGATTCTGAAGGTGACACTAATTTGGGAAAAATCAAACTGTCTTTATCCTACAGCCAACA
GAGGCAAAAACTAATCGTAGAAGTGCATGAAGT
phase +2
aajj01000056.1 90611-90703

AGATTTAGTCCGGAAACGCGCCCAAATCTACGTCAAACTTTACCTCCAAACCGACAAACA
ACGCAACCACCGCAAAAAAACAAAA
phase 0
aajj01000056.1 90749-90833

ATCGCCAAAACCAAAGACGCTGTTTTCAACGAATCCTTCGACTATTTAATTTCAAACGCG
GACCTCAACTGGACCAACTTACTCGTAATGGTGAAAACCGACAAAGGCCTGTTGAAGAAA
AGCCTCGGCCGAACCGTCATCTCCCTGCAAGCGTGTGGCAATTTAACCGAACCGTTCACC
GACTGGTTCGACTTGCGCTCAAAACACGGGGAACATGTGTATCATTTGATCAAATAA
aajj01000056.1 90878-91114

Tribolium *FAM62-3*

MTESDNLIPPLEPEIQLGQLNGSAMKKRFFVIKIFYK**^ 0**
AILIFVGYAISYMQWSFILLFVTAGTLIWLEQRDNTHASKIKVKATACSFTKQDLVRRID
EIPSW**^ 0**
VKFPDRERAEWLNQVIAQLWPTVESYIVKLFRTSIQTKIRKKYDSFQFESIDFGPT**^ 0**
PPKIDGIKVYTAAATTDSIIIDFDV**^ +2**

FYDGDCDINFSFSGAEIGGIRDFQ**^ 0**
LSVEVRVVLKPLLPKVPLIGGIQIYFLNTPDINFTLEGLSGIPGLSSFIRSKIEEKITKK
IVFPNKITKRFSKSVAPSELKALEPAGVLRVHVFEAKDLMAKDITGKSDPYVILYVGAQE
RKSNTVNQCLNPKWDYWCE**^ 0**
FVIIDPKAQHLGFKLYDRDNVNEDDFLG**^ +2**
SGEVDIASVLKGQTDQWITLDSAKHGAIHLRFTWLSLSSDLEDLDE**^ 0**
ISRETKLLQVDHISTALLTIYVDTATKLPEAKRLVKPHPYFILTLRDQKEKSRVKKHTND
PCWEQGFVMLVPNPLEDSLHMAILDKPTGSLLTQFSYKISDLMQLPDLEISKKEFILDNE
ESKVVLSLQLRILTNESYKIEDESESDSEGGLSRQSSLE**^ +1**
ETSVKSRGSFRNSPKKILEDVLSKTSVSPQSAKPPIQRTPSV**^ +2**
NEHRLGRLEISLEYNEPRQKLLVTVHRV**^ +2**
SNLPLKDPSDIPDPYVRIKMYSQGHTTGPTYRTK**^ 0**
VVTDNCNPVYEETFEYLFSKSDAYEQTLVATVKSKKFLHNNTMGQ**^ 0**
VEINLKYVNLSERYREWFDLCPKSS

ATGACCGAGAGTGACAATTTAATTCCACCTCTCGAACCTGAAATACAACTCGGGCAACTC
AACGGAAGTGCCATGAAGAAACGGTTCTTCGTCATAAAAATCTTCTACAAG
phase 0
aajj01000056.1 92082-92192

GCAATTTTGATTTTCGTCGGCTATGCCATCTCCTACATGCAATGGTCTTTCATCCTCCTA
TTTGTGACTGCGGGTACTTTAATATGGCTCGAACAGAGAGATAACACCCATGCCAGCAAG
ATAAAAGTGAAAGCTACGGCCTGTTCGTTCACGAAACAAGACTTAGTCCGACGAATCGAC
GAAATACCGTCATGG
phase 0
aajj01000056.1 92239-92433

GTCAAATTCCCCGACAGGGAACGCGCCGAATGGCTGAACCAGGTAATAGCCCAACTGTGG
CCCACTGTCGAAAGCTACATTGTCAAGCTGTTCCGAACCTCAATCCAGACGAAAATTCGG
AAAAAATATGACTCTTTCCAGTTCGAAAGCATCGACTTCGGGCCTACA
phase 0
aajj01000056.1 92483-92650

CCACCCAAAATAGACGGTATTAAAGTATACACAGCCGCCGCAACCACAGACTCGATTATC
ATCGACTTTGATGTTTT
phase +2
aajj01000056.1 92699-92775

CTATGACGGCGACTGTGATATTAATTTCTCCTTCTCGGGGGCCGAAATCGGTGGCATTCG
CGATTTTCAG
phase 0
aajj01000056.1 92822-92891

TTAAGCGTCGAAGTCCGAGTTGTCCTGAAGCCACTCCTGCCCAAAGTCCCCCTAATTGGA
GGAATCCAAATCTATTTCCTCAATACGCCCGATATAAATTTCACTCTGGAAGGTCTATCC
GGCATTCCAGGCCTTAGCTCGTTCATTCGGTCCAAAATCGAGGAAAAAATCACCAAAAAG
ATCGTTTTTCCGAATAAGATCACTAAAAGGTTCTCCAAATCGGTGGCACCGTCTGAACTG
AAGGCCTTAGAACCGGCCGGTGTGCTCCGGGTGCACGTTTTCGAAGCGAAGGATTTGATG
GCAAAGGATATAACCGGAAAATCTGATCCTTATGTTATCCTGTATGTGGGGGCGCAGGAA
CGGAAGAGTAACACTGTGAATCAGTGCCTTAACCCCAAGTGGGATTACTGGTGTGAG
phase 0
aajj01000056.1 92938-93354

TTTGTTATAATTGACCCGAAGGCGCAACACCTGGGATTCAAGCTTTATGATCGGGATAAC
GTCAATGAGGATGATTTTTTGGGAAG
phase +2
aajj01000056.1 93404-93489

TGGCGAAGTTGACATAGCCTCAGTCCTCAAAGGACAAACTGACCAATGGATCACCTTAGA
TAGTGCCAAACATGGGGCAATCCACCTACGTTTCACTTGGCTGTCTCTCTCCTCGGATCT
TGAAGATCTTGATGAG
phase 0
aajj01000056.1 93536-93671

ATTTCAAGAGAGACAAAATTATTACAAGTTGACCACATAAGCACCGCTCTCTTGACCATA

TACGTCGATACGGCCACGAAACTTCCCGAAGCTAAACGACTCGTGAAACCCCATCCTTAC
TTTATCTTAACACTGAGGGACCAAAAAGAGAAAAGTCGGGTCAAAAAACACACAAATGAC
CCGTGTTGGGAGCAAGGATTCGTAATGTTGGTACCGAATCCTTTGGAAGATTCCCTCCAC
ATGGCCATTTTGGACAAACCAACAGGGAGTCTTTTGACGCAATTCTCCTACAAAATCTCC
GATCTGATGCAACTACCAGATTTGGAAATCAGCAAAAAAGAGTTTATTTTAGATAACGAG
GAAAGTAAAGTCGTACTTTCGCTACAATTACGGATACTAACAAATGAAAGTTACAAAATC
GAGGATGAGAGTGAGAGTGACTCTGAGGGTGGATTGTCACGTCAAAGCTCACTAGAAG
phase +1
aajj01000056.1 93719-94196

AAACAAGTGTGAAATCGCGGGGTTCGTTCCGAAACTCCCCCAAAAAAATTCTCGAAGACG
TACTATCCAAGACATCAGTCTCGCCCCAATCGGCTAAGCCACCAATCCAACGCACACCAA
GTGTTAA
phase +2
aajj01000056.1 94244-94370

CGAGCACCGGCTAGGCCGCCTCGAAATAAGTTTAGAGTACAACGAGCCGCGACAAAAACT
CCTAGTGACAGTACACCGAGTATC
phase +2
aajj01000056.1 94423-94506

TAATTTGCCGCTGAAAGACCCCTCCGACATTCCCGACCCTTACGTTAGGATAAAAATGTA
TTCGCAAGGTCACACCACAGGCCCCACCTACCGTACTAAG
phase 0
aajj01000056.1 94552-94651

GTGGTGACGGATAACTGCAACCCCGTCTACGAGGAGACTTTCGAGTACTTATTCTCAAAA
TCGGACGCTTACGAACAGACGCTTGTGGCAACGGTCAAGTCGAAGAAATTCCTCCATAAT
AACACAATGGGACAG
phase 0
aajj01000056.1 94701-94835

GTTGAAATTAATTTGAAATATGTTAATTTGAGCGAGCGGTATCGAGAATGGTTCGATCTG
TGCCCCAAGTCGAGTTAAGTCCGTGTTGTGATCGCGAGTGATTGTCCAAGTCTCTTTTAC
GGACGAATTTTTTAGGGCACAGTTTGCAAATGTGAGGGCGTGTCCCCGAGTGGATTTTTT
CGTGGGTGCGCAAGTTGGACGACTGGCAAAACGCCTTCGAGCAGAATTTACAAATGTATG
GCTTACTCTGTAACAAATAAAGTATTATTTCTCC
aajj01000056.1 94882-95155

Ciona intestinalis *FAM62*

Eukaryota; Metazoa; Chordata; Urochordata; Ascidiacea; Enterogona; Phlebobranchia; Cionidae; Ciona.

MPVESRRTDSGFTDEDNLDG**^ +1**
EFENSNEEVFNFMNPNEKGVDDAPSVSDVSSTTESDGTRPQSVGDRIQTVVKA**^ +2**
RIDNTIALVKLGVTRFFIAVFIWLLGYFNFSILWIVI**^ +1**
GVWLAIAISERMRKQKQLTE**^ 0**
VLKNTTESPTKFIETLKELYRSRDGHLPSW**^ 0**
IYFPDVEKAEWLNKIIQQVWPYLTNYVKKVISDE**^ 0**
VQSSVQNSSSLLSSFSFTDINLGCRAPRVAGVKVYDDSITRRNEVVMDIQI**^ +2**
VYDSECNCGVSVNRLQAGICDLRLRGLLRVEFHPLIEDLPLIGAVSVGFVNDP**^ 0**
FIDFDLTDLANLFDLPGFNSLLRGAISDSVCGMMVLPDKYVIKLCPDIDISRLRFPLPQ**^ 0**
GVIRIHVIEARNLEEKDKKVLGFGGGSDPYVTVQ**^ +1**
VGHRQKFKTAVVTHNLNPVWNEVFDVVVPDVPTTQIQFSLFDDDGALNKSDNLG**^ +2**
MCSIPVKSVFKQGIIDE**^ 0**
WVQLSDVSTGAIHVRLEFYELSDNPKDLKG**^ 0**
ALNHSHMNDKLFSSFLNIYVDGAQNLP**^ 0**
EFNQECYDANPQLKITLPGKEPLKTR**^ 0**
VAMHTNNPVWEENFHVLISHPELDLVTFQ**^ 0**
[IKRKDGIKSTLMQHSDALRKVLLMKTEPS**^ 0]**
MENDHGNQNLGFMKFPLKHLLRAQDMTIEHPFTLKSSGPSSVLNMRLTLR**^ 0**
ILKLKSLSESPEAKFTVHTKPKEPMDNT**^ +1**
ENTSNSPSVDASEPSESGSSFSRSTGPTEF**^ +1**
[VDPTLKSFF**^ +1]**
EGITPSPSGLRSNTSLDRTSSAASEISNVGEISNLRKRLNA**^ +2**
RVQNSNNIETNDNGLGRLELTIRYYNKHLVVVVLRA**^ +2**

ANLIVCDDDEKTSDPYVRVYILPDKRSRKKTKVIKNNLNPVWDQ**^ +2**
RLEFDVSKSEVMHKKLHVSVKNQTGFLSSEKVLMGQVIVDLSKLDLHQPTTE**^ +2**
WYNLQVAT

<u>ATG</u>CCCGTCGAAAGCAGACGGACGGACTCTGGCTTTACAGACGAGGATAACTTAGATGGTG
phase +1
aabs01000069.1 291243-291303

AATTTGAAAACTCAAACGAAGAGGTTTTTAACTTTATGAATCCTAACGAAAAAGGTGTGG
ATGACGCTCCATCAGTGAGTGATGTATCGTCCACCACAGAAAGTGATGGCACAAGACCTC
AATCTGTAGGAGATCGAATACAAACAGTCGTCAAAGCAAG
phase +2
aabs01000069.1 291538-291697

GATTGACAACACAATTGCATTGGTAAAACTGGGGGTCACAAGATTTTTCATTGCAGTGTT
TATTTGGTTGCTTGGATATTTCAATTTCAGCATATTATGGATTGTGATTG
phase +1
aabs01000069.1 292563-292672

GTGTCTGGCTAGCCATAGCCATAAGTGAACGCATGAGAAAGCAAAAACAACTAACTGAG
phase 0
aabs01000069.1 293414-293472

GTGCTTAAAAATACGACAGAAAGTCCGACCAAATTCATTGAAACTCTAAAGGAGCTTTAC
AGATCGAGAGACGGTCATTTACCATCATGG
phase 0
aabs01000069.1 293973-294062

ATTTATTTTCCTGATGTAGAAAAGGCAGAATGGCTCAATAAGATAATACAACAAGTGTGG
CCATACCTTACCAACTATGTAAAGAAAGTTATCTCAGATGAG
phase 0
aabs01000069.1 296805-296906

GTACAGAGTTCAGTCCAGAACTCGTCAAGCCTTCTGTCAAGCTTTAGTTTTACGGACATC
AACCTGGGCTGCAGAGCGCCTCGTGTGGCAGGAGTAAAGGTCTACGATGACTCGATTACT
CGGAGAAATGAAGTTGTCATGGATATACAGATTGT
phase +2
aabs01000069.1 297206-297360

ATATGACAGCGAATGTAACTGTGGGGTGTCAGTGAACAGATTGCAAGCTGGTATATGTGA
TTTAAGGTTACGAGGTCTCCTACGTGTTGAGTTCCACCCTCTTATTGAGGACTTGCCACT
TATTGGAGCTGTTTCTGTAGGATTTGTAAATGACCCA
phase 0
aabs01000069.1 298014-298170

TTCATCGATTTCGATCTCACTGATCTTGCCAACCTCTTTGATCTTCCGGGATTTAACTCC
TTGCTCCGAGGAGCGATCTCTGACAGTGTGTGCGGGATGATGGTCCTCCCGGATAAATAC
GTCATAAAGTTGTGTCCGGACATCGATATAAGTCGACTTAGATTTCCCCTGCCACAG
phase 0
aabs01000069.1 298571-298747

GGCGTGATTAGGATTCACGTGATTGAGGCTCGCAATTTGGAAGAAAAAGACAAAAAAGTT
CTTGGCTTTGGAGGAGGTAGTGACCCCTATGTTACAGTACAAG
phase +1
aabs01000069.1 298887-298989

TGGGTCACCGTCAAAAATTCAAGACAGCCGTCGTCACCCATAATTTGAACCCTGTATGGA
ATGAAGTGTTTGATGTAGTTGTACCTGACGTACCAACAACACAAATTCAATTCTCTTTGT
TTGATGATGATGGCGCCCTAAACAAGTCAGATAACTTGGGAAT
phase +2
aabs01000069.1 303720-303882

GTGTTCTATTCCTGTCAAGTCAGTTTTCAAGCAAGGCATTATAGATGAG
phase 0
aabs01000069.1 304384-304432

TGGGTGCAGCTATCTGATGTCAGCACGGGTGCAATTCATGTTCGTTTGGAATTTTATGAA
CTTTCCGACAATCCTAAAGACTTGAAAGGG
phase 0
aabs01000847.1 (18557-18468)

GCGCTTAACCATTCTCATATGAATGACAAACTATTTTCATCTTTTCTCAATATTTACGTCG
ATGGAGCACAAAACCTACCA
phase 0
aabs01000847.1 (17500-17420)

GAATTTAACCAAGAATGCTATGACGCAAACCCCCAGTTGAAAATTACTTTGCCAGGAAAGG
AACCTCTCAAAACTCGG
phase 0
aabs01000847.1 (16779-16702)

GTTGCCATGCACACCAACAACCCAGTTTGGGAGGAAAACTTCCATGTTTTAATCTCACACC
CTGAGTTAGACCTTGTTACTTTTCAA
phase 0
aabs01000847.1 (16365-16279)

sometimes skipped
ATTAAAAGAAAGGACGGTATCAAGTCAACCCTCATGCAGCACAGTGATGCGCTGCGTAAAG
TCTTACTTATGAAAACAGAACCATCG
phase 0
aabs01000847.1 (16365-16279)

ATGGAAAACGATCACGGGAACCAGAATCTTGGTTTCATGAAATTCCCGCTGAAACATCTTC
TCCGAGCACAAGACATGACGATCGAACATCCGTTTACTCTCAAATCATCAGGTCCCTCATC
TGTTCTTAATATGCGACTCACGCTTCGG
phase 0
aabs01000847.1 (14293-14144)

ATTTTAAAGTTAAAATCACTGTCAGAGTCACCAGAAGCAAAATTCACCGTCCACACAAAAC
CAAAAGAACCTATGGATAATACAG
phase +1
aabs01000847.1 (13726-13642)

AAAACACAAGCAACTCACCATCTGTGGATGCATCAGAGCCAAGTGAATCTGGAAGCTCATTT
TCACGATCTACAGGACCCACTGAGTTTG
phase +1
aabs01000847.1 (13109-13020)

sometimes skipped
TTGACCCCACACTCAAGTCGTTTTTTG
phase +1
aabs01000847.1 (12660-12634)

AGGGGATTACCCCTTCACCCTCGGGGTTGCGGTCGAACACGTCGCTTGATAGGACATCTAGT
GCTGCCTCGGAGATATCTAATGTTGGGGAAATATCGAACCTGAGAAAACGACTCAACGCAAG
phase +2
aabs01000847.1 (11373-11250)

GGTACAGAACAGCAACAATATTGAAACAAATGACAATGGATTAGGAAGACTGGAGTTGACCA
TTCGTTATTATAATAAACATCTTGTGGTCGTTGTTTTAAGGGCAGC
phase +2
aabs01000847.1 (10956-10849)

GAATTTAATTGTTTGTGATGATGATGAGAAAACATCGGACCCTTACGTTCGAGTTTACATACT
CCCTGATAAGCGATCGCGAAAGAAAACTAAGGTCATCAAAAACAACCTAAACCCAGTATGGGA
CCAAAG
phase +2
aabs01000847.1 (10435-10304)

ACTAGAATTTGATGTTTCAAAAAGTGAAGTGATGCACAAAAAACTTCACGTCTCCGTGAAAAA
TCAAACTGGATTTCTGTCATCAGAGAAAGTATTAATGGGCCAAGTGATTGTTGACCTTTCAAA
ACTTGATTTGCACCAGCCTACCACTGAATG
phase +2
aabs01000847.1 (9961-9806)

GTATAATCTTCAGGTTGCAACTTGA
aabs01000847.1 (8751-8727)

Strongylocentrotus purpuratus *FAM62*

Eukaryota; Metazoa; Echinodermata; Eleutherozoa; Echinozoa; Echinoidea; Euechinoidea; Echinacea; Echinoida; Strongylocentrotidae; Strongylocentrotus.

MVPGNSETVGDETTTEPVRNIKVEESTDLLSSIRKHGFILGILLMVWLVGYLGFSVLWLL
LIVVVSVWRDRASRRKARSTALARAAVENERDSIVGVVRDLPSW**^ 0**
VYFPDIERAEWLNQ**^ 0**
IVKHLWPYLEGYVEDLLRTSVEPAVQDNLPSYLKSFRFEKIRLGRY**^ 0**
SPRIGGVKAYTEHVGRDEMILDLEI**^ +2**
FYAGDCDIEISVKTVKRLKAGIQDLQ**^ 0**
LHGTLRVEMRPLVNKMPLIGGMSIYFLNRP**^ 0**
AIDFNLTNLADLLDVPGL**^ +2**
SNMLHGILEDQFACFLVLPNRIPLTFMDTTDINELKYPMPK**^ 0**
GVLRITAVEARNLVRADMGLLKKGKSDPYLIIN**^ +1**
VGMQKFKTKTINNNLNPKWNQTFE**^ 0**
ALVYEEHGQTLDVDCWDEDPGSKDDPLG**^ +2**
NLSIDIHYISKMGTFDS**^ 0**
WLPLEDIKHGDLHLHLEWLVPSENFDIIHD**^ 0**
QQVADCIQVSSPTSESLHSCALLVVKLDSAKDLP**^ 0**
VSSRSTSMPSPVCTLKVGQTMQKSH**^ 0**
VQQKTMRPVWEETYHFLVMNPAMQSLDIE**^ 0**
VTDSKKGNKTMGNVSVPLKELLLSQPDMVIERPFKLSNSGPQSNITLKMCLR**^ 0**
ALEKGQAREQHGPFEPMAAL**^ 0**
AKNQLLEEEEDEGVVNDNIDGQMEKSLEKSSDAKSDLPAAEVTINGPEEEDGQSSTPPPN
GTSPRVGDGENSGVELRKRNVHPYVY**^ +2**
[ENDAETSESLKIPENG**^ +2]**
DPKAPFPYGRVQMTIRYSSPRGKVIVVIHKA**^ +2**
SQLTIPDSEDMPDSYIRAYLLPDKSKSGKQKTKVIKDTRDPVFDH**^ +2**
TFEFSCTSTELSERVLDICIKNSHSFLPLNNPTIGQ**^ 0**
VDIDLATLDLSKATTE**^ +2**
WYNLKRISPDSVSRLSMSFTS

ATGGTACCTGGAAATTCAGAAACGGTGGGAGATGAAACAACCACTGAACCTGTCCGGAAT
ATCAAGGTGGAAGAGAGCACCGATTTGCTGTCATCGATCAGAAAACATGGCTTCATTTTG
GGAATTTTGTTGATGGTTTGGCTTGTCGGATACCTCGGCTTCAGTGTATTATGGCTGCTG
CTCATTGTTGTCGTCAGTGTGTGGAGAGATCGCGCCAGCCGCCGCAAAGCCCGGTCGACC
GCACTGGCCAGGGCAGCCGTGGAGAATGAGCGAGATAGCATTGTCGGTGTAGTTCGGGAT
TTGCCGTCATGG
phase 0
ac177115.1 (108349-108038)

GTATACTTTCCTGACATCGAGAGGGCAGAATGGCTCAACCAG
phase 0
ac177115.1 (105934-105893)

ATTGTGAAGCACCTTTGGCCGTACCTGGAGGGGTATGTGGAGGATCTCTTGAGAACATCC
GTAGAGCCCGCCGTCCAGGATAATCTACCATCCTACCTCAAGTCATTCCGCTTTGAGAAG
ATCAGACTTGGAAGATAT
phase 0
ac180489.1 (18226-18089)

TCACCTCGTATTGGAGGAGTTAAGGCCTATACGGAGCATGTCGGGAGAGATGAAATGATT
CTGGATCTGGAGATATT
phase +2
ac180489.1 (13764-13688)

CTATGCAGGTGACTGTGACATTGAGATCAGTGTGAAGACAGTCAAGAGATTGAAAGCAG

GAATCCAAGATCTACAG
phase 0
ac177115.1 (1816-1741)

CTCCATGGTACCTTGAGAGTAGAGATGAGGCCATTGGTTAACAAGATGCCCTTGATTGGT
GGGATGTCAATCTACTTTTTGAACAGACCG
phase 0
aagj01062566.1 779-868

GCCATAGACTTCAATCTTACCAACTTGGCAGACCTATTGGATGTACCAGGTCTAAG
phase +2
ac180489.1 (109499-109444)

CAACATGCTTCATGGTATTTTAGAGGATCAGTTTGCCTGTTTCCTGGTGCTACCCAATAG
AATTCCCCTCACTTTCATGGACACGACTGATATCAATGAACTCAAGTACCCAATGCCTAAG
phase 0
ac180489.1 (108702-108582)

GGAGTGTTGCGTATCACAGCAGTAGAAGCAAGGAATCTGGTCAGAGCTGACATGGGTCTG
CTCAAGAAGGGCAAATCTGACCCCTATCTCATCATCAATG
phase +1
ac180489.1 (107722-107623)

TTGGAATGCAGAAATTCAAGACCAAAACGATCAACAACAATCTCAATCCTAAGTGGAATC
AGACTTTTGAG
phase 0
ac180489.1 (107098-107028)

GCCCTAGTGTATGAGGAGCATGGGCAGACTTTAGATGTGGATTGTTGGGATGAGGATCCA
GGGAGCAAAGATGACCCTCTAGGAAA
phase +2
ac180489.1 (106530-106445)

TTTGAGCATTGACATCCACTACATCTCTAAGATGGGAACGTTCGATTCT
phase 0
ac180489.1 (105895-105847)

TGGTTGCCCCTTGAAGACATCAAGCATGGTGATCTCCATCTTCATTTGGAATGGCTTGTT
CCTTCAGAGAACTTTGATATCATCCATGAT
phase 0
ac180489.1 (105495-105406)

CAGCAAGTAGCCGACTGCATACAGGTATCCTCTCCAACCTCTGAGTCACTTCATTCTT
GTGCCCTACTGGTGGTCAAGCTGGACTCTGCCAAGGATCTACCG
phase 0
ac180489.1 (105019-104918)

GTATCATCAAGGTCTACCAGTATGCCTAGTCCTGTATGTACGCTGAAGGTTGGTCAGACT
ATGCAGAAGAGTCAT
phase 0
ac180489.1 (101472-101398)

GTTCAACAGAAGACAATGCGCCCTGTCTGGGAAGAGACGTATCACTTCCTTGTGATGAAT
CCTGCCATGCAGTCCCTTGATATAGAG
phase 0
ac180489.1 (100926-100840)

GTGACAGACAGCAAGAAGGGAAACAAAACTATGGGTAATGTCTCTGTCCCTCTGAAAGAG
CTGCTACTGAGCCAACCAGACATGGTGATCGAGAGACCATTTAAGCTGAGTAACTCTGGC
CCACAGAGTAATATCACACTCAAGATGTGTCTCAGG
phase 0
ac180489.1 (3642-3487)

GCTCTGGAAAAAGGACAAGCAAGGGAACAACATGGTCCTTTTGAGCCTATGGCTGCTCTG
phase 0

ac180489.1 (3085-3026)

GCAAAGAACCAGCTACTGGAAGAAGAAGAAGACGAGGGTGTTGTCAATGACAACATTGAC
GGTCAGATGGAGAAATCCCTGGAGAAGTCATCCGATGCTAAATCAGATCTTCCAGCGGCC
GAAGTGACCATCAACGGTCCCGAGGAAGAAGACGGTCAGAGCAGCACCCCACCCCCTAAT
GGGACCTCCCCTAGGGTGGGGGATGGAGAGAACAGCGGGGTGGAACTGAGGAAGAGGAAT
GTGCATCCATATGTATATGA
phase +2
ac177115.1 76494-76753

possible skipping indicated by Paracentrotus est am197569
[GAATGATGCAGAAACTTCAGAAAGTCTCAAAATCCCTGAGAATGGAGA]
phase +2
ac177115.1 77959-78006

TCCCAAGGCTCCGTTCCCATATGGTCGTGTCCAGATGACAATTAGATACAGTTCACCGAG
AGGGAAGGTCATAGTCGTCATTCACAAGGCATC
phase +2
ac177115.1 79634-79726

GCAACTGACGATCCCTGACTCTGAAGACATGCCTGATTCTTACATCAGGGCATACCTTCT
ACCAGACAAGTCTAAAAGTGGTAAACAGAAGACCAAAGTCATCAAGGACACAAGAGATCC
TGTCTTTGATCACAC
phase +2
ac177115.1 80718-80852

GTTTGAGTTTTCCTGCACGTCGACAGAGTTATCCGAGAGAGTTCTAGATATCTGCATCAA
GAATTCACACTCTTTCCTTCCCCTCAACAACCCGACCATTGGCCAG
phase 0
ac180489.1 113787-113892

GTTGACATCGACCTTGCCACCCTAGACCTCAGCAAGGCTACAACAGAATG
phase +2
ac180489.1 114486-114535

GTATAATCTGAAGAGGATTAGCCCAGACTCTGTCTCTCGGCTTTCTATGAGCTTCACATC
ATGA
ac180489.1 115339-115402

Danio rerio *FAM62* genes

Eukaryota; Metazoa; Chordata; Craniata; Vertebrata; Euteleostomi; Actinopterygii; Neopterygii; Teleostei; Ostariophysi; Cypriniformes; Cyprinidae; Danio.

FAM62A1

MQKSPMSVDDAGPNAGAAPEAADAAPTTDSAGKHAVSVLWSFGKCVGAFLPVYLAGYFGF
SISVVLLGLLVYIGWKHSRDGKKARLQSAMYFLENEQDVTTTRVFRSKRDLPAW**^ 0**
VNFPDVEKVEWINK**^ 0**
ILQQAWPFVGQYLEKLLVETIAPSIRATSAHLQTLSFTKVDLGDR**^ 0**
AMKVVGVKAYTEFDRRQVILDLYI**^ +2**
SYAGDVEINVEVKKYFCKAGVKGIQ**^ 0**
LHGKLRVILEPLIGDVPLVGAITMFFIRRP**^ 0**
KLDINWTGMTNLLDIPGL**^ +2**
NAMSDTMIMDAIASFLVLPNRLTVPLVANLHVAQLRSPLPR**^ 0**
GIVRIHLLEAENLPAKDNYMKGVISGKSDPYAVLRVGTQIFTSHHVDNNLNPQWREMYE**^ 0**
VIVHEVPGQELELEVFDKDPDQDDFLG**^ +2**
RMKLDLGIVKKAVLLDE**^ 0**
WYTLKDAASGQVHLRLEWLSLLPSAERLSE**^ 0**
VLERNQNITVPSKTADPPSAAVLTVYLDRAQDLP**^ 0**
FKKGNKDPSPMVQISVQDTTKESR**^ 0**
TVYGTNNPAWEDAFTFFIQDPRKQDIDIQ**^ 0**

VKDDDRALTLGSLYIPMSRLLSSPELTMDQWFQLEKSGPASRIYITAMLR**^ 0**
VLWLNEDAILTSPVSPIPGEGYGETEVSSGATKVTATPKRPEHTSPDSNFASE**^ 0**
GVLRIHLVEAQSLVAKDNLMGGMMKGKSDPYVKIRVGGLAFKSQVIKENLNPVWNELYE**^ 0**
VILTQLPGQEVEFDLFDKDIDQDDFLG**^ +2**
RVKVSLRDLISAQFTDQ**^ 0**
WYTLNDVKTGRIHLVLEWVPKISDPIRLEQ**^ 0**
ILQYNYRQSYLNKIVPSAALLFVYIERAHGLP**^ 0**
LKKSGKEPKAGAEVSLKNVSYRTK**^ 0**
VVNRSTSPQWDEALHFLIHNPTEDTLIVK**^ 0**
VSHSWGQALGSLVLPVRELLEEKDLTIDRWFSLNGAMPESQILLRAELK**^ 0**
LLDSKLAQCSDEEDASHVIPSAEPTVAANSELRHRNVPVQ**^ +2**
GGEDASSTGKAQIKLSISYSSEEHRLIIKVHAC**^ +2**
RNLPSSSKEPPDSYISFILLPDKNRNTKKKTSVKKKSLKPEFNE**^ +2**
IFEFDMSLEEAKQKHLEVSVKNSVSFMSREKELLGK**^ 0**
LQIDFSPLDLKTGVSQ**^ +2**
WYDLSHETN

ATGCAGAAATCTCCGATGTCAGTTGATGATGCAGGGCCGAATGCGGGTGCCGCTCCGGAGG
CTGCGGACGCGGCCCCGACTACCGATTCCGCTGGAAAACACGCCGTGTCCGTTCTCTGGTC
CTTTGGAAAGTGCGTCGGCGCCTTCCTGCCGGTCTACCTGGCTGGTTATTTCGGCTTCAGT
ATCAGTGTAGTTCTGCTCGGGCTCCTGGTTTATATAGGATGGAAACACAGTCGCGATGGGA
AAAAAGCGCGACTGCAGAGCGCGATGTATTTTTTGGAGAACGAACAGGATGTCACAACTAC
ACGAGTGTTCAGAAGTAAACGGGATTTGCCTGCATGG
phase 0
cr457457.15 (111477-111136)

GTGAACTTCCCTGACGTGGAAAAGGTTGAGTGGATCAACAAG
phase 0
cr457457.15 (107666-107625)

ATATTGCAGCAGGCCTGGCCGTTTGTTGGACAGTATCTGGAAAAGCTGCTGGTGGAAACTA
TCGCTCCTTCAATCCGAGCTACCAGCGCTCATCTGCAGACTCTAAGCTTCACTAAAGTTGA
CTTGGGTGACAGG
phase 0
cr457457.15 (104463-104329)

GCTATGAAGGTAGTTGGTGTGAAGGCCTACACTGAGTTCGATAGGCGTCAGGTTATACTGG
ATCTGTACATCAG
phase +2
cr457457.15 (104189-104116)

TTATGCTGGAGATGTGGAGATTAATGTGGAGGTGAAGAAGTACTTCTGTAAAGCTGGAGTT
AAAGGCATTCAG
phase 0
cr457457.15 (101914-101842)

CTGCATGGAAAGTTGAGAGTGATCTTAGAGCCTCTAATTGGGGATGTTCCTCTCGTGGGAG
CCATCACCATGTTCTTCATTCGCAGGCCT
phase 0
cr457457.15 (101768-101679)

AAACTCGACATCAACTGGACTGGCATGACCAATTTATTAGACATACCGGGTTTGAA
phase +2
cr457457.15 (101586-101531)

TGCCATGTCGGACACTATGATAATGGATGCAATCGCCTCTTTCCTGGTTCTCCCCAATCGT
CTCACTGTGCCTTTGGTGGCCAATCTGCACGTAGCACAGCTGCGTTCCCCTCTTCCACGG
phase 0
cr457457.15 (101194-101074)

GGTATAGTTCGTATCCACTTGCTGGAGGCAGAAAACCTTCCCGCCAAGGATAACTACATGA
AAGGTGTGATATCAGGGAAATCTGACCCATATGCGGTGTTGCGTGTGGGGACGCAAATCTT
CACCTCCCATCACGTGGACAACAACCTTAATCCTCAGTGGAGAGAGATGTACGAG
phase 0
cr457457.15 (92286-92110)

GTGATTGTCCATGAAGTGCCTGGTCAGGAGCTAGAGCTTGAGGTATTTGATAAAGACCCTG
ACCAAGATGACTTTCTTGGAAG
phase +2
cr457457.15 (91770-91688)

AATGAAGTTGGATTTAGGCATTGTAAAGAAAGCAGTTCTTTTGGATGAG
phase 0
cr457457.15 (91588-91540)

TGGTACACTTTAAAGGATGCTGCATCGGGTCAGGTTCATCTGAGGTTAGAATGGCTCTCGC
TGCTGCCCTCTGCAGAACGTCTCAGCGAA
phase 0
cr457457.15 (89490-89401)

GTGCTGGAGAGAAATCAGAACATTACAGTGCCTAGCAAGACAGCAGATCCTCCTTCTGCTG
CCGTATTGACTGTGTATCTTGACCGTGCACAAGATCTACCT
phase 0
cr457457.15 (89322-89221)

TTCAAGAAAGGTAACAAGGACCCCAGCCCTATGGTGCAGATCTCTGTCCAGGACACCACCA
AAGAAAGCAGG
phase 0
cr457457.15 (89135-89064)

ACAGTGTATGGGACTAATAATCCAGCATGGGAGGATGCCTTCACGTTTTTTATTCAGGATC
CCCGCAAACAAGATATCGACATTCAG
phase 0
cr457457.15 (88970-88884)

GTGAAGGATGATGACAGGGCTTTGACTCTGGGAAGCCTATATATCCCAATGTCTCGTCTGC
TGTCCAGCCCTGAGCTTACCATGGATCAGTGGTTTCAGCTAGAAAAATCCGGACCAGCCAG
TCGTATCTACATCACAGCTATGCTGAGG
phase 0
cr457457.15 (87143-86994)

GTGTTGTGGCTGAATGAAGACGCCATCCTTACCTCTCCTGTATCTCCAATACCTGGAGAAG
GATACGGCGAAACTGAAGTGTCTTCAGGAGCCACAAAAGTGACCGCCACTCCAAAACGCCC
TGAGCACACTAGTCCAGACAGCAATTTTGCATCTGAG
phase 0
cr457457.15 (86923-86765)

GGTGTTCTGCGGATCCATCTGGTGGAGGCACAGAGTCTCGTCGCTAAGGACAATTTAATGG
GAGGAATGATGAAGGGAAAGAGTGACCCATATGTGAAGATCAGAGTGGGAGGATTGGCCTT
TAAGAGTCAGGTCATTAAAGAGAACCTCAACCCTGTCTGGAATGAGCTTTATGAG
phase 0
cr457457.15 (84320-84144)

GTGATCTTGACCCAGCTCCCTGGTCAGGAGGTAGAGTTTGACCTGTTTGACAAAGACATTG
ACCAGGATGACTTTCTTGGCAG
phase +2
cr457457.15 (83975-83893)

GGTTAAAGTGAGTCTGAGAGACCTCATCAGTGCTCAGTTTACTGATCAG
phase 0
cr457457.15 (80914-80866)

TGGTATACTCTGAATGATGTGAAGACCGGAAGAATCCATCTGGTGTTAGAATGGGTGCCTA
AAATTTCAGATCCCATCAGACTTGAACAG
phase 0
cr457457.15 (80741-80652)

ATCCTACAGTACAACTACAGGCAGTCTTACCTGAATAAAATAGTTCCCTCTGCTGCACTGC
TGTTTGTTTATATTGAGAGAGCACATGGGCTGCCT
phase 0
cr457457.15 (80554-80459)

TTAAAGAAGAGTGGAAAAGAGCCTAAAGCTGGAGCAGAAGTGTCACTCAAAAATGTATCTT
ACAGGACCAAA
phase 0
cr457457.15 (80381-80310)

GTTGTGAATCGCTCCACTTCTCCACAATGGGACGAAGCCCTTCATTTCCTGATTCATAATC
CAACAGAAGACACACTTATTGTCAAA
phase 0
cr457457.15 (80227-80141)

GTCAGTCACAGCTGGGGTCAGGCTCTTGGTTCACTTGTTCTTCCTGTACGAGAGCTGCTGG
AGGAGAAGGATCTTACGATAGACCGCTGGTTTAGTCTGAATGGAGCCATGCCTGAAAGTCA
GATTCTGCTGAGAGCCGAACTAAAG
phase 0
cr457457.15 (79720-79574)

CTCCTAGACAGCAAGTTGGCACAATGTAGTGATGAAGAGGACGCTAGTCATGTGATTCCCAG
TGCAGAACCAACTGTTGCAGCCAATAGTGAGCTCCGACACAGAAATGTACCTGTCCAAGG
phase +2
cr457457.15 (78049-77928)

AGGGGAAGATGCCAGTTCAACTGGGAAAGCCCAAATTAAACTATCTATTAGCTATTCATCTG
AAGAGCACCGATTGATTATCAAAGTCCATGCTTGCAG
phase +2
cr457457.15 (77815-77717)

GAACTTGCCGTCCTCCTCTAAGGAACCTCCAGACTCTTACATCTCATTCATTCTTCTTCCG
GACAAGAACCGGAACACCAAAAAGAAGACCAGTGTTAAAAAGAAAAGCCTCAAACCAGAGT
TCAATGAGAT
phase +2
cr457457.15 (76472-76341)

ATTTGAGTTTGACATGTCACTTGAAGAAGCAAAGCAGAAACATCTTGAAGTTTCAGTTAAG
AACAGCGTTTCCTTCATGAGTCGAGAAAAAGAGCTTCTTGGCAAG
phase 0
cr457457.15 (76264-76159)

CTTCAGATAGATTTTAGTCCGCTGGATCTCAAGACTGGAGTGTCACAATG
phase +2
cr457457.15 (73556-73507)

GTATGACTTGTCACACGAGACCAACTGA
cr457457.15 (73427-73400)

FAM62A2

MSHVDSETTNDSQKITEPAQDSSSGEMDVPKTKLFDAKAILWTFGKCLTALLPVYLAGYY
RMSTSLVVFGMMVYAGWKHTREAKEARLRSAIQLVNDEQEYVSSKSFRSKRDLPSW**^ 0**
VNFPDVEKVEWLNK**^ 0**
VIHQAWPFIGQYLEKLLTETIAPAIRGSSAHLQTLSFTKIDFGGK**^ 0**
PMKVVGVKAHTENDKGQILLDVYI**^ +2**
SYVGDVEINVEVKRYFCKAGVKGIQ**^ 0**
LHGMMRVILEPLISDVPIVGAVTMFFIQRP**^ 0**
KLTINWTGLTNLLDIPGL**^ +2**
NVMSDTMIMDAIASFLVLPNRLTVPLVADLPVAQLRCPLPR**^ 0**
GVVRIHLLEADNLAAKDNYVKGVMAGMSDPYAIVRVGPQTFKSHHLDNTLSPKWGEVYE**^ 0**
VVVHEVPGQELEVEVFDKDPDHDDFLG**^ +2**
RTKLDLGIVKKSKIVDE**^ 0**
WFNLKDTQTGRVHLKLEWLTLETHTERLKE**^ 0**
VLKRNESVVSKAAEPPSAAILAVYLDKAEALP**^ 0**
MKKGNKDPNPIVQISVQNATRDSR**^ 0**
ICWNTVNPQWEDAFTFFIRDPNNQDISVQ**^ 0**
VKDNDRVQLLGKMSIPASRLLSHPDLSMDEWYNLENSGPKSRIHINTVLR**^ 0**
VLWLDEAAVTASLLSSGPLSKSSRPEKTTPHSSFATE**^ 0**

GLLRIHLVEGQNLVAKDNLMGGMVKGKSDPYVKIQIGGETFKSHVIKENLNPTWNEMYE**^ 0**
VVLTELPGQELTLEVFDKDMDMKDDFMG**^ +2**
RLKMSLSDIISSQYINE**^ 0**
WFSLSDVKRGRVHLALEWLPTVTKPEKLQQ**^ 0**
VLHFQSKSSFLNKAVPSAALLFVYVEQAYELP**^ 0**
LKKSGKEPKVGAELVLGGTSRKTT**^ 0**
VCDRTSTPKWDEAFYFLVRDPLNEDLIVK**^ 0**
LSHNWDFSVGSVVIPIKELLSEPDLLLDQWLDLDGASPQSQILLRAQLK**^ 0**
ILCPKKMESSEEQHEEPKHHEESSIRRKQQEELMQ**^ +2**
KSSIEEVPPSPVSRTSSVSVPEEEEAPEVTQVSSSDDLRPLHTSPDPSFGTE**^ 0**
GVLRLILLEAQDLVAKDGLMGGMVKGKSDPYVKIHIGDTTFKSHVIKENLNPTWNEMYE**^ 0**
LVLTSSSSSEVLVEVFDKDMDKDDFLG**^ +2**
RMKISLQEIIQSQITDR**^ 0**
WFSLSDVKHGRVHLILEWLNTVTKPDPLQK**^ 0**
AVQLQSDHSYLNKSVPSAALFFILLERAHNLP**^ 0**
LKKSGKEPKAAAELVLGDITHKTK**^ 0**
VCERSMSPQWSEAFHFLVHKPTEEILIIK**^ 0**
LSSAFEQPLGSLVLPIRELLSKTDLLMDQWLSLDGAAADSQILLRAQLK**^ 0**
KQTTHAIEEQHTPSTPKTHMPTE**^ 0**
DLSKAPDSTTNIAEHKESAHKDPQHLDKATE**^ +2**
PSHKSSSPSVPAEETKVTSSTDTRPQKTSHNSNFGTK**^ 0**
GLLRLHLLEAQDLVAKDGLMGGMMKGKSDPYVKIHIGDTTFKSHVIKENLNPTWNEMYE**^ 0**
LILSPDPNLEVKFEVYDKDVDSDDFLG**^ +2**
RFKLRLGDIIKSQYNDE**^ 0**
WFTLNDIKHGRVHLVVEWLPTVTQRDKLEQ**^ 0**
VMQMQSSQSYQNKSVASAALLFILLDRAHQLP**^ 0**
LKKSGKEPKAAAELTLGGTSYKSK**^ 0**
VCERSSSPHWNETFDFLVHDPKKDVLVIK**^ 0**
LSSAWDQPMGSLVLPIRELLLKPDLLLDQWLSLDGASAQSQILLRAQLK**^ 0**
ILDSKMAALVAMGSGPVLSNKQTATTGQIQLSMSFQKKLTLLVHNC**^ +2**
RGLVTSSKDSLDTYVSIILLPDKSKATKRKTSVKKKTLNPEFNE**^ +2**
KFEFDMSMEEVQRRELSVCVKNASSSFMNRDKDVIGQ**^ 0**
VQIDLGHIDLISGVTQ**^ +2**
WFDLKEEQN

ATGTCGCATGTAGATTCAGAGACGACAAATGACTCGCAGAAGATTACCGAACCAGCGCAG
GACAGTTCGTCTGGAGAAATGGACGTCCCGAAGACCAAATTGTTCGACGCCAAGGCGATC
TTGTGGACTTTTGGGAAATGTCTCACCGCCTTGTTGCCCGTGTACCTGGCCGGGTACTAT
CGGATGAGCACCAGTTTGGTGGTGTTTGGGATGATGGTTTACGCAGGATGGAAACACACG
CGTGAGGCGAAGGAGGCGAGACTGAGGTCTGCCATACAGCTAGTAAACGACGAGCAAGAA
TATGTGTCCTCCAAATCATTTAGAAGTAAAAGAGACCTGCCTTCTTGG
phase 0
al929108.5 34397-34744

GTCAACTTCCCAGATGTGGAGAAGGTTGAATGGCTAAACAAG
phase 0
al929108.5 35951-35992

GTTATCCACCAGGCCTGGCCATTTATTGGTCAGTATTTGGAGAAGCTGCTGACGGAGACC
ATTGCTCCGGCCATCCGTGGCTCCAGTGCTCATCTGCAGACCCTCAGCTTCACCAAGATA
GACTTCGGTGGTAAG
phase 0
al929108.5 41266-41400

CCAATGAAAGTGGTCGGCGTGAAGGCTCATACAGAAAACGATAAAGGTCAAATTCTGCTT
GACGTTTACATCAG
phase +2
al929108.5 45402-45475

TTATGTTGGGGATGTGGAGATAAACGTGGAGGTGAAGAGATACTTCTGCAAAGCTGGTGT
GAAGGGAATACAG
phase 0
al929108.5 49665-49737

CTTCATGGAATGATGCGTGTGATTTTGGAGCCGCTGATCAGCGACGTCCCAATAGTAGGA
GCTGTGACCATGTTCTTCATCCAGAGACCT
phase 0
al929108.5 49831-49920

AAACTGACCATTAACTGGACCGGTCTGACCAACCTGCTGGACATTCCAGGACTCAA
phase +2
al929108.5 52714-52769

TGTGATGTCTGACACCATGATCATGGATGCGATTGCTTCATTCCTGGTGTTGCCCAATCG
CCTGACAGTTCCTCTGGTGGCAGACCTGCCTGTGGCACAGCTGCGGTGCCCTTTGCCCAGG
phase 0
al929108.5 52892-53012

GGAGTTGTGCGTATTCACCTGCTGGAGGCTGATAATCTAGCTGCGAAGGATAATTATGTA
AAGGGTGTGATGGCCGGCATGTCTGACCCGTACGCCATTGTCCGAGTCGGACCACAAACC
TTCAAATCCCATCACCTGGACAACACGCTCAGTCCCAAATGGGGTGAAGTGTATGAG
phase 0
al929108.5 56023-56199

GTTGTCGTTCATGAGGTGCCTGGTCAGGAGCTAGAGGTGGAAGTGTTTGATAAAGATCCC
GATCATGATGATTTCCTGGGCAG
phase +2
al929108.5 56607-56689

GACTAAATTAGACTTGGGGATTGTGAAGAAATCTAAAATAGTTGATGAG
phase 0
al929108.5 58679-58727

TGGTTCAACCTGAAGGACACTCAAACAGGACGAGTTCACCTCAAACTGGAGTGGCTGACG
CTTGAAACTCACACAGAAAGACTGAAAGAG
phase 0
al929108.5 60093-60182

GTTTTGAAGCGAAATGAAAGTGTGGTGAGTAAAGCTGCAGAGCCGCCCTCAGCCGCCATC
TTGGCAGTGTATTTGGACAAGGCTGAGGCACTTCCT
phase 0
al929108.5 60475-60570

ATGAAGAAAGGCAATAAGGATCCCAATCCGATAGTTCAAATCTCAGTGCAAAATGCTACT
CGTGACAGCCGG
phase 0
al929108.5 65998-66069

ATTTGCTGGAATACTGTAAATCCCCAGTGGGAGGACGCTTTTACTTTCTTCATCAGAGAT
CCAAACAATCAGGACATTAGTGTACAG
phase 0
al929108.5 66163-66249

GTGAAGGATAATGACCGTGTTCAGCTTTTGGGGAAAATGTCTATTCCTGCATCCCGTCTC
CTCTCTCATCCCGATCTGTCTATGGATGAATGGTATAATCTGGAGAATTCTGGCCCAAAG
AGTCGCATTCACATCAACACAGTGCTCAGG
phase 0
al929108.5 77541-77690

GTTCTCTGGTTGGATGAAGCCGCTGTAACTGCGTCGCTCCTCTCTAGTGGACCTCTTTCA
AAGAGCTCACGGCCAGAGAAAACCACTCCCCACTCCAGCTTCGCCACCGAG
phase 0
al929108.5 78127-78237

GGGCTGCTGCGGATCCACTTGGTCGAGGGTCAGAATCTGGTGGCTAAAGACAATCTGATG
GGTGGGATGGTGAAGGGGAAAAGCGACCCATATGTCAAGATCCAAATCGGAGGTGAAACC
TTCAAAAGTCACGTGATCAAGGAGAACCTCAACCCCACCTGGAATGAGATGTATGAG
phase 0
al929108.5 78394-78570

GTGGTTTTGACGGAGCTTCCGGGTCAAGAGCTCACGTTAGAAGTTTTTGATAAGGACATG
GATATGAAAGATGACTTTATGGGCAG
phase +2

al929108.5 82709-82794

GCTGAAGATGAGTCTGAGTGACATCATCAGCTCTCAGTACATTAATGAG
phase 0
al929108.5 82881-82929

TGGTTTTCTCTGAGCGATGTAAAGCGTGGTCGTGTTCACCTCGCTCTGGAGTGGCTTCCC
ACTGTAACTAAACCAGAGAAACTACAGCAG
phase 0
al929108.5 83580-83669

GTGCTGCATTTCCAGTCAAAGAGCTCATTTCTGAATAAGGCAGTGCCGTCTGCAGCTCTC
CTGTTTGTGTATGTGGAGCAAGCATATGAGCTTCCT
phase 0
al929108.5 83751-83846

CTCAAGAAGAGCGGGAAGGAGCCTAAAGTTGGAGCTGAGCTGGTCTTAGGAGGAACATCT
CGAAAAACAACG
phase 0
al929108.5 84890-84961

GTGTGTGATCGCACCAGCACTCCTAAATGGGATGAAGCGTTTTACTTTCTGGTTCGAGAC
CCTTTGAATGAAGACCTTATTGTGAAG
phase 0
al929108.5 86371-86457

TTGTCCCATAACTGGGATTTTTCCGTGGGGTCTGTGGTGATTCCTATCAAAGAGCTTCTC
TCTGAGCCGGATCTGCTGCTGGACCAGTGGCTGGATCTGGACGGAGCTTCACCTCAGAGT
CAGATTCTGCTCAGAGCTCAGCTGAAA
phase 0
al929108.5 86545-86691

ATTCTTTGCCCTAAAAAGATGGAGAGCTCAGAGGAGCAGCATGAGGAGCCCAAACATCAT
GAAGAGTCCAGCATCAGAAGAAAACAACAAGAGGAGCTCATGCAGAA
phase +2
al929108.5 87800-87906

GTCTAGTATTGAGGAGGTTCCTCCATCTCCCGTCAGCAGGACGTCCTCAGTTTCTGTCCC
AGAAGAAGAGGAGGCACCAGAGGTCACACAAGTCTCCAGTTCAGATGACCTTCGACCCCT
GCACACCAGCCCTGACCCCAGCTTTGGTACAGAG
phase 0
al929108.5 88309-88462

GGTGTTCTGCGGCTCATCCTGCTGGAGGCTCAAGATCTGGTGGCGAAGGATGGTCTGATG
GGTGGGATGGTGAAGGGGAAAAGTGACCCGTATGTTAAGATCCACATCGGTGACACAACA
TTTAAGAGTCACGTAATCAAGGAGAACCTCAACCCCACCTGGAATGAGATGTATGAG
phase 0
al929108.5 89276-89452

CTGGTTCTGACCTCCAGCTCCTCTTCTGAAGTGCTCGTAGAGGTTTTCGACAAGGATATG
GACAAAGATGACTTTTTAGGGAG
phase +2
al929108.5 89536-89618

GATGAAGATCAGTCTACAGGAGATCATTCAGTCTCAGATCACTGACAGA
phase 0
al929108.5 90284-90332

TGGTTTTCTCTGAGTGACGTCAAACATGGTCGTGTTCATTTGATTCTGGAGTGGCTGAAC
ACAGTGACAAAACCTGACCCACTGCAAAAG
phase 0
al929108.5 90410-90499

GCTGTGCAGTTACAGTCAGATCATTCATACCTGAACAAAAGTGTGCCATCGGCTGCTCTG

TTCTTCATCCTCCTGGAAAGAGCTCATAATCTGCCT
phase 0
al929108.5 90993-91088

CTGAAGAAGAGTGGAAAAGAGCCTAAAGCTGCTGCAGAGCTGGTGTTAGGAGACATCACT
CACAAAACTAAG
phase 0
al929108.5 91198-91269

GTGTGTGAGAGATCCATGTCTCCTCAGTGGAGCGAAGCTTTTCACTTCCTGGTTCACAAA
CCTACTGAGGAGATTCTTATTATCAAG
phase 0
al929108.5 91349-91435

CTTTCCAGTGCATTCGAGCAGCCGCTGGGCTCTCTGGTTCTGCCAATCAGAGAGCTGCTT
TCTAAAACAGACCTGCTGATGGACCAATGGCTGAGTCTGGATGGAGCAGCTGCTGACAGT
CAGATTCTTCTCAGAGCTCAACTCAAG
phase 0
al929108.5 93094-93240

AAACAAACAACTCACGCCATTGAGGAGCAACACACACCTTCGACACCTAAAACACACATG
CCGACTGAG
phase 0
al929108.5 95087-95155

GATCTCAGTAAAGCGCCTGACTCCACTACTAATATTGCTGAACATAAGGAATCAGCCCAT
AAAGATCCACAACACCTGGATAAAGCAACAGAACC
phase +2
al929108.5 95812-95906

GTCTCACAAGAGTAGTTCTCCCAGTGTTCCTGCTGAGGAGACTAAAGTTACCAGTTCTAC
AGACACTCGACCCCAAAAGACGAGCCACAACTCCAACTTTGGCACCAAG
phase 0
al929108.5 96020-96128

GGGCTGCTGCGGCTCCACCTGCTGGAGGCTCAGGATCTGGTGGCGAAGGATGGTCTGATG
GGTGGGATGATGAAGGGGAAAAGTGACCCCTATGTCAAGATCCACATCGGAGACACAACA
TTTAAGAGTCACGTGATCAAGGAAAACCTCAACCCCACCTGGAATGAGATGTATGAG
phase 0
al929108.5 97534-97710

CTGATTCTGAGTCCTGATCCTAATCTAGAGGTGAAGTTCGAGGTCTATGATAAAGATGTT
GATTCTGATGATTTTCTTGGAAG
phase +2
al929108.5 98063-98145

ATTTAAGCTCAGACTTGGAGACATCATCAAATCTCAGTACAATGATGAG
phase 0
al929108.5 98638-98686

TGGTTTACATTGAATGATATCAAACATGGCCGTGTGCATCTGGTTGTGGAGTGGCTGCCC
ACCGTCACCCAGCGGGACAAACTGGAGCAG
phase 0
al929108.5 98915-99004

GTTATGCAGATGCAGAGCTCGCAGTCCTACCAGAACAAGAGTGTGGCTTCAGCAGCGCTG
CTCTTCATTCTGTTAGACAGAGCTCATCAGCTGCCC
phase 0
al929108.5 99092-99187

CTGAAGAAAAGTGGAAAGGAACCCAAAGCTGCAGCTGAACTTACACTTGGAGGAACGTCT
TATAAATCCAAG
phase 0 gc donor
al929108.5 102915-102986

GTGTGTGAACGTTCATCTTCTCCTCACTGGAATGAAACTTTTGACTTTCTGGTTCACGAC
CCCAAAAAGGACGTGCTGGTGATCAAG
phase 0
al929108.5 103081-103167

CTCTCGAGTGCCTGGGATCAACCAATGGGCTCTTTGGTTCTGCCAATCAGAGAGCTGCTT
TTAAAACCAGATCTTCTGCTGGACCAATGGCTGAGCCTGGATGGAGCTTCAGCACAGAGT
CAGATTCTGCTCAGAGCTCAGCTGAAG
phase 0
al929108.5 103558-103704

ATCTTGGATTCCAAAATGGCGGCTTTAGTGGCGATGGGTTCAGGTCCGGTTTTGAGTAAC
AAGCAAACGGCCACAACAGGACAAATCCAGCTCTCCATGTCCTTCCAGAAGAAGCTGACG
CTGCTCGTCCACAACTGCAG
phase +2
al929108.5 104858-104997

GGGTCTGGTCACATCATCTAAGGACAGCTTAGACACTTACGTCTCCATCATTCTGCTGCC
AGATAAAAGCAAGGCCACCAAGAGAAAGACCAGTGTGAAGAAGAAAACCCTCAATCCAGA
GTTCAATGAAAA
phase +2
al929108.5 105177-105308

GTTTGAGTTTGATATGAGTATGGAAGAGGTTCAGAGACGAGAGCTCAGTGTGTGTGTGAA
AAACGCTTCCTCGTCCTTCATGAACCGAGACAAAGACGTGATCGGCCAG
phase 0
al929108.5 106443-106551

GTGCAAATAGACCTGGGACACATTGATTTAATCTCTGGAGTCACACAATG
phase +2
al929108.5 107304-107353

GTTTGATCTCAAAGAAGAGCAGAACTGA
al929108.5 108105-108132

FAM62B

MSASVNGVEPKPAPAVSQNGPGSPPQTPKDVTPPELELPDEEPQSSVTEATQMGIKFAKT
FLLIFPIYLLGYLEFSFSWVLIGLGLVFWLKRNQGSRFARVNQAMAFLEQEERAVRQTIR
SSELPPW**^ 0**
VHFPDVERVEWLNK**^ 0**
TVQQMWPYICQFVEKIFKETIEPAVQGANTHLSTFTFSKIDMGDK**^ 0**
PLRVDGVKVYTENVDKRQIIMDLQI**^ +2**
NFVGNTEIDVDIKKYYCRAGIKSIQ**^ 0**
LNGVLRVIMEPLLGDMPLVGALSVFFLKKP**^ 0**
FLDINWTGLTNMLDIPGV**^ +2**
NSLCDNVIQDIINGCLVNPNKITIPLADDALISKLRFPMPR**^ 0**
GILRVHFLEGQDLLSKDTYMGGLIKGKSDPYGVIQINNQLFRSKIIKDSLNPRWNEVYE**^ 0**
AIVYDGQGQVVFIELFDEDTDHDDFLG**^ +2**
SLTMEIDEIQKQQKVDE**^ 0**
WFDLIGVPNGKLHVKAEWLSLHPTPDKLDE**^ 0**
VLSSIKADKGQANDGLSSALLLVHLDSAKNLP**^ 0**
RNPLEFNSAGLKKGAVNKAVK**^ 0**
SGKKVTSVPNPFVQFTVGHRSFESK**^ 0**
TRFKTIEPVWEETFTFLIHNPKCQDLEVE**^ 0**
VKDEKHECSLGTITLPLSQLLKEKQMTMSQRFPLKNSGPGSTLKMKMALR**^ 0**
ILSLDKLAASDKPSSAQVHRAGSVRKTSNATPQRPAVSEPAKTSKTQQPAPAPRTQPTPT
PSPRVEPVTDRKPLEESPPHLAKSGKSMSNLAISGSNLHLNSKEQTPSIASDISNLAATQ
ELQKTIQHLH**^ +2**
NGASPGFAPLGEIELTIRHSPQRNKLIVVVHKC**^ +2**
RNLISASQNGSDPYVRLYLLPDKRRSGRRKTSTAKKTVNPVFDQ**^ +2**
TFEFTVSIVELQKRTLDVAVKNGGGILAKHRGLLGK**^ 0**
VIVEFNLEDPSKSSTQ**^ +2**
WYELSVDGFKRPSP

<u>ATG</u>AGTGCGTCAGTGAACGGAGTGGAGCCCAAACCCGCTCCAGCGGTCAGCCAGAACGGACCCGGGTCTCCT
CCTCAGACCCCCAAAGATGTCACACCACCGGAGCTTGAACTACCGGACGAGGAGCCGCAGTCTTCGGTCACG
GAAGCCACGCAGATGGGGATTAAGTTCGCAAAGACGTTCCTACTGATTTTCCCTATATACTTGCTGGGATAT
CTGGAGTTCAGTTTCAGCTGGGTTTTGATCGGCCTGGGATTGGTGTTTTGGCTGAAACGGAATCAGGGCAGC
AGGTTCGCCCGGGTGAACCAGGCGATGGCGTTTCTGGAGCAGGAGGAGCGCGCGGTGCGGCAGACCATCCGG
AGCTCAGAGCTGCCGCCGTGG
phase 0
bx005044.9 17406-17786

GTTCATTTTCCAGATGTGGAGAGAGTGGAGTGGCTCAATAAG
phase 0
bx005044.9 41332-41373

ACTGTGCAGCAGATGTGGCCGTATATTTGTCAGTTTGTAGAGAAAATCTTCAAAGAGACCATTGAACCAGCT
GTGCAGGGAGCAAACACGCACCTTAGTACTTTCACCTTCTCCAAGATAGATATGGGGGACAAG
phase 0
bx005044.9 41534-41668

CCTCTCAGGGTGGATGGAGTGAAAGTTTATACAGAGAATGTCGATAAACGACAGATTATTATGGACCTACAG
ATCAA
phase +2
bx005044.9 42105-42181

TTTTGTTGGAAACACTGAAATTGATGTGGACATCAAGAAATACTACTGCCGTGCAGGAATCAAAAGTATTCAG
phase 0
bx005044.9 43702-43774

CTGAATGGGGTGTTGAGGGTCATCATGGAGCCGCTTTTAGGAGACATGCCTCTGGTCGGCGCACTGTCTGTG
TTCTTCCTCAAGAAACCT
phase 0
bx005044.9 47200-47289

TTTCTGGACATTAATTGGACGGGTCTCACAAATATGCTGGACATTCCTGGAGTAAA
phase +2
bx005044.9 49962-50017

TTCTCTGTGTGACAATGTTATTCAGGACATCATAAACGGTTGCCTTGTGAACCCAAACAAAATCACCATCCC
GCTGGCAGATGATGCTCTCATTAGCAAACTTCGCTTCCCAATGCCGAGG
phase 0
bx005044.9 52770-52890

GGCATTCTGCGAGTTCACTTTCTGGAGGGACAGGACCTGTTATCGAAGGACACGTATATGGGAGGTTTGATT
AAAGGAAAGTCAGACCCGTATGGAGTCATTCAAATCAACAATCAGCTCTTCAGATCCAAAATCATCAAAGAT
TCCTTAAACCCTAGGTGGAATGAAGTATACGAG
phase 0
bx005044.9 52974-53150

GCGATTGTGTATGATGGTCAAGGACAGGTTGTGTTCATAGAGCTGTTTGATGAGGATACGGATCATGATGAT
TTTCTGGGAAG
phase +2
bx005044.9 53226-53308

CCTCACCATGGAAATAGATGAGATACAGAAGCAACAGAAAGTTGATGAG
phase 0
bx005044.9 54817-54865

TGGTTTGATCTTATCGGTGTTCCTAACGGAAAGCTCCACGTGAAGGCTGAATGGCTTTCTCTTCACCCAACA
CCAGATAAACTTGATGAG
phase 0
bx005044.9 61766-61855

GTTTTAAGCAGCATTAAAGCAGATAAAGGACAGGCCAACGATGGACTGTCATCAGCATTACTGCTAGTGCAC
CTGGACTCAGCCAAAAATCTGCCA
phase 0
bx005044.9 63024-63119

CGCAATCCTTTAGAATTCAACAGTGCAGGTCTGAAGAAGGGTGCAGTCAATAAAGCCGTAAAG
phase 0
bx005044.9 70097-70159

TCTGGAAAAAAAGGTCACCAGTGTGCCCAATCCATTCGTCCAGTTCACTGTCGGACACAGATCGTTTGAAAGC
AAG
phase 0
bx005044.9 72105-72179

ACAAGATTTAAAACAATTGAGCCGGTTTGGGAGGAGACCTTCACATTCCTCATCCACAATCCAAAGTGTCAA
GACCTCGAAGTCGAG
phase 0
bx005044.9 74922-75008

GTAAAGGATGAGAAACACGAGTGTTCTCTGGGGACGATCACTCTTCCTCTGTCTCAGCTGCTGAAGGAGAAA
CAGATGACGATGAGTCAGAGATTCCCACTCAAGAACTCAGGACCCGGTTCTACACTTAAAATGAAGATGGCA
CTTAGG
phase 0
bx005044.9 75796-75945

ATTCTGTCTCTGGATAAATTGGCAGCATCAGATAAACCGTCCTCAGCTCAGGTCCACAGGGCCGGCTCTGTG
AGGAAGACATCCAACGCTACTCCTCAGCGGCCTGCAGTTTCAGAGCCTGCAAAAACATCCAAAACCCAGCAG
CCCGCACCTGCACCCCGAACCCAGCCCACCCCAACTCCCTCTCCACGGGTGGAACCCGTAACAGACCGCAAA
CCGCTGGAGGAATCACCCCCACATCTGGCTAAATCAGGCAAGAGCATGTCTAACCTGGCCATTTCTGGCTCC
AATCTCCACCTGAACAGCAAAGAGCAAACGCCCAGCATCGCCTCAGACATCTCCAATCTCGCCGCCACACAG
GAGCTCCAGAAGACCATACAGCATTTACATAA
phase +2
bx005044.9 77300-77691

CGGCGCTTCACCGGGTTTCGCTCCTCTGGGTGAAATTGAGCTAACCATCAGACACAGTCCCCAAAGAAATAA
GCTGATTGTCGTGGTACACAAATGCAG
phase +2
bx005044.9 81696-81794

AAATCTGATCTCTGCTTCTCAAAATGGTTCCGATCCATACGTGCGTTTGTATTTACTACCTGACAAGCGGCG
CTCTGGACGCAGAAAAACAAGCACAGCGAAGAAAACAGTGAACCCCGTCTTTGATCAAAC
phase +2
bx005044.9 84262-84393

GTTTGAGTTCACAGTGTCCATCGTGGAGTTGCAGAAGAGAACTCTAGATGTGGCCGTGAAGAATGGAGGAGG
AATACTGGCAAAACACAGAGGGCTGCTAGGAAAG
phase 0
bx005044.9 84523-84628

GTGATAGTTGAATTTAACCTCGAGGACCCATCAAAGAGCTCAACGCAATG
phase +2
bx005044.9 86981-87030

GTATGAGCTTTCAGTGGACGGATTTAAGCGACCATCACCGTAA
bx005044.9 87175-87217

FAM62C

MSNGVNEPPLSTEQPEGLKPKDVSQMILEFLMFMMRAIAICYPVYLTGTFGLSVSWILLS
MFMWTMWKNNRRWKEQRIDTAIDFLENEKDVISTELKAMDMPPW**^ 0**
IHFADVEKAAWINK**^ 0**
ILQQSWPFFGVYMEKLLIENIQTVVRSVHPHLKTFTFTKVHMGQK**^ 0**
APTITGIRAYTDELETREVILDLNI**^ +2**
VYEADVDIDADVNRAIKVGIKGLQ**^ 0**
LQGMLRVILEPLIGQAPLVGGVTMFFIRRP**^ 0**
ALQINWTGVTNVLDGPGL**^ +2**
SHLSESAIVDVIASLMVLPNRMCFPLIDQVKVEQMRFPLPR**^ 0**
GVVRVHVLEARDLVAKDSHMMGLVKGKSDPYTVLRVGNKHFKTKTIKETLNPRWNEVYE**^ 0**
FVIHEAPGQELEVELYDEDKDADDFLG**^ +2**

RFSMDCGDVRKDREIDK**^ 0**
WYTLEDIESGQIHFKLQWFSLCSNPELLKE**^ 0**
TSDGLACAMLALYLDCASNLP**^ 0**
KDQREVTHNEKHGKQPKESR**^ 0**
VTRKTNNPNSYVEFSIDLQSQKSK**^ 0**
VVFASKDPIFDECFTFFVHSVKNQVLNVE**^ 0**
VKEHEKKSSLGKFSLPLVRLLNVSDMTLDQRFQLERSAPNSQVKLKAVLR**^ 0**
ILTLEKQQPKVVTSAPQDKNTSTPNRPEPRTPNPTSNPVPNPAPPPAAAQLVQSNPKEKG
PISSVPLSKTQVPFSVVPLNDLQAEYPPYRRSTFVGSEGLQSTPSTPGPMRRYDSHSLLS
ENSIASSRVDLTDSYPYP**^ +2**
EAIMNHQGTFGQIQITLRYATLRKRLIVIVNCC**^ +2**
NNLFSSNESGSDTYVRMYLLPDQTWKHRKRTAVKKKTVNPVFDE**^ +2**
TFEFAVSLEEARNRKLDVAVKNNKMLHKRERKEIGM**^ 0**
VLIDMSEIDLTKGSTE**^ +2**
WYELTLPGLKKTNWQS

ATGAGTAACGGGGTCAACGAGCCCCCTCTCTCCACCGAACAACCGGAGGGACTCAAGCCGAAG
GATGTCAGTCAGATGATCCTGGAGTTCCTGATGTTCATGATGAGGGCGATAGCCATCTGCTAC
CCCGTGTATTTGACGGGCACATTTGGACTAAGCGTCAGCTGGATTCTTCTGAGCATGTTTATG
TGGACCATGTGGAAAAACAACCGCAGGTGGAAAGAGCAGAGAATCGACACAGCCATTGACTTT
TTAGAAAATGAGAAGGACGTGATCAGTACTGAACTCAAAGCCATGGACATGCCACCTTGG
phase 0
cr533429.6 (97900-97589)

ATTCATTTTGCAGATGTTGAGAAGGCTGCGTGGATCAACAAG
phase 0
cr533429.6 (96474-96433)

ATTCTTCAGCAGTCATGGCCGTTCTTCGGAGTGTACATGGAGAAACTACTCATTGAAAACATC
CAGACAGTGGTCAGATCAGTCCACCCTCATCTAAAGACTTTCACCTTTACTAAAGTTCACATG
GGACAGAAG
phase 0
cr533429.6 (95807-95673)

GCTCCAACTATTACTGGCATTCGGGCGTATACAGATGAGCTGGAAACAAGAGAAGTCATTCTT
GATCTCAACATTGT
phase +2
cr533429.6 (95563-95487)

TTACGAGGCTGATGTGGACATTGATGCTGATGTAAACCGAGCCATTAAAGTAGGAATCAAAGG
GCTTCAG
phase 0
cr533429.6 (91677-91608)

CTTCAGGGGATGCTGCGTGTAATTCTAGAACCTCTGATTGGCCAGGCACCCCTTGTAGGTGGA
GTCACTATGTTTTTCATTCGTCGACCT
phase 0
cr533429.6 (90079-89990)

GCTCTGCAAATCAATTGGACTGGAGTGACAAACGTTTTGGATGGTCCAGGTTTAAG
phase +2
cr533429.6 (89258-89203)

TCATTTGTCAGAATCAGCCATTGTGGACGTCATCGCCTCTCTCATGGTTCTGCCCAATCGCAT
GTGCTTCCCTTTAATTGATCAAGTCAAGGTTGAACAAATGAGGTTTCCTCTTCCTCGA
phase 0
cr533429.6 (86343-86223)

GGTGTTGTACGCGTCCATGTTCTGGAAGCCCGCGATCTTGTAGCAAAGGACTCACACATGATGG
GACTAGTAAAGGGCAAGTCAGATCCATATACTGTGCTGAGAGTTGGAAACAAGCATTTCAAAAC
AAAGACCATCAAAGAAACTCTGAATCCACGCTGGAATGAAGTTTATGAG
phase 0
cr533429.6 (86134-85958)

TTTGTCATTCATGAAGCCCCGGGGCAGGAGTTGGAGGTGGAACTCTACGATGAAGACAAAGACG
CTGATGACTTTTTGGGCAG

phase +2
cr533429.6 (85863-85781)

GTTCAGTATGGATTGTGGGGATGTGAGAAAAGACCGAGAGATAGATAAG
phase 0
cr533429.6 (85696-85648)

TGGTACACTCTGGAGGACATTGAGAGTGGTCAGATACATTTTAAACTGCAGTGGTTTTCTTTGT
GCTCAAACCCAGAACTGCTGAAGGAG
phase 0
cr533429.6 (85553-85464)

ACCAGTGATGGGCTTGCTTGTGCTATGCTGGCATTGTATCTAGACTGTGCATCTAATCTGCCT
phase 0
cr533429.6 (84029-83967)

AAAGATCAACGTGAAGTCACCCATAATGAAAAACATGGAAAACAACCTAAAGAGTCTCGG
phase 0
cr533429.6 (83848-83789)

GTCACTAGAAAAACAAATAATCCCAACTCCTATGTGGAGTTTTCCATTGATCTGCAAAGTCAG
AAAAGCAAG
phase 0
cr533429.6 (83004-82933)

GTTGTGTTTGCCTCAAAAGACCCGATTTTTGATGAATGCTTCACATTCTTTGTGCACAGTGTT
AAAAACCAAGTGTTAAATGTTGAG
phase 0
cr533429.6 (82821-82735)

GTAAAGGAACATGAGAAGAAATCCTCACTGGGCAAATTCAGTCTGCCGTTGGTGCGGCTGCTC
AATGTTTCTGACATGACTTTGGACCAGCGCTTTCAGCTGGAGCGCTCCGCACCCAATAGCCAG
GTCAAGTTGAAAGCTGTTCTTAGG
phase 0
cr533429.6 (81175-81026)

ATTCTTACATTAGAGAAACAACAACCAAAGGTTGTCACTTCAGCACCTCAGGATAAAAATACA
TCAACCCCAAATCGTCCTGAGCCACGTACTCCAAATCCAACTTCAAATCCAGTTCCAAATCCA
GCTCCACCTCCGGCTGCAGCTCAGCTAGTACAGAGCAACCCAAAAGAAAAAGGGCCCATTTCA
TCAGTCCCGCTCTCTAAAACCCAAGTTCCATTTTCAGTTGTGCCCCTTAAATGATCTTCAAGCA
GAGTATCCTCCCTACAGACGAAGCACTTTTGTGGGCTCAGAGGGGCTGCAATCGACCCCTTCC
ACCCCCGGTCCAATGCGACGCTATGATTCCCACAGCCTTTTGTCTGAGAACTCGATTGCTTCT
TCACGCGTTGATCTCACAGACAGTTACCCTTACCCAGA
phase +2
cr533429.6 (79939-79524)

GGCCATCATGAATCATCAGGGCACCTTTGGACAGATCCAGATTACATTGCGTTATGCCACCTT
AAGAAAGCGGCTTATTGTGATAGTCAACTGCTGCAA
phase +2
cr533429.6 (76063-75965)

CAATCTGTTTTCCTCCAATGAGAGTGGCTCTGATACTTACGTTCGCATGTATCTGCTGCCTGA
CCAGACGTGGAAACACCGAAAGCGCACCGCTGTTAAAAAGAAGACTGTGAATCCTGTCTTTGA
TGAAAC
phase +2
cr533429.6 (75881-75750)

TTTTGAGTTTGCAGTGTCACTGGAGGAAGCAAGAAACAGAAAGTTGGACGTTGCTGTAAAAAA
CAATAAAATGCTTCATAAGAGAGAGCGAAAAGAGATTGGAATG
phase 0
cr533429.6 (75658-75553)

GTTTTGATTGATATGTCTGAAATAGATCTGACGAAGGGCTCAACAGAATG
phase +2
cr533429.6 (75409-75360)

GTATGAACTCACTCTCCCGGGGCTAAAGAAGACAAACTGGCAAAGCTGA
cr533429.6 (75216-75168)

***FAM62* fragment**

xGFSDQMIQDIISAYMVLPNRITVPLIGEVELAQLRFPMPK**^ 0**
GVLRIYFIEAQNLEVKDTYLGGLIKGKSDPYGMLLVSNQLFRSKTIKECLHPKWNEVYE**^ 0**
ALVYEPSGQHLEIELFDEDPDKDDFLG**^ +2**
SLMIDLTELHKEQKVDE**^ 0**
WFDLEEVTTGKLHLRLEWLSLYSSAEKLDQ**^ 0**
VQKSIRTNDNLSSALLIVNLDSASNLP**^ 0**

Danio ests bm860829 and al920225 give in addition, roughly

SGKKVSIDPNPFVKLTVGQKTCTSK
VRYKTSEPLWEETFPFLIKNPQTQ...

TGGCTTTTCAGATCAAATGATCCAGGATATTATCAGCGCTTACATGGTGTTGCCTAACAGG
ATCACAGTGCCGTTGATAGGTGAGGTTGAGCTGGCACAGCTTCGCTTCCCCATGCCAAAG
phase 0
caak03018817 (2836-2716)

GGAGTGCTGAGGATTTATTTTATTGAAGCTCAGAATCTGGAAGTGAAGGACACGTATCTTG
GCGGACTAATCAAAGGCAAGTCTGACCCATATGGTATGCTGCTGGTCAGCAACCAACTTTT
CAGAAGTAAGACAATCAAAGAGTGTCTGCACCCCAAGTGGAACGAGGTGTATGAG
phase 0
caak03018817 (2591-2415)

GCACTAGTATATGAACCTTCAGGACAACATCTGGAAATTGAGCTCTTTGATGAAGACCCAG
ATAAAGATGATTTCTTGGGAAG
phase +2
caak03018817 (2335-2253)

TCTAATGATTGACTTGACAGAACTTCATAAAGAACAGAAGGTTGATGAG
phase 0
caak03018817 (2160-2112)

TGGTTTGATTTGGAAGAAGTTACTACAGGCAAACTTCACCTGAGATTAGAGTGGCTGTCAC
TGTACTCGAGTGCTGAAAAACTAGATCAA
phase 0
caak03018817 (1071-982)

GTACAGAAGAGCATAAGGACTAATGACAATCTCTCTTCCGCTCTGCTTATTGTTAACCTGG
ATTCAGCCAGTAATTTGCCA
phase 0
caak03018817 (875-796 and 391-311)

Gallus gallus *FAM62* genes

Eukaryota; Metazoa; Chordata; Craniata; Vertebrata; Euteleostomi; Archosauria; Aves; Neognathae; Galliformes; Phasianidae; Phasianinae; Gallus.

FAM62B

MSGAAEKQSPGAGTPAAAEKAAAGGDAGPEPPPPSMLSVDVTGLVSQFARSFVLIFPVYV
LGYLGLSFSWVLIALCGLFWIRRHRGGKTSRLGRALAFLEDEEEAVRLSVSSADLPAW**^ 0**
VHFPDTERAEWLNK**^ 0**
TVKQMWPFICQFIEKLFRETIEPAVRGANNHLSTFSFTKIDIGHQ**^ 0**
PLRINGVKVYTENVDKRQIILDLQI**^ +2**
SFAGNCEIDLEIKRYFCRAGVKSIQ**^ 0**
IHGTMRVILEPLIGDMPLIGALSLFFLRKP**^ 0**
LLEINWTGLTNLLDVPGL**^ +2**

NGLSDTIILDIISNYLVLPNRITVPLVSEVQIAQLRFPIPK**^ 0**
GVLRIHFIEAQDLEGKDTYLKGIVKGKSDPYGIIRVGNQIFQSKVIKENLNPKWNEVYE**^ 0**
ALVYEHPGQELEIELFDEDPDKDDFLG**^ +2**
SLMIDLIEVEKERLLDE**^ 0**
WFTLDEVSKGKLHLKLEWLTLMPTAENLDK**^ 0**
VLTSIRADKDQANDGLSSALLILYLDSARNLP**^ 0**
HNPLEFNPDALKKSAVQKALK**^ 0**
SGKKLNSNPNPLVLLSVGHKAQESK**^ 0**
IRYKTNEPVWEENFTFFVHNPKRQDLEVE**^ 0**
VRDEQHQCSLGNFKLPLSQLLESEDLTMHQRFQLSNSGPNSTINMKIALR**^ 0**
VLSLEKQARSPDHQHSAQVKRPSVSKDARKSSFKPQVPVSPPLDSSKHAPASPVADSDKK
TDVAEKSQPPNASPQWPTDLSRSSSSLHASNFNYSPSHLSVKEPTPSIASDISLPIATQE
LRQRLRQLE**^ +2**
NGTTLGQSPLGQIQLTIRHSSQRNKLIVVVHSC**^ +2**
RNLIAFSEEGSDPYVRMYLLPDKRRSGRRKTHVSKKTLNPVFDQ**^ +2**
IFDFSVSLPEVQRRTLDVAVKNSGGFLSKDKGLLGK**^ 0**
VLIPLTSEELAKGWTQ**^ +2**
WYDLTEDGTRPHGAS

exon1 from est aj729155
ATGAGCGGGGCCGCGGAGAAGCAGTCCCCCGGCGCGGGGACTCCCGCCGCGGCGGAGAAA
GCGGCGGCCGGGGGGGACGCGGGGCCGGAGCCGCCGCCGCCTTCGATGCTGAGCGTGGAT
GTGACGGGCTTGGTGTCGCAGTTCGCCAGGAGCTTCGTGCTGATCTTCCCCGTGTACGTG
CTGGGCTACCTGGGGCTGAGCTTCAGCTGGGTGCTCATCGCCCTGTGCGGGCTCTTCTGG
ATCCGGCGGCACCGCGGCGGCAAGACCTCCCGCCTGGGCCGGGCGCTCGCCTTCCTGGAG
GACGAGGAGGAGGCGGTGCGGCTCAGCGTCTCCTCCGCCGATCTGCCTGCGTGG
phase 0

GTCCATTTTCCAGATACGGAAAGAGCAGAATGGCTCAACAAG
phase 0
aadn02000371.1 (104276-104235)

ACTGTAAAACAAATGTGGCCTTTTATTTGCCAATTTATTGAGAAACTCTTTCGGGAGACC
ATAGAACCAGCAGTAAGAGGAGCAAACAACCACCTCAGTACCTTCAGTTTTACGAAGATT
GATATTGGTCATCAG
phase 0
aadn02000371.1 (103523-103389)

CCTCTGAGGATAAATGGTGTAAAAGTGTACACTGAAAATGTTGACAAAAGGCAGATCATT
TTGGATCTTCAGATCAG
phase +2
aadn02000371.1 (102249-102173)

TTTTGCTGGAAATTGTGAAATTGATTTGGAGATCAAGAGATACTTCTGTAGAGCTGGTGT
AAAAAGTATACAG
phase 0
aadn02000371.1 (98499-98427)

ATCCATGGTACTATGAGGGTTATCCTGGAACCACTAATTGGAGACATGCCCCTGATTGGA
GCACTATCACTTTTTTTCCTTAGGAAACCT
phase 0
aadn02000371.1 (97552-97463)

CTTTTAGAAATAAACTGGACTGGGCTGACCAATCTTCTGGATGTTCCAGGGCTGAA
phase +2
aadn02000371.1 (85696-85641)

TGGCTTATCAGATACGATAATATTGGATATAATATCCAACTACCTGGTCCTGCCAAACAG
AATTACAGTCCCTCTTGTCAGCGAAGTGCAGATTGCTCAGCTGCGGTTTCCTATACCAAAG
phase 0
aadn02000371.1 (80751-80631)

GGTGTTTTAAGGATACATTTTATTGAAGCTCAGGACTTGGAGGGTAAAGATACTTATCTG
AAAGGGATTGTCAAAGGCAAGTCAGATCCCTATGGAATCATTCGTGTGGGCAACCAGATT
TTCCAAAGCAAGGTCATCAAAGAAAATCTCAATCCAAAATGGAATGAAGTTTATGAG
phase 0

aadn02000371.1 (80006-79830)

GCCTTAGTATATGAACATCCAGGACAGGAGCTAGAGATTGAGCTCTTTGATGAAGATCCA
GACAAAGATGACTTCCTTGGAAG
phase +2
aadn02000371.1 (79460-79378)

TTTGATGATAGATTTAATTGAGGTTGAAAAGGAGCGACTTTTAGATGAG
phase 0
aadn02000371.1 (78030-77982)

TGGTTTACTTTGGATGAGGTGTCCAAAGGAAAATTGCATTTAAAACTGGAATGGCTCACA
TTGATGCCAACAGCAGAAAACCTAGACAAG
phase 0
aadn02000371.1 (77353-77264)

GTATTAACAAGCATTAGAGCTGATAAAGACCAAGCCAATGATGGGCTGTCTTCTGCATTG
CTTATTCTCTATTTGGACTCAGCAAGAAACCTGCCT
phase 0
aadn02000371.1 (76763-76668)

CATAATCCATTAGAATTTAACCCTGATGCCTTGAAGAAGTCTGCTGTTCAGAAAGCCCTAAAG
phase 0
aadn02000371.1 (70044-69982)

TCAGGAAAGAAACTAAACAGCAATCCCAACCCACTTGTTTTGCTGTCGGTTGGACATAAG
GCCCAGGAGAGTAAG
phase 0
aadn02000371.1 (68023-67949)

ATTCGATACAAGACCAATGAACCAGTATGGGAGGAAAACTTCACTTTCTTTGTACACAAT
CCCAAAAGACAGGATCTTGAAGTTGAG
phase 0
aadn02000371.1 (66188-66102)

GTGAGGGATGAACAGCACCAGTGTTCTCTAGGGAACTTCAAATTGCCTCTAAGCCAGTTG
CTGGAGAGTGAAGATTTAACGATGCATCAGCGGTTCCAGTTGAGCAACTCCGGTCCAAAC
AGCACTATAAACATGAAGATTGCACTCAGG
phase 0
aadn02000371.1 (65406-65257)

GTCCTTTCTCTTGAAAAGCAAGCAAGATCTCCTGATCATCAACACTCAGCACAAGTAAAA
AGGCCGTCTGTTTCCAAAGATGCAAGAAAATCATCTTTTAAGCCTCAGGTTCCTGTCTCG
CCACCGCTTGATTCAAGCAAACACGCTCCAGCTTCCCCAGTGGCGGACAGTGATAAAAAG
ACTGACGTGGCTGAAAAAAGTCAGCCTCCTAATGCGAGTCCACAGTGGCCAACAGATCTC
AGCCGAAGTTCCTCCAGTCTTCATGCCTCTAACTTCAATTATTCTCCCAGCCACCTGTCA
GTCAAAGAACCAACTCCTAGCATAGCCTCAGACATATCTCTGCCCATTGCCACACAGGAG
CTCCGGCAAAGACTACGGCAACTTGAAAA
phase +2
aadn02000371.1 (64999-64611)

CGGAACAACTCTGGGGCAGTCTCCATTGGGACAAATACAGCTAACAATTCGGCATAGTTC
ACAAAGAAACAAATTGATTGTGGTGGTGCATTCCTGCAG
phase +2
aadn02000371.1 (64320-64222)

AAATCTAATAGCATTTTCAGAAGAAGGATCTGATCCATACGTTCGAATGTATTTATTGCC
CGATAAGAGAAGATCGGGAAGAAGAAAAACACATGTATCAAAGAAGACATTAAACCCGGT
GTTTGATCAAAT
phase +2
aadn02000371.1 (63520-63389)

ATTTGATTTCAGTGTTTCCCTGCCTGAAGTACAGAGAAGAACACTAGATGTAGCAGTGAA
GAACAGCGGTGGTTTCTTGTCCAAAGATAAAGGGCTGCTTGGCAAA
phase 0

aadn02000371.1 (62494-62389)

GTACTGATACCTCTGACATCTGAAGAACTTGCTAAAGGCTGGACCCAGTG
phase +2
aadn02000371.1 (61368-61319)

GTATGATTTAACAGAAGATGGCACAAGACCGCATGGTGCGAGCTAG
aadn02000371.1 (61208-61163)

FAM62C

MDPAASRPAAPQRALLSSAGRALLWLGPVYLAGYLGLSGSWLLLGLALALGWARNRRGKR
ERLAAASVLLEDEREAVRRGLAARHLPAW**^ 0**
VHFPDVERVEWLNK**^ 0**
VLEQAWPYFGTIMEKTFKEVLEPKIRAKSVHLKTCTFTKIQFGEK**^ 0**
CPRINGVKVYTKEIDRRQVILDLQI**^ +2**
CYVGDCEIHMDISKFNLGVKGVQ**^ 0**
LYGTLRVILEPLLTDAPFIGAVTLFFMQKP**^ 0**
HLEFNWAGMSNLLDVPGI**^ +2**
NVMSDSLIQDYIAARLVLPNRITVPLKKNMSIAQLRFPVPH**^ 0**
GVIRVHLLEAENLVQKDNFLGAIRGKSDPYALLRLGTVQYRSKTISRDLNPIWNETFE**^ 0**
FVVHEVLGQDLEVDLYDADPDKDDFMG**^ +2**
SLLISLLDIKNDKTVDE**^ 0**
WFPLSKTTSGHLHLKLEWLSLVNDQEKLHE**^ 0**
DKKGLSTAILIVYLDSAFNLP**^ 0**
KNHFEYSNGECGARKIKNNKYLK**^ 0**
KTEREPSSFVLLTVGSKTQKSK**^ 0**
TCNFNKDPKWGQAFTFFVHSAHSQSLHIE**^ 0**
IKDKDQDSSLGTSVVCLSHLLKDPNMTLDQRFQLDHSSSDSFIKIKLVLR**^ 0**
ALNVEEPDPQRVKAGVGATKKGPMHVMEKGGNQQEFVPPPKPEVSKVPPVSKDTAVQESQ
PKKDSSEDLDTSNSSAVPAASETVASLDETESEQDPEHRAPSALHTRAAVVPMLPVVEEL
RLAPSVTSLGSLPSSCFELSSSNLDLH**^ +2**
NGMEMPLGEIQLTVRYASIRQSLVVLVNGC**^ +2**
RNLIPSSNRGVDPYVRIYLLPDRRWTSRKKTSVKKRTLNPQYDE**^ +2**
KFEFFESLEEVKKRTLDVAVKNSRPFISQEKKELGK**^ 0**
VWIDLSQEDLIKGFTQ**^ +2**
WYELTRSRRKKN

ATGGATCCCGCCGCCTCCCGCCCAGCCGCCCCGCAGCGGGCGCTGCTCTCGTCCGCCGGC
CGGGCGCTGCTGTGGCTCGGCCCCGTGTACCTGGCGGGGTACCTGGGGCTGAGCGGCAGC
TGGCTGCTGCTGGGGCTGGCGCTGGCTCTGGGCTGGGCGCGCAACCGGCGGGGGAAGCGC
GAGCGGCTGGCGGCCGCCTCGGTGCTGCTGGAGGACGAGAGGGAAGCGGTGCGGCGGGGC
CTGGCCGCCCGGCACCTGCCGGCATGG
phase 0
aadn02016908 (59680-59414)

GTCCATTTCCCTGATGTTGAGCGAGTGGAGTGGCTGAACAAG
phase 0
aadn02016908 (55314-55273)

GTCCTTGAACAGGCTTGGCCATACTTTGGGACAATCATGGAAAAAACATTTAAAGAAGTT
TTGGAACCAAAAATTAGAGCAAAGAGCGTACATCTGAAGACGTGCACCTTTACCAAGATC
CAGTTTGGAGAGAAG
phase 0
aadn02016908 (54454-54320)

TGCCCTAGAATCAACGGAGTAAAAGTCTACACCAAAGAAATTGATAGAAGACAAGTTATC
CTAGACCTGCAGATATG
phase +2
aadn02016908 (53799-53723)

TTACGTAGGAGACTGCGAGATTCACATGGACATATCGAAGTTTAATCTTGGGGTGAAAGG
TGTGCAG
phase 0
aadn02016908 (48647-48581)

TTGTATGGGACTTTGCGAGTGATACTGGAACCTCTTCTCACTGATGCCCCTTTCATTGGA
GCAGTGACCTTGTTTTTATGCAGAAACCG
phase 0
aadn02016908 (47942-47853)

CACTTGGAATTCAACTGGGCAGGCATGAGCAACCTCCTGGATGTCCCAGGGATTAA
phase +2
aadn02016908 (47043-46988)

TGTAATGTCAGACTCACTGATTCAAGATTACATTGCTGCACGGCTGGTTCTGCCAAACAG
GATCACGGTGCCTCTGAAGAAGAACATGAGCATTGCCCAGTTGAGGTTCCCTGTCCCACAT
phase 0
aadn02016908 (45194-45074)

GGAGTAATAAGGGTTCATCTGCTAGAAGCTGAAAACCTTGTCCAGAAAGATAATTTCCTT
GGTGCCATCAGGGGGAAGTCTGACCCATACGCTCTTCTTCGGCTTGGCACAGTGCAGTAT
CGGAGCAAGACAATTTCCCGAGATCTTAATCCCATCTGGAATGAGACATTTGAG
phase 0
aadn02016908 (44103-43930)

TTTGTTGTTCACGAAGTGCTTGGTCAGGACTTAGAAGTGGACTTGTATGATGCAGATCCA
GATAAAGATGACTTCATGGGCAG
phase +2
aadn02016908 (43386-43304)

CTTGCTTATAAGCCTGCTGGATATAAAGAATGACAAAACTGTTGATGAG
phase 0
aadn02016908 (42328-42280)

TGGTTTCCCTTGAGCAAGACAACAAGTGGACACTTGCACTTAAAGCTGGAGTGGCTTTCA
CTAGTAAATGACCAAGAAAAGCTACACGAG
phase 0
aadn02016908 (41688-41599)

GATAAGAAGGGCCTGTCTACAGCAATTCTGATAGTCTACTTGGACAGCGCTTTCAACCTTCCA
phase 0
aadn02016908 (40736-40674)

AAAAACCACTTCGAGTATTCAAATGGTGAATGTGGAGCAAGGAAGATAAAAAATAACAAG
TACCTCAAG
phase 0
aadn02016908 (40569-40501)

AAGACAGAACGAGAACCTTCCTCCTTTGTCCTGCTCACAGTAGGAAGCAAGACTCAAAAA
AGCAAG
phase 0
aadn02016908 (39252-39187)

ACCTGCAATTTCAACAAAGATCCCAAGTGGGGCCAGGCTTTCACCTTCTTTGTCCACAGT
GCTCATTCCCAGTCACTGCACATTGAG
phase 0
aadn02016908 (38233-38147)

ATAAAAGACAAGGATCAGGATAGTTCTCTGGGAACATCGGTGGTATGTCTCTCTCACTTA
CTTAAGGACCCAAACATGACTCTGGATCAGAGATTTCAGCTGGACCATTCCAGTTCAGAC
AGCTTCATTAAGATTAAACTTGTGCTGCGG
phase 0
aadn02016908 (37827-37678)

GCTTTGAATGTTGAGGAACCTGATCCACAGAGAGTCAAGGCTGGTGTCGGTGCCACGAAG
AAAGGTCCCATGCATGTCATGGAGAAAGGTGGAAACCAGCAGGAGTTTGTCCCTCCACCA
AAACCAGAAGTCTCAAAAGTACCTCCTGTGAGCAAAGACACAGCTGTGCAGGAATCTCAG
CCAAAAAAAGACAGCAGTGAAGACCTGGACACAAGCAACAGTTCTGCAGTGCCTGCAGCA
AGTGAAACTGTTGCCAGCCTTGATGAGACAGAGAGTGAGCAAGACCCAGAGCATCGTGCG

CCCTCAGCACTGCATACCAGGGCAGCAGTAGTACCCATGTTGCCAGTCGTAGAGGAACTG
AGGCTTGCACCTAGTGTTACTTCACTGGGTTCTCTGCCTTCTTCTTGCTTTGAACTCAGT
AGCAGCAATCTGGACCTTCATAA
phase +2
aadn02016908 (37165-36723)

TGGGATGGAAATGCCTCTGGGAGAGATTCAGCTCACAGTGCGCTATGCTTCCATACGGCA
GAGCCTCGTTGTGCTGGTGAATGGCTGCAG
phase +2
aadn02016908 (36116-36027)

AAACTTAATACCCTCATCCAATCGTGGAGTGGATCCGTATGTCCGTATATATCTGCTTCC
AGATAGAAGATGGACAAGTAGGAAGAAGACTTCAGTTAAGAAAAGAACTCTGAACCCGCA
GTATGATGAAAA
phase +2
aadn02016908 (35602-35471)

GTTTGAATTTTTTGAATCTTTGGAAGAAGTCAAGAAAAGGACCCTCGATGTTGCAGTGAA
AAACAGCAGGCCATTCATTTCACAGGAAAAAAAGGAGCTGGGGAAA
phase 0
aadn02016908 (34220-34115)

GTGTGGATTGATTTGTCACAGGAGGATTTAATCAAGGGTTTTACACAGTG
phase +2
aadn02016908 (33518-33469)

GTATGAGCTGACAAGAAGTAGACGCAAGAAAAACTGA
aadn02016908 (32580-32544)

Bos taurus *FAM62* genes

Eukaryota; Metazoa; Chordata; Craniata; Vertebrata; Euteleostomi; Mammalia; Eutheria; Laurasiatheria; Cetartiodactyla; Ruminantia; Pecora; Bovidae; Bovinae; Bos.

FAM62A

MEPSLGDSSSSSPSPSPVDQPYPPSEPPDQPTTAHAKPEQSSGDQPAGPGAAGEALAVLT
SFGRRLLVLVPVYLAGAMGLSVGFVLFGLALYLGWRRVREEKERSLRVARQLLDDEERLT
AKTLYMSHRELPAW**^ 0**
VSFPDVEKAEWLNK**^ 0**
IVAQVWPFLGQYMEKLLAETVAPAVRGSNPHLQTFTFTRVELGEK**^ 0**
PLRILGVKVHTGQSKKQILLDLNI**^ +2**
SYVGDLQIDVEVKKYFCKAGVKGMQ**^ 0**
LHGVLRVILEPLMGDLPIVGAVSMFFIRRP**^ 0**
TLDINWTGMTNLLDIPGL**^ +2**
SSLSDTMIMDSIAAFLVLPNRLLVPLVPDLQDVAQLRSPLPR**^ 0**
GIIRIHLLAARGLSSKDKYVKGLIEGKSDPYALVRVGTQAFCSRVINEELNPQWGETYE**^ 0**
VMVHEVPGQEIEVEVFDKDPDKDDFLG**^ +2**
RMKLDVGKVLQAGVMDE**^ 0**
WFPLQGGQGQVHLRLEWLSLLPDAEKLEQ**^ 0**
ILQWNRGVSSRPEPPSAAILVVYLDRAQDLP**^ 0**
LKKGNKEPNPMVQLSIQDVTQESK**^ 0**
AVYSTNCPVWEQAFRFFLQDPQSQELDVQ**^ 0**
VKDDSRALTLGALTLPLARLLTAPELTLDQWFQLSSSGLNSRLYMKLVMR**^ 0**
LLYLDTSEVRFPAMPGIPGAWDLDDSPQTGSSVDVPPRPCHTTPDSSFGTE**^ 0**
NVLRLHVLEAQDLIAKDRFLGGLVKGKSDPYVKLKLAGRSFHSRVVREDLNPRWNEIFE**^ 0**
VIVTSIPGQELEVEVFDKDLDKDDFLG**^ +2**
RCKVSLTTVLNSGFLDE**^ 0**
WLTLEDVPSGRLHLRLERLTPRPTAAELEE**^ 0**
VLQVNSLIQTQKSAELAAALLSVYLERAEDLP**^ 0**
LRKGTKPPSPYATLAVGDTSHKTK**^ 0**
TVPQTATPVWNESASFLIRKPNTESLELQ**^ 0**
VRGEGSGTLGSLSLPLSELLVADRLCLDRWFTLINGQGQVLLRAQLG**^ 0**
ILVSQHSGVEAHSHSYSHSSSSLSELELWSGLPQVTSSAPELRQRLTHSD**^ +2**
SPPEAPAGPLGQVKLTVWYHSEERKLVGFIHSC**^ +2**
RALRQNGRDPPDPYVSLLLLPDKNRGTKRKTSQKKRTLNPEFNE**^ +2**
RFEWELPLDEALQRKLDVSVKSSSSFMSRERDLLGK**^ 0**

VQLDLAEIDLSQGAAQ**^ +2**
WYDLMDDKDKGSS

ATGGAGCCCTCTCTTGGAGACAGCTCTAGCTCTAGCCCCAGCCCCAGCCCGGTGGACCAG
CCCTACCCTCCCTCCGAACCCCCTGACCAGCCCACCACTGCTCACGCAAAGCCAGAGCAG
AGTTCTGGGGACCAACCTGCCGGCCCAGGAGCGGCGGGTGAGGCCTTGGCGGTGCTGACT
TCGTTCGGGCGGCGGTTGCTGGTGCTAGTGCCGGTGTACCTGGCCGGGGCAATGGGACTC
AGCGTGGGTTTTGTGCTCTTCGGCCTCGCCCTCTATCTGGGCTGGCGTCGGGTCCGCGAG
GAGAAAGAACGGAGCCTTCGAGTCGCGCGGCAGCTGCTGGACGATGAAGAACGGCTTACG
GCGAAAACTCTTTACATGAGCCATCGAGAGCTACCTGCCTGG
phase 0
ac156912.2 87582-87983

GTCAGCTTCCCAGATGTGGAAAAGGCTGAGTGGCTCAATAAG
phase 0
ac156912.2 89593-89634

ATTGTGGCCCAGGTCTGGCCCTTCCTGGGCCAGTATATGGAGAAGCTTCTCGCTGAAACT
GTGGCCCCAGCTGTTCGAGGATCTAACCCCCACCTGCAGACATTTACATTTACACGAGTG
GAACTGGGTGAAAAG
phase 0
ac156912.2 89806-89940

CCACTGCGCATCCTTGGAGTCAAGGTTCACACTGGTCAGAGCAAAAAACAGATCCTGCTG
GACTTGAACATCAG
phase +2
ac156912.2 90048-90121

CTACGTAGGTGACCTTCAGATTGATGTGGAAGTGAAGAAATACTTCTGCAAAGCAGGAGT
CAAGGGCATGCAG
phase 0
ac156912.2 90238-90310

CTCCATGGTGTCTTACGGGTGATTCTCGAACCACTCATGGGGGACCTTCCCATCGTGGGG
GCTGTGTCGATGTTCTTCATCCGACGCCCG
phase 0
ac156912.2 90466-90555

ACCCTAGATATCAACTGGACGGGGATGACCAACCTGCTGGATATCCCAGGACTCAG
phase +2
ac156912.2 90760-90815

CTCCCTCTCTGACACCATGATCATGGATTCCATCGCTGCCTTCCTTGTGTTGCCCAACCG
ATTGTTGGTGCCCCTTGTGCCTGACCTTCAAGATGTGGCCCAGTTGCGTTCCCCTCTTCC
CAGG
phase 0
ac156912.2 91413-91536

GGCATAATTCGGATTCACTTGCTGGCTGCACGAGGGCTGAGCTCCAAGGATAAATACGTT
AAGGGCCTGATTGAGGGCAAGTCAGACCCCTATGCGCTTGTGCGAGTGGGCACCCAGGCA
TTCTGCAGCCGTGTCATCAATGAGGAACTCAACCCCCAGTGGGGAGAGACTTATGAG
phase 0
ac156912.2 91624-91800

GTGATGGTGCATGAGGTCCCAGGACAGGAAATTGAGGTGGAGGTGTTTGACAAGGATCCA
GACAAAGATGACTTTTTGGGCAG
phase +2
ac156912.2 91920-92002

AATGAAGCTGGATGTAGGGAAAGTATTGCAGGCTGGAGTAATGGATGAA
phase 0
ac156912.2 92294-92342

TGGTTCCCTCTTCAAGGTGGGCAAGGCCAAGTTCACTTAAGGCTAGAATGGCTTTCGCTT

TTGCCAGATGCCGAAAAACTGGAGCAG
phase 0
ac156912.2 92472-92558

ATTCTACAGTGGAACCGTGGAGTCTCTTCCCGCCCAGAGCCCCCGTCGGCCGCCATCTTA
GTTGTTTATCTGGATCGGGCCCAGGATCTTCCT
phase 0
ac156912.2 92661-92753

CTGAAGAAGGGGAACAAGGAGCCCAACCCCATGGTACAACTGTCAATCCAGGATGTGACC
CAGGAGAGCAAG
phase 0
ac156912.2 92982-93053

GCTGTCTACAGCACCAACTGCCCAGTGTGGGAGCAGGCCTTCCGGTTCTTCCTGCAAGAC
CCTCAAAGCCAGGAGCTTGATGTGCAG
phase 0
ac156912.2 93231-93317

GTGAAGGATGACTCCAGGGCCCTGACTTTAGGGGCACTGACCCTGCCTCTGGCTCGCCTG
CTGACTGCCCCGGAACTCACCCTGGACCAGTGGTTCCAGCTCAGCAGCTCTGGCCTAAAC
TCCCGCCTCTACATGAAACTAGTTATGAGG
phase 0
ac156912.2 94455-94604

CTCTTATACTTGGATACATCAGAAGTGCGGTTCCCTGCTATGCCTGGTATTCCTGGGGCT
TGGGACCTGGATGATAGCCCTCAGACAGGCAGCAGTGTGGATGTCCCACCTCGACCCTGT
CACACTACTCCTGATAGTAGCTTTGGGACAGAG
phase 0
ac156912.2 95097-95249

AATGTGCTTCGGCTCCATGTATTAGAGGCCCAGGACCTGATTGCCAAAGACCGCTTCTTG
GGGGGATTAGTGAAGGGCAAGTCAGACCCCTACGTCAAACTAAAGCTGGCAGGACGAAGC
TTCCACAGCCGTGTTGTTCGGGAAGATCTCAATCCCCGTTGGAACGAGATCTTTGAG
phase 0
ac156912.2 95385-95561

GTGATCGTCACATCAATCCCAGGTCAAGAGCTAGAGGTTGAGGTTTTTGACAAGGACCTG
GACAAGGATGACTTTCTGGGCAG
phase +2
ac156912.2 95717-95799

GTGTAAAGTGAGTCTCACCACTGTCTTAAACAGTGGCTTCCTTGATGAG
phase 0
ac156912.2 95924-95972

TGGCTGACCCTGGAGGATGTCCCTTCTGGCCGCCTACACTTGCGTCTGGAGCGTCTGACC
CCCCGCCCCACTGCTGCTGAGTTAGAGGAG
phase 0
ac156912.2 96310-96399

GTGCTGCAGGTGAACAGCTTGATCCAGACCCAGAAGAGTGCAGAACTTGCAGCGGCCCTG
CTGTCTGTCTACCTGGAGCGGGCTGAGGACCTGCCG
phase 0
ac156912.2 96531-96626

CTCCGAAAAGGTACCAAGCCTCCCAGCCCTTATGCTACTCTTGCTGTGGGAGATACGTCT
CATAAAACTAAG
phase 0
ac156912.2 96879-96950

ACTGTTCCCCAAACGGCCACCCCCGTCTGGAATGAGAGCGCCTCCTTTCTCATCAGGAAA
CCAAACACTGAGAGTCTGGAGTTGCAG
phase 0
ac156912.2 97049-97135

GTTCGGGGGGAAGGCTCTGGCACGCTGGGCTCATTATCCCTGCCCCTCTCCGAGCTCCTT
GTGGCTGATCGGCTCTGCCTGGACCGCTGGTTTACGCTCATCAACGGTCAAGGGCAGGTG
CTACTGAGAGCACAGCTCGGG
phase 0
ac156912.2 99008-99148

ATCCTGGTGTCTCAGCACTCCGGGGTGGAAGCTCACAGCCACAGCTACAGCCACAGCTCC
TCATCTCTGAGTGAACTAGAGCTCTGGAGTGGACTCCCTCAGGTCACCTCTTCAGCCCCA
GAGCTCAGGCAGCGCCTAACACACAGTGACAG
phase +2
ac156912.2 99227-99378

TCCCCCTGAGGCTCCAGCCGGGCCGCTGGGCCAGGTGAAACTGACTGTTTGGTACCACAG
TGAAGAACGCAAGCTGGTTGGCTTCATTCACAGTTGCCG
phase +2
ac156912.2 99485-99583

GGCCCTTCGACAAAATGGACGGGATCCCCCCGATCCCTACGTGTCACTGTTGCTACTGCC
AGATAAGAACCGGGGCACCAAGAGGAAGACTTCACAGAAGAAGAGGACCCTAAATCCTGA
ATTCAACGAGCG
phase +2
ac156912.2 99668-99799

GTTTGAGTGGGAACTGCCCCTGGATGAGGCCCTCCAGCGAAAGCTGGATGTCTCTGTGAA
GTCTAGTTCCTCCTTCATGTCAAGAGAGCGTGACCTGCTGGGGAAG
phase 0
ac156912.2 99877-99982

GTGCAGTTGGACCTCGCTGAGATAGACCTTTCCCAGGGTGCGGCCCAGTG
phase +2
ac156912.2 100340-100389

GTATGACCTCATGGATGACAAGGACAAGGGCAGCTCCTAG
ac156912.2 100563-100602

FAM62B

MSGSRAPGPEVGASGAEPPGAALSVDVAGLLAQLARSFALLLPVYALGYLGLSFSWVLLA
LGLLVx**^ 0**
VHFPDTERAEWLNK**^ 0**
TVKHMWPFICQFIEKLFRETIEPAVRGAHTHLSTFSFTRVDLGQQ**^ 0**
PLRINGVKVYTENVDKRQIILDLQI**^ +2**
SFVGNCEIDLEIKRYFCRAGVQSIQ**^ 0**
IHGTMRVILEPLIGDMPLVGALSIFFLRKP**^ 0**
LLEINWTGLTNLLDIPGL**^ +2**
NGLSDTIILDIISNYLVLPNRITVPLVSEVQIAQLRFPVPK**^ 0**
GVLRIHFIEAQDLQGKDTYLKGLVKGKSDPYGVIRVGNQIFQSKVIKENLSPKWNEVYE**^ 0**
ALVYEHPGQELEIELFDEDPDKDDFLG**^ +2**
SLMIDLIEVEKERLLDE**^ 0**
WFTLDEVPRGKLHLKLEWLTLMPDASNLEQ**^ 0**
VLTDIRADKDQANDGLSSSLLILYLDSARNLP**^ 0**
SNPLEFNPDVLKKAAIQKALK**^ 0**
SGKKINSNPNPLVQMSVGHKAQESK**^ 0**
IRYKTNEPVWEENFTFFIHNPKRQELEVE**^ 0**
VRDEQHQCSLGNLRIPLSQLLAREDMTLNQRFQLSNSGPNSSLKMKLALR**^ 0**
VLHLEKQERPPDHQHSAQVKRPSVSKEGRKVSVRPQMSASPGSGDGGTAPSTPVAGGDER
PGVEERGLPVEAGLQGPRDLGRSSSSLQAGPTGSPSHVSVKEPTPSIASDISLPIATQEL
RQRLRQLE**^ +2**
NGTTLGQSPLGQIQLTIRHSSQRNKLVVVVHSC**^ +2**
RNLIAFSEDGSDPYVRLYLLPDKRRSGRRKTHVSKKTLNPVFDQ**^ +2**
SFDFSVSLPDVQRRTLDVAVKNSGGFLSKDKGLLGK**^ 0**
VLVGLASEELAKGWTQ**^ +2**
WYDLTEDGTRPHVVT

ATGAGCGGCTCCAGGGCTCCGGGCCCCGAGGTGGGCGCCTCCGGGGCAGAGCCCCCCGGC

GCGGCGCTGAGCGTGGACGTGGCAGGGCTGCTGGCACAGCTGGCGCGCAGCTTCGCGCTG
TTGTTGCCGGTGTACGCGCTGGGCTACCTGGGCCTGAGCTTCAGCTGGGTGCTGCTGGCG
CTCGGGCTGCTCGTGNNN
phase 0
aafc03066338.1 2543-2738 (frameshifts)

GTTCATTTTCCAGACACTGAGAGAGCAGAATGGCTAAATAAG
phase 0
ac170328.2 (146001-145960)

ACTGTAAAACACATGTGGCCTTTTATTTGCCAGTTTATAGAGAAGTTGTTTCGAGAAACC
ATAGAGCCGGCCGTGCGGGGCGCACACACCCACCTCAGCACCTTCAGCTTCACGAGGGTG
GACCTGGGGCAGCAG
phase 0
ac170328.2 (144732-144598)

CCCCTCAGGATCAATGGTGTTAAGGTGTATACTGAGAATGTGGATAAAAGGCAGATTATT
CTGGACCTTCAGATCAG
phase +2
ac170328.2 (142329-142253)

TTTTGTAGGGAATTGTGAGATCGATCTGGAGATCAAGCGATATTTTTGTAGAGCTGGTGT
GCAGAGTATACAG
phase 0
ac170328.2 (137970-137898)

ATTCATGGTACAATGCGGGTGATCCTGGAACCATTGATTGGAGACATGCCCTTAGTAGGA
GCTTTGTCTATCTTCTTCCTTAGGAAGCCA
phase 0
ac170328.2 (137679-137590)

CTTTTAGAAATTAACTGGACAGGACTGACTAATCTTCTCGACATTCCTGGATTGAA
phase +2
ac170328.2 (129609-129554)

TGGTTTATCAGATACTATCATTTTGGATATAATATCAAACTATCTGGTGCTTCCCAATCG
AATCACGGTCCCTCTTGTCAGCGAAGTTCAAATAGCTCAGCTGCGATTTCCTGTACCAAAG
phase 0
ac170328.2 (125850-125730)

GGTGTCCTGAGGATACATTTTATTGAAGCTCAGGATCTTCAAGGAAAAGACACTTACCTT
AAGGGACTTGTCAAGGGGAAGTCAGACCCCTACGGAGTGATTCGAGTTGGCAACCAGATC
TTCCAAAGCAAAGTCATCAAGGAGAACCTCAGTCCGAAGTGGAACGAGGTGTATGAG
phase 0
ac170328.2 (123542-123366)

GCTCTGGTCTATGAACATCCCGGACAGGAATTGGAGATCGAGCTCTTTGACGAAGACCCA
GACAAGGATGACTTCCTCGGAAG
phase +2
ac170328.2 (122387-122305)

TCTTATGATTGATCTTATTGAAGTTGAAAAGGAGCGCCTTTTAGATGAA
phase 0
ac170328.2 (120886-120838)

TGGTTCACCCTGGACGAGGTGCCCAGAGGGAAGCTGCACTTGAAGCTGGAGTGGCTCACG
CTCATGCCTGACGCGTCGAACCTTGAGCAG
phase 0
ac170328.2 (119793-119704)

GTGCTGACAGATATCAGAGCTGACAAAGACCAAGCCAATGACGGGCTGTCCTCCTCGCTG
CTCATCTTGTACTTGGACTCGGCCCGGAACCTTCCG
phase 0
ac170328.2 (119303-119208)

AGTAACCCATTAGAATTTAACCCTGATGTCTTGAAGAAGGCTGCAATTCAGAAAGCTTTAAAG
phase 0
ac170328.2 (114446-114384)

TCTGGGAAGAAAATAAACAGCAATCCAAATCCTCTTGTCCAAATGTCAGTTGGTCACAAG
GCCCAGGAGAGCAAG
phase 0
ac170328.2 (110795-110721)

ATTCGGTACAAAACCAACGAACCTGTGTGGGAGGAAAATTTCACCTTCTTCATTCACAAC
CCCAAGCGCCAGGAGCTCGAAGTGGAG
phase 0
ac170328.2 (109922-109836)

GTGAGAGATGAGCAGCACCAGTGTTCGCTGGGGAACCTCAGGATCCCTCTCAGCCAGCTG
CTCGCCCGCGAGGACATGACCCTGAACCAGCGGTTCCAGCTCAGCAACTCAGGTCCCAAC
AGCTCTCTGAAGATGAAGCTCGCCCTCAGG
phase 0
ac170328.2 (108095-107946)

GTGCTCCACCTGGAGAAGCAGGAGAGGCCTCCAGACCACCAGCACTCGGCTCAAGTGAAA
CGGCCTTCTGTGTCCAAGGAAGGCAGGAAAGTCTCTGTCAGGCCTCAGATGTCTGCATCG
CCGGGCTCGGGTGACGGTGGCACAGCCCCGTCCACACCAGTCGCCGGGGGCGATGAGAGG
CCTGGTGTGGAGGAGCGAGGCTTGCCCGTGGAGGCCGGCCTGCAGGGCCCAAGGGACCTC
GGCCGGAGCTCCTCCAGCCTCCAGGCGGGCCCCACCGGCTCCCCCAGCCACGTCTCCGTC
AAGGAACCCACCCCAAGCATCGCCTCGGACATCTCGCTGCCCATTGCCACACAGGAGCTG
CGGCAGAGGCTGCGGCAGCTTGAGAA
phase +2
ac170328.2 (106838-106453)

CGGAACCACCCTGGGACAGTCCCCCTTGGGGCAGATCCAGCTGACCATCCGGCACAGCTC
GCAGAGGAACAAGCTGGTGGTGGTTGTGCACTCCTGCAG
phase +2
ac170328.2 (105531-105433)

AAATCTCATCGCCTTCTCTGAGGATGGCTCTGATCCCTACGTCCGCCTGTATTTGTTACC
AGACAAGAGGAGGTCGGGAAGGAGGAAAACACACGTGTCGAAGAAGACACTGAACCCCGT
GTTTGATCAGAG
phase +2
ac170328.2 (104058-103927)

CTTTGATTTTAGCGTCTCACTGCCAGACGTGCAGAGGAGAACCTTGGATGTGGCCGTGAA
GAACAGCGGTGGCTTCCTGTCCAAAGACAAAGGGCTTCTTGGCAAA
phase 0
ac170328.2 (102669-102564)

GTGCTGGTTGGTCTAGCATCTGAAGAGCTCGCCAAAGGCTGGACCCAGTG
phase +2
ac170328.2 (102246-102197)

GTACGACCTCACAGAAGACGGGACGAGGCCCCACGTGGTGACGTAG
ac170328.2 (102105-102060)

FAM62C

MPSEDPCPPGARGTERAPGREPRLPSQLLPELYAFAARVLFCLAPVYLAGYLGLSITWLL
LGVLLWMWWRRNRRGKLGRLAAAFEFLDNERQFISRELLGQHLPAW**^ 0**
IHFPDVERVEWANK**^ 0**
IISQIWPYLSMIMENKFREKLEPKIREKSMHLRTFTFTKLYFGQK**^ 0**
CPRVNGVKTHTDQRNRRQVVLDLQI**^ +2**
CYIGDCEINVELQKIQAGVNGIQ**^ 0**
LQGTLRIILEPLLVDKPFVGAVTMFFLQKP**^ 0**
HLQINWTGLTNLLDAPGI**^ +2**

NEMTDSLLEDLIAAHLVLPNRVTVPVKKGLDVTNLRFPLPC**^ 0**
GVIRVHLLEAEKLAQKDSFLGIRGKSDPYAKVNIGLQHFRSRTIYKNLSPTWNEVFE**^ 0**
FIVYEVPGQDLEVDLYDEDPDKDDFLG**^ +2**
SLQICLGDVMTNRVVDE**^ 0**
WFALNNTTSGRLHLRLEWLSLITEPDAVTE**^ 0**
DHGSFSTAILVVFLESACNLP**^ 0**
RSPFEYLNGEYQAKKLSRFAR**^ 0**
NKVSRDPSSYVRLSVGKKTHLSK**^ 0**
TCPCSKDPVWSQVFSFFVHNVAAEQLHLK**^ 0**
VLDDDQECALGVLEFPLCQILPHADLTLEQRFQLDHSGLDSLISMRLVLR**^ 0**
FLHLEERELGSPYTGPEALKKGPLFIKKVTANQDPRAPPQGEGLTDPDPASEIKQAAKNT
TSATITATKPMLEETGPEPKGKDSAPMGKKSLATIYLAVPGPHSPGPIKSPRPMKCPASP
FAWPPKGLAPSMSSLNSLASSCFDLTDISLNI**^ +2**
EGGELGHRGLGEIQLTVRYVCLRRCLSVLINGC**^ +2**
RNLTPCTSSGADPYVRVYLLPERRWASRKKTSVKRKTLEPRFDE**^ +2**
TFEFFVSMDEVQKRSLDVAVKNSRPLGSHRRKELGK**^ 0**
VLIDLSKEDLIKGFSQ**^ +2**
WYELTPDGQPRS

<u>ATG</u>CCCTCGGAGGACCCCTGCCCCCCCGGCGCCCGGGGGACCGAGCGCGCGCCGGGCCGC
GAGCCGCGCCTACCCAGCCAGCTGCTGCCAGAGCTCTACGCCTTCGCGGCGCGCGTGCTGTTCTGC
CTGGCGCCCGTCTACCTAGCCGGCTACCTGGGACTCAGCATCACCTGGCTGCTGCTCGGCGTCCTG
CTGTGGATGTGGTGGCGCAGGAACCGCCGCGGGAAGCTCGGGCGCCTGGCTGCCGCCTTCGAATTC
CTAGACAACGAACGCCAGTTCATCAGCCGCGAGCTGCTGGGCCAGCACCTGCCCGCTTGG
phase 0
ac156133.2 (48588-48271)

ATTCACTTCCCGGACGTGGAACGGGTTGAATGGGCCAACAAG
phase 0
ac156133.2 (254281-254240)

ATCATCTCCCAGATCTGGCCCTACCTCAGCATGATCATGGAAAACAAGTTCCGGGAGAAACTGGAG
CCCAAGATCCGGGAGAAGAGCATGCACCTGAGGACCTTCACCTTCACCAAGCTCTACTTTGGACAGAAG
phase 0
ac156133.2 (252688-252554)

TGCCCCAGGGTCAACGGCGTCAAGACGCACACAGACCAGCGCAACCGCAGACAGGTGGTCCTGGAC
TTGCAGATCTG
phase +2
ac156133.2 (248457-248381)

CTATATCGGGGACTGTGAGATCAATGTGGAGCTGCAGAAGATACAGGCTGGTGTGAACGGGATCCAG
phase 0
ac156133.2 (246846-246780)

CTGCAGGGCACGCTGCGGATCATCCTGGAGCCCCTACTGGTGGACAAGCCCTTTGTGGGAGCTGTG
ACTATGTTCTTCCTTCAAAAGCCG
phase 0
ac156133.2 (246180-246091)

CACCTGCAGATCAACTGGACAGGCCTGACCAACCTGCTGGATGCACCAGGAATCAA
phase +2
ac156133.2 (245615-245560)

TGAGATGACAGATAGCCTGCTGGAGGACCTCATCGCCGCCCACCTGGTGCTGCCCAACCGTGTGAC
TGTGCCCGTGAAGAAGGGGCTGGACGTAACCAACCTGCGCTTCCCTCTGCCCTGT
phase 0
ac156133.2 (244200-244080)

GGAGTGATCAGAGTCCACTTGCTGGAGGCTGAAAAGCTGGCCCAGAAGGACAGCTTCCTGGGAATC
CGAGGCAAGTCAGACCCCTACGCCAAGGTGAACATCGGCCTGCAGCACTTCCGGAGCAGGACCATC
TATAAGAACCTGTCCCCCACCTGGAATGAGGTGTTTGAG
phase 0
ac156133.2 (238910-238740)

TTCATAGTGTATGAAGTCCCTGGGCAGGACCTGGAAGTGGACCTGTATGATGAGGATCCTGACAAG

GACGACTTCTTGGGCAG
phase +2
ac156133.2 (238064-237982)

CCTGCAGATCTGCCTCGGGGATGTCATGACCAACAGAGTGGTGGATGAG
phase 0
ac156133.2 (236375-236327)

TGGTTTGCGCTGAACAACACAACGAGCGGGCGGCTGCACCTGCGGCTGGAATGGCTTTCACTGATC
ACAGAGCCGGACGCTGTGACTGAG
phase 0
ac156133.2 (236126-236037)

GACCATGGGAGCTTTTCCACTGCCATCCTCGTGGTCTTCTTGGAGAGCGCCTGCAACCTGCCG
phase 0
ac156133.2 (235656-235594)

AGAAGCCCTTTTGAGTACCTAAATGGTGAATATCAAGCCAAAAAGCTGTCCAGATTTGCCAGG
phase 0
ac156133.2 (235490-235428)

AATAAAGTCAGCAGAGACCCTTCTTCCTACGTCAGGCTCTCTGTAGGCAAGAAGACACACCTGAGCAAG
phase 0
ac156133.2 (234894-234826)

ACCTGTCCCTGCAGCAAGGACCCTGTGTGGAGCCAGGTGTTCTCCTTCTTCGTGCACAATGTGGCT
GCTGAACAGCTGCATCTGAAG
phase 0
ac156133.2 (234294-234208)

GTGCTTGATGACGACCAAGAGTGTGCTCTAGGAGTGCTGGAGTTCCCCCTGTGCCAGATTCTCCCC
CATGCAGACCTTACTCTGGAGCAGCGCTTCCAGCTGGACCACTCAGGCCTGGACAGCCTCATCTCC
ATGAGGCTGGTGCTTCGG
phase 0
ac156133.2 (233642-233493)

TTCCTGCACTTGGAAGAACGGGAGCTGGGAAGTCCATACACAGGACCTGAAGCCCTAAAGAAAGGC
CCTCTGTTCATTAAGAAGGTCACCGCCAACCAAGACCCTAGAGCCCCACCCCAGGGAGAGGGCCTT
ACAGATCCAGACCCTGCTTCTGAGATCAAGCAAGCCGCCAAGAACACCACTTCTGCCACCATCACT
GCCACCAAGCCCATGCTTGAAGAGACGGGCCCAGAGCCCAAAGGCAAGGACAGTGCACCCATGGGG
AAGAAGAGTCTGGCCACTATCTACCTGGCTGTGCCAGGCCCCCACTCTCCGGGGCCCATCAAGTCA
CCCAGACCCATGAAATGCCCTGCCTCCCCATTCGCATGGCCACCCAAAGGGCTGGCTCCCAGCATG
TCCTCGCTCAACTCCCTGGCCTCTTCCTGCTTTGACCTGACAGATATCAGCCTCAACATTGA
phase +2
ac156133.2 (231811-231354)

AGGCGGGGAGCTTGGGCATCGGGGGCTGGGTGAGATTCAGCTCACGGTGCGCTATGTGTGTCTGCG
GCGCTGCCTCAGCGTGCTCATCAACGGCTGCAG
phase +2
aafc03071617.1 (412-314)

AAACCTGACGCCCTGTACCAGCAGTGGAGCTGATCCCTACGTCCGAGTCTACTTGTTGCCAGAAAG
GAGGTGGGCAAGTCGTAAGAAGACCTCAGTGAAGCGGAAGACCCTGGAGCCCCGGTTTGATGAGAC
phase +2
ac156133.2 (229827-229696)

ATTTGAATTTTTTGTTTCCATGGATGAAGTACAGAAGAGGTCACTAGATGTCGCAGTGAAAAACAG
TAGACCACTTGGTTCACACAGAAGGAAGGAACTGGGAAAG
phase 0
ac156133.2 (224534-224429)

GTACTGATTGACTTATCAAAAGAAGATCTGATTAAGGGCTTCTCACAGTG
phase +2
ac156133.2 (223596-223547)

GTATGAGCTGACTCCAGATGGACAGCCTAGAAGTTGA
ac156133.2 (223212-223176)

Canis familiaris *FAM62* genes

Eukaryota; Metazoa; Chordata; Craniata; Vertebrata; Euteleostomi; Mammalia; Eutheria; Laurasiatheria; Carnivora; Fissipedia; Canidae; Canis.

FAM62B

xLLLPVYALGYLVLSFSWVLLALGLLLWCRRSRGLKATRLCRALALLEDEERAVRLGVRA
CDLPAW**^ 0**
VHFPDTERAEWLNK**^ 0**
TVKHMWPFICQFIEKLFRETIEPAVRGANTHLSTFSFTKVDVGQQ**^ 0**
PLRINGVKVYTENVDKRQIILDLQI**^ +2**
SFVGNCEIDLEIKRYFCRAGVQSIQ**^ 0**
IHGTMRVILEPLIGDMPLVGALSIFFLRKP**^ 0**
LIEINWTGLTNLLDIPGL**^ +2**
NGLSDTIILDIISNYLVLPNRITVPLVSEVQIAQLRFPIPK**^ 0**
GVLRIHFIEAQDLQGKDTYLKGLIKGKSDPYGIIRVGNQIFQSKVIKESLSPKWNEVYE**^ 0**
ALVYEHPGQELEIELFDEDPDKDDFLG**^ +2**
SLMIDLIEVEKERLLDE**^ 0**
WFALDEVPRGKLHLKLEWLTLMPNASNLDK**^ 0**
VLTDIRADKDQANDGLSSSLLILYLDSARNLP**^ 0**
SNPLEFNPDVLKKSAVQKALK**^ 0**
SGKKINSNPNPLVQMSVGHKAQESK**^ 0**
IRYKTNEPVWEENFTFFIHNPKRQDLQVE**^ 0**
VKDEQHQCSLGHLKIPLSQLLTSDDMTMNQRFQLSNSGPNSTLKMKIALR**^ 0**
VLHLEKQERSPNHQHSAQVKRPSVSKEGRKVSVRSHMSASPGSGDSSTAPCTPVIGSSDK
PGVEDRAQPVEASPQGPRDLGRSSSSLQAGATCSPSHISVKEPTPSIASDISLPIATQEL
RQRLRQLE**^ +2**
NGMTLGQSPLGQIQLTIRHSSQRNKLVVVVHSC**^ +2**
RNLIAFSEDGSDPYVRMYLLPDKRRSGRRKTHVSKKTLNPVFDQ**^ +2**
SFDFSVSLPEVQRRTLDVAVKNSGGFLSKDKGLLGK**^ 0**
VLVGLASEELAKGWTQ**^ +2**
WYDLTEDGTRPHVVT

nnGCTGCTGCTGCCGGTGTACGCGCTCGGCTACCTCGTACTGAGCTTCAGCTGGGTGCTC
TTGGCGCTCGGGCTGCTCTTGTGGTGCCGCAGGAGCCGCGGCCTCAAAGCGACGCGCCTG
TGCCGGGCGCTGGCGCTGCTGGAGGACGAGGAGCGCGCCGTGCGCCTGGGGGTGCGCGCC
TGCGACCTGCCGGCCTGG
phase 0
aaex02035677.1 (31096-30901)

GTTCATTTTCCAGACACTGAAAGAGCAGAATGGCTAAATAAG
phase 0
aaex02035677.1 (1050-1009)

ACTGTAAAACATATGTGGCCTTTTATTTGCCAGTTTATAGAGAAGTTGTTTCGAGAAACT
ATTGAGCCAGCTGTGCGGGGAGCAAACACCCACCTTAGCACTTTCAGTTTCACAAAAGTT
GACGTGGGCCAACAG
phase 0
aaex02035677.1 (154-20)

CCTCTTAGGATCAATGGTGTTAAGGTGTACACTGAAAATGTGGACAAAAGGCAAATTATT
TTGGACCTTCAGATTAG
phase +2
aaex02035676.1 (53681-53605)

TTTTGTAGGAAACTGTGAGATTGATTTGGAGATCAAGCGATATTTTTGTAGAGCCGGTGT
TCAAAGTATACAG
phase 0
aaex02035676.1 (49424-49352)

ATTCATGGTACAATGCGGGTGATCCTGGAACCGTTGATTGGAGACATGCCCTTAGTGGGA

GCCTTGTCCATTTTCTTCCTTAGGAAACCA
phase 0
aaex02035676.1 (49114-49025)

CTTATAGAAATTAACTGGACAGGACTGACTAATCTTCTCGATATCCCAGGATTAAA
phase +2
aaex02035676.1 (41422-41367)

TGGTCTGTCGGATACTATCATTCTGGATATAATATCAAACTATCTGGTGCTTCCCAATCG
GATCACTGTTCCTCTTGTCAGTGAAGTTCAAATAGCTCAATTGCGGTTTCCTATACCAAAG
phase 0
aaex02035676.1 (35731-35611)

GGTGTTCTAAGGATACATTTTATTGAAGCTCAGGATCTTCAAGGGAAAGATACTTATCTT
AAGGGACTCATCAAGGGAAAGTCAGACCCATATGGAATTATTCGAGTTGGCAACCAAATC
TTCCAAAGCAAGGTCATCAAAGAGAGCCTCAGTCCAAAGTGGAATGAGGTATATGAG
phase 0
aaex02035676.1 (33180-33004)

GCTTTAGTCTATGAACATCCTGGACAGGAATTAGAGATTGAGCTCTTTGATGAAGACCCA
GACAAGGATGACTTCCTAGGAAG
phase +2
aaex02035676.1 (32002-31920)

TCTTATGATTGATCTTATTGAAGTTGAAAAGGAGCGCCTTTTAGATGAA
phase 0
aaex02035676.1 (30527-30479)

TGGTTCGCCCTGGACGAGGTCCCCAGAGGAAAGCTCCACTTGAAGCTGGAGTGGCTCACG
TTAATGCCAAATGCTTCAAACCTTGACAAG
phase 0
aaex02035676.1 (29388-29299)

GTGCTAACAGACATCAGGGCTGACAAAGACCAAGCCAATGATGGTCTTTCCTCTTCGTTG
CTGATCTTGTATTTGGATTCAGCAAGGAACCTTCCG
phase 0
aaex02035676.1 (28834-28739)

AGTAACCCATTAGAATTTAACCCTGATGTCTTGAAGAAGTCTGCAGTTCAGAAAGCTTTAAAG
phase 0
aaex02035676.1 (22547-22485)

TCAGGGAAGAAAATAAACAGCAATCCAAATCCTCTTGTGCAGATGTCAGTTGGACACAAG
GCCCAGGAGAGCAAG
phase 0
aaex02035676.1 (19308-19234)

ATTCGATACAAAACCAATGAACCCGTGTGGGAAGAAAACTTCACCTTCTTCATTCACAAT
CCCAAGCGCCAGGACCTCCAAGTCGAG
phase 0
aaex02035676.1 (18069-17983)

GTCAAAGATGAACAGCATCAGTGTTCTCTGGGGCATCTGAAGATCCCTCTCAGTCAGCTG
CTTACCAGCGATGACATGACCATGAACCAGCGATTCCAACTCAGTAATTCAGGCCCCAAC
AGCACTCTAAAGATGAAGATTGCCCTCAGG
phase 0
aaex02035676.1 (16260-16111)

GTACTCCACCTTGAAAAGCAAGAAAGGTCTCCCAACCACCAACACTCAGCTCAAGTAAAA
CGACCTTCTGTTTCTAAAGAAGGGAGGAAAGTGTCTGTCAGATCTCATATGTCTGCATCA
CCAGGTTCTGGTGACAGCAGCACGGCCCCATGCACACCAGTTATTGGGAGCAGTGATAAG
CCTGGTGTGGAAGACAGAGCCCAGCCTGTGGAGGCCAGCCCCCAGGGGCCCCGGGACCTG
GGCAGGAGCTCCTCCAGCCTCCAGGCTGGTGCCACCTGCTCCCCAAGCCACATCTCCGTC
AAGGAGCCCACCCCAAGCATCGCCTCTGACATATCACTGCCCATTGCCACCCAGGAGCTT

CGGCAGAGGCTGCGGCAGCTGGAGAA
phase +2
aaex02035676.1 (15003-14618)

TGGGATGACCCTGGGGCAGTCGCCCCTGGGGCAGATCCAGCTGACCATCCGGCACAGCTC
ACAGAGAAACAAGCTGGTGGTGGTCGTGCACTCCTGCAG
phase +2
aaex02035676.1 (13199-13101)

AAATCTCATTGCCTTCTCTGAAGATGGCTCTGACCCATATGTCCGCATGTATTTGTTACC
AGACAAGAGGAGATCAGGAAGGAGAAAAACACACGTGTCAAAGAAAACACTGAACCCTGT
GTTTGACCAGAG
phase +2
aaex02035676.1 (11605-11474)

CTTTGATTTCAGTGTTTCATTACCAGAAGTACAGAGGAGAACGTTGGATGTAGCAGTAAA
GAACAGCGGCGGCTTCCTGTCCAAAGATAAAGGGCTTCTTGGCAAG
phase 0
aaex02035676.1 (9631-9526)

GTCCTGGTGGGTCTGGCATCCGAAGAACTAGCCAAGGGTTGGACCCAGTG
phase +2
aaex02035676.1 (8872-8823)

GTACGACCTCACGGAAGATGGCACGAGACCCCACGTGGTGACGTAG
aaex02035676.1 (8728-8683)

FAM62C

xSVYLAGYLGLSITWLLLGALLWMWWRRNRRGKLGRLAAAFQFLDNERQFISRELRGQHL
PAW**^ 0**
IHFPDVERVEWANK**^ 0**
IISQIWPYLSMIMENKFREKLEPKIREKSSHLRTFTFTKLYFGQK**^ 0**
CPRVNGVKAHTNKRNRRQVVLDLQI**^ +2**
CYIGDCEISAELQKIQAGVNGIQ**^ 0**
LQGTLRVILEPLLVDKPFVGAVTVFFLQKP**^ 0**
HLQINWTGLTNLLDAPGI**^ +2**
NEISDSLLEDLIATHLVLPNRVTVPVKKGLDVTNLLFPLPC**^ 0**
GVIRVHLLEAEKLAQKDNFLGIRGKSDPYAKVSIGLQQFRSKTIYKNLNPTWNEVFE**^ 0**
FVVYEVPGQDLEVDLYDEDPDRDDFLG**^ +2**
SLQICLGDVMTNRVVDE**^ 0**
WFVLNDTTSGRLHLRLEWLSLIANPEALIE**^ 0**
DQGGLSTAILIVFLESACNLP**^ 0**
RNPFDYLNGEYRAKKLSRFTK**^ 0**
NKVSRDPSSYVKLSVGKKTQMSK**^ 0**
TCPHSKDPVWSQMFSFFVYNVAAEELHLK**^ 0**
VLDDTQECALGVLEFPLCQILPYTDLTLEQRFQLDHSGLDSLISMRLVLR**^ 0**
FLRVEEREMGSPYTGPEALKKGPLFIKKVDTNQNPKAPTQGECPANLPCPPDPASDTKEA
SKSTTTTTSATIVATEPTPQDTGPEPKGKDSARGFCEPMGKKKSSATIFLTVPGPHSPGP
IKSPRPMKCPASPLAWPPKRLAPSMSSLNSLASSCFDLTEISFNI**^ +2**
EGGDLRQWRLGEIQLTVRYVCLRRCLSVLINGC**^ +2**
RNLTPCTSSGADPYVRIYLLPERRWASRKKTSVKRKTLEPLFDE**^ +2**
TFEFFVPMEEVKKRSLDVAVKNSRPLGSHRRKELGK**^ 0**
VLIDLSKEDLIKGFSR**^ +2**
WYELTPDGQPRS

nGCTCCGTCTACCTGGCCGGCTACCTGGGGCTCAGCATAACCTGGCTGCTGCTCGGCGCT
CTGCTGTGGATGTGGTGGCGCAGGAACCGGCGCGGGAAGCTCGGGCGCCTGGCGGCCGCC
TTCCAGTTCCTGGACAACGAGCGCCAGTTCATTAGCCGGGAGCTGCGGGGCCAGCACCTG
CCGGCCTGG
phase 0
aaex02018233.1 (61978-61791)

ATCCACTTCCCGGATGTGGAGCGGGTCGAGTGGGCCAACAAG
phase 0
aaex02018233.1 (43580-43539)

ATCATCTCTCAGATCTGGCCCTACCTGAGCATGATCATGGAAAACAAGTTCCGGGAAAAG
CTCGAGCCCAAGATCCGAGAGAAGAGCAGCCACCTGAGGACCTTCACATTCACCAAGCTC
TACTTTGGACAAAAG
phase 0
aaex02018233.1 (41655-41521)

TGCCCCAGGGTCAACGGTGTCAAGGCGCACACTAACAAGCGCAACCGAAGACAGGTGGTC
CTGGACCTGCAGATTTG
phase +2
aaex02018233.1 (38774-38698)

CTACATCGGGGACTGCGAGATCAGTGCGGAGCTGCAGAAGATACAGGCTGGCGTGAACGG
GATTCAG
phase 0
aaex02018233.1 (36831-36765)

TTGCAGGGCACGCTGCGGGTCATCCTGGAGCCCCTCCTGGTGGACAAGCCCTTTGTGGGA
GCCGTTACCGTGTTCTTCCTTCAAAAGCCG
phase 0
aaex02018233.1 (36126-36037)

CATCTGCAGATCAACTGGACAGGCCTGACCAACCTGTTGGATGCACCGGGAATCAA
phase +2
aaex02018233.1 (35597-35542)

TGAGATATCAGACAGCCTGCTGGAGGACCTCATTGCCACCCACCTGGTGCTGCCCAACCG
CGTGACCGTGCCTGTGAAGAAGGGGCTGGATGTGACCAACCTGCTCTTCCCTCTGCCCTGC
phase 0
aaex02018233.1 (34168-34048)

GGAGTGATCAGAGTCCACTTGCTGGAGGCAGAGAAGCTGGCTCAGAAGGACAACTTCCTA
GGGATCCGAGGCAAGTCAGACCCCTACGCCAAGGTGAGCATTGGCCTACAGCAATTCCGG
AGTAAGACCATCTACAAGAACCTGAACCCCACCTGGAATGAGGTATTTGAG
phase 0
aaex02018233.1 (32540-32370)

TTTGTAGTGTATGAAGTCCCTGGGCAGGACCTAGAGGTGGACCTGTATGATGAGGATCCC
GACAGGGACGACTTCCTGGGCAG
phase +2
aaex02018233.1 (31919-31837)

CCTGCAGATCTGCCTCGGGGATGTCATGACGAACAGAGTGGTGGATGAG
phase 0
aaex02018233.1 (30237-30189)

TGGTTTGTCCTGAACGACACAACCAGTGGGCGGCTGCACCTGCGGCTGGAGTGGCTTTCG
CTCATCGCCAACCCAGAAGCTCTGATTGAG
phase 0
aaex02018233.1 (29924-29835)

GACCAAGGTGGCCTTTCAACTGCCATCCTCATAGTCTTCTTGGAGAGTGCCTGCAACCTGCCG
phase 0
aaex02018233.1 (29332-29270)

AGAAATCCTTTTGACTACCTGAATGGTGAATATCGAGCCAAAAAACTCTCCAGGTTTACCAAG
phase 0
aaex02018233.1 (29173-29111)

AATAAAGTCAGCAGAGACCCTTCTTCCTATGTCAAGCTATCTGTAGGCAAGAAGACACAG
ATGAGCAAG
phase 0
aaex02018233.1 (28576-28508)

ACCTGTCCCCACAGCAAGGACCCAGTGTGGAGCCAGATGTTCTCCTTCTTTGTGTACAAT
GTGGCAGCTGAGGAGCTGCATCTGAAG
phase 0
aaex02018233.1 (28059-27973)

GTGCTTGATGATACCCAAGAGTGTGCTCTGGGAGTGCTGGAGTTCCCCCTGTGCCAGATC
CTCCCCTACACAGACCTCACCCTCGAGCAGCGCTTCCAGCTGGACCACTCAGGCCTAGAC
AGCCTCATCTCCATGAGGCTGGTGCTTCGG
phase 0
aaex02018233.1 (27328-27179)

TTCCTGCGCGTGGAAGAACGAGAGATGGGGAGCCCATACACAGGACCTGAAGCCCTAAAG
AAAGGTCCTCTGTTCATTAAGAAGGTGGACACCAACCAGAACCCCAAAGCCCCAACCCAG
GGAGAGTGCCCTGCAAATTTGCCATGCCCCCCAGATCCTGCTTCTGATACCAAAGAAGCC
TCCAAGAGCACCACGACAACCACCAGTGCCACTATTGTTGCCACCGAGCCCACACCCCAA
GACACAGGCCCAGAGCCCAAAGGCAAGGACAGTGCCAGAGGGTTCTGTGAGCCCATGGGG
AAGAAGAAGAGTTCGGCCACCATCTTCCTGACTGTCCCAGGCCCCCACTCTCCAGGGCCC
ATCAAGTCACCCAGACCCATGAAATGCCCTGCCTCCCCACTCGCATGGCCGCCCAAGAGG
CTAGCTCCCAGCATGTCCTCGCTCAACTCCCTGGCCTCCTCCTGCTTTGATCTGACAGAG
ATCAGCTTCAACATTGA
phase +2
aaex02018233.1 (25336-24840)

AGGCGGGGATCTCAGGCAGTGGAGGCTGGGTGAGATTCAGCTCACAGTGCGTTATGTGTG
TCTGCGGCGCTGCCTCAGTGTGCTCATCAATGGCTGCAG
phase +2
aaex02018233.1 (24202-24104)

AAACCTGACACCCTGTACCAGCAGTGGAGCTGATCCCTATGTCCGCATCTACTTGTTACC
AGAAAGGAGGTGGGCAAGTCGTAAGAAGACCTCAGTAAAACGGAAGACCCTGGAACCCCT
GTTTGATGAGAC
phase +2
aaex02018233.1 (23604-23473)

ATTTGAATTTTTTGTTCCCATGGAAGAAGTAAAGAAGAGGTCACTAGATGTTGCAGTGAA
AAACAGTAGGCCACTTGGCTCACATAGAAGGAAGGAACTAGGAAAA
phase 0
aaex02018233.1 (17164-17059)

GTACTGATTGACTTATCAAAAGAGGATCTAATTAAGGGCTTCTCACGATG
phase +2
aaex02018233.1 (16076-16027)

GTATGAGCTGACTCCAGATGGACAGCCCAGAAGTTGA
aaex02018233.1 (15686-15650)

Mus musculus *FAM62* genes

Eukaryota; Metazoa; Chordata; Craniata; Vertebrata; Euteleostomi; Mammalia; Eutheria; Euarchontoglires; Glires; Rodentia; Sciurognathi; Muroidea; Muridae; Murinae; Mus.

FAM62A

MEHSPEEGASPEPSGQPPATDSTRDGGSGVPPAGPGAASEALAVLTSFGRRLLVLVPVYL
AGAAGLSVGFVLFGLALYLGWRRVRDGKERSLRAARQLLDDEERITAETLYMSHRELPAW**^ 0**
VSFPDVEKAEWLNK**^ 0**
IVAQVWPFLGQYMEKLLAETVAPAVRGANPHLQTFTFTRVELGEK**^ 0**
PLRIIGVKVHPSQRKDQILLDLNV**^ +2**
SYVGDVQIDVEVKKYFCKAGVKGMQ**^ 0**
LHGVLRVILEPLTGDLPIVGAVSMFFIKRP**^ 0**
TLDINWTGMTNLLDIPGL**^ +2**
SSLSDTMIMDSIAAFLVLPNRLLVPLVPDLQDVAQLRSPLPR**^ 0**
GIIRIHLLAARGLSSKDKYVKGLIEGKSDPYALVRVGTQTFCSRVIDEELNPHWGETYE**^ 0**
VIVHEVPGQEIEVEVFDKDPDKDDFLG**^ +2**
RMKLDVGKVLQAGVLDN**^ 0**

WYPLQGGQGQVHLRLEWLSLLPDAEKLDQ**^ 0**
VLQWNRGITSRPEPPSAAILVVYLDRAQDLP**^ 0**
LKKGNKEPNPMVQLSVQDVTRESK**^ 0**
ATYSTNSPVWEEAFRFFLQDPRSQELDVQ**^ 0**
VKDDSRALTLGALTLPLARLLTASELTLDQWFQLSSSGPNSRLYMKLVMR**^ 0**
ILYLDYSEIRFPTVPGAQDWDRESLETGSSVDAPPRPYHTTPNSHFGTE**^ 0**
NVLRIHVLEAQDLIAKDRFLGGLVKGKSDPYVKLKVAGKSFRTHVVREDLNPRWNEVFE**^ 0**
VIVTSIPGQELEIEVFDKDLDKDDFLG**^ +2**
RYKVSLTTVLNSGFLDE**^ 0**
WLTLEDVPSGRLHLRLERLTPRPTAAELEE**^ 0**
VLQVNSLIQTQKSSELAAALLSVFLERAEDLP**^ 0**
LRKGTKPPSPYATITVGETSHKTK**^ 0**
TVSQSSAPVWEESASFLIRKPHAESLELQ**^ 0**
VRGEGTGTLGSVSLPLSELLQEDQLCLDHWFALSGQGQVLMRAQLG**^ 0**
ILVSQHSGVEAHSHSYSHSHSSSSLNDEPEALGGPTHPASPVLEVRHRLTHGD**^ +2**
SPSEAPVGPLGQVKLTVWYHSDEQKLISIIHSC**^ +2**
RALRQNGRDLPDPYVSVLLLPDKNRSTKRKTPQKKRTLNPEFNE**^ +2**
RFEWDLPLDGTLRRKLDVSVKSNSSFMSRERELLGK**^ 0**
VQLDLAEIDLSQGAAQ**^ +2**
WYDLMDDRDKGGS

ATGGAGCACTCCCCTGAAGAGGGCGCCAGCCCCGAGCCGTCAGGGCAGCCCCCTGCCACG
GACTCCACGCGGGACGGGGGCTCCGGGGTCCCACCTGCCGGCCCAGGTGCGGCGAGCGAG
GCCCTGGCGGTGCTGACTTCCTTCGGGCGCCGCTTGTTGGTGCTGGTGCCGGTGTACCTG
GCAGGGGCAGCGGGTCTTAGCGTAGGTTTCGTGCTTTTCGGCCTCGCCCTGTACTTGGGC
TGGCGCCGGGTCCGCGATGGGAAAGAACGGAGCCTGAGGGCAGCGAGGCAGCTGCTGGAT
GACGAGGAGCGGATCACCGCAGAGACGCTTTATATGAGCCACCGGGAACTACCTGCCTGG
phase 0
ac122159.30 48133-48492

GTCAGCTTCCCAGATGTGGAAAAGGCCGAATGGCTGAACAAG
phase 0
ac122159.30 50841-50882

ATCGTGGCTCAGGTGTGGCCCTTCCTAGGCCAGTATATGGAGAAGCTTCTGGCGGAGACA
GTGGCCCCAGCTGTCCGGGGAGCTAACCCTCATCTGCAGACATTCACATTCACACGTGTG
GAGCTGGGTGAAAAG
phase 0
ac122159.30 51401-51535

CCATTACGAATCATTGGCGTCAAAGTTCACCCTAGTCAGAGGAAAGATCAGATTCTGTTG
GACTTGAATGTCAG
phase +2
ac122159.30 51634-51707

CTATGTAGGTGATGTACAGATTGACGTGGAGGTGAAGAAATATTTCTGCAAAGCTGGAGT
CAAGGGCATGCAG
phase 0
ac122159.30 51829-51901

CTCCATGGTGTCTTGCGAGTGATTCTTGAGCCACTCACAGGGGACCTTCCTATCGTGGGG
GCTGTGTCCATGTTCTTTATCAAACGCCCG
phase 0
ac122159.30 52040-52129

ACGCTTGACATCAACTGGACAGGGATGACCAACCTGCTAGATATCCCAGGACTTAG
phase +2
ac122159.30 52287-52342

CTCACTCTCTGACACCATGATCATGGACTCCATTGCTGCCTTCCTCGTGCTCCCTA
ACCGACTGTTGGTGCCCCTTGTGCCCGACCTTCAAGATGTGGCCCAGCTGCGGTCC
CCACTACCCAGG
phase 0
ac122159.30 52926-53049

GGCATTATCCGGATTCACCTGCTGGCAGCCCGAGGTCTGAGTTCCAAGGACAAGTA

TGTGAAAGGCCTGATTGAGGGCAAATCGGATCCCTACGCGCTCGTCCGTGTGGGCA
CCCAGACGTTCTGCAGCCGTGTCATAGATGAGGAGCTCAACCCTCACTGGGGAGAG
ACATATGAG
phase 0
ac122159.30 53136-53312

GTGATAGTCCACGAGGTTCCAGGACAGGAGATCGAGGTGGAGGTATTTGACAAAGA
TCCAGATAAAGATGATTTTCTGGGAAG
phase +2
ac122159.30 53601-53683

AATGAAGCTGGACGTGGGGAAGGTATTACAGGCTGGAGTCCTGGATAAT
phase 0
ac122159.30 54119-54167

TGGTACCCTCTGCAAGGCGGGCAAGGCCAAGTTCACTTGAGACTAGAATGGCTATC
ACTCCTGCCAGATGCAGAGAAGCTGGATCAG
phase 0
ac122159.30 54271-54357

GTCCTGCAGTGGAATCGGGGCATCACTTCTCGGCCAGAGCCCCCATCGGCTGCCAT
CCTCGTTGTCTACTTGGACCGAGCCCAGGACCTTCCT
phase 0
ac122159.30 54436-54528

CTGAAGAAAGGCAACAAGGAACCCAACCCCATGGTGCAACTATCAGTTCAGGATGT
GACCCGGGAGAGCAAG
phase 0
ac122159.30 54743-54814

GCTACCTACAGCACCAACAGCCCGGTGTGGGAAGAGGCTTTCCGGTTTTTCCTGCA
GGACCCTCGAAGCCAGGAGCTTGATGTACAG
phase 0
ac122159.30 54942-55028

GTGAAGGACGACTCCCGAGCCCTGACTTTAGGGGCCCTGACTCTCCCTCTTGCGCG
CCTGTTGACTGCCTCTGAACTCACCCTGGACCAGTGGTTCCAGCTCAGCAGCTCAG
GCCCAAACTCCAGGCTCTACATGAAACTGGTCATGCGG
phase 0
ac122159.30 56192-56341

ATCTTATACTTAGATTACTCGGAAATCCGCTTCCCCACTGTGCCTGGTGCCCAGGA
CTGGGACCGTGAGAGCCTAGAGACAGGCAGCAGTGTGGATGCCCCACCTCGGCCCT
ATCACACAACCCCTAACAGCCACTTTGGGACTGAG
phase 0
ac122159.30 56561-56707

AATGTTCTTCGGATCCATGTATTAGAAGCGCAGGACCTAATTGCCAAAGACCGTTT
CTTGGGAGGCCTGGTGAAGGGCAAATCAGACCCCTACGTCAAACTAAAGGTGGCAG
GAAAAAGTTTCCGGACCCATGTTGTTCGGGAAGATCTCAATCCCCGCTGGAATGAG
GTTTTTGAG
phase 0
ac122159.30 56826-57002

GTGATTGTCACATCAATCCCTGGCCAAGAGCTTGAGATTGAGGTCTTTGATAAGGA
CTTGGACAAGGATGACTTTTTGGGCAG
phase +2
ac122159.30 57198-57280

GTATAAAGTGAGTCTCACCACAGTGCTCAACAGTGGCTTTCTTGATGAG
phase 0
ac122159.30 57375-57423

TGGCTGACCCTGGAGGATGTCCCATCCGGGCGCCTGCACTTGCGCCTGGAGCGTCT

GACCCCTAGACCGACTGCTGCTGAGTTAGAGGAG
phase 0
ac122159.30 57508-57597

GTGCTGCAGGTGAACAGCCTGATCCAGACTCAGAAGAGTTCAGAGCTGGCAGCAGC
CCTGCTGTCCGTCTTCCTGGAGCGGGCAGAAGATCTGCCG
phase 0
ac122159.30 57716-57811

CTCCGAAAAGGTACCAAGCCTCCCAGCCCTTACGCTACTATCACCGTGGGAGAGAC
TTCCCATAAAACCAAG
phase 0
ac122159.30 58020-58091

ACTGTCTCCCAATCTTCGGCCCCTGTCTGGGAGGAGAGTGCCTCTTTTCTCATCAG
GAAGCCACACGCTGAGAGCCTGGAGCTGCAG
phase 0
ac122159.30 58184-58270

GTTCGGGGTGAAGGGACCGGCACCCTGGGCTCTGTATCCCTTCCTCTCTCTGAGCT
CCTCCAGGAGGACCAGCTCTGCCTGGACCACTGGTTTGCACTCAGTGGTCAGGGCC
AGGTGCTGATGAGAGCGCAGCTTGGG
phase 0
ac122159.30 61492-61629

ATCCTGGTATCTCAGCACTCGGGAGTGGAAGCCCACAGCCACAGCTACAGCCACAG
TCACAGCTCCTCCTCGCTCAATGACGAACCTGAGGCCTTAGGGGGACCCACTCACC
CTGCCTCCCCGGTCCTAGAGGTCAGGCATCGCCTAACACATGGTGACAG
phase +2
ac122159.30 61738-61898

TCCCTCTGAGGCTCCCGTTGGGCCCCTGGGCCAGGTGAAGCTGACAGTGTGGTACC
ACAGCGACGAACAAAAGCTGATCAGCATTATTCACAGCTGCCG
phase +2
ac122159.30 61989-62087

GGCCCTTCGACAAAATGGACGTGATCTCCCAGACCCCTATGTGTCAGTGTTGCTGC
TCCCAGACAAGAACCGAAGCACCAAGAGGAAGACCCCGCAGAAGAAGAGGACCCTC
AATCCTGAGTTCAATGAGCG
phase +2
ac122159.30 62170-62301

GTTTGAGTGGGATCTGCCCCTGGATGGGACTCTCAGGCGGAAGCTCGATGTCTCTG
TGAAGTCAAACTCCTCCTTCATGTCACGAGAGCGTGAGCTTCTGGGGAAG
phase 0
ac122159.30 62385-62490

GTGCAGCTGGATCTAGCAGAGATAGACCTTTCCCAGGGTGCAGCCCAGTG
phase +2
ac122159.30 62927-62976

GTATGACCTGATGGATGACAGAGATAAGGGCGGTTCCTAG
ac122159.30 63297-63336

FAM62B

MSSAGGEGPEAGPGRAGGRSEPEAPGSALSVDLPGLLGQLARSFALLLPVYALGYLGLSF
SWVLLALGLLAWCRRSRGLKASRLCRALALLEDEEQAVRLGVRACDLPAW**^ 0**
VHFPDTERAEWLNK**^ 0**
TVKHMWPFICQFIEKLFRETIEPAVRGANAHLSTFSFTKVDVGQQ**^ 0**
PLRVNGVKVYTENVDKRQIILDLQI**^ +2**
SFVGNCEIDLEIKRYFCRAGVKSIQ**^ 0**
IHGTMRVILEPLIGDMPLVGALSIFFLRKP**^ 0**
LLEINWTGLTNLLDIPGL**^ +2**
NGLSDTIILDIISNYLVLPNRITVPLVSEVQIAQLRFPIPK**^ 0**

GVLRIHFIEAQDLQGKDTYLKGLVKGKSDPYGIIRVGNQIFQSKVIKENLSPKWNEVYE**^ 0**
ALVYEHPGQELEIELFDEDPDKDDFLG**^ +2**
SLMIDLIEVEKERLLDE**^ 0**
WFTLDEVPKGKLHLKLEWLTLMPDAANLDK**^ 0**
VLADIRADKDQASDGLSSALLILYLDSARNLP**^ 0**
[SNPLDFNPGVLKKSAVQRALK**^ 0]**
SGKKINSNPNPLVQMSVGHKAQESK**^ 0**
IRYKTSEPVWEENFTFFIHNPRRQDLEVE**^ 0**
VKDEQHQCSLGSLRIPLSQLLTSDNMTINQRFQLSNSGPNSTLKMKIALR**^ 0**
VLHLEKQERPPDYQHSAQVKRPSVSKEGRKMPIKSQMSASPGTGGANTAPSTPVMGVDDK
PAMEEKPQPPEASPLGHRDLGRSSSSLLASPSHIAAKEPTPSIASDISLPIATQELRQRL
RQLE**^ +2**
NGTTLGQSPLGQIQLTIRHSSQRNKLIVVVHSC**^ +2**
RNLIAFSEDGSDPYVRMYLLPDKRRSGRRKTHVSKKTLNPVFDQ**^ +2**
SFDFSVSLPEVQRRTLDVAVKNSGGFLSKDKGLLGK**^ 0**
VLVVLASEELAKGWTQ**^ +2**
WYDLTEDGTRPQVIT

ATGAGTAGCGCGGGGGGCGAGGGGCCGGAGGCCGGTCCTGGCCGGGCTGGGGGCCGCTCC
GAGCCCGAGGCCCCGGGCAGCGCTCTGAGTGTGGACTTGCCGGGGCTGCTGGGGCAGCTG
GCGCGCAGCTTCGCGCTGCTGCTGCCGGTGTACGCCCTGGGCTACCTGGGGCTGAGCTTC
AGTTGGGTGCTGCTGGCGCTCGGGCTGCTCGCCTGGTGCCGCCGGAGCCGCGGCCTCAAG
GCCAGCCGCCTGTGCCGCGCGCTGGCGCTGTTGGAGGACGAGGAGCAAGCTGTGCGCCTG
GGAGTGCGCGCCTGCGACCTGCCCGCCTGG
phase 0
ac104832.8 156136-156465

GTTCATTTTCCAGACACTGAAAGAGCAGAATGGCTAAATAAG
phase 0
ct030173.7 23584-23625

ACTGTAAAGCACATGTGGCCCTTTATTTGTCAGTTTATCGAGAAGCTCTTTCGAGAAACC
ATAGAGCCAGCTGTGCGTGGAGCAAATGCTCACCTCAGCACCTTCAGCTTCACAAAAGTG
GATGTGGGTCAGCAG
phase 0
ct030173.7 24558-24692

CCCCTGAGAGTCAATGGTGTTAAGGTTTATACTGAAAATGTAGACAAAAGACAAATTATT
CTGGACCTTCAGATTAG
phase +2
ct030173.7 26881-26957

TTTTGTAGGAAATTGTGAGATTGATTTGGAGATCAAAAGATATTTTTGTAGAGCTGGTGT
GAAAAGTATTCAG
phase 0
ct030173.7 29594-29666

ATCCATGGGACAATGCGGGTGATACTGGAGCCCCTGATTGGAGACATGCCTTTAGTTGGA
GCTTTGTCCATCTTCTTCCTTAGGAAACCA
phase 0
ct030173.7 29875-29964

CTTTTAGAGATTAACTGGACAGGGCTGACTAACCTTCTGGACATCCCTGGACTGAA
phase +2
ct030173.7 39955-40010

TGGTTTATCTGATACTATCATTTTGGATATAATATCAAACTATCTAGTGCTTCCCAATCG
AATCACCGTTCCTCTTGTCAGTGAAGTTCAAATAGCTCAGCTGCGGTTTCCCATACCAAAG
phase 0
ct030173.7 45301-45421

GGTGTCCTAAGGATTCACTTTATTGAAGCTCAAGATCTTCAGGGCAAAGACACCTACCTT
AAAGGCCTTGTCAAGGGAAAATCAGACCCCTATGGAATTATCCGGGTGGGCAACCAAATC
TTCCAGAGCAAAGTCATCAAAGAGAACCTTAGTCCAAAATGGAATGAAGTGTATGAG
phase 0
ct030173.7 47830-48006

GCTTTAGTGTATGAACACCCTGGACAAGAATTGGAGATTGAACTGTTTGATGAAGATCCA
GACAAGGATGATTTCTTAGGAAG
phase +2
ct030173.7 49192-49274

TCTTATGATTGATCTTATTGAAGTTGAGAAGGAGCGCCTTTTAGATGAA
phase 0
ct030173.7 52258-52306

TGGTTCACTCTGGACGAGGTTCCCAAAGGAAAGCTGCATTTGAAGTTAGAGTGGCTCACA
CTGATGCCTGATGCTGCAAACCTTGACAAG
phase 0
ct030173.7 53480-53569

GTGCTGGCAGACATCAGAGCTGACAAGGACCAAGCCAGTGATGGCCTTTCATCTGCACTG
CTGATCTTGTATTTGGATTCTGCAAGGAACCTTCCG
phase 0
ct030173.7 53973-54068

often skipped
AGTAACCCATTAGACTTTAACCCCGGTGTCTTGAAGAAGTCTGCAGTTCAGAGAGCTTTGAAG
phase 0
ct030173.7 59581-59643

TCAGGGAAGAAAATAAACAGCAACCCAAATCCTCTTGTCCAGATGTCAGTCGGTCACAAG
GCTCAAGAGAGTAAG
phase 0
ct030173.7 62866-62940

ATTCGATACAAAACTAGTGAGCCTGTATGGGAGGAAAACTTCACTTTCTTCATTCATAAC
CCCAGGCGCCAGGACCTTGAGGTTGAG
phase 0
ct030173.7 63602-63688

GTTAAAGATGAGCAGCATCAGTGTTCTCTGGGGAGCCTGAGGATCCCACTCAGTCAACTG
CTTACAAGTGACAACATGACTATAAACCAGCGGTTCCAGCTTAGCAACTCGGGTCCAAAC
AGCACCTTAAAAATGAAGATTGCCCTCCGG
phase 0
ct030173.7 69149-69298

GTCCTCCATCTTGAAAAGCAAGAAAGGCCTCCAGACTACCAGCATTCAGCTCAAGTGAAA
CGGCCCTCTGTCTCCAAAGAAGGGAGGAAAATGCCTATCAAATCTCAGATGTCTGCGTCG
CCAGGCACCGGTGGTGCCAACACTGCTCCGTCAACACCAGTCATGGGAGTCGATGATAAG
CCTGCCATGGAGGAGAAGCCCCAGCCCCCTGAGGCCAGCCCTCTCGGGCATCGTGACCTA
GGCAGGAGCTCCTCCAGCCTCTTGGCCTCTCCAAGCCACATCGCAGCCAAGGAACCCACC
CCAAGCATTGCCTCAGACATATCACTGCCCATTGCCACTCAGGAGCTGAGACAAAGGCTA
CGTCAACTTGAAAA
phase +2
ct030173.7 71309-71682

TGGGACAACCCTGGGACAATCTCCTCTGGGGCAGATCCAGCTGACTATCCGGCACAGCTC
CCAAAGGAATAAGCTCATCGTGGTTGTGCACTCCTGCAG
phase +2
ct030173.7 72684-72782

AAACCTCATTGCCTTCTCGGAAGATGGCTCTGACCCATATGTCCGCATGTATTTATTACC
AGACAAGAGACGCTCAGGAAGAAGGAAAACACATGTATCAAAGAAGACATTGAACCCTGT
GTTTGATCAGAG
phase +2
ct030173.7 73462-73593

CTTCGACTTCAGTGTTTCGCTGCCTGAGGTGCAAAGGAGAACATTGGATGTGGCTGTGAA
GAACAGTGGTGGCTTCCTGTCCAAAGATAAAGGGCTTCTTGGCAAA

phase 0
ct030173.7 74490-74595

GTGCTGGTTGTTCTGGCATCTGAAGAACTCGCCAAGGGCTGGACCCAGTG
phase +2
ct030173.7 75766-75815

GTATGACCTCACAGAAGATGGGACAAGGCCACAGGTGATAACG<u>TAG</u>
ct030173.7 75925-75970

FAM62C

MQPEEPCAPSAPGGPDVPERGQRSRDPGPRLSGQLLPELYSFVARVLFYLAPVYLAGYLG
LSVTWLLLGALLWMWWRRNRRGKLGRLEAAFEFLEHEREFISRELRGQHLPAW**^ 0**
IHFPDVERVEWANK**^ 0**
IIIQIWPYLSMIMENKIREKLEPKIREKSIHLRTFTFTKLYFGQK**^ 0**
CPKVNGVKVHTDKRNRRKVTLDLQI**^ +2**
CYIGDCEISVELQKIRGGVSGVQ**^ 0**
LQGTLRVILEPLLVDKPFIGAVTVFFLQKP**^ 0**
HLQINWTGLTNLLDMPGI**^ +2**
NELSDSLLEDLIAAHLVLPNRVTVPVKKGLDVTNLRVPLPC**^ 0**
GVIRVHLLEAKKLAQKDNFLGLGGKSDPYAKVSIGLQHCRSRTIYKNLNPTWNEVFE**^ 0**
FMVYEVPGQDLEVDLYDEDTDKDDFLG**^ +2**
SLQICLGDVMKNRVVDE**^ 0**
WFALNDTTSGRLHLRLEWLSLLTDQEALTE**^ 0**
NDSGLSTAILVVFLENACNLP**^ 0**
RNPFDYLNGEYRAKKLSRFVK**^ 0**
NKASRDPSSYVKLTVGKKTFTSK**^ 0**
TCPHSKDPVWSQVFSFFVHSVAAEQLCLK**^ 0**
VLDDELECALGVLEFPLCRILPCADLTLEQCFQLDHSGLDSLISMRLVLR**^ 0**
FLRVEGRELGSPYTGPDALKKGPLFIKKVATNQGCKAPPLNEGLADVTSTSNPASYIKGA
SKSIDNISAATTDPEPMPEPQGPGPEPKGKDSARGLCESPGKKKNPATTFLTVPGLHSPG
PIKSPRPMSRPAFPFAWPLTRVAPSMSSLNSLASSCFDLTDVSLNT**^ +2**
EAGDSRQGRLGEIQLTVRYVCLRHCLRVLVNGC**^ +2**
RNLTPCTSSGADPYVRIYLLPERRWASRKKTSVKQKTLEPLFDE**^ +2**
TFEFFVPMGEVQKRSLDVAVKNSRPLGSHRRKELGK**^ 0**
VLIDLSKQDLIKGFSQ**^ +2**
WYELTADGQPRS

<u>ATG</u>CAGCCGGAGGAGCCCTGCGCCCCGAGCGCGCCCGGCGGTCCTGACGTCCCGGAGAGA
GGCCAGCGCTCGCGGGACCCCGGACCGCGCCTGTCGGGCCAGCTACTGCCCGAGCTCTAC
AGCTTCGTGGCGCGCGTCCTTTTCTACCTGGCACCGGTCTACCTGGCGGGCTACCTGGGG
CTCAGCGTCACCTGGCTGCTGCTTGGCGCCCTGCTCTGGATGTGGTGGCGCCGGAACCGC
CGCGGGAAGCTGGGGCGCTTGGAGGCCGCCTTCGAATTCCTGGAGCACGAGCGCGAGTTC
ATCAGCCGTGAGCTGCGGGGCCAGCATTTGCCAGCCTGG
phase 0
ac157994.2 119426-119764

ATACATTTCCCAGATGTGGAGCGTGTGGAGTGGGCCAATAAG
phase 0
ac157994.2 139006-139047

ATCATCATACAGATCTGGCCCTACCTAAGCATGATCATGGAGAACAAGATCCGGGAGAAG
CTGGAGCCCAAGATCCGGGAGAAGAGCATCCACCTCAGAACCTTCACCTTCACCAAGCTC
TACTTTGGGCAGAAG
phase 0
ac157994.2 141616-141750

TGTCCCAAGGTGAACGGCGTCAAGGTACACACGGACAAACGCAACCGGAGGAAAGTGACC
CTGGACCTGCAGATCTG
phase +2
ac157994.2 144933-145009

CTACATCGGGGACTGTGAGATCAGCGTGGAACTTCAGAAGATCCGGGGCGGTGTGAGCGG
CGTCCAG
phase 0

ac157994.2 146390-146456

TTGCAGGGGACCCTGCGTGTCATTCTGGAGCCCCTGCTGGTGGACAAGCCCTTCATAGGA
GCTGTGACAGTGTTCTTCCTTCAAAAGCCG
phase 0
ac157994.2 147025-147114

CACCTGCAGATTAACTGGACAGGCTTGACCAACCTGCTGGACATGCCGGGGATCAA
phase +2
ac157994.2 148004-148059

TGAGTTATCAGACAGCCTGCTGGAGGACCTCATTGCTGCCCACCTGGTGCTGCCCAACCG
TGTGACTGTGCCTGTGAAGAAGGGGCTCGATGTAACCAACCTGCGCGTCCCTCTCCCCTGT
phase 0
ac157994.2 149796-149916

GGTGTGATCCGAGTCCATTTGCTGGAGGCCAAGAAGCTGGCCCAGAAGGACAACTTCCTA
GGGCTCGGAGGCAAGTCAGACCCCTACGCCAAGGTGAGCATTGGCTTGCAGCATTGCCGG
AGCAGAACCATCTACAAGAACCTGAACCCCACCTGGAATGAAGTATTTGAG
phase 0
ac157994.2 152748-152918

TTTATGGTGTACGAGGTACCTGGGCAGGACCTGGAGGTGGACCTGTATGATGAGGATACT
GACAAGGATGACTTCCTGGGCAG
phase +2
ac157994.2 153575-153657

CCTGCAGATCTGCCTTGGAGATGTCATGAAGAACAGAGTGGTGGATGAG
phase 0
ac157994.2 155170-155218

TGGTTTGCCCTGAATGACACGACCAGTGGGCGGCTGCACCTGCGGCTGGAGTGGCTCTCA
CTACTCACTGACCAGGAAGCGCTGACGGAG
phase 0
ac157994.2 155628-155717

AATGATAGTGGCCTCTCCACTGCCATCCTCGTGGTCTTCTTAGAGAATGCCTGCAACCTGCCG
phase 0
ac157994.2 156226-156288

AGAAACCCTTTTGACTATCTGAATGGTGAATATCGAGCCAAAAAACTCTCCAGGTTTGTCAAA
phase 0
ac157994.2 156371-156433

AATAAGGCCAGCAGAGACCCTTCTTCATACGTCAAGCTCACTGTAGGCAAGAAGACCTTT
ACAAGTAAG
phase 0
ac157994.2 156913-156981

ACTTGTCCCCACAGCAAGGACCCTGTGTGGAGCCAGGTGTTCTCCTTCTTTGTGCACAGC
GTGGCAGCCGAGCAGCTCTGCCTGAAG
phase 0
ac157994.2 157474-157560

GTGCTGGACGATGAGCTAGAGTGTGCTCTGGGAGTGCTGGAGTTCCCCCTGTGCCGGATC
CTCCCCTGTGCTGACCTCACCCTCGAACAGTGCTTCCAGCTGGATCACTCAGGCCTGGAC
AGCCTCATCTCCATGAGGCTGGTGCTTCGG
phase 0
ac157994.2 158163-158312

TTCCTACGTGTGGAGGGACGAGAGCTGGGAAGCCCATACACTGGACCCGACGCTCTAAAG
AAAGGTCCTCTGTTCATCAAGAAGGTGGCCACCAACCAGGGTTGTAAAGCCCCACCCCTA
AACGAAGGCCTTGCAGATGTGACAAGCACCTCGAACCCTGCCTCTTACATCAAAGGAGCC
TCCAAGAGCATTGACAATATCAGCGCAGCCACCACAGACCCTGAACCTATGCCTGAGCCC

CAGGGGCCAGGCCCGGAGCCCAAAGGCAAGGACAGTGCCAGAGGGCTCTGTGAGTCCCCT
GGAAAGAAGAAGAACCCAGCTACCACCTTCCTGACTGTCCCAGGTCTCCACTCTCCAGGG
CCCATCAAGTCACCCAGACCCATGAGCCGCCCTGCCTTCCCATTCGCATGGCCACTCACG
AGGGTGGCTCCCAGCATGTCCTCACTCAACTCCCTGGCCTCTTCCTGCTTTGACCTGACA
GATGTCAGCCTCAACACTGA
phase +2
ac157994.2 159428-159927

AGCTGGGGATTCCAGGCAGGGACGGCTGGGTGAGATTCAGCTCACAGTGCGCTATGTGTG
TCTCCGACACTGTCTCAGAGTGCTGGTCAACGGCTGCAG
phase +2
ac157994.2 160511-160609

AAACTTAACACCATGCACCAGCAGTGGAGCTGACCCCTACGTCCGCATCTACCTGCTGCC
AGAGAGAAGATGGGCGAGTCGGAAGAAGACCTCAGTAAAACAGAAGACCCTGGAGCCCCT
ATTCGATGAGAC
phase +2
ac157994.2 161038-161169

GTTTGAGTTTTTTGTCCCCATGGGAGAAGTGCAGAAGAGATCACTGGATGTCGCAGTGAA
AAACAGCAGGCCTCTCGGCTCACACAGAAGGAAGGAGTTGGGGAAA
phase 0
ac157994.2 162523-162628

GTGCTGATTGACTTATCAAAGCAAGATCTGATTAAGGGCTTCTCACAGTG
phase +2
ac157994.2 165820-165869

GTATGAACTGACTGCAGATGGACAGCCCAGAAGCTGA
ac157994.2 166138-166174

Homo sapiens *FAM62* genes

Eukaryota; Metazoa; Chordata; Craniata; Vertebrata; Euteleostomi; Mammalia; Eutheria; Euarchontoglires; Primates; Haplorrhini; Catarrhini; Hominidae; Homo.

FAM62A
Gene 120 Craxton, M. BMC Genomics. 2004 Jul 6;5(1):43

MERSPGEGPSPSPMDQPSAPSDPTDQPPAAHAKPDPGSGGQPAGPGAAGEALAVLTSFGR
RLLVLIPVYLAGAVGLSVGFVLFGLALYLGWRRVRDEKERSLRAARQLLDDEEQLTAKTL
YMSHRELPAW**^ 0**
VSFPDVEKAEWLNK**^ 0**
IVAQVWPFLGQYMEKLLAETVAPAVRGSNPHLQTFTFTRVELGEK**^ 0**
PLRIIGVKVHPGQRKEQILLDLNI**^ +2**
SYVGDVQIDVEVKKYFCKAGVKGMQ**^ 0**
LHGVLRVILEPLIGDLPFVGAVSMFFIRRP**^ 0**
TLDINWTGMTNLLDIPGL**^ +2**
SSLSDTMIMDSIAAFLVLPNRLLVPLVPDLQDVAQLRSPLPR**^ 0**
GIIRIHLLAARGLSSKDKYVKGLIEGKSDPYALVRLGTQTFCSRVIDEELNPQWGETYE**^ 0**
VMVHEVPGQEIEVEVFDKDPDKDDFLG**^ +2**
RMKLDVGKVLQASVLDD**^ 0**
WFPLQGGQGQVHLRLEWLSLLSDAEKLEQ**^ 0**
VLQWNWGVSSRPDPPSAAILVVYLDRAQDLP**^ 0**
[LKKGNKEPNPMVQLSIQDVTQESK**^ 0]**
AVYSTNCPVWEEAFRFFLQDPQSQELDVQ**^ 0**
VKDDSRALTLGALTLPLARLLTAPELILDQWFQLSSSGPNSRLYMKLVMR**^ 0**
ILYLDSSEICFPTVPGCPGAWDVDSENPQRGSSVDAPPRPCHTTPDSQFGTE**^ 0**
HVLRIHVLEAQDLIAKDRFLGGLVKGKSDPYVKLKLAGRSFRSHVVREDLNPRWNEVFE**^ 0**
VIVTSVPGQELEVEVFDKDLDKDDFLG**^ +2**
RCKVRLTTVLNSGFLDE**^ 0**
WLTLEDVPSGRLHLRLERLTPRPTAAELEE**^ 0**
VLQVNSLIQTQKSAELAAALLSIYMERAEDLP**^ 0**
LRKGTKHLSPYATLTVGDSSHKTK**^ 0**
TISQTSAPVWDESASFLIRKPHTESLELQ**^ 0**
VRGEGTGVLGSLSLPLSELLVADQLCLDRWFTLSSGQGQVLLRAQLG**^ 0**
ILVSQHSGVEAHSHSYSHSSSSLSEEPELSGGPPHITSSAPELRQRLTHVD**^ +2**

SPLEAPAGPLGQVKLTLWYYSEERKLVSIVHGC**^ +2**
RSLRQNGRDPPDPYVSLLLLPDKNRGTKRRTSQKKRTLSPEFNE**^ +2**
RFEWELPLDEAQRRKLDVSVKSNSSFMSRERELLGK**^ 0**
VQLDLAETDLSQGVAR**^ +2**
WYDLMDNKDKGSS

FAM62B
Gene 122 Craxton, M. BMC Genomics. 2004 Jul 6;5(1):43

MTPPSRAEAGVRRSRVPSEGRWRGAEPPGISASTQPASAGRAARHCGAMSGARGEGPEAG
AGGAGGRAAPENPGGVLSVELPGLLAQLARSFALLLPVYALGYLGLSFSWVLLALALLAW
CRRSRGLKALRLCRALALLEDEERVVRLGVRACDLPAW**^ 0**
VHFPDTERAEWLNK**^ 0**
TVKHMWPFICQFIEKLFRETIEPAVRGANTHLSTFSFTKVDVGQQ**^ 0**
PLRINGVKVYTENVDKRQIILDLQI**^ +2**
SFVGNCEIDLEIKRYFCRAGVKSIQ**^ 0**
IHGTMRVILEPLIGDMPLVGALSIFFLRKP**^ 0**
LLEINWTGLTNLLDVPGL**^ +2**
NGLSDTIILDIISNYLVLPNRITVPLVSEVQIAQLRFPVPK**^ 0**
GVLRIHFIEAQDLQGKDTYLKGLVKGKSDPYGIIRVGNQIFQSRVIKENLSPKWNEVYE**^ 0**
ALVYEHPGQELEIELFDEDPDKDDFLG**^ +2**
SLMIDLIEVEKERLLDE**^ 0**
WFTLDEVPKGKLHLRLEWLTLMPNASNLDK**^ 0**
VLTDIKADKDQANDGLSSALLILYLDSARNLP**^ 0**
[SNPLEFNPDVLKKTAVQRALK**^ 0]**
SGKKISSNPNPVVQMSVGHKAQESK**^ 0**
IRYKTNEPVWEENFTFFIHNPKRQDLEVE**^ 0**
VRDEQHQCSLGNLKVPLSQLLTSEDMTVSQRFQLSNSGPNSTIKMKIALR**^ 0**
VLHLEKRERPPDHQHSAQVKRPSVSKEGRKTSIKSHMSGSPGPGGSNTAPSTPVIGGSDK
PGMEEKAQPPEAGPQGLHDLGRSSSSLLASPGHISVKEPTPSIASDISLPIATQELRQRL
RQLE**^ +2**
NGTTLGQSPLGQIQLTIRHSSQRNKLIVVVHAC**^ +2**
RNLIAFSEDGSDPYVRMYLLPDKRRSGRRKTHVSKKTLNPVFDQ**^ +2**
SFDFSVSLPEVQRRTLDVAVKNSGGFLSKDKGLLGK**^ 0**
VLVALASEELAKGWTQ**^ +2**
WYDLTEDGTRPQAMT

FAM62C
Gene 121 Craxton, M. BMC Genomics. 2004 Jul 6;5(1):43

MRAEEPCAPGAPSALGAQRTPGPELRLSSQLLPELCTFVVRVLFYLGPVYLAGYLGLSIT
WLLLGALLWMWWRRNRRGKLGRLAAAFEFLDNEREFISRELRGQHLPAW**^ 0**
IHFPDVERVEWANK**^ 0**
IISQTWPYLSMIMESKFREKLEPKIREKSIHLRTFTFTKLYFGQK**^ 0**
CPRVNGVKAHTNTCNRRRVTVDLQI**^ +2**
CYIGDCEISVELQKIQAGVNGIQ**^ 0**
LQGTLRVILEPLLVDKPFVGAVTVFFLQKP**^ 0**
HLQINWTGLTNLLDAPGI**^ +2**
NDVSDSLLEDLIATHLVLPNRVTVPVKKGLDLTNLRFPLPC**^ 0**
GVIRVHLLEAEQLAQKDNFLGLRGKSDPYAKVSIGLQHFRSRTIYRNLNPTWNEVFE**^ 0**
FMVYEVPGQDLEVDLYDEDTDRDDFLG**^ +2**
SLQICLGDVMTNRVVDE**^ 0**
WFVLNDTTSGRLHLRLEWLSLLTDQEVLTE**^ 0**
DHGGLSTAILVVFLESACNLP**^ 0**
[RNPFDYLNGEYRAKKLSRFAR**^ 0]**
NKVSKDPSSYVKLSVGKKTHTSK**^ 0**
TCPHNKDPVWSQVFSFFVHNVATERLHLK**^ 0**
VLDDDQECALGMLEVPLCQILPYADLTLEQRFQLDHSGLDSLISMRLVLR**^ 0**
FLQVEERELGSPYTGPEALKKGPLLIKKVATNQGPKAQPQEEGPTDLPCPPDPASDTKDV
SRSTTTTTSATTVATEPTSQETGPEPKGKDSAKRFCEPIGEKKSPATIFLTVPGPHSPGP
IKSPRPMKCPASPFAWPPKRLAPSMSSLNSLASSCFDLADISLNI**^ +2**
EGGDLRRRQLGEIQLTVRYVCLRRCLSVLINGC**^ +2**
RNLTPCTSSGADPYVRVYLLPERKWACRKKTSVKRKTLEPLFDE**^ +2**
TFEFFVPMEEVKKRSLDVAVKNSRPLGSHRRKELGK**^ 0**
VLIDLSKEDLIKGFSQ**^ +2**
WYELTPNGQPRS
