# Supplementary material for: Evolutionary genomics of plant genes encoding N-terminal-TM-C2 domain proteins and the similar FAM62 genes and synaptotagmin genes of metazoans
Source: BMC Genomics. 2007 Jul 31;8:259. doi: 10.1186/1471-2164-8-259 (PMC1976326; doi:10.1186/1471-2164-8-259)
Supplement: Additional file 4 — New Syt sequences, Rabphilin and Doc2 sequences. [file 1471-2164-8-259-S4.pdf]

Additional file 4 - new *Syt* sequences
Taxonomy from NCBI

translation start/stop codons are underlined
^ indicates an intron
complementary strand nucleotide positions indicated within parentheses

Figure 10 *Syt genes*

Caenorhabditis (1) Gene 1 Craxton, M. BMC Genomics. 2004 Jul 6;5(1):43

Caenorhabditis (2) Gene 2 Craxton, M. BMC Genomics. 2004 Jul 6;5(1):43

Caenorhabditis (3) Gene 3 Craxton, M. BMC Genomics. 2004 Jul 6;5(1):43

Caenorhabditis *Sytα* Gene 5 Craxton, M. BMC Genomics. 2004 Jul 6;5(1):43

Caenorhabditis *Syt1* Gene 4 Craxton, M. BMC Genomics. 2004 Jul 6;5(1):43

Caenorhabditis *Syt4* Gene 7 Craxton, M. BMC Genomics. 2004 Jul 6;5(1):43

Caenorhabditis *Syt7* Gene 6 Craxton, M. BMC Genomics. 2004 Jul 6;5(1):43

Drosophila *Sytα* Gene 16 Craxton, M. BMC Genomics. 2004 Jul 6;5(1):43

Drosophila *Syt1* Gene 13 Craxton, M. BMC Genomics. 2004 Jul 6;5(1):43

Drosophila *Syt4* Gene 14 Craxton, M. BMC Genomics. 2004 Jul 6;5(1):43

Drosophila *Syt7* Gene 15 Craxton, M. BMC Genomics. 2004 Jul 6;5(1):43

Drosophila *Syt12* Gene 18 Craxton, M. BMC Genomics. 2004 Jul 6;5(1):43

Drosophila *Syt13* Gene 17 Craxton, M. BMC Genomics. 2004 Jul 6;5(1):43

Drosophila *Syt16* Gene 19 Craxton, M. BMC Genomics. 2004 Jul 6;5(1):43

Anopheles *Sytα* Gene 10 Craxton, M. BMC Genomics. 2004 Jul 6;5(1):43

Anopheles *Syt1* Gene 8 Craxton, M. BMC Genomics. 2004 Jul 6;5(1):43

Anopheles *Syt4* Gene 9 Craxton, M. BMC Genomics. 2004 Jul 6;5(1):43

Anopheles *Syt13* Gene 11 Craxton, M. BMC Genomics. 2004 Jul 6;5(1):43

Anopheles *Syt16* Gene 12 Craxton, M. BMC Genomics. 2004 Jul 6;5(1):43

Ciona *Sytα*

Ciona intestinalis synaptotagmin alpha

Eukaryota; Metazoa; Chordata; Urochordata; Ascidiacea; Enterogona; Phlebobranchia; Cionidae; Ciona.

MLMYCIQCIVVVFFALFLVSLTACIKTYKR**^ +2**
SLNVAPGGQNLNRRRSSMQIPIDLRQIDPKLYKVSLSADG**^ +2**
VDSTEAEADTPMIHFDVSYNGDLEVLNVKLIQ**^ 0**
ARNLATQDFSGTSDPYCTVALVPGFNPRRSKVHKKTSNPEFGESFVFSVSSDNLE**^ 0**
DKVLQVKTYDFDQFSRDECTGVMELNLKEIDFVMTPNIDLWRKMKFPDDHE**^ 0**
TSETFGDLMLALSYLRSAEKLTVAVQKARNLKEPESRKGLP**^ +1**
DPYVKVALYKGKQRIKKKKTDTIHSTCNPVFNQALTFSVPFDTLQAADTKLICHVVHDFK
LGYNEDLGQVEIGP**^ +1**
NCMDSEERRHWSEMMAQHINRPIARWHHLKQPSPPTQRLSTDENLPSSSGRRKSFAVPEA
LQLHGLADVSRRSFS

<u>ATG</u>CTCATGTATTGCATACAATGTATAGTTGTTGTATTTTTCGCTTTGTTTCTCGTAAGT
CTCACCGCGTGTATTAAAACTTACAAACGAAG
phase +2
aabs01000021.1 207788-207879

TTTAAACGTGGCACCGGGAGGTCAGAACTTGAATCGTCGGCGAAGCTCAATGCAGATCCC
GATTGATTTGCGGCAAATTGACCCCAAGCTATACAAGGTAAGCCTTTCAGCGGATGG
phase +2
aabs01000021.1 208645-208761

TGTGGACTCGACAGAAGCAGAGGCAGATACGCCAATGATTCATTTTGATGTTTCATACAA
TGGCGACCTAGAAGTATTGAACGTTAAACTTATCCAG
phase 0
aabs01000021.1 209437-209533

GCCCGGAATCTTGCCACTCAAGATTTCAGCGGAACTTCCGACCCTTACTGTACCGTTGCA
CTGGTGCCCGGGTTCAATCCACGGAGGAGCAAAGTTCATAAAAAGACATCGAACCCGGAA
TTTGGAGAAAGCTTTGTCTTTAGTGTTTCAAGCGACAATTTGGAG
phase 0
aabs01000021.1 209999-210163

GATAAAGTGCTTCAGGTGAAAACGTACGATTTCGACCAGTTTTCCAGAGACGAATGCACT
GGTGTTATGGAACTTAACCTAAAAGAAATTGATTTTGTGATGACACCCAACATTGACCTC
TGGCGGAAAATGAAATTTCCCGACGACCACGAA
phase 0
aabs01000021.1 210378-210530

ACCAGCGAAACGTTCGGAGACTTAATGCTTGCCTTGTCATATCTCCGCAGTGCTGAGAAA
CTCACTGTTGCTGTCCAGAAAGCGAGAAATTTAAAAGAACCCGAGTCAAGAAAGGGATTA
CCAG
phase +1
aabs01000021.1 210795-210918

ATCCATACGTTAAGGTCGCTCTTTACAAAGGGAAGCAACGAATAAAGAAAAAGAAAACGG
ACACAATCCATTCAACGTGTAACCCCGTTTTTAACCAAGCTCTCACGTTTAGTGTTCCAT
TCGACACTCTACAAGCGGCTGATACGAAGCTAATTTGTCACGTGGTGCATGATTTTAAAC
TCGGTTACAACGAAGACTTAGGACAAGTGGAAATAGGACCAA
phase +1
aabs01000021.1 211386-211607

ACTGCATGGACAGCGAGGAACGCCGCCATTGGTCAGAAATGATGGCGCAACATATCAACC
GACCAATAGCAAGATGGCACCACTTAAAACAACCCAGCCCTCCTACACAACGACTCTCAA
CCGACGAAAACCTCCCCAGTTCATCGGGTCGAAGGAAATCCTTTGCGGTCCCGGAGGCTC
TTCAGTTACATGGGCTAGCGGATGTTAGTCGAAGATCCTTTAGT<u>TAG</u>
aabs01000021.1 211866-212092

Ciona *Syt1* Gene 20 Craxton, M. BMC Genomics. 2004 Jul 6;5(1):43

Ciona *Syt7* Gene 21 Craxton, M. BMC Genomics. 2004 Jul 6;5(1):43

Ciona *Syt15* Gene 22 Craxton, M. BMC Genomics. 2004 Jul 6;5(1):43

Ciona *Syt16* Gene 23 Craxton, M. BMC Genomics. 2004 Jul 6;5(1):43

Strongylocentrotus *Syt1*

Strongylocentrotus purpuratus synaptotagmin 1

Eukaryota; Metazoa; Echinodermata; Eleutherozoa; Echinozoa; Echinoidea; Euechinoidea; Echinacea; Echinoida; Strongylocentrotidae; Strongylocentrotus.

MGDQEVSQAAVPNESAAAPPAPTEAAADGGGGGGGGIGGAFANAGTNMKNKISDVMDKIP**^ +1**
LPTWAVVAIAIVAGLILLCCCFCICKKCCCKKRKKKEGKKGLKGAVDLKSVQMLGNSYKEK**^ 0**
PDVDDLDNGGDEEGDTDSVKSEIKLGKLQFSLDYDFQEGK**^ 0**
LNVGVMQASELPGMDFSGTSDPYVKVYLMPDKKKKYETKVHRKTLNPVFNETFTFKV**^ 0**
PYSEVSSKTLVFAIYDFDRFSRHDIIGEVKVKLSQVDLGSVVEEWRDLQSAEVPGGE**^ 0**
GKSELGDICFSLRYVPTAGKLTVVILEAKNLKKMDVGGLS**^ +1**
DPYVKISLYMNNKRMKKKKTTIKKRTLNPYYNESFGFEVPFEQIQ**^ 0**
KVTLVVTVVDYDRMGSSEPIGKVVLGCNATGAGLRHWSDMLASPRRPIAQWHTLQEPEEGK

ATGGGCGACCAAGAGGTCTCACAAGCAGCCGTCCCTAATGAGTCTGCAGCTGCACCTCCC
GCCCCAACAGAGGCTGCCGCGGATGGCGGGGGAGGAGGAGG[AGGAGG] not in cdnas
CATTGGAGGTGCCTTTGCAAATGCGGGCACCAACATGAAGAACAAGATAAGCGACGTGAT
GGATAAAATTCCTC
phase +1
ac184654.1 131228-131408 includes the repeat
aagj01172256.1 (1547-1373) excludes the 2 codon repeat
aagj01185991.1 3561-3741 includes the repeat
aagj01280211.1 (683-503) includes the repeat
aagj01140037.1 (750-571) includes the repeat

TTCCAACATGGGCTGTGGTAGCCATTGCCATTGTGGCAGGCCTTATACTCCTCTGTTGCT
GCTTCTGTATCTGTAAAAAATGTTGCTGTAAGAAGAGGAAAAAGAAGGAAGGCAAGAAAG
GTCTAAAGGGGGCCGTCGACCTGAAGAGCGTCCAGATGCTGGGCAACAGTTATAAAGAGAAG
phase 0
ac184654.1 132608-132789

CCTGATGTCGATGATCTAGATAACGGAGGGGACGAAGAAGGCGATACCGACTCCGTTAAG
TCTGAGATCAAGCTCGGAAAGCTACAGTTCTCGCTGGATTATGACTTCCAAGAAGGAAAG
phase 0
ac184654.1 55478-55597

CTCAATGTGGGTGTCATGCAAGCATCGGAGCTACCAGGGATGGACTTCTCAGGGACATCG
GATCCCTACGTCAAGGTTTACCTCATGCCAGACAAGAAGAAGAAGTACGAAACCAAAGTC
CACAGAAAGACCCTCAACCCAGTCTTCAATGAGACCTTCACATTCAAG
phase 0
ac184654.1 154456-154623

GTGCCATACAGTGAAGTGTCGAGTAAGACGCTGGTGTTTGCCATTTACGATTTTGACCGA
TTCTCCCGCCATGACATCATAGGGGAGGTCAAGGTCAAACTAAGCCAGGTTGACCTGGGC
AGTGTCGTTGAAGAGTGGCGCGACCTCCAGAGCGCTGAGGTCCCAGGAGGAGAG
phase 0
ac184654.1 154860-155033

GGCAAGTCAGAGCTGGGAGACATTTGTTTCTCCTTGCGTTACGTCCCCACTGCAGGCAAG
CTGACCGTGGTCATCTTAGAGGCCAAGAACCTCAAGAAAATGGACGTCGGTGGACTATCAG
phase +1
ac184654.1 157010-157130

ag?]
ATCCATATGTGAAGATTTCCCTCTACATGAACAACAAGAGGATGAAGAAAAAGAAGACCA
CCATTAAGAAGAGGACACTGAATCCTTACTACAACGAATCCTTTGGCTTTGAGGTGCCAT
TCGAACAGATTCAG
phase 0 gc donor
ac184654.1 269-402

AAAGTGACGTTGGTGGTGACTGTAGTGGACTATGATCGCATGGGCAGCAGCGAACCTATC
GGGAAAGTAGTACTTGGATGCAATGCCACAGGGGCGGGACTCCGTCACTGGAGCGACATG
CTGGCGTCGCCACGGCGACCAATCGCCCAATGGCATACACTCCAAGAACCCGAGGAAGGG
AAATAA
ac184654.1 1291-1476

Strongylocentrotus *Syt4*

Strongylocentrotus purpuratus synaptotagmin 4

N terminus missing, I don't think xm_779204 is right.

DPTTGASDPYVKLCLLPEKKHKVKTRVLRKTLNPIYEETFTFYGLAYNQLQ**^ 0**
GVTLHFVVMSFDRFSRDEVIGEVVMPLSNVDLSTRPVNLCRDIKPRNTR**^ 0**
IPKSQGRGELLTSLCYQPAANRLTVVVLKAKNLPKMDVTGLA**^ +1**
DPYVKIYVMYRNQRLAKKKTRLKKRTLNPVFNESFLFDIPMEGLEYLKIEFQ**^ 0**
VLDHDRVTKNEIIGRLVIGRDEDGEAESQHWKEIMQNPRKQIAEWHKLTE

(phase 0)

GATCCAACTACTGGTGCGTCCGACCCCTACGTTAAGCTGTGCCTGCTCCCTGAGAAGAAACAC
AAGGTGAAGACCAGGGTCCTGAGGAAAACTCTCAATCCCATCTACGAAGAAACATTTACCTTC
TATGGGCTGGCATATAACCAACTACAG
phase 0
aagj02103076.1 2231-2383

GGTGTAACTCTTCACTTTGTAGTGATGAGTTTTGATCGTTTCTCCCGGGACGAAGTGATAGGT
GAGGTGGTGATGCCCCTGTCCAACGTCGATCTGAGCACCAGACCGGTCAATCTATGTCGAGAT
ATCAAACCAAGAAATACACGG
phase 0
aagj02103076.1 3123-3269

ATCCCCAAGTCACAGGGCCGTGGTGAGTTGCTGACGTCACTGTGCTATCAACCGGCAGCTAAC
CGACTTACTGTGGTGGTCCTCAAGGCAAAGAATCTACCCAAGATGGATGTCACTGGACTTGCAG
phase +1
aagj02103076.1 3900-4026

ACCCATACGTGAAGATTTATGTGATGTACCGCAACCAGCGCCTAGCTAAGAAGAAGACTCGTC
TCAAGAAACGAACTCTAAACCCTGTCTTCAATGAATCCTTCCTCTTCGATATACCAATGGAGG
GACTGGAGTATCTCAAGATTGAATTTCAG
phase 0
aagj02103076.1 4427-4581

GTGCTGGATCACGACCGAGTGACAAAGAACGAAATCATCGGCCGATTGGTGATTGGACGAGAT
GAAGATGGAGAGGCGGAGTCGCAGCACTGGAAGGAGATCATGCAGAACCCGCGTAAACAGATC
GCAGAATGGCATAAACTCACCGAATAA
aagj02103076.1 5031-5183

Strongylocentrotus *Syt7*

Strongylocentrotus purpuratus synaptotagmin 7

N terminus missing.

xSERLKPNVGVEHIQPDPSKLAQSQLGNGNGQAEAIGI**^ 0**
AQGLDDDSMQFGEELGKIQFSLMYDFPDQTLVLRIVKANHLPAKDF**^ +2**
SGTSDPFVKIMLLPDKKVKMETKVKRKNLNPIWNESFHFE**^ +1**
GYPYSKIQERVLHLQVLDYDRFSRNDPIGEINLPLAEIDLTHEKLYWRSLTPSKKSS**^ 0**
GKLGSLLISLCYAPTAGRITITVLKCQNLAAKDITGKS**^ +1**
DPYVKIWHMHKDKRVEKKKTVIKYHTLNPVYNESFVFNIPLDRIRDTTFVVSVLDKDRL
SKNDMIGGILLGARTSPAEMSHWNEMMSKPRTNIAKWHVLKGVN

(phase +2)

GTCTGAAAGACTGAAACCCAATGTAGGCGTGGAACACATCCAGCCTGATCCAAGCAAACT
GGCACAGAGCCAACTGGGGAACGGCAATGGACAAGCAGAAGCAATCGGAATT
phase 0
ac178939.1 (4144-4033)

GCCCAAGGTTTAGACGATGACTCGATGCAGTTTGGGGAAGAGCTAGGGAAGATCCAGTTC
TCGCTCATGTATGACTTCCCAGACCAAACGCTGGTGCTAAGGATAGTCAAAGCAAATCAT
CTACCTGCCAAAGATTTTAG
phase +2
ac178939.1 (3513-3374)

CGGAACAAGTGATCCTTTTGTCAAAATCATGCTCCTGCCAGATAAAAAAGTCAAAATGGA
GACAAAGGTGAAGAGGAAAAACCTGAATCCCATCTGGAACGAGTCTTTCCATTTTGAAG
phase +1
ac178939.1 (2216-2098)

GTTATCCATACAGCAAAATACAAGAGCGTGTCCTACATCTGCAGGTGCTAGACTACGATC
GCTTCAGTAGAAACGACCCGATTGGAGAGATCAACTTGCCACTAGCCGAGATCGACCTGA
CCCACGAGAAGTTATACTGGAGGTCACTTACGCCGAGTAAAAAATCATCA
phase 0
aagj01008932.1 1571-1740

GGTAAACTAGGAAGTTTACTAATATCTCTCTGCTATGCACCTACGGCAGGAAGGATAACT
ATAACAGTCCTCAAGTGTCAAAACCTTGCAGCCAAAGATATCACCGGAAAATCAG
phase +1
aagj01008932.1 2809-2923

ATCCTTACGTGAAAATCTGGCACATGCACAAGGACAAACGCGTCGAGAAGAAGAAAACCG
TCATCAAGTACCACACGCTTAACCCCGTTTATAACGAGTCCTTCGTCTTCAACATCCCGC
TCGACAGGATACGCGACACGACCTTCGTCGTCTCGGTCCTCGACAAGGACCGGCTCTCAA
AGAACGATATGATCGGTGGGATCCTCCTTGGGGCGCGAACGTCGCCGGCCGAAATGAGCC
ATTGGAACGAGATGATGAGCAAGCCGCGGACTAATATCGCAAAATGGCACGTGCTGAAAG
GCGTTAATTGA
aagj01008932.1 4040-4350

Strongylocentrotus *Syt9*

Strongylocentrotus purpuratus synaptotagmin 9

MAFEGTGIGLTAFYIEEDVQCVDQKRIR**^ +1**
GRIFPLEEIAIPVCLSVAIVLIAFITLYCSKKKKTLSEPQDLYTVQRKVFNHHSYEIDSD
SDNDSMFQSRDGSPTGSRSSSGRPSPTRPTPLPRQSTVPLLPQKLRQEAFRKQRQLSLQL
NLTNVEFSVKNINSTRKDQTDLIGTLRPELYRQESKDKGKLNGESRPNCGRLVFSLFYDY
ATETLNVHIERAVGLPAKDFSGTSDPYVKIYLCPDRKRKYQTKVHRKNCDPVFDERFAFQ
IPYNELESKTLKFTVYDFDRFSRHDLIGEVNITSLLTDRDLSKETQYVEDIMKGTSQ**^ 0**
EKADLGEVMFSLNYLPTACRLTLTVIKARNLKAMDITGAS**^ +1**
DPYVKVSLMSQGKRIKKKKTTVKKNTLNPVYNEAMVFDVAPDSMENICLIIAVVDYD**^ +2**
WVGHSELIGVCEVGPNAPVQGAAHWADMLTNPRKPIAQWYQLQESTPALSVASLSAGL
KNCMGSSTQKSFDRD

exon1 as predicted in xm_776655 but I'm not sure this is right
ATGGCGTTCGAAGGTACAGGAATAGGTCTGACGGCGTTCTACATAGAGGAAGATGTGCAATGTGTCG
ACCAGAAACGCATCAGGG
phase +1 gc donor
aagj01171912.1 5651-5735

GGCGCATCTTTCCCTTGGAGGAGATTGCCATTCCCGTTTGCTTATCAGTAGCGATCGTGCTGATCGC
ATTTATTACTCTTTATTGCTCGAAAAAGAAGAAAACCCTGTCGGAGCCTCAAGACTTGTATACCGTC
CAGCGCAAGGTGTTCAATCATCACAGCTACGAGATTGACTCGGACTCGGACAACGATAGTATGTTCC
AAAGCCGTGATGGATCTCCTACAGGTAGTCGATCCAGCTCGGGAAGACCCAGTCCAACTCGGCCGAC

CCCTCTACCGCGTCAGTCAACTGTCCCGCTACTGCCGCAGAAGCTCCGTCAGGAAGCATTCCGAAAG
CAACGTCAGCTCTCCTTACAGCTTAACCTGACGAACGTCGAGTTCAGCGTCAAGAACATCAACTCCA
CGCGCAAAGACCAAACAGATCTGATAGGTACATTACGTCCAGAGCTCTACCGCCAAGAATCCAAAGA
CAAGGGCAAGCTCAACGGTGAGAGCCGACCCAACTGTGGCAGATTGGTTTTCAGCCTTTTCTACGAC
TACGCGACGGAAACGCTTAACGTTCACATAGAGCGAGCAGTTGGCCTTCCGGCCAAGGACTTCTCAG
GAACGTCAGATCCCTACGTGAAGATTTATCTGTGCCCTGACCGGAAGCGGAAGTACCAGACCAAAGT
TCATCGGAAAAATTGTGACCCTGTTTTCGATGAGAGATTTGCCTTTCAAATACCTTACAACGAGCTG
GAGAGCAAAACTCTGAAATTCACCGTCTACGATTTCGACAGGTTCTCCAGGCACGACCTTATAGGAG
AAGTGAACATCACCAGTCTTCTCACCGATAGGGATCTGAGTAAAGAAACACAATACGTCGAAGACAT
AATGAAAGGAACATCGCAA
phase 0
aagj01171912.1 9641-10530

GAGAAAGCGGATTTAGGGGAGGTGATGTTCTCACTCAACTATCTGCCTACAGCATGCCGGTTAACAC
TTACAGTCATCAAAGCCCGTAACTTAAAGGCAATGGACATCACAGGTGCCTCAG
phase +1
aagj01171912.1 12778-12898

ATCCCTACGTTAAAGTGTCTCTAATGTCGCAAGGGAAGCGGATCAAGAAAAAGAAAACGACTGTGAA
GAAGAACACTCTCAACCCCGTGTACAACGAGGCAATGGTCTTCGATGTCGCCCCGGACAGCATGGAA
AACATATGCCTCATCATCGCCGTAGTCGATTACGACTG
phase +2
aagj01171912.1 17413-17584

GGTTGGACACAGCGAACTGATCGGTGTCTGTGAAGTAGGGCCCAACGCTCCGGTCCAGGGTGCAGCC
CACTGGGCGGACATGCTGACGAACCCACGGAAGCCCATCGCGCAATGGTACCAACTCCAAGAATCAA
CGCCCGCCCTCTCCGTCGCGTCCTTGTCAGCAGGGCTCAAGAACTGCATGGGGTCTTCAACGCAGAA
GTCCTTTGATCGGGACTGA
aagj01171912.1 18941-19160

Strongylocentrotus *Syt12*

Strongylocentrotus purpuratus synaptotagmin 12

N termimus missing, I don't think xm_779838 is right.

xAGQVEVIIEYSSDPSRLIVTVVQARDLRPLPEGAALSDTYVTLYLDPDNDVKGQTKIYR
RSFSPVYNE**^ +2**
RFSMRVRWEDLPRRTLRLTVMNYDRHARHEEIGQTELQLEDIDWQHGPFNTWLNLHDSNE**^ 0**
KPENLGDIMFSLSYLPTAERLTVVIVKARGLIWSDNKKSG**^ +1**
DPFVKVYLLQNGKKISKKKTSMKRSETCPIFNEAMMFSVPSTILE**^ 0**
KVTLRITVAQFGMGGKTPSVGHVLIGANAKGSSLSHWNQMLISLRKPVAMWHSLRK

(phase +2)

AGCGGGTCAGGTTGAGGTCATTATCGAGTATTCATCAGATCCATCTCGTCTTATCGTCAC
CGTGGTACAGGCTCGTGACCTTCGACCTTTACCTGAAGGGGCGGCACTTTCTGACACCTA
CGTCACACTCTATCTTGACCCAGATAACGATGTCAAAGGTCAAACGAAGATATACAGAAG
AAGCTTCAGTCCGGTCTACAATGAAAG
phase +2
ac172487.2 (16687-16481)

GTTTTCAATGCGGGTCCGATGGGAGGACCTACCCCGCCGGACATTACGATTGACAGTGAT
GAACTATGATAGACACGCTCGTCATGAGGAGATAGGCCAGACCGAGCTACAACTAGAGGA
CATTGATTGGCAACATGGACCCTTTAACACGTGGCTTAATCTTCACGATTCTAATGAA
phase 0
ac172487.2 (14583-14406)

AAACCAGAGAACCTAGGAGACATCATGTTTTCTCTGAGCTATCTTCCAACAGCTGAGCGA
CTTACCGTGGTCATCGTTAAAGCTAGGGGCCTTATATGGAGTGATAACAAAAAATCAGGAG
phase +1
ac172487.2 (12193-12073)

ATCCATTTGTGAAAGTGTACCTTCTACAGAACGGCAAGAAGATAAGTAAAAAGAAGACATCAATGAA
AAGAAGTGAAACGTGTCCTATCTTTAATGAGGCTATGATGTTTTCTGTGCCATCGACCATTCTAGAG
phase 0
ac172487.2 (10478-10345)

AAAGTCACCCTACGTATTACTGTAGCTCAGTTTGGTATGGGTGGTAAGACCCCCAGCGTCGGTCACG
TGTTAATTGGTGCTAACGCCAAAGGATCATCACTAAGCCATTGGAATCAGATGCTAATCTCTCTCAG
AAAACCTGTCGCTATGTGGCATTCCCTCAGGAAATAG
ac172487.2 (9563-9393)

Strongylocentrotus *Syt17*

Strongylocentrotus purpuratus synaptotagmin 17

N terminus missing, I don't think xm_782888 is right.

HRGGGILWQLTATLCCGARCCRACCGDCCRPHHSSFQDLADDFDDDDNVDVPISPGYSSD
IPSSTSHSRLASTSSRRSSCGESRRGSTPVSEYSSSGYRRGSSTPVPVIDMKPIEFWPPN
MSQEPVQPRPLTRRYTSDLSSFGEADKIEPKLYEVNENEEDLTDEEKVARFKLGKIHYAL
KYEVNEGRLNVRIIKARDLPPPMFYDSSKQDLSHSNPYVKVCLLPDQKDARQTSVKRKTQ
NPNFEESFCFNIPHQEAQRRMLLLSVQDFDKFSRHCVIGQHTVPLAGLNLVKGGHYWKPL
QPPNQ**^ 0**
NNPGRGEILLSLNYLPSAGRLNVDVIKAKQLLQTDIVGGS**^ +1**
DPFVKLQMISGQKVIKTKKTSTKKNTLDPVFNETFSFHVTPSSLSDVSLLVSIWDYNTKS
RDYFTGQIIMGKFAS**^ +1**
GHSEVTHWQRMRNSQRTPVAQWHTLRTREDCEKVLPHAMMVS

(phase 0)

CACCGCGGTGGTGGTATACTATGGCAATTAACAGCGACTCTATGCTGTGGTGCTCGTTGC
TGCCGTGCCTGCTGCGGTGATTGCTGCCGTCCACACCACTCATCCTTCCAAGACCTCGCC
GACGATTTTGATGACGACGACAACGTCGACGTGCCTATATCGCCTGGTTACTCGAGCGAC
ATCCCATCATCGACATCACACTCGAGGTTAGCCTCCACCAGCTCGAGACGATCGTCGTGC
GGTGAATCGCGTCGTGGTTCGACACCGGTGTCGGAGTATTCGAGTTCGGGGTACCGACGG
GGTAGCAGCACCCCAGTCCCCGTCATAGACATGAAACCGATTGAGTTTTGGCCTCCTAAT
ATGAGCCAGGAACCGGTCCAGCCGAGGCCGCTCACGAGACGATATACGAGTGATCTCTCA
TCATTCGGTGAAGCTGATAAAATAGAACCAAAGCTATACGAGGTGAATGAAAATGAAGAA
GATCTGACGGACGAAGAGAAAGTGGCAAGGTTCAAATTGGGTAAGATCCACTACGCCCTC
AAATACGAGGTGAACGAGGGACGATTAAACGTCCGGATCATCAAGGCAAGGGACCTTCCA
CCGCCCATGTTCTACGACTCCTCCAAACAAGACTTATCACATTCCAACCCTTACGTCAAG
GTATGCCTCCTACCTGATCAGAAAGATGCCAGACAGACCTCGGTGAAACGGAAAACACAG
AATCCCAACTTTGAGGAGAGTTTCTGTTTTAATATCCCTCACCAAGAGGCGCAGCGGAGG
ATGTTGCTTTTGAGTGTACAAGACTTTGATAAGTTCAGCAGGCATTGTGTTATAGGACAG
CACACTGTACCCCTGGCGGGACTCAACCTGGTGAAGGGGGGCCATTACTGGAAACCCCTT
CAACCACCAAATCAG
phase 0
aagj01176051.1 (8816-7902)

AACAACCCCGGTCGTGGCGAGATACTATTATCTCTCAACTATCTACCCAGTGCAGGTAGG
CTCAATGTTGACGTCATCAAAGCCAAACAGCTGTTGCAGACTGATATAGTCGGAGGTTCGG
phase +1
aagj01176051.1 (7309-7189)

ATCCGTTTGTCAAGCTACAGATGATTAGTGGCCAAAAGGTGATCAAGACAAAGAAGACTT
CGACCAAGAAGAACACTCTCGATCCCGTCTTTAACGAGACCTTCAGTTTCCATGTGACGC
CGTCGTCACTGAGTGACGTAAGCTTACTGGTCTCCATATGGGACTACAACACCAAGAGCA
GGGACTACTTCACGGGACAGATCATCATGGGAAAGTTCGCATCAG
phase +1
aagj01176051.1 (6000-5776)

GTCATAGTGAGGTCACACATTGGCAACGCATGCGCAATTCGCAACGAACTCCTGTGGCAC
AGTGGCATACACTGCGCACTCGCGAAGACTGTGAAAAGGTTTTGCCACATGCCATGATGG
TTTCATGA
aagj01176051.1 (4989-4862)

Danio *Syt1* Gene 24 Craxton, M. BMC Genomics. 2004 Jul 6;5(1):43

Danio *Syt2* Gene 29 Craxton, M. BMC Genomics. 2004 Jul 6;5(1):43

Danio *Syt4*

Danio rerio synaptotagmin 4

Eukaryota; Metazoa; Chordata; Craniata; Vertebrata; Euteleostomi; Actinopterygii; Neopterygii; Teleostei; Ostariophysi; Cypriniformes; Cyprinidae; Danio.

MAPVTTEEAHF**^ +1**
AEVPVSVAVVSVFGLVFSVSIFAWICCQRKANKSSNKTPPYKFVHMLKGVDIYPESLSGK
KKFGGEKTPEAHGKQTLSPTGGRPDLHLDLEKRDLNGNFTTKPPTLQLKVRSSPDIDIPA
LQGGFANQEAGTPESVVSSHTPTPAVEKSQDKEGGLGTLFFSVEYNFEKKAFMVHIKEAH
GLSPTDEQSLTSDPYIKLTLLPEKKHKVKTRVLRKTLDPAFDETFSFYGIPFARVSQLAL
HFMVLSFDRFSRDEVIGETLVPLADIDLSEGRVLMSRDIIKKNIR**^ 0**
RSAGRGELLLSLCYQSTTSTLTVVVLKARHLPKADSSGPS**^ +1**
DPYVKVNLFQGKKRVCKKKTHVKKCAPNPVFNELFVFDLPSEDGLRDTSVELLLLDSDRT
SRTPVIGRLVLGTSSPGTAGEHWREICDHPRRQIAKWHALSED

ATGGCACCAGTGACGACAGAAGAAGCACATTTTG
phase +1
cr388231.3 205541-205574

CGGAGGTTCCTGTGAGCGTGGCAGTGGTCAGTGTGTTTGGTCTGGTCTTCAGTGTTTCCA
TCTTTGCATGGATTTGCTGTCAACGCAAAGCCAACAAGAGCAGCAACAAGACCCCACCCT
ACAAGTTCGTCCACATGCTGAAGGGGGTTGACATATACCCTGAAAGCCTGAGTGGAAAGA
AGAAGTTTGGAGGAGAAAAGACACCAGAAGCCCATGGAAAACAAACCCTCAGTCCTACTG
GTGGACGGCCAGACCTCCATCTAGACCTGGAGAAACGAGACCTAAATGGAAACTTCACCA
CAAAGCCACCGACACTCCAACTGAAGGTGCGTAGTTCTCCAGATATTGACATCCCAGCTC
TTCAGGGAGGATTTGCGAACCAGGAGGCTGGTACTCCAGAAAGCGTGGTATCCAGTCATA
CACCAACACCAGCCGTGGAGAAATCCCAGGACAAGGAAGGTGGTCTGGGAACGCTCTTCT
TTTCCGTTGAGTACAACTTTGAGAAGAAGGCTTTCATGGTTCACATCAAGGAGGCACATG
GTCTGTCGCCCACAGATGAACAGTCGCTGACCTCTGACCCCTATATTAAGCTGACCCTGC
TGCCTGAGAAGAAGCACAAGGTGAAGACACGTGTTTTGAGGAAGACTCTGGACCCGGCCT
TTGACGAGACCTTCAGCTTCTATGGAATCCCATTTGCACGAGTTTCCCAGCTGGCTCTGC
ACTTCATGGTGCTGAGTTTCGACCGATTTTCACGAGATGAAGTCATTGGAGAAACCCTTG
TTCCCCTGGCTGATATTGACCTGTCTGAGGGGCGCGTCCTCATGAGCCGAGACATTATTA
AGAAAAATATCAGG
phase 0
cr388231.3 206819-207672

AGGAGTGCTGGACGAGGAGAGCTGCTTCTGTCCCTGTGTTATCAGTCGACAACCAGCACT
CTGACTGTAGTGGTGCTGAAAGCCCGTCATTTGCCCAAAGCTGACTCCAGCGGACCCTCAG
phase +1
cr388231.3 1635-1755

ACCCTTACGTGAAGGTGAACCTGTTCCAGGGGAAGAAGCGTGTATGCAAGAAGAAGACGC
ATGTGAAAAAGTGTGCCCCCAACCCAGTCTTCAACGAGCTCTTCGTCTTCGACCTGCCCT
CAGAAGACGGCCTGCGAGACACCAGCGTGGAGCTTCTCCTGCTGGACTCCGACAGGACGT
CTCGTACGCCTGTCATTGGCCGGCTTGTTCTGGGAACCTCCTCTCCTGGCACCGCAGGCG
AGCACTGGCGCGAGATCTGTGACCACCCGCGGCGACAGATCGCCAAGTGGCATGCGCTTT
CCGAAGACTAG
cr388231.3 3835-4145

Danio *Syt5.1* Gene 26 Craxton, M. BMC Genomics. 2004 Jul 6;5(1):43

Danio *Syt5.2*

Danio rerio synaptotagmin 5.2

MGFRVRRTAEPAEPEPEPEPEPEPHSKPEHRPHPAPPTHHDYDNMKSKFMNELEHLP**^ +1**
LPMWAVGAIVVVVLALVACFTYCMFKKCFGKKKKSKKARERKRAARKKVEGTEGEGQGEQK**^ 0**
DEGEKKEGEEQKEEHENLGKLEFSLDYNFTDAQ**^ 0**
LIVGILQAQDLAAMDIGGTSDPYVKVYLLPDKKKKFETKVQRKNLCPVFNETFIFK**^ 0**
IPYAELGGKTLVLQVFDFDRFGKHDVIGQIKIPMNCVDLAQPLHEWRELENGEKEE**^ 0**
EKLGDVCISLRYVPTAGKLTVNIMEAKNLKKMDVGGLS**^ +1**
DPYVKIVLQHNGKRLKKKKTTVKKNTLNPYFNESFSFEVPFEQIQ**^ 0**
KVQLLITVYDYDKLGSNDPIGKTFIGYGATGVGLRHWSDMLANPRRPVAQWHTLQPEEEV
EAALKAPHR

ATGGGTTTCCGTGTTCGGCGCACTGCTGAACCAGCAGAACCAGAACCAGAACCTGAACCT
GAACCTCATTCTAAACCAGAACACAGGCCACATCCTGCTCCTCCAACTCATCATGACTAC
GACAACATGAAGAGCAAGTTCATGAATGAGCTGGAACATCTTCCAT
phase +1
ct573322.5 (136405-136240)

TGCCGATGTGGGCTGTAGGTGCCATCGTGGTGGTGGTTCTCGCCCTTGTGGCTTGTTTTA
CATATTGCATGTTCAAGAAATGCTTTGGCAAGAAAAAGAAATCCAAGAAAGCTCGAGAGA
GGAAGCGAGCAGCACGGAAAAAAGTCGAAGGAACTGAAGGAGAAGGTCAAGGAGAGCAAAAG
phase 0
ct573322.5 (133203-133022)

GATGAAGGGGAAAAAAAGGAGGGAGAGGAGCAAAAGGAGGAGCATGAAAATCTGGGAAAG
CTGGAATTCTCTTTGGATTATAACTTTACAGATGCCCAG
phase 0
ct573322.5 (132855-132757)

CTGATAGTTGGGATTCTTCAAGCTCAGGATCTGGCTGCAATGGATATCGGTGGCACGTCT
GATCCATATGTAAAAGTTTACCTGCTTCCAGACAAGAAGAAGAAGTTTGAAACCAAAGTC
CAACGCAAAAACCTCTGCCCTGTGTTCAACGAGACCTTTATTTTCAAG
phase 0
ct573322.5 (132672-132505)

ATTCCCTATGCTGAGTTGGGTGGTAAAACTTTGGTGCTTCAGGTGTTTGATTTCGATCGG
TTCGGTAAACACGATGTGATTGGCCAGATAAAGATTCCCATGAACTGCGTGGATCTTGCT
CAGCCGCTGCATGAATGGAGGGAACTCGAAAATGGAGAGAAGGAGGAG
phase 0
ct573322.5 (129472-129305)

GAGAAACTCGGAGACGTTTGTATTTCTTTGCGTTATGTGCCAACTGCTGGTAAACTCACA
GTCAACATAATGGAAGCCAAAAATCTTAAGAAGATGGATGTGGGTGGTTTATCAG
phase +1
ct573322.5 (129192-129078)

ATCCGTATGTGAAGATTGTGTTGCAGCATAATGGAAAGCGTCTTAAAAAGAAGAAGACGA
CCGTTAAAAAGAACACCCTGAACCCATACTTCAATGAGAGCTTTAGCTTTGAGGTCCCAT
TCGAACAGATTCAG
phase 0
ct573322.5 (126934-126801)

AAAGTCCAGCTCCTCATCACTGTGTATGATTACGACAAGTTGGGCAGCAATGATCCTATT
GGCAAAACCTTTATTGGATACGGAGCCACCGGTGTCGGTTTACGCCACTGGTCGGACATG
CTTGCTAATCCCCGCCGTCCAGTGGCCCAGTGGCACACACTTCAACCAGAAGAGGAGGTG
GAAGCTGCTCTGAAGGCGCCACACCGCTAA
ct573322.5 (126717-126508)

Danio *Syt6.1* Gene 44 Craxton, M. BMC Genomics. 2004 Jul 6;5(1):43

Danio *Syt6.2*

Danio rerio synaptotagmin 6.2

N terminal exon missing

xVSVGLPVGVIIVCVLALLLLASFGSWKLCWIPWRNKALSSSSAALAPDDCPSPHIPSVL
PSPQPSEAMATEKEKYPMASMGFLEAAVKISHTSPDIPAEVQLSMREHFLRRTQRMQRQT
TEPASST**^ +2**
RHSSFKRHLPRQMQVSSLDLGDDYDVDEQPTSIGRIKPELYKQMTTENDESANSKGGKNC
GKINFSLRYDYENEMLLVKILKAFDLPAKDLCGSSDPYVKIYLLPDRKQKFQTRVHRKTL
NPTFDESFQFPVPYDELAVRKLHLSVFDFDRFSRHDMIGEVILDNLFEVSDLSRETSIWR
DIQYATS**^ 0**
ESVDLGEIMFSLCYLPTAGRLTLTVIKCRNLKAMDITGYS**^ +1**
DPYVKVSLICDGRRLKKKKTTTKKNTLNPTYNEAIIFDIPPESMDQVSLHISVMDYD**^ +2**
LVGHNEIIGVCRLGCGAEGLGRDHWNEMLAYPRKPIAHWHPLLESKKTEKE**^ 0**
WKARTASFDSQGSCPSPKPPASP

(phase +1)

ACGTCTCTGTTGGCCTGCCGGTAGGTGTGATAATCGTGTGTGTCCTGGCACTGCTGCTGC
TCGCCTCATTCGGCTCCTGGAAGCTCTGCTGGATCCCCTGGAGGAATAAAGCCCTGTCGT
CCAGCTCTGCTGCCCTCGCTCCGGACGACTGCCCCTCGCCTCACATCCCATCTGTTCTGC
CCAGTCCTCAGCCGTCCGAGGCCATGGCGACGGAGAAGGAGAAGTACCCGATGGCATCCA
TGGGCTTCCTGGAAGCTGCTGTTAAAATCAGCCACACGTCTCCAGACATCCCGGCCGAGG
TGCAGCTCTCCATGAGAGAACACTTTCTGAGGAGGACACAGCGCATGCAAAGGCAGACCA
CAGAGCCTGCCTCGTCCACCAG
phase +2
cu041397.4 71101-71482

GCATAGCTCTTTCAAACGCCACCTTCCCCGTCAGATGCAGGTAAGCAGTCTGGACCTGGG
GGATGACTACGACGTGGACGAGCAGCCGACCAGCATCGGCCGCATCAAGCCTGAACTCTA
CAAACAGATGACCACAGAAAACGACGAGTCCGCCAACAGCAAAGGCGGGAAAAACTGTGG
CAAGATCAACTTCTCCCTCCGGTACGACTATGAGAACGAAATGCTACTAGTCAAAATTCT
CAAAGCTTTTGATCTGCCGGCCAAGGATCTCTGCGGCAGCTCTGACCCGTACGTTAAAAT
CTACCTGCTGCCCGACCGCAAGCAGAAGTTTCAGACGCGAGTCCACAGAAAAACACTCAA
CCCTACGTTTGACGAGTCTTTCCAGTTTCCTGTGCCGTACGATGAGCTGGCAGTCAGAAA
GCTCCATCTCAGCGTTTTTGACTTCGACCGCTTCTCTCGGCATGATATGATCGGAGAGGT
CATTCTGGACAATCTGTTTGAAGTGTCTGATCTCTCGAGGGAGACGTCCATCTGGAGAGA
CATTCAGTATGCTACCTCT
phase 0
cu041397.4 75317-75875

GAGAGTGTGGACCTTGGGGAGATCATGTTTTCACTCTGCTATCTACCTACTGCAGGCAGA
TTAACACTCACAGTCATCAAGTGCAGGAACCTGAAGGCCATGGATATTACAGGATATTCAG
phase +1
cu041397.4 84803-84923

ATCCCTATGTGAAAGTATCCCTCATTTGTGACGGCAGACGTCTGAAAAAGAAGAAGACCA
CAACAAAGAAAAACACCCTGAATCCAACGTATAATGAGGCCATTATCTTCGACATCCCTC
CTGAGAGTATGGACCAAGTCAGCTTGCACATCTCAGTCATGGATTATGACCT
phase +2
cu041397.4 87481-87652

TGTGGGCCATAATGAGATAATCGGTGTGTGTCGTTTGGGCTGTGGTGCTGAAGGTTTGGG
CCGAGACCACTGGAATGAGATGCTTGCGTACCCCCGTAAGCCCATTGCACACTGGCACCC
CCTGCTGGAGTCCAAGAAAACAGAAAAAGAG
phase 0
cu041397.4 90692-90842

TGGAAAGCCAGAACAGCTAGTTTTGACAGTCAGGGGTCTTGTCCTTCCCCCAAACCTCCT
GCCAGCCCT<u>TGA</u>
cu041397.4 93874-93945

Danio *Syt7*

Danio rerio synaptotagmin 7

N terminus missing, I don't think xm_684834 is right.

xLEQFGWCWTGEVTLTKTNYSDRFV**^ 0**
LSPGSEDDDHEGPVSEKLGRIQFSLGYSFQDTTLTVKILKGQDLPAKDFSGTSDPFVKIY
LLPDRKHKLETKVKRKNLNPHWNETFLFE**^ +1**
GFPYEKVRERTLYLQVLDYDRFSRNDPIGEVSIPLNKVELGQLKSFWKDLKPCSDGS**^ 0**
GSRGDLLVSLCYNPTANTITVNIIKARNLKAMDIGGTS**^ +1**
DPYVKVWLMHKDKRVEKKKTVTIKRCLNPVFNESFPFDVPAHVLRETTIIITVMDKDRLS
RNDVIGK**^ 0**
IYLSWKSGPGEVKHWKDMLSRPRTNVAQWHALKA

(phase +2)

CTTGGAGCAATTTGGATGGTGTTGGACCGGCGAGGTTACATTAACCAAAACAAACTATTC
TGACAGATTTGTG
phase 0
caak03064162.1 (29643-29571)

TTGTCTCCGGGGTCGGAGGACGATGACCATGAAGGTCCAGTGAGTGAGAAACTGGGCAGG
ATTCAGTTTTCTTTGGGCTACAGTTTCCAAGACACAACTCTGACGGTGAAGATCCTTAAA
GGTCAAGATCTGCCTGCTAAAGACTTCTCTGGGACCTCCGACCCTTTTGTGAAAATCTAC
CTACTGCCTGACAGAAAGCACAAACTTGAGACCAAAGTCAAGAGGAAAAATCTCAACCCG
CACTGGAACGAAACCTTCCTGTTTGAAG
phase +1
caak03064162.1 (28920-28653)

GGTTCCCATATGAGAAGGTGCGGGAGCGGACGCTGTACCTCCAGGTGCTGGATTACGATC
GGTTCAGCCGTAACGACCCAATCGGAGAGGTTTCCATTCCTCTGAATAAAGTGGAGCTCG
GACAGCTGAAGTCCTTCTGGAAGGATCTCAAACCCTGCAGTGACGGCAGC
phase 0
caak03064162.1 (28572-28403)

GGAAGTCGTGGTGATCTGCTGGTGTCTCTGTGTTATAACCCCACTGCCAACACCATCACT
GTCAACATCATAAAAGCTCGCAACCTCAAAGCCATGGACATCGGGGGCACCTCAG
phase +1
caak03064162.1 (28035-27921)

ATCCCTATGTAAAAGTATGGCTAATGCACAAAGACAAGCGAGTGGAAAAGAAGAAGACAG
TGACCATCAAACGCTGCCTGAACCCCGTTTTTAATGAATCCTTCCCCTTTGACGTGCCCG
CACATGTTCTCCGAGAGACCACCATCATCATCACCGTCATGGATAAAGATCGTCTGAGCC
GCAATGATGTCATTGGAAAG
phase 0
caak03064162.1 (27826-27627)

ATTTACCTGTCATGGAAGAGTGGTCCTGGTGAGGTCAAACATTGGAAGGACATGTTGAGC
CGGCCACGTACAAATGTGGCACAGTGGCACGCCCTCAAAGCCTGA
caak03064162.1 (27444-27340)

Danio *Syt8*

Danio rerio synaptotagmin 8

N terminus missing.

LTVGIKEAAALKAMDSGGTSDPYVKVYILPNKSKTFETKVFRKTLNPVFNENFKYQ**^ 0**
IPQKELTESTLVMQVYDFNRFSKHDIIGEIRLNLSTVDWNHVIEEWRDLSEASKHE**^ 0**
QEHLGEICFSLRYVPTSSKLTVIILEAKNLKKMDQVGSS**^ +1**
DPYVKVQLILEKKKWKKKKTSVKKRTLNPYFNESFTFDVSFEQIQ**^ 0**
KVQLVISVWDHDKMSRNDAIGKIYLGCDATGNQLRHWADMLSNPRKPVAQWHTLLS
AEQVDTTLALKHTLKIPFTNKNF

(phase 0)

CTGACTGTAGGAATAAAAGAAGCCGCTGCTTTGAAAGCGATGGATTCAGGAGGGACATCT
GACCCCTATGTGAAAGTCTACATCCTACCCAACAAGTCCAAAACATTTGAGACAAAAGTC
TTCAGGAAGACCCTTAACCCCGTGTTCAATGAAAATTTCAAATATCAG
phase 0
caak03065215.1 272-439

ATCCCTCAAAAGGAGCTAACCGAGTCGACACTGGTGATGCAAGTGTATGACTTTAACCGA
TTCTCCAAGCATGACATTATTGGAGAGATCAGACTGAATCTGTCCACGGTGGACTGGAAT
CATGTGATTGAAGAGTGGAGAGATCTCAGCGAAGCTTCCAAACACGAG
phase 0
caak03065215.1 1034-1201

CAAGAGCATCTGGGAGAGATCTGCTTTTCTCTCCGCTATGTCCCGACTAGTAGTAAACTT
ACTGTGATCATTCTGGAAGCCAAAAACCTGAAGAAGATGGACCAGGTAGGCTCTTCAG
phase +1
caak03065215.1 3069-3186

ACCCTTATGTGAAAGTACAACTGATTCTTGAAAAAAAGAAGTGGAAAAAGAAGAAAACAT
CAGTGAAGAAGCGGACGCTCAATCCGTACTTTAATGAGTCTTTCACTTTTGATGTGTCTT
TTGAACAAATTCAG
phase 0
caak03065215.1 4429-4562

AAAGTCCAGCTGGTCATCTCAGTGTGGGATCATGACAAGATGAGCAGGAATGACGCGATC
GGCAAGATCTATCTGGGCTGTGATGCCACAGGAAACCAGCTGCGCCACTGGGCCGATATG
CTGTCCAATCCACGCAAGCCTGTGGCTCAGTGGCACACTTTACTGTCCGCTGAACAGGTG
GACACGACGCTGGCCCTGAAGCACACGCTAAAGATCCCATTCACAAACAAAAACTTCTGA
caak03065215.1 5049-5288

Danio *Syt9.1* Gene 39 Craxton, M. BMC Genomics. 2004 Jul 6;5(1):43

Danio *Syt9.2*

Danio rerio synaptotagmin 9.2

C terminal exon missing

MPVDREDEICQKALELLSDLCSKGEVQNENCLDFIYYFRDLARPRYSDS**^ +1**
DISISLLSLVVTACGLALFGVSLFVSWKLCWIPWRERGLSPGTKEGHPDPPHPPPPPLQP
QPIYTEVDATLDRRSNARSSVVKETTVPPTPVSPAVPGSPPAVPVPEAALKISHTSPDIP
LEVESKTQENGVHGNTRMQRQITDPSSTCV**^ +2**
RQRSLDGEDSRSSRVGSCGRLHFILKYDCDLEQLIVKIHRAQDLPAKDFSGTSDPYVKIY
LLPDRKTKHQTKVHRKTLNPVFDEVFLFPVAYADLPTRKLHFSVYDFDRFSRHDIIGQVV
VDNFLDLVDFPRETKLCRDIQYVSS**^ 0**
DNVDLGDLMFSLCYLPTAGRLTITMIKARNLKAMDITGAS**^ +1**
DPYVKVSLMCDGRRLKKRKTSTKRNTLNPVYNEAIVFDVPPENIDQISLLVAVMDYD**^ +2**
RVGHNEVIGVCRVGNDAEGLGREHWNEMLTYPRKPIAHWHPLVE**^ 0**

ATGCCTGTCGACAGAGAGGACGAGATTTGTCAAAAAGCGCTAGAACTCTTATCGGATCTT
TGTTCTAAGGGAGAGGTTCAGAACGAAAACTGTTTGGATTTCATTTATTATTTCCGAGAC
CTTGCCAGGCCGAGGTATTCGGACTCAG
phase +1
caak03048970.1 (5707-5560)

ATATATCCATTAGTCTGCTGTCATTGGTGGTGACCGCTTGTGGCTTGGCCCTGTTTGGCG
TTTCCCTGTTTGTATCATGGAAATTGTGCTGGATTCCATGGCGCGAACGAGGCCTCTCTC
CAGGCACCAAGGAGGGACATCCAGACCCCCCTCATCCTCCTCCTCCACCCCTTCAACCCC
AGCCCATCTATACCGAGGTGGACGCCACCCTCGACCGCCGGAGTAATGCCCGCTCTTCAG
TGGTAAAGGAGACCACAGTGCCGCCCACACCAGTCTCTCCAGCGGTTCCAGGATCTCCTC
CTGCCGTGCCAGTGCCTGAGGCAGCCCTTAAAATAAGCCACACTTCACCTGATATTCCGC
TGGAGGTGGAATCAAAGACTCAAGAGAATGGTGTCCATGGAAACACACGTATGCAGAGGC
AAATCACGGATCCCTCCTCGACCTGTGTCAG

phase +2
caak03048968.1 (5840-5390)

GCAACGATCCCTTGATGGCGAAGACAGTCGAAGCAGTCGTGTGGGCAGCTGTGGACGCCT
ACACTTTATTCTTAAATACGACTGTGACCTGGAGCAGCTCATCGTCAAGATTCATCGAGC
CCAAGACCTCCCTGCCAAGGACTTTTCTGGAACCTCTGACCCATATGTTAAAATCTACCT
TCTCCCAGACCGCAAAACTAAGCACCAGACCAAGGTGCATCGTAAGACTCTGAACCCTGT
CTTTGATGAGGTCTTCCTCTTCCCTGTGGCGTATGCAGATCTGCCCACAAGGAAGTTGCA
CTTCAGCGTCTACGATTTTGATCGATTTTCACGCCACGATATTATTGGGCAGGTGGTGGT
TGACAACTTTCTGGATCTAGTGGACTTTCCCAGGGAGACCAAACTCTGCAGGGACATCCA
GTATGTGTCTTCG
phase 0
caak03048968.1 (2761-2330)

GACAATGTGGATCTGGGGGATTTGATGTTCTCTCTATGTTACCTGCCCACTGCTGGGCGA
CTGACCATCACCATGATCAAAGCACGGAATCTGAAAGCCATGGACATCACTGGAGCTTCAG
phase +1
caak03049142.1 (45235-45115)

ATCCTTATGTGAAAGTTTCTCTCATGTGTGATGGACGGAGACTGAAGAAAAGAAAAACCT
CCACTAAGAGGAACACATTAAACCCTGTGTACAATGAGGCCATAGTGTTTGATGTCCCTC
CAGAGAACATTGACCAAATCAGCCTTCTCGTAGCTGTTATGGATTATGACCG
phase +2
caak03049142.1 (39962-39791)

TGTAGGTCATAATGAAGTTATTGGCGTGTGCCGAGTAGGTAATGATGCAGAGGGTTTGGG
CAGAGAACACTGGAATGAAATGCTGACTTACCCACGCAAACCCATCGCACACTGGCACCC
TCTCGTCGAG
phase 0
caak03049142.1 (36489-36360)

Danio *Syt10* Gene 42 Craxton, M. BMC Genomics. 2004 Jul 6;5(1):43

Danio *Syt11.1* Gene 35 Craxton, M. BMC Genomics. 2004 Jul 6;5(1):43

Danio *Syt11.2*

Danio rerio synaptotagmin 11.2

Very short N terminal exon not identified

xMSPVLAGFIGAGVLVVAVFALIYLWTCCQKHYNRTNYKLHGIQSEHSDPLTDPPYKFIH
MLKGMSIYPDALTNSKRIIRVAREARAQTSDRVQGSKGRPVVLVNMDSAAESGLLGEKQM
RLEESGHEVSRLERELPVRADYCCLDSSSASSSQTSSTTVSSTATPFTPADEPSRGDLSI
AIDYNFPKKALVVTILEARGLPAVEGQTGSADPYVKMTILPEKKHRVKTRVLRKTLEPAF
DETFTFYGVPYSSLSDLTLHFLVLSFDRFSRDDVIGEAMVPLAGVDPSTGRVHITQQITK
RNMQ**^ 0**
CVSHGELLVSLSYQPVSHRLSVVVLKAKHLPKLDITGLSA**^ +1**
NPYVKLNVFYGHKRIAKKKTHVKKCTLNPVFNESFIYDVPAELLPDISIEFLVMDFDRTT
KNQALGRLVLGADSPCPSGAAHWQEVCQNPRRQISKWHTLN

(phase +1)

ACATGTCTCCAGTGCTGGCAGGGTTTATCGGTGCTGGCGTGTTGGTCGTTGCAGTCTTTG
CGTTAATCTACCTGTGGACTTGCTGTCAGAAACATTACAACAGGACCAACTACAAGCTCC
ATGGCATCCAGTCGGAACACAGCGATCCTCTCACAGACCCTCCGTACAAATTTATCCACA
TGCTGAAAGGGATGAGCATCTATCCTGATGCACTTACCAACAGCAAGAGAATAATTCGAG
TGGCACGTGAAGCCAGAGCTCAAACCTCTGACCGAGTACAAGGCAGCAAGGGCCGCCCAG
TGGTGCTGGTGAATATGGATTCGGCAGCAGAAAGTGGCTTGCTTGGGGAAAAGCAGATGA
GGCTTGAGGAAAGTGGGCATGAGGTTTCCAGGCTGGAGAGGGAGCTTCCGGTGCGAGCCG
ATTACTGCTGCTTGGATAGCAGCTCCGCAAGCAGCAGTCAGACGTCCAGCACTACGGTTT
CCAGCACTGCTACACCCTTCACCCCTGCCGATGAACCCAGCCGGGGAGACCTCAGCATTG

CAATCGACTATAACTTCCCCAAGAAGGCCTTGGTGGTCACCATCCTGGAGGCTCGTGGTT
TGCCGGCAGTGGAAGGTCAGACAGGCAGCGCCGACCCCTATGTAAAGATGACCATTCTTC
CGGAGAAGAAGCATCGAGTGAAGACTCGAGTTTTGAGGAAGACTCTGGAGCCTGCGTTTG
ATGAAACCTTCACGTTTTATGGCGTACCGTACAGCTCCTTGTCAGATCTCACGCTGCACT
TCTTGGTGTTGAGCTTTGATCGTTTTTCACGAGATGATGTGATTGGTGAGGCTATGGTTC
CGCTGGCTGGCGTGGACCCGAGTACAGGACGGGTTCATATTACACAACAGATCACAAAGA
GAAACATGCAG
phase 0
cr854916.5 22803-23713

TGTGTGAGCCATGGAGAGCTGCTGGTTTCTCTTTCCTATCAGCCTGTTTCTCACAGACTC
AGTGTGGTGGTGTTAAAAGCAAAACATCTTCCAAAACTGGACATCACTGGCTTGTCTGCAA
phase +1
cr854916.5 26098-26218

ATCCTTACGTGAAGCTGAATGTGTTCTACGGCCACAAGCGCATTGCCAAGAAGAAAACGC
ATGTGAAAAAATGCACGCTCAACCCCGTCTTTAACGAATCCTTCATCTATGACGTGCCAG
CTGAGTTGCTCCCTGACATCTCTATTGAGTTCCTGGTCATGGACTTTGACCGCACTACTA
AAAACCAGGCTTTGGGACGCCTGGTGCTCGGCGCAGACAGCCCATGCCCTTCAGGTGCTG
CCCACTGGCAGGAAGTCTGTCAAAACCCACGACGCCAAATATCAAAGTGGCACACGCTCA
AT<u>TAA</u>
cr854916.5 27326-27630

Danio *Syt12*

Danio rerio synaptotagmin 12

MSVQGQDISEYHLS**^ +1**
VVLNPPGWEVCLFVFGFLVLFAVVIVNLWRLYKSGTFPTPSPFPNFHYRYLQEKYGSSHSEVRQK**^ 0**
RVAACNQRRASSASRKPSLQLLDTPDGLRDLGTLELMSRELDQSGGSLNRSVSSESLCSI
SSVAQTFGHDFTVGQLEVTLELDTRASLLLVALHQGKDLLEKEEENFPGCFITVTLVPQQ
INLGATQ**^ 0**
VQRNAFTVVFDERFSVPLESVNLEENSLRFSTFGVDSDERNITAGVAELKLSDLDLPYRP
FNAWLYLQDINK**^ 0**
AVDAVGEILLSLSYLPTAERLTVVIAKAKNLVWTNGKTTA**^ +1**
DPFVKVYLLQDGRKISKKKTSIKRDDTNPIFNEAMIFSVPAIVLQ**^ 0**
DLSLRVTVAESTEDGRGENVGHVIIGPEASGMGITHWNQMLATLRKPVSMWHPLRRT

<u>ATG</u>TCTGTACAGGGTCAGGATATTTCTGAATATCATCTGAGCG
phase +1
caak03064445.1 (842-800)

TGGTGCTGAACCCGCCGGGATGGGAGGTGTGTTTGTTTGTGTTCGGGTTTCTGGTTCTGT
TTGCTGTGGTTATAGTCAATCTGTGGAGATTATATAAATCCGGCACCTTCCCGACTCCCT
CTCCATTCCCGAACTTTCACTACCGCTACCTGCAGGAGAAATATGGCTCGTCCCACTCCG
AGGTCAGACAAAAG
phase 0
caak03064445.1 (706-513)

CGTGTTGCTGCTTGTAACCAGCGGAGGGCGTCGTCTGCAAGCCGAAAGCCGAGTCTGCAG
CTGCTGGACACTCCAGACGGTCTCCGTGATTTGGGGACCCTGGAGCTGATGAGTCGAGAG
CTGGACCAGAGCGGAGGCTCCCTGAACCGCTCCGTGTCCTCAGAGTCGCTGTGCTCCATC
TCGTCTGTGGCTCAGACCTTCGGGCATGATTTTACGGTGGGCCAGCTGGAGGTCACGCTG
GAGCTGGACACTCGGGCGTCTCTGCTGCTCGTCGCTCTGCATCAGGGCAAAGACCTGCTG
GAGAAAGAGGAGGAGAACTTCCCCGGATGCTTCATCACCGTCACACTCGTCCCGCAGCAG
ATCAACCTGGGGGCCACGCAG
phase 0
caak03064444.1 (16085-15705)

GTCCAGAGGAACGCCTTCACCGTGGTATTTGACGAGCGTTTCTCTGTTCCTCTGGAATCA
GTCAATCTGGAGGAGAACAGTCTGCGATTCTCCACCTTTGGTGTGGATTCAGACGAAAGG
AACATCACTGCTGGAGTGGCAGAACTCAAACTGTCGGATCTGGATTTGCCATATCGGCCG
TTTAACGCCTGGCTCTATTTACAGGACATCAACAAG
phase 0

caak03064444.1 (12097-11882)

GCTGTGGATGCTGTGGGAGAGATCCTGCTGTCTCTCAGTTATCTGCCCACTGCAGAGCGT
CTCACCGTGGTCATCGCTAAAGCCAAGAACCTGGTCTGGACTAATGGAAAGACCACAGCAG
phase +1
caak03064444.1 (11761-11641)

ATCCATTTGTGAAGGTGTACCTGCTCCAGGACGGCAGGAAGATCAGTAAAAAAAAGACGT
CCATAAAGAGGGATGATACTAATCCAATCTTCAATGAGGCTATGATCTTCTCAGTGCCAG
CTATCGTCCTGCAG
phase 0
caak03064444.1 (11504-11371)

GATCTCTCTCTGAGAGTGACGGTCGCTGAGAGCACAGAGGACGGCCGTGGTGAAAACGTA
GGTCATGTGATCATCGGCCCGGAGGCCAGTGGGATGGGAATCACTCACTGGAACCAGATG
CTGGCGACTCTGCGCAAACCCGTCTCAATGTGGCACCCGCTGCGGAGGACCTAA
caak03064444.1 (9434-9261)

Danio *Syt13*

Danio rerio synaptotagmin 13

MLVSATALLGATLGTVSGVLTLCGLSLLCKSCKKGKLESGDEADPEKAKPSILHTLTQ**^ 0**
FSVHKCTEPIQPQASLKFPQIYRPKPSVTSQEVINYKEHGASNDTSAAELDTCNQATERE
EVFSLPRQ**^ +1**
ASADEIPCSSEQTGAMTTSSSILYPKLHFSISLHKESGELHINIVE**^ +1**
AENISVEAGCEGYISGCVSVSEEQKHAHTAVHKLAVHVQWGEELVFALPMESTEDTDSLD
GEVALSLHCCDRFSHNSTLGMMRFKLADVSMMLDADCWVDLQPPKQ**^ 0**
EVTSSTGELLLSLSYLPAANRLGVVVMKARGLQSDKLKDNI**^ +1**
DLSVKLTLKHQNAKLKKKQTRRVKHKMNPVWNEMMMLELPSELLAKSSVDLEVLNLASPG
TLLPLGRCMLGLQTSGTGLQHWKQMLDNPRKQIAMWHPLYT

ATGCTGGTGTCTGCGACAGCGCTGTTGGGGGCTACGCTGGGCACAGTGTCTGGAGTGTTG
ACACTATGTGGTCTCTCCCTGCTTTGCAAGAGCTGTAAAAAGGGGAAACTTGAGAGTGGA
GATGAGGCTGACCCAGAGAAAGCCAAACCTAGTATCCTGCATACTCTGACACAG
phase 0
bx957256.6 (210101-209928)

TTCAGTGTGCATAAGTGCACTGAACCCATCCAGCCTCAAGCATCTCTGAAGTTTCCTCAA
ATCTACCGGCCCAAACCTTCTGTGACTTCTCAAGAGGTGATAAACTACAAAGAACATGGA
GCTTCCAATGACACGTCTGCAGCCGAGCTCGACACCTGTAACCAAGCAACCGAACGTGAG
GAGGTTTTCTCCCTCCCGCGGCAAG
phase +1
bx957256.6 (206489-206285)

CTTCTGCTGATGAAATACCTTGCTCATCTGAACAAACTGGTGCCATGACGACAAGTAGCT
CCATCCTGTATCCGAAACTGCACTTCTCCATCAGCCTGCACAAAGAAAGCGGAGAGCTAC
ACATTAACATTGTTGAAG
phase +1
bx957256.6 (205183-205046)

CGGAGAATATATCAGTGGAAGCAGGCTGTGAGGGATATATATCAGGGTGTGTCAGTGTCT
CCGAAGAGCAGAAACATGCACACACAGCGGTCCACAAGCTGGCAGTGCATGTGCAGTGGG
GCGAGGAGCTGGTGTTTGCACTGCCTATGGAGAGCACAGAAGACACTGACAGTCTAGATG
GAGAGGTGGCGCTTTCCCTTCACTGCTGTGACCGATTCTCCCATAACTCCACTTTGGGCA
TGATGCGCTTCAAGCTGGCTGATGTGAGCATGATGTTGGACGCCGACTGCTGGGTTGACT
TACAGCCACCCAAACAG
phase 0
bx957256.6 (201382-201066)

GAAGTGACATCATCCACTGGAGAATTACTACTGTCGCTCAGTTATCTACCAGCAGCCAAC
AGACTTGGGGTGGTAGTAATGAAGGCTAGAGGACTCCAGTCAGATAAACTCAAAGACAAC
ATAG
phase +1

bx957256.6 (196458-196335)

ATCTATCCGTAAAGCTGACCTTAAAGCACCAAAATGCCAAGCTGAAGAAGAAGCAGACGC
GGCGAGTAAAGCACAAGATGAATCCAGTCTGGAACGAGATGATGATGTTGGAGCTGCCAA
GTGAGCTTCTGGCTAAATCCAGTGTGGATTTGGAGGTACTGAACCTGGCCAGCCCCGGGA
CCCTGCTCCCTCTGGGCCGCTGCATGCTGGGGCTCCAGACATCCGGCACTGGCCTGCAGC
ACTGGAAACAGATGCTAGATAATCCACGCAAACAAATCGCAATGTGGCATCCTCTATACA
CCTAA
bx957256.6 (194077-193773)

Danio *Syt14* Gene 50 Craxton, M. BMC Genomics. 2004 Jul 6;5(1):43

Danio *Syt16* Gene 49 Craxton, M. BMC Genomics. 2004 Jul 6;5(1):43

Danio *Syt17*

Danio rerio synaptotagmin 17

MAYTQ**^ 0**
LEPINE**^ 0**
GLLSRLSDLLLCRWSCRSCWQWCWECSCCQSSEEEVEILGPFPAQTPSW**^ +2**
LVNDYNDEKGTISHLVDQDGASSPQSDPTINCRPPTSEAARSTFSLA**^ +1**
GLNARRPSSPMVDVKPIEFWAMGPRKEVVQPLRKPPTPPDDYFRKLEPHLYSLDSCSDDV
DSLTDEEILMRYQLGMLHFSTQYDLINAHLIVRVIEARDLPPPVTCDGARQDMAHSNPYV
KMSLLPDNKNSRQTGVKRKTQNPVFEERFTFDLPFLEAQRRTLLLSVVDFDKFSRHCVIG
KVALPLSEVDLVKGGHWWKALVPSSQ**^ 0**
NEVELGELLLSLNYLPSAGRLNVDIIRAKQLLQTDMCQGS**^ +1**
DPFVKVQLVTGLKLMKSKKTSCMRGTIDPCYNESFSFRVPQEDLCEVSLVLT**^ +1**
VYGHNVKSSNDFVGRIVIGQFSSGPQETTHWRRLLSSQRTPVEQWHSLRSRAECDRVSPASLEVT

ATGGCGTACACACAG
phase 0
caak03006411.1 1944-1958

TTGGAACCCATCAATGAG
phase 0
caak03006411.1 4823-4840

GGTCTTCTGTCCAGACTGTCTGATTTGCTGTTGTGCCGCTGGTCATGCCGCTCCTGTTGG
CAGTGGTGTTGGGAGTGCAGCTGCTGTCAGTCTTCTGAAGAAGAGGTGGAGATTTTGGGG
CCTTTTCCAGCTCAGACTCCATCATGGCT
phase +2
caak03006411.1 6965-7113

TGTCAATGACTACAATGATGAGAAAGGCACAATTTCTCACCTCGTGGATCAGGACGGCGC
TTCTTCTCCCCAATCCGATCCCACTATAAACTGCAGGCCACCCACCTCAGAAGCTGCCCG
ATCCACCTTCAGCTTGGCAG
phase +1
caak03006412.1 4471-4610

GTCTGAATGCCCGACGCCCAAGCTCCCCCATGGTGGATGTAAAGCCAATTGAATTTTGGG
CCATGGGACCCAGAAAGGAAGTAGTTCAGCCTCTGCGAAAGCCACCTACACCACCGGACG
ACTACTTTCGCAAGCTGGAGCCCCATCTTTACTCTTTAGACTCATGCAGTGATGATGTGG
ACTCTCTAACAGATGAAGAGATCCTGATGCGGTACCAGCTGGGCATGCTGCACTTCAGCA
CACAATACGACCTCATCAATGCCCACCTCATCGTGCGAGTCATTGAGGCCAGGGACCTTC
CTCCTCCTGTAACATGCGACGGTGCCCGCCAAGACATGGCCCACTCGAACCCCTACGTGA
AGATGAGCTTGCTGCCCGACAACAAGAACTCAAGGCAGACGGGAGTTAAGCGAAAAACCC
AGAACCCAGTGTTTGAAGAGCGTTTCACTTTTGATCTCCCCTTCCTGGAGGCCCAAAGAC
GCACACTACTGCTCTCTGTGGTCGACTTTGACAAGTTTTCACGCCACTGTGTCATCGGAA
AAGTGGCTCTTCCTCTTAGTGAGGTTGATCTGGTGAAGGGAGGACATTGGTGGAAGGCCC
TGGTGCCCAGCTCTCAG
phase 0
caak03006412.1 4704-5320

AATGAGGTTGAGCTTGGTGAGCTGCTGCTGTCCTTAAACTATCTGCCCAGTGCTGGCAGG
TTAAATGTGGACATCATCCGAGCAAAGCAGCTGCTGCAGACAGACATGTGCCAAGGCTCAG
phase +1
caak03006412.1 8119-8239

ATCCATTTGTGAAGGTGCAGTTGGTGACGGGTCTGAAGCTGATGAAGTCTAAGAAGACAT
CCTGTATGAGAGGCACTATTGACCCCTGCTACAATGAGTCCTTTAGCTTCCGTGTACCAC
AAGAGGACCTGTGTGAAGTCAGTCTTGTGTTAACAG
phase +1
caak03006412.1 9572-9727

TTTACGGGCACAACGTGAAGAGTAGCAACGATTTTGTTGGCCGTATTGTGATTGGCCAGT
TCTCCAGCGGGCCTCAGGAAACCACACACTGGCGTAGACTGCTGAGCTCCCAGCGAACCC
CAGTGGAACAGTGGCACAGCCTGCGCTCCCGAGCTGAGTGTGACCGCGTCTCTCCTGCAT
CTCTTGAGGTCACATAA
caak03006412.1 21007-21203

Homo *Syt1* Gene 69 Craxton, M. BMC Genomics. 2004 Jul 6;5(1):43

Homo *Syt2* Gene 70 Craxton, M. BMC Genomics. 2004 Jul 6;5(1):43

Homo *Syt3* Gene 71 Craxton, M. BMC Genomics. 2004 Jul 6;5(1):43

Homo *Syt4* Gene 72 Craxton, M. BMC Genomics. 2004 Jul 6;5(1):43

Homo *Syt5* Gene 73 Craxton, M. BMC Genomics. 2004 Jul 6;5(1):43

Homo *Syt6* Gene 74 Craxton, M. BMC Genomics. 2004 Jul 6;5(1):43

Homo *Syt7* Gene 75 Craxton, M. BMC Genomics. 2004 Jul 6;5(1):43

Homo *Syt8* Gene 76 Craxton, M. BMC Genomics. 2004 Jul 6;5(1):43

Homo *Syt9* Gene 77 Craxton, M. BMC Genomics. 2004 Jul 6;5(1):43

Homo *Syt10* Gene 78 Craxton, M. BMC Genomics. 2004 Jul 6;5(1):43

Homo *Syt11* Gene 80 Craxton, M. BMC Genomics. 2004 Jul 6;5(1):43

Homo *Syt12* Gene 79 Craxton, M. BMC Genomics. 2004 Jul 6;5(1):43

Homo *Syt13* Gene 81 Craxton, M. BMC Genomics. 2004 Jul 6;5(1):43

Homo *Syt14* Gene 82 Craxton, M. BMC Genomics. 2004 Jul 6;5(1):43

Homo *Syt15* Gene 84 Craxton, M. BMC Genomics. 2004 Jul 6;5(1):43

Homo *Syt16* Gene 83 Craxton, M. BMC Genomics. 2004 Jul 6;5(1):43

Homo *Syt17*

Homo sapiens synaptotagmin 17

MAYIQ**^ 0**

LEPLNE**^ 0**
GFLSRISGLLLCRWTCRHCCQKCYESSCCQSSEDEVEILGPFPAQTPPW**^ +2**
LMASRSSDKDGDSVHTASEVPLTPRTNSPDGRRSSSDTSKSTYSLTRRIS**^ +1**
SLESRRPSSPLIDIKPIEFGVLSAKKEPIQPSVLRRTYNPDDYFRKFEPHLYSLDSNSDD
VDSLTDEEILSKYQLGMLHFSTQYDLLHNHLTVRVIEARDLPPPISHDGSRQDMAHSNPY
VKICLLPDQKNSKQTGVKRKTQKPVFEERYTFEIPFLEAQRRTLLLTVVDFDKFSRHCVI
GKVSVPLCEVDLVKGGHWWKALIPSSQ**^ 0**
NEVELGELLLSLNYLPSAGRLNVDVIRAKQLLQTDVSQGS**^ +1**
DPFVKIQLVHGLKLVKTKKTSFLRGTIDPFYNESFSFKVPQEELENASLVFT**^ +1**
VFGHNMKSSNDFIGRIVIGQYSSGPSETNHWRRMLNTHRTAVEQWHSLRSRAECDRVSPASLEVT

<u>ATG</u>GCGTACATCCAG
phase 0
ac010494.4 (49426-49412)

TTGGAACCATTAAACGAG
phase 0
ac010494.4 (45313-45296)

GGTTTTCTTTCTAGAATCTCTGGTCTGCTGCTGTGCAGATGGACCTGCCGGCACTGCTGT
CAGAAGTGCTACGAGTCCAGCTGTTGCCAGTCAAGTGAGGATGAAGTTGAAATTCTGGGA
CCTTTCCCTGCTCAGACCCCTCCCTGGCT
phase +2
ac010494.4 (44643-44495)

GATGGCCAGCCGGAGCAGTGACAAGGATGGTGACTCTGTCCACACGGCCAGCGAAGTCCC
GCTGACCCCACGGACCAATTCCCCGGATGGAAGACGCTCGTCCTCAGACACATCCAAGTC
TACATACAGCCTGACGCGGAGGATTTCGA
phase +1
ac010494.4 (37682-37534)

GTCTTGAGTCAAGACGTCCCAGCTCTCCACTCATCGATATTAAACCCATCGAGTTTGGCG
TTCTCAGCGCCAAGAAGGAGCCCATCCAACCTTCGGTGCTCAGACGGACCTATAACCCCG
ACGACTATTTCAGGAAGTTCGAACCCCACCTGTACTCCCTCGACTCCAACAGCGACGATG
TGGACTCTCTGACAGACGAGGAGATCCTGTCCAAGTACCAGCTGGGCATGCTGCACTTCA
GCACTCAGTACGACCTGCTGCACAACCACCTCACCGTGCGCGTGATCGAGGCCAGGGACC
TGCCACCTCCCATCTCCCACGATGGCTCGCGCCAGGACATGGCGCACTCCAACCCCTACG
TCAAGATCTGTCTCCTGCCAGACCAGAAGAACTCAAAGCAGACCGGGGTCAAACGCAAGA
CCCAGAAGCCCGTGTTTGAGGAGCGCTACACCTTCGAGATCCCCTTCCTGGAGGCCCAGA
GGAGGACCCTGCTCCTGACCGTGGTGGATTTTGATAAGTTCTCCCGCCACTGTGTCATTG
GGAAAGTTTCTGTGCCTTTGTGTGAAGTTGACCTGGTCAAGGGCGGGCACTGGTGGAAGG
CGCTGATTCCCAGTTCTCAG
phase 0
ac010494.4 (34545-33926)

AATGAAGTGGAGCTGGGGGAGCTGCTTCTGTCACTGAATTATCTCCCAAGTGCTGGCAGA
CTGAATGTTGATGTCATTCGAGCCAAGCAACTTCTTCAGACAGATGTGAGCCAAGGTTCAG
phase +1
ac003003.1 39847-39967

ACCCCTTTGTGAAAATCCAGCTGGTGCATGGACTCAAACTTGTGAAAACCAAGAAGACGT
CCTTCTTAAGGGGCACAATTGATCCTTTCTACAATGAATCCTTCAGCTTCAAAGTTCCCC
AAGAAGAACTGGAAAATGCCAGCCTAGTGTTTACAG
phase +1
ac003003.1 41485-41640

TTTTCGGCCACAACATGAAGAGCAGCAATGACTTCATCGGGAGGATCGTCATTGGCCAGT
ACTCTTCAGGCCCCTCTGAGACCAACCACTGGAGGCGCATGCTCAACACGCACCGCACAG
CCGTGGAGCAGTGGCATAGCCTGAGGTCCCGAGCTGAGTGTGACCGCGTGTCTCCTGCCT
CCCTGGAGGTGACC<u>TGA</u>
ac003003.1 83682-83878

Rabphilin and Doc2 genes

Rabphilin and Doc2 are related genes with a partly shared gene structure which encodes two tandem C terminal C2 domains. Doc2 genes comprise the C terminal half of Rabphilin genes and thus lack the N terminal Rabphilin effector domain. Whereas Rabphilin genes are widely distributed among metazoans, Doc2 genes appear to be restricted to vertebrates. Mouse has one Rabphilin gene and three Doc2 genes. The examples below illustrate the common gene structure.

Ciona intestinalis Rabphilin

MLTGNAEDAAQHAWVCPSDRQLTLRSKLR**^ +2**
AGWSVRAAQTDRQRRSSTLSDEELKKIRQVIERAENMDQAEQKRV**^ +2**
GKLVHRLDDMRSNSAGDGKLRCILCAEAFGKLMGASSFVCVDCKK**^ 0**
NVCSKCSVEFSPHLIRGSHSMDSSRGRSSNRKSSTTKWLCKICSEGRELWKRSGAWFFQSLP**^ +2**
RYILPEQTSNGMNQNFPIRTLGSTIIPQSPGSPMHKPTKDFATTPTSTYTQHKYM**^ +1**
VRRRSSSSESSDSSDISSFGKPASFYK**^ +1**
KSDSASITSSTSYTNSNDASSRRSEQILFDNNPARS**^ +1**
NGFAALSGSENSLNVPGGARKRRISGTRNRQHKKSVGDKSIEDI**^ +1**
GTNNPDMWSLHSSGVDGGSYIVETNAKNEIVTENT**^ +1**
AGLGSLEFTLLHDSHKLALYVTVIRAR**^ 0**
GLKAMDINGFSDPYVKLHLLPGSKK**^ 0**
STKMRTKTKQKTLNPTFDETLTYWGVTDADIQKKTLRLTVLDED**^ +2**
RLGDNEFIGEVRIQLKNFNLTQTNTYNMGLTEHQ**^ 0**
DIKEDEDSSERGRIMVTLSYQPRDTMLVATINRCSSLLPVARTNTIDPQVK**^ +2**
LCLKSEANPSEFTFKKTERRKNTKTSNPAFNETMKLPLPAQARELVNCSLDVSVWDKDTFGKEHLI**^ +1**
GAVCFGIHSKGDKLKQWFNCVKKPQEAHEMWHSLTLPNDPEKISSLAEAGRRSRTPRFHRKLHS

ATGCTTACGGGAAACGCAGAAGACGCAGCACAGCATGCTTGGGTTTGCCCATCTGATCGT
CAACTAACTCTACGCTCAAAACTACG
phase +2
aabs01000139.1 (23920-23835)

AGCTGGGTGGTCAGTTAGAGCTGCACAAACTGACCGACAGAGGCGATCCAGCACGCTTAG
TGATGAGGAATTAAAGAAGATACGTCAAGTCATAGAAAGAGCAGAAAACATGGATCAAGC
GGAGCAAAAACGAGTGGG
phase +2
aabs01000139.1 (23358-23221)

AAAGCTGGTGCACAGACTTGATGATATGAGAAGTAATTCAGCTGGTGACGGGAAGTTACG
ATGTATATTATGTGCTGAGGCATTTGGGAAGCTAATGGGAGCTTCTTCATTTGTTTGTGT
GGATTGTAAAAAG
phase 0
aabs01000139.1 (22765-22633)

AATGTATGCAGCAAATGTTCTGTTGAATTTTCCCCTCATTTAATACGTGGATCACACTCA
ATGGATAGCAGCCGTGGAAGGAGCAGCAACCGAAAGTCTTCAACTACTAAATGGCTTTGT
AAAATATGCAGTGAGGGCAGGGAGTTGTGGAAAAGATCGGGTGCTTGGTTCTTTCAAAGC
TTGCCAAG
phase +2
aabs01000139.1 (22341-22154)

GTATATACTGCCAGAACAGACATCTAATGGGATGAACCAAAACTTCCCGATCCGTACGTT
AGGTAGCACCATTATCCCTCAATCCCCTGGATCTCCAATGCATAAGCCAACCAAAGACTT
TGCAACAACACCTACATCTACATACACACAACATAAATATATGG
phase +1
aabs01000139.1 (21792-21629)

TCCGAAGAAGGTCGTCCAGCAGTGAGAGTTCGGACAGCAGCGATATTTCAAGTTTCGGAA
AACCTGCAAGCTTTTACAAAA
phase +1
aabs01000139.1 (20452-20372)

AAAGCGATTCAGCGAGCATCACAAGCAGCACAAGTTACACAAACAGTAACGACGCTTCTA
GCCGAAGATCGGAGCAAATTTTGTTCGACAATAATCCAGCGCGCTCAA
phase +1
aabs01000139.1 (19470-19363)

ATGGGTTTGCTGCGTTATCGGGCAGTGAAAACAGTCTCAATGTACCAGGTGGGGCAAGGA

AACGTAGAATTTCTGGCACCAGGAACCGACAGCATAAGAAATCCGTTGGAGATAAATCAA
TAGAAGACATTG
phase +1
aabs01000139.1 (18816-18685)

GCACCAACAACCCAGACATGTGGAGTTTACATTCAAGTGGTGTCGATGGAGGAAGCTATA
TTGTTGAAACGAATGCAAAAAATGAAATTGTTACTGAAAATACAG
phase +1
aabs01000139.1 (17906-17802)

CTGGCCTTGGTAGTTTGGAGTTCACCCTACTACACGACTCCCACAAGCTTGCATTGTATG
TCACTGTGATCCGCGCAAGG
phase 0
aabs01000139.1 (16245-16166)

GGTCTTAAAGCAATGGATATCAACGGGTTCTCTGATCCTTATGTTAAGCTCCACCTTTTA
CCAGGTTCCAAGAAG
phase 0
aabs01000139.1 (15652-15578)

TCAACTAAAATGAGGACCAAGACGAAACAAAAGACACTGAACCCGACATTTGATGAAACC
CTCACATATTGGGGGGTTACGGACGCCGATATTCAAAAGAAAACTTTACGTCTCACAGTC
TTGGATGAAGACAG
phase +2
aabs01000139.1 (12887-12754)

ACTTGGAGATAATGAATTCATTGGTGAAGTAAGGATTCAATTAAAGAATTTCAACTTAAC
TCAAACCAATACATACAACATGGGACTCACTGAACATCAG
phase 0
aabs01000139.1 (12269-12170)

GATATAAAGGAAGATGAAGATAGTAGTGAAAGAGGAAGGATTATGGTGACCTTATCCTAC
CAACCTAGAGATACAATGTTAGTCGCAACCATTAACAGATGTTCAAGTCTTCTTCCTGTT
GCAAGGACAAACACAATTGACCCACAAGTTAAACT
phase +2
aabs01000139.1 (11770-11616)

CTGCTTAAAGTCAGAAGCAAATCCTTCAGAGTTCACCTTCAAGAAAACTGAGCGTCGAAA
AAATACGAAGACCTCTAACCCTGCTTTCAATGAAACTATGAAGCTACCATTGCCCGCACA
AGCCCGTGAACTTGTTAATTGCTCGTTGGATGTGAGTGTGTGGGACAAAGACACCTTTGG
GAAGGAACATCTTATTG
phase +1
aabs01000139.1 (11426-11230)

GTGCTGTGTGCTTTGGTATTCACAGTAAAGGTGACAAACTAAAGCAATGGTTCAACTGTG
TAAAGAAACCTCAAGAAGCACACGAGATGTGGCATTCACTAACATTACCAAACGATCCAG
AGAAAATTAGCAGCCTAGCTGAAGCAGGGAGACGAAGCAGAACTCCACGTTTCCATAGAA
AACTACATTCATAG
aabs01000139.1 (10914-10721)

Strongylocentrotus purpuratus Rabphilin incomplete

MSYVYTCRTTLLQCEGHSYLDI**^ +2**
RRLVDKLDNMKKNSLGNGTTQCILCGDEFGLLGASPMTCYDCYK**^ 0**
x
?CLKAMDSNGISDPYVKLHLLPGATK**^ 0**
STKLRTKTVAKTLNPDFNETLTYYGVTEDDLSRKIL**^ +2**
RLSVLDEDRFGHNDFIGEYRLPLRKLTPYQTKSLSVYLEKPLP**^ 0**
LEKDDELAGERGKLMVGLKYVSTRQCLVVSIIRGAGLAAMDSNGYSDPYVK**^ +2**
VYLKPDAGKRTKHKTAVKKRTLNPEFNE**^ 0**
EFYYEVKHPELAKKTLEITVWDKDIAKANDYI**^ +1**
GGVQLGITSKGERLRHWFETLKGIDKKYERWHTLSDESFGDE

ATGAGCTATGTTTACACATGCAGGACCACATTACTGCAGTGTGAAGGCCATTCATATCTA

GACATCCG
phase +2
ac178080.1 99681-99748

GCGACTGGTGGATAAGCTAGACAATATGAAGAAAAACTCCCTTGGTAATGGGACAACTCA
GTGTATCCTCTGTGGGGATGAGTTTGGTCTCCTAGGAGCTTCACCTATGACCTGCTATGA
TTGTTACAAG
phase 0
ac178080.1 100295-100424

gap

nnnTGTCTCAAAGCCATGGACTCTAACGGCATATCAGATCCTTACGTCAAGCTACATTTG
TTGCCCGGGGCAACCAAG
phase 0
ac178080.1 106441-106515

TCTACCAAGCTCCGAACCAAGACCGTTGCCAAAACCCTGAATCCAGACTTCAACGAAACT
CTGACTTACTATGGGGTTACAGAGGATGACCTCTCAAGAAAGATCCTCAG
phase +2
ac178080.1 107382-107491

GTTATCAGTATTGGATGAGGATAGATTTGGTCACAATGATTTCATTGGAGAATACAGACT
ACCGCTCAGAAAGCTAACACCATACCAAACCAAGTCACTTAGTGTCTACCTAGAAAAACC
TCTTCCA
phase 0
ac178080.1 108115-108241

TTGGAGAAGGATGATGAGTTAGCTGGAGAGCGAGGCAAGCTGATGGTTGGTCTGAAGTAC
GTCAGCACCAGACAGTGCTTGGTAGTGAGTATTATCCGAGGGGCGGGGCTAGCTGCCATG
GACTCCAACGGTTACTCAGATCCTTACGTTAAAGT
phase +2
ac178080.1 109387-109541

ATACCTGAAGCCAGACGCAGGGAAGAGAACCAAGCATAAGACTGCAGTCAAGAAGAGGAC
CCTCAATCCTGAATTCAATGAG
phase 0
ac178080.1 110377-110458

GAATTCTACTATGAGGTGAAGCACCCTGAGTTGGCCAAGAAGACTCTAGAGATCACAGTG
TGGGATAAAGACATCGCCAAGGCAAATGATTATATTG
phase +1
ac178080.1 111067-111163

GTGGTGTGCAACTTGGGATCACCAGTAAGGGTGAGCGTCTACGTCACTGGTTTGAGACTC
TCAAAGGAATCGACAAGAAGTATGAGAGATGGCACACTCTTTCTGATGAAAGCTTTGGAG
ATGAATAA
aagj01174363.1 5319-5446

Mus musculus Rabphilin3a

MTDTVVNRWMYPGDGPLQS**^ +2**
NDKE**^ +2**
QLQAGWSVHPGAQTDRQRKQEELTDEEKEIINRVIARAEKMEAMEQERI**^ +2**
GRLVDRLETMRKNVAGDGVNRCILCGEQLGMLGSACVVCEDCKK**^ 0**
NVCTKCGVETSNNRPHPVWLCKICLEQRE**^ 0**
VWKRSGAWFFKGFPKQVLPQPMPIKKTKPQQPAGEPATQEQPTPESRHPARAPAR**^ +1**
GDMEDRRPPGQKP**^ +1**
GPDLTSAPGRGSHGPPTRRASEARMSTAARDSEGWDHAHGGGTGDTSRSPA**^ +1**
GLRRANSVQAARPAPAPVPSPAPPQPVQP**^ +1**
GPPGGSRATPGPGRFPEQST**^ +1**
EAPPSDPGYPGAVAPAREERTGPAGGFQAAPHTAAPYSQAAPARQPPPAEEEEEEANSYDSDEA**^ +1**
TTLGALEFSLLYDQDNSNLQCTIIRAK**^ 0**
GLKPMDSNGLADPYVKLHLLPGASK**^ 0**
SNKLRTKTLRNTRNPVWNETLQYHGITEEDMQRKTL**^ +2**

RISVCDEDKFGHNEFIGETRFSLKKLKANQRKNFNICLERVIP**^ 0**
MKRAGTTGSARGMALYEEE**^ 0**
QVERIGDIEERGKILVSLMYSTQQGGLIVGIIRCVHLAAMDANGYSDPFVK**^ +2**
LWLKPDMGKKAKHKTQIKKKTLNPEFNE**^ 0**
EFFYDIKHSDLAKKSLDISVWDYDIGKSNDYI**^ +1**
GGCQLGISAKGERLKHWYECLKNKDKKIERWHQLQNENHVSSD

start1
CTTCAGTTTGCAGATGGGACGTGGAAACATTAAGTGCCATCCCCAGGGGTGCATGGGGCT
GGCCCAGGCTCCCTGAAACCTGTCAGATGTGGTTGTTTAAACAAACCACCATCCCCTTCA
GGCACTCCTGTTTTTTGTTGTTTTGTTTTGTTTTTTAGCCTTGTTTTTTTCCTGCAGTCT
TCTGAGCCTGAGTTGCAGACTTCAGCAGCAATGTGGATGATCCTGGAAGCCACAGGGAGA
CGCACAG
ac015535.11 (226713-226467)

start 2
GCAAGCTGGGCTCCCCTGCTCCGCCGGGAAGCGGCTGCCCCTCCCTCTGGATCACACAGG
GTTCTTGCAGTAGGTGACAGCAGGATCGGGCACCAGGGAAGCAATTGATTCGTCTCTTAC
CCGCATCCCAGAGTTGCCTACGCTGCCCAGAGAGGGAGAGAGGAAAGAGAAGAAGGGAGA
GAAAGAAAGAGACTTACAAAACCTTCCATGTGGAGTAGTCTGGGTGATTCGCTGCCCACC
GCTGCAGACTCTAGCTGCACTTAGTTTTCGCCTCTGACGGG
ac015535.11 (226151-225871)

GCATATCTGCTGTTCTTTCAAGCCAAGTCGCTGGGACTTAACACCTCCCCCCCCCCCCAG
CAGGGAGTTGGCAAGAATTTGGCTCCCGTATTCTGCTGCAATCCTCTACTGTCCCG
ac015535.11 (225732-225617)

GTGTGCCAGCCTTCCACCATGACTGACACTGTGGTGAACCGATGGATGTACCCTGGTGAT
GGCCCTCTGCAGTCAAA
phase +2
ac015535.11 (203146-203070)

TGACAAGGAACA
phase +2
ac015535.11 (196774-196763)

GCTGCAGGCAGGATGGTCCGTCCATCCTGGAGCACAGACCGACAGGCAGAGGAAGCAGGA
AGAACTGACAGACGAGGAGAAGGAGATCATCAACAGAGTGATTGCTCGGGCAGAGAAGAT
GGAAGCCATGGAACAGGAACGCATTGG
phase +2
ac015535.11 (190077-189931)

GCGCCTGGTGGACCGTCTGGAGACCATGAGGAAGAATGTGGCTGGAGATGGCGTGAACCG
CTGCATTCTGTGTGGGGAACAGCTGGGTATGCTGGGCTCGGCCTGTGTCGTGTGTGAAGA
CTGTAAGAAG
phase 0
ac015535.11 (180586-180457)

AATGTCTGCACCAAGTGTGGGGTTGAGACCTCCAACAACCGTCCGCATCCGGTATGGCTC
TGCAAGATCTGCCTTGAGCAGAGAGAG
phase 0
ac015535.11 (179118-179032)

GTCTGGAAGCGCTCAGGAGCATGGTTCTTCAAAGGTTTCCCCAAGCAGGTCCTTCCACAG
CCCATGCCTATAAAGAAGACCAAGCCCCAGCAGCCTGCTGGTGAACCGGCCACCCAGGAG
CAGCCTACACCTGAGTCCAGGCATCCAGCCAGGGCTCCAGCTCGAG
phase +1
ac015535.11 (178024-177859)

GTGACATGGAGGACAGGAGGCCCCCAGGGCAGAAGCCAG
phase +1
ac015535.11 (176166-176128)

GCCCTGACCTCACCTCTGCTCCTGGGAGAGGAAGCCATGGGCCTCCCACGCGTAGGGCCT

CTGAGGCACGGATGAGTACAGCCGCCCGGGATTCTGAGGGCTGGGACCATGCCCATGGTG
GGGGTACTGGAGACACCAGCCGTAGCCCAGCAG
phase +1
ac015535.11 (176044-175892)

GTTTGAGGCGAGCTAACTCAGTCCAGGCAGCCCGCCCTGCCCCAGCCCCAGTGCCAAGCC
CAGCACCTCCTCAGCCGGTGCAGCCAG
phase +1
ac015535.11 (172616-172530)

GGCCCCCTGGGGGCAGCAGGGCCACTCCTGGGCCAGGACGCTTTCCGGAGCAGAGCACAG
phase +1 gc donor
ac015535.11 (172077-172018)

AGGCTCCTCCAAGTGACCCTGGCTATCCAGGGGCTGTCGCCCCAGCCCGAGAGGAGAGGA
CAGGACCTGCGGGGGGCTTCCAGGCAGCGCCGCACACTGCAGCCCCCTATTCCCAGGCAG
CCCCTGCTCGCCAGCCACCACCTGCGGAGGAGGAGGAGGAAGAAGCCAATAGTTATGACT
CTGATGAAGCAA
phase +1
ac015535.11 (171229-171038)

CCACACTGGGTGCCCTGGAATTCAGCCTTCTCTATGACCAAGACAACAGCAACCTGCAGT
GCACCATCATCAGGGCGAAG
phase 0
ac015535.11 (168774-168695)

GGACTGAAGCCCATGGATTCCAATGGCTTGGCAGATCCCTATGTGAAGCTTCATCTGCTG
CCTGGAGCCAGCAAG
phase 0
ac015535.11 (167019-166945)

TCCAACAAGCTTCGTACAAAGACCCTGCGCAACACTCGGAACCCTGTGTGGAATGAGACA
CTGCAGTATCATGGCATTACAGAGGAGGACATGCAGAGGAAGACACTAAG
phase +2
ac015535.11 (165481-165372)

GATCTCCGTGTGTGACGAGGACAAGTTTGGCCACAACGAGTTCATTGGTGAGACCAGGTT
CTCGCTCAAGAAGCTGAAGGCTAACCAGAGGAAAAACTTCAACATCTGCCTGGAGCGGGT
GATCCCG
phase 0
ac015535.11 (164607-164481)

ATGAAGAGAGCAGGGACCACCGGGTCGGCCCGTGGCATGGCTCTCTATGAGGAGGAG
phase 0
ac015535.11 (163741-163685)

CAGGTAGAGCGGATCGGCGATATAGAGGAACGGGGCAAGATCCTGGTGTCCCTCATGTAC
AGCACGCAGCAGGGCGGCCTCATTGTGGGAATCATCCGCTGTGTGCACCTGGCCGCCATG
GATGCCAACGGCTACTCAGACCCCTTTGTCAAGCT
phase +2
ac015535.11 (163139-162985)

CTGGCTGAAACCGGACATGGGGAAGAAAGCCAAGCACAAGACTCAGATTAAAAAGAAGAC
CCTGAATCCCGAGTTTAACGAG
phase 0
ac015535.11 (162102-162021)

GAGTTCTTTTATGATATCAAACACAGCGACCTGGCTAAAAAGTCCCTGGATATCTCGGTG
TGGGACTACGACATTGGCAAGTCTAATGATTACATCG
phase +1
ac015535.11 (159747-159651)

GAGGCTGCCAGCTGGGGATCTCGGCCAAAGGCGAGCGCTTGAAACATTGGTATGAGTGTT
TGAAGAACAAAGACAAGAAGATTGAGCGCTGGCACCAACTGCAGAACGAGAACCACGTGT

CCAGTGATTAG
ac015535.11 (158931-158801)

Mus musculus Doc2 alpha

MRGRRGDRMTINIQEHMAINVCPGPIRPIRQISDYFPRRGPGPEGGGGGGGTGCGEAPAH
LAPLALAPPAALLGATTPDDGAEVDSYDSDDT**^ +1**
TALGTLEFDLLYDQASCMLHCRILRAK**^ 0**
GLKPMDFNGLADPYVKLHLLPGACK**^ 0**
ANKLKTKTQRNTLNPVWNEELTYSGITDDDITHKVL**^ +2**
RISVCDEDKLSHNEFIGEIRVPLRRLKPSQKKHFNICLERQVP**^ 0**
LPSPSSMSAALRGISCYLKE**^ 0**
LEQAEQGPGLLEERGRILLSLSYSSRRHGLLVGIVRCAHLAAMDVNGYSDPYVK**^ +2**
TYLRPDVDKKSKHKTCVKKKTLNPEFNE**^ 0**
EFFYEIELSTLATKTLEVTVWDYDIGKSNDFI**^ +1**
GGVSLGPGARGEAQKHWNDCLHQPDTALERWHTLTSELPPAAGAYPLA

TGCTTTCTAGGATGGGGGTGTGAGTGGAAACCAGCGTCGCTATGGCAACCAGGCCGGGTG
GTCGGGCCCTGGAGCCCTGGGAGCCAGCGAAGGCGAGAGGCGGGGCAGCGGGAGGCGCCA
GGGCTGCGCGGCGGGGCGGGGGCCTGGGTCTCCCGGGAGGAGCTGGGGAACACCGGGCGC
CTCTCGCGGAGGTGCACGCCAAGTTCTCGGGTGAGTGTGCACGCGTGTGAGCTGGCCCAA
GGCTCACGGGCGGGCGTGAGCACGAGCACACGTGTCGAGTGTGCACTCGCGTGCGTTCTG
GGGCCCGTGTCTGGCCCTCTGCGCCCCGCGCCCCTCCCGGCCGCCCGCAGCAGCTCCCGC
CACTCCCGCCCCGCGCCGCTTCTCGCTCGGTCCTCGCCCGCCGCGCCGCAGTCGCCGCCG
CCGCCCAGCCTGCGCCCACGGGCCGCCCGTGCCGGGCACCTGTTGGAGGCTCAGGGACAG
ac124505.4 188321-188800

GACTGATCTGGCAACCCACCCAGCCCTTTGTGAAGCCAGGCCTCCTGCCTGCCCACCAGC
CAGCGCAGATCATCTTTTCCCTCGACACCCAGGAAGGGAGGGCAGTGAGGTTCCTACAGA
CCCCAGCCGGCCACTCCAGCTCTACACCCGTCTCCCAGTCAGAGGTGCTGCATGAGGGGC
CGCAGGGGCGATCGCATGACCATCAACATCCAGGAGCACATGGCCATCAACGTGTGCCCT
GGACCCATCAGGCCCATCCGCCAGATCTCCGACTACTTCCCTCGCAGGGGGCCAGGACCA
GAGGGTGGCGGCGGCGGCGGTGGCACGGGCTGCGGGGAAGCCCCAGCTCATCTGGCCCCT
CTGGCTCTGGCCCCCCCTGCGGCTCTCCTTGGGGCCACTACACCCGACGATGGAGCTGAG
GTAGACAGCTACGACTCGGATGATACCA
phase +1
ac124505.4 189183-189630

CCGCCCTGGGCACACTGGAATTTGACCTTCTCTATGATCAGGCTTCCTGCATGCTGCACT
GTAGAATCCTCAGGGCCAAG
phase 0
ac124505.4 189988-190067

GGCCTCAAGCCCATGGATTTCAATGGCCTGGCTGACCCCTATGTAAAGCTTCACCTCCTG
CCAGGAGCCTGCAAG
phase 0
ac124505.4 190250-190324

GCCAATAAGCTAAAAACCAAGACACAGAGGAACACACTGAACCCTGTGTGGAATGAGGAG
CTGACGTACAGCGGGATCACGGATGATGACATCACCCACAAGGTGCTCAG
phase +2
ac124505.4 190430-190539

GATCTCTGTCTGTGATGAGGACAAGCTGAGCCACAATGAATTCATTGGGGAGATCCGAGT
GCCCCTCCGCCGCCTCAAGCCTTCACAGAAGAAGCATTTTAACATCTGCCTTGAGCGCCA
GGTCCCG
phase 0
ac124505.4 191702-191828

CTTCCTTCACCCTCTTCAATGTCTGCGGCGCTGAGGGGCATATCCTGTTACCTGAAGGAG
phase 0
ac124505.4 191908-191967

CTGGAGCAGGCAGAGCAGGGACCTGGGCTGCTGGAAGAGCGCGGGCGCATCCTGCTGAGC

CTCAGCTACAGCTCTCGGCGGCATGGGCTGCTGGTGGGCATTGTTCGCTGTGCGCACCTG
GCTGCAATGGATGTTAACGGCTACTCTGACCCTTATGTGAAGAC
phase +2
ac124505.4 192051-192214

GTACTTGAGACCAGATGTGGATAAGAAATCCAAGCACAAAACATGTGTAAAGAAGAAGAC
ACTAAATCCGGAATTTAATGAG
phase 0
ac124505.4 192305-192386

GAATTCTTCTATGAGATTGAACTCTCCACTCTGGCCACTAAGACCCTGGAGGTCACAGTC
TGGGACTACGACATTGGCAAATCCAATGACTTCATAG
phase +1
ac124505.4 192477-192573

GTGGTGTGTCTCTGGGGCCAGGAGCCCGGGGAGAGGCCCAGAAACACTGGAATGACTGTC
TACATCAGCCGGACACAGCCCTGGAGCGCTGGCATACTCTGACCAGCGAGCTGCCCCCTG
CAGCAGGGGCTTACCCTTTGGCTTGA
ac124505.4 192659-192804

Mus musculus Doc2 beta

MTLRRRGEKATISIQEHMAIDVCPGPIRPIKQISDYFPRFPRGLPPTAAPRAPAPPDAPA
RSPAASASPRSPSDGARDDDEDVDQLFGAYGASPGPSPGPSPARPPAKPPEDEPDVDGYE
SDDC**^ +1**
TALGTLDFSLLYDQENNALHCTISKAK**^ 0**
GLKPMDHNGLADPYVKLHLLPGASK**^ 0**
ANKLRTKTLRNTLNPSWNETLTYYGITDEDMVRKTL**^ +2**
RISVCDEDKFRHNEFIGETRVPLKKLKPNHTKTFSICLEKQLP**^ 0**
VDKAEDKSLEERGRILISLKYSSQKQGLLVGIVRCAHLAAMDANGYSDPYVK**^ +2**
TYLKPDVDKKSKHKTAVKKKTLNPEFNE**^ 0**
EFCYEIKHGDLAKKTLEVTVWDYDIGKSNDFI**^ +1**
GGVVLGINAKGERLKHWFDCLKNKDKRIERWHTLTNELPGAVLSD

ATGACCCTCCGGCGGCGCGGGGAGAAGGCGACCATCAGCATCCAGGAGCATATGGCCATC
GACGTGTGTCCCGGCCCCATTCGGCCTATCAAGCAGATCTCCGATTATTTTCCCCGCTTC
CCGCGGGGCCTCCCCCCTACCGCCGCGCCCCGCGCCCCCGCGCCCCCGGACGCCCCCGCG
CGCTCTCCCGCAGCCAGCGCCAGCCCCCGCAGCCCCTCCGACGGCGCCCGCGACGACGAC
GAAGATGTGGACCAGCTCTTCGGAGCCTACGGAGCCAGCCCAGGCCCCAGCCCCGGCCCC
AGCCCCGCGAGGCCGCCCGCCAAGCCCCCCGAGGACGAGCCGGACGTCGACGGCTACGAG
TCAGACGACTGCA
phase +1
al669897.15 (81796-81424)

CCGCCCTGGGTACGCTGGACTTCAGTCTGCTCTATGACCAGGAGAACAACGCACTGCACT
GCACCATCAGCAAGGCCAAG
phase 0
al669897.15 (72075-71996)

GGCCTGAAGCCGATGGACCACAATGGACTGGCTGATCCCTACGTCAAACTACACCTGCTG
CCTGGAGCCAGCAAG
phase 0
al669897.15 (67598-67524)

GCAAATAAGCTCAGAACAAAAACTCTTCGGAACACCCTGAACCCCTCGTGGAACGAGACC
CTCACTTATTACGGAATCACGGATGAGGACATGGTCCGAAAGACCCTGAG
phase +2
al669897.15 (66267-66158)

GATCTCCGTGTGTGATGAGGACAAATTCCGCCACAATGAGTTCATTGGAGAGACTCGGGT
GCCCCTGAAGAAGCTGAAGCCCAATCACACCAAGACATTCAGCATCTGCCTGGAGAAGCA
GCTGCCG
phase 0
al669897.15 (66068-65942)

GTGGACAAGGCAGAGGACAAGTCTCTGGAAGAGCGAGGCCGCATCCTCATCTCCCTCAAG
TACAGCTCACAGAAGCAGGGCCTGCTGGTGGGCATCGTGCGCTGTGCACACCTGGCTGCC
ATGGATGCTAATGGCTACTCGGACCCCTATGTGAAAAC
phase +2
al669897.15 (63060-62903)

ATATCTGAAGCCAGATGTAGACAAGAAATCCAAGCATAAGACAGCAGTGAAGAAGAAAAC
ACTAAACCCAGAATTCAATGAG
phase 0
al669897.15 (59523-59442)

GAATTCTGTTACGAGATCAAGCATGGAGACCTGGCCAAAAAGACTCTGGAGGTCACTGTC
TGGGATTATGACATTGGAAAATCCAATGATTTCATCG
phase +1
al669897.15 (58522-58426)

GTGGTGTGGTTCTGGGCATCAACGCCAAGGGCGAGCGCCTGAAGCACTGGTTTGACTGCT
TGAAGAACAAGGACAAGAGGATTGAGCGTTGGCACACGCTCACCAATGAGCTCCCAGGGG
CTGTACTCAGCGACTGA
al669897.15 (57971-57835)

Mus musculus Doc2 gamma

MACAGPASGRQRVSMQEHMAIDVSPGPIRPIRLISNYFPHFYPFLEPVLRAPDRQAMLAP
AIPSAPQLQPNPEPEGDSDDS**^ +1**
TALGTLEFTLLFDEDNSALHCTAHRAK**^ 0**
GLKPPAAGSVDTYVKANLLPGASK**^ 0**
ASQLRTRTVRGTREPVWEETLTYHGFTCQDAGRKTL**^ +2**
RLCVCEDSRLRRRRRGPPLGELRVPLRKLVPNRARSFDICLEKRKL**^ 0**
TKRPKSLDTARGMSLYEE**^ 0**
EEMEAEVFGEERGRILLSLCYSSERGGLLVGVLRCVHLAPMDANGYSDPFVR**^ +2**
LFLHPSSGKKSKYKTSVRRKTLNPEFNE**^ 0**
EFFYAGHREELAQKALLVSVWDYDLGTADDFI**^ +1**
GGVQLSGRASGERLRHWRECLGHCDHRLELWHLLDSVPPQLGD

complicated 5'UTS exons and splicing

ag]GTGTGTGCTCGGTGCCCCCAGTAGGTGGGAGCTTCTCTGAGTCCCTATGTCTGGACC
ACCATGGCATGTGCAGGGCCAGCCAGTGGGCGACAGCGGGTGAGCATGCAGGAACACATG
GCCATCGATGTGAGCCCTGGCCCCATTCGGCCCATCCGCCTCATTTCCAACTACTTCCCA
CATTTCTACCCCTTCTTGGAGCCGGTGCTGCGTGCCCCAGATCGGCAGGCAATGCTGGCC
CCAGCCATCCCCTCTGCACCCCAGCTGCAGCCCAACCCTGAGCCCGAAGGAGACTCGGAT
GACAGCA
phase +1
ac109138.10 11929-12045

CTGCCCTAGGCACCCTAGAATTCACACTTCTCTTTGATGAGGACAACAGCGCCCTGCACT
GCACAGCCCATCGTGCAAAG
phase 0
ac109138.10 12363-12442

GGTCTCAAGCCACCAGCCGCCGGCTCTGTGGACACCTATGTCAAAGCCAACTTACTACCA
GGGGCCAGCAAG
phase 0
ac109138.10 12514-12585

GCCAGCCAGCTTCGGACACGCACTGTTCGAGGCACCAGGGAACCTGTCTGGGAGGAGACT
CTCACCTATCATGGCTTTACATGCCAGGATGCTGGACGGAAGACCCTGAG
phase +2
ac109138.10 12741-12850

ACTATGTGTGTGTGAGGACTCACGGCTGCGGCGCCGGCGGCGAGGACCCCCCCTGGGGGA
GCTACGAGTGCCCCTGAGGAAGCTGGTGCCAAACCGAGCCAGGAGCTTTGACATCTGTCT
AGAGAAGCGGAAGCTG
phase 0

ac109138.10 12931-13066

ACCAAGAGGCCCAAGAGCCTGGACACAGCTCGTGGCATGTCTCTATATGAGGAG
phase 0
ac109138.10 13181-13234

GAGGAGATGGAGGCAGAGGTGTTTGGGGAGGAGCGTGGCCGCATCCTACTGTCCCTGTGC
TACAGTTCCGAGCGCGGTGGCCTGCTGGTGGGTGTGCTACGCTGTGTTCACCTTGCTCCC
ATGGATGCCAATGGCTACTCAGACCCCTTTGTCCGCCT
phase +2
ac109138.10 14528-14685

GTTCCTGCATCCAAGTTCTGGGAAGAAATCCAAATATAAGACCAGTGTTCGGAGGAAGAC
CCTGAACCCTGAGTTCAATGAG
phase 0
ac109138.10 14776-14857

GAATTCTTTTATGCAGGTCATCGGGAGGAGCTAGCCCAGAAGGCACTGCTGGTGTCTGTG
TGGGACTATGACCTGGGCACAGCTGATGATTTCATTG
phase +1
ac109138.10 14970-15066

GTGGGGTGCAGCTGAGTGGCAGAGCCAGTGGGGAACGCCTGCGCCACTGGCGGGAGTGCT
TAGGCCACTGTGACCACCGGCTGGAACTGTGGCACCTGCTGGACAGCGTGCCCCCCCAAC
TTGGTGAC<u>TAG</u>
ac109138.10 15137-15267
