# Supplementary material for: Evolutionary genomics of plant genes encoding N-terminal-TM-C2 domain proteins and the similar FAM62 genes and synaptotagmin genes of metazoans
Source: BMC Genomics. 2007 Jul 31;8:259. doi: 10.1186/1471-2164-8-259 (PMC1976326; doi:10.1186/1471-2164-8-259)
Supplement: Additional file 5 — Other non-metazoan genes. [file 1471-2164-8-259-S5.pdf]

# Additional file 5 - non-metazoan genes

Taxonomy from NCBI

^ indicates an intron
complementary strand nucleotide positions indicated within parentheses

## **Saccharomyces cerevisiae Tricalbin genes.** These genes have no introns.

Eukaryota; Fungi; Ascomycota; Saccharomycotina; Saccharomycetes; Saccharomycetales; Saccharomycetaceae; Saccharomyces.

**Tricalbin1** Gene 125 Craxton, M. BMC Genomics. 2004 Jul 6;5(1):43
**Tricalbin2** Gene 124 Craxton, M. BMC Genomics. 2004 Jul 6;5(1):43
**Tricalbin3** Gene 123 Craxton, M. BMC Genomics. 2004 Jul 6;5(1):43

## **Neurospora crassa** gene with 5 C2 domains. 4 coding exons.

Eukaryota; Fungi; Ascomycota; Pezizomycotina; Sordariomycetes; Sordariomycetidae; Sordariales; Sordariaceae; Neurospora.

aabx01000033.1 48598..48789,48846..49150,49332..51876,51941..53263

MATQQSAQELKQQGAIEAAQDPNSSVTAENAEKKIVDESQQLGITAFTFDPNASAAEKAAQAR**^**
EAIPDGLRRPKGLAIASDLDDGTKADIDLPPPSKAGALDLPTTADGNVIEDGRTPEQIEADLIKKVGWAP
RFGWPKDPEEEQDLLDHATWVESNLSENYFGGT**^**
TSIRRVRRNVRDDITRELGLKKLETDNESVEWINSFLVKFWPIYQPVLAQTVINSVDQVLSMSTPAFLDS
LKLKTFTLGSKPPRMEHVKTYPKAEDEVVIMDWMFSFTPNDTADMTARQLQNKVNPKVVLEIRVGKAMIS
KGLDVIVEDMSFSGLMRLKIKFQMAFPHIEKVEMCFLERPKIDYVCKPLGGETFGFDINFIPGLESFIQE
MIHATLAPMMYAPNVFPIEVAKMLAGTPVDRAIGVVAITLHGAQGLKNPDKFSGTPDPYASLSLSKRQPL
AQTKVIKENDNPRWNETHYIIISSFNDSLDIDVFDFNEIRKDKKLGTASFPLENLEEINEFENERLELKY
DGKARGVVSCDIRFFPVLEETKLSDGTVEPPPESNTGILSFTVEQAKELDASKSMVGQLNPYGILLLNGK
EVHKTKTMKRTNQPIWPNGSKEILITDRKNAKLGVALKDDRDIAGDQLLGTYQIKLDDMLELMAKGQDWY
NLAGAKTGRVKMMAQWRPVAISGAAASTGGYSTPAGVLRLHFINARSLRNVEALGKSDPYVRVLLSGIER
GRTVTHKNNLNPDFDEVLYIPVHSAKERLQLDVMDAENMGRDRSLGLTEIFAGEYMHKDPETGEWLVHEK
KELHQDGLRMHGKGVAKGVLSYTVAFYPCLNVADPEDDEKDEQSDQSSEPKQSLDVPRSSDAGQFSSTLE
RTNSIKPENGGTSRPITPKTPVTPTSIAPSRKSRDEKEPPKLHLNPQELLQYESGLVIFKLMEAELPKSQ
CRVEVCVDDYAFPSYVSSTSRTKTHTFDEIGDCFIRELEFSKLTIKINERSEKQEEGGKENTLARLSGNT
LDTLKQCL**^**
NNPTTLKLKNEDGKVYSIKVSLKYVPVRMQLDPSESINNMGHLRVDVLDAQNLPSADSNGKSDPYVKFDF
NGQEVFKSKTVKKTLNPTWNEFFEVPVPSRTAASFKATVWDWDFADKPDFLGAADINLGQLEPFRAQEVR
LTLDGKSGVLRLRLLFRPDYVTRTRQGTSTLVGTFSVPGKIVTGVAGVPLKGGAAVAGAVGHGVGKGASF
IKRGFRSHTKRDDDSDSMTSSSVELPTIVTNAPEAASGLKRSAVITEGDGSSPNELRHARTRSVGASSVR
SAMLPGASSGTASFTVVSASGYPPSTDVYVTITQMKDGKSKAVGKTKHRKSSSGTVKFDETFNIQCTPDS
QFKIEAKEHHTFGSDDHLGESVYFVDESNSGQEKALKVGSGTVYIKSGFAPSESAQDASSALSPDQSPKS
SSMRRSFLSKGARSPSRDGP

## **Aspergillus nidulans** gene with 4 C2 domains. 6 coding exons.

Eukaryota; Fungi; Ascomycota; Pezizomycotina; Eurotiomycetes; Eurotiales; Trichocomaceae; Emericella.

aacd01000169.1 (284030-283824) (283756-283694) (283635-283383) (283333-280925) (280774-280727) (280674-279227)

MASQNAESAELKQQGAIETAQAASQDPQSHIQPETVEKKLVEETRKAGLPAYQFNPDASPQEKAAEAES**^**
CVPPGFHRDREPKAIGVITDK**^**
DDGTPAPYDLPPPKSATTLLDEKSQEPAADKKEEEVDDEQRWARDRTGWAPRFVHEETQEEHDEATLLDH
QTFLEAKLDEKFFG**^**
DWYHNAGVIVFACLSSWIVAVLGGGLAWVFIIMAACGTYYRTSIRRVRRNFRDDVNREMAKQRLETDTES
LEWINSFLVKFWPIYAPVMCDTIINSVDQVLSTATPAFLDSLRLKTFILGSKPPRLEHVKTYPKTEVDTV
IMDWKFSFTPNDTMDLTARQVKDKINPKVVLEVRVGKGVVSKGLDVIVEDMACSGLMRVKVKLQIPFPHI
ERVDVCFLGRPELDYVCKPLGGDTLGFDINFIPGLESFIKEQIHANLGPMMYEPNVFPIEIAKMLAGNAV
DQAIGVVAVTLHGARQLKNPDKFAGTPDPYAVVSLNNRTEVGRTKTIQDTDSPRWNETIYVIITSFTDTL
TIQPYDWNEFRKDKELGTATFALDKLEQEPEHESVYLEVLASGRSRGSIHADIRFFPVLEGRKLENGETE
PPPELNTGIARFTVEQAKDLDGSRSLVGQLNPYGVLLLNGKEIHITNKLKRTNNPIFQNASKEFLVTDRK
TARLGLVIKDDRDLVKDPILGSYQIKMNDMLKMMEKGHQWFHLHGAKSGRAKLVLDWKPVAVGGIAGSAG
YVDPIGVMRIHFKDAADLRNLEKMGKSDPYARVLLAGYMKGRTVTFRNNLNPDWDEVVYVPIHSAREKLT
LEVMDEESVGSDRSLGSVELSAADYVHENEAGEYEIDDEKQLVSSSLRLGQRAKGTLNYTVAFYPTIPVV
NPEDEEEEEEEAEAEAEAETEAAGLTRKSTDSRRKSYHSKSRSVDSKISDNKGATNGTADLQSNGRPSLE
SRSGTVKDSETASVRSIKEVPKTYISVDDLANH**^**
VWSSPKIRTKTAKIED**^**
IGDAFVRELEFSKITLRIVEKVNADTDDDEHIVAKLTGDTFSTLQRILYTPTELVLRSTDGEVSRVTVSA
RYIPVTMKLDPSESINNMGTLRVDVLDAADLPSADRNGYSDPYCKFRLDGKEIFKTKVQKKTLHPAWNEF
FETPIKSRIGANFRCDVYDWDFGDKADYLGGVPINLEMLEPFQAQEVSLTLDGKSGAIRLKLLFKPTYVM
RSRQGSSTFSGTFATPGKIVGAPVKGVGFVGGNVIKGASFLGRGIMSKIRGDDSSSDEEQEQQKEVPSAI
LVDGETPPNSTPKSDLNHSRTRSVAASHYGDRLSIGGKGESGTARISIVSAGGYPPSANVRVLVRALGAK
GAKEVLKTKAIKSGGGPVVFDSASESCRVHNTTADAQYQIRVVDHSTFGSDNVLGEAAFFVGDQGSVAGQ
EKTVNVGSGTVVVRSSFSAAGDSLRPGTAYSNAGDNASEVTDSPDSKKPRRSFLSKRSVSGA

## **Trypanosoma brucei** gene with 1 C2 domain. No introns.

Eukaryota; Euglenozoa; Kinetoplastida; Trypanosomatidae; Trypanosoma.

Gene 126 Craxton, M. BMC Genomics. 2004 Jul 6;5(1):43

## **Ostreococcus tauri genes.** These genes have no introns.

Eukaryota; Viridiplantae; Chlorophyta; Prasinophyceae; Mamiellales; Mamiellaceae; Ostreococcus.

**NTMC2.1** This has Nterm TMs and 2xC2. No closely matching ests. Top hits are plant NtermTM-C2Type2 and Type4 genes.

cr954203.2 825947-828739

MGVCSSKSGAAPEAQETEFRLRKTADGRRLGDDSTKNLSAFAEDSGMSVGSEREVTRGEP
IASLRPQTRNQEISKKDFKTKKKNAIAVEDDQVLEGDDDDVYRFDQSLLNSARAAMNEDE
DADQNRSLLRRQSIVKAPNQTVAKPPVELSERLKRYEAEPKLKANELPVSYGPEWLAPPD
IQRETTVRKPKKYSIGNLARGDHKLEPTNLTDKDFSDYAPGNGPWFNGPFPSMMASSAKQ
MGLAYGKFCVVGVITLFWVPNFLLYHRLLMNGTFQAYSRLLSAILSSLFMTRTVLNMNME
LCRMFWRGSIVNSQVICSLIGAVGFFWQTLVIFTVGWLSDNRGIFSFLIAFGVGWFIVWR
QDQRHEKQQRIRTVMGAFLALEKDAKHMAQLMGSPVVRTNDIQYMNAAPVWARYRPDELV
PWLNNFLTQVWPFYNKAASELVREIVEPLMEQSRPSMLKRLTFKQLDFGENPFMVRSVSY
VGKKAEDKGMSLDIDFAWAGRSNIVLAAKTHIGADINIAVKDLEIYTKLRVTLNPLVPLP
SPLGGVVISMTERPIVEFHVELPSGLDVLYAAIDKWLEEFVAGLLGDMFIQPERLVIPLS
FNFDPIVMPDGEVKPFKWYDHNVLQLRNTGVLKVTVVRAENVPSADLLSKTDPFVKMFVK
KHGLQVNTTTIMNNEDPVWNEIFYIPVDDVDLRVLKVAMYDHDVDPLSSDDKLGATEVRI
DTIKAATADGSEQELWLDFPEQVKGNVKKPPMKLLLNAQFISFGSDIAQNMFTGLGLLSV
HVIRGRNLQPMDSNGLSDPYVKVKVPKFTLDSMDMDKGKILRGKRGKKGKKNAEAHDYTV
YSSKIHYKNLNPEFNAMFEFSPASEDTKVSIELFDVDSTFPMGTKSKFMGNLEVPISTII
HHGGSMEARFKVGNAKSGELDIAFNWQPYT

This has linker but no C2. No closely matching ests. Top hits are plant NtermTM-C2Type2 and Type4 genes.

cr954202.3 272766-274421

MVDLRVQNTQRYRRKCCALRLKRRKTPNTFHVCPQRKKTDTKLLGLTRSLGLTRSIPVST
ELLPVSQLLDPVFSHENFKHVEYSQSFLLIEGFVTGAVFTSIFLNQAERWRKYKPNNGIT
SALRSTIDYLEAQGPSGDFPKQLGRDVQSFRWLNEVVKVAWPYLDAATSAVIVSALDPIL
QNTRPSFLTSIEFERFSFGSVPAIIEAVKVYEAGNEGALEIDLHVFWAGDPDVVLKIRAA
QAALAVPVSLTEFECTFTLRMIFAPLIGTFPCFGALTLSLTEDPVVKFDLRVVGGDITLL
PGLAQPLRTYIQALIASFLVWPRCITVPIPSTGYSLPDRESANAGLLHVEIHSHNDSVGS
PAEIALQLRWPGTSGSNASQEVRLQASPSGSFFNSREVTLPVEDTTRQILSVRWYTSDGN
TEEKGKIVGETSLLLDDIVQQLKADTHEDYKNWGPITIAAELESVLSTELSEEKTSKTTD
TVYGRVTKLGTGLFSRIRRASSNVSSKFVADAKYDASFDHSNASTRIVQLTVRYQSLDSL
QVAGLEDNAEL

**NTMC2.2** This has linker and 1xC2. No closely matching ests. Top hits are plant NtermTM-C2Type1 genes.

cr954213.2 318056-321133

MGSTRSREKIYQRQRAVSFALAREDDGVLGRRVSSRDGRVDDARRLLQATAQDRSIPFLH
LSTRERARERRQPLPRLRAHQNPTRGLVQTMTRFRFRQHAKIRRAHLARYRQHPLRAPPS
TAPRSDRRAIVRFHRDRRVRVFVQHHHLPRASFLFEPQLSARPPSRDVSRQHLEHVPRRD
AIAASYDLTVSRDRLPSARPLRARRRRKRFRKHHRERPRHRVSVARVVHDHRLPRDVRRG
RLPRRPRPSRRRRRLARARHRARASHARVCGRRHLSPVTRDPLSPFTGDLSTPVDACRRL
STGGVSDRIDRPSRDRRARDDLASRVGRRDDERASTTMDLVRDLARAARTRVRTTVVENA
STIIELDRGRVVDATATAGATGRGTVGFVMYVVVYLARTILGQIAVAVGTIVLGFAMGTT
CARATRSDGAKWMRTAMKKRMGRQATREVTWIEKKRAERGLGGEDEDEITFHEDFKETFS
THAPIWTKDSSYSRAHWLNRVIDGAWPYIDTGVSKTVKESVEPILRELLPTWVTWIGFEK
FTLGPRAPTITGIRSHQSHMENSILDIELSWASDCDVVVTIYVFGVRFPVTVRGLQIKML
AQVTFDPLVDVIPCLGALEACLMEMPEILDFRLFIPGGVDLLALPFVHRTVLKIVRQSIG
EMLLYPYKLHIPIMPASGIQAASTGMMRIRFLNGKAFYKRRNYSKLSRKRGKKNSRFTQM
LKTDSYFIKYWTREQRQLSTPPRSGETPSWEGTADAFVLCDRDTPLYFRLLKEGADRISN
YGEIQIMCGEIADRGGGKVVIELPFIEPSFYKEECPLEYLAAADGYSYEEIMDRWAEITA
WHAKADAGAVQARTKYFYRCLEEMDRRKGVISKYRHPTLALELEYIDTGAPDELDDGDEN
YELGVLTVEVKEADNLLRVDSQLPNPMATLRCAKQEYCTQRILKSSHPKWNERYVFYNVV

AEIDPLEIEITGFEKSLGRVVIDTTLVRLNGLISDRFKLQDVSKGEVLLELSYTPMAAKK
TIARP

## **Cyanidioschyzon merolae** gene with no introns.

Eukaryota; Rhodophyta; Bangiophyceae; Cyanidiales; Cyanidiaceae; Cyanidioschyzon.

This has 1 C2 domain. No closely matching ests. Top hits are plant NtermTM-C2Type5 genes.

ap006488.1 (132604-130172)

MERQVPCAVGSVQVGTNAPMERHSHSQVSVCFCTGLGPMYEAVSRGRGTRKPQFSVHGKR
RPQSRRRGPFTWLRGALPLDALLPHQELQHRQQHFRRHSFSTCQGMKRTVSVPRQSEQCT
RLPSVVADATGQRTRATWTRPQRSSHKSIVVCFLDGKRTFRKASRTGFFNPSLMFSQTES
RIKTWWTQTLIQIRSFTDEVSKRFADARRRQPVDPAAANRPPLYPDGLYVLLGMWILTYL
VVRLGQFVCDLLAFIVAIVVSVFYVPGQARQRRAKKTAELQVDAATSADGGRGAIPEDSA
SMNTLGSSSAARERQRLQAIAARETTEAESAEWINAAIRKMWRLYNTELSVTGKMILQDL
IDANLKNNRPPFVQSVTVERLELHERALRLPSVEKLPTRSDGDLVLLVGVRYDGDAQLHL
RVNFGVTQRASFAVPVVVSGLDIDSQLWIRARMIPEAPYLGDVNVALLRRPLIDLQLRPF
KVVDVMEIPGLRPFLRKLLTCDIPDLFVLPRRMPILRLSSLEQLQRYARMGAWDGSRNDP
CMGALTKARFRRRSESAAPGRDDLSTDDLSDAGESEPDILDDPSLDDGLLIVMLYGARNL
SGTTSLGLSNPFCYISVDGITVRSKPDKSTSARSVRGQPIWNQLFELPVRNPNTARLHIE
VADRYGLKHRIIGAFSMAVSALRDGQRRDMWVPLRGSVAAESRLHIGVQYQAYVDADNDD
LLLLSSSSSSQALRSKAPNIDSIVSMTSLTRMFPLGVPRSSSTSAASAHTVDRDVHVDEA
NEQGVQASNHTTEENVTPVQVEVIQPPEKR
